# UHRF1-repressed 5’-hydroxymethylcytosine is essential for the male meiotic prophase I

**DOI:** 10.1038/s41419-020-2333-3

**Published:** 2020-02-21

**Authors:** Hongjie Pan, Ning Jiang, Shenfei Sun, Hanwei Jiang, Jianze Xu, Xiaohua Jiang, Qian Gao, Liang Li, Haili Wu, Huajun Zheng, Qi Qi, Tianqi Li, Meixing Zhang, Lingling Zhang, Xiaofeng Wan, Xinhua Lin, Jiemin Wong, Qinghua Shi, Runsheng Li

**Affiliations:** 1 National Health Commission (NHC) Key Laboratory of Reproduction Regulation (Shanghai Institute of Planned Parenthood Research), Fudan University, 200032 Shanghai, P.R. China; 2 State Key Laboratory of Genetic Engineering, Institute of Biostatistics and Computational Biology, School of Life Sciences, Fudan University, 200438 Shanghai, P.R. China; 3 The First Affiliated Hospital of USTC, Hefei National Laboratory for Physical Sciences at the Microscale, The CAS Key Laboratory of Innate Immunity and Chronic Diseases, School of Life Sciences, University of Science and Technology of China, 230027 Hefei, Anhui P.R. China; 4 East China Normal University and Shanghai Fengxian District Central Hospital Joint Center for Translational Medicine, Shanghai Key Laboratory of Regulatory Biology, School of Life Sciences, East China Normal University, 200241 Shanghai, P.R. China; 5 Shanghai Endangered Species Conservation and Research Centre, Shanghai Zoo, 200335 Shanghai, P.R. China

**Keywords:** Disease model, Mouse, Spermatogenesis, Epigenetics, Infertility

## Abstract

5’-hydroxymethylcytosine (5hmC), an important 5’-cytosine modification, is altered highly in order in male meiotic prophase. However, the regulatory mechanism of this dynamic change and the function of 5hmC in meiosis remain largely unknown. Using a knockout mouse model, we showed that UHRF1 regulated male meiosis. UHRF1 deficiency led to failure of meiosis and male infertility. Mechanistically, the deficiency of UHRF1 altered significantly the meiotic gene profile of spermatocytes. *Uhrf1* knockout induced an increase of the global 5hmC level. The enrichment of hyper-5hmC at transcriptional start sites (TSSs) was highly associated with gene downregulation. In addition, the elevated level of the TET1 enzyme might have contributed to the higher 5hmC level in the *Uhrf1* knockout spermatocytes. Finally, we reported *Uhrf1*, a key gene in male meiosis, repressed hyper-5hmC by downregulating TET1. Furthermore, UHRF1 facilitated RNA polymerase II (RNA-pol2) loading to promote gene transcription. Thus our study demonstrated a potential regulatory mechanism of 5hmC dynamic change and its involvement in epigenetic regulation in male meiosis.

## Introduction

Meiosis, characterized with a single round of DNA replication followed by two successive divisions, fosters genetic diversity and allows the total DNA content to be maintained through successive generations. In male, meiosis is an essential process of spermatogenesis. Meiotic disorder is one of the major causes of male infertility^1,2^. The progression of meiosis is highly guided by epigenetic transitions^3^.

DNA modification, one of the important epigenetic mechanisms that influence spermatogenesis and male fertility, associates with meiosis functionally^4–6^. DNA methylation, typically in the context of CpG in the promoter regions and constitutive heterochromatin, is generally associated with reduced gene transcription and silencing^7–10^. Methylation of cytosine at the fifth carbon can be converted into 5’-hydroxymethylcytosine (5hmC), through consecutive oxidation reactions catalyzed by ten-eleven-translocation 1, 2, and 3 (TET1, 2, and 3) enzymes. Global detections of 5hmC in human, mouse, zebrafish, and Xenopus^11–19^ suggest that the dynamics and abundance of 5hmC are cell-type dependent and developmentally regulated. In mice, germ cells are first specified as primordial germ cells (PGCs) in the developing embryo around embryonic day 6.25 (E6.25)^20^. The first global DNA demethylation takes place in PGCs to reset the epigenome for totipotency. Epigenetic reprogramming (including 5-methylcytosine (5mC) and 5hmC) enables the transition from PGC to gonocyte^21^. In addition, 5hmC takes roles as a *cis* element promoting or repressing gene expression, because it can be localized at enhancer, promoter, transcriptional start site (TSS), gene body, 3′ untranslated region (UTR), or intragenic regions. Although the 5hmC alters in a highly order in germ line during spermatogenesis^22^, to date, the underlying molecular mechanism and physiological function of 5hmC in germ cells after the PGC period remain not well understood.

A previous study showed that the 5mC in differentiating spermatogonia was higher than that in undifferentiating spermatogonia. This suggested that spermatogonia underwent a KIT^neg^ to KIT^pos^ transition accompanied with an enhancement of DNA methylation. They also pointed out that the DNA methylation might regulate directly or indirectly the genes guiding spermatogonial differentiation (for example, *Plzf* and *Kit*)^23^. One of the key player of DNA methylation is UHRF1, a multi-functional protein^24–26^, which is essential for maintenance or de novo DNA methylation^27–34^. However, the role of UHRF1 in male germ cells after the spermatogonia differentiation, for example, meiosis, remains largely unknown. A structural analysis showed UHRF1 interacted directly and to a similar degree with 5mC or 5hmC^35^. Although it has been well established that UHRF1 mediates DNA methylation, its function linking to 5hmC is still in its infancy.

Here we showed that UHRF1 controlled the meiosis of male mice. Knockout of *Uhrf1* in mice resulted in a serious of impairment of meiotic events, like deficient synaptonemal complex (SC) assembly, skewed meiotic recombination, and failed H1t incorporation. In this study, we identified a previously unknown role that UHRF1 repressed 5hmC and facilitated the RNA-pol2 binding to DNA. Benefiting from conditional knockout mice, we provided a potential regulatory mechanism for the 5hmC dynamic change and demonstrated the physiological function of 5hmC in meiosis.

## Materials and methods

### Mice

The C57BL/6 *Uhrf1-flox* mouse strain was prepared as mentioned by the method of gene trap. The C57BL/6 *Stra8-cre* mouse strain, which expressed knock-in CRE recombinase driven by endogenous *Stra8* promoter^36^, was provided as a gift by Professor Minghan Tong at the Institute of Biochemistry and Cell Biology, CAS. All mice were kept under the controlled photoperiod conditions (lights on 07:00–19:00 hours) and supplied with food and sterilized H_2_O ad libitum. All experiments were conformed to the regulations drafted by Association for Assessment and Accreditation of Laboratory Animal Care in Shanghai and were approved by the Shanghai Institute of Planned Parenthood Research Center for Animal Research. To generate the *Uhrf1*^*f/f*^*;Stra8-cre* male mice, we intercrossed female *Uhrf1*^*f/f*^ and male *Uhrf1*^*f/+*^*;Stra8-cre* mice. Genomic DNA was extracted from tail biopsies and analyzed using the TaKaRa Taq™ Hot version (Cat# R007A). Primers for genotyping were listed in Table S1.

### Cell, plasmids, and transfection

GC1 cells (ATCC, Cat# CRL-2053) were cultured in high-glucose Dulbecco’s Modified Eagle’s Medium (DMEM) (GIBCO, 11965–092) with 10% fetal bovine serum (GIBCO, 10437028), 100 U/mL penicillin, and 100 mg/mL streptomycin (GIBCO, 15140122) and maintained at 5% CO_2_. Plasmids (pcDNA3.1-*UHRF1*-*Flag*, pcDNA3.1-_*m*_*UHRF1-ΔSRA-Flag*, pcDNA3.1-_*m*_*UHRF1-ΔTTD-Flag*, and pcDNA3.1-_*m*_*UHRF1-ΔRING-Flag*) were from Jiemin Wong’s laboratory. Cells were passaged 2–3 times after thawing and transfected at 70–80% confluency. Transfection of plasmids was performed using lipofectamine 3000 (Invitrogen, L3000001). Detection of mycoplasma in GC-1 cell line using polymerase chain reaction (PCR) methods was performed as previously described^37^.

### Hematoxylin and eosin (H&E) staining and immunohistochemistry (IHC)

Testes were collected and fixed immediately in Bouin’s solution for H&E staining and in 4% paraformaldehyde (PFA) for IHC. For IHC assay, sections (4–5 μm) were deparaffinized in xylene and rehydrated in gradient alcohols. After antigen retrieval, the sections were denatured or not with 2 N HCl, at 37 °C, for 30 min, followed by blocking. Sections were then incubated with primary antibodies overnight at 4 °C. The sections were incubated with secondary antibodies for 20 min the next day and then developed with 3,3′-diaminobenzidine and counterstained with hematoxylin. Antibodies were diluted as follows: 5-mC at 1:100 (Active Motif, Cat# 39649), 5-hmC at 1:100 (Active Motif Cat# 39769), TET1 at 1:100 (Millipore Cat# 09–872), TET2 at 1:100 (Millipore Cat# ABE364), TET3 at 1:100 (Millipore Cat# MABE1133), and UHRF1 at 1:200 (Active Motif, Cat# 61341). To ensure reproducibility of the results, samples from ≥3 animals were evaluated.

### Meiotic prophase cell spreading and immunofluorescence staining

Spreads of spermatocytes and immunofluorescence staining were prepared according to the previous references^38,39^. Briefly, seminiferous tubules were incubated in hypotonic extraction buffer (50 mM Sucrose, 17 mM Sodium citrate, 30 mM Tris (pH 8.2), 2.5 mM dithiothreitol, 1 mM phenylmethylsulfonyl fluoride (pH 8.3), and 5 mM EDTA) on ice for 20 min, minced in 100 mM sucrose, spread on slides, and fixed in 1% PFA with 0.1% Triton X-100. Slides were incubated in a humid chamber overnight, dried, and washed in phosphate-buffered saline (PBS) and water containing Photoflo (Kodak, NY, USA). Following blocking in 10% donkey serum and 3% bovine serum albumin, immunofluorescence staining was performed by incubating with the primary antibodies: γH2AX (1:1000; Novus, Cat# NB100–384), SYCP3 (1:100; Abcam, Cat# ab97672 or Novus, Cat# NB300–232), DMC1 (1:100, Santa Cruz, Cat# sc-22768), MLH1 (1:100, BD, Cat# 551092), SYCP1 (1:100, NOVAS, Cat# NB300–229), and RNA-Polymerase II (1:200, Active Motif, Cat# 39097), overnight at room temperature. Alexa 488 (1:400, Thermo Fisher) or Alexa 555 (1:200, Thermo Fisher) fluorescent secondary antibody was used. Slides were incubated with secondary antibodies at 37 °C for 1 h in dark, washed, and mounted with Vecta shield cover slips (Vector Laboratories, Cat# H-1000).

### Primary germ cell preparation

Testicular cells were obtained as previously described^40^. Briefly, the capsules of the testis were removed and testicular tubules were minced and transferred to a 50-mL Falcon tube. Tissue was suspended in F12/DMEM (Gibco), centrifuged, collected, and then subjected to digestion. Trypsin/EDTA (0.1 mg/mL; Sigma), DNase (0.02 mg/mL; Sigma), glycine (1 M; Sigma), EDTA (2 mM; Sigma), and STI (0.1 mg/mL; Sigma) were used to eliminate Leydig cells. Collagenase IV (0.1 mg/mL; Sigma) and DNase (5 μg/mL; Sigma) were used to reduce peritubular cells. Then testicular cells were washed and plated in medium with gentamicin (0.02 g/L; Sigma) and maintained in a humidified atmosphere at 34 °C with 5% CO_2_ for 6–8 h. Germ cells were harvested by shaking and suction gently^41^. The residual aggregate consisted of sertoli cells. For the subgroup meioitc prophase I spermatocytes, the method of STA-PUT was applied, and cells were isolated according to the diameters: leptotene, 8–10 μm; zygotene, 10–12 μm; and pachytene, 14–18 μm.

### RNA isolation and real-time PCR

Total RNA was extracted from the control and *Uhrf1-cKO* mouse spermatocytes (leptotene/zygotene stage and pachytene stage) were isolated. The primary cells were homogenized in TRIzol reagent (Invitrogen), followed by RNA precipitation. cDNA was synthesized from 1 μg RNA with a reverse transcription kit (TaKaRa). Real-time PCR was performed using SYBR Premier EX Taq (TaKaRa). Relative levels of mRNAs were normalized to the levels of endogenous β-Actin in the same samples. Genes were amplified with the specific primers (Table S1).

### Western blot

Sixteen day post-partum (dpp) control and *Uhrf1-cKO* mouse tissue extracts containing 30 μg proteins were resolved by sodium dodecyl sulfate–polyacrylamide gel electrophoresis and transferred to nitrocellulose (NC) membrane (Millipore Corp). After probing with primary antibodies, the membranes were incubated with secondary mouse or rabbit antibodies (1:2000). The primary antibodies used were UHRF1 (1:1000, Active motif, Cat# 61341) and β-Actin (1:10,000, Abcam, Cat# ab8227).

### Hydroxy-methylated DNA IP (hMeDIP)-qPCR

Genomic DNA was extracted with the PureLink Genomic DNA Mini Kit (Thermo). Purified Genomic DNA was sonicated to an average size around 200 bp (range: 100–500 bp) with a bioruptor (Diagenode). DNA fragments were end-repaired. A-tailed and custom TruSeq adapters were ligated using the TreSeq DNA sample preparation Kit (Illumina). The DNA fragments ligated with adapters were immunoprecipitated with Protein A+G magnetic beads coupled with 5mC or 5hmC antibody. The purified DNA samples were then ready for the qPCR test. The specific primers were listed in Table S1.

### Bisulfite sequencing

Bisulfite conversion was performed using the EpiTect Bisulfite Kit (Qiagen) according to the manufacturer’s protocol. Bisulfite-treated DNA was then used to amplify. The amplified regions were cloned into pEASYT1 (TransGen Biotech) and sequenced. The primers for PCR amplification were listed in Table S1.

### Dot blot assay

Genomic DNA samples were heated at 95 °C for 10 min and allowed to cool on ice, then the ice-cold 20× side scatter (SSC) was added. Meanwhile, an NC membrane and two filter papers were wetted with 6× SSC and then mounted on a 96-well dot blot apparatus. To the wells to be used, 500 μL of water was added and pulled through the membrane with gentle vacuum pressure. Subsequently, the diluted samples were added and pulled through. The wells were then washed with 500 μL of 2× SSC solution. The membrane was next allowed to air dry before ultraviolet DNA cross-linking for 5 min at 100 μJ/cm^2^. In all, 5% milk was applied for blocking for 1 h, at room temperature. Then the membrane was incubated with 5hmC antibody (1:3000) for 3 h at room temperature. After three times washing with PBST (5 min each) and incubation with secondary antibody (1:2000) for 1 h, the samples were developed with ECL. The density was calculated by the software of ImageJ 1.52a.

### Quantification and statistical analyses

#### RNA-seq

For RNA sequencing, four *Uhrf1*-cKO and control mice were sacrificed for isolating spermatocytes (leptotene/zygotene and pachytene stages) by the method of STA-PUT. To prevent cross-contamination, the leptotene/zygotene stage spermatocytes were isolated from 12 dpp mice and the pachytene stage spermatocytes were isolated from 16 dpp mice. The indicated spermatocyte pools were then subjected to library construction. Libraries were prepared using NEB Next Ultra Directional RNA library preparation kit for Illumina. Quality control was carried out with a Bio-analyzer (Agilent), and 150-base-pair (bp) paired-ends sequencing was performed with a HiSeq X-ten sequencer (Illumina). For each sample, the RNA-seq data was mapped to mm10 genome by TopHat v2.0.7 with no more than two mismatches, and then only the uniquely mapped reads were used to estimate the expression values in gene level by RPKM (reads per kilobase of transcript, per million mapped reads) with featureCount (V1.5.3). Statistical significant test of differentially expressed genes (DEGs) was performed by DEGseq with R. Genes with absolute log_2_-transformed fold changes >2 were regarded as DEGs and a threshold of *p* value < 0.001 was used. DEGs were identified as significantly differential expression in either leptotene/zygotene or pachytene stages. Hierarchical clustering of log_2_-transformed RPKMs was generated by Cluster 3.0 and visualized by Java TreeView. The raw next-generation sequencing (NGS) data were deposited to the NCBI SRA database under accession number (SRP201556). Some RNA sequencing results were verified by real-time PCR.

#### MeDIP-seq and hMeDIP-seq

The MeDIP and hMeDIP sequencing were performed as previously mentioned^42,43^. The meiotic prophase spermatocytes from 6 to 8 *Uhrf1*-cKO and control mice (16 dpp) were prepared as aforementioned. The antibodies used for immunoprecipitation (IP) were 5mC and 5hmC (Active motif). Mouse or Rabbit IgGs were applied for nonspecific IP experiment as control samples. DNA libraries were generated using the NEB Next Ultra DNA library preparation kit for Illumina. Quality control was carried out with a Bioanalyzer (Agilent). Sequencing was performed on Illumina HiSeq X-ten sequencing platform. Sequencing reads were aligned to the reference genome (mm10) using Bowtie2 v2.3.3.1 with no more than two mismatches, and then only the uniquely mapped reads were used for peak calling analysis and mapping depth analysis. The mapping depth was normalized by the total mapped reads for each sequenced sample. The measurement of 5(h)mC level (density) was only summarized with the normalized mapping depth of CpG sites in mouse reference genome (mm10). The peaks detection was performed by MACS V1.4.2 with default cut-off. Peaks were assigned to the nearest genes using Homer V4.8.2. The raw NGS data were deposited to the NCBI SRA database under accession number SRP201555.

#### Statistical analysis

All statistical data were analyzed with GraphPad Prism version 5. The statistical data of litter size, testis weight, numbers of spermatocytes with the indicated meiotic biomarkers, and RT-PCRs were presented as means ± SEM. Analysis of variance or Student’s *t* test were used for statistical comparison to determine significance. Statistical significance was set as: NS, *p* > 0.05; *p ≤ 0.05; **p ≤ 0.01; ***p ≤ 0.001. All presented results were from at least three independent experiments. The investigators were blinded to the group allocation during the experiment and when assessing the outcome.

## Results

### The distribution of UHRF1 in male germ cells

**Fig. 1:**
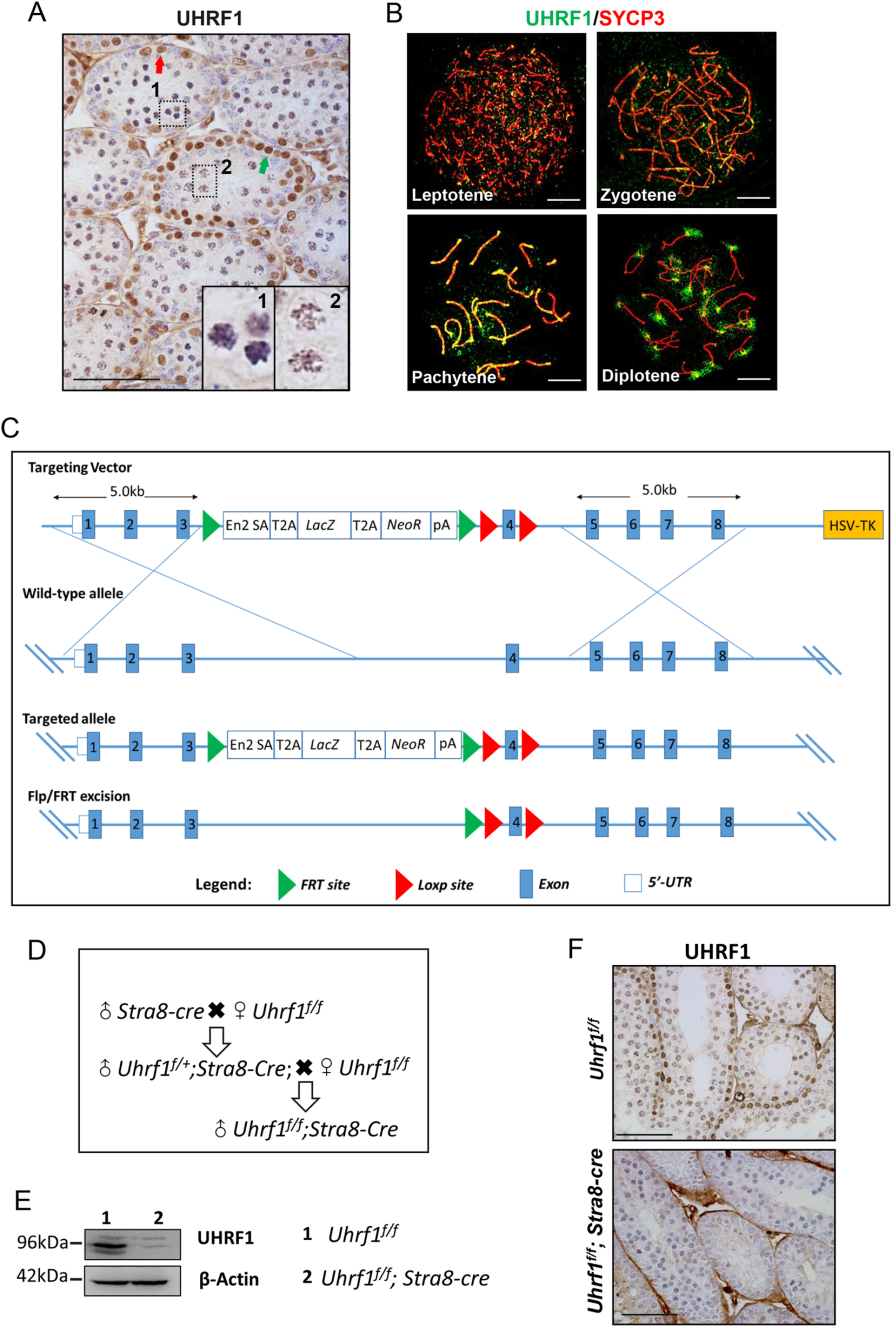
UHRF1 was expressed in the meiotic prophase I and the gene-trapped *Uhrf1* conditional knockout mouse was prepared. **a** The expression of UHRF1 protein in the mouse testicular tissue. **b** The distribution of UHRF1 protein (green) in the sub-stages of mouse meiotic prophase. **c** Schematic diagram of *lox P*-deletion system in the *Uhrf1* gene in mouse; **d** mouse strategy; **e**, **f** western blot and immunohistochemical assay to the 16 dpp mouse testicle tissue showing the knockout efficiency of UHRF1 protein. At least three independent experiments were carried out. Scale bar, 25 μm in **a**, **f**, 5 μm in **b**.

The initial experiments were aimed to examine the expression pattern of UHRF1 in germ cells across the male meiotic prophase I utilizing an IHC assay. The UHRF1 protein was detected within the entire nuclei in spermatogonia (arrowed in red) and preleptonema/leptonema (arrowed in green) but started to concentrate on the chromosomes from zygotene stage (Fig. 1a and enlargement). Note that the enlargements 1 and 2 showed the expression of UHRF1 in the zygonema and pachynema/diplonema, respectively. Next we analyzed the precise location of UHRF1 in testicular spreads. Figure 1b showed an alteration of UHRF1 expression from leptonema to diplonema. The expression of UHRF1 was scattered in leptonema and zygonema but condensed in pachynema and diplonema. The shift of the UHRF1 expression pattern from a random to condensed appearance implied that *Uhrf1* exerted different physiological roles in the murine prophase meiosis I.

### Ablation of UHRF1 in germ cells caused male sterility with impaired testis development

To analyze the physiological role of UHRF1 in male meiosis, a conditional knockout mouse strain was established. The *Stra8-cre* tool mouse was used to delete the floxed exon4 of *Uhrf1* (Fig. 1c, d). Western blot assay showed that the level of UHRF1 was reduced apparently in the *Uhrf1*^*f/f*^*;Stra8-cre* mouse testicle tissue. IHC analysis showed the dramatic loss of UHRF1 in the *Uhrf1*^*f/f*^*;Stra8-cre* mouse germ cells compared with the *Uhrf1*^*f/f*^ germ cells (Fig. 1e, f). These results indicated that UHRF1 was deleted efficiently in germ cells.

**Fig. 2:**
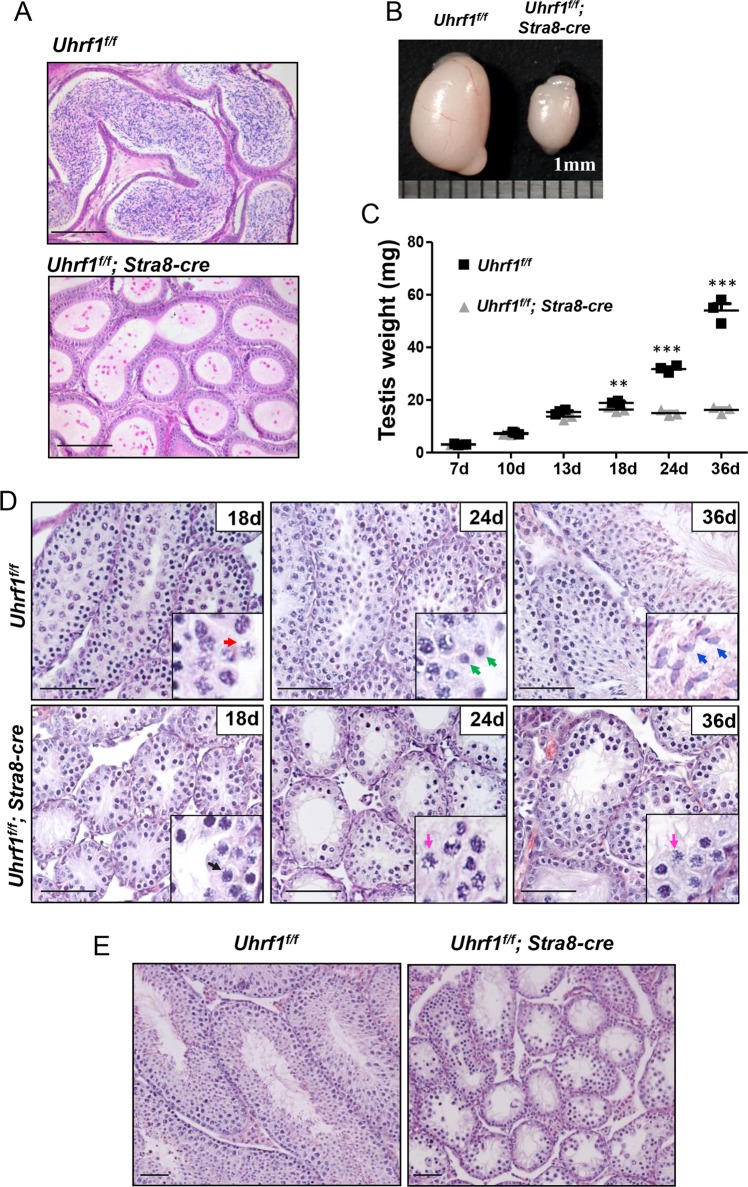
UHRF1 was required for the mouse spermatogenesis and fertility. **a** H&E staining of epididymal sections from 12-week-old *Uhrf1*^*f/f*^*;Stra8-cre* and *Uhrf1*^*f/f*^ mice. **b** Morphology of testes. **c** The mean testicular weights of the mice from 7 to 36 dpp. **d** H&E staining of testicular sections at 18, 24, and 36 dpp. **e** Histology assay showing the testicular tubes in the adult *Uhrf1*^*f/f*^*;Stra8-cre* and *Uhrf1*^*f/f*^ sections. Scale bar, 25 μm. dpp day post-partum. At least three independent experiments were carried out, data are presented as mean ± SEM of three mice in **c**. ***p ≤ 0.001; **p ≤ 0.01; NS, *p* > 0.05.

The UHRF1-deficient male mice were apparently normal in growth (data not shown) but infertile. The mutant adult mice were lacking spermatozoa and had reduced size testes (Fig. 2a, b). To validate the testicular defects, we examined the weight gain between the *Uhrf1*^*f/f*^*;Stra8-cre* and control mice at 7, 10, 13, 18, 24, and 36 dpp, respectively. The weight of the *Uhrf1*^*f/f*^*;Stra8-cre* and control groups showed no apparent difference from 7 to 13 dpp. As the growth continued, the mean testicle weight of the *Uhrf1*^*f/f*^*;Stra8-cre* group were significantly lower than that in the control group at each indicated time points (*Uhrf1*^*f/f*^*:* 18.9 ± 0.7 mg (18 dpp), 31.8 ± 1.3 mg (24 dpp), 54.0 ± 4.5 mg (36 dpp); *Uhrf1*^*f/f*^*;Stra8-cre*: 16.4 ± 0.9 mg (18 dpp), 15.1 ± 1.0 mg (24 dpp), 16.3 ± 1.2 mg (36 dpp); *n* = 3; Fig. 2c). These results suggested that loss of UHRF1 led to a failure of testicle development.

The development of the *Uhrf1* knockout mouse germ cell was apparently normal shortly after entering into the meiosis. An ordered arrangement of preleptotene, leptotene, zygotene, and early pachytene stage spermatocytes was observed both in the *Uhrf1*^*f/f*^*;Stra8-cre* and *Uhrf1*^*f/f*^ groups at 7 and 12 dpp (data not shown). Figure 2d showed the middle/late pachytene spermatocytes with the characteristic of thick fibers separated within the nucleus and obvious sex bodies (arrowed in red) in the control sections. However, the spermatocytes were still in the early pachytene-like stage with the characteristic of heavily stained thick fibers but absent sex bodies in the mutant mouse testis sections at 18 dpp (arrowed in black). At 24 and 36 dpp, we detected the haploid spermatids (arrowed in green) and spermatozoa (arrowed in blue) in the control testes. However, the most advanced stage of spermatocyte was still at the early pachytene-like stage (arrowed in pink) in the *Uhrf1*^*f/f*^*;Stra8-cre* mouse sections without spermatids or spermatozoa. These histological defects implied that the impairment of spermatocyte development caused by the deletion of UHRF1 occurred in the process of meiosis I. In adult, we detected that the *Uhrf1* conditional knockout testes were impaired even severely with fewer spermatogonium or early stage spermatocytes and were also devoid of spermatids (Fig. 2e). We assumed this might probably be due to the first expression of *Stra8-cre* in differentiating spermatogonia and the age effect of spermatogonia.

### Loss of UHRF1 resulted in a disturbed meiotic prophase progression, impaired SC assembly, and affected homology synapsis

**Fig. 3:**
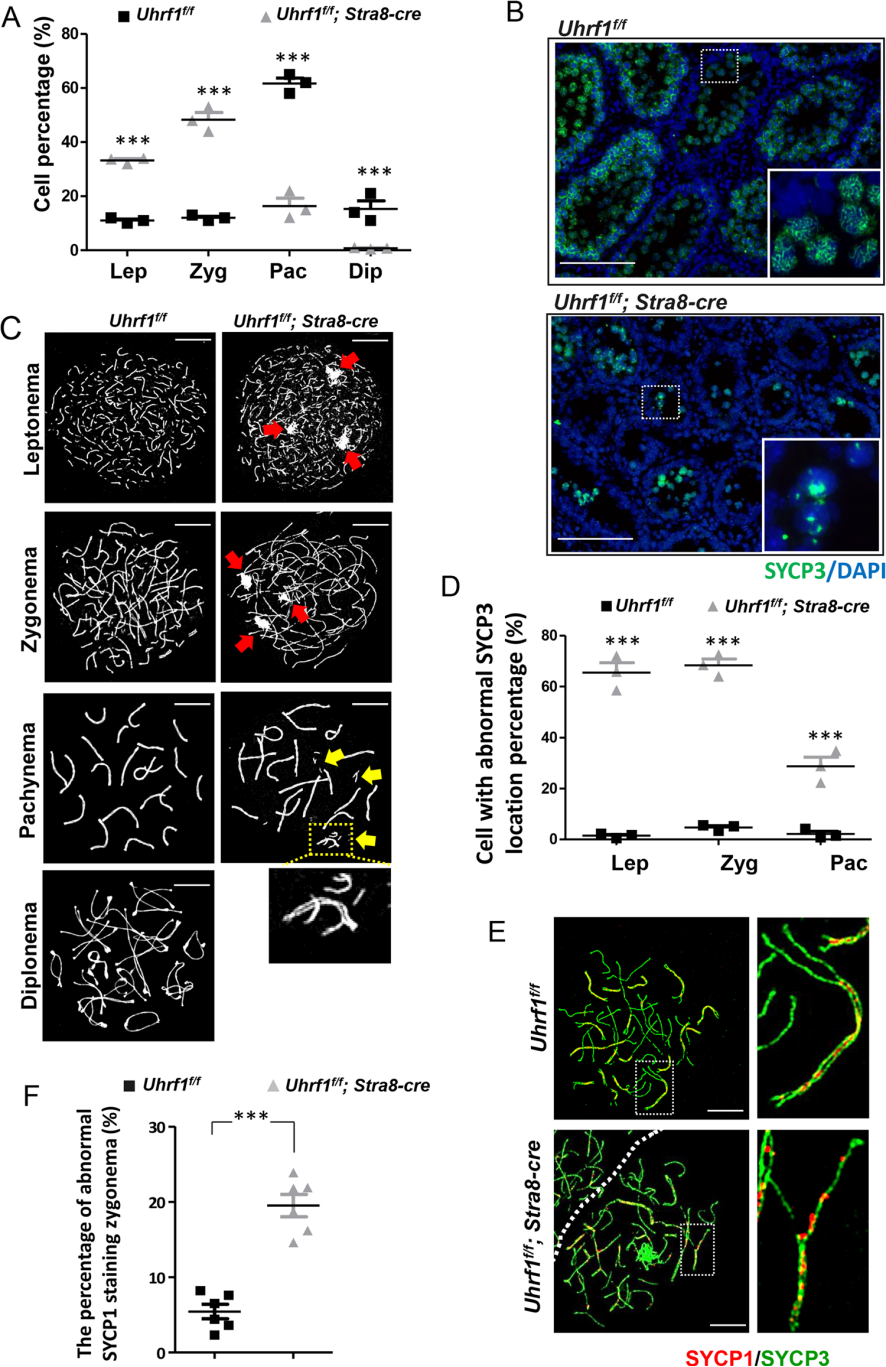
UHRF1 deletion disrupted the meiotic progression and synaptonemal complex assembly. **a** Relative amounts of four spermatocyte populations (leptotene stage, zygotene stage, pachytene stage, and diplotene stage) during the prophase I in testes based on analyzing >600 spermatocytes in each stage. **b**, **c** The immunostaining of SYCP3 in the testicular sections (**b**) and surface-spread chromatin preparations of *Uhrf1* deletion and control mice (**c**); **d** the percentage of spermatocytes with abnormal SYCP3 location. **e** Double immunofluorescence of testicular spread preparations of the adult mice, SYCP3 (green) and SYCP1 (red). **f** The percentage of spermatocytes with abnormal SYCP1 location. Lep leptotene, Zyg zygotene, Pac pachytene, Dip diplotene. Data are presented as mean ± SEM of three mice. ***p ≤ 0.001. Scale bar, 25 μm in **b**, 5 μm in **c**, **e**.

For further analysis, we examined spermatocyte spreads from 8-week-old mice. The ratios of the leptotene and zygotene stage cells of the *Uhrf1*^*f/f*^*;Stra8-cre* mice were increased significantly compared with the control group (~33.3% vs ~11.0%, ~45.6% vs ~12.0%), while the ratios of pachytene and diplotene stage cells were decreased in the *Uhrf1*^*f/f*^*;Stra8-cre* mice (~16.4% vs ~61.7%, ~2.2% vs ~15.3%) (Fig. 3a). This data suggested that UHRF1 deficiency interfered severely with the progression of meiotic prophase.

The SC is formed by two lateral elements (LEs), between which the transverse filaments (SYCP1) spanned. The thread-like SYCP3, a major composition of LE, was observed in the control spermatocytes, whereas a large number of spot-like distribution pattern of SYCP3 was found in the mutant germ cells (Fig. 3b). The leptonema and zygonema without UHRF1 were preferentially associated with defected SYCP3 stretches (Fig. 3c, d). These aberrant localizations of SYCP3 (arrowed in red) indicated the impaired assembly of LEs. In the pachytene-like stage, we found the failure of homologous synapsis in some chromosomes (arrowed in yellow and enlargement) in the mutant spermatocytes. Together with the dramatically reduced pachynema number, these above observations suggested that UHRF1 deficiency led to the impaired assembly of axial/lateral elements (AE/LEs), which resulted in the failure of SC formation. The abnormal distribution of SYCP1 verified such SC assembly defect (Fig. 3e, f).

### UHRF1 was essential for the meiotic recombination and pachytene development

**Fig. 4:**
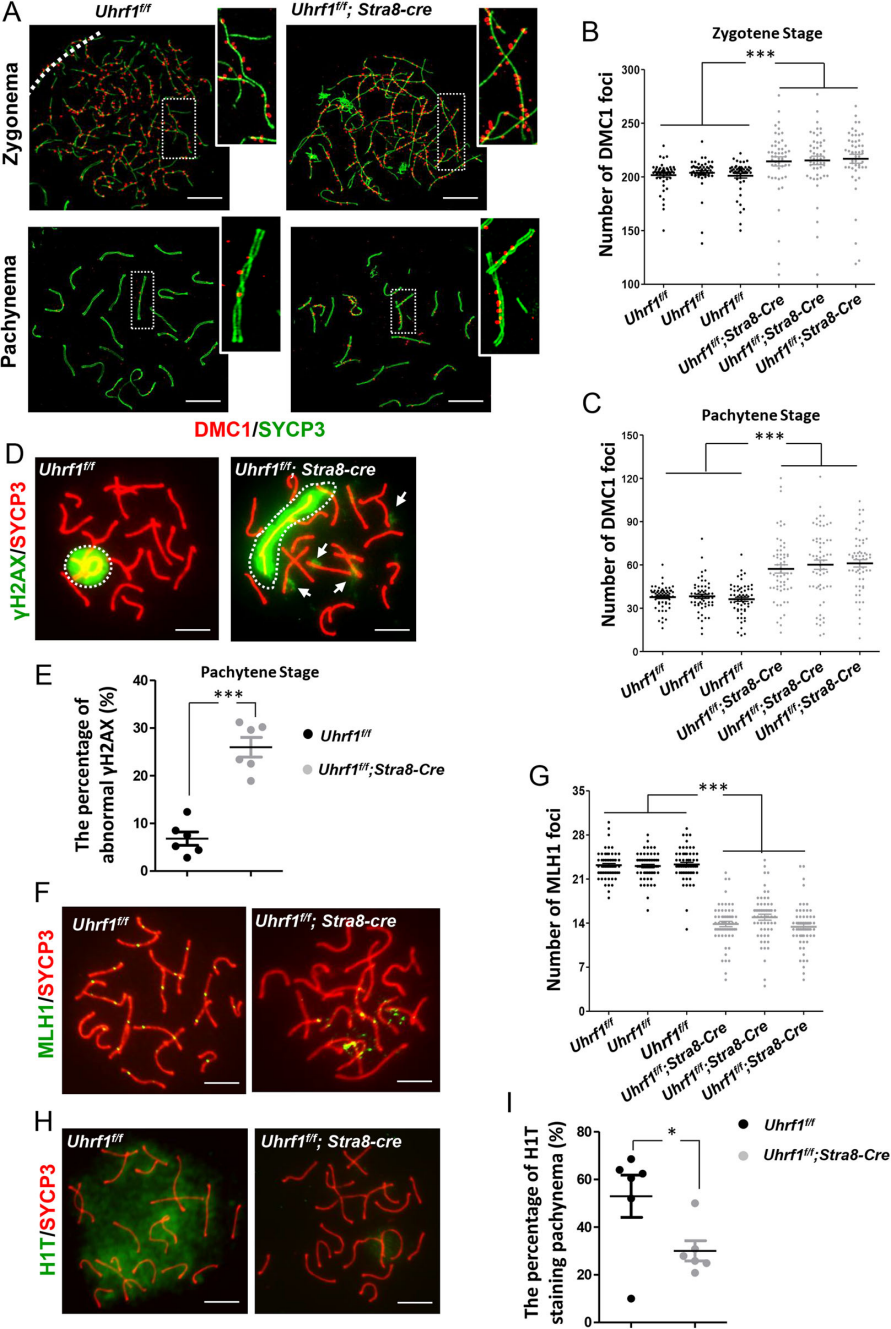
UHRF1 deficiency resulted in impaired meiotic recombination and defective pachynema. **a** Double immunofluorescence of SYCP3 (green) and DMC1 (red) in testicular spread preparations. **b**, **c** The number of DMC1 foci in zygotene stage (**b**) and pachytene stage (**c**). **d** Immunostaining for SYCP3 (red) and γH2AX (green). **e** The percentage of abnormal γH2AX foci in the pachytene stage. **f** Immunostaining for SYCP3 (red) and MLH1 (green). **g** The number of MLH1 foci in pachynema. **h** Immunostaining for SYCP3 (red) and H1t (green). **i** The percentage of spermatocytes with H1T staining. ***p ≤ 0.001; *p ≤ 0.05. Scale bar, 5 μm in **a**, **d**, **f**, **h**.

Meiotic recombination is tightly coupled with synapsis^44^, so we determined whether the deficiency of UHRF1 impaired meiotic recombination. Following the DNA double-strand break (DSB) formation, the DNA ends are engaged in a process of maturation. The DMC1 complex is recruited to the 3′ single-strand DNA tails to promote homology search and strand exchange in the zygotene stage. After the finish of DSB repair, most of the DMC1 are released from the resolved DSBs in pachynema. Recombination shuffles parental genomes through genetic exchanges leading to crossovers (COs) or non-crossovers (NCOs)^45^. The NCOs have only a limited and local effect on genetic diversity, while COs exert critical roles in ensuring an accurate segregation of homologs and generating genetic diversity^44^. The presence of MLH1 sites on paired chromosomes is the final marker for sites that will result in COs^46^. Here the DMC1 foci were increased both in the zygotene and pachytene stages. The excessive DMC1 foci suggested that loss of UHRF1 led to an increased number of DSB sites in the *Uhrf1*^*f/f*^*;Stra8-cre* zygonema. And the retention of DMC1 foci implying the unresolved DSBs suggested that loss of UHRF1 led to DSB repair deficiency (Fig. 4a–c). This deficient DSB repair was further confirmed by the γH2AX defect (a histone variant H2AX with phosphor-Ser139, restricted to the DSB foci; Fig. 4d, e). Only the sex body (white dashed regions, left) was stained with γH2AX antibody in the control pachynema, indicating that most DSBs were repaired in the autosomes. However, the UHRF1-deficient pachynema were with impaired sex body (white dashed regions, right). γH2AX foci remaining on the autosomes (arrowed in white) indicated the presence of un-repaired DSBs. A reduced number of MLH1 foci was also detected in the mutant pachynema (Fig. 4f, g) showing that loss of UHRF1 also resulted in impaired CO. The failure of H1T staining, a known mid/late pachytene marker^47^, further demonstrated the defective pachynema in the mutant mice (Fig. 4h, i).

Collectively, deletion of UHRF1 caused a skewed recombination profile with the characteristic of excessive and retained early recombination foci but depleted the late foci and a poor incorporation of histone H1T into meiotic chromatin.

### Knockout of UHRF1 altered the gene expression and led to global hypomethylation

**Fig. 5:**
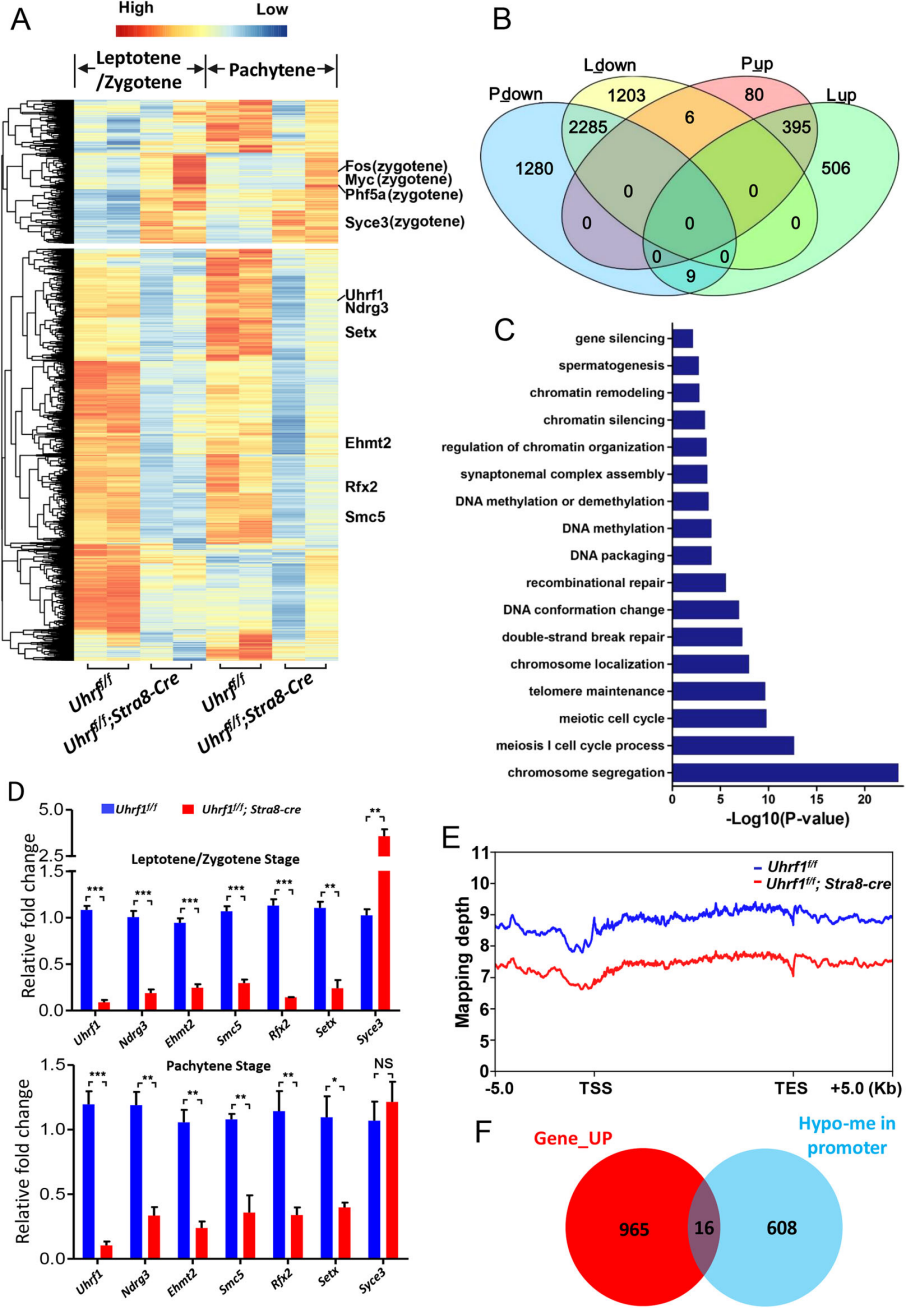
The gene expression and global methylation after UHRF1 deletion. **a** RNA-seq for mouse spermatocytes (leptotene/zygotene and pachytene stages). Clustered heat map of log_2_-transformed RPKMs showing the differentially expressed genes after UHRF1 deletion. Indicated genes are marked in right. **b** Identified differentially expressed genes were highly consistent between leptotene/zygotene and pachytene stages. (P pachytene stage genes, L leptotene/zygotene stage genes). **c** Gene ontology analysis of DEGs using the database of ClusterProfiler V3.4.4. **d** RT-PCR assay showing the relative change of some representative meiotic genes. **e** DNAme densities across the gene bodies of all reference genes. **f** Venn diagram depicting a set of 16 genes that were induced in transcripts and hypomethylated in promoter and 5’ UTR regions (unique in control) from the leptotene to pachytene stage in the UHRF1-deficient spermatocytes. For the upregulated transcripts, the cut-off was *p* value < 0.001 with fold change >2.

To identify DEGs in the UHRF1-deficient spermatocytes that impair the progression of meiotic prophase, we performed RNA-seq and qPCR to isolate spermatocytes from *Uhrf1*^*f/f*^*;Stra8-cre* and *Uhrf1*^*f/f*^ mouse (leptotene/zygotene and pachytene stages). A total number of 4768 genes were downregulated and 981 genes were upregulated with *p* value < 0.001, |log_2_fold change| >1 in either of these two stages (Fig. 5a, b and Table S2). Gene Ontology (Cluster Profiler V3.4.4) highlighted the that DEGs were involved in the regulation of the meiosis process (Fig. 5c). Many meiosis-associated genes were altered. The downregulation of SC assembly gene *Ehmt2*^48^, recombination-associated gene *Setx*^49^ and *Ndrg3*^50^, chromosome segregation gene *Smc5*^51,52^, and *H1t* transcriptional regulator *Rfx2*^53^ provided the molecular mechanisms by which how deficiency of UHRF1 generated the defects in SC assembly, recombination, and H1t incorporation (Fig. 5d). Interestingly, we detected an increase of *Syce3*^54,55^, which was specifically located in the central element in the leptotene/zygotene stage. The increase of *Syce3* as well as the inter-sister chromosome distribution of SYCP1 (Fig. 3e) further confirmed the SC assembly defect.

Considering that UHRF1 is critical to DNA methylation (DNAme), we examined the genomic level of DNAme. The methylated DNA immunoprecipitation-sequencing (MeDIP-seq) showed an average loss of DNAme approximately by 15.83% in genome in the *Uhrf1*^*f/f*^*;Stra8-cre* spermatocytes (Fig. 5e). A validation was performed by the bisulfite assay to some retrotransposable elements, which were reported to be hyper-methylated^56,57^ (Supplementary Fig. S1). The methylation rates of IAPEz, L1Md_T, and RTRL8-int were 88.19%, 88.39%, and 88.07% in the *Uhrf1*^*f/f*^ meiotic prophase cells, while the rates were decreased to 25.69%, 61.16%, and 40.34% in the *Uhrf1*^*f/f*^*;Stra8-cre* cells, respectively. Given that the 5mC enrichment in the promoter regions is negatively correlated to the transcriptional level^58^, we compared the results from MeDIP-seq and RNA-seq (upregulated genes). Figure 5f showed that the DNA hypo-5mC in the promoters had a limited effect on the transcriptional level. Only 16 (~1.63%) genes, most of which were without clear function in meiosis, were found to have hypomethylation levels in their promoter regions in the *Uhrf1*^*f/f*^*;Stra8-cre* spermatocytes. The little effect on gene transcription of hypomethylation in the *Uhrf1* knockout mice implied an unexplained mechanism of UHRF1 on the regulation of meiotic gene expression.

### Blocking of UHRF1 exhibited hyper-5hmC in the TSS region of downregulated genes

**Fig. 6:**
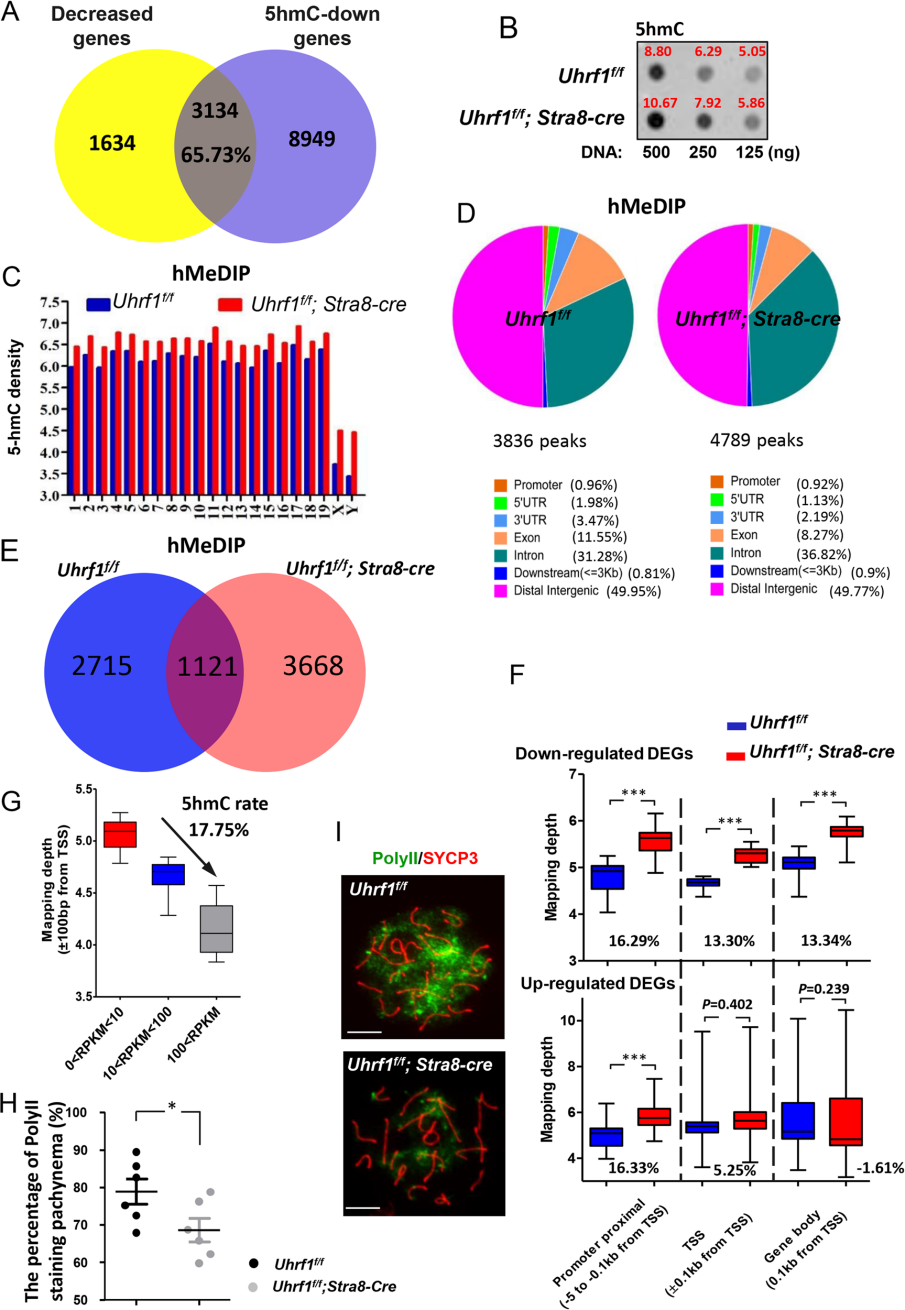
Hyper-5hmC resulted from UHRF1 deletion. **a** Venn diagram depicting reduced transcripts associated with 5hmC downregulation. **b** 5hmC level of meiosis prophase I spermatocytes (16 dpp). **c** 5hmC densities of all chromosomes. **d** The distribution of 5hmC density on the genome of spermatocytes. **e** Venn diagram depicting 5hmC peaks in *Uhrf1*^*f/f*^*;Stra8-cre* and *Uhrf1*^*f/f*^ spermatocytes. **f** 5hmC densities was shown in the proximal promoter, TSS, and gene body regions of the DEGs. **g** 5hmC densities in TSSs of the total refgenes with different RPKMs. **h** The percentage of RNA polymerase II staining in pachynema. **i** Double immunofluorescence of testicular spread preparations, SYCP3 (red) and RNA polymerase II (green). Scale bar, 5 μm in **i**.

During the meiotic prophase I, the global DNAme maintains constantly, whereas the 5hmC reduces dramatically^22,59^. There were 12,083 genes reported with 5hmC downregulated from preleptonema to pachynema^22^. As the distribution of 5hmC is highly associated with gene expression, we compared our RNA-seq data (significantly downregulated genes) with this reported set of 12,083 genes and found >65.73% of the repressed genes in the UHRF1-deficient spermatocytes were overlapped (Fig. 6a). We assumed that the deficiency of UHRF1 led to an altered 5hmC. To verify this assumption, we checked the global 5hmC in the *Uhrf1*^*f/f*^*;Stra8-cre* and *Uhrf1*^*f/f*^ mouse meiotic prophase cells. Dot blot assay revealed that the deficiency of UHRF1 resulted in an upregulation of global DNA 5hmC (Fig. 6b). For further analysis, the hMeDIP-seq was carried out. hMeDIP-seq showed UHRF1 deficiency led to a dysregulated DNA hyper-5hmC in all chromosomes (Fig. 6c). 4789 hMeDIP peaks were detected in the mutant spermatocytes, whereas the number in the control was 3836. Both of the hMeDIP peaks were diversified in distribution at promoter, 5′ or 3′ UTR, and exon and intron or intragenic regions (Fig. 6d). However, it would be noteworthy that there were 3668 peaks unique in the mutant cells, suggesting the 5hmC profile was greatly changed without UHRF1 (Fig. 6e).

The 5hmC distribution in the promoter region and gene body contributes to gene repression and activation, respectively^60,61^. We mapped the hMeDIP peaks of DEGs, finding the average level of 5hmC raised by 16.29%, 13.30%, and 13.34% significantly in the regions of proximal promoter (−5 to −0.1 kb from TSS), TSS (±0.1 kb from TSS), and gene body (0.1 kb from TSS), respectively, in the downregulated gene set. In the upregulated gene set, the average level of 5hmC rate raised by 16.33% significantly in the region of proximal promoter. However, the changes of average level of 5hmC rate in the regions of TSS and gene body was not statistically significant (*p* = 0.402 and *p* = 0.239, respectively, Student’s *t* test; Fig. 6f). A previous report suggested that the inhibition of transcriptional activity is primarily due to the presence of 5hmC in the promoter and that 5hmC in the gene body has a minimal effect on transcription^61^. Here our result showing the elevated 5hmC level in TSS was highly associated with downregulated DEGs implied the primary repressing role of 5hmC in TSS in gene transcription. However, the upregulated genes seemed to be resistant to this repressive effect of UHRF1 to 5hmC around TSS, because the upregulated genes failed to raise much 5hmC rate significantly. UHRF1 deletion might increase their expression by other mechanisms, considering it a multiple functional domain-containing protein. Furthermore, we found that the refgenes’ peaks of 5hmC in TSS region were negatively associated with RPKMs. The 5hmC in TSS region was decreased by 17.75% from the low RPKM gene set (0 < RPKM < 10) to high RPKM gene set (RPKM > 100) (Fig. 6g). To validate, we examined the 5hmC level in TSS region of some decreased meiotic genes such as *Dazap1*^62^, *Ehmt2*^48^, *Rif1*^63^, and *Rad23*^64^ using the hMeDIP-qPCR^65^ and EpiMark 5-hmC and 5-mC Analysis Kit (NEB, E3317S), which was applied to determine the 5hmC rate by us and others previously^66,67^. The consensus results further confirmed that the deficiency of UHRF1 exhibited hyper-5hmC in the TSS region in the downregulated genes (Supplementary Fig. S2).

5hmC at TSS inhibits the binding of RNA-Pol2 to DNA^60,61,68^. Here a diminished RNA-Pol2 in the UHRF1-deleted spermatocytes probably provided a piece of evidence that the hyper-5hmC in the repressed gene TSS region could possibly contribute to the affected RNA-Pol2 loading to decrease gene transcription. The average percentage of RNA-Pol 2 staining was 78.9% in the control spermatocytes, while the ratio was decreased significantly to 68.6% in the UHRF1-deficient spermatocytes (Fig. 6h). In some *Uhrf1* knockout spermatocytes, a severe loss of RNA-Pol2 signal was found (Fig. 6i). The different degrees of the impairment of RNA-Pol2 staining implied that there might be other potential mechanisms that UHRF1 regulated RNA-Pol2 binding besides the 5hmC-TSS-dependent way.

### The levels of 5hmC and TET1 were upregulated after hypo-methylation in the UHRF1-deleted spermatocyte

**Fig. 7:**
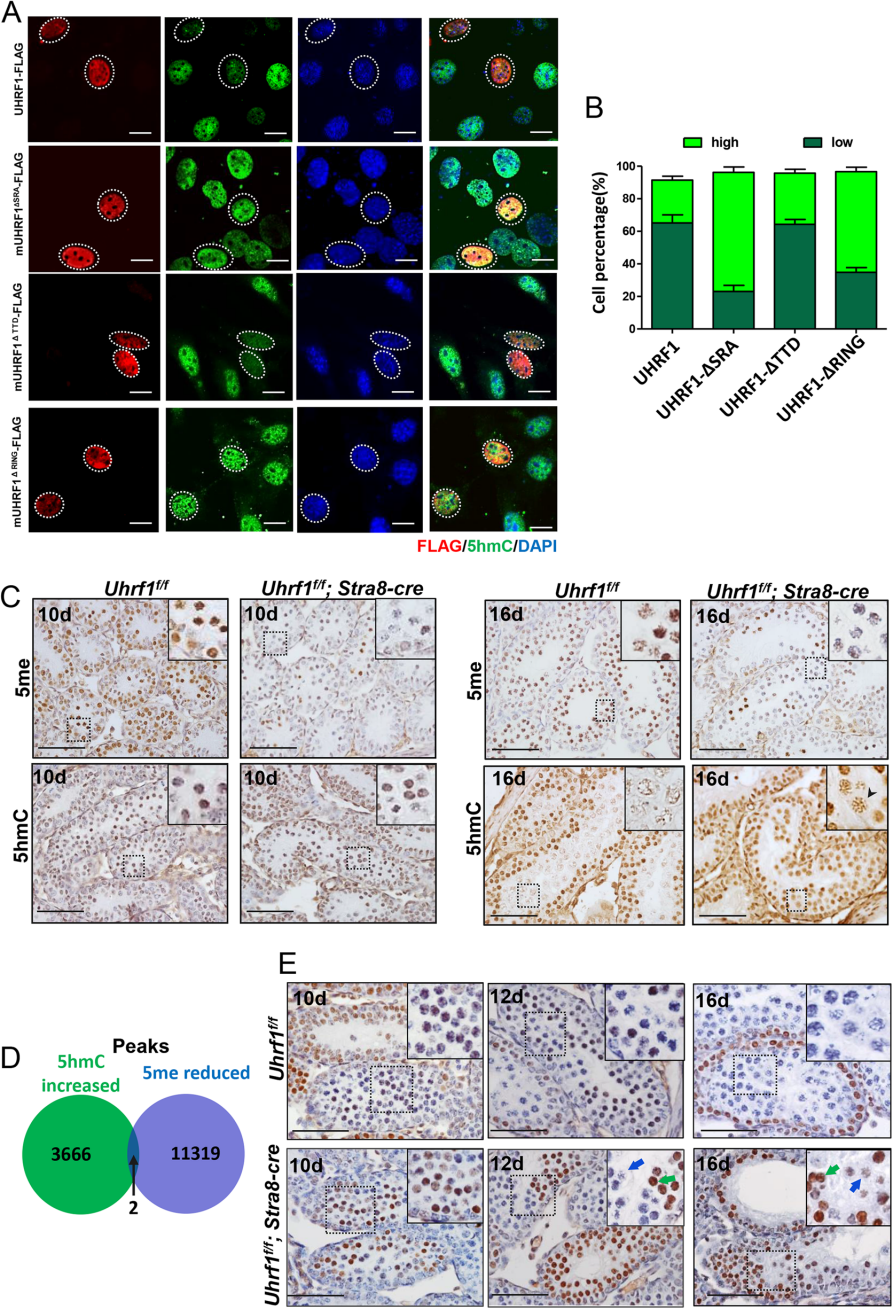
The levels of 5hmC and TET1 were upregulated after hypo-DNAme. **a** Overexpression of *UHRF1*, *mUHRF1-ΔSRA-FLAG*, *mUHRF1-ΔTTD-FLAG*, and *mUHRF1-ΔRING-FLAG* plasmids and the level of 5hmC in GC1 cells. **b** Percentage of the cells with strong or weak intensity of green fluorescence. **c** Immunohistochemistry assay of DNAme and 5hmC in the mouse spermatocytes. **d** Venn diagram showing the overlap between hyper-5hmC and hypo-DNAme peaks. The significance was evaluated by Fisher’s exact test. **e** Immunohistochemistry assay of TET1 in the mouse testis. Scale bar, 10 μm in **a**, 25 μm in **c**, **e**.

Considering the presence of hyper-5hmC caused by the deletion of UHRF1, we assumed UHRF1 repressed 5hmC in spermatocytes. UHRF1 harbors multiple domains^69^. For example, the Tandem Tudor domain (TTD) allows UHRF1 to bind to di-/tri-methylated H3K9, the Set and RING Associated (SRA) domain facilitates in maintaining DNA methylation and histone modifications by recruiting DNMT1 and HDAC1, and Really Interesting New Gene (RING) domain confers intrinsic E3 ligase activity toward histones and non-histone proteins. Thus we next determined which domain was critical in this repression role. In vitro assay showed that a weak level of 5hmC signal was observed in approximately 65.1% and 64.27% GC-1 cells transiently transfected with full *UHRF1* (pcDNA3.1-*UHRF1-Flag*) and mutant *UHRF1* with *TTD* domain deleted (pcDNA3.1-_*m*_*UHRF1-ΔTTD-Flag*) plasmids, respectively. However, when the cells overexpressed the mutant *UHRF1* with *SRA* domain deleted (pcDNA3.1-_*m*_*UHRF1-ΔSRA-Flag*) and mutant *UHRF1* with *RING* domain deleted (pcDNA3.1-_*m*_*UHRF1-ΔRING-Flag*) plasmids, 73.1% and 61.83% GC-1 cells were with strong 5hmC signals, respectively (Fig. 7a, b). These results indicated that SRA and RING domains were indispensable for UHRF1-repressed global DNA 5’-hydoxymethylation.

As we observed that DNA 5’-hydoxymethylation was increased in the context of global hypo-DNAme in vivo, we next analyzed the relationship between the hypo-5me and hyper-5hmC caused by UHRF1 inactivation. In 10 dpp sections, the downregulation of 5mC, but not upregulation of 5hmC, was observed in the mutant spermatocytes. However, both of the two events were clearly observed in the 16 dpp sections (Fig. 7c). The dot blot assay also confirmed that the upregulation of 5hmC was apparent in the pachytene but not in leptotene stage (Supplementary Fig. S3). In addition, we compared the 3668 upregulated hMeDIP peaks (unique in the mutant spermatocytes) with 11,321 downregulated MeDIP peaks (unique in the control spermatocytes), finding that the overlapping rate was insignificant (Fisher’s exact test: *p* value = 1) (Fig. 7d). Together, our results revealed a spatiotemporal discrepancy of the reduced 5mC and raised 5hmC in the spermatocytes with depleted UHRF1. Raised 5hmC peaks were converted from DNAme sites but not from the hypo-DNAme sites in the mutant spermatocytes.

TET1–3 are physiologically downregulated in male meiotic prophase^22^. However, we found that TET1, but not TET2 or TET3, was increased gradually in the mutant testis, whereas it was constantly weak from 10 dpp to 16 dpp in the control spermatocytes (Fig. 7e and Supplementary Fig. S4). Note that, in 12 and 16 dpp, the meiotic cells with high TET1 level displayed a characteristic of early meiotic cells (leptotene-like, arrowed in green), whereas the cells with low TET1 level displayed a characteristic of later meiotic cells (zygotene or early pachytene-like, arrowed in blue). The upregulation of TET1 provided a rational explanation for this presence of hyper-5hmC in the spermatocytes without UHRF1.

## Discussion

Collectively, we reported that UHRF1 regulated hydroxymethylation in male meiosis. Loss of UHRF1 caused global upregulation of 5hmC. Hyper-5hmC in the TSS region was highly associated with gene repressing in the prophase of meiosis I. In this present study, the repression role of UHRF1 to 5hmC might partially be the mechanism of physiological downregulation of 5hmC in male meiotic prophase I.

In our model, an attenuated DNA methylation was detected. We assumed that this is because of the DNA methylation maintenance role of UHRF1and *Stra8* expression prior to meiotic prophase. A primary role of DNA methylation is to safeguard the genome via transcriptional silencing of transposable elements during meiosis^6,70,71^. The redundant DMC1 foci and abnormal distribution of γH2AX in the *Uhrf1* knockout spermatocytes strongly implied an impaired genome. A strong activation of RNA retrotransposable elements (for example, SINEs, LINEs, and LTRs) was detected (Supplementary Fig. S5) here. However, we found downregulation of DNA methylation here had little effect on gene transcription increase. A similar conclusion is also reported elsewhere^34,72^. So we think the mechanism of UHRF1 regulating gene transcription in meiosis is different from that in spermatogonium differentiation.

A distinct characteristic of meiosis is a highly protracted cell cycle due to long meiotic S phase and prophase I^73–75^. During the meiotic S phase and prophase I, a series of profound epigenetic changes occur. The genome-wide 5mC marks are produced prior to meiotic prophase (S phase). Once established, the DNA methylation keeps at relatively high and constant levels in the subsequent stages of meiotic prophase in male germ cells^59,72^. 5hmC, however, decreases dramatically from preleptotene to pachytene stage. As the influence of 5hmC distribution on gene expression, it might be possible that such highly ordered 5hmC dynamic change is required for the spatiotemporal expression of genes in meiosis. By the conditional *Uhrf1* knockout mouse model whose spermatocyte DNA 5hmC level was upregulated, we found that elevated 5hmC in TSS region might probably contribute to the prevention of RNA-Pol2 binding to downregulate gene expression. However, it should be pointed out that the mechanism of UHRF1 regulating gene expression was complicated. For example, although a peak of 5hmC was found upregulated at the TSS region (data not shown), the *Syce3* gene was increased in the leptotene/zygotene stage. We assumed that this increase would be possibly due to the enhanced levels of transcription factors, as we found that *Myc*, *Fos*, and *Phf5a* were enhanced significantly, and such *cis* elements were found in the promoter of *Syce3* gene in silico (Supplementary Fig. S6).

A number of recent studies in PGCs shows that the lack of DNA methylation maintenance is the main cause of global demethylation^76–78^. A recent study in cultured embryonic stem cells reveals that the impairment of DNA methylation maintenance causes a transient upregulation of 5hmC in a TET1/2-dependent way^79^. Our model showing the downregulation of DNAme was followed by a global upregulation of 5hmC in vivo, further implying a synergistic epigenetic regulation of DNA methylation maintenance and hydroxymethylation. Although the specific mechanism of how loss of UHRF1 upregulating 5hmC is still unclear, we show that TET1 but not TET2 or TET3 is increased aberrantly. TET1 controls meiosis while its deficiency results in univalent chromosomes and unresolved DSBs in female^80^. In this study, we showed that an aberrant upregulation of TET1 also affected the meiotic progression.

In summary, by the genetic modified mouse model, we pinpointed the dominant role of UHRF1 regulating male meiosis. This study showing UHRF1-repressed 5hmC highlighted a novel mechanism modifying the epigenetic landscape in spermatogenesis. Although it is unclear currently how UHRF1 targets TET1, we show such repressing role depends on its SRA and RING domains. 5hmC is one of the reported factors that interact with the SRA domain^35^. Therefore, it is worthy to further study whether 5hmC is required for the suppressive effect of UHRF1 on TET1 and how SRA and RING domain work synergistically in male meiosis.

## Supplementary information

Supplemental information

Supplemental figure1

Supplemental figure2

Supplemental figure3

Supplemental figure4

Supplemental figure5

Supplemental figure6

Primer list

DEGs in the leptotene zygotene and pachytene stages
