# Supplementary material for: UHRF1-repressed 5’-hydroxymethylcytosine is essential for the male meiotic prophase I
Source: Cell Death Dis. 2020 Feb 21;11(2):142. doi: 10.1038/s41419-020-2333-3 (PMC7035279; doi:10.1038/s41419-020-2333-3)
Supplement: Supplementary file 1 — Supplemental information [file 41419_2020_2333_MOESM1_ESM.docx]

**Supplementary Information**

**Figure S1.**

**UHRF1 deficiency led to hypo-methylation of IAPEz, L1Md_T and RLTR8-int.**

The DNA methylation status of IAPEz, L1Md_T and RLTR8-int was analyzed by bisulfite sequencing. The percentage of 5mC was calculated and shown.

**Figure S2.**

**Hyper 5’-hydroxymethylcytosine in TSS in the down regulated meiotic genes.**

(A) The fold change of 5hmC level was detected in the -500bp~TSS region of *Dazap1, Ehmt2, Rif1* and *Rad23* by the method of hMeDIP-qPCR. (B) Schematic representation of location of the CCGG loci in the -500bp~TSS region of *Dazap1, Ehmt2, Rif1* and *Rad23*. (C) The 5hmC rates of *Dazap1, Ehmt2, Rif1* and *Rad23* were measured by EpiMark 5-hmC and 5-mC Analysis Kit between the *Uhrf1^f/f^;Stra8-cre* and *Uhrf1^f/f^* spermatocyte DNA.

**Figure S3.**

**5hmC levels of different stages of meiosis prophase I spermatocytes**

Dot blot assay showing the 5hmC levels of the isolated leptotene (10dpp) and Pachytene (16dpp) stage spermatocytes.

**Figure S4.**

**TET2 and TET3 in mouse testis**

Immunohistochemistry assay of TET2 and TET3 in the mouse testis. Scale bar, 25 μm.

**Figure S5.**

**UHRF1 deficiency led to activated RNA retrotransposable elements.**

Heat maps showing expression fold changes of different TEs determined by RNA-seq.

**Figure S6.**

**UHRF1 deficiency resulted into the up-regulation of some transcription factors.**

(A) RT-PCR assay showing the relative changes of *Myc*, *Fos* and *Phf5a*. (B) Schematic representation of the locations of the binding sites of the indicated transcription factors of the *Syce3* gene.

**Table S 1: Primer list**

**Table S 2: Differential Expression Genes in the Leptotene/zygotene and Pachytene stages**

DEGs were identified as significantly differential expression in either of Leptotene/zygotene and Pachytene stages.
