# Supplementary figures and images for: UHRF1-repressed 5’-hydroxymethylcytosine is essential for the male meiotic prophase I
Source: Cell Death Dis. 2020 Feb 21;11(2):142. doi: 10.1038/s41419-020-2333-3 (PMC7035279; doi:10.1038/s41419-020-2333-3)

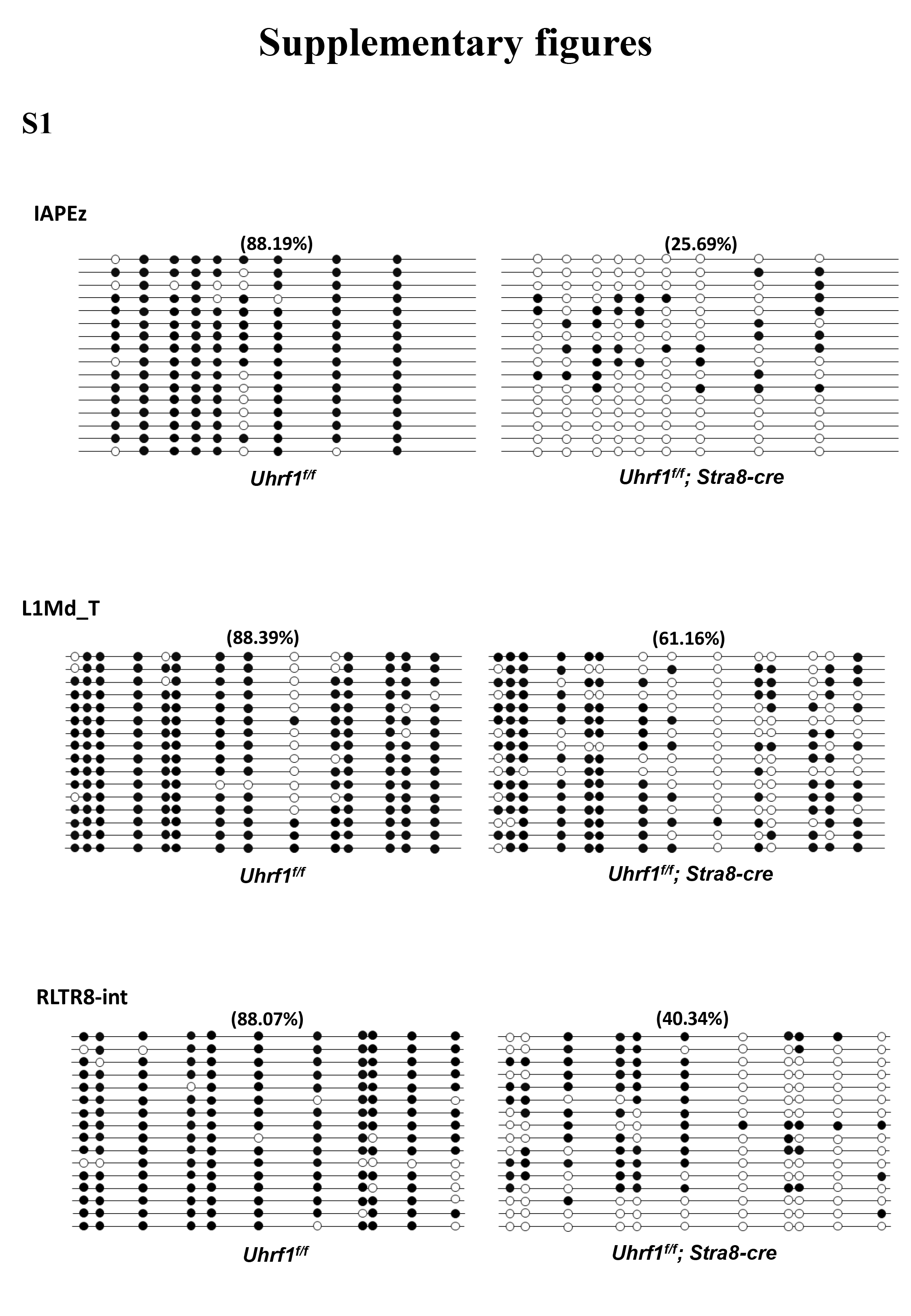

Supplement: Supplementary file 2 — Supplemental figure1 [file 41419_2020_2333_MOESM2_ESM.tif]

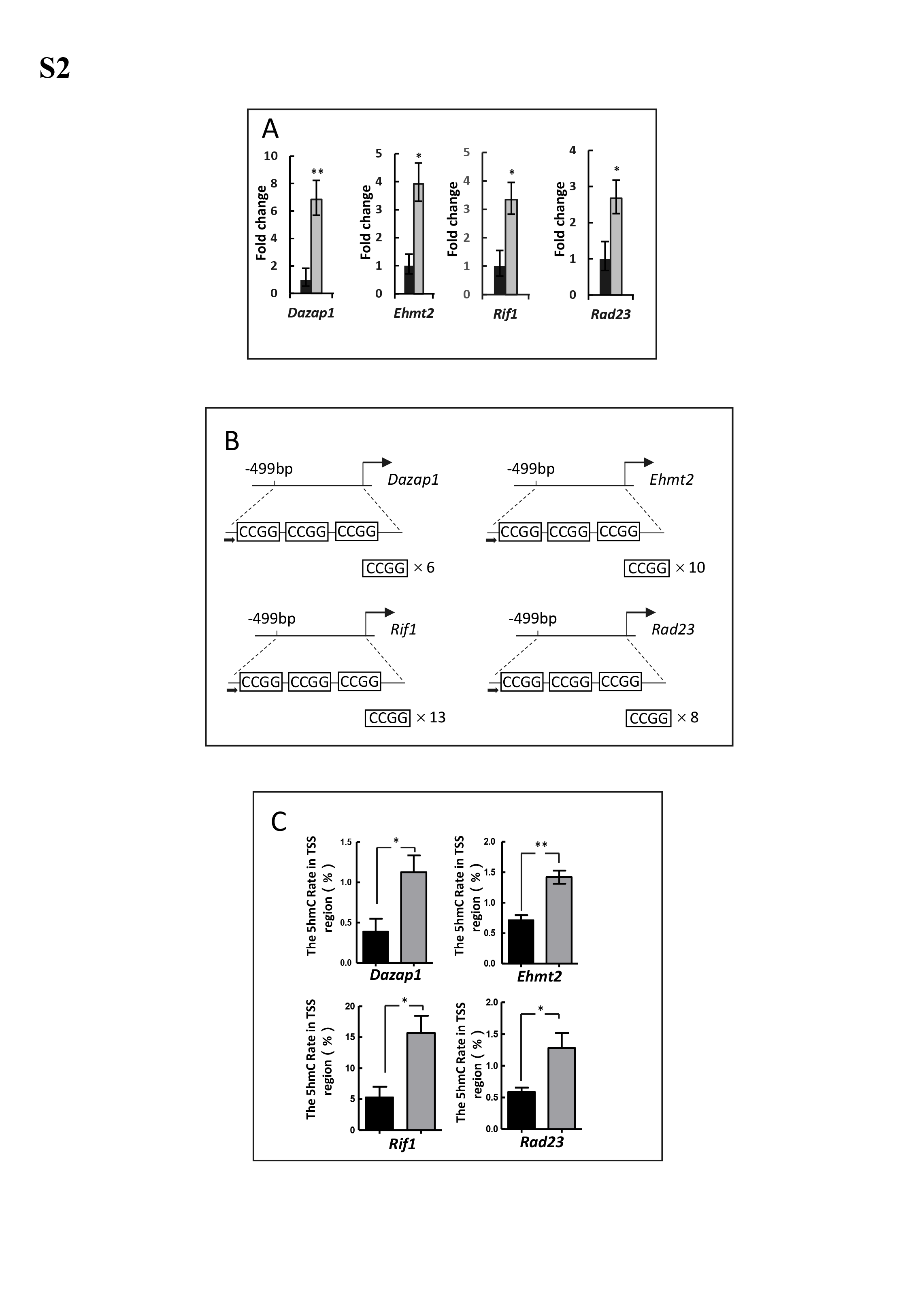

Supplement: Supplementary file 3 — Supplemental figure2 [file 41419_2020_2333_MOESM3_ESM.tif]

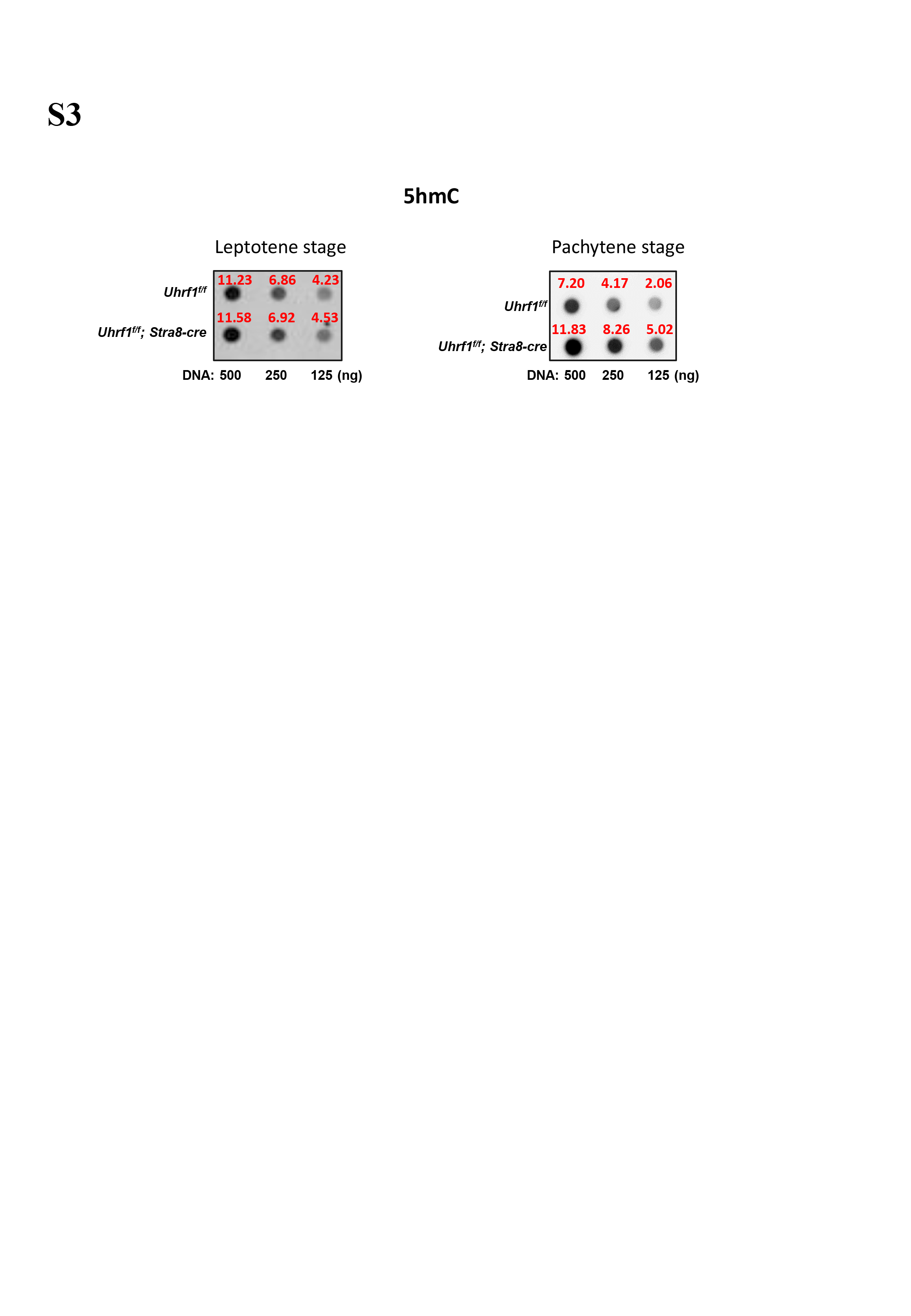

Supplement: Supplementary file 4 — Supplemental figure3 [file 41419_2020_2333_MOESM4_ESM.tif]

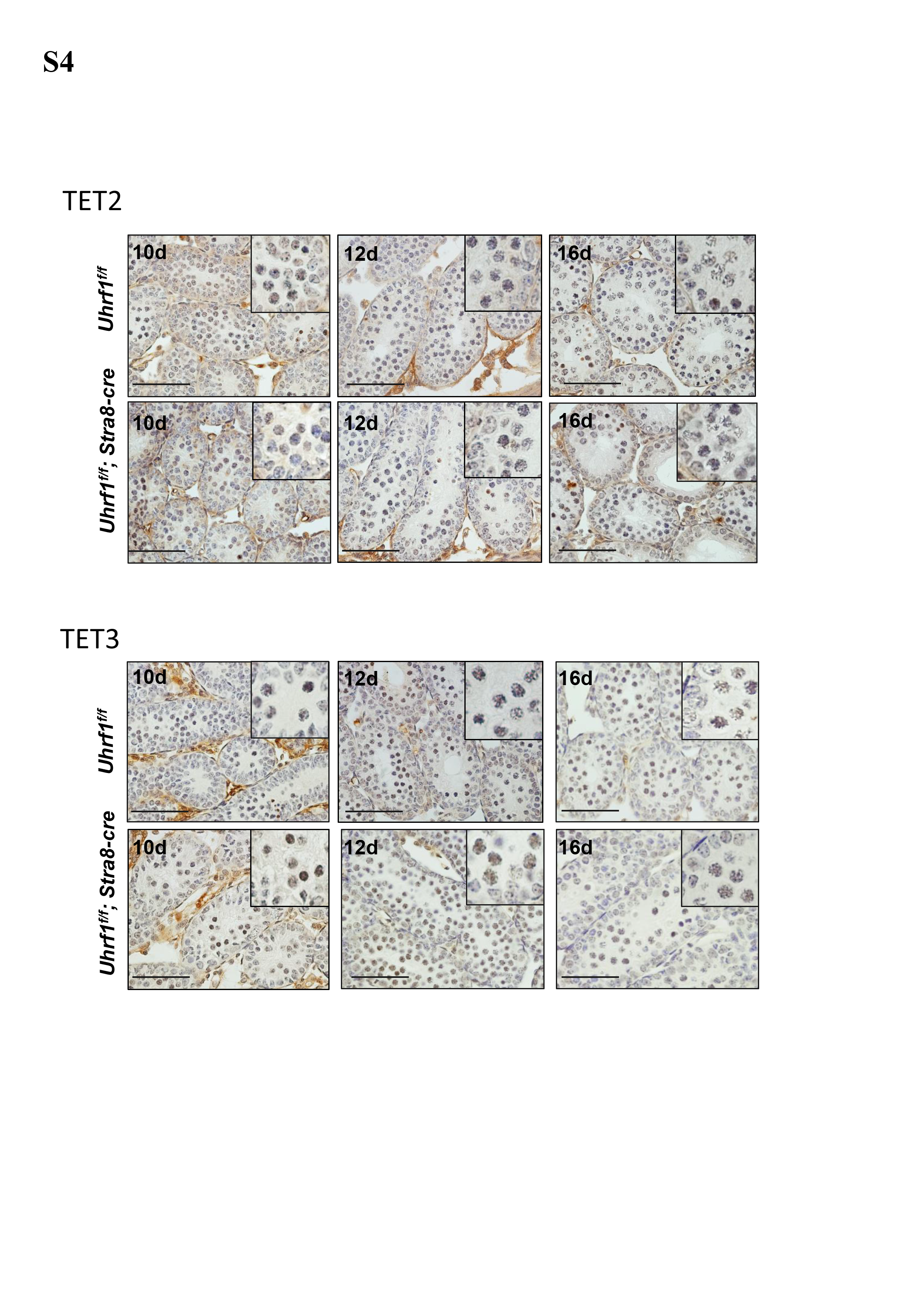

Supplement: Supplementary file 5 — Supplemental figure4 [file 41419_2020_2333_MOESM5_ESM.tif]

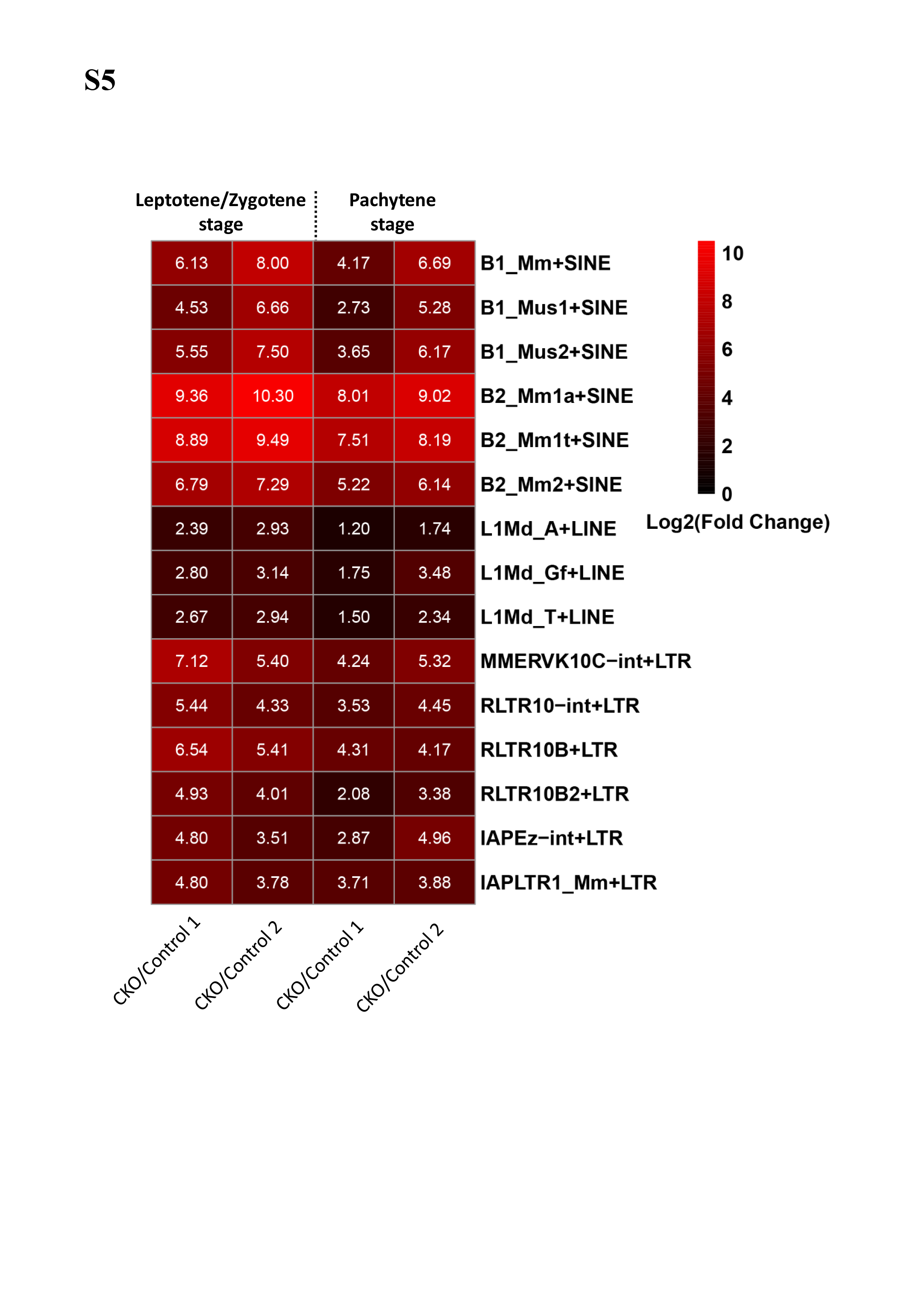

Supplement: Supplementary file 6 — Supplemental figure5 [file 41419_2020_2333_MOESM6_ESM.tif]

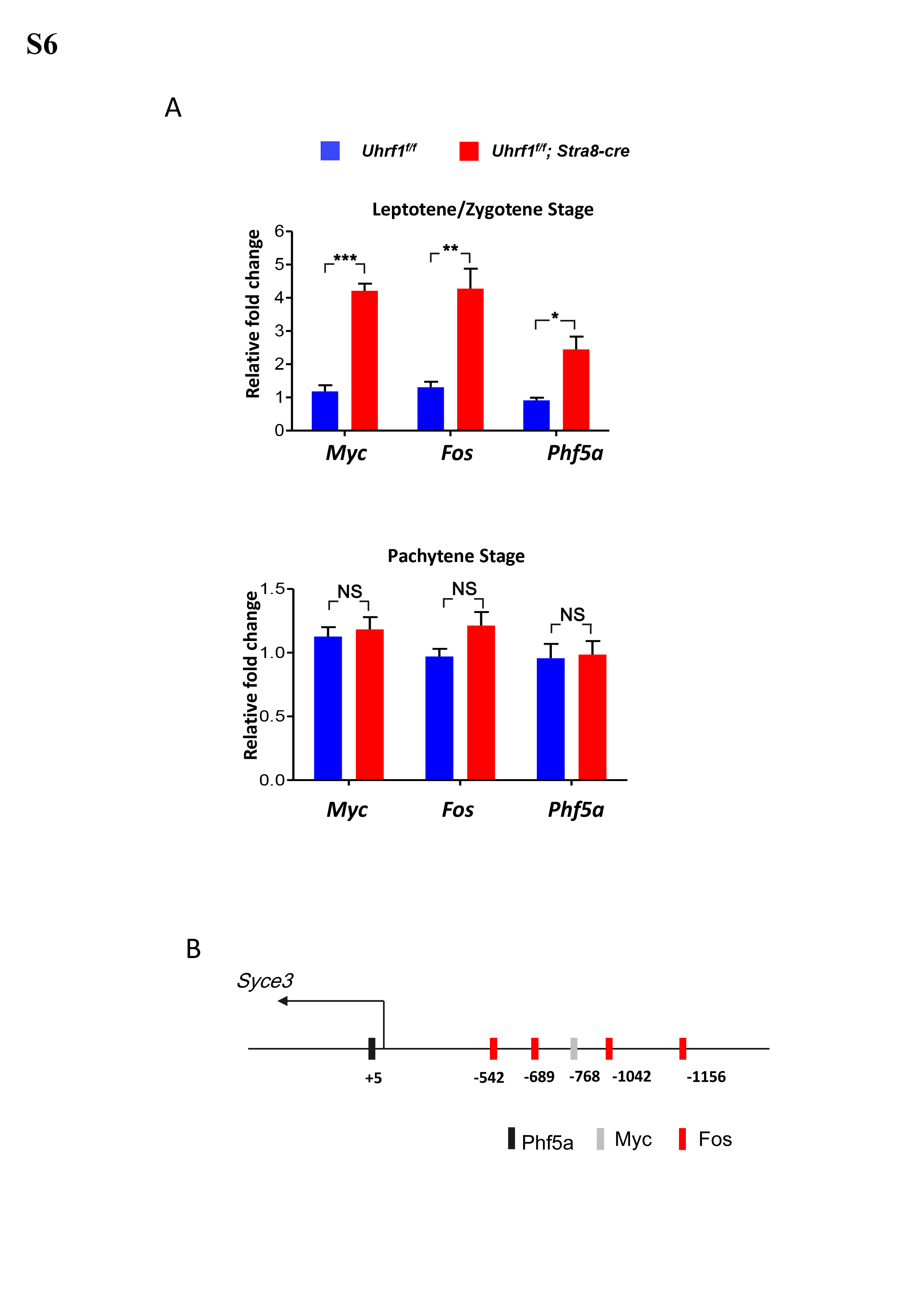

Supplement: Supplementary file 7 — Supplemental figure6 [file 41419_2020_2333_MOESM7_ESM.tif]
