# Supplementary material for: UHRF1-repressed 5’-hydroxymethylcytosine is essential for the male meiotic prophase I
Source: Cell Death Dis. 2020 Feb 21;11(2):142. doi: 10.1038/s41419-020-2333-3 (PMC7035279; doi:10.1038/s41419-020-2333-3)
Supplement: Supplementary file 8 — Primer list [file 41419_2020_2333_MOESM8_ESM.pdf]

Table S 1.

Primer List

**Primers for genotyping**		
Uhrf1-flox	ACTCTTGATCTGTGCCCTGC	sense
	ATCCCAGGCCTCCATACACT	anti-sense
Stra8-cre	ACTCCAAGCACTGGGCAGAA	sense
	GCCACCATAGCAGCATCAAA	anti-sense1
	CGTTTACGTCGCCGTCCAG	anti-sense2
**Primers for RT-PCR**		
Uhrf1	CGCTTCAGAGTCCAGGTCAG	sense
	TACGCTTGTTGCCAGAGAGG	anti-sense
Ndrg3	GAGCTGAGCATCCACCTACC	sense
	TTGGTAACGTGGTCCTGTGG	anti-sense
Ehmt2	CCGGTTCCTTGTCTCCCCTC	sense
	AGCTATGAACTCTCTCGGCG	anti-sense
Smc5	CCAGACACAAGTACCCACCAT	sense
	GAGTACTCCTCAACCACCGA	anti-sense
Rxf2	AGCAGCGCAGCATCTTACTT	sense
	CAGAAGCCACTGGAGCGTG	anti-sense
Syce3	CTGTCCTCAAGCAACCTCCG	sense
	GGTTGCCTGCACTGAGATTTT	anti-sense
Stex	TCGATGATGGCCCGATTCTG	sense
	CCACTTCTGCAGGTCCTTTCT	anti-sense
Myc	GCTGTTTGAAGGCTGGATT	sense
	CTGCTGTTGCTGGTGATAGA	anti-sense
Fos	GTTTCAACGCCGACTACGA	sense
	CCTCCTGACACGGTCTTCAC	anti-sense
Phf5a	CTGCACCCTGGTCCGCATAT	sense
	CCACCCACCCATCACCTCTT	anti-sense
**Primers for Bisulfite sequencing**		
IAPEz	AGTTTGTTGATTGGTTTTAGGGTAGT	sense
	TACCTATTATATTTTCCTTTATTCCTCTCT	anti-sense
RLTR6-int	GTTTTGATGTTTGTGTTTTGATGTT	sense
	AACCCCAAATAATCTCTTAACCTTC	anti-sense
L1Md_T	GGTTATTTTTTTGGTGAGAGTA	sense
	CTATACCACAAACCTCTTAAACCTA	anti-sense
**Primers for hMeDIP and EpiMark 5-hmC&5-mC Analysis Kit**		
Ehmt2	GGCACACGCGGCAGCTGCAAAGGCTCAG	sense
	CTTGCGCGGGGGGCCGAGCCGGC	anti-sense
Rif1	ATCTCTAGCCCCGGGCTGGAGGC	sense
	ACCCTGCAGCGAGGCCGGAAGAC	anti-sense
Rad23	CTGCTTGCGAAGTGCGCCTGCGC	sense
	CATGGTGCCTAAGCCGGGGCCTG	anti-sense
Dazap1	CCCACGCAAGACCAAGATTCGCG	sense
	CCGCTCCTCCGGAAGGTCACCG	anti-sense
