# Supplementary material for: UHRF1-repressed 5’-hydroxymethylcytosine is essential for the male meiotic prophase I
Source: Cell Death Dis. 2020 Feb 21;11(2):142. doi: 10.1038/s41419-020-2333-3 (PMC7035279; doi:10.1038/s41419-020-2333-3)
Supplement: Supplementary file 9 — DEGs in the leptotene zygotene and pachytene stages [file 41419_2020_2333_MOESM9_ESM.pdf]

Table S 2: Differential Expression Genes in the Leptotene/zygotene and Pachytene stages

Gene_ID	Symbol	Leptotene/zygotene log2Foldchange	Leptotene/zygotene qvalue	Leptotene/zygotene sig	Pachytene log2Foldchange	Pachytene qvalue	Pachytene sig
ENSMUSG00000096375	Gm7094	-1.31E+00	3.12E-70	down	-8.72E-01	5.50E-39	no
ENSMUSG00000073730	4933415F23Rik	-2.07E+00	1.53E-79	down	-1.70E+00	6.16E-53	down
ENSMUSG00000026063	Pih1d3	-2.29E+00	3.86E-03	no	-2.83E+00	3.34E-09	down
ENSMUSG00000091318	Gm5415	-3.88E+00	1.18E-04	down	-1.42E+00	6.17E-01	no
ENSMUSG00000044744	1700001G17Rik	-5.88E-01	5.26E-01	no	-1.70E+00	5.63E-04	down
ENSMUSG00000091844	Gm8251	-5.97E+00	1.35E-09	down	-4.35E+00	8.20E-02	no
ENSMUSG00000045336	Hsfy2	-3.80E+00	2.14E-99	down	-7.69E-01	2.61E-02	no
ENSMUSG00000036574	1700019O17Rik	-3.94E+00	4.83E-23	down	-2.25E+00	8.95E-03	no
ENSMUSG00000079139	Gm4204	8.92E-02	7.71E-01	no	-1.86E+00	2.66E-30	down
ENSMUSG00000078193	Gm2000	2.44E+00	0.00E+00	up	9.84E-01	7.26E-103	no
ENSMUSG00000053664	Uck2	-2.38E+00	6.67E-03	no	-2.62E+00	5.10E-04	down
ENSMUSG00000050229	Pigm	-2.77E+00	2.06E-05	down	-1.56E+00	7.49E-02	no
ENSMUSG00000078184	B020018G12Rik	2.81E+00	7.71E-17	up	6.81E-01	1.15E-02	no
ENSMUSG00000043429	4922505E12Rik	-4.63E+00	1.13E-61	down	-2.53E+00	2.81E-06	down
ENSMUSG00000044854	1700056E22Rik	-1.80E+00	2.87E-12	down	-6.87E-02	9.36E-01	no
ENSMUSG00000039224	D1Pas1	-3.19E+00	3.15E-43	down	-2.74E+00	6.77E-39	down
ENSMUSG00000091017	Fam71a	-4.47E+00	3.56E-39	down	-2.89E+00	1.59E-04	down
ENSMUSG00000046404	Yod1	-3.37E+00	2.42E-08	down	-2.17E+00	1.85E-02	no
ENSMUSG00000048174	Tmem81	-3.59E+00	6.76E-12	down	-2.15E+00	4.35E-03	no
ENSMUSG00000020423	Btg2	3.40E+00	1.63E-04	up	2.00E+00	1.56E-01	no
ENSMUSG00000055547	Apobec4	-4.36E+00	6.35E-51	down	-1.28E+00	4.13E-02	no
ENSMUSG00000040485	Lrrc52	-2.46E+00	4.12E-19	down	7.07E-01	2.45E-01	no
ENSMUSG00000042800	1700015E13Rik	-9.87E-01	2.52E-40	no	1.40E+00	3.96E-26	up
ENSMUSG00000073722	4931408C20Rik	-5.50E+00	5.21E-06	down	-3.69E+00	2.79E-01	no
ENSMUSG00000048411	Gm597	-5.84E+00	5.44E-08	down	-5.67E+00	4.05E-05	down
ENSMUSG00000038323	1700066M21Rik	-1.92E+00	1.30E-05	down	-2.39E+00	5.34E-10	down
ENSMUSG00000047383	Als2cr11	-4.49E+00	4.23E-225	down	-3.86E+00	9.21E-196	down
ENSMUSG00000026182	Tnp1	-5.32E+00	0.00E+00	down	-4.60E+00	0.00E+00	down
ENSMUSG00000032968	Inha	2.17E+00	2.39E-04	up	1.95E+00	7.15E-10	up
ENSMUSG00000072978	Gm5830	-1.55E+00	8.90E-02	no	-2.53E+00	4.94E-04	down
ENSMUSG00000016526	Dyrk3	-2.45E+00	2.03E-66	down	-1.30E+00	1.41E-12	down
ENSMUSG00000038370	Pcp4l1	4.94E+00	8.42E-04	up	2.86E+00	1.04E-01	no
ENSMUSG00000063659	Zbtb18	-2.87E+00	1.61E-03	no	-4.44E+00	8.94E-16	down
ENSMUSG00000057072	1700022P22Rik	-1.76E+00	4.10E-127	down	1.57E-01	3.78E-01	no
ENSMUSG00000026112	Coa5	-1.64E+00	8.34E-03	no	-2.71E+00	7.38E-10	down
ENSMUSG00000025961	4933402D24Rik	-2.08E+00	1.09E-04	down	-2.61E-01	8.73E-01	no
ENSMUSG00000045210	Vcpip1	-1.84E+00	2.68E-02	no	-2.96E+00	3.05E-05	down
ENSMUSG00000079330	Lemd1	-3.01E+00	1.84E-25	down	-1.42E+00	5.35E-03	no
ENSMUSG00000046062	Ppp1r15b	-1.77E+00	2.43E-02	no	-2.64E+00	9.19E-04	down
ENSMUSG00000050526	4933406M09Rik	-5.73E+00	4.87E-16	down	-4.69E+00	7.97E-03	no
ENSMUSG00000090623	Cfhr3	-3.07E+00	3.24E-05	down	-1.45E+00	4.11E-01	no
ENSMUSG00000079658	Tceb1	-1.22E+00	5.81E-20	down	-1.26E+00	2.78E-20	down
ENSMUSG00000038026	Kcnj9	-2.51E+00	3.22E-10	down	-3.02E+00	4.02E-31	down
ENSMUSG00000037499	Nenf	3.48E+00	1.54E-13	up	1.96E+00	5.55E-03	no
ENSMUSG00000061518	Cox5b	3.12E+00	1.95E-57	up	2.54E+00	1.08E-56	up
ENSMUSG00000010290	AI597479	-1.91E+00	3.12E-02	no	-2.39E+00	4.78E-04	down
ENSMUSG00000046330	Rpl37a	3.30E+00	3.12E-21	up	2.11E+00	2.82E-11	up
ENSMUSG00000046367	4931440L10Rik	-5.05E+00	1.63E-14	down	-3.41E+00	5.83E-03	no
ENSMUSG00000042111	Ccdc115	2.89E+00	4.30E-06	up	-6.90E-02	9.25E-01	no
ENSMUSG00000026125	Prss39	-3.45E+00	1.46E-185	down	-3.12E+00	2.10E-106	down
ENSMUSG00000026162	Nhej1	-1.27E-01	8.61E-01	no	-1.63E+00	1.44E-05	down
ENSMUSG00000026238	Ptma	3.17E+00	9.28E-20	up	2.58E-01	6.87E-01	no
ENSMUSG00000026374	Tsn	2.63E+00	1.57E-09	up	4.88E-01	1.83E-01	no
ENSMUSG00000026578	Ccdc181	-2.28E+00	1.02E-118	down	-1.78E+00	8.75E-87	down
ENSMUSG00000015962	1700016C15Rik	-4.33E+00	2.02E-48	down	-2.94E+00	5.40E-07	down
ENSMUSG00000008136	Fhl2	4.78E+00	2.67E-12	up	3.26E+00	6.55E-02	no
ENSMUSG00000026107	Nabp1	-2.25E+00	1.80E-19	down	-8.26E-01	9.94E-02	no
ENSMUSG00000043629	1700019D03Rik	-1.67E+00	2.01E-21	down	4.16E-02	9.44E-01	no
ENSMUSG00000073676	Hspe1	2.92E+00	5.54E-08	up	1.92E+00	3.43E-03	no

ENSMUSG00000096094	A630095N17Rik	-3.03E+00	7.04E-11	down	-4.44E-01	7.14E-01	no
ENSMUSG00000043230	Fam124b	-4.76E+00	1.55E-06	down	-1.90E+00	3.77E-01	no
ENSMUSG00000026279	Thap4	-2.35E+00	4.02E-32	down	-2.34E+00	2.97E-42	down
ENSMUSG00000026484	Rnf2	-1.09E+00	2.71E-02	no	-2.11E+00	7.58E-08	down
ENSMUSG00000026558	Uck2	-2.51E+00	2.30E-33	down	-1.38E+00	1.32E-08	down
ENSMUSG00000058715	Fcer1g	7.43E+00	8.35E-61	up	5.11E+00	7.68E-06	up
ENSMUSG00000038179	Slamf7	9.46E-01	3.00E-05	no	1.68E+00	1.45E-18	up
ENSMUSG00000026495	Efcab2	-9.54E-01	4.34E-05	no	-1.38E+00	3.54E-13	down
ENSMUSG00000038633	Degs1	2.64E+00	6.85E-04	up	4.19E-01	6.64E-01	no
ENSMUSG00000039384	Dusp10	-1.70E+00	2.54E-04	down	9.81E-01	2.90E-01	no
ENSMUSG00000037408	Cnnm4	-1.37E+00	1.82E-01	no	-3.39E+00	7.01E-07	down
ENSMUSG00000079470	Utp14b	-3.12E+00	9.20E-06	down	-2.80E+00	1.82E-03	no
ENSMUSG00000042305	Tmem183a	-1.66E+00	1.36E-05	down	-1.47E+00	6.20E-04	down
ENSMUSG00000026575	Nme7	-2.69E+00	5.08E-35	down	-1.78E+00	2.44E-17	down
ENSMUSG00000026094	Stk17b	-9.82E-01	2.96E-02	no	-1.87E+00	5.32E-06	down
ENSMUSG00000026021	Sumo1	4.97E-02	8.84E-01	no	-1.50E+00	2.68E-12	down
ENSMUSG00000034432	Cops8	1.14E+00	7.31E-04	up	-6.84E-01	2.80E-03	no
ENSMUSG00000026429	Ube2t	2.61E+00	1.53E-22	up	-1.26E-01	5.66E-01	no
ENSMUSG00000026729	4930562F07Rik	-2.69E+00	5.98E-04	down	-2.12E-01	9.28E-01	no
ENSMUSG00000058267	Mrps14	1.64E+00	1.35E-04	up	8.23E-01	4.40E-02	no
ENSMUSG00000026628	Atf3	4.10E+00	1.02E-13	up	3.70E+00	2.47E-04	up
ENSMUSG00000043015	Tmem194b	-3.37E+00	4.52E-04	down	-2.05E+00	3.45E-01	no
ENSMUSG00000060715	1700019A02Rik	-9.96E-01	1.31E-37	no	-1.21E+00	5.24E-69	down
ENSMUSG00000026039	Sgol2	-1.21E+00	3.07E-01	no	-3.08E+00	1.18E-06	down
ENSMUSG00000049339	Fam134a	-1.10E+00	1.10E-08	down	-1.44E+00	5.63E-20	down
ENSMUSG00000034220	Gpc1	-1.68E+00	1.91E-01	no	-3.27E+00	5.87E-05	down
ENSMUSG00000025925	Terf1	-1.36E+00	3.88E-05	down	-2.41E+00	8.98E-17	down
ENSMUSG00000042901	Aida	-2.37E-03	9.98E-01	no	-1.97E+00	4.58E-04	down
ENSMUSG00000037529	Prss40	-5.71E+00	5.10E-26	down	-5.34E+00	9.63E-16	down
ENSMUSG00000026311	Asb1	-1.37E+00	2.34E-02	no	-1.93E+00	1.30E-04	down
ENSMUSG00000025935	Tram1	-2.62E+00	1.34E-29	down	-2.89E+00	3.45E-48	down
ENSMUSG00000018189	Uchl5	-1.32E+00	2.09E-05	down	-1.84E+00	3.93E-13	down
ENSMUSG00000026698	Pigc	-2.42E+00	1.80E-04	down	-2.01E+00	1.69E-03	no
ENSMUSG00000026566	Mpzl1	1.85E+00	9.98E-05	up	1.22E+00	1.40E-02	no
ENSMUSG00000026687	Aldh9a1	-1.20E+00	1.35E-02	no	-2.61E+00	6.19E-13	down
ENSMUSG00000006412	Pfdn2	1.88E+00	8.34E-05	up	-5.18E-01	1.52E-01	no
ENSMUSG00000060679	Mrps9	-4.58E-01	1.95E-01	no	-1.22E+00	6.93E-07	down
ENSMUSG00000026018	Ica1l	-2.62E+00	2.13E-15	down	-2.15E+00	6.54E-11	down
ENSMUSG00000025963	Mdh1b	-2.90E+00	8.46E-45	down	-2.87E+00	9.91E-71	down
ENSMUSG00000039323	Igfbp2	4.89E+00	1.89E-12	up	3.62E+00	5.22E-06	up
ENSMUSG00000026255	Efhd1	-2.26E+00	3.76E-69	down	-2.34E+00	1.60E-100	down
ENSMUSG00000026421	Csrp1	2.89E+00	1.28E-04	up	-2.61E-01	9.02E-01	no
ENSMUSG00000040629	Mael	-1.66E+00	1.21E-204	down	-1.37E+00	2.02E-160	down
ENSMUSG00000026547	Tagln2	4.47E+00	2.38E-09	up	2.86E+00	2.45E-02	no
ENSMUSG00000049353	Rd3	-2.97E+00	1.87E-12	down	-6.98E-01	3.07E-01	no
ENSMUSG00000044457	Gm4776	-2.26E+00	6.56E-04	down	6.57E-01	7.53E-01	no
ENSMUSG00000051223	Bzw1	-1.38E+00	1.40E-06	down	-1.66E+00	4.32E-06	down
ENSMUSG00000041040	Fam117b	-2.34E+00	5.90E-05	down	-2.50E+00	1.33E-05	down
ENSMUSG00000026281	Dtymk	-3.37E-01	4.13E-01	no	-1.00E+00	1.34E-04	down
ENSMUSG00000026353	Ubxn4	-1.16E+00	5.54E-06	down	-1.41E+00	1.41E-08	down
ENSMUSG00000040843	Tiprl	-1.57E+00	1.53E-15	down	-1.53E+00	2.37E-13	down
ENSMUSG00000040713	Creg1	3.16E+00	7.57E-05	up	5.45E-01	6.18E-01	no
ENSMUSG00000049598	Vsig8	1.14E+00	1.38E-01	no	4.17E+00	5.64E-04	up
ENSMUSG00000072258	Taf1a	-2.12E+00	5.29E-03	no	-2.57E+00	1.00E-04	down
ENSMUSG00000041809	Efhc1	-3.54E+00	2.03E-122	down	-2.94E+00	1.70E-87	down
ENSMUSG00000073616	Myeov2	2.49E+00	2.42E-79	up	1.72E+00	2.30E-68	up
ENSMUSG00000086277	4930558K02Rik	-2.64E+00	7.94E-13	down	2.00E-01	8.57E-01	no
ENSMUSG00000026554	Dcaf8	-1.83E+00	1.67E-09	down	-2.33E+00	3.80E-19	down
ENSMUSG00000004880	Lbr	6.58E-01	4.72E-01	no	-1.86E+00	5.37E-05	down
ENSMUSG00000026134	Prim2	-3.96E-01	4.91E-01	no	-1.64E+00	3.82E-05	down
ENSMUSG00000033793	Atp6v1h	-1.90E-02	9.72E-01	no	-2.41E+00	4.56E-20	down
ENSMUSG00000026142	Rhbdd1	-3.33E+00	1.56E-10	down	-2.50E+00	6.30E-05	down
ENSMUSG00000026234	Ncl	3.08E+00	1.40E-13	up	4.93E-01	4.28E-01	no
ENSMUSG00000026331	Slco6c1	-4.19E+00	1.22E-64	down	-3.80E+00	2.52E-67	down

ENSMUSG00000053286	Trmt1l	-1.67E+00	1.16E-02	no	-2.07E+00	1.16E-05	down
ENSMUSG00000058076	Sdhc	1.43E+00	1.73E-04	up	-8.48E-02	8.56E-01	no
ENSMUSG00000038235	F11r	4.28E+00	5.68E-05	up	2.07E+00	6.06E-02	no
ENSMUSG00000037474	Dtl	-1.14E+00	5.81E-02	no	-2.83E+00	1.72E-11	down
ENSMUSG00000026126	Ptpn18	3.29E+00	3.93E-04	up	-1.20E-01	9.57E-01	no
ENSMUSG00000047528	Als2cr12	-1.88E+00	5.14E-23	down	-1.77E+00	4.29E-27	down
ENSMUSG00000026020	Nop58	-9.81E-01	1.20E-04	no	-1.79E+00	1.67E-12	down
ENSMUSG00000034292	Traf3ip1	-2.66E+00	5.58E-15	down	-2.10E+00	4.78E-09	down
ENSMUSG00000025917	Cops5	-1.62E+00	2.21E-16	down	-1.46E+00	1.18E-17	down
ENSMUSG00000026600	Soat1	-2.07E+00	1.33E-01	no	-3.45E+00	4.89E-04	down
ENSMUSG00000005681	Apoa2	4.59E+00	2.91E-16	up	2.29E+00	3.91E-04	up
ENSMUSG00000062169	Cnih4	4.33E+00	1.52E-09	up	1.14E+00	7.01E-02	no
ENSMUSG00000037568	Vash2	-4.71E+00	3.78E-33	down	-1.94E+00	1.33E-02	no
ENSMUSG00000055833	1700034H15Rik	-4.26E+00	1.37E-04	down	-1.99E+00	3.28E-01	no
ENSMUSG00000026638	Irf6	-3.62E+00	6.91E-04	down	-1.67E-01	9.58E-01	no
ENSMUSG00000016494	Cd34	2.70E+00	1.56E-06	up	1.36E+00	4.11E-01	no
ENSMUSG00000073725	Lmbrd1	-2.40E+00	4.83E-07	down	-7.85E-01	5.01E-01	no
ENSMUSG00000033124	Atg9a	-3.70E+00	2.35E-51	down	-2.70E+00	1.22E-24	down
ENSMUSG00000070732	Rbm44	-1.91E+00	1.24E-05	down	-2.82E+00	1.38E-20	down
ENSMUSG00000026581	Sell	-1.90E+00	1.41E-01	no	-2.49E+00	8.00E-04	down
ENSMUSG00000026611	Spata17	-2.26E+00	5.46E-31	down	-1.46E+00	5.79E-11	down
ENSMUSG00000025986	Slc39a10	-3.45E+00	2.04E-12	down	-2.28E+00	3.59E-04	down
ENSMUSG00000073650	Gm216	-2.87E+00	1.20E-10	down	-2.19E+00	2.99E-06	down
ENSMUSG00000033159	Cnppd1	-9.54E-01	2.95E-02	no	-1.68E+00	3.79E-07	down
ENSMUSG00000026203	Dnajb2	-2.59E+00	2.26E-44	down	-1.81E+00	3.89E-27	down
ENSMUSG00000073633	Fbxo36	-1.54E+00	4.77E-15	down	-6.81E-01	2.86E-04	no
ENSMUSG00000033701	Acbd6	-1.49E+00	5.04E-17	down	-1.53E+00	2.83E-29	down
ENSMUSG00000059498	Fcgr3	-1.32E-02	9.82E-01	no	-1.57E+00	5.15E-05	down
ENSMUSG00000072295	Als2cr11	-3.12E+00	1.12E-81	down	-2.51E+00	2.83E-57	down
ENSMUSG00000026417	Pigr	5.47E+00	1.02E-03	no	5.51E+00	3.72E-04	up
ENSMUSG00000041570	Camsap2	-3.70E+00	6.07E-06	down	-3.23E+00	5.01E-03	no
ENSMUSG00000033557	Fam20b	-2.76E+00	1.90E-02	no	-3.49E+00	2.27E-04	down
ENSMUSG00000040918	Slc19a2	-7.27E-01	4.63E-01	no	-2.18E+00	7.23E-04	down
ENSMUSG00000026571	Dcaf6	-5.82E+00	6.41E-59	down	-4.92E+00	2.41E-49	down
ENSMUSG00000026301	Iqca	-2.24E+00	9.59E-08	down	1.08E+00	2.56E-01	no
ENSMUSG00000026356	Dars	-1.48E+00	3.47E-05	down	-2.73E+00	1.10E-22	down
ENSMUSG00000042708	Shcbp1l	-1.71E+00	1.32E-19	down	-2.39E+00	1.36E-109	down
ENSMUSG00000040782	Rfwd2	-2.27E+00	1.48E-02	no	-2.97E+00	4.41E-05	down
ENSMUSG00000026563	Tada1	-6.34E-01	4.55E-01	no	-1.75E+00	3.58E-04	down
ENSMUSG00000007122	Casq1	-3.84E+00	3.50E-06	down	-3.17E-01	8.88E-01	no
ENSMUSG00000057335	Cep170	-3.73E+00	3.15E-14	down	-2.80E+00	3.43E-05	down
ENSMUSG00000037461	Ints7	-3.32E-01	6.75E-01	no	-2.35E+00	3.46E-06	down
ENSMUSG00000026622	Nek2	-1.73E+00	8.58E-10	down	-2.37E+00	3.08E-30	down
ENSMUSG00000003135	Cnot11	-7.78E-01	3.16E-01	no	-1.80E+00	4.28E-04	down
ENSMUSG00000047361	Gm973	-2.15E+00	5.26E-16	down	-1.05E+00	1.68E-04	down
ENSMUSG00000026401	Daf2	-2.61E+00	8.83E-52	down	-2.45E+00	1.28E-42	down
ENSMUSG00000038949	Cnst	-3.41E+00	2.39E-03	no	-3.33E+00	9.66E-05	down
ENSMUSG00000037395	Rcor3	-2.80E+00	4.71E-12	down	-2.60E+00	4.79E-15	down
ENSMUSG00000026153	Fam135a	-2.85E+00	3.20E-10	down	-3.02E+00	1.40E-14	down
ENSMUSG00000010453	Kansl3	-2.16E+00	7.08E-08	down	-3.52E+00	7.68E-36	down
ENSMUSG00000070871	Ccnyl1	-3.19E+00	4.88E-13	down	-3.37E+00	3.50E-15	down
ENSMUSG00000026187	Xrcc5	2.57E+00	3.11E-09	up	1.68E+00	7.87E-09	up
ENSMUSG00000044340	Phlpp1	-5.16E+00	3.20E-26	down	-1.99E+00	4.55E-02	no
ENSMUSG00000071890	Mroh9	-4.64E+00	1.02E-29	down	-1.76E+00	4.62E-02	no
ENSMUSG00000015961	Adss	-2.79E+00	1.60E-11	down	-3.42E+00	2.32E-20	down
ENSMUSG00000057173	Rfx8	-2.25E+00	9.09E-17	down	-2.51E+00	1.25E-29	down
ENSMUSG00000006299	Aamp	-2.18E+00	3.51E-97	down	-2.30E+00	5.48E-148	down
ENSMUSG00000054387	Mdm4	-2.21E+00	1.45E-06	down	-2.53E+00	1.68E-08	down
ENSMUSG00000044835	Ankrd45	-2.92E+00	1.24E-10	down	-1.85E+00	2.40E-04	down
ENSMUSG00000007107	Atp1a4	-4.27E+00	1.82E-108	down	-1.50E+00	1.54E-05	down
ENSMUSG00000026496	Parp1	-8.72E-01	7.41E-02	no	-2.53E+00	8.72E-15	down
ENSMUSG00000026516	Nvl	-1.09E+00	1.75E-01	no	-2.23E+00	2.07E-04	down
ENSMUSG00000026115	Vwa3b	-4.08E+00	1.93E-81	down	-1.53E+00	8.65E-06	down
ENSMUSG00000026102	Inpp1	-3.82E+00	1.66E-07	down	-1.50E+00	1.82E-01	no

ENSMUSG00000026385	Dbi	2.10E+00	3.08E-14	up	7.93E-01	5.90E-04	no
ENSMUSG00000040297	Suco	-3.07E+00	6.51E-25	down	-3.30E+00	6.19E-31	down
ENSMUSG00000026526	Fh1	-2.25E+00	1.34E-04	down	-5.78E-01	6.81E-01	no
ENSMUSG00000039342	Ankar	-3.65E+00	1.07E-16	down	-3.79E+00	4.19E-36	down
ENSMUSG00000067028	Cntnap5b	-5.89E+00	5.12E-12	down	-3.69E+00	2.88E-02	no
ENSMUSG00000001674	Ddx18	2.07E+00	9.21E-09	up	1.20E+00	6.09E-06	up
ENSMUSG00000090553	Snrpe	4.29E+00	3.15E-51	up	1.56E+00	3.51E-14	up
ENSMUSG00000047369	Dnahc14	-5.66E+00	3.28E-07	down	-5.57E+00	1.99E-13	down
ENSMUSG00000058407	Txndc9	-1.76E+00	1.53E-04	down	-1.76E+00	2.03E-04	down
ENSMUSG00000025982	Sf3b1	-7.48E-02	8.43E-01	no	-1.42E+00	8.18E-10	down
ENSMUSG00000043716	Rpl7	-5.17E-01	1.87E-01	no	-1.86E+00	1.86E-09	down
ENSMUSG00000026623	Lpgat1	-2.32E+00	3.69E-06	down	-2.63E+00	2.98E-11	down
ENSMUSG00000026095	Asnsd1	-3.26E+00	9.13E-40	down	-3.07E+00	3.02E-46	down
ENSMUSG00000038079	Tmem237	-1.95E+00	1.34E-10	down	-1.45E+00	3.51E-05	down
ENSMUSG00000033364	Usp37	-3.78E+00	8.03E-14	down	-2.81E+00	3.05E-05	down
ENSMUSG00000026159	Agfg1	-2.61E+00	1.92E-22	down	-2.77E+00	9.48E-23	down
ENSMUSG00000056211	R3hdm1	-3.46E+00	8.12E-28	down	-3.15E+00	3.55E-20	down
ENSMUSG00000062963	Ufc1	-1.43E+00	7.80E-04	down	-1.80E+00	5.56E-07	down
ENSMUSG00000058729	Lin9	-1.41E+00	1.91E-01	no	-3.31E+00	2.97E-07	down
ENSMUSG00000038776	Ephx1	3.50E+00	9.79E-04	up	3.71E+00	2.45E-04	up
ENSMUSG00000026111	Unc50	1.32E+00	1.93E-03	no	-1.16E+00	2.34E-06	down
ENSMUSG00000025978	Rftn2	-3.21E+00	3.71E-05	down	-2.39E+00	6.78E-03	no
ENSMUSG00000036707	Cab39	-2.10E+00	1.18E-07	down	-3.22E+00	7.70E-21	down
ENSMUSG00000044783	Hjurp	-1.17E+00	1.72E-01	no	-2.65E+00	2.45E-05	down
ENSMUSG00000042699	Dhx9	-2.03E+00	3.40E-03	no	-3.93E+00	1.47E-17	down
ENSMUSG00000026333	Gin1	-6.90E-01	4.92E-01	no	-2.22E+00	8.59E-05	down
ENSMUSG00000018196	Glrx2	-3.62E+00	3.07E-13	down	-2.15E+00	5.71E-03	no
ENSMUSG00000026683	Nuf2	-2.18E+00	1.84E-11	down	-2.75E+00	1.18E-25	down
ENSMUSG00000041926	Rnpep	-3.02E+00	4.11E-06	down	-3.09E+00	4.07E-05	down
ENSMUSG00000026721	Rabgap1l	-1.56E+00	8.72E-02	no	-2.77E+00	1.82E-05	down
ENSMUSG00000041763	Tpp2	-3.36E+00	2.01E-33	down	-3.34E+00	4.71E-34	down
ENSMUSG00000070738	Dgkd	-2.25E+00	8.24E-10	down	-2.69E+00	8.00E-17	down
ENSMUSG00000026577	Blzf1	-2.12E+00	2.98E-04	down	-2.11E+00	5.46E-04	down
ENSMUSG00000050122	Vwa3b	-3.09E+00	3.80E-20	down	-1.28E+00	3.38E-03	no
ENSMUSG00000026615	Eprs	-1.02E+00	2.63E-03	no	-1.26E+00	9.72E-05	down
ENSMUSG00000006304	Arpc2	-1.29E+00	2.19E-14	down	-1.59E+00	5.96E-24	down
ENSMUSG00000026163	Sphkap	-4.58E+00	2.83E-12	down	-4.43E+00	4.16E-14	down
ENSMUSG00000048865	Arhgap30	-4.35E+00	7.46E-05	down	-4.12E+00	3.44E-02	no
ENSMUSG00000026270	Capn10	-7.33E-01	4.66E-01	no	-1.77E+00	4.94E-04	down
ENSMUSG00000038866	Zcchc2	-4.16E+00	2.67E-17	down	-3.78E+00	4.69E-17	down
ENSMUSG00000042554	Zp3r	-4.08E+00	1.64E-71	down	-1.16E+00	1.13E-02	no
ENSMUSG00000026450	Chit1	-3.39E+00	8.37E-07	down	-6.73E-01	7.03E-01	no
ENSMUSG00000038936	Sccpdh	-1.94E+00	6.46E-43	down	-1.89E+00	2.40E-48	down
ENSMUSG00000025955	Akr1cl	3.68E+00	4.22E-04	up	3.40E+00	1.01E-04	up
ENSMUSG00000026226	Spata3	-3.06E+00	8.78E-68	down	-1.36E+00	2.36E-05	down
ENSMUSG00000025939	Ube2w	-1.72E+00	3.68E-03	no	-2.19E+00	4.91E-04	down
ENSMUSG00000055322	Tns1	-3.48E+00	3.14E-31	down	-8.94E-01	1.66E-01	no
ENSMUSG00000026245	Farsb	-9.15E-01	2.56E-02	no	-1.76E+00	3.04E-09	down
ENSMUSG00000033684	Qsox1	-1.50E+00	3.08E-02	no	-2.53E+00	8.92E-05	down
ENSMUSG00000025962	Fastkd2	-3.38E+00	1.24E-06	down	-2.26E+00	1.14E-02	no
ENSMUSG00000034212	Ankmy1	-4.47E+00	1.18E-14	down	-3.26E+00	1.90E-07	down
ENSMUSG00000026277	Stk25	-9.13E-01	1.65E-04	no	-1.93E+00	5.74E-29	down
ENSMUSG00000025912	Mybl1	-3.42E+00	3.65E-06	down	-4.56E+00	4.21E-18	down
ENSMUSG00000023150	Ivns1abp	-2.47E+00	2.64E-16	down	-2.77E+00	8.91E-19	down
ENSMUSG00000025967	Eef1b2	1.44E-01	7.37E-01	no	-1.11E+00	2.13E-06	down
ENSMUSG00000041605	5730559C18Rik	-3.77E+00	3.16E-06	down	-3.54E+00	1.07E-04	down
ENSMUSG00000025916	Ppp1r42	-3.99E+00	9.74E-70	down	-3.71E+00	1.90E-62	down
ENSMUSG00000026546	Ccdc19	-2.82E+00	8.54E-90	down	-1.76E+00	5.67E-29	down
ENSMUSG00000026201	Stk16	-5.28E-01	2.57E-01	no	-1.23E+00	6.71E-05	down
ENSMUSG00000026004	Kansl1l	-2.64E+00	2.92E-05	down	-2.94E+00	3.62E-06	down
ENSMUSG00000040423	Rc3h1	-3.28E+00	2.35E-05	down	-3.48E+00	2.78E-04	down
ENSMUSG00000026585	Kifap3	-2.91E+00	7.89E-63	down	-9.71E-01	4.13E-04	no
ENSMUSG00000026116	Tmem131	-2.29E+00	4.19E-03	no	-3.52E+00	1.67E-06	down
ENSMUSG00000026096	Osgepl1	-1.97E+00	7.71E-03	no	-2.31E+00	9.23E-06	down

ENSMUSG00000026219	Trip12	-4.24E+00	5.07E-68	down	-4.40E+00	3.79E-80	down
ENSMUSG00000026455	Klhl12	-2.78E+00	1.02E-05	down	-1.15E+00	3.35E-01	no
ENSMUSG00000013973	Dedd	-2.34E+00	1.33E-04	down	-1.20E+00	1.77E-01	no
ENSMUSG00000034343	Ube2f	-1.69E+00	1.93E-05	down	-1.98E+00	5.93E-06	down
ENSMUSG00000013997	Nit1	-2.58E+00	1.85E-06	down	-2.22E+00	6.15E-07	down
ENSMUSG00000039630	Hnrnpu	-2.23E+00	1.94E-38	down	-2.53E+00	5.99E-48	down
ENSMUSG00000053333	Dis3l2	-2.66E+00	3.24E-09	down	-1.65E+00	2.09E-02	no
ENSMUSG00000026274	Pask	-3.07E+00	6.24E-10	down	-2.84E+00	7.48E-11	down
ENSMUSG00000060985	Tdrd5	-8.91E-01	2.57E-01	no	-2.74E+00	4.09E-15	down
ENSMUSG00000053153	Spag16	-2.78E+00	2.51E-16	down	-2.30E+00	5.80E-15	down
ENSMUSG00000026592	Tex35	-3.91E+00	3.43E-101	down	-2.18E+00	4.68E-12	down
ENSMUSG00000026336	Slco6d1	-3.96E+00	7.00E-20	down	-3.95E+00	1.35E-27	down
ENSMUSG00000042641	Rgsl1	-3.74E+00	2.58E-09	down	-8.40E-01	6.33E-01	no
ENSMUSG00000025968	Ndufs1	-1.67E+00	5.05E-06	down	-2.08E+00	4.59E-10	down
ENSMUSG00000053161	Daw1	-2.22E+00	1.97E-07	down	-2.36E+00	5.75E-16	down
ENSMUSG00000062939	Stat4	-4.77E+00	2.05E-124	down	-1.98E+00	1.84E-07	down
ENSMUSG00000041757	Plekha6	-5.08E+00	1.07E-17	down	-1.59E+00	1.77E-01	no
ENSMUSG00000004364	Cul3	-4.06E+00	2.25E-67	down	-3.88E+00	2.00E-63	down
ENSMUSG00000026696	Vamp4	-2.11E+00	1.31E-03	no	-3.86E+00	1.52E-17	down
ENSMUSG00000047021	Ccdc108	-4.35E+00	8.28E-30	down	-4.71E+00	1.08E-68	down
ENSMUSG00000036086	Zranb3	-2.72E+00	8.08E-03	no	-3.97E+00	8.26E-06	down
ENSMUSG00000026632	Tatdn3	-1.96E+00	5.02E-02	no	-2.64E+00	7.80E-07	down
ENSMUSG00000046337	Fam178b	-1.71E+00	7.47E-27	down	-1.39E+00	2.47E-23	down
ENSMUSG00000036962	Gm101	-2.51E+00	9.11E-09	down	-1.42E+00	1.90E-03	no
ENSMUSG00000056050	Mia3	-2.54E+00	1.06E-08	down	-2.17E+00	5.12E-06	down
ENSMUSG00000064294	Aox3	-4.06E+00	2.70E-05	down	-7.52E-01	7.95E-01	no
ENSMUSG00000026254	Eif4e2	-1.86E+00	1.69E-07	down	-2.24E+00	8.72E-12	down
ENSMUSG00000026523	Wdr64	-5.31E+00	1.64E-28	down	-2.92E+00	3.99E-03	no
ENSMUSG00000026155	Smap1	-1.04E+00	1.16E-05	down	7.01E-01	1.52E-01	no
ENSMUSG00000034088	Hdlbp	-3.71E+00	3.99E-56	down	-3.54E+00	5.47E-55	down
ENSMUSG00000026469	Xpr1	-3.05E+00	3.55E-05	down	-2.59E+00	5.98E-03	no
ENSMUSG00000026491	Ahctf1	-3.66E+00	2.81E-21	down	-3.41E+00	4.63E-18	down
ENSMUSG00000025977	Boll	-1.72E+00	3.81E-06	down	-2.73E+00	3.27E-23	down
ENSMUSG00000026017	Carf	-2.81E+00	1.18E-04	down	-3.73E+00	3.16E-08	down
ENSMUSG00000026019	Wdr12	-1.81E+00	1.78E-09	down	-1.74E+00	7.74E-12	down
ENSMUSG00000003458	Ncstn	-5.24E-01	5.00E-01	no	-2.15E+00	2.76E-04	down
ENSMUSG00000038733	Wdr26	-2.86E+00	1.61E-13	down	-2.30E+00	9.90E-07	down
ENSMUSG00000026199	Ankzf1	-2.63E+00	1.14E-05	down	-2.98E+00	1.63E-08	down
ENSMUSG00000026171	Rnf25	-3.04E+00	2.59E-33	down	-1.67E+00	1.94E-07	down
ENSMUSG00000026307	Scly	-3.82E+00	1.89E-04	down	-4.25E+00	3.80E-09	down
ENSMUSG00000026200	Glb1l	-3.86E+00	3.54E-15	down	-3.37E+00	3.87E-14	down
ENSMUSG00000032883	Acsl3	2.08E-01	8.01E-01	no	-2.14E+00	3.08E-07	down
ENSMUSG00000026584	Scyl3	-5.64E-01	5.51E-01	no	-2.49E+00	6.04E-05	down
ENSMUSG00000016493	Cd46	-4.88E+00	1.54E-04	down	-2.82E+00	3.76E-01	no
ENSMUSG00000033671	Cep350	-4.17E+00	4.27E-15	down	-3.70E+00	4.71E-12	down
ENSMUSG00000041144	Dnahc7b	-3.35E+00	1.08E-04	down	-3.70E+00	8.09E-10	down
ENSMUSG00000096141	Dnahc7a	-3.25E+00	1.77E-05	down	-3.39E+00	8.59E-09	down
ENSMUSG00000067851	Arfgef1	-2.34E+00	3.38E-06	down	-2.86E+00	5.93E-06	down
ENSMUSG00000042207	Kdm5b	-1.79E+00	9.83E-08	down	-2.01E+00	1.55E-14	down
ENSMUSG00000042772	Smg7	-4.71E+00	9.31E-21	down	-3.55E+00	3.89E-09	down
ENSMUSG00000026049	Tex30	4.42E-01	3.66E-02	no	-1.16E+00	6.41E-17	down
ENSMUSG00000026229	Psmd1	1.45E-01	7.65E-01	no	-1.24E+00	2.47E-04	down
ENSMUSG00000026269	Rnpepl1	-3.72E+00	7.60E-04	down	-2.10E+00	4.11E-01	no
ENSMUSG00000039210	Gpatch2	-3.03E+00	1.50E-07	down	-2.84E+00	4.66E-07	down
ENSMUSG00000026504	Sdccag8	-2.70E+00	8.27E-08	down	-2.57E+00	3.84E-07	down
ENSMUSG00000022995	Enah	-5.28E+00	1.34E-34	down	-2.61E+00	2.24E-04	down
ENSMUSG00000042581	Thsd7b	-3.40E+00	3.23E-03	no	-3.51E+00	6.99E-04	down
ENSMUSG00000026594	Ralgps2	-1.71E+00	2.06E-10	down	-1.97E+00	8.48E-20	down
ENSMUSG00000053483	Usp21	-8.89E-01	3.49E-01	no	-1.89E+00	6.39E-04	down
ENSMUSG00000026601	Axdnd1	-4.14E+00	4.88E-36	down	-3.75E+00	9.77E-31	down
ENSMUSG00000033257	Ttll4	-3.94E+00	9.70E-13	down	-2.34E+00	1.21E-03	no
ENSMUSG00000073643	Wdfy1	-2.02E+00	1.26E-07	down	-1.10E+00	1.43E-02	no
ENSMUSG00000042197	Zfp451	-3.49E+00	5.10E-19	down	-1.39E+00	3.75E-02	no
ENSMUSG00000033021	Gmppa	-1.60E+00	2.32E-04	down	-1.39E+00	1.42E-04	down

ENSMUSG00000025983	Ccdc150	-4.47E+00	1.84E-33	down	-2.52E+00	6.13E-05	down
ENSMUSG00000060771	Tsga10	-4.04E+00	1.32E-28	down	-3.40E+00	6.96E-19	down
ENSMUSG00000026150	Mff	-1.75E+00	1.40E-19	down	-1.77E+00	2.32E-20	down
ENSMUSG00000026466	Tor1aip1	-3.77E+00	1.01E-32	down	-2.25E+00	3.42E-05	down
ENSMUSG00000041642	Kif21b	-4.01E+00	7.93E-04	down	-3.36E+00	5.93E-02	no
ENSMUSG00000040648	Ppip5k2	-3.07E+00	3.78E-03	no	-3.46E+00	6.99E-04	down
ENSMUSG00000026384	Ptpn4	-3.87E+00	1.69E-14	down	-2.46E+00	6.99E-04	down
ENSMUSG00000079429	Mroh2a	-4.13E+00	1.20E-22	down	-3.95E+00	1.47E-30	down
ENSMUSG00000026463	Atp2b4	-3.11E+00	7.26E-05	down	-3.78E+00	2.29E-11	down
ENSMUSG00000026276	43710	-2.58E+00	2.34E-13	down	-3.76E+00	7.59E-34	down
ENSMUSG00000025907	Rb1cc1	-3.78E+00	4.68E-21	down	-3.95E+00	3.91E-29	down
ENSMUSG00000026553	Copa	-7.86E-01	2.82E-01	no	-2.54E+00	3.95E-07	down
ENSMUSG00000026173	Plcd4	-3.94E+00	2.30E-25	down	-2.44E+00	1.51E-07	down
ENSMUSG00000033276	Stk36	-3.48E+00	2.07E-20	down	-1.58E+00	1.39E-03	no
ENSMUSG00000064302	Clasp1	-3.91E+00	4.62E-07	down	-2.92E+00	9.22E-03	no
ENSMUSG00000026567	Adcy10	-4.04E+00	2.70E-45	down	-2.07E+00	5.13E-10	down
ENSMUSG00000062590	Armc9	-1.95E+00	2.10E-02	no	-2.35E+00	1.67E-04	down
ENSMUSG00000015222	Map2	-4.40E+00	1.10E-05	down	-1.64E+00	5.14E-01	no
ENSMUSG00000026131	Dst	-3.11E+00	7.40E-04	down	-2.13E+00	3.27E-02	no
ENSMUSG00000078348	Sf3b5	2.15E+00	1.80E-20	up	9.14E-01	6.35E-09	no
ENSMUSG00000069713	4933406P04Rik	-1.40E+00	2.18E-01	no	-3.49E+00	1.07E-12	down
ENSMUSG00000069712	4930444G20Rik	-5.43E+00	7.10E-08	down	-5.20E+00	7.43E-02	no
ENSMUSG00000069682	Gm10275	-7.21E-01	4.44E-14	no	-1.83E+00	7.70E-130	down
ENSMUSG00000039684	Gm5422	-1.68E+00	2.37E-05	down	-1.27E+00	2.56E-03	no
ENSMUSG00000039485	Tspyl4	-1.38E+00	4.72E-01	no	-3.14E+00	1.07E-04	down
ENSMUSG00000045886	Gm9803	2.35E+00	2.84E-230	up	2.10E+00	1.03E-251	up
ENSMUSG00000062224	4933411G06Rik	-3.10E+00	2.19E-36	down	-8.63E-01	5.59E-02	no
ENSMUSG00000091526	BB019430	-3.76E+00	1.76E-09	down	-1.42E+00	3.76E-01	no
ENSMUSG00000053830	Gm9923	-2.57E+00	3.21E-04	down	-1.25E+00	2.42E-01	no
ENSMUSG00000064317	Gm10146	5.45E+00	6.97E-211	up	2.34E+00	2.65E-114	up
ENSMUSG00000044581	4932415D10Rik	-4.83E+00	3.69E-64	down	-1.83E+00	1.67E-05	down
ENSMUSG00000050490	Gm8394	1.14E+00	4.81E-05	up	7.01E-01	4.60E-03	no
ENSMUSG00000090816	Gm6729	-5.80E+00	2.21E-08	down	-2.83E+00	1.80E-01	no
ENSMUSG00000090308	Gm5174	-4.54E+00	5.40E-04	down	-3.45E+00	1.28E-01	no
ENSMUSG00000056366	Fabp3-ps1	2.40E+00	2.93E-08	up	2.67E+00	1.14E-14	up
ENSMUSG00000047025	Ccer1	-2.55E+00	4.51E-125	down	-8.65E-02	7.98E-01	no
ENSMUSG00000046934	Csl	-2.94E+00	4.96E-76	down	-2.29E+00	1.51E-56	down
ENSMUSG00000091086	Gm5428	-6.17E-01	4.58E-02	no	-1.68E+00	3.30E-12	down
ENSMUSG00000074734	4933416C03Rik	-2.08E+00	7.28E-31	down	-2.35E+00	2.46E-52	down
ENSMUSG00000044921	Rassf9	-2.52E+00	5.84E-09	down	6.80E-01	5.45E-01	no
ENSMUSG00000035759	Bbs10	-2.66E+00	2.28E-03	no	-3.28E+00	1.60E-06	down
ENSMUSG00000039910	Cited2	4.82E+00	4.82E-09	up	2.25E+00	1.68E-03	no
ENSMUSG00000044624	Gm4922	-4.05E+00	1.34E-08	down	-2.81E+00	1.09E-02	no
ENSMUSG00000047669	Msl3l2	-1.82E+00	7.67E-10	down	-1.34E+00	3.85E-07	down
ENSMUSG00000045391	1700120B22Rik	-2.89E+00	1.99E-07	down	-1.54E+00	1.09E-01	no
ENSMUSG00000050035	Fhl4	-2.95E+00	0.00E+00	down	-2.43E+00	0.00E+00	down
ENSMUSG00000074780	Anapc15-ps	-1.57E-01	7.39E-01	no	-1.28E+00	5.91E-08	down
ENSMUSG00000036478	Btg1	-2.05E+00	7.63E-12	down	-2.18E+00	8.63E-09	down
ENSMUSG00000028630	Dyrk2	-2.94E+00	9.33E-09	down	3.45E-02	9.82E-01	no
ENSMUSG00000019865	Nmbr	-2.92E+00	4.85E-02	no	-4.93E+00	7.95E-09	down
ENSMUSG00000050844	1700020N01Rik	1.64E+00	4.79E-54	up	1.19E+00	5.71E-34	up
ENSMUSG00000075266	Cenpw	2.41E+00	4.32E-26	up	1.73E+00	1.73E-22	up
ENSMUSG00000037710	Cisd1	2.02E+00	1.08E-21	up	-1.11E-01	5.63E-01	no
ENSMUSG00000033307	Mif	2.80E+00	2.30E-16	up	1.40E+00	7.82E-05	up
ENSMUSG00000096573	1700009J07Rik	-5.65E+00	1.27E-61	down	-4.60E+00	2.31E-12	down
ENSMUSG00000005054	Cstb	5.09E+00	8.27E-27	up	2.71E+00	2.56E-07	up
ENSMUSG00000020219	Timm13	2.85E+00	8.04E-32	up	1.65E+00	4.32E-23	up
ENSMUSG00000049038	Mterfd3	-3.18E+00	1.14E-05	down	-3.24E+00	5.82E-06	down
ENSMUSG00000069516	Lyz2	5.54E+00	1.37E-155	up	4.85E+00	7.46E-25	up
ENSMUSG00000040280	Ndufa4l2	-1.63E+00	3.49E-10	down	-1.91E+00	1.20E-16	down
ENSMUSG00000074639	BC089597	3.47E+00	6.39E-04	up	7.25E+00	1.74E-04	up
ENSMUSG00000039879	Heca	-2.82E+00	4.25E-03	no	-2.77E+00	8.71E-04	down
ENSMUSG00000038587	Akap12	-5.10E+00	2.89E-273	down	-3.13E+00	3.82E-48	down
ENSMUSG00000020087	Tysnd1	-5.40E-02	9.57E-01	no	-1.73E+00	1.17E-04	down

ENSMUSG00000020180	Snrpd3	8.35E-01	1.95E-05	no	-1.14E+00	1.42E-17	down
ENSMUSG00000049422	Chchd10	5.07E+00	3.49E-30	up	3.66E+00	1.17E-30	up
ENSMUSG00000035215	Lsm7	3.70E+00	1.13E-08	up	1.69E+00	1.63E-03	no
ENSMUSG00000015312	Gadd45b	4.90E+00	2.54E-30	up	1.41E+00	9.78E-03	no
ENSMUSG00000020018	Snrpf	1.91E+00	4.46E-16	up	1.37E+00	8.39E-09	up
ENSMUSG00000097407	4933408J17Rik	1.50E+00	4.41E-03	no	4.07E+00	2.98E-16	up
ENSMUSG00000020020	Usp44	-3.95E+00	3.73E-29	down	-1.24E+00	1.79E-02	no
ENSMUSG00000074781	Ube2n	-2.28E+00	2.34E-10	down	-1.78E+00	3.78E-05	down
ENSMUSG00000020213	Glipr1l1	-2.61E+00	8.70E-82	down	-1.46E+00	8.41E-15	down
ENSMUSG00000062074	Wisp3	3.67E+00	5.09E-03	no	3.76E+00	7.79E-04	up
ENSMUSG00000038583	Pln	-2.36E+00	2.59E-05	down	-4.94E-01	7.65E-01	no
ENSMUSG00000034932	Mrpl54	1.40E+00	1.00E-43	up	1.11E+00	1.22E-39	up
ENSMUSG00000046841	Ckap4	-3.15E+00	2.91E-24	down	-3.27E+00	3.22E-25	down
ENSMUSG00000020029	Nudt4	-3.20E+00	3.19E-161	down	-3.11E+00	9.13E-177	down
ENSMUSG00000020186	Csrp2	-2.04E+00	1.93E-06	down	-2.75E+00	2.60E-14	down
ENSMUSG00000047648	Fbxo30	-2.55E+00	4.06E-33	down	-1.88E+00	3.73E-13	down
ENSMUSG00000006736	Tspan31	4.06E+00	1.06E-05	up	3.95E-01	7.71E-01	no
ENSMUSG00000025381	Cnpy2	3.81E+00	5.36E-06	up	1.73E+00	7.36E-02	no
ENSMUSG00000090841	Myl6	1.32E+00	1.29E-06	up	-7.89E-01	6.43E-04	no
ENSMUSG00000000711	Rab5b	-2.22E+00	1.90E-03	no	-3.83E+00	1.15E-08	down
ENSMUSG00000064030	Wibg	-2.35E+00	2.66E-04	down	-4.35E+00	7.55E-27	down
ENSMUSG00000019815	Zc2hc1b	-7.81E+00	7.72E-04	down	-4.82E+00	1.85E-01	no
ENSMUSG00000019808	Adat2	-2.76E+00	1.90E-06	down	-1.02E+00	3.13E-01	no
ENSMUSG00000019818	Cd164	-1.44E+00	2.83E-14	down	-2.25E+00	3.62E-33	down
ENSMUSG00000048696	Mex3d	-5.36E+00	2.73E-61	down	-3.17E+00	3.62E-08	down
ENSMUSG00000035278	Plekhj1	2.13E+00	2.23E-10	up	2.62E-01	4.63E-01	no
ENSMUSG00000047129	1700113H08Rik	-2.29E+00	1.32E-45	down	4.59E-01	2.01E-01	no
ENSMUSG00000062981	Mrpl42	1.09E+00	5.05E-08	up	2.45E-03	9.94E-01	no
ENSMUSG00000046567	4930430F08Rik	-3.59E+00	3.36E-10	down	-3.54E+00	6.42E-10	down
ENSMUSG00000020132	Rab21	-2.18E+00	1.65E-03	no	-2.74E+00	8.42E-07	down
ENSMUSG00000020171	Yeats4	-8.16E-01	4.94E-04	no	-1.58E+00	8.92E-21	down
ENSMUSG00000039824	Myl6b	-2.83E+00	7.79E-04	down	-3.79E+00	5.48E-07	down
ENSMUSG00000025354	Dnajc14	-2.71E+00	6.32E-04	down	-2.55E+00	1.52E-03	no
ENSMUSG00000019992	Mtfr2	-1.52E+00	7.83E-02	no	-3.14E+00	2.67E-06	down
ENSMUSG00000037440	Vnn1	-1.51E+00	3.95E-02	no	-2.17E+00	6.62E-04	down
ENSMUSG00000047692	4930533K18Rik	-2.71E+00	3.86E-04	down	-3.13E-01	8.95E-01	no
ENSMUSG00000053329	D10Jhu81e	-1.51E+00	3.63E-03	no	-2.63E+00	1.05E-11	down
ENSMUSG00000078442	Ccdc105	-2.32E+00	1.02E-46	down	-3.05E-02	9.47E-01	no
ENSMUSG00000063457	Rps15	1.26E+00	8.79E-47	up	6.76E-02	4.80E-01	no
ENSMUSG00000054452	Aes	1.78E+00	6.27E-04	up	-2.15E-01	8.29E-01	no
ENSMUSG00000052681	Rap1b	1.98E+00	1.44E-07	up	4.82E-01	4.71E-01	no
ENSMUSG00000075232	Amd1	-2.59E-01	8.25E-01	no	-2.46E+00	1.09E-06	down
ENSMUSG00000078451	Ppil6	-2.23E+00	1.86E-17	down	-4.87E-01	3.51E-01	no
ENSMUSG00000023068	Nus1	-1.60E+00	4.29E-02	no	-2.73E+00	1.17E-05	down
ENSMUSG00000009115	Spatc1l	-3.78E+00	2.68E-91	down	-9.20E-01	3.06E-03	no
ENSMUSG00000035890	Rnf126	-2.71E+00	3.19E-98	down	-2.52E+00	3.39E-104	down
ENSMUSG00000020163	Uqcr11	5.81E+00	0.00E+00	up	4.59E+00	0.00E+00	up
ENSMUSG00000054934	Kcnmb4	-1.20E+00	4.95E-02	no	-1.43E+00	4.50E-04	down
ENSMUSG00000064181	Rab3ip	-2.26E+00	7.65E-19	down	-2.03E+00	5.29E-16	down
ENSMUSG00000025417	Pip4k2c	-7.39E-01	5.11E-01	no	-2.58E+00	3.45E-04	down
ENSMUSG00000039531	Zufsp	-3.81E-01	7.03E-01	no	-3.05E+00	7.55E-15	down
ENSMUSG00000019917	43718	-2.64E+00	1.97E-34	down	-2.59E+00	3.60E-43	down
ENSMUSG00000009093	Gstt4	-1.86E+00	1.79E-97	down	4.13E-01	8.85E-03	no
ENSMUSG00000055862	Izumo4	-2.82E-01	6.72E-02	no	-1.04E+00	4.03E-23	down
ENSMUSG00000025359	Pmel	-2.35E+00	3.16E-04	down	-2.33E+00	2.20E-03	no
ENSMUSG00000019814	Ltv1	-1.34E+00	1.50E-05	down	-1.81E+00	4.55E-09	down
ENSMUSG00000019997	Ctgf	4.09E+00	3.02E-12	up	2.10E+00	2.09E-01	no
ENSMUSG00000020307	Cdc34	-2.12E+00	3.23E-60	down	-1.62E+00	1.72E-28	down
ENSMUSG00000004931	Apba3	-1.99E+00	1.65E-04	down	-1.99E+00	1.82E-04	down
ENSMUSG00000019948	Actr6	-5.10E-01	2.44E-01	no	-2.14E+00	1.74E-18	down
ENSMUSG00000036602	Alx1	-3.00E+00	8.58E-09	down	-2.65E+00	1.77E-11	down
ENSMUSG00000019906	Lin7a	-3.61E+00	6.42E-14	down	-3.34E+00	5.11E-15	down
ENSMUSG00000020214	Glipr1l2	-1.33E+00	1.94E-02	no	-2.76E+00	4.64E-17	down
ENSMUSG00000020102	Slc16a7	-6.07E+00	6.49E-137	down	-3.73E+00	3.61E-23	down

ENSMUSG00000019797	1700021F05Rik	-1.14E+00	8.99E-03	no	-1.42E+00	1.06E-05	down
ENSMUSG00000019878	Hsf2	-4.64E+00	6.89E-27	down	-2.48E+00	3.79E-04	down
ENSMUSG00000020190	Mknk2	-1.08E+00	2.38E-02	no	-2.42E+00	1.15E-09	down
ENSMUSG00000035529	Prdm4	-2.90E+00	1.26E-11	down	-1.51E+00	2.72E-02	no
ENSMUSG00000056912	1700017N19Rik	-4.25E+00	6.80E-103	down	-2.01E+00	1.30E-09	down
ENSMUSG00000038594	Cep85l	-3.95E+00	5.01E-68	down	-1.90E+00	3.28E-06	down
ENSMUSG00000019945	1700040L02Rik	-1.58E+00	5.63E-28	down	-1.68E+00	3.25E-59	down
ENSMUSG00000001663	Gstt1	4.56E+00	4.78E-04	up	3.08E+00	3.38E-01	no
ENSMUSG00000061032	Rrp1	-1.62E+00	3.46E-58	down	-1.63E+00	1.38E-87	down
ENSMUSG00000034854	Mfsd12	-1.85E+00	1.45E-05	down	-2.23E+00	2.57E-10	down
ENSMUSG00000020038	Cry1	-1.44E-01	8.47E-01	no	-2.16E+00	1.51E-09	down
ENSMUSG00000025410	Dctn2	-1.97E+00	4.09E-69	down	-1.77E+00	3.30E-70	down
ENSMUSG00000034994	Eef2	-2.64E+00	1.13E-268	down	-2.09E+00	8.81E-149	down
ENSMUSG00000025373	Rnf41	-2.50E+00	2.35E-08	down	-9.95E-01	2.28E-01	no
ENSMUSG00000039810	Zc3h10	-3.58E+00	9.58E-08	down	-2.93E+00	5.12E-06	down
ENSMUSG00000025358	Cdk2	-3.51E-01	5.85E-01	no	-2.89E+00	4.43E-28	down
ENSMUSG00000020075	Ddx21	-1.62E+00	1.21E-04	down	-1.87E+00	2.92E-05	down
ENSMUSG00000020076	Ddx50	-1.83E+00	6.72E-08	down	-1.78E+00	4.13E-05	down
ENSMUSG00000035206	Sppl2b	-2.08E+00	5.18E-11	down	-1.80E+00	1.32E-10	down
ENSMUSG00000020250	Txnrd1	-9.29E-01	1.13E-01	no	-2.61E+00	3.65E-06	down
ENSMUSG00000060904	Arl1	-1.31E+00	1.84E-05	down	-2.27E+00	7.84E-17	down
ENSMUSG00000019988	Nedd1	-2.06E+00	1.39E-04	down	-2.47E+00	6.80E-07	down
ENSMUSG00000020022	Ndufa12	1.05E+00	9.85E-15	up	7.48E-01	4.05E-09	no
ENSMUSG00000019936	Epyc	-3.24E+00	3.78E-07	down	-3.62E+00	2.25E-13	down
ENSMUSG00000034024	Cct2	-2.90E+00	3.79E-83	down	-2.70E+00	1.22E-61	down
ENSMUSG00000051236	Msrb3	-3.77E+00	9.94E-08	down	-4.37E+00	1.77E-19	down
ENSMUSG00000025351	Cd63	2.41E+00	2.12E-20	up	-4.53E-01	2.04E-01	no
ENSMUSG00000019791	Hint3	1.97E+00	2.59E-06	up	4.14E-01	1.26E-01	no
ENSMUSG00000019834	Slc22a16	-2.86E+00	1.71E-55	down	-2.61E+00	7.17E-59	down
ENSMUSG00000019861	Gopc	-7.81E-01	4.55E-01	no	-2.97E+00	1.98E-09	down
ENSMUSG00000019872	Smpdl3a	-2.06E+00	3.68E-04	down	-2.90E+00	5.15E-09	down
ENSMUSG00000038010	Ccdc138	-2.05E+00	1.92E-05	down	-1.86E+00	1.03E-03	no
ENSMUSG00000040021	Lats1	-3.36E+00	6.93E-04	down	-2.48E+00	6.29E-02	no
ENSMUSG00000003072	Atp5d	2.39E+00	2.37E-50	up	1.39E+00	1.98E-27	up
ENSMUSG00000001783	D10Wsu52e	5.56E-01	3.11E-01	no	-1.27E+00	1.16E-04	down
ENSMUSG00000020024	Ccdc41	-3.30E+00	3.52E-67	down	-2.35E+00	2.93E-21	down
ENSMUSG00000019929	Dcn	3.89E+00	9.40E-112	up	2.55E+00	1.56E-06	up
ENSMUSG00000020130	Tbc1d15	-2.10E+00	6.83E-18	down	-2.06E+00	5.11E-15	down
ENSMUSG00000061315	Naca	-2.26E+00	8.40E-05	down	-1.87E+00	9.13E-03	no
ENSMUSG00000090247	Bloc1s1	1.52E+00	4.82E-06	up	3.66E-01	2.92E-01	no
ENSMUSG00000038876	Rnf146	-2.79E+00	2.46E-07	down	-1.34E+00	1.67E-01	no
ENSMUSG00000020317	Theg	-6.73E+00	2.00E-112	down	-3.81E+00	4.54E-14	down
ENSMUSG00000035781	R3hdm4	3.19E+00	1.42E-04	up	9.68E-01	4.65E-01	no
ENSMUSG00000046822	Slc39a3	-1.78E+00	2.65E-03	no	-2.50E+00	2.32E-09	down
ENSMUSG00000020015	Cdk17	-2.26E+00	6.14E-03	no	-2.88E+00	1.82E-06	down
ENSMUSG00000071072	Ptges3	-1.26E+00	5.46E-11	down	-2.27E+00	1.60E-49	down
ENSMUSG00000071359	Tbpl1	-1.26E+00	2.41E-19	down	-1.44E+00	1.47E-32	down
ENSMUSG00000020089	Ppa1	2.83E+00	3.27E-05	up	1.16E-01	8.99E-01	no
ENSMUSG00000020078	Vps26a	-3.21E+00	5.76E-87	down	-3.23E+00	8.37E-88	down
ENSMUSG00000048701	Ccdc6	-3.73E+00	1.28E-06	down	-1.46E+00	4.39E-01	no
ENSMUSG00000020057	Dram1	-2.80E+00	4.08E-06	down	-5.61E-01	7.25E-01	no
ENSMUSG00000040034	Nup43	-2.00E+00	1.59E-04	down	-1.09E+00	1.09E-01	no
ENSMUSG00000019813	Cep57l1	-2.35E+00	8.82E-16	down	-1.74E+00	7.88E-07	down
ENSMUSG00000001665	Gstt3	-4.40E-01	5.68E-01	no	-1.42E+00	3.10E-04	down
ENSMUSG00000040006	Ginm1	-1.57E+00	3.62E-04	down	-1.37E+00	2.54E-02	no
ENSMUSG00000035351	Nup37	-1.05E+00	7.19E-03	no	-1.15E+00	8.67E-05	down
ENSMUSG00000044937	BC030307	-4.59E+00	8.41E-21	down	-3.61E+00	5.33E-10	down
ENSMUSG00000019951	Uhrf1bp1l	-2.77E+00	4.21E-10	down	-2.31E+00	1.19E-08	down
ENSMUSG00000020124	Usp15	-3.80E+00	1.16E-111	down	-2.34E+00	1.09E-28	down
ENSMUSG00000093674	Rpl41	3.48E+00	0.00E+00	up	2.75E+00	0.00E+00	up
ENSMUSG00000044475	Ascc1	-2.69E+00	5.95E-32	down	-1.62E+00	3.50E-10	down
ENSMUSG00000033255	Gm5134	-2.62E+00	5.21E-04	down	-3.69E+00	2.20E-17	down
ENSMUSG00000019977	Hbs1l	-3.09E+00	1.11E-17	down	-3.03E+00	8.19E-16	down
ENSMUSG00000003345	Csnk1g2	-4.05E+00	5.39E-115	down	-2.25E+00	3.65E-15	down

ENSMUSG00000020114	Cand1	-2.71E+00	1.47E-17	down	-2.65E+00	4.74E-18	down
ENSMUSG00000025357	Dgka	-3.93E+00	1.83E-51	down	-3.18E+00	9.03E-25	down
ENSMUSG00000061759	1700052N19Rik	-8.35E-01	1.28E-01	no	-2.21E+00	1.35E-12	down
ENSMUSG00000034667	Xpot	-1.72E+00	7.86E-02	no	-3.00E+00	1.50E-05	down
ENSMUSG00000025364	Pa2g4	-1.53E+00	1.98E-10	down	-1.61E+00	1.11E-14	down
ENSMUSG00000037608	Bclaf1	-6.16E-01	2.42E-01	no	-2.32E+00	1.24E-09	down
ENSMUSG00000038481	Cdk19	-2.57E+00	6.22E-05	down	-2.41E+00	2.99E-05	down
ENSMUSG00000020100	Slc29a3	-4.01E+00	2.36E-05	down	-2.37E+00	1.66E-01	no
ENSMUSG00000020074	Ccar1	-1.18E+00	1.65E-04	down	-2.13E+00	3.72E-14	down
ENSMUSG00000020064	Herc4	-4.10E+00	1.66E-28	down	-3.32E+00	8.65E-13	down
ENSMUSG00000020059	Sycp3	2.65E+00	1.98E-135	up	-4.00E-01	4.20E-06	no
ENSMUSG00000019907	Ppp1r12a	-1.84E-01	8.03E-01	no	-1.51E+00	8.50E-04	down
ENSMUSG00000005682	Pan2	-1.58E+00	2.63E-02	no	-2.74E+00	7.59E-08	down
ENSMUSG00000059554	Ccdc28a	-3.22E+00	1.19E-20	down	-1.35E+00	3.14E-02	no
ENSMUSG00000004667	Polr2e	1.60E+00	6.70E-06	up	3.60E-01	2.85E-01	no
ENSMUSG00000004929	Thop1	-2.39E+00	2.53E-37	down	-1.85E+00	2.83E-22	down
ENSMUSG00000048661	Lemd3	-1.77E+00	7.80E-02	no	-3.00E+00	1.04E-04	down
ENSMUSG00000025401	Myo1a	-2.35E+00	3.41E-03	no	-3.93E+00	1.80E-12	down
ENSMUSG00000020211	Sf3a2	-2.32E+00	9.90E-06	down	-2.70E+00	2.14E-09	down
ENSMUSG00000035365	Parpbp	-3.78E+00	2.39E-07	down	-3.63E+00	9.51E-06	down
ENSMUSG00000040415	Dtx3	-2.44E+00	1.45E-10	down	-3.12E+00	7.34E-24	down
ENSMUSG00000020003	Pex7	2.57E+00	6.53E-04	up	6.01E-02	9.47E-01	no
ENSMUSG00000003341	Atp8b3	-3.86E+00	0.00E+00	down	-2.68E+00	3.39E-167	down
ENSMUSG00000020021	Fgd6	-2.85E+00	6.19E-04	down	-3.47E+00	3.65E-06	down
ENSMUSG00000040462	Os9	-1.06E+00	9.88E-02	no	-2.33E+00	3.09E-06	down
ENSMUSG00000003226	Ranbp2	-2.81E+00	3.98E-06	down	-3.21E+00	2.77E-06	down
ENSMUSG00000058799	Nap1l1	-5.33E-01	4.49E-01	no	-2.69E+00	1.35E-13	down
ENSMUSG00000063334	Krr1	-2.05E+00	3.27E-02	no	-3.05E+00	3.20E-04	down
ENSMUSG00000058297	Spock2	-3.30E+00	1.31E-09	down	-4.25E+00	2.03E-16	down
ENSMUSG00000035504	Reep6	-4.84E+00	1.42E-119	down	-2.11E+00	4.94E-11	down
ENSMUSG00000020246	Hcfc2	-2.74E+00	2.20E-17	down	-2.53E+00	4.87E-15	down
ENSMUSG00000003923	Tfam	-3.84E+00	8.41E-18	down	-3.53E+00	4.81E-06	down
ENSMUSG00000045193	Cirbp	2.79E+00	2.32E-20	up	1.68E-01	5.97E-01	no
ENSMUSG00000020198	Ap3d1	-2.15E+00	1.01E-19	down	-1.82E+00	2.38E-10	down
ENSMUSG00000033444	Specc1l	-6.93E-01	3.97E-01	no	-2.01E+00	5.55E-05	down
ENSMUSG00000040195	Tmem194	-1.62E+00	4.96E-05	down	-2.41E+00	5.81E-14	down
ENSMUSG00000013858	Tmem259	-1.42E+00	3.55E-04	down	-2.15E+00	2.59E-11	down
ENSMUSG00000020153	Ndufs7	3.16E+00	1.85E-33	up	1.88E+00	5.24E-18	up
ENSMUSG00000035242	Oaz1	1.19E+00	7.28E-04	up	9.06E-01	8.79E-02	no
ENSMUSG00000019961	Tmpo	-1.58E+00	3.73E-02	no	-2.70E+00	8.44E-06	down
ENSMUSG00000075706	Gpx4	8.99E-01	1.58E-14	no	1.39E+00	9.33E-32	up
ENSMUSG00000035621	Midn	-4.46E+00	1.45E-06	down	-3.22E+00	7.60E-02	no
ENSMUSG00000062075	Lmnb2	-2.99E+00	7.88E-27	down	-3.83E-01	5.54E-01	no
ENSMUSG00000019996	Map7	-3.69E+00	4.25E-22	down	-3.00E+00	2.43E-10	down
ENSMUSG00000020079	Supv3l1	-2.08E+00	6.25E-04	down	-2.00E+00	5.90E-03	no
ENSMUSG00000019942	Cdk1	8.89E-01	1.17E-01	no	-1.22E+00	8.65E-05	down
ENSMUSG00000019564	Arid3a	-3.14E+00	3.89E-05	down	-7.81E-01	7.30E-01	no
ENSMUSG00000020069	Hnrnph3	-2.43E-01	5.45E-01	no	-1.89E+00	3.00E-16	down
ENSMUSG00000010461	Eya4	-5.34E+00	4.99E-08	down	-3.36E+00	1.02E-01	no
ENSMUSG00000036955	2510003E04Rik	-2.30E+00	1.09E-07	down	-7.22E-01	3.79E-01	no
ENSMUSG00000036168	Ccdc38	-3.80E+00	3.99E-87	down	-3.77E+00	1.62E-107	down
ENSMUSG00000019763	Rmnd1	-3.35E+00	6.69E-11	down	-5.93E-01	6.92E-01	no
ENSMUSG00000019978	Epb4.1l2	-3.77E+00	1.69E-49	down	-1.37E+00	4.76E-03	no
ENSMUSG00000091415	Akd1	-3.22E+00	2.15E-10	down	-3.28E+00	8.13E-14	down
ENSMUSG00000009291	Pttg1ip	-2.81E-01	4.00E-01	no	-1.12E+00	2.24E-04	down
ENSMUSG00000009070	Rtdr1	-2.07E+00	7.46E-04	down	-9.55E-01	1.94E-01	no
ENSMUSG00000020056	Ccdc53	-1.33E+00	1.49E-02	no	-2.10E+00	3.68E-08	down
ENSMUSG00000019892	Lrriq1	-2.28E+00	1.04E-06	down	-1.52E+00	4.33E-03	no
ENSMUSG00000038774	Ascc3	-2.71E+00	1.16E-05	down	-2.07E+00	9.44E-03	no
ENSMUSG00000037747	Phyhipl	-3.84E+00	2.75E-29	down	-1.96E+00	6.08E-04	down
ENSMUSG00000005897	Nr2c1	-2.99E+00	2.42E-04	down	-2.77E+00	1.41E-04	down
ENSMUSG00000020109	Dnajb12	-2.92E+00	3.24E-09	down	-1.99E+00	2.39E-03	no
ENSMUSG00000001785	Pwp1	-5.41E-01	4.89E-01	no	-1.86E+00	2.99E-05	down
ENSMUSG00000020048	Hsp90b1	-1.47E+00	1.25E-28	down	-2.78E+00	1.37E-132	down

ENSMUSG00000039480	Nt5dc1	-2.39E+00	5.01E-04	down	-2.65E+00	6.22E-06	down
ENSMUSG00000038510	Rpf2	-2.02E+00	1.58E-07	down	-1.18E+00	2.12E-02	no
ENSMUSG00000020189	Osbpl8	-5.15E-01	5.83E-01	no	-2.60E+00	4.63E-05	down
ENSMUSG00000020085	Aifm2	-1.80E+00	1.94E-02	no	-2.02E+00	2.94E-04	down
ENSMUSG00000020227	Irak3	-2.47E+00	3.37E-07	down	-2.88E+00	5.94E-12	down
ENSMUSG00000025374	Nabp2	-7.51E-01	3.29E-01	no	-1.81E+00	1.60E-04	down
ENSMUSG00000019952	Poc1b	-3.62E+00	3.61E-23	down	-2.42E+00	9.72E-07	down
ENSMUSG00000019876	Pkib	-2.53E+00	7.93E-06	down	-5.93E-01	6.79E-01	no
ENSMUSG00000035694	Caps2	-3.61E+00	1.81E-03	no	-3.33E+00	4.69E-04	down
ENSMUSG00000019971	Cep290	-2.85E+00	5.40E-23	down	-1.39E+00	6.18E-04	down
ENSMUSG00000025409	Mbd6	-2.57E+00	6.46E-06	down	-2.55E+00	1.58E-06	down
ENSMUSG00000000290	Itgb2	4.44E+00	1.38E-06	up	4.89E+00	3.79E-07	up
ENSMUSG00000013833	Med16	-2.53E-01	4.63E-01	no	-1.18E+00	2.14E-08	down
ENSMUSG00000020063	Sirt1	1.72E+00	2.00E-03	no	-2.34E+00	2.51E-05	down
ENSMUSG00000020235	Fzr1	-1.20E+00	9.05E-06	down	-8.32E-01	5.35E-03	no
ENSMUSG00000052798	Nup107	-1.08E+00	1.33E-01	no	-2.84E+00	1.44E-09	down
ENSMUSG00000020212	Mdm1	-3.24E+00	4.20E-28	down	-1.53E+00	7.05E-04	down
ENSMUSG00000020238	Ncln	-3.16E-01	5.79E-01	no	-1.34E+00	1.75E-04	down
ENSMUSG00000069565	Dazap1	-2.58E+00	3.53E-12	down	-3.38E+00	1.06E-41	down
ENSMUSG00000003068	Stk11	-2.80E+00	1.66E-15	down	-2.82E+00	4.87E-18	down
ENSMUSG00000020096	Tbata	-4.46E+00	1.20E-95	down	-1.62E+00	5.00E-05	down
ENSMUSG00000060843	Ctnna3	-4.67E+00	1.11E-06	down	-3.07E+00	2.46E-02	no
ENSMUSG00000040054	Baz2a	-3.26E+00	9.44E-23	down	-3.45E+00	1.29E-27	down
ENSMUSG00000019802	Sec63	-1.50E+00	1.86E-02	no	-2.69E+00	4.13E-07	down
ENSMUSG00000020131	Pcsk4	-3.18E+00	1.43E-31	down	-2.33E+00	2.24E-17	down
ENSMUSG00000060002	Chpt1	-2.09E+00	9.74E-04	down	-1.51E+00	4.20E-02	no
ENSMUSG00000069539	Scyl2	-3.42E+00	6.18E-03	no	-4.13E+00	1.08E-04	down
ENSMUSG00000020184	Mdm2	-1.38E+00	2.08E-07	down	-1.57E+00	2.93E-08	down
ENSMUSG00000034818	Celf5	-4.86E+00	9.75E-07	down	-3.11E+00	5.43E-02	no
ENSMUSG00000020037	Rfx4	-2.23E+00	1.24E-07	down	-1.83E+00	2.67E-06	down
ENSMUSG00000020156	Mum1	-9.22E-01	1.41E-04	no	-1.09E+00	5.85E-09	down
ENSMUSG00000019809	Pex3	-1.84E+00	3.94E-10	down	-4.02E-01	4.11E-01	no
ENSMUSG00000047417	Rexo1	-2.21E+00	4.14E-06	down	-7.46E-01	3.77E-01	no
ENSMUSG00000040354	Mars	2.05E+00	1.55E-08	up	2.12E+00	3.58E-16	up
ENSMUSG00000019856	Fam184a	-3.41E+00	2.58E-22	down	-2.23E+00	3.50E-08	down
ENSMUSG00000001211	Agpat3	-2.28E+00	3.91E-18	down	-1.81E+00	1.92E-12	down
ENSMUSG00000025369	Smarcc2	-4.05E+00	1.77E-24	down	-4.25E+00	1.73E-32	down
ENSMUSG00000071324	Armc2	-2.24E+00	2.43E-05	down	-1.23E+00	4.54E-02	no
ENSMUSG00000019944	Rhobtb1	-2.66E+00	8.67E-05	down	-2.61E+00	3.06E-06	down
ENSMUSG00000061589	Dot1l	-3.23E+00	1.67E-20	down	-2.78E+00	4.27E-14	down
ENSMUSG00000001120	Pcbp3	-1.59E+00	5.55E-25	down	-1.57E+00	1.66E-34	down
ENSMUSG00000006498	Ptbp1	-1.09E+00	8.84E-03	no	-1.79E+00	1.83E-06	down
ENSMUSG00000050994	Adgb	-5.10E+00	2.49E-21	down	-1.93E+00	5.88E-02	no
ENSMUSG00000019841	Rev3l	-3.38E+00	1.64E-05	down	-2.16E+00	8.39E-02	no
ENSMUSG00000020014	Gm872	-3.11E+00	8.09E-03	no	-3.49E+00	2.52E-04	down
ENSMUSG00000037012	Hk1	-4.67E+00	4.23E-133	down	-2.45E+00	2.56E-16	down
ENSMUSG00000020166	Cnot2	-2.10E+00	3.71E-07	down	-2.67E+00	1.36E-11	down
ENSMUSG00000025404	R3hdm2	-1.36E+00	9.74E-02	no	-2.63E+00	1.69E-06	down
ENSMUSG00000034813	Grip1	-4.61E+00	1.24E-18	down	-1.67E+00	1.06E-01	no
ENSMUSG00000032889	Gm6685	-3.60E+00	2.64E-06	down	-1.52E+00	2.62E-01	no
ENSMUSG00000078157	4931440F15Rik	-1.36E-01	7.16E-01	no	1.21E+00	6.12E-04	up
ENSMUSG00000078153	Psme2b-ps	3.91E+00	1.06E-10	up	2.75E+00	1.15E-06	up
ENSMUSG00000049427	Gm9837	-3.30E+00	1.22E-04	down	-1.26E+00	4.42E-01	no
ENSMUSG00000096083	Gm10428	-5.09E+00	1.13E-08	down	-2.69E+00	3.24E-04	down
ENSMUSG00000045176	2310047M10Rik	-4.07E-01	6.49E-01	no	-2.34E+00	4.42E-08	down
ENSMUSG00000050107	Gsg2	-3.15E+00	1.69E-180	down	-2.33E+00	1.90E-86	down
ENSMUSG00000046010	Zfp830	-1.56E+00	9.16E-05	down	-1.51E+00	3.38E-04	down
ENSMUSG00000034031	1700106J16Rik	-3.12E+00	8.28E-30	down	-6.31E-01	2.94E-01	no
ENSMUSG00000046755	Kif2b	-3.35E+00	3.16E-246	down	-7.57E-01	8.78E-05	no
ENSMUSG00000020870	Cdc34-ps	-3.33E+00	1.10E-05	down	-1.90E+00	2.74E-02	no
ENSMUSG00000078134	Gm12355	5.45E-01	3.86E-01	no	-1.89E+00	1.46E-08	down
ENSMUSG00000048878	Hexim1	-6.60E-01	7.08E-01	no	-3.40E+00	6.48E-05	down
ENSMUSG00000048732	Klhl11	-2.76E+00	4.66E-06	down	-1.93E+00	5.38E-02	no
ENSMUSG00000075420	Smim6	-2.15E+00	1.48E-10	down	-2.69E-01	7.47E-01	no

ENSMUSG00000043644	0610009L18Rik	1.64E+00	1.27E-27	up	1.59E+00	1.01E-74	up
ENSMUSG00000079010	Gm11032	1.01E+01	5.64E-116	up	9.97E+00	2.21E-109	up
ENSMUSG00000061650	Med9	9.56E-01	5.12E-06	no	1.07E+00	1.01E-08	up
ENSMUSG00000042200	Cdrt4	-2.90E+00	1.18E-132	down	-1.15E+00	3.33E-07	down
ENSMUSG00000018776	Slc35g3	-3.46E+00	1.41E-52	down	-6.77E-01	1.75E-01	no
ENSMUSG00000053783	1700016K19Rik	-1.10E+00	3.03E-34	down	-1.95E-01	7.55E-02	no
ENSMUSG00000072620	Slfn2	4.87E+00	2.41E-04	up	4.62E+00	1.39E-01	no
ENSMUSG00000090107	Gm11492	-5.13E+00	3.08E-35	down	-1.83E+00	5.11E-02	no
ENSMUSG00000037573	Tob1	-2.94E+00	1.37E-10	down	-3.22E+00	4.57E-09	down
ENSMUSG00000049506	Sppl2c	-4.26E+00	9.99E-61	down	-1.43E+00	2.30E-03	no
ENSMUSG00000034227	Foxj1	-6.48E+00	1.13E-42	down	-4.02E+00	1.19E-06	down
ENSMUSG00000078572	1810043H04Rik	3.06E+00	9.39E-84	up	2.16E+00	1.54E-64	up
ENSMUSG00000039337	Tex19.2	2.27E+00	1.37E-04	up	-5.47E-01	1.34E-01	no
ENSMUSG00000044949	Ubtd2	-3.49E+00	1.72E-35	down	-2.59E+00	6.25E-13	down
ENSMUSG00000020434	4921536K21Rik	-3.93E+00	1.63E-22	down	-4.32E+00	7.02E-29	down
ENSMUSG00000020475	Pgam2	-1.11E+00	1.36E-138	down	-6.97E-01	3.66E-84	no
ENSMUSG00000044084	4933402P03Rik	-4.42E+00	1.58E-92	down	-2.29E+00	1.43E-10	down
ENSMUSG00000041165	Spem1	-7.19E+00	1.58E-197	down	-6.15E+00	1.01E-48	down
ENSMUSG00000020830	Vmo1	8.02E+00	2.99E-19	up	6.65E+00	2.81E-31	up
ENSMUSG00000035373	Ccl7	4.96E+00	9.27E-13	up	7.03E+00	2.36E-03	no
ENSMUSG00000009185	Ccl8	5.40E+00	1.18E-42	up	5.10E+00	3.66E-07	up
ENSMUSG00000035085	1700020L24Rik	-1.08E+00	1.70E-11	down	1.29E+00	4.01E-06	up
ENSMUSG00000000982	Ccl3	4.26E+00	3.01E-17	up	4.97E+00	2.07E-03	no
ENSMUSG00000018930	Ccl4	6.39E+00	1.44E-36	up	6.14E+00	1.28E-07	up
ENSMUSG00000069792	Wfdc17	7.58E+00	1.62E-60	up	6.13E+00	1.61E-10	up
ENSMUSG00000069785	Gm11444	-3.43E+00	3.66E-05	down	-1.95E+00	2.71E-01	no
ENSMUSG00000047988	4933428G20Rik	-2.22E+00	3.89E-06	down	-1.80E+00	1.05E-03	no
ENSMUSG00000018543	1700001P01Rik	-9.68E-01	1.39E-16	no	1.38E+00	1.70E-12	up
ENSMUSG00000020810	Cygb	3.67E+00	2.41E-07	up	5.67E-01	7.84E-01	no
ENSMUSG00000070330	Tmem235	-4.10E+00	1.77E-01	no	-3.80E+00	1.58E-04	down
ENSMUSG00000076433	BC100451	-4.20E+00	3.56E-189	down	-2.10E+00	3.59E-20	down
ENSMUSG00000047205	Dusp18	-2.33E+00	1.63E-04	down	-2.19E+00	4.94E-05	down
ENSMUSG00000072983	4933414I15Rik	-3.25E+00	3.91E-17	down	-3.92E+00	7.81E-60	down
ENSMUSG00000007777	0610009B22Rik	-1.04E+00	2.77E-04	down	-1.39E+00	1.44E-10	down
ENSMUSG00000020383	Il13	2.47E+00	8.78E-03	no	2.28E+00	6.85E-04	up
ENSMUSG00000000594	Gm2a	-1.41E+00	8.52E-06	down	-1.50E+00	8.12E-07	down
ENSMUSG00000043648	Pld6	-2.70E-01	5.94E-01	no	-1.61E+00	1.26E-19	down
ENSMUSG00000019505	Ubb	5.02E+00	0.00E+00	up	4.29E+00	0.00E+00	up
ENSMUSG00000070388	Fbxo39	-5.50E+00	1.94E-27	down	-3.41E+00	3.94E-04	down
ENSMUSG00000047260	Emc6	2.24E+00	4.68E-26	up	1.06E+00	8.66E-11	up
ENSMUSG00000018844	Fndc8	-4.27E+00	8.15E-25	down	-2.19E+00	9.05E-03	no
ENSMUSG00000002580	Mien1	1.34E+00	9.43E-09	up	2.75E-01	1.43E-01	no
ENSMUSG00000038150	Ormdl3	-1.47E+00	2.79E-04	down	1.91E-01	8.36E-01	no
ENSMUSG00000044787	Spata32	-4.24E+00	2.29E-32	down	-2.59E+00	1.74E-05	down
ENSMUSG00000000125	Wnt3	-4.67E+00	2.20E-05	down	-3.93E+00	4.59E-02	no
ENSMUSG00000039329	Tex19.1	4.23E+00	1.37E-11	up	1.60E+00	8.11E-04	up
ENSMUSG00000069919	Hba-a1	8.94E+00	2.59E-27	up	8.44E+00	1.42E-08	up
ENSMUSG00000040489	Sox30	-3.80E+00	1.60E-49	down	-2.60E+00	3.89E-15	down
ENSMUSG00000020401	Fam71b	-4.89E+00	5.54E-56	down	-3.36E+00	1.56E-06	down
ENSMUSG00000050087	Cby3	-3.63E+00	4.12E-12	down	-2.03E+00	6.64E-02	no
ENSMUSG00000020267	Hint1	2.06E+00	6.21E-149	up	8.36E-01	6.55E-42	no
ENSMUSG00000020482	Ccdc117	-1.50E+00	5.34E-07	down	-2.72E+00	5.55E-35	down
ENSMUSG00000037001	Zfp39	-3.45E+00	5.88E-39	down	-2.69E+00	9.62E-18	down
ENSMUSG00000049291	Prss38	-1.06E+00	3.01E-01	no	-2.12E+00	9.31E-04	down
ENSMUSG00000014243	Zswim7	1.40E+00	3.73E-15	up	3.78E-01	4.07E-03	no
ENSMUSG00000035385	Ccl2	4.71E+00	4.45E-13	up	4.50E+00	4.79E-03	no
ENSMUSG00000034993	Vat1	-3.28E+00	1.15E-09	down	-3.54E+00	9.02E-10	down
ENSMUSG00000020733	Slc9a3r1	-1.71E+00	3.71E-06	down	4.29E-01	6.66E-01	no
ENSMUSG00000044034	Npb	-2.78E+00	7.54E-05	down	-6.87E-01	6.62E-01	no
ENSMUSG00000045942	BC049762	-2.12E+00	2.27E-88	down	6.98E-02	8.32E-01	no
ENSMUSG00000018931	Gm16515	-2.07E+00	1.83E-02	no	-2.07E+00	1.30E-04	down
ENSMUSG00000070345	Hsf5	-3.26E+00	1.18E-42	down	-2.87E+00	7.95E-49	down
ENSMUSG00000020553	Pctp	-1.35E+00	9.52E-02	no	-2.24E+00	2.42E-05	down
ENSMUSG00000000120	Ngfr	-5.54E+00	1.71E-06	down	-5.81E+00	1.45E-04	down

ENSMUSG00000001558	Klhl10	-3.65E+00	1.53E-159	down	-8.18E-01	1.10E-03	no
ENSMUSG00000020611	Gna13	-5.04E-01	6.14E-01	no	-2.54E+00	9.11E-05	down
ENSMUSG00000017716	Birc5	-2.92E-01	5.18E-01	no	-1.09E+00	8.82E-05	down
ENSMUSG00000039198	Ptchd3	-2.19E+00	3.57E-22	down	-2.18E+00	1.12E-29	down
ENSMUSG00000044502	Bod1	-2.98E-01	3.01E-01	no	-1.42E+00	6.96E-16	down
ENSMUSG00000044056	Efcab9	-8.12E-01	2.15E-19	no	1.32E+00	1.10E-17	up
ENSMUSG00000059534	Uqcr10	3.28E+00	0.00E+00	up	2.70E+00	0.00E+00	up
ENSMUSG00000046879	Irgm1	4.93E+00	3.98E-04	up	3.26E+00	1.81E-01	no
ENSMUSG00000001056	Nhp2	2.38E+00	1.45E-17	up	1.61E+00	2.35E-09	up
ENSMUSG00000018585	Atox1	2.87E+00	8.56E-68	up	1.85E+00	8.93E-56	up
ENSMUSG00000049154	Fam183b	-1.58E+00	2.18E-62	down	5.03E-01	4.53E-03	no
ENSMUSG00000020441	2310033P09Rik	2.24E+00	2.49E-04	up	7.97E-01	6.70E-02	no
ENSMUSG00000094845	Tmem95	-2.76E+00	2.80E-18	down	1.35E+00	1.69E-02	no
ENSMUSG00000040978	Gm11992	-2.32E+00	6.96E-09	down	-2.00E+00	5.45E-08	down
ENSMUSG00000014349	Ube2z	-4.26E-01	5.88E-01	no	-1.97E+00	7.06E-04	down
ENSMUSG00000020193	Zpbp	-1.77E+00	1.92E-22	down	-1.28E+00	3.24E-11	down
ENSMUSG00000041598	Cdc42ep4	-1.05E+00	2.49E-01	no	-2.84E+00	3.98E-14	down
ENSMUSG00000070332	4933422H20Rik	-3.82E+00	2.16E-63	down	-1.04E+00	1.60E-02	no
ENSMUSG00000046756	Mrps7	2.47E+00	1.62E-05	up	6.61E-01	2.06E-01	no
ENSMUSG00000020270	1700008A04Rik	-3.78E+00	3.74E-61	down	-2.74E+00	6.36E-13	down
ENSMUSG00000018239	Zcchc10	3.01E+00	1.08E-05	up	3.06E-01	6.28E-01	no
ENSMUSG00000042650	Alkbh5	-1.12E+00	1.29E-01	no	-2.47E+00	3.43E-05	down
ENSMUSG00000043284	Tmem11	2.92E+00	1.22E-08	up	1.27E+00	8.06E-03	no
ENSMUSG00000042189	Tekt3	-2.83E+00	1.76E-86	down	-1.56E+00	2.76E-24	down
ENSMUSG00000002064	Sdf2	-4.14E-01	5.74E-01	no	-2.05E+00	1.15E-04	down
ENSMUSG00000020878	Lrrc46	-1.01E+00	5.29E-80	down	-3.25E-01	1.96E-09	no
ENSMUSG00000006777	Krt23	5.48E+00	3.52E-16	up	5.43E+00	3.33E-12	up
ENSMUSG00000049800	Sertad2	-2.35E+00	3.40E-13	down	-1.59E+00	7.32E-05	down
ENSMUSG00000044894	Uqcrq	2.01E+00	3.62E-135	up	1.89E+00	8.48E-254	up
ENSMUSG00000041126	H2afv	3.19E+00	4.41E-27	up	1.28E+00	9.12E-07	up
ENSMUSG00000018293	Pfn1	3.41E+00	1.83E-52	up	7.73E-01	6.48E-06	no
ENSMUSG00000038352	Arl5c	7.08E+00	2.38E-29	up	5.62E+00	5.32E-08	up
ENSMUSG00000020930	Ccdc103	-2.25E+00	1.23E-11	down	-1.31E+00	6.44E-05	down
ENSMUSG00000020116	Pno1	-5.20E-01	3.45E-01	no	-1.29E+00	9.81E-04	down
ENSMUSG00000036275	9530068E07Rik	-5.41E-01	2.33E-01	no	-1.66E+00	5.26E-05	down
ENSMUSG00000020844	Nxn	-2.60E+00	6.05E-21	down	-2.89E+00	1.82E-50	down
ENSMUSG00000020525	Ppm1d	-3.38E+00	2.92E-113	down	-1.82E+00	2.51E-18	down
ENSMUSG00000061666	Gdpd1	-2.86E+00	6.16E-30	down	-1.95E+00	3.11E-11	down
ENSMUSG00000078632	Lrrc37a	-5.85E+00	7.84E-18	down	-2.65E+00	4.10E-02	no
ENSMUSG00000049354	Dcaf7	-1.91E+00	7.78E-11	down	-1.68E+00	6.91E-10	down
ENSMUSG00000040919	4930505A04Rik	-2.79E+00	1.32E-81	down	-6.35E-01	3.58E-02	no
ENSMUSG00000075702	Selm	3.37E+00	1.42E-07	up	2.77E+00	4.27E-02	no
ENSMUSG00000002741	Ykt6	-1.09E+00	4.63E-02	no	-2.61E+00	4.33E-09	down
ENSMUSG00000053184	Spaca3	-6.87E-01	1.31E-10	no	1.57E+00	3.68E-19	up
ENSMUSG00000018479	1700125H20Rik	-1.50E+00	4.52E-12	down	-8.45E-01	1.40E-03	no
ENSMUSG00000020869	Lrrc59	-1.77E+00	1.08E-11	down	-2.36E+00	1.35E-24	down
ENSMUSG00000018882	Mrpl45	1.29E+00	2.45E-02	no	-1.08E+00	6.02E-04	down
ENSMUSG00000069744	Psmb3	2.49E+00	5.41E-06	up	9.43E-01	2.54E-02	no
ENSMUSG00000020283	Pex13	-1.96E+00	2.32E-09	down	-2.24E+00	2.90E-15	down
ENSMUSG00000020335	Zfp354b	-6.00E+00	3.51E-05	down	-6.14E+00	1.11E-05	down
ENSMUSG00000018286	Psmb6	1.22E+00	7.18E-31	up	1.15E+00	3.95E-39	up
ENSMUSG00000040158	Tax1bp3	5.72E+00	2.41E-09	up	4.03E+00	1.82E-03	no
ENSMUSG00000020483	Dynll2	8.60E-02	5.62E-01	no	1.34E+00	9.89E-12	up
ENSMUSG00000025134	Alyref	2.66E+00	4.25E-05	up	-2.13E-02	9.79E-01	no
ENSMUSG00000013643	Lypd8	2.93E+00	1.68E-04	up	2.52E+00	1.59E-06	up
ENSMUSG00000004394	Tmed4	-8.29E-01	1.47E-02	no	-1.71E+00	2.73E-18	down
ENSMUSG00000000282	Mnt	-3.63E+00	4.25E-12	down	-2.60E+00	5.16E-05	down
ENSMUSG00000037857	Nufip2	-1.97E+00	7.84E-03	no	-3.18E+00	8.66E-08	down
ENSMUSG00000044122	Proca1	-4.36E+00	8.25E-91	down	-2.27E+00	1.22E-09	down
ENSMUSG00000000278	Scpep1	7.34E-01	2.06E-01	no	-1.59E+00	8.62E-05	down
ENSMUSG00000037601	Nme1	2.42E+00	1.72E-06	up	1.34E+00	3.20E-03	no
ENSMUSG00000038485	Socs7	-3.57E+00	9.81E-16	down	-3.21E+00	1.08E-14	down
ENSMUSG00000001751	Naglu	-2.52E+00	1.44E-05	down	-2.88E+00	1.05E-09	down
ENSMUSG00000020945	Lyzl6	-1.90E+00	1.57E-60	down	5.63E-01	1.05E-02	no

ENSMUSG00000087418	Ace3	-4.52E+00	7.53E-53	down	-1.18E+00	7.05E-02	no
ENSMUSG00000039275	Foxk2	-3.24E+00	8.08E-11	down	-2.96E+00	2.58E-10	down
ENSMUSG00000036964	Trim17	-3.84E+00	4.61E-46	down	-1.70E+00	3.39E-04	down
ENSMUSG00000018415	Gid4	-1.58E+00	6.09E-04	down	-1.92E+00	1.68E-04	down
ENSMUSG00000018567	Gabarap	-1.01E+00	9.37E-28	down	-1.11E+00	7.55E-40	down
ENSMUSG00000010392	Gosr1	-2.92E+00	2.17E-06	down	-2.80E+00	1.29E-07	down
ENSMUSG00000010841	1700006E09Rik	-4.41E+00	3.45E-140	down	-2.76E+00	9.05E-19	down
ENSMUSG00000043372	Hexim2	-2.31E+00	2.06E-06	down	-1.72E+00	8.93E-04	down
ENSMUSG00000020617	1700012B07Rik	-2.49E+00	1.74E-47	down	-1.40E-01	7.91E-01	no
ENSMUSG00000041623	D11Wsu47e	-4.22E+00	1.25E-132	down	-1.84E+00	8.61E-11	down
ENSMUSG00000020286	1700093K21Rik	-4.08E+00	4.34E-147	down	-1.82E+00	3.35E-10	down
ENSMUSG00000020520	Galnt10	-3.75E+00	1.58E-08	down	-4.13E+00	1.03E-11	down
ENSMUSG00000018347	Zkscan6	-2.98E+00	9.68E-08	down	-2.99E+00	8.98E-11	down
ENSMUSG00000032807	Alox12b	5.55E+00	4.36E-04	up	5.68E+00	3.29E-03	no
ENSMUSG00000087279	4930544D05Rik	-3.70E+00	4.05E-60	down	-2.35E+00	6.12E-11	down
ENSMUSG00000051232	Tmem199	2.12E+00	2.22E-05	up	1.77E-01	7.62E-01	no
ENSMUSG00000018925	Gm11435	-3.13E+00	2.18E-18	down	-2.51E-01	8.22E-01	no
ENSMUSG00000020935	Dcakd	2.11E+00	6.36E-04	up	8.69E-01	1.64E-01	no
ENSMUSG00000020946	Gosr2	-1.44E+00	2.16E-06	down	-1.93E+00	3.23E-13	down
ENSMUSG00000020712	Tcam1	-1.31E+00	1.14E-04	down	-2.60E+00	5.38E-47	down
ENSMUSG00000020191	4930415F15Rik	-4.02E+00	1.39E-54	down	-1.56E+00	1.02E-03	no
ENSMUSG00000016559	H3f3b	3.22E+00	1.73E-233	up	8.31E-01	2.09E-26	no
ENSMUSG00000034120	Srsf2	8.84E-01	1.49E-02	no	-1.46E+00	3.27E-07	down
ENSMUSG00000040365	Trim41	-2.97E+00	1.96E-17	down	-3.15E+00	2.02E-18	down
ENSMUSG00000045915	Ccdc42	-4.15E+00	8.01E-87	down	-3.37E+00	1.88E-72	down
ENSMUSG00000070394	Tmem256	3.69E+00	2.69E-05	up	2.30E+00	2.60E-02	no
ENSMUSG00000020521	Rnft1	-2.94E+00	7.63E-09	down	-2.96E+00	1.11E-08	down
ENSMUSG00000020485	Supt4a	1.54E+00	1.32E-04	up	-3.11E-01	4.49E-01	no
ENSMUSG00000024414	Mrpl27	4.49E+00	1.12E-19	up	1.15E+00	1.06E-03	no
ENSMUSG00000025130	P4hb	1.27E+00	4.03E-04	up	-1.15E+00	1.42E-03	no
ENSMUSG00000018339	Gpx3	4.13E+00	1.17E-08	up	3.15E+00	2.72E-02	no
ENSMUSG00000020477	Mrps24	4.80E+00	3.23E-18	up	2.52E+00	1.74E-08	up
ENSMUSG00000032921	Odf4	-1.17E+00	6.13E-31	down	-6.50E-01	6.47E-14	no
ENSMUSG00000018774	Cd68	3.83E+00	9.39E-09	up	2.13E+00	6.27E-02	no
ENSMUSG00000019173	Rab5c	-3.12E+00	1.71E-12	down	-2.71E+00	5.10E-09	down
ENSMUSG00000051455	Gm1564	-1.67E+00	8.82E-06	down	-3.61E+00	7.60E-45	down
ENSMUSG00000020736	Nt5c	-2.05E+00	1.59E-06	down	-2.24E+00	9.24E-09	down
ENSMUSG00000041278	Ttc1	-1.21E+00	2.83E-05	down	-1.03E+00	1.06E-04	down
ENSMUSG00000020390	Ube2b	-7.45E-01	1.16E-04	no	-1.20E+00	6.97E-12	down
ENSMUSG00000020455	Trim11	-1.00E+00	7.05E-02	no	-2.40E+00	9.70E-18	down
ENSMUSG00000018924	Alox15	3.81E+00	2.20E-06	up	4.70E+00	1.42E-06	up
ENSMUSG00000078653	Cntd1	-2.01E+00	2.85E-03	no	-2.59E+00	1.56E-08	down
ENSMUSG00000051378	Kif18b	-2.76E+00	1.01E-09	down	-1.99E+00	2.79E-04	down
ENSMUSG00000057322	Rpl38	2.09E+00	6.43E-23	up	1.36E+00	7.72E-15	up
ENSMUSG00000033880	Lgals3bp	3.17E+00	6.24E-05	up	2.09E+00	2.07E-01	no
ENSMUSG00000060938	Rpl26	2.20E+00	3.49E-20	up	1.55E+00	4.05E-13	up
ENSMUSG00000069825	Spata22	-7.46E-02	9.57E-01	no	-2.72E+00	2.32E-04	down
ENSMUSG00000040985	Sun3	-4.54E+00	1.13E-67	down	-2.91E+00	7.88E-18	down
ENSMUSG00000020864	Ankrd40	-2.16E+00	2.67E-05	down	-2.66E+00	9.55E-09	down
ENSMUSG00000006057	Atp5g1	3.38E+00	8.19E-10	up	2.54E+00	1.74E-04	up
ENSMUSG00000020705	Ddx42	-7.81E-01	1.52E-01	no	-2.30E+00	1.03E-08	down
ENSMUSG00000000049	Apoh	-2.99E+00	1.46E-20	down	-6.26E-01	3.55E-01	no
ENSMUSG00000057286	St6galnac2	-5.55E+00	3.50E-108	down	-2.60E+00	2.26E-10	down
ENSMUSG00000035455	Fignl1	-5.37E-01	5.46E-01	no	-2.81E+00	4.14E-11	down
ENSMUSG00000051355	Commd1	-7.01E-01	2.66E-01	no	-1.68E+00	2.25E-06	down
ENSMUSG00000020460	Rps27a	-7.04E-02	9.44E-01	no	-2.07E+00	7.86E-05	down
ENSMUSG00000020461	Clhc1	-3.48E+00	2.09E-15	down	-3.31E+00	5.62E-25	down
ENSMUSG00000020376	Rnf130	2.50E+00	5.33E-05	up	2.88E-01	8.22E-01	no
ENSMUSG00000063652	Slc22a21	-1.54E+00	2.62E-06	down	-2.11E+00	3.19E-18	down
ENSMUSG00000049755	Zfp672	-1.10E+00	1.98E-02	no	-2.11E+00	4.84E-09	down
ENSMUSG00000036860	Mrpl55	1.04E+00	2.82E-07	up	4.20E-01	2.33E-02	no
ENSMUSG00000032615	Nt5m	-1.69E+00	2.90E-04	down	-1.48E+00	6.28E-06	down
ENSMUSG00000020805	Slc13a5	-3.71E+00	3.25E-07	down	-4.01E+00	3.29E-20	down
ENSMUSG00000020677	Ddx52	-1.01E+00	1.28E-01	no	-2.55E+00	3.63E-09	down

ENSMUSG00000020922	Lsm12	-3.04E+00	1.31E-10	down	-3.28E+00	3.78E-13	down
ENSMUSG00000020936	Nmt1	-1.39E+00	1.26E-06	down	-7.84E-01	6.10E-03	no
ENSMUSG00000078970	Wdr92	-9.01E-01	3.86E-01	no	-2.25E+00	1.60E-06	down
ENSMUSG00000049811	Fam161a	-3.43E+00	4.10E-24	down	-1.60E+00	2.58E-04	down
ENSMUSG00000040767	Snrnp25	1.85E+00	1.32E-10	up	2.76E-01	1.89E-01	no
ENSMUSG00000020448	Rnf185	-2.20E+00	1.43E-05	down	-1.64E+00	2.15E-03	no
ENSMUSG00000042436	Mfap4	4.85E+00	9.20E-07	up	3.58E+00	2.42E-01	no
ENSMUSG00000018921	Pelp1	-3.61E+00	5.26E-14	down	-3.53E+00	3.18E-14	down
ENSMUSG00000040548	Tex2	-4.93E+00	1.77E-95	down	-3.80E+00	2.93E-46	down
ENSMUSG00000048277	Syngr2	4.02E+00	3.42E-06	up	1.74E+00	9.79E-02	no
ENSMUSG00000020467	Efemp1	2.90E+00	9.19E-04	up	1.82E+00	5.10E-01	no
ENSMUSG00000018217	Pmp22	4.04E+00	4.29E-08	up	3.04E+00	9.71E-02	no
ENSMUSG00000020526	Znhit3	-7.03E-01	2.87E-02	no	-1.38E+00	1.14E-12	down
ENSMUSG00000018666	Cbx1	1.90E+00	7.79E-04	up	-6.65E-01	9.16E-02	no
ENSMUSG00000003518	Dusp3	-2.16E+00	2.32E-05	down	-8.32E-01	5.12E-01	no
ENSMUSG00000025150	Cbr2	5.57E+00	7.56E-26	up	5.30E+00	9.05E-05	up
ENSMUSG00000020272	Stk10	-1.93E+00	1.06E-05	down	-1.85E+00	2.27E-05	down
ENSMUSG00000020397	Med7	-1.27E+00	7.58E-03	no	-1.91E+00	4.07E-07	down
ENSMUSG00000020849	Ywhae	-1.94E+00	2.32E-39	down	-2.08E+00	1.50E-49	down
ENSMUSG00000017837	Nkiras2	-1.84E+00	1.99E-11	down	-1.72E+00	5.96E-15	down
ENSMUSG00000020799	Tekt1	-2.01E+00	8.42E-126	down	-9.53E-01	4.22E-30	no
ENSMUSG00000018733	Pex12	-2.43E+00	5.99E-07	down	-1.43E+00	2.90E-02	no
ENSMUSG00000018379	Srsf1	-7.20E-01	2.03E-01	no	-2.77E+00	9.09E-17	down
ENSMUSG00000033983	Coil	-4.28E+00	9.06E-43	down	-3.26E+00	7.09E-31	down
ENSMUSG00000011254	Thg1l	-2.48E+00	8.00E-05	down	-2.74E+00	7.92E-07	down
ENSMUSG00000001054	Rmnd5b	-1.23E+00	9.00E-05	down	-8.55E-01	9.60E-03	no
ENSMUSG00000018559	Ctdnep1	-2.25E+00	1.28E-07	down	-2.68E+00	1.09E-10	down
ENSMUSG00000038976	Ppp1r9b	-3.05E+00	2.15E-03	no	-4.59E+00	2.71E-11	down
ENSMUSG00000038967	Pdk2	-2.56E+00	5.22E-30	down	-1.84E+00	1.62E-12	down
ENSMUSG00000097487	Ptges3l	-5.95E-01	4.62E-01	no	-1.70E+00	7.14E-06	down
ENSMUSG00000001891	Ugp2	-2.23E+00	8.43E-05	down	-2.69E+00	7.06E-06	down
ENSMUSG00000049491	Slc36a3	-3.99E+00	1.67E-10	down	-1.14E+00	4.51E-01	no
ENSMUSG00000001105	Ift20	1.70E+00	1.81E-09	up	-3.87E-01	4.78E-02	no
ENSMUSG00000020462	Ccdc104	-2.15E+00	4.34E-100	down	-2.08E+00	1.80E-104	down
ENSMUSG00000020332	4930404A10Rik	-1.33E+00	1.95E-02	no	-1.63E+00	1.05E-05	down
ENSMUSG00000020471	Pold2	-1.05E+00	2.09E-02	no	-1.28E+00	4.16E-04	down
ENSMUSG00000020798	Spns3	4.95E+00	3.84E-04	up	4.41E+00	3.20E-04	up
ENSMUSG00000034239	Gm884	-5.26E+00	1.98E-15	down	-3.84E+00	6.50E-08	down
ENSMUSG00000020300	Cpeb4	-3.87E+00	1.80E-07	down	-3.64E+00	2.46E-05	down
ENSMUSG00000002129	Sf3a1	-1.23E+00	4.24E-02	no	-2.28E+00	3.63E-06	down
ENSMUSG00000020372	Gnb2l1	1.11E+00	1.44E-02	no	-1.54E+00	1.05E-07	down
ENSMUSG00000020268	Lyrm7	-1.89E+00	2.38E-06	down	-1.02E-01	9.23E-01	no
ENSMUSG00000020698	Cct6b	-2.34E+00	5.50E-53	down	-2.25E+00	3.08E-70	down
ENSMUSG00000001440	Kpnb1	-2.91E+00	3.54E-14	down	-4.15E+00	1.27E-46	down
ENSMUSG00000020755	Sap30bp	-1.74E+00	2.50E-04	down	-8.75E-01	1.68E-01	no
ENSMUSG00000020775	Mrpl38	-1.18E+00	1.30E-03	no	-1.86E+00	2.15E-13	down
ENSMUSG00000025575	Cant1	-1.85E+00	6.28E-04	down	-2.06E+00	1.80E-05	down
ENSMUSG00000020904	Wdr16	-3.06E+00	1.13E-37	down	-2.44E+00	1.01E-30	down
ENSMUSG00000054079	Utp18	-1.90E+00	1.46E-07	down	-2.62E+00	7.34E-16	down
ENSMUSG00000006058	Snf8	-1.25E+00	2.53E-02	no	-1.83E+00	8.57E-05	down
ENSMUSG00000038756	Ttll6	-3.55E+00	1.55E-34	down	-2.61E+00	1.41E-19	down
ENSMUSG00000018678	Sp2	-9.20E-01	4.95E-01	no	-3.70E+00	8.66E-08	down
ENSMUSG00000078622	Ccdc47	1.68E-01	8.97E-01	no	-2.25E+00	8.41E-04	down
ENSMUSG00000020903	Stx8	-1.81E+00	3.28E-03	no	-1.92E+00	4.39E-05	down
ENSMUSG00000018765	Fxr2	-1.52E+00	9.76E-05	down	-2.53E+00	3.09E-14	down
ENSMUSG00000020802	Ube2o	-2.52E+00	4.36E-07	down	-2.41E+00	1.04E-06	down
ENSMUSG00000020457	Drg1	-9.02E-01	4.28E-04	no	-1.14E+00	4.37E-08	down
ENSMUSG00000020537	Drg2	-1.33E+00	1.30E-02	no	-1.52E+00	2.17E-05	down
ENSMUSG00000034793	G6pc3	-2.34E+00	5.17E-24	down	-2.13E+00	1.08E-25	down
ENSMUSG00000034773	BC030867	-1.59E+00	1.79E-02	no	-2.46E+00	1.50E-05	down
ENSMUSG00000048445	Ccdc57	-2.83E+00	3.84E-05	down	-1.84E+00	1.33E-01	no
ENSMUSG00000006169	Clint1	-2.08E+00	3.31E-04	down	-1.59E+00	3.57E-02	no
ENSMUSG00000036309	Skp1a	-6.13E-01	2.90E-05	no	-1.61E+00	4.53E-35	down
ENSMUSG00000035992	Fnip1	-2.03E+00	4.90E-04	down	-2.75E+00	5.61E-08	down

ENSMUSG00000025161	Slc16a3	-2.53E+00	3.03E-04	down	-2.39E+00	1.07E-03	no
ENSMUSG00000020311	Erlec1	-2.80E+00	3.37E-12	down	-3.37E+00	1.30E-17	down
ENSMUSG00000015837	Sqstm1	2.15E+00	2.49E-15	up	-8.02E-03	9.89E-01	no
ENSMUSG00000020897	Aurkb	-3.59E-01	7.62E-01	no	-2.38E+00	5.19E-05	down
ENSMUSG00000018554	Ybx2	-3.31E+00	0.00E+00	down	-3.03E+00	0.00E+00	down
ENSMUSG00000001100	Poldip2	-1.25E+00	2.06E-06	down	-1.40E+00	8.49E-10	down
ENSMUSG00000017221	Psmd3	-3.74E-01	4.98E-01	no	-1.42E+00	6.69E-05	down
ENSMUSG00000020614	Fam20a	1.41E+00	3.85E-08	up	2.73E+00	7.75E-42	up
ENSMUSG00000018727	Cpsf4l	-3.51E+00	1.15E-16	down	-6.98E-01	4.98E-01	no
ENSMUSG00000018697	Aatf	-1.74E+00	2.93E-06	down	-1.78E+00	2.22E-07	down
ENSMUSG00000041895	Wipi1	-2.34E+00	4.70E-04	down	-1.54E+00	1.72E-01	no
ENSMUSG00000020472	Zkscan17	-4.16E+00	7.93E-33	down	-3.58E+00	4.02E-26	down
ENSMUSG00000069855	Slc47a2	-1.27E+00	1.09E-01	no	-3.13E+00	1.56E-21	down
ENSMUSG00000047342	Zfp286	-2.52E+00	3.95E-02	no	-3.33E+00	4.12E-05	down
ENSMUSG00000018425	Dhx40	-2.76E+00	2.79E-20	down	-3.14E+00	4.26E-25	down
ENSMUSG00000020690	Efcab3	-4.67E+00	2.55E-87	down	-3.08E+00	2.33E-19	down
ENSMUSG00000078627	43534	-3.08E+00	1.45E-100	down	-2.13E+00	3.31E-43	down
ENSMUSG00000070331	Qrich2	-4.71E+00	1.10E-172	down	-3.66E+00	2.98E-121	down
ENSMUSG00000020305	Asb3	-3.07E-01	8.37E-01	no	-2.52E+00	3.11E-04	down
ENSMUSG00000020523	Fam114a2	6.70E-01	4.51E-01	no	-1.63E+00	2.54E-04	down
ENSMUSG00000057054	Inca1	1.40E+00	6.48E-04	up	-7.31E-01	1.11E-01	no
ENSMUSG00000001123	Lgals9	3.97E+00	3.82E-04	up	3.72E+00	1.22E-01	no
ENSMUSG00000052915	Msl1	-3.15E+00	2.73E-35	down	-2.94E+00	1.01E-33	down
ENSMUSG00000006784	Ttc25	-3.08E+00	5.06E-31	down	-2.21E+00	3.41E-16	down
ENSMUSG00000020940	1700023F06Rik	-1.71E+00	7.18E-26	down	9.34E-01	9.16E-04	no
ENSMUSG00000020706	Ftsj3	-5.02E-01	4.12E-01	no	-1.70E+00	6.38E-05	down
ENSMUSG00000056962	Jmjd6	-2.23E+00	5.59E-07	down	-2.19E+00	1.04E-06	down
ENSMUSG00000000753	Serpinf1	3.85E+00	2.36E-09	up	2.80E+00	1.11E-01	no
ENSMUSG00000057522	Spop	1.01E-01	8.85E-01	no	-2.06E+00	4.10E-04	down
ENSMUSG00000078676	Casc3	-7.23E-01	3.46E-01	no	-2.87E+00	4.09E-12	down
ENSMUSG00000062825	Actg1	1.53E+00	4.58E-23	up	-3.43E-01	8.10E-03	no
ENSMUSG00000034614	Pik3ip1	-1.57E+00	5.22E-06	down	-1.48E+00	5.23E-09	down
ENSMUSG00000020362	Cnot6	-1.89E+00	2.37E-03	no	-2.27E+00	2.20E-04	down
ENSMUSG00000018593	Sparc	5.85E+00	5.24E-202	up	2.95E+00	7.98E-39	up
ENSMUSG00000017417	Plxdc1	-2.72E+00	9.23E-12	down	-2.09E+00	1.35E-06	down
ENSMUSG00000078652	Psme3	-1.97E+00	5.89E-10	down	-1.90E+00	6.86E-08	down
ENSMUSG00000020412	Ascc2	-3.04E+00	2.32E-16	down	-2.95E+00	2.43E-17	down
ENSMUSG00000020368	Canx	-1.25E+00	2.03E-05	down	-2.11E+00	4.18E-16	down
ENSMUSG00000020361	Hspa4	-3.10E+00	7.49E-36	down	-3.38E+00	3.88E-49	down
ENSMUSG00000020476	Dbnl	2.89E-02	9.65E-01	no	-1.54E+00	2.62E-05	down
ENSMUSG00000020528	Prpsap2	1.58E-01	8.94E-01	no	-2.52E+00	2.61E-05	down
ENSMUSG00000020905	Usp43	5.34E+00	4.14E-11	up	4.81E+00	3.05E-13	up
ENSMUSG00000023170	Gps2	3.67E-01	8.84E-03	no	1.19E+00	8.69E-30	up
ENSMUSG00000018572	Phf23	-6.33E-01	3.49E-01	no	-2.33E+00	1.45E-08	down
ENSMUSG00000025792	Slc25a10	-2.05E+00	1.38E-03	no	-2.59E+00	2.06E-09	down
ENSMUSG00000047804	Akap10	-3.64E+00	4.31E-12	down	-2.50E+00	1.20E-03	no
ENSMUSG00000017195	Zpbp2	-1.64E+00	1.64E-66	down	-1.45E+00	4.59E-64	down
ENSMUSG00000020610	Amz2	-1.65E+00	4.01E-18	down	-1.35E+00	8.62E-15	down
ENSMUSG00000037331	Larp1	-2.44E+00	9.62E-32	down	-2.20E+00	2.91E-34	down
ENSMUSG00000018442	Derl2	-1.93E+00	9.23E-03	no	-2.29E+00	3.33E-04	down
ENSMUSG00000006930	Hap1	-4.45E+00	7.62E-10	down	-4.23E+00	6.11E-12	down
ENSMUSG00000015542	Nat9	-1.23E+00	3.14E-04	down	-1.30E+00	1.80E-07	down
ENSMUSG00000025162	Csnk1d	-2.09E+00	1.99E-17	down	-1.76E+00	4.41E-10	down
ENSMUSG00000018736	Ndel1	-1.21E+00	5.42E-04	down	-9.40E-01	8.29E-02	no
ENSMUSG00000020513	Tubd1	-2.79E+00	1.07E-08	down	-2.88E+00	4.20E-16	down
ENSMUSG00000017802	Fam134c	-3.09E+00	4.50E-07	down	-1.94E+00	7.15E-02	no
ENSMUSG00000020319	Wdpcp	-2.74E+00	1.03E-05	down	-2.32E+00	5.35E-06	down
ENSMUSG00000020430	Pes1	-1.23E+00	7.90E-03	no	-2.32E+00	6.29E-12	down
ENSMUSG00000051427	Ccdc157	-2.24E+00	4.20E-06	down	-2.24E+00	1.62E-05	down
ENSMUSG00000046311	Zfp62	-2.99E+00	1.62E-05	down	-3.17E+00	3.50E-07	down
ENSMUSG00000018548	Trim37	-3.49E+00	2.60E-77	down	-3.31E+00	1.29E-94	down
ENSMUSG00000001552	Jup	-3.63E+00	5.68E-06	down	-1.15E+00	5.81E-01	no
ENSMUSG00000039703	Nploc4	-2.58E+00	7.96E-13	down	-3.20E+00	1.44E-32	down
ENSMUSG00000020696	Rffl	-2.99E+00	1.79E-16	down	-9.40E-01	1.63E-01	no

ENSMUSG00000020680	Taf15	-3.79E+00	6.89E-15	down	-5.05E+00	5.41E-25	down
ENSMUSG00000020867	Spata20	-3.88E+00	1.12E-291	down	-1.17E+00	5.86E-11	down
ENSMUSG00000069631	Strada	-1.88E+00	8.51E-07	down	-1.82E+00	2.39E-08	down
ENSMUSG00000057113	Npm1	-6.50E-01	2.04E-02	no	-1.88E+00	5.91E-16	down
ENSMUSG00000020435	Osbp2	-3.82E+00	4.48E-67	down	-1.90E+00	9.36E-10	down
ENSMUSG00000020415	Pttg1	7.20E-01	6.47E-03	no	-1.43E+00	9.11E-23	down
ENSMUSG00000020745	Pafah1b1	-3.57E+00	3.10E-26	down	-3.46E+00	3.65E-23	down
ENSMUSG00000034708	Grn	4.82E+00	3.83E-17	up	4.42E+00	3.49E-07	up
ENSMUSG00000038290	Smg6	-2.50E+00	7.23E-08	down	-2.02E+00	1.78E-05	down
ENSMUSG00000050944	Efcab5	-3.98E+00	1.54E-42	down	-3.96E+00	8.98E-63	down
ENSMUSG00000020780	Srp68	-2.11E+00	5.02E-09	down	-2.78E+00	9.56E-24	down
ENSMUSG00000019373	Cops3	-1.48E+00	6.98E-09	down	-2.07E+00	4.55E-24	down
ENSMUSG00000005204	Senp3	-1.91E+00	8.48E-04	down	-3.12E+00	3.18E-12	down
ENSMUSG00000006127	Inpp5k	-2.13E+00	2.55E-09	down	-1.73E+00	5.09E-07	down
ENSMUSG00000061086	Myl4	-2.16E+00	1.84E-04	down	-1.57E+00	1.18E-02	no
ENSMUSG00000020288	Ahsa2	-1.48E+00	3.79E-01	no	-3.45E+00	4.05E-04	down
ENSMUSG00000020463	Smek2	-1.77E+00	2.72E-04	down	-3.29E+00	2.28E-18	down
ENSMUSG00000032633	Flcn	1.00E-01	9.34E-01	no	-2.39E+00	2.97E-05	down
ENSMUSG00000056598	Lrrc48	-2.44E+00	2.27E-11	down	-2.30E+00	8.12E-17	down
ENSMUSG00000040746	Rnf167	-7.10E-01	5.88E-02	no	-1.33E+00	1.59E-05	down
ENSMUSG00000010342	Tex14	-2.14E+00	7.51E-06	down	-2.90E+00	1.27E-09	down
ENSMUSG00000018661	Cog1	-3.05E+00	4.37E-10	down	-2.74E+00	1.59E-08	down
ENSMUSG00000037926	Ssh2	-2.71E+00	1.00E-06	down	-3.11E+00	2.32E-14	down
ENSMUSG00000017548	Suz12	-1.48E+00	8.44E-02	no	-3.25E+00	9.04E-06	down
ENSMUSG00000020744	Slc25a19	-1.23E+00	5.76E-02	no	-1.92E+00	3.04E-06	down
ENSMUSG00000034931	Dhx8	-2.59E+00	1.09E-08	down	-2.29E+00	3.01E-05	down
ENSMUSG00000018372	Cep95	-3.75E+00	3.07E-16	down	-4.06E+00	1.82E-21	down
ENSMUSG00000018565	Elp5	-2.62E-01	2.26E-01	no	-1.21E+00	3.12E-25	down
ENSMUSG00000017781	Pitpna	-5.03E-01	2.34E-01	no	-1.24E+00	1.59E-04	down
ENSMUSG00000049659	Aftph	-2.89E+00	5.24E-07	down	-3.30E+00	7.09E-10	down
ENSMUSG00000020719	Ddx5	-1.28E+00	1.13E-22	down	-1.62E+00	3.57E-32	down
ENSMUSG00000007739	Cct4	-2.28E+00	8.42E-41	down	-2.60E+00	6.44E-72	down
ENSMUSG00000004798	Ulk2	-3.11E+00	1.07E-20	down	-2.79E+00	2.17E-15	down
ENSMUSG00000040829	Zmynd15	-3.86E+00	2.53E-33	down	-3.02E+00	6.69E-16	down
ENSMUSG00000039110	Mycbpap	-4.23E+00	5.53E-80	down	-2.90E+00	1.15E-23	down
ENSMUSG00000020687	Cdc27	-4.22E+00	9.22E-27	down	-3.53E+00	2.10E-18	down
ENSMUSG00000020459	Mtif2	-2.39E-02	9.79E-01	no	-1.83E+00	4.79E-04	down
ENSMUSG00000002052	Supt6	-1.27E+00	1.78E-02	no	-2.05E+00	1.50E-05	down
ENSMUSG00000018428	Akap1	-5.40E+00	4.42E-95	down	-2.99E+00	7.01E-10	down
ENSMUSG00000020914	Top2a	-5.90E-01	1.79E-01	no	-2.08E+00	1.40E-09	down
ENSMUSG00000078619	Smarcd2	-1.34E+00	9.04E-03	no	-2.50E+00	2.39E-10	down
ENSMUSG00000090173	Fbxw10	-3.93E+00	9.34E-64	down	-3.23E+00	4.23E-37	down
ENSMUSG00000005947	Itgae	-1.36E+00	3.13E-04	down	-1.69E+00	4.67E-08	down
ENSMUSG00000017405	Nek8	-1.97E+00	6.34E-02	no	-2.78E+00	4.84E-04	down
ENSMUSG00000010086	Rnf112	-4.81E+00	2.11E-03	no	-5.88E+00	1.57E-11	down
ENSMUSG00000060600	Eno3	-1.26E+00	7.83E-04	down	-2.92E+00	3.57E-44	down
ENSMUSG00000034940	Synrg	-3.51E+00	2.97E-08	down	-3.36E+00	1.05E-08	down
ENSMUSG00000020409	Slu7	-1.45E+00	3.02E-03	no	-2.15E+00	1.35E-07	down
ENSMUSG00000000384	Tbrg4	-9.24E-01	8.91E-02	no	-1.76E+00	2.26E-05	down
ENSMUSG00000002055	Spag5	-2.57E+00	1.23E-12	down	-3.13E+00	3.96E-22	down
ENSMUSG00000020273	Papolg	-9.20E-01	5.40E-01	no	-4.00E+00	1.04E-04	down
ENSMUSG00000005198	Polr2a	-9.83E-01	1.00E-02	no	-2.29E+00	9.12E-14	down
ENSMUSG00000000751	Rpa1	-1.75E+00	2.17E-11	down	-2.08E+00	3.63E-24	down
ENSMUSG00000034274	Thoc5	-3.05E+00	2.21E-21	down	-2.78E+00	6.29E-22	down
ENSMUSG00000014195	Dnajc7	-2.12E+00	2.54E-17	down	-1.86E+00	9.05E-14	down
ENSMUSG00000039963	Ccdc40	-4.23E+00	3.53E-19	down	-4.11E+00	1.61E-26	down
ENSMUSG00000018570	2810408A11Rik	-3.61E+00	2.39E-56	down	-1.59E+00	1.98E-05	down
ENSMUSG00000011877	Git1	-3.20E+00	2.34E-80	down	-2.27E+00	9.20E-34	down
ENSMUSG00000020458	Rtn4	-1.56E-01	8.80E-01	no	-2.79E+00	7.87E-06	down
ENSMUSG00000020681	Ace	-4.41E+00	7.57E-69	down	-2.36E+00	8.08E-07	down
ENSMUSG00000001323	Srr	-3.65E+00	8.55E-07	down	-2.01E+00	1.39E-01	no
ENSMUSG00000025579	Gaa	2.83E+00	1.79E-04	up	4.94E-01	7.20E-01	no
ENSMUSG00000025137	Pcyt2	-6.59E-01	2.53E-01	no	-1.45E+00	1.97E-06	down
ENSMUSG00000025156	Gps1	-4.99E-01	4.99E-01	no	-1.75E+00	2.44E-06	down

ENSMUSG00000038453	Srcin1	-6.76E+00	2.01E-08	down	-5.35E+00	3.78E-02	no
ENSMUSG00000059995	Atxn7l3	-2.36E+00	1.44E-02	no	-3.59E+00	3.58E-06	down
ENSMUSG00000034706	Dnaic2	-4.35E+00	2.29E-19	down	-3.81E+00	2.21E-17	down
ENSMUSG00000020823	Sec14l1	-3.62E+00	2.90E-23	down	-2.50E+00	3.58E-08	down
ENSMUSG00000020464	Pnpt1	-8.46E-01	4.58E-01	no	-3.11E+00	6.77E-05	down
ENSMUSG00000018412	Kansl1	-2.05E+00	1.35E-03	no	-2.90E+00	1.05E-06	down
ENSMUSG00000010021	Kif19a	-5.40E+00	1.51E-03	no	-7.10E+00	1.01E-11	down
ENSMUSG00000040850	Psme4	-4.62E+00	1.49E-68	down	-3.63E+00	9.12E-40	down
ENSMUSG00000032740	Ccdc88a	-2.80E+00	3.19E-12	down	-2.27E+00	8.59E-07	down
ENSMUSG00000034543	Morc2a	-2.11E+00	1.19E-04	down	-3.12E+00	3.16E-10	down
ENSMUSG00000010277	2610507B11Rik	-2.99E+00	1.12E-24	down	-2.63E+00	3.59E-21	down
ENSMUSG00000017176	Nt5c3b	-1.46E+00	1.14E-07	down	1.04E+00	9.48E-03	no
ENSMUSG00000020481	Ankrd36	-2.35E+00	6.69E-46	down	-1.56E+00	8.36E-23	down
ENSMUSG00000018669	Cdk5rap3	-1.07E+00	6.30E-04	down	-1.39E+00	5.09E-09	down
ENSMUSG00000020863	Luc7l3	6.89E-01	5.17E-01	no	-2.55E+00	9.51E-05	down
ENSMUSG00000020280	Pus10	-2.74E+00	1.63E-05	down	-3.78E+00	1.33E-10	down
ENSMUSG00000006575	Rundc3a	-2.36E+00	1.06E-04	down	-1.48E+00	5.84E-02	no
ENSMUSG00000040594	Ranbp17	-3.15E+00	5.62E-07	down	-3.30E+00	1.02E-10	down
ENSMUSG00000000804	Usp32	-3.22E+00	1.48E-13	down	-3.89E+00	5.84E-24	down
ENSMUSG00000018401	Mtmr4	-2.04E+00	7.21E-04	down	-2.30E+00	3.32E-04	down
ENSMUSG00000020807	4933427D14Rik	-2.76E+00	1.04E-07	down	-2.35E+00	2.26E-06	down
ENSMUSG00000020752	Recql5	-3.15E+00	3.00E-04	down	-1.70E+00	2.16E-01	no
ENSMUSG00000044072	Eml6	-3.83E+00	5.23E-04	down	-2.34E+00	2.32E-01	no
ENSMUSG00000020534	Shmt1	-1.24E+00	3.43E-02	no	-1.89E+00	2.70E-05	down
ENSMUSG00000018160	Med1	-2.41E+00	1.08E-05	down	-3.14E+00	5.35E-10	down
ENSMUSG00000047126	Cltc	-1.57E+00	4.24E-04	down	-2.52E+00	3.74E-10	down
ENSMUSG00000017421	Zfp207	-6.44E-01	3.34E-01	no	-2.37E+00	3.39E-08	down
ENSMUSG00000020385	Clk4	-2.17E+00	9.84E-06	down	-2.03E+00	6.69E-04	down
ENSMUSG00000078812	Eif5a	1.07E+00	4.40E-43	up	2.22E-01	1.42E-03	no
ENSMUSG00000020882	Cacnb1	-4.91E+00	4.99E-12	down	-3.36E+00	1.70E-04	down
ENSMUSG00000018363	Smurf2	-2.42E+00	1.37E-05	down	-3.41E+00	5.82E-11	down
ENSMUSG00000002814	Top3a	-1.66E+00	8.46E-02	no	-2.99E+00	2.81E-06	down
ENSMUSG00000020741	Cluh	-2.56E+00	2.74E-05	down	-2.51E+00	1.88E-05	down
ENSMUSG00000025372	Baiap2	-2.62E+00	2.94E-17	down	-2.73E+00	1.96E-17	down
ENSMUSG00000025155	Dus1l	-1.45E+00	3.40E-04	down	-1.76E+00	8.53E-09	down
ENSMUSG00000009090	Ap1b1	-4.07E-01	5.77E-01	no	-1.78E+00	1.20E-04	down
ENSMUSG00000020865	Abcc3	-1.99E+00	2.27E-06	down	-1.99E+00	7.62E-08	down
ENSMUSG00000055805	Fmnl1	-3.57E+00	9.30E-06	down	-2.99E+00	2.49E-03	no
ENSMUSG00000020747	2310067B10Rik	-3.16E+00	1.68E-09	down	-2.59E+00	2.63E-07	down
ENSMUSG00000037275	Gemin5	-2.92E+00	2.34E-10	down	-2.45E+00	2.55E-07	down
ENSMUSG00000001036	Epn2	-4.00E-01	6.78E-01	no	-2.32E+00	5.02E-06	down
ENSMUSG00000025793	Hgs	-2.66E+00	1.73E-33	down	-2.18E+00	1.76E-21	down
ENSMUSG00000018395	Kif3a	-9.36E-01	5.43E-04	no	-1.13E+00	4.67E-06	down
ENSMUSG00000038909	Kat7	-1.85E+00	1.34E-07	down	-2.96E+00	5.91E-35	down
ENSMUSG00000009079	Ewsr1	-1.25E+00	9.14E-09	down	-1.71E+00	1.43E-19	down
ENSMUSG00000020818	Mfsd11	-2.34E+00	1.87E-07	down	-2.30E+00	8.09E-08	down
ENSMUSG00000042302	Ehbp1	-4.59E+00	1.81E-08	down	-4.71E+00	1.20E-09	down
ENSMUSG00000039781	Azi1	-3.63E+00	1.81E-10	down	-3.49E+00	3.72E-13	down
ENSMUSG00000041164	Zmiz2	-3.29E+00	9.30E-21	down	-3.33E+00	4.47E-25	down
ENSMUSG00000020366	Mapk9	-1.79E+00	1.36E-02	no	-2.33E+00	1.11E-04	down
ENSMUSG00000020456	Ogdh	-2.36E+00	2.95E-09	down	-2.36E+00	1.96E-06	down
ENSMUSG00000020451	Limk2	-2.21E+00	1.06E-04	down	-1.39E+00	1.76E-01	no
ENSMUSG00000020893	Per1	-3.92E+00	7.07E-12	down	-3.48E+00	1.22E-08	down
ENSMUSG00000020527	Myo19	-2.95E+00	1.01E-02	no	-3.13E+00	4.61E-04	down
ENSMUSG00000035152	Ap2b1	-4.16E+00	1.79E-51	down	-3.11E+00	4.73E-27	down
ENSMUSG00000020792	Exoc7	-1.44E+00	6.03E-03	no	-1.66E+00	1.48E-04	down
ENSMUSG00000020530	Ggnbp2	-2.46E+00	3.02E-56	down	-1.95E+00	2.83E-31	down
ENSMUSG00000020850	Prpf8	-1.34E+00	1.31E-02	no	-2.87E+00	1.50E-15	down
ENSMUSG00000040667	Nup88	-2.03E+00	8.27E-08	down	-2.13E+00	1.09E-07	down
ENSMUSG00000020549	Elac2	-1.07E+00	1.16E-01	no	-2.47E+00	2.55E-08	down
ENSMUSG00000035086	Becn1	-1.32E+00	1.18E-02	no	-1.77E+00	1.63E-05	down
ENSMUSG00000061306	Slc38a10	-2.13E+00	1.23E-04	down	-3.22E+00	2.00E-08	down
ENSMUSG00000007850	Hnrnph1	-1.06E+00	7.12E-04	down	-2.40E+00	1.62E-22	down
ENSMUSG00000020290	Xpo1	-1.72E+00	2.60E-02	no	-3.68E+00	1.84E-11	down

ENSMUSG00000017686	Rhot1	-2.97E+00	3.09E-08	down	-3.17E+00	2.00E-09	down
ENSMUSG00000009073	Nf2	-2.46E+00	3.03E-07	down	-2.71E+00	3.59E-09	down
ENSMUSG00000059474	Mbtd1	-1.61E+00	7.55E-02	no	-2.48E+00	2.29E-05	down
ENSMUSG00000042208	0610010F05Rik	-2.84E+00	2.65E-09	down	-2.78E+00	3.25E-11	down
ENSMUSG00000020929	Eftud2	-1.47E+00	1.12E-02	no	-2.29E+00	1.07E-06	down
ENSMUSG00000020486	43712	-3.82E+00	9.36E-24	down	-1.56E+00	2.85E-02	no
ENSMUSG00000017210	Med24	-2.30E+00	5.61E-09	down	-1.96E+00	1.35E-06	down
ENSMUSG00000020776	Fbf1	-2.69E+00	5.68E-05	down	-2.90E+00	1.25E-10	down
ENSMUSG00000005417	Mprip	-3.23E+00	4.00E-28	down	-2.48E+00	2.41E-12	down
ENSMUSG00000020694	Tlk2	-2.54E+00	6.67E-14	down	-2.49E+00	7.91E-18	down
ENSMUSG00000020900	Myh10	-3.50E+00	7.30E-09	down	-3.58E+00	2.21E-09	down
ENSMUSG00000020697	Lig3	-3.34E+00	2.80E-19	down	-2.85E+00	1.27E-14	down
ENSMUSG00000020389	Cdkl3	-3.70E+00	3.59E-54	down	-2.49E+00	1.45E-20	down
ENSMUSG00000001441	Npepps	-2.47E+00	1.51E-05	down	-2.98E+00	1.95E-10	down
ENSMUSG00000017774	Myo1c	-2.78E+00	1.32E-08	down	-2.72E+00	1.98E-07	down
ENSMUSG00000020716	Nf1	-3.21E+00	8.69E-04	down	-3.89E+00	4.93E-05	down
ENSMUSG00000005237	Dnahc2	-2.69E+00	1.35E-04	down	-3.47E+00	5.93E-17	down
ENSMUSG00000047284	Neurl4	-2.57E+00	6.98E-08	down	-2.76E+00	7.26E-10	down
ENSMUSG00000020728	Cep112	-2.84E+00	7.87E-16	down	-1.07E+00	1.08E-01	no
ENSMUSG00000020859	Spag9	-1.82E+00	7.50E-04	down	-1.61E+00	1.33E-02	no
ENSMUSG00000017119	Nbr1	-4.82E+00	4.32E-57	down	-2.38E+00	6.47E-06	down
ENSMUSG00000020333	Acsl6	-3.73E+00	2.50E-09	down	-3.64E+00	3.49E-10	down
ENSMUSG00000033987	Dnahc17	-2.83E+00	4.19E-13	down	-2.74E+00	6.53E-23	down
ENSMUSG00000091732	Gm17541	1.09E+01	8.18E-166	up	9.84E+00	4.05E-146	up
ENSMUSG00000063129	Aldoart2	-2.05E+00	2.27E-85	down	1.71E-01	5.68E-01	no
ENSMUSG00000091396	Spanxn4	-1.41E+00	1.62E-68	down	-1.53E-02	9.56E-01	no
ENSMUSG00000043060	Fscb	-5.45E+00	1.55E-70	down	-2.84E+00	7.06E-07	down
ENSMUSG00000043998	Mgat2	-3.26E-02	9.63E-01	no	-1.69E+00	4.48E-05	down
ENSMUSG00000072974	Gm4787	-5.05E+00	9.58E-65	down	-2.32E+00	1.85E-05	down
ENSMUSG00000072972	Adam4	-5.34E+00	2.45E-21	down	-3.01E+00	9.48E-03	no
ENSMUSG00000054383	Pnma1	-1.78E+00	2.06E-04	down	-1.94E+00	1.07E-08	down
ENSMUSG00000021041	2700073G19Rik	4.20E+00	9.69E-04	up	1.15E+00	2.03E-01	no
ENSMUSG00000092054	Kif4-ps	-3.99E+00	3.85E-03	no	-4.40E+00	9.97E-11	down
ENSMUSG00000051804	Adam6b	-5.23E+00	1.29E-21	down	-2.68E+00	1.51E-02	no
ENSMUSG00000043945	Adam6a	-4.28E+00	3.72E-27	down	-2.20E+00	1.24E-02	no
ENSMUSG00000021908	Gm6768	-1.67E+00	5.44E-09	down	-8.75E-01	1.20E-02	no
ENSMUSG00000043319	Cox8c	3.33E+00	0.00E+00	up	2.78E+00	0.00E+00	up
ENSMUSG00000096753	Fam181a	2.49E-01	4.76E-01	no	-1.01E+00	1.39E-14	down
ENSMUSG00000021101	4930408O17Rik	-2.50E+00	5.66E-06	down	-1.40E+00	2.54E-01	no
ENSMUSG00000021102	Glrx5	4.02E+00	7.55E-24	up	1.83E+00	1.02E-09	up
ENSMUSG00000020607	Fam84a	-3.78E+00	1.13E-08	down	-3.35E+00	1.73E-08	down
ENSMUSG00000069755	Zfp125	-2.77E+00	4.61E-11	down	-2.84E+00	4.96E-14	down
ENSMUSG00000035148	Gpr33	6.43E+00	8.48E-07	up	9.85E+00	7.05E-06	up
ENSMUSG00000079104	Prps1l3	-1.27E+00	2.76E-04	down	-1.09E+00	6.46E-03	no
ENSMUSG00000035560	Wdr20b	-2.46E+00	2.93E-24	down	-2.50E+00	2.64E-36	down
ENSMUSG00000045690	Wdr89	1.52E+00	2.86E-13	up	3.65E-01	9.74E-02	no
ENSMUSG00000059970	Hspa2	-1.78E+00	5.02E-140	down	-1.94E+00	4.18E-231	down
ENSMUSG00000008438	Adam21	-4.05E+00	3.25E-12	down	-1.06E+00	4.89E-01	no
ENSMUSG00000037957	Wdr20a	-2.22E+00	1.66E-05	down	-1.80E+00	8.96E-04	down
ENSMUSG00000020644	Id2	5.63E+00	5.43E-38	up	3.22E+00	1.38E-05	up
ENSMUSG00000020660	Pomc	3.03E+00	8.92E-11	up	1.27E+00	4.97E-10	up
ENSMUSG00000014905	Dnajb9	-2.12E+00	3.52E-24	down	-2.59E+00	1.47E-29	down
ENSMUSG00000020973	Dnaaf2	-1.17E+00	1.40E-02	no	-2.20E+00	7.34E-12	down
ENSMUSG00000021054	Sgpp1	-7.07E-02	9.60E-01	no	-2.30E+00	5.02E-04	down
ENSMUSG00000042734	Ttc9	-2.26E+00	5.27E-07	down	-2.50E+00	1.19E-09	down
ENSMUSG00000045064	Zc2hc1c	-2.71E+00	4.69E-10	down	-1.76E+00	1.31E-04	down
ENSMUSG00000079017	Ifi27l2a	5.60E+00	1.09E-18	up	3.59E+00	8.35E-04	up
ENSMUSG00000021290	2010107E04Rik	1.18E+00	5.83E-55	up	1.30E+00	6.94E-37	up
ENSMUSG00000037361	0610009D07Rik	1.26E+00	1.87E-03	no	-1.70E+00	5.96E-11	down
ENSMUSG00000062929	Cfl2	-9.34E-01	2.52E-03	no	-1.17E+00	1.06E-05	down
ENSMUSG00000034883	Lrr1	3.94E+00	2.62E-04	up	-7.80E-02	9.63E-01	no
ENSMUSG00000071234	Syndig1l	-2.56E+00	4.45E-03	no	-3.19E+00	1.17E-05	down
ENSMUSG00000021242	Npc2	1.44E+00	7.83E-04	up	-7.03E-02	9.21E-01	no
ENSMUSG00000034157	2310044G17Rik	-3.00E+00	7.62E-08	down	-3.08E+00	5.64E-09	down

ENSMUSG00000058207	Serpina3k	3.00E+00	8.38E-04	up	1.58E+00	2.41E-01	no
ENSMUSG00000041347	Bdkrb1	5.45E+00	5.28E-12	up	4.82E+00	3.21E-21	up
ENSMUSG00000006360	Crip1	5.12E+00	1.05E-141	up	3.03E+00	8.28E-17	up
ENSMUSG00000043061	Tmem18	2.20E-02	9.81E-01	no	-2.77E+00	6.47E-15	down
ENSMUSG00000047227	Gm527	-3.43E+00	5.39E-19	down	-1.35E+00	3.87E-02	no
ENSMUSG00000020948	Klhl28	-3.05E+00	1.53E-07	down	-7.93E-01	5.89E-01	no
ENSMUSG00000054894	Atp5s	-8.81E-01	1.53E-03	no	-1.04E+00	5.06E-05	down
ENSMUSG00000021067	Sav1	-2.05E+00	1.08E-08	down	-3.99E+00	1.45E-51	down
ENSMUSG00000034442	Trmt5	-2.30E+00	1.56E-05	down	-2.06E+00	6.35E-06	down
ENSMUSG00000021127	Zfp36l1	2.52E+00	1.42E-14	up	3.52E+00	6.81E-06	up
ENSMUSG00000056508	1700001K19Rik	-3.64E+00	1.71E-83	down	-1.74E+00	4.52E-09	down
ENSMUSG00000062054	Iah1	3.77E+00	4.43E-20	up	3.22E+00	2.46E-37	up
ENSMUSG00000020955	Ap4s1	-5.67E-01	4.01E-01	no	-1.65E+00	4.26E-06	down
ENSMUSG00000035133	Arhgap5	-2.27E+00	8.61E-09	down	-2.62E+00	7.75E-13	down
ENSMUSG00000021025	Nfkbia	4.97E+00	1.31E-33	up	3.71E+00	2.19E-13	up
ENSMUSG00000020993	Trappc6b	5.81E-01	1.30E-01	no	-1.20E+00	1.52E-08	down
ENSMUSG00000021096	Ppm1a	-4.47E+00	1.68E-65	down	-2.50E+00	7.64E-10	down
ENSMUSG00000085793	Lin52	-4.57E-01	6.86E-01	no	-2.76E+00	3.52E-07	down
ENSMUSG00000072919	Noxred1	-1.60E+00	8.52E-12	down	7.81E-01	3.45E-02	no
ENSMUSG00000021286	Zfyve21	4.35E+00	6.36E-05	up	1.73E+00	1.48E-01	no
ENSMUSG00000064326	Siva1	4.15E+00	1.66E-47	up	2.64E+00	1.27E-25	up
ENSMUSG00000037638	Zbtb42	-3.31E+00	5.19E-07	down	-3.85E+00	5.99E-22	down
ENSMUSG00000006356	Crip2	1.91E-01	3.74E-01	no	1.22E+00	2.04E-04	up
ENSMUSG00000061477	Rps7	-2.19E+00	1.52E-08	down	-3.19E+00	1.12E-24	down
ENSMUSG00000050545	Fam228b	-2.70E+00	1.13E-06	down	-7.40E-01	6.15E-01	no
ENSMUSG00000020949	Fkbp3	3.02E+00	9.98E-28	up	4.57E-01	1.12E-01	no
ENSMUSG00000021094	Dhrs7	5.24E+00	4.83E-04	up	2.22E+00	1.22E-01	no
ENSMUSG00000020585	Laptm4a	-1.23E+00	5.53E-20	down	-1.17E+00	1.65E-14	down
ENSMUSG00000001270	Ckb	5.64E+00	1.42E-07	up	3.36E+00	1.71E-01	no
ENSMUSG00000049792	Bag5	-2.43E+00	6.23E-143	down	-1.46E+00	8.97E-40	down
ENSMUSG00000037466	4930427A07Rik	-6.52E-01	3.12E-01	no	-2.06E+00	8.39E-09	down
ENSMUSG00000035983	Gm7008	-1.56E+00	3.63E-06	down	-2.14E-01	7.69E-01	no
ENSMUSG00000079177	Fam228a	-1.45E+00	1.29E-03	no	-2.15E+00	1.34E-11	down
ENSMUSG00000021116	Eif2s1	-1.91E+00	1.62E-18	down	-2.07E+00	3.41E-24	down
ENSMUSG00000021264	Yy1	-5.66E-01	4.20E-01	no	-2.54E+00	1.85E-10	down
ENSMUSG00000020639	Pfn4	-2.74E+00	9.57E-76	down	-1.94E+00	2.91E-36	down
ENSMUSG00000021028	Mbip	3.50E-01	7.36E-01	no	-2.45E+00	1.74E-05	down
ENSMUSG00000020994	Pnn	2.61E-01	6.41E-01	no	-1.71E+00	3.47E-07	down
ENSMUSG00000059436	Max	-1.13E+00	4.12E-01	no	-3.83E+00	2.86E-06	down
ENSMUSG00000056987	Fam71d	-3.89E+00	3.34E-173	down	-1.48E+00	8.09E-08	down
ENSMUSG00000021114	Atp6v1d	-8.92E-01	8.44E-08	no	-1.98E+00	1.45E-63	down
ENSMUSG00000049106	Dcaf5	-4.50E+00	2.58E-23	down	-3.34E+00	7.11E-11	down
ENSMUSG00000032705	Exd2	-6.32E-01	4.28E-01	no	-2.62E+00	1.54E-09	down
ENSMUSG00000021131	Erh	2.05E+00	2.01E-20	up	3.85E-01	2.16E-02	no
ENSMUSG00000021033	Gstz1	-2.01E+00	7.90E-08	down	-1.97E+00	1.02E-08	down
ENSMUSG00000021037	Ahsa1	-7.23E-01	1.33E-02	no	-1.59E+00	1.92E-12	down
ENSMUSG00000072849	Serpina1e	2.86E+00	7.10E-05	up	3.21E+00	7.88E-05	up
ENSMUSG00000020633	Dcdc2c	-4.72E+00	3.61E-12	down	-2.18E+00	1.13E-01	no
ENSMUSG00000043153	Ispd	-3.62E+00	3.79E-04	down	-1.56E+00	3.94E-01	no
ENSMUSG00000020988	L2hgdh	-1.54E+00	6.59E-02	no	-2.44E+00	2.03E-04	down
ENSMUSG00000020990	Cdkl1	-1.85E+00	3.77E-04	down	-1.69E+00	5.75E-05	down
ENSMUSG00000021056	Tex21	-4.03E+00	5.64E-114	down	-1.94E+00	7.93E-12	down
ENSMUSG00000042320	Prox2	-2.36E+00	1.41E-06	down	-1.45E-01	9.22E-01	no
ENSMUSG00000034258	Mfsd7c	-2.82E+00	1.31E-08	down	-9.90E-01	4.11E-01	no
ENSMUSG00000021178	Psmc1	-4.03E-01	1.54E-01	no	-1.42E+00	2.10E-15	down
ENSMUSG00000041712	Ubr7	-3.30E+00	2.14E-27	down	-2.12E+00	3.79E-07	down
ENSMUSG00000021258	Ccnk	-5.11E+00	4.54E-151	down	-4.30E+00	7.60E-125	down
ENSMUSG00000037594	BC022687	-2.44E+00	1.74E-11	down	-1.56E+00	4.69E-06	down
ENSMUSG00000012211	Tex22	-1.25E+00	4.09E-30	down	-1.13E+00	3.04E-32	down
ENSMUSG00000052221	Ppp1r36	-3.18E+00	7.19E-74	down	-2.77E+00	7.44E-68	down
ENSMUSG00000041702	Btbd7	-2.02E+00	2.82E-03	no	-2.45E+00	3.43E-05	down
ENSMUSG00000011179	Odc1	-2.59E+00	2.10E-35	down	-2.25E+00	2.82E-27	down
ENSMUSG00000044573	Acp1	-2.04E+00	4.68E-03	no	-3.15E+00	4.00E-08	down
ENSMUSG00000090258	Churc1	4.13E+00	3.89E-12	up	1.60E+00	3.00E-04	up

ENSMUSG00000042628	Zfyve1	-3.56E+00	1.21E-24	down	-1.46E+00	5.18E-03	no
ENSMUSG00000090812	Samd15	-2.19E+00	4.06E-11	down	-1.99E+00	2.00E-10	down
ENSMUSG00000021076	Actr10	-2.00E+00	4.75E-39	down	-1.94E+00	7.35E-31	down
ENSMUSG00000020664	Dld	-2.02E+00	1.13E-12	down	-2.72E+00	3.70E-26	down
ENSMUSG00000021022	Ppp2r3c	-2.30E+00	3.02E-10	down	-1.86E+00	3.08E-09	down
ENSMUSG00000021051	Ppp2r5e	-2.63E+00	1.91E-17	down	-2.64E+00	6.09E-18	down
ENSMUSG00000021250	Fos	4.31E+00	4.29E-19	up	3.08E+00	1.98E-03	no
ENSMUSG00000021039	Snw1	-1.74E+00	1.62E-07	down	-1.57E+00	5.17E-08	down
ENSMUSG00000041781	Cpsf2	-2.05E+00	3.36E-04	down	-2.29E+00	1.42E-05	down
ENSMUSG00000059669	Taf1b	-1.09E+00	3.41E-03	no	-1.99E+00	8.10E-17	down
ENSMUSG00000020650	Bcap29	-2.48E+00	3.76E-69	down	-3.01E+00	5.74E-140	down
ENSMUSG00000020577	Tspan13	-1.48E+00	1.37E-01	no	-3.44E+00	1.35E-06	down
ENSMUSG00000021020	Srp54c	-3.66E+00	1.48E-05	down	-2.81E+00	8.59E-02	no
ENSMUSG00000021066	Atl1	-2.21E+00	1.32E-06	down	-2.30E+00	2.79E-08	down
ENSMUSG00000021112	Mpp5	-1.89E+00	1.87E-05	down	-2.02E+00	2.26E-04	down
ENSMUSG00000090843	Gm17673	-4.42E+00	1.93E-27	down	-1.10E+00	2.26E-01	no
ENSMUSG00000004789	Dlst	-2.03E+00	2.25E-09	down	-3.13E+00	1.11E-32	down
ENSMUSG00000059114	Gm6772	-4.85E+00	5.43E-65	down	-1.95E+00	3.97E-05	down
ENSMUSG00000020545	Lrrc72	-2.63E+00	1.08E-12	down	-2.67E-01	8.09E-01	no
ENSMUSG00000020657	Dnajc27	-3.31E+00	8.02E-24	down	-2.90E+00	3.15E-21	down
ENSMUSG00000021003	Galc	-3.14E+00	1.81E-04	down	-3.45E+00	9.75E-06	down
ENSMUSG00000001175	Calm1	2.02E+00	2.10E-163	up	1.68E+00	3.97E-223	up
ENSMUSG00000041323	Ak7	-3.91E+00	7.83E-29	down	-3.76E+00	2.82E-34	down
ENSMUSG00000011158	Brf1	-1.40E+00	3.11E-05	down	-1.90E+00	1.85E-12	down
ENSMUSG00000054309	Cpsf3	-1.24E+00	1.35E-02	no	-1.81E+00	2.93E-06	down
ENSMUSG00000056459	Zbtb25	-2.03E+00	5.31E-03	no	-2.32E+00	6.47E-04	down
ENSMUSG00000037904	Ankrd9	-1.96E+00	7.11E-07	down	5.04E-01	6.28E-01	no
ENSMUSG00000047446	Arl4a	-1.72E+00	1.85E-28	down	-1.87E+00	2.75E-38	down
ENSMUSG00000035614	Fam179b	-2.97E+00	1.78E-14	down	-1.34E+00	2.79E-02	no
ENSMUSG00000021124	Vti1b	3.61E-01	3.71E-01	no	-1.03E+00	2.47E-05	down
ENSMUSG00000066441	Rdh11	-2.77E+00	1.73E-36	down	-2.56E+00	1.04E-47	down
ENSMUSG00000020962	Gtf2a1	-1.43E+00	1.22E-01	no	-3.03E+00	2.74E-06	down
ENSMUSG00000021285	Ppp1r13b	-1.67E+00	3.77E-03	no	-2.52E+00	3.23E-05	down
ENSMUSG00000020659	Cbll1	-1.66E+00	3.57E-03	no	-1.95E+00	3.81E-05	down
ENSMUSG00000021086	Ccdc175	-3.14E+00	3.52E-64	down	-1.99E+00	1.50E-17	down
ENSMUSG00000034126	Pomt2	-2.70E+00	4.28E-13	down	-2.54E+00	2.81E-09	down
ENSMUSG00000042050	Wdr60	-1.12E+00	1.84E-06	down	-4.41E-01	1.19E-01	no
ENSMUSG00000021171	Esyt2	-1.42E+00	9.94E-02	no	-2.47E+00	7.26E-04	down
ENSMUSG00000056899	Immp2l	-9.94E-01	1.75E-02	no	-1.07E+00	4.24E-04	down
ENSMUSG00000034290	Nek9	-2.84E+00	1.80E-04	down	-3.45E+00	4.25E-07	down
ENSMUSG00000057963	Itpk1	-2.48E+00	4.31E-07	down	-2.25E+00	8.39E-05	down
ENSMUSG00000035933	Cog5	-2.42E+00	2.17E-03	no	-2.38E+00	5.10E-04	down
ENSMUSG00000057265	Ccdc176	-1.90E+00	2.25E-56	down	-1.40E+00	2.58E-42	down
ENSMUSG00000021143	Pacs2	-3.55E+00	1.68E-31	down	-1.11E+00	9.15E-02	no
ENSMUSG00000020636	Allc	-4.18E+00	2.07E-255	down	-1.32E+00	8.99E-10	down
ENSMUSG00000020570	Sypl	-1.23E+00	4.52E-04	down	-2.90E-01	6.57E-01	no
ENSMUSG00000021024	Psma6	-1.32E+00	4.47E-67	down	-3.24E-01	3.09E-03	no
ENSMUSG00000066438	Plekhd1	-3.01E+00	1.52E-02	no	-3.76E+00	1.73E-05	down
ENSMUSG00000019969	Psen1	-3.10E+00	6.90E-50	down	-2.38E+00	4.24E-22	down
ENSMUSG00000021245	Mlh3	-1.05E+00	3.48E-01	no	-2.52E+00	2.55E-06	down
ENSMUSG00000021209	Ppp4r4	-3.36E+00	2.06E-14	down	-2.81E+00	6.85E-09	down
ENSMUSG00000020952	Scfd1	-5.55E-01	3.49E-01	no	-2.04E+00	2.95E-09	down
ENSMUSG00000021192	Golga5	-2.72E+00	3.79E-17	down	-2.19E+00	8.05E-08	down
ENSMUSG00000037149	Ddx1	-1.55E+00	1.79E-23	down	-1.62E+00	7.93E-28	down
ENSMUSG00000034574	Daam1	-3.07E+00	3.58E-04	down	-3.03E+00	3.32E-04	down
ENSMUSG00000047014	Catsperb	-4.52E+00	1.09E-15	down	-4.61E+00	1.92E-25	down
ENSMUSG00000021097	Clmn	-6.09E+00	1.89E-14	down	-4.34E+00	1.41E-02	no
ENSMUSG00000017843	Ppp2r5c	-3.26E+00	1.04E-56	down	-2.75E+00	1.45E-52	down
ENSMUSG00000020608	Smc6	-1.73E+00	1.94E-09	down	-2.70E+00	8.68E-33	down
ENSMUSG00000021234	Fam161b	-1.67E+00	6.12E-02	no	-2.70E+00	4.47E-06	down
ENSMUSG00000021203	Otub2	-4.37E+00	9.16E-49	down	-2.58E+00	5.76E-07	down
ENSMUSG00000041992	Rapgef5	-3.33E+00	1.65E-05	down	-5.68E-01	8.22E-01	no
ENSMUSG00000056458	Stk30	-2.58E+00	1.24E-22	down	-2.02E+00	2.15E-16	down
ENSMUSG00000054302	Eapp	-1.26E+00	8.45E-04	down	-1.34E+00	6.10E-05	down

ENSMUSG00000041846	Smek1	-2.60E+00	3.63E-06	down	-3.06E+00	1.54E-05	down
ENSMUSG00000076432	Ywhaq	-8.09E-01	5.99E-02	no	-1.95E+00	5.46E-11	down
ENSMUSG00000020656	Grhl1	-2.04E+00	8.29E-02	no	-3.30E+00	3.61E-06	down
ENSMUSG00000020978	Klhdc2	-1.51E+00	1.82E-04	down	-1.57E+00	1.75E-05	down
ENSMUSG00000020628	Trappc12	-2.27E+00	9.36E-05	down	-2.22E+00	4.99E-05	down
ENSMUSG00000021257	Angel1	-3.19E+00	3.25E-41	down	-2.54E+00	3.63E-28	down
ENSMUSG00000020571	Pdia6	7.51E-02	7.97E-01	no	-1.19E+00	1.42E-09	down
ENSMUSG00000021087	Rtn1	-2.50E+00	8.94E-06	down	-1.80E+00	2.65E-03	no
ENSMUSG00000021190	Lgmn	2.66E+00	7.11E-06	up	4.03E-02	9.70E-01	no
ENSMUSG00000020622	Nt5c1b	-2.97E+00	2.56E-144	down	-4.53E-01	5.92E-02	no
ENSMUSG00000020982	Nemf	-1.21E+00	9.22E-04	down	-1.62E+00	3.71E-06	down
ENSMUSG00000020652	Cenpo	-5.28E-01	7.73E-01	no	-2.76E+00	5.52E-04	down
ENSMUSG00000054003	Tdrd9	-1.88E+00	6.27E-07	down	-2.54E+00	6.09E-20	down
ENSMUSG00000021140	Pcnx	-2.82E+00	2.29E-06	down	-2.89E+00	3.02E-09	down
ENSMUSG00000036257	Pnpla8	-3.26E+00	1.70E-13	down	-1.95E+00	2.88E-03	no
ENSMUSG00000021065	Fut8	-2.61E+00	3.41E-08	down	-2.42E+00	4.50E-06	down
ENSMUSG00000046782	Ttc6	-3.74E+00	4.59E-15	down	-3.00E+00	2.62E-10	down
ENSMUSG00000021270	Hsp90aa1	1.59E+00	1.48E-110	up	6.35E-01	1.32E-26	no
ENSMUSG00000020669	Sh3yl1	-3.16E+00	2.45E-06	down	-1.15E+00	3.06E-01	no
ENSMUSG00000021189	Atxn3	-3.37E+00	1.31E-22	down	-1.71E+00	4.09E-04	down
ENSMUSG00000020593	Lpin1	-4.69E+00	1.80E-60	down	-2.52E+00	7.65E-09	down
ENSMUSG00000021007	Spata7	-4.40E+00	2.40E-55	down	-1.98E+00	2.86E-05	down
ENSMUSG00000042700	Sipa1l1	-3.05E+00	5.05E-05	down	-3.73E-01	8.56E-01	no
ENSMUSG00000042523	Dnalc1	-2.38E+00	2.15E-02	no	-2.77E+00	4.87E-04	down
ENSMUSG00000020986	Sec23a	-3.15E+00	2.36E-08	down	-3.22E+00	3.14E-08	down
ENSMUSG00000020590	Snx13	-2.75E+00	7.49E-05	down	-2.15E+00	1.25E-02	no
ENSMUSG00000035293	G2e3	-2.61E+00	2.51E-04	down	-1.66E+00	1.41E-01	no
ENSMUSG00000020576	Nbas	-2.84E+00	4.12E-06	down	-3.11E+00	3.16E-11	down
ENSMUSG00000021012	Zc3h14	-3.06E+00	7.69E-60	down	-2.70E+00	5.88E-45	down
ENSMUSG00000021266	Wars	-1.65E+00	3.00E-02	no	-2.23E+00	5.24E-05	down
ENSMUSG00000021221	Dpf3	-2.04E+00	4.26E-05	down	3.81E-01	7.79E-01	no
ENSMUSG00000056770	Setd3	-2.00E+00	2.25E-05	down	-2.82E+00	4.25E-12	down
ENSMUSG00000021013	Ttc8	-6.66E-01	6.26E-01	no	-2.51E+00	1.19E-04	down
ENSMUSG00000021177	Tdp1	-2.41E+00	3.71E-10	down	-1.33E+00	3.32E-03	no
ENSMUSG00000021038	Vipas39	-1.02E+00	1.05E-01	no	-1.75E+00	3.15E-04	down
ENSMUSG00000021182	Ccdc88c	-2.85E+00	3.92E-06	down	-3.37E+00	1.25E-10	down
ENSMUSG00000042350	1110018G07Rik	-4.31E+00	3.97E-12	down	-2.62E+00	1.12E-03	no
ENSMUSG00000021188	Trip11	-8.61E-01	3.83E-01	no	-2.52E+00	9.21E-04	down
ENSMUSG00000035021	Baz1a	-1.58E+00	1.18E-03	no	-2.47E+00	9.92E-17	down
ENSMUSG00000021134	Srsf5	-4.39E-01	3.02E-01	no	-2.05E+00	2.49E-10	down
ENSMUSG00000034601	2700049A03Rik	-2.86E+00	4.92E-14	down	-2.44E+00	1.03E-12	down
ENSMUSG00000021281	Tnfaip2	-3.27E+00	4.13E-11	down	-2.42E+00	1.01E-03	no
ENSMUSG00000021027	Ralgapa1	-2.03E+00	3.16E-04	down	-1.98E+00	1.23E-03	no
ENSMUSG00000035247	Hectd1	-5.49E-01	5.29E-01	no	-2.08E+00	2.36E-04	down
ENSMUSG00000021224	Numb	-1.26E+00	1.85E-01	no	-3.42E+00	2.44E-07	down
ENSMUSG00000021236	Entpd5	-2.94E+00	4.73E-04	down	-2.57E+00	2.51E-02	no
ENSMUSG00000018707	Dync1h1	-2.96E+00	3.93E-20	down	-3.02E+00	2.37E-23	down
ENSMUSG00000021244	Ylpm1	-3.42E+00	1.53E-11	down	-3.33E+00	4.54E-13	down
ENSMUSG00000034145	Tmem63c	-2.34E+00	2.47E-04	down	3.30E-01	8.72E-01	no
ENSMUSG00000066643	Wdr35	-2.64E+00	1.18E-10	down	-2.65E+00	2.00E-13	down
ENSMUSG00000061533	Cep128	-3.51E+00	1.99E-21	down	-3.05E+00	2.46E-15	down
ENSMUSG00000021144	Mta1	-4.23E+00	4.22E-17	down	-4.38E+00	4.01E-19	down
ENSMUSG00000021090	Lrrc9	-5.00E+00	6.55E-46	down	-3.00E+00	6.46E-08	down
ENSMUSG00000021111	Papola	-1.62E+00	3.50E-06	down	-1.65E+00	1.52E-04	down
ENSMUSG00000012609	Ttll5	-3.88E+00	2.53E-14	down	-3.67E+00	7.78E-14	down
ENSMUSG00000075053	Vdac3-ps1	-1.51E+00	7.53E-53	down	-9.93E-01	1.87E-27	no
ENSMUSG00000059395	Nkapl	-1.03E+00	3.06E-09	down	-7.32E-01	2.56E-08	no
ENSMUSG00000079941	Gm11273	1.68E+00	2.21E-33	up	1.33E+00	3.56E-29	up
ENSMUSG00000063021	Hist1h2ak	6.55E+00	2.55E-15	up	5.59E+00	2.92E-08	up
ENSMUSG00000060639	Hist1h4i	3.93E+00	6.32E-04	up	2.20E+00	3.80E-02	no
ENSMUSG00000069301	Hist1h2ag	3.23E+00	4.93E-07	up	4.53E+00	1.43E-04	up
ENSMUSG00000061991	Hist1h2af	1.03E+01	2.43E-23	up	6.59E+00	1.99E-15	up
ENSMUSG00000061482	Hist1h4d	6.36E+00	7.91E-09	up	6.22E+00	7.57E-05	up
ENSMUSG00000050799	Hist1h2ba	2.04E+00	1.11E-05	up	7.27E-01	2.94E-01	no

ENSMUSG00000060081	Hist1h2aa	2.73E+00	6.30E-10	up	1.21E+00	4.07E-02	no
ENSMUSG00000045835	Hdgfl1	-1.54E+00	1.68E-33	down	-1.42E+00	2.79E-37	down
ENSMUSG00000090497	Gm17611	-5.75E+00	0.00E+00	down	-2.11E+00	4.84E-18	down
ENSMUSG00000091383	Hist1h2al	3.34E+00	3.63E-04	up	2.42E+00	5.45E-04	up
ENSMUSG00000044444	Pfn3	8.21E-01	1.76E-10	no	2.75E+00	3.34E-47	up
ENSMUSG00000007836	Hnrnpa0	-1.29E+00	8.55E-02	no	-3.58E+00	1.16E-21	down
ENSMUSG00000064063	BC048507	2.53E+00	5.24E-110	up	3.41E+00	4.74E-60	up
ENSMUSG00000066407	Gm10263	2.48E+00	2.26E-22	up	6.79E-01	1.19E-02	no
ENSMUSG00000062382	Gm10116	9.14E+00	0.00E+00	up	6.93E+00	8.07E-129	up
ENSMUSG00000093668	Pou5f2	-1.35E+00	2.31E-01	no	-3.22E+00	1.94E-26	down
ENSMUSG00000046957	Spz1	-3.70E+00	0.00E+00	down	-1.62E+00	8.59E-37	down
ENSMUSG00000045022	1700024P04Rik	-3.19E+00	2.84E-186	down	-1.94E+00	9.75E-22	down
ENSMUSG00000097022	BC001981	8.14E+00	2.21E-09	up	4.60E+00	2.53E-05	up
ENSMUSG00000059751	Rps3a3	-7.19E-01	2.80E-01	no	-2.78E+00	4.54E-09	down
ENSMUSG00000094237	AF067063	#VALUE!	NA	no	5.39E+00	3.05E-04	up
ENSMUSG00000042275	Pelo	-1.52E+00	4.23E-02	no	-2.59E+00	5.94E-07	down
ENSMUSG00000094114	Gm21967	-4.23E+00	5.22E-75	down	-3.81E+00	1.19E-69	down
ENSMUSG00000012429	Mplkip	-3.56E-01	5.60E-01	no	-1.75E+00	4.40E-07	down
ENSMUSG00000018102	Hist1h2bc	5.00E+00	2.06E-10	up	2.10E+00	1.03E-02	no
ENSMUSG00000038246	Fam50b	-2.24E+00	6.08E-76	down	-1.15E+00	5.91E-26	down
ENSMUSG00000091264	Smim13	-2.44E+00	1.98E-06	down	-3.32E+00	7.06E-21	down
ENSMUSG00000021607	Mrpl36	1.89E+00	2.21E-08	up	8.61E-01	4.94E-04	no
ENSMUSG00000041995	Zbed3	-2.27E+00	1.24E-05	down	-2.28E+00	1.72E-05	down
ENSMUSG00000092116	Gm10320	2.36E+00	2.25E-09	up	7.99E-01	2.68E-02	no
ENSMUSG00000016982	Pom121l2	-3.98E+00	2.31E-57	down	-3.45E+00	3.17E-51	down
ENSMUSG00000006717	Acot13	3.41E+00	7.36E-06	up	2.03E+00	2.30E-02	no
ENSMUSG00000062248	Cks2	4.48E+00	7.24E-121	up	1.79E+00	1.30E-73	up
ENSMUSG00000034686	Prr7	-1.71E+00	1.10E-01	no	-2.88E+00	1.12E-04	down
ENSMUSG00000035367	Rmi1	-2.85E+00	1.44E-05	down	-3.47E+00	5.04E-12	down
ENSMUSG00000056223	Spata31	-2.94E+00	3.51E-53	down	2.64E-01	6.53E-01	no
ENSMUSG00000021534	1700001L19Rik	-1.87E+00	1.11E-30	down	4.38E-01	2.08E-01	no
ENSMUSG00000069184	Zfp72	-4.61E+00	2.45E-13	down	-1.62E+00	2.45E-01	no
ENSMUSG00000052075	1700029F12Rik	-1.29E+00	5.78E-61	down	-8.29E-01	4.27E-29	no
ENSMUSG00000068184	Ndufaf2	2.01E+00	6.52E-94	up	1.62E+00	3.13E-64	up
ENSMUSG00000001707	Eef1e1	2.52E+00	2.67E-06	up	3.46E-02	9.56E-01	no
ENSMUSG00000000078	Klf6	3.74E+00	5.62E-06	up	1.52E+00	1.91E-01	no
ENSMUSG00000050876	Spata31d1a	-6.73E+00	2.33E-16	down	-4.64E+00	5.51E-05	down
ENSMUSG00000091311	Spata31d1b	-5.85E+00	2.49E-29	down	-3.18E+00	3.45E-03	no
ENSMUSG00000043986	Spata31d1d	-5.81E+00	2.41E-36	down	-2.79E+00	1.98E-03	no
ENSMUSG00000074849	Spata31d1c	-7.49E+00	2.45E-12	down	-5.16E+00	3.33E-02	no
ENSMUSG00000057396	Zfp759	-5.41E+00	4.16E-15	down	-2.46E+00	5.15E-02	no
ENSMUSG00000055480	Zfp458	-4.97E+00	1.93E-07	down	-2.38E+00	2.63E-01	no
ENSMUSG00000058246	Gm10037	-2.91E+00	9.29E-08	down	-7.10E-01	4.70E-01	no
ENSMUSG00000042043	Tbca	1.02E+00	7.08E-21	up	-3.59E-01	1.72E-05	no
ENSMUSG00000021764	Ndufs4	-5.56E-01	2.76E-03	no	-1.30E+00	1.85E-23	down
ENSMUSG00000021731	Mrps30	-1.53E+00	1.04E-04	down	-1.93E+00	1.30E-10	down
ENSMUSG00000071451	Psmg4	2.35E+00	1.09E-08	up	1.24E+00	2.00E-04	up
ENSMUSG00000038982	Bloc1s5	-8.47E-01	2.17E-01	no	-1.74E+00	3.54E-05	down
ENSMUSG00000021486	Prelid1	3.68E+00	8.89E-19	up	1.35E+00	4.06E-05	up
ENSMUSG00000049902	4921517D22Rik	-6.14E+00	4.05E-06	down	-4.04E+00	1.06E-01	no
ENSMUSG00000051054	1700014D04Rik	-4.88E+00	9.46E-36	down	-1.94E+00	1.24E-02	no
ENSMUSG00000021590	Spata9	-3.10E+00	4.60E-73	down	-4.23E-01	2.61E-01	no
ENSMUSG00000021537	Cetn3	-2.03E+00	2.24E-62	down	-2.62E+00	1.18E-146	down
ENSMUSG00000049115	Agtr1a	-4.15E+00	7.36E-07	down	-3.40E+00	3.20E-04	down
ENSMUSG00000021361	Tmem14c	3.85E+00	3.52E-07	up	1.74E+00	3.59E-02	no
ENSMUSG00000037960	1110007C09Rik	2.86E+00	4.66E-14	up	1.02E+00	7.19E-03	no
ENSMUSG00000044792	Isca1	-2.64E+00	8.31E-12	down	-2.80E+00	1.10E-14	down
ENSMUSG00000021482	Aaed1	-1.70E+00	2.21E-05	down	-1.93E+00	2.48E-05	down
ENSMUSG00000021520	Uqcrb	2.44E+00	7.15E-39	up	9.82E-01	9.01E-12	no
ENSMUSG00000034334	Fam151b	-4.87E+00	6.80E-10	down	-4.68E+00	4.25E-07	down
ENSMUSG00000060739	Nsa2	-2.68E+00	1.29E-04	down	-3.40E+00	1.82E-09	down
ENSMUSG00000036721	Zscan12	-3.86E+00	1.92E-22	down	-3.10E+00	7.28E-16	down
ENSMUSG00000069237	C78339	-3.53E+00	1.61E-13	down	-3.01E+00	6.82E-07	down
ENSMUSG00000021453	Gadd45g	3.39E+00	2.11E-07	up	2.54E+00	1.03E-02	no

ENSMUSG00000095432	Zfp748	-3.88E+00	2.78E-03	no	-5.00E+00	2.12E-10	down
ENSMUSG00000021718	4933425L06Rik	-8.17E-01	4.73E-01	no	-2.13E+00	1.36E-04	down
ENSMUSG00000015671	Psma2	1.70E+00	5.68E-12	up	-2.06E-01	3.77E-01	no
ENSMUSG00000021414	Fam217a	-4.02E+00	1.55E-107	down	-1.28E+00	2.31E-04	down
ENSMUSG00000063200	Nol7	2.43E+00	9.19E-08	up	4.02E-01	4.49E-01	no
ENSMUSG00000034789	Rab24	3.50E+00	3.32E-09	up	4.99E-01	2.76E-01	no
ENSMUSG00000021484	Lman2	-4.51E-01	2.36E-01	no	-1.13E+00	4.73E-04	down
ENSMUSG00000021501	Caml	-1.75E+00	2.72E-05	down	-1.89E+00	6.37E-08	down
ENSMUSG00000021476	Habp4	-4.13E+00	7.54E-61	down	-2.57E+00	9.93E-11	down
ENSMUSG00000021477	Ctsl	3.22E+00	6.16E-22	up	9.01E-01	2.27E-03	no
ENSMUSG00000021483	Cdk20	-7.94E-01	6.10E-02	no	-1.62E+00	6.32E-12	down
ENSMUSG00000004341	Gpx6	-3.06E+00	2.84E-11	down	-5.23E-01	6.73E-01	no
ENSMUSG00000032621	Srek1	4.78E-01	4.32E-01	no	-2.04E+00	1.68E-09	down
ENSMUSG00000093930	Hmgcs1	-2.82E+00	4.54E-08	down	-2.29E+00	3.00E-04	down
ENSMUSG00000019132	BC005537	-3.23E+00	1.43E-34	down	-3.55E+00	9.94E-41	down
ENSMUSG00000038042	Ptpdc1	-3.18E+00	6.56E-43	down	-3.15E+00	5.59E-54	down
ENSMUSG00000069189	Zdhhc11	-2.55E+00	1.05E-18	down	4.37E-01	5.59E-01	no
ENSMUSG00000021622	Ckmt2	-2.04E+00	3.52E-08	down	-7.12E-01	1.52E-01	no
ENSMUSG00000021650	Ptcd2	-1.64E+00	1.87E-03	no	-2.15E+00	5.22E-09	down
ENSMUSG00000021339	Mrs2	-2.52E+00	4.82E-09	down	-2.67E+00	1.24E-16	down
ENSMUSG00000021415	4933417A18Rik	-3.32E+00	9.62E-84	down	-7.14E-01	4.72E-02	no
ENSMUSG00000021218	Gdi2	-2.68E+00	1.48E-50	down	-2.71E+00	5.21E-41	down
ENSMUSG00000021606	Ndufs6	3.22E+00	3.47E-205	up	2.28E+00	5.05E-213	up
ENSMUSG00000021690	Jmy	-3.02E+00	6.36E-05	down	-1.51E+00	3.73E-01	no
ENSMUSG00000035958	Tdp2	-6.74E-02	8.96E-01	no	-1.55E+00	1.62E-05	down
ENSMUSG00000021572	Cep72	-2.36E+00	4.19E-29	down	-1.99E+00	1.09E-23	down
ENSMUSG00000001542	Ell2	-4.38E+00	1.16E-27	down	-2.27E+00	7.11E-03	no
ENSMUSG00000043190	Rfesd	-1.07E+00	9.38E-02	no	-2.15E+00	5.01E-07	down
ENSMUSG00000035762	Tmem161b	-1.31E+00	8.52E-03	no	-2.14E+00	1.17E-07	down
ENSMUSG00000062822	4833420G17Rik	-1.55E+00	5.36E-02	no	-2.79E+00	3.71E-08	down
ENSMUSG00000021420	Fars2	-1.13E+00	3.06E-05	down	-1.29E+00	2.05E-08	down
ENSMUSG00000069805	Fbp1	-1.16E+00	3.76E-54	down	-7.46E-01	1.29E-36	no
ENSMUSG00000021518	Ptdss1	-2.12E+00	3.63E-06	down	-1.92E+00	2.91E-05	down
ENSMUSG00000034152	Exoc3	-1.43E+00	5.85E-04	down	-1.29E+00	3.49E-03	no
ENSMUSG00000021671	Poc5	-2.33E+00	8.67E-05	down	-3.03E+00	2.81E-12	down
ENSMUSG00000038546	Ranbp9	-4.27E+00	6.07E-112	down	-3.57E+00	1.54E-74	down
ENSMUSG00000032846	Zswim6	-3.82E+00	6.16E-06	down	-3.11E+00	7.91E-03	no
ENSMUSG00000047789	Slc38a9	-2.72E+00	2.23E-04	down	-2.15E+00	8.39E-03	no
ENSMUSG00000094870	Zfp131	-8.35E-01	1.41E-01	no	-2.17E+00	3.82E-07	down
ENSMUSG00000021337	Scgn	-3.38E+00	1.44E-06	down	-2.13E+00	9.90E-02	no
ENSMUSG00000046949	Nqo2	-3.76E+00	9.12E-04	down	-2.36E+00	2.22E-01	no
ENSMUSG00000025869	Nop16	2.19E+00	1.25E-05	up	-2.62E-01	6.14E-01	no
ENSMUSG00000097333	Zfp87	-2.49E+00	9.04E-04	down	3.07E-01	9.02E-01	no
ENSMUSG00000021629	Slc30a5	-7.81E-01	4.65E-01	no	-3.49E+00	6.39E-06	down
ENSMUSG00000057069	Ero1lb	-1.80E+00	4.25E-12	down	-1.54E+00	2.25E-11	down
ENSMUSG00000021326	Trim27	-1.96E+00	7.52E-21	down	-1.08E+00	2.55E-06	down
ENSMUSG00000021364	Elovl2	-3.57E+00	1.55E-32	down	-3.61E+00	1.04E-56	down
ENSMUSG00000038068	Rnf144b	-3.79E+00	1.36E-02	no	-5.41E+00	1.10E-15	down
ENSMUSG00000021499	Catsper3	-3.17E+00	5.84E-49	down	-2.33E-01	7.13E-01	no
ENSMUSG00000059288	Cdyl	-4.11E+00	9.63E-51	down	-1.69E+00	9.02E-04	down
ENSMUSG00000021494	Ddx41	-8.68E-01	8.84E-03	no	-1.11E+00	4.74E-05	down
ENSMUSG00000060176	Kif27	-5.06E+00	5.30E-62	down	-3.35E+00	7.34E-16	down
ENSMUSG00000021610	Clptm1l	-7.55E-01	2.00E-03	no	-1.64E+00	9.85E-17	down
ENSMUSG00000021149	Gtpbp4	-8.56E-01	2.48E-02	no	-1.69E+00	4.07E-07	down
ENSMUSG00000021392	Nol8	-2.12E+00	5.17E-22	down	-2.65E+00	1.85E-50	down
ENSMUSG00000071291	Zfp58	-4.43E+00	2.28E-05	down	-1.94E+00	3.61E-01	no
ENSMUSG00000044934	Zfp367	-2.23E+00	2.34E-03	no	-3.50E+00	2.06E-13	down
ENSMUSG00000021643	Serf1	1.76E+00	5.73E-222	up	1.41E+00	4.81E-224	up
ENSMUSG00000069255	Dusp22	-1.47E+00	9.48E-04	down	-1.73E+00	4.99E-05	down
ENSMUSG00000033799	BC016423	-4.76E+00	2.16E-31	down	-2.20E+00	4.44E-03	no
ENSMUSG00000057531	Dtnbp1	-6.56E-01	2.41E-03	no	-1.71E+00	6.56E-34	down
ENSMUSG00000037966	Ninj1	5.97E+00	8.78E-15	up	3.23E+00	2.42E-04	up
ENSMUSG00000071252	2210408I21Rik	-3.23E+00	1.37E-08	down	-2.50E+00	2.92E-04	down
ENSMUSG00000021411	Pxdc1	-1.50E-01	8.74E-01	no	-3.07E+00	4.00E-07	down

ENSMUSG00000005312	Ubqln1	-1.90E+00	1.77E-11	down	-1.77E+00	5.95E-09	down
ENSMUSG00000021215	Net1	-3.20E+00	2.84E-04	down	-2.93E+00	4.16E-04	down
ENSMUSG00000038025	Phf2	-2.62E+00	1.26E-03	no	-4.17E+00	1.43E-29	down
ENSMUSG00000045410	Akr1e1	-1.87E+00	3.62E-05	down	-1.73E+00	4.63E-07	down
ENSMUSG00000055313	Pgbd1	-3.80E+00	4.72E-02	no	-4.18E+00	3.98E-04	down
ENSMUSG00000037933	Bicd2	-3.04E+00	1.70E-04	down	-3.05E+00	5.20E-04	down
ENSMUSG00000021615	Xrcc4	-1.48E+00	9.35E-03	no	-2.35E+00	3.52E-10	down
ENSMUSG00000014850	Msh3	-2.61E+00	6.79E-04	down	-3.77E+00	5.17E-10	down
ENSMUSG00000058022	Adtrp	-1.51E+00	7.98E-04	down	1.93E+00	4.39E-03	no
ENSMUSG00000021373	Cap2	-5.17E+00	3.69E-21	down	-4.05E+00	1.20E-08	down
ENSMUSG00000041817	Fam169a	-1.10E+00	2.35E-01	no	-2.22E+00	5.27E-04	down
ENSMUSG00000016018	Skiv2l2	-2.94E+00	1.60E-36	down	-2.42E+00	3.06E-17	down
ENSMUSG00000042426	Dhx29	-2.80E+00	2.09E-04	down	-2.86E+00	4.68E-05	down
ENSMUSG00000021686	Ap3b1	-3.35E+00	4.33E-32	down	-2.81E+00	1.10E-16	down
ENSMUSG00000035248	Zcchc6	-2.43E+00	8.66E-04	down	-1.98E+00	3.48E-02	no
ENSMUSG00000042284	Itga1	-4.80E+00	5.09E-08	down	-2.02E+00	2.01E-01	no
ENSMUSG00000021301	Hecw1	-5.28E+00	1.24E-04	down	-3.35E+00	3.69E-01	no
ENSMUSG00000021668	Polk	-4.18E+00	3.70E-15	down	-2.22E+00	3.79E-03	no
ENSMUSG00000042590	Ipo11	-1.68E+00	5.62E-02	no	-2.63E+00	6.54E-05	down
ENSMUSG00000006720	Zfp184	-3.15E+00	2.73E-04	down	-2.16E+00	1.19E-02	no
ENSMUSG00000021585	Cast	-3.52E+00	1.54E-88	down	-8.17E-01	1.53E-02	no
ENSMUSG00000074794	Arrdc3	-3.55E+00	9.84E-07	down	-4.60E+00	2.49E-14	down
ENSMUSG00000021687	Scamp1	-3.04E+00	1.00E-22	down	-1.23E+00	1.33E-02	no
ENSMUSG00000006715	Gmnn	1.20E+00	1.82E-03	no	-1.31E+00	8.71E-12	down
ENSMUSG00000021635	Rad17	-2.32E+00	1.68E-12	down	-1.92E+00	1.19E-06	down
ENSMUSG00000021712	Trim23	-7.89E-01	4.79E-01	no	-3.35E+00	1.40E-06	down
ENSMUSG00000021374	Nup153	-4.09E+00	2.52E-30	down	-3.00E+00	6.69E-10	down
ENSMUSG00000043183	Simc1	-2.68E+00	1.49E-03	no	-3.06E+00	3.39E-04	down
ENSMUSG00000044566	Cage1	-4.08E+00	1.99E-91	down	-3.30E+00	2.45E-56	down
ENSMUSG00000021577	Sdha	-5.63E-01	6.08E-02	no	-1.14E+00	1.77E-07	down
ENSMUSG00000041431	Ccnb1	1.95E+00	2.76E-04	up	5.37E-01	5.58E-01	no
ENSMUSG00000041138	Nme8	-4.60E+00	1.31E-108	down	-3.97E+00	6.11E-66	down
ENSMUSG00000049985	Ankrd55	-1.79E+00	4.56E-02	no	-2.82E+00	1.11E-06	down
ENSMUSG00000035139	Secisbp2	-1.73E+00	3.03E-02	no	-2.39E+00	8.96E-04	down
ENSMUSG00000021363	Mak	-3.15E+00	7.64E-39	down	-1.71E+00	3.14E-08	down
ENSMUSG00000021758	Ddx4	-2.72E+00	5.80E-91	down	-2.69E+00	1.63E-90	down
ENSMUSG00000015937	H2afy	-1.61E-01	8.33E-01	no	-1.33E+00	8.59E-04	down
ENSMUSG00000033102	Cdc14b	-3.56E+00	3.75E-11	down	-1.96E+00	4.98E-02	no
ENSMUSG00000050244	Heatr1	-1.75E+00	4.43E-02	no	-2.74E+00	2.67E-04	down
ENSMUSG00000021500	Ddx46	-1.14E+00	3.83E-05	down	-1.42E+00	6.98E-09	down
ENSMUSG00000021697	Depdc1b	-3.11E+00	2.01E-04	down	-2.08E+00	2.82E-02	no
ENSMUSG00000025871	4833439L19Rik	-3.17E-01	5.60E-01	no	-1.86E+00	9.88E-08	down
ENSMUSG00000032745	Gpbp1	-3.98E+00	7.84E-67	down	-3.85E+00	3.18E-51	down
ENSMUSG00000021519	Mterfd1	-1.93E+00	3.94E-04	down	-3.08E+00	8.29E-12	down
ENSMUSG00000021549	Rasa1	-1.49E+00	1.14E-02	no	-2.22E+00	3.43E-05	down
ENSMUSG00000021669	Col4a3bp	-3.41E+00	3.87E-13	down	-2.50E+00	1.81E-04	down
ENSMUSG00000025873	Faf2	-1.50E+00	2.65E-03	no	-1.68E+00	2.03E-04	down
ENSMUSG00000025453	Nnt	-2.71E+00	7.52E-05	down	-3.02E+00	3.84E-08	down
ENSMUSG00000032727	Mier3	-3.24E+00	1.14E-10	down	-3.38E+00	3.08E-13	down
ENSMUSG00000021357	Exoc2	-2.93E+00	4.03E-07	down	-1.96E+00	2.42E-03	no
ENSMUSG00000021481	Zfp346	-4.26E-01	6.76E-01	no	-2.86E+00	3.84E-10	down
ENSMUSG00000025451	Paip1	-1.17E+00	1.84E-01	no	-4.33E+00	3.03E-22	down
ENSMUSG00000057554	Lgals8	-3.23E+00	9.67E-52	down	-2.06E+00	1.72E-13	down
ENSMUSG00000009470	Tnpo1	-2.76E+00	7.06E-06	down	-3.18E+00	2.19E-06	down
ENSMUSG00000021595	Nsun2	-2.66E+00	1.00E-20	down	-2.30E+00	6.09E-15	down
ENSMUSG00000034675	Dbn1	-2.70E+00	6.96E-07	down	-9.34E-01	3.66E-01	no
ENSMUSG00000021670	Hmgcr	-2.76E+00	1.20E-07	down	-2.52E+00	4.38E-06	down
ENSMUSG00000060147	Serpinb6a	-1.39E+00	4.83E-05	down	-1.86E+00	6.12E-13	down
ENSMUSG00000006191	Cdkal1	-3.18E+00	2.79E-04	down	-2.46E+00	2.42E-02	no
ENSMUSG00000034928	Rnf44	-2.34E+00	1.28E-07	down	-1.65E+00	6.41E-03	no
ENSMUSG00000021555	Naa35	2.01E-01	7.61E-01	no	-1.45E+00	2.82E-04	down
ENSMUSG00000021693	Kif2a	-3.69E+00	5.27E-30	down	-3.47E+00	2.56E-26	down
ENSMUSG00000021196	Pfkp	-2.64E+00	1.71E-09	down	-2.70E+00	4.46E-12	down
ENSMUSG00000021338	Lrrc16a	-3.81E+00	3.52E-07	down	-9.87E-01	5.90E-01	no

ENSMUSG00000021546	Hnrnpk	1.14E-01	7.83E-01	no	-1.63E+00	9.30E-11	down
ENSMUSG00000021557	Agtpbp1	-3.72E+00	1.96E-14	down	-3.24E+00	2.27E-10	down
ENSMUSG00000021986	Amer2	-4.82E+00	1.81E-25	down	-2.33E+00	1.20E-02	no
ENSMUSG00000043881	Kbtbd7	-2.64E+00	2.36E-02	no	-4.05E+00	6.58E-07	down
ENSMUSG00000075502	Zbtbd6	-1.44E+00	2.82E-02	no	-2.60E+00	1.46E-11	down
ENSMUSG00000062611	Rps3a2	-5.54E-01	3.75E-02	no	-2.69E+00	1.13E-38	down
ENSMUSG00000060143	Gm10076	3.92E+00	0.00E+00	up	2.88E+00	0.00E+00	up
ENSMUSG00000071229	Timm8a2	-1.11E+00	6.29E-02	no	-2.13E+00	9.85E-14	down
ENSMUSG00000045107	Saysd1	6.47E-01	2.04E-08	no	1.49E+00	4.53E-30	up
ENSMUSG00000078127	Fam170b	-1.35E+00	1.83E-19	down	1.28E+00	5.97E-07	up
ENSMUSG00000040822	1700123O20Rik	-1.43E+00	1.84E-02	no	-1.73E+00	3.76E-05	down
ENSMUSG00000022215	Fitm1	9.00E-01	4.34E-02	no	1.74E+00	6.38E-05	up
ENSMUSG00000021967	Mrp63	3.17E+00	1.52E-59	up	2.92E+00	1.12E-60	up
ENSMUSG00000053868	Gm5142	-1.02E+00	3.78E-43	down	1.68E-01	1.43E-01	no
ENSMUSG00000043157	Arl11	-4.03E+00	6.04E-09	down	-1.71E+00	3.13E-01	no
ENSMUSG00000075573	Defb47	8.39E+00	1.29E-18	up	7.05E+00	8.55E-32	up
ENSMUSG00000044186	Nkx2-6	-3.11E-01	3.74E-01	no	2.02E+00	5.75E-06	up
ENSMUSG00000033730	Egr3	-6.15E+00	1.11E-10	down	-5.67E+00	5.18E-02	no
ENSMUSG00000034689	4921530L21Rik	-4.44E+00	5.59E-58	down	-2.66E+00	2.33E-07	down
ENSMUSG00000042888	1700110M21Rik	-3.76E+00	1.82E-155	down	-9.85E-01	1.15E-04	no
ENSMUSG00000022116	4930449E01Rik	-2.23E+00	9.41E-08	down	-6.53E-01	4.95E-01	no
ENSMUSG00000049960	Mrps16	2.69E+00	4.77E-17	up	4.79E-01	4.69E-02	no
ENSMUSG00000063787	Chchd1	4.10E-01	8.40E-02	no	-1.52E+00	8.77E-29	down
ENSMUSG00000042485	Mustn1	5.86E+00	7.71E-07	up	4.78E+00	1.13E-01	no
ENSMUSG00000072473	1700024G13Rik	-1.49E+00	3.49E-24	down	2.27E-01	5.60E-01	no
ENSMUSG00000041068	4930596D02Rik	-3.59E+00	1.62E-40	down	-1.03E-01	9.04E-01	no
ENSMUSG00000072616	Gm7853	-1.31E+00	6.29E-04	down	2.96E+00	2.24E-06	up
ENSMUSG00000096405	4930474N05Rik	-2.50E+00	1.46E-08	down	-1.09E+00	3.23E-01	no
ENSMUSG00000096001	2610528A11Rik	-2.40E+00	2.01E-16	down	1.06E-01	9.10E-01	no
ENSMUSG00000022193	Psmb5	2.34E+00	3.17E-33	up	1.12E+00	4.35E-12	up
ENSMUSG00000045211	Nudt18	-1.08E+00	1.85E-10	down	-9.88E-01	7.09E-11	no
ENSMUSG00000052584	Serp2	-1.89E+00	5.57E-06	down	-4.27E-01	7.05E-01	no
ENSMUSG00000063821	Dupd1	1.05E-01	9.12E-01	no	-1.69E+00	5.29E-04	down
ENSMUSG00000021791	Dydc2	-7.08E+00	1.99E-34	down	-6.70E+00	2.31E-09	down
ENSMUSG00000072595	4930503E14Rik	3.15E-01	7.39E-01	no	-1.68E+00	5.83E-04	down
ENSMUSG00000021876	Rnase4	3.65E+00	4.30E-06	up	3.01E+00	1.17E-01	no
ENSMUSG00000021928	Ebpl	5.15E+00	4.26E-04	up	2.85E+00	9.95E-02	no
ENSMUSG00000021961	4930578I06Rik	-2.39E+00	2.30E-85	down	1.97E-01	5.25E-01	no
ENSMUSG00000034248	Slc25a37	-4.11E+00	3.40E-04	down	-2.35E+00	3.22E-01	no
ENSMUSG00000005148	Klf5	-2.00E+00	9.11E-08	down	8.56E-01	3.41E-01	no
ENSMUSG00000023243	Kcnk5	-3.26E+00	2.44E-04	down	-4.69E-01	8.54E-01	no
ENSMUSG00000021811	Dnajc9	-1.55E+00	1.21E-11	down	-6.01E-01	1.45E-01	no
ENSMUSG00000072624	Gm5460	-6.10E+00	5.78E-08	down	-4.17E+00	8.86E-02	no
ENSMUSG00000095493	A630023A22Rik	-2.96E+00	1.70E-07	down	-5.23E-01	7.45E-01	no
ENSMUSG00000090505	Gm3486	1.02E+00	2.29E-05	up	2.30E+00	3.93E-07	up
ENSMUSG00000095384	1700001F09Rik	5.76E-02	8.32E-01	no	1.57E+00	8.84E-05	up
ENSMUSG00000072145	BC061237	1.78E+00	2.96E-38	up	3.23E+00	5.82E-34	up
ENSMUSG00000091296	Gm4181	1.05E+00	2.37E-02	no	3.71E+00	8.62E-04	up
ENSMUSG00000022174	Dad1	2.56E+00	1.97E-60	up	2.81E+00	7.34E-54	up
ENSMUSG00000010406	Mrpl52	2.89E+00	6.39E-83	up	1.46E+00	7.36E-53	up
ENSMUSG00000021966	Prss52	-4.05E+00	1.04E-35	down	-2.53E+00	9.97E-05	down
ENSMUSG00000022002	4930564B18Rik	-3.87E+00	2.64E-07	down	-2.70E-01	9.15E-01	no
ENSMUSG00000095280	Gm21738	1.02E+01	1.25E-122	up	4.50E+00	3.56E-04	up
ENSMUSG00000058351	Smim4	3.14E+00	5.31E-06	up	2.23E+00	2.25E-04	up
ENSMUSG00000021891	Mettl6	-2.02E+00	1.18E-07	down	-2.48E+00	6.00E-15	down
ENSMUSG00000037722	Gnpnat1	-3.57E-01	7.17E-01	no	-2.22E+00	1.16E-04	down
ENSMUSG00000021846	Peli2	-2.30E+00	7.93E-13	down	-1.84E+00	1.49E-08	down
ENSMUSG00000015443	Gzmn	-3.61E+00	5.10E-21	down	-1.06E+00	1.78E-01	no
ENSMUSG00000035235	Trim13	-3.07E+00	4.02E-14	down	-3.46E+00	3.14E-30	down
ENSMUSG00000035095	Fam167a	-4.83E+00	1.35E-11	down	-2.44E+00	1.19E-01	no
ENSMUSG00000071591	Gm10339	-1.54E+00	2.23E-02	no	-2.06E+00	2.97E-05	down
ENSMUSG00000022013	Dnajc15	1.17E+00	4.35E-107	up	1.42E+00	5.87E-179	up
ENSMUSG00000093945	Gm3543	2.42E+00	7.54E-05	up	2.98E+00	1.49E-02	no
ENSMUSG00000008813	Tppp2	-5.50E+00	2.42E-126	down	-4.19E+00	2.87E-31	down

ENSMUSG00000007591	Tssk4	-4.26E+00	2.62E-81	down	-1.16E+00	6.21E-03	no
ENSMUSG00000021958	Pinx1	-8.23E-01	3.71E-01	no	-2.00E+00	3.94E-04	down
ENSMUSG00000022085	Pebp4	-2.56E+00	5.95E-134	down	-2.24E+00	4.75E-149	down
ENSMUSG00000022109	Med4	1.68E+00	9.14E-09	up	-3.61E-01	2.13E-01	no
ENSMUSG00000053453	Thoc7	2.26E-01	2.51E-01	no	-1.21E+00	3.48E-35	down
ENSMUSG00000021807	2700060E02Rik	-1.12E+00	4.53E-06	down	-1.83E+00	3.85E-22	down
ENSMUSG00000057156	Homez	-2.77E+00	1.41E-10	down	-1.37E+00	2.06E-02	no
ENSMUSG00000022210	Dhrs4	-2.75E+00	8.10E-09	down	-3.13E+00	2.87E-11	down
ENSMUSG00000021273	Fdft1	-2.78E+00	4.76E-103	down	-1.81E+00	2.70E-35	down
ENSMUSG00000033589	Reep4	-9.84E-01	3.22E-05	no	-1.15E+00	2.03E-08	down
ENSMUSG00000022111	Uchl3	-8.87E-01	1.07E-12	no	-1.33E+00	5.77E-38	down
ENSMUSG00000041765	Ubac2	-7.90E-01	6.84E-03	no	-1.27E+00	2.15E-07	down
ENSMUSG00000042682	Selk	1.11E+00	9.70E-08	up	5.10E-01	8.69E-02	no
ENSMUSG00000079267	Gm5930	1.96E+00	2.24E-01	no	-2.14E+00	1.37E-05	down
ENSMUSG00000092165	Gm5624	1.32E+00	3.03E-01	no	-1.86E+00	1.19E-04	down
ENSMUSG00000022199	Slc22a17	4.68E+00	4.83E-05	up	3.93E+00	1.10E-04	up
ENSMUSG00000010376	Nedd8	2.28E+00	3.33E-113	up	1.18E+00	3.66E-48	up
ENSMUSG00000007589	Tinf2	-4.85E-01	2.34E-01	no	-1.52E+00	6.62E-07	down
ENSMUSG00000090643	Gm3453	1.55E+00	1.82E-03	no	3.03E+00	4.47E-04	up
ENSMUSG00000021930	Spryd7	-3.38E-01	5.97E-01	no	-1.53E+00	6.83E-04	down
ENSMUSG00000022089	Bin3	-1.82E+00	7.62E-08	down	-1.82E+00	2.09E-14	down
ENSMUSG00000094021	Gm3685	2.84E+00	2.84E-04	up	4.42E+00	5.41E-04	up
ENSMUSG00000047911	Npm2	-3.06E+00	2.99E-04	down	-2.61E+00	1.76E-03	no
ENSMUSG00000022103	Gfra2	-4.10E+00	1.31E-08	down	-3.10E+00	5.04E-03	no
ENSMUSG00000025290	Rps24	-1.12E+00	9.18E-27	down	-2.32E+00	1.00E-156	down
ENSMUSG00000002326	Gmpr2	-1.13E+00	4.37E-04	down	-5.92E-01	8.56E-02	no
ENSMUSG00000019297	Nop9	-1.99E+00	6.57E-18	down	-1.81E+00	2.08E-21	down
ENSMUSG00000079371	Gm3476	2.03E+00	6.45E-04	up	3.30E+00	3.74E-03	no
ENSMUSG00000075571	Defb30	-3.32E+00	2.39E-04	down	-3.42E+00	3.44E-02	no
ENSMUSG00000021939	Ctsb	4.04E+00	1.30E-14	up	3.39E+00	6.44E-06	up
ENSMUSG00000035078	Mtmr9	-2.76E+00	2.02E-18	down	-2.57E+00	2.70E-17	down
ENSMUSG00000022052	Ppp2r2a	-1.39E+00	4.28E-03	no	-2.27E+00	4.62E-07	down
ENSMUSG00000090404	Gm8362	1.64E+00	2.33E-04	up	3.27E+00	4.39E-05	up
ENSMUSG00000022075	Rhobtb2	-4.99E+00	2.35E-05	down	-2.21E+00	4.31E-01	no
ENSMUSG00000021997	Lrrc63	-4.53E+00	1.66E-48	down	-4.10E+00	2.19E-53	down
ENSMUSG00000034913	Spert	-3.70E+00	1.02E-214	down	-1.42E+00	1.86E-11	down
ENSMUSG00000022003	Slc25a30	-2.80E+00	9.85E-05	down	-4.89E-01	8.00E-01	no
ENSMUSG00000022023	Wbp4	-3.22E-01	4.47E-01	no	-1.55E+00	1.77E-11	down
ENSMUSG00000021749	Oit1	-3.08E+00	9.75E-48	down	-1.90E+00	8.63E-19	down
ENSMUSG00000033111	3830406C13Rik	-1.95E+00	1.61E-07	down	-2.41E+00	2.36E-16	down
ENSMUSG00000039197	Adk	2.55E+00	1.05E-05	up	1.05E+00	1.35E-01	no
ENSMUSG00000021902	Phf7	-1.63E+00	0.00E+00	down	-1.22E+00	1.37E-234	down
ENSMUSG00000049295	Zfp219	-3.36E+00	3.95E-07	down	-1.81E+00	2.29E-01	no
ENSMUSG00000021981	Cab39l	-2.87E+00	4.12E-23	down	-2.39E+00	5.37E-13	down
ENSMUSG00000022136	Dnajc3	4.92E-01	3.36E-01	no	-1.72E+00	2.13E-06	down
ENSMUSG00000068758	Il3ra	-1.52E+00	1.26E-04	down	-2.05E+00	8.04E-11	down
ENSMUSG00000021940	Ptpn20	-3.14E+00	2.19E-14	down	-4.30E+00	7.82E-56	down
ENSMUSG00000034450	Gulo	6.37E+00	5.31E-10	up	7.97E+00	8.15E-12	up
ENSMUSG00000022070	Bora	-2.19E+00	1.92E-07	down	-2.63E+00	2.23E-10	down
ENSMUSG00000039308	Ndst2	-2.50E+00	2.15E-04	down	-1.92E+00	1.69E-03	no
ENSMUSG00000015971	Actr8	-1.64E+00	6.73E-07	down	-1.86E+00	2.64E-11	down
ENSMUSG00000071547	Nt5dc2	2.18E+00	1.32E-06	up	1.57E+00	2.48E-02	no
ENSMUSG00000022024	Sugt1	-2.06E+00	1.46E-22	down	-1.98E+00	4.01E-23	down
ENSMUSG00000021832	Psmc6	-7.08E-01	1.27E-02	no	-2.15E+00	2.29E-18	down
ENSMUSG00000040997	Abhd4	-3.18E+00	7.66E-49	down	-1.41E+00	1.64E-06	down
ENSMUSG00000021987	Mtmr6	-3.16E+00	2.18E-38	down	-3.16E+00	1.04E-44	down
ENSMUSG00000052099	Prss51	-4.73E+00	3.66E-20	down	-2.80E+00	7.57E-03	no
ENSMUSG00000022092	Ppp3cc	-2.51E+00	1.17E-56	down	-2.12E+00	5.43E-44	down
ENSMUSG00000041625	Ggact	-2.98E+00	1.06E-05	down	-1.04E+00	4.80E-01	no
ENSMUSG00000021918	Nek4	-3.47E+00	1.26E-21	down	-3.40E+00	2.88E-24	down
ENSMUSG00000042354	Gnl3	2.14E+00	2.01E-12	up	2.65E-01	5.18E-01	no
ENSMUSG00000021790	Dydc1	-1.96E+00	1.46E-23	down	-1.36E+00	2.09E-10	down
ENSMUSG00000055128	Cgrrf1	-9.73E-01	1.27E-02	no	-1.35E+00	2.73E-06	down
ENSMUSG00000063895	Nupl1	-3.33E+00	4.52E-27	down	-3.01E+00	5.65E-20	down

ENSMUSG00000034623	Prss55	-4.75E+00	4.96E-53	down	-3.22E+00	1.43E-08	down
ENSMUSG00000022031	Elp3	-1.96E+00	3.64E-04	down	-2.21E+00	1.85E-06	down
ENSMUSG00000021737	Psmd6	-5.51E-01	8.22E-02	no	-1.25E+00	1.33E-08	down
ENSMUSG00000022033	Pbk	-4.93E-01	6.88E-02	no	-2.17E+00	2.85E-62	down
ENSMUSG00000021901	Bap1	-2.22E+00	3.38E-18	down	-1.46E+00	4.60E-07	down
ENSMUSG00000047441	Antxrl	-2.47E+00	5.37E-33	down	7.85E-01	6.60E-02	no
ENSMUSG00000060373	Hnrnpc	2.90E-01	4.60E-01	no	-1.63E+00	9.03E-12	down
ENSMUSG00000064128	Cenpj	-2.85E+00	3.89E-04	down	-3.17E+00	2.50E-05	down
ENSMUSG00000021975	Ints9	-1.88E+00	2.37E-03	no	-2.03E+00	2.10E-04	down
ENSMUSG00000046753	Ccdc66	-2.62E+00	6.65E-08	down	-2.01E+00	8.25E-06	down
ENSMUSG00000041028	Ghitm	-1.69E+00	1.78E-19	down	-1.14E+00	3.49E-07	down
ENSMUSG00000021850	1700011H14Rik	-3.02E+00	2.46E-45	down	-5.63E-01	2.15E-01	no
ENSMUSG00000022064	Pibf1	-2.22E+00	1.04E-05	down	-2.81E+00	1.01E-09	down
ENSMUSG00000021785	Ngly1	-3.54E+00	3.50E-43	down	-2.11E+00	1.71E-10	down
ENSMUSG00000015970	Chdh	-4.09E+00	2.82E-03	no	-5.35E+00	6.02E-09	down
ENSMUSG00000021977	1700129C05Rik	-2.63E+00	3.78E-146	down	-1.49E+00	2.33E-27	down
ENSMUSG00000021993	Mipep	-3.78E+00	2.04E-70	down	-1.97E+00	1.01E-11	down
ENSMUSG00000035161	Ints6	-3.36E+00	3.61E-22	down	-3.09E+00	1.79E-15	down
ENSMUSG00000021770	Samd8	-1.76E+00	5.85E-05	down	-1.44E+00	2.79E-03	no
ENSMUSG00000021916	Glt8d1	-2.10E+00	2.12E-37	down	-2.44E+00	5.32E-67	down
ENSMUSG00000037536	Fbxo34	-2.27E+00	6.37E-07	down	-1.25E+00	3.46E-02	no
ENSMUSG00000022014	Epsti1	-3.00E+00	1.59E-07	down	-2.86E+00	2.20E-06	down
ENSMUSG00000062014	Gmfb	-8.17E-01	7.37E-03	no	-1.93E+00	1.08E-18	down
ENSMUSG00000022039	Adam2	-2.50E+00	1.10E-77	down	-2.52E+00	1.36E-123	down
ENSMUSG00000033712	2610301G19Rik	-2.16E+00	6.03E-05	down	-3.84E+00	6.03E-27	down
ENSMUSG00000022091	Sorbs3	-3.45E+00	6.13E-27	down	-1.21E+00	5.97E-02	no
ENSMUSG00000022025	Lect1	-3.81E+00	1.38E-01	no	-5.78E+00	6.46E-04	down
ENSMUSG00000033166	Dis3	-3.68E+00	4.67E-21	down	-1.93E+00	4.99E-03	no
ENSMUSG00000002332	Dhrs1	-1.86E+00	5.36E-18	down	-1.11E+00	3.04E-08	down
ENSMUSG00000034194	R3hcc1	-3.56E+00	2.49E-29	down	-1.31E+00	5.26E-03	no
ENSMUSG00000039367	Sec24c	-2.41E+00	2.62E-07	down	-2.87E+00	3.48E-14	down
ENSMUSG00000016831	Tox4	-1.26E+00	4.52E-02	no	-2.80E+00	2.14E-08	down
ENSMUSG00000033644	Piwil2	-5.00E-01	3.26E-01	no	-2.20E+00	1.83E-16	down
ENSMUSG00000034893	Cog3	-6.05E-01	5.43E-01	no	-1.96E+00	7.29E-04	down
ENSMUSG00000040651	D14Abb1e	-1.56E+00	9.22E-02	no	-3.10E+00	1.11E-05	down
ENSMUSG00000041673	Lrrc18	-3.42E+00	1.80E-111	down	-1.02E+00	4.31E-04	down
ENSMUSG00000021945	Zmym2	-2.49E+00	1.05E-03	no	-3.76E+00	6.96E-12	down
ENSMUSG00000021733	Slc4a7	-2.63E+00	7.34E-05	down	-1.56E+00	1.48E-01	no
ENSMUSG00000050335	Lgals3	3.82E+00	1.16E-06	up	1.88E+00	1.85E-01	no
ENSMUSG00000021938	Pspc1	-7.09E-01	2.82E-01	no	-3.11E+00	4.87E-18	down
ENSMUSG00000030662	Ipo5	-4.61E+00	2.64E-194	down	-3.35E+00	8.59E-81	down
ENSMUSG00000021814	Anxa7	-1.05E+00	2.17E-01	no	-2.84E+00	1.32E-05	down
ENSMUSG00000021819	Zswim8	-2.09E+00	1.07E-03	no	-3.30E+00	1.79E-09	down
ENSMUSG00000035726	Supt16	-3.91E-01	5.27E-01	no	-2.04E+00	3.09E-07	down
ENSMUSG00000021957	Tkt	3.20E+00	5.72E-03	no	2.89E+00	5.96E-04	up
ENSMUSG00000002324	Rec8	-4.68E+00	3.39E-36	down	-1.38E+00	4.04E-02	no
ENSMUSG00000079184	Mphosph8	1.97E+00	7.25E-11	up	-4.08E-01	4.25E-02	no
ENSMUSG00000014496	Ankrd28	-3.56E+00	1.97E-08	down	-2.17E+00	2.19E-02	no
ENSMUSG00000034731	Dgkh	-4.78E+00	6.04E-35	down	-1.68E+00	6.52E-03	no
ENSMUSG00000021768	Dusp13	-2.46E+00	3.09E-48	down	8.43E-02	8.73E-01	no
ENSMUSG00000025544	Tm9sf2	-2.43E+00	9.22E-26	down	-2.10E+00	2.88E-13	down
ENSMUSG00000038925	E330034G19Rik	-2.19E+00	9.17E-06	down	1.81E-01	9.19E-01	no
ENSMUSG00000022110	Sucla2	-2.14E+00	3.86E-60	down	-1.56E+00	9.66E-30	down
ENSMUSG00000000365	Rnf17	-2.62E+00	1.62E-22	down	-2.20E+00	2.80E-15	down
ENSMUSG00000033885	Pxk	-3.93E+00	2.88E-12	down	-2.31E+00	1.05E-03	no
ENSMUSG00000061356	Nuggc	-2.09E+00	1.07E-04	down	-2.15E+00	7.56E-08	down
ENSMUSG00000021982	Cdadc1	-8.18E-01	2.01E-01	no	-1.94E+00	4.13E-05	down
ENSMUSG00000042156	Dzip1	-3.96E+00	2.82E-51	down	-2.47E+00	3.43E-14	down
ENSMUSG00000022037	Clu	2.43E+00	5.00E-12	up	1.62E+00	3.81E-19	up
ENSMUSG00000039599	Fam149b	-2.77E+00	1.39E-10	down	-3.02E+00	3.80E-11	down
ENSMUSG00000021792	Fam213a	-2.19E-01	6.66E-01	no	-1.37E+00	7.06E-08	down
ENSMUSG00000021959	Lats2	-4.09E+00	6.09E-14	down	-2.16E+00	4.88E-02	no
ENSMUSG00000022010	Tsc22d1	-1.12E+00	3.17E-03	no	-1.44E+00	1.61E-06	down
ENSMUSG00000021771	Vdac2	-1.01E+00	1.08E-11	down	-9.52E-01	1.80E-12	no

ENSMUSG00000022194	Pabpn1	-2.39E+00	1.74E-07	down	-1.87E+00	2.60E-04	down
ENSMUSG00000022019	Tdrd3	-3.34E+00	1.41E-05	down	-3.36E+00	2.29E-07	down
ENSMUSG00000022021	Diap3	-2.42E+00	3.18E-06	down	-6.16E-01	6.25E-01	no
ENSMUSG00000056234	Ncoa4	-2.84E+00	9.96E-05	down	-2.38E+00	2.13E-03	no
ENSMUSG00000021983	Atp8a2	-2.41E+00	2.57E-06	down	1.32E+00	1.18E-01	no
ENSMUSG00000034327	Kctd9	-2.30E+00	5.62E-06	down	-2.98E+00	2.37E-11	down
ENSMUSG00000021820	Camk2g	-1.20E+00	3.08E-01	no	-2.87E+00	5.40E-05	down
ENSMUSG00000021838	Samd4	-3.55E+00	1.22E-30	down	-9.18E-01	1.82E-01	no
ENSMUSG00000040040	Ift88	-1.27E+00	5.26E-04	down	-2.06E-01	6.72E-01	no
ENSMUSG00000058690	Ccser2	-2.36E+00	1.09E-08	down	-6.69E-01	4.32E-01	no
ENSMUSG00000048922	Cdca2	-2.98E+00	2.33E-23	down	-1.49E+00	7.00E-04	down
ENSMUSG00000021879	Dnahc12	-3.96E+00	3.78E-07	down	-4.12E+00	1.21E-12	down
ENSMUSG00000019027	Dnahc1	-2.99E+00	1.25E-07	down	-3.05E+00	1.34E-13	down
ENSMUSG00000033487	Fndc3a	-2.92E+00	1.25E-14	down	-2.81E+00	1.08E-10	down
ENSMUSG00000022216	Psme1	4.17E+00	1.06E-06	up	3.48E+00	1.44E-03	no
ENSMUSG00000022119	Rbm26	-2.28E+00	1.45E-05	down	-2.61E+00	1.58E-06	down
ENSMUSG00000041408	Wapal	-2.84E+00	1.05E-15	down	-2.89E+00	1.58E-15	down
ENSMUSG00000021911	Parg	-7.65E-01	5.64E-01	no	-3.14E+00	2.17E-05	down
ENSMUSG00000006527	Sfmbt1	-2.14E+00	9.44E-07	down	-2.24E+00	1.61E-09	down
ENSMUSG00000037697	Ddhd1	-4.52E+00	1.31E-43	down	-3.10E+00	4.12E-14	down
ENSMUSG00000039543	Ttc18	-3.51E+00	8.16E-30	down	-3.52E+00	4.33E-51	down
ENSMUSG00000022214	Dcaf11	-2.68E-01	8.04E-01	no	-2.19E+00	4.92E-04	down
ENSMUSG00000002319	Ipo4	-2.66E+00	3.72E-32	down	-1.88E+00	2.06E-14	down
ENSMUSG00000021948	Prkcd	-4.03E+00	3.51E-20	down	-2.77E+00	5.27E-03	no
ENSMUSG00000042286	Stab1	5.31E+00	3.35E-05	up	6.01E+00	8.55E-08	up
ENSMUSG00000021870	Slmap	-9.17E-01	3.19E-01	no	-2.36E+00	1.35E-04	down
ENSMUSG00000057132	Rpgrip1	-5.19E+00	4.47E-17	down	-4.98E+00	3.29E-14	down
ENSMUSG00000022185	Acin1	-3.06E+00	8.48E-14	down	-3.24E+00	4.28E-18	down
ENSMUSG00000022106	Rcbtb2	-2.96E+00	2.11E-04	down	-3.03E+00	3.46E-04	down
ENSMUSG00000004562	Arhgef40	5.39E+00	1.31E-04	up	4.70E+00	9.29E-03	no
ENSMUSG00000047565	Acot10	-3.83E+00	2.57E-25	down	-1.09E+00	1.78E-01	no
ENSMUSG00000051198	4930548G14Rik	-3.64E+00	5.10E-10	down	-1.68E+00	9.31E-02	no
ENSMUSG00000071749	4933412E24Rik	-2.00E+00	1.07E-02	no	-3.34E+00	2.48E-18	down
ENSMUSG00000096173	Gm3150	2.15E+00	2.01E-205	up	1.79E+00	6.93E-151	up
ENSMUSG00000044276	4933427E11Rik	-1.93E+00	1.90E-15	down	-2.27E+00	2.15E-38	down
ENSMUSG00000096210	H1f0	3.48E+00	4.64E-07	up	8.39E-01	2.87E-01	no
ENSMUSG00000047108	Dnajb7	-4.31E+00	9.88E-121	down	-2.19E+00	9.10E-11	down
ENSMUSG00000055745	Ldoc1l	-3.58E+00	7.69E-08	down	-2.80E+00	1.34E-05	down
ENSMUSG00000052496	Pkdrej	-3.19E+00	2.08E-08	down	-3.01E+00	2.97E-07	down
ENSMUSG00000054117	Zdhhc25	-2.26E+00	2.27E-18	down	5.95E-01	2.77E-01	no
ENSMUSG00000029875	AI836003	-2.27E+00	2.60E-09	down	-5.14E-01	5.48E-01	no
ENSMUSG00000048077	H1fnt	-3.05E+00	1.21E-105	down	-2.64E-01	4.05E-01	no
ENSMUSG00000058057	Mettl7a3	4.39E+00	5.91E-04	up	3.14E+00	7.56E-02	no
ENSMUSG00000061923	Odf1	-4.17E+00	0.00E+00	down	-1.81E+00	1.54E-39	down
ENSMUSG00000068522	Aard	2.53E+00	4.93E-05	up	8.46E-01	4.11E-02	no
ENSMUSG00000037075	Rnf139	-4.62E+00	1.81E-103	down	-3.02E+00	1.78E-24	down
ENSMUSG00000056665	Them6	-2.34E+00	1.67E-07	down	-3.53E+00	2.29E-48	down
ENSMUSG00000043460	Elfn2	1.95E+00	2.54E-06	up	2.37E+00	3.78E-17	up
ENSMUSG00000075524	4930407I10Rik	-6.52E+00	2.35E-55	down	-4.01E+00	5.31E-06	down
ENSMUSG00000023020	Cox14	5.27E+00	7.56E-41	up	2.50E+00	1.21E-22	up
ENSMUSG00000037204	9430023L20Rik	3.23E+00	1.65E-16	up	2.05E+00	1.09E-08	up
ENSMUSG00000075408	6030408B16Rik	6.00E+00	5.11E-06	up	3.46E+00	3.46E-02	no
ENSMUSG00000022598	Psca	-2.09E+00	2.39E-02	no	-2.79E+00	4.21E-04	down
ENSMUSG00000063522	2010109I03Rik	-3.53E+00	1.61E-09	down	-5.70E-01	7.15E-01	no
ENSMUSG00000075602	Ly6a	4.40E+00	1.15E-16	up	3.03E+00	4.30E-03	no
ENSMUSG00000042406	Atf4	2.25E+00	2.29E-19	up	-9.56E-01	3.13E-06	no
ENSMUSG00000022450	Ndufa6	1.81E+00	2.82E-101	up	1.35E+00	6.96E-82	up
ENSMUSG00000023046	Igfbp6	7.06E+00	0.00E+00	up	4.31E+00	2.47E-23	up
ENSMUSG00000022200	Golph3	-1.49E+00	1.76E-04	down	-1.75E+00	6.21E-04	down
ENSMUSG00000022330	Osr2	4.07E+00	1.27E-07	up	3.01E+00	3.59E-02	no
ENSMUSG00000062397	Zfp706	2.27E+00	5.97E-05	up	3.32E-01	6.55E-01	no
ENSMUSG00000038622	Med30	-4.82E-01	3.66E-01	no	-1.48E+00	7.02E-05	down
ENSMUSG00000022354	Ndufb9	3.73E+00	0.00E+00	up	2.16E+00	6.52E-295	up
ENSMUSG00000086361	Gm6569	9.60E-01	8.12E-06	no	2.91E+00	9.95E-18	up

ENSMUSG00000068600	Hemt1	3.01E+00	1.95E-84	up	1.77E+00	4.89E-43	up
ENSMUSG00000022579	Gpihbp1	7.23E+00	5.54E-11	up	7.94E+00	3.39E-08	up
ENSMUSG00000022526	Zfp251	-1.14E+00	4.16E-01	no	-2.57E+00	2.33E-04	down
ENSMUSG00000068220	Lgals1	9.78E-01	5.86E-06	no	-1.46E+00	4.56E-08	down
ENSMUSG00000022426	Josd1	-2.21E+00	1.10E-05	down	-2.49E+00	2.01E-08	down
ENSMUSG00000051518	Rps19bp1	3.11E+00	3.56E-15	up	5.99E-01	1.97E-02	no
ENSMUSG00000048755	Mcat	-8.50E-01	3.81E-03	no	-1.17E+00	2.27E-09	down
ENSMUSG00000041736	Tspo	7.82E+00	1.81E-29	up	5.32E+00	1.68E-08	up
ENSMUSG00000022993	4930415O20Rik	-3.66E+00	1.13E-93	down	-1.62E+00	3.54E-07	down
ENSMUSG00000044636	Csrnp2	-1.44E+00	5.10E-02	no	-2.44E+00	2.61E-05	down
ENSMUSG00000063971	1700011A15Rik	-9.36E-01	1.66E-13	no	1.62E+00	1.33E-17	up
ENSMUSG00000022236	Ropn1l	1.76E+00	0.00E+00	up	1.72E+00	0.00E+00	up
ENSMUSG00000048230	Fbxo43	-2.68E+00	2.97E-02	no	-4.46E+00	2.40E-16	down
ENSMUSG00000022338	Eny2	2.21E+00	1.00E-05	up	-3.24E-01	5.24E-01	no
ENSMUSG00000049653	Spatc1	-4.91E+00	7.72E-45	down	-3.51E+00	5.41E-08	down
ENSMUSG00000034259	Exosc4	1.58E+00	3.50E-04	up	1.44E+00	3.78E-04	up
ENSMUSG00000048385	Scrt1	-4.69E+00	2.98E-04	down	-4.26E+00	8.33E-02	no
ENSMUSG00000063236	1110038F14Rik	3.24E+00	4.47E-07	up	-8.14E-02	9.02E-01	no
ENSMUSG00000033020	Polr2f	2.69E+00	1.70E-48	up	2.14E+00	9.19E-34	up
ENSMUSG00000022400	Rbx1	3.90E+00	1.78E-34	up	1.65E+00	4.56E-15	up
ENSMUSG00000047394	Odf3b	-6.58E+00	1.99E-07	down	-3.77E+00	5.54E-02	no
ENSMUSG00000022622	Acr	-1.57E+00	1.14E-37	down	-3.77E-01	1.10E-02	no
ENSMUSG00000039676	Capsl	-2.33E+00	5.30E-06	down	-2.48E+00	2.49E-07	down
ENSMUSG00000022602	Arc	-2.76E+00	1.25E-44	down	-2.25E+00	1.41E-24	down
ENSMUSG00000022554	Fam203a	-1.46E+00	2.06E-03	no	-1.97E+00	1.32E-07	down
ENSMUSG00000003970	Rpl8	-2.14E-01	1.30E-02	no	-1.25E+00	6.09E-90	down
ENSMUSG00000050439	Enthd1	-2.31E+00	1.61E-10	down	-2.12E+00	4.95E-10	down
ENSMUSG00000022455	Wbp2nl	-4.10E+00	2.80E-31	down	-2.44E+00	3.97E-04	down
ENSMUSG00000078938	Syce3	1.10E+00	2.04E-23	up	9.37E-01	4.15E-15	no
ENSMUSG00000023004	Tuba1b	3.27E+00	3.66E-17	up	1.29E+00	2.65E-04	up
ENSMUSG00000053508	BC048502	-2.06E+00	7.69E-76	down	-1.28E+00	3.64E-16	down
ENSMUSG00000060311	Mucl1	-3.00E+00	4.74E-08	down	6.88E-02	9.65E-01	no
ENSMUSG00000022339	Ebag9	-1.30E+00	2.33E-02	no	-2.30E+00	5.11E-09	down
ENSMUSG00000044678	Ly6k	3.11E+00	8.35E-214	up	1.65E+00	5.59E-95	up
ENSMUSG00000068349	Gml	3.03E+00	6.53E-113	up	1.57E+00	1.77E-47	up
ENSMUSG00000022564	Grina	-1.06E+00	1.96E-23	down	-7.12E-01	1.01E-11	no
ENSMUSG00000022551	Cyc1	3.03E+00	7.28E-38	up	1.38E+00	3.00E-13	up
ENSMUSG00000016637	Ift27	-6.91E-01	4.88E-02	no	-1.06E+00	1.09E-06	down
ENSMUSG00000033029	1700088E04Rik	-2.33E+00	6.35E-09	down	-1.60E+00	7.44E-06	down
ENSMUSG00000022427	Tomm22	-5.33E-01	3.98E-08	no	-1.06E+00	5.93E-44	down
ENSMUSG00000063480	Nhp2l1	2.02E+00	5.86E-22	up	1.60E+00	4.04E-09	up
ENSMUSG00000068114	Ccdc134	-1.68E+00	3.30E-03	no	-3.02E+00	2.97E-18	down
ENSMUSG00000022431	Ribc2	-2.38E+00	1.85E-22	down	-1.41E+00	5.85E-09	down
ENSMUSG00000053559	Smagp	4.15E+00	2.44E-04	up	3.94E+00	7.45E-03	no
ENSMUSG00000000552	Zfp385a	-4.64E+00	4.80E-34	down	-4.31E+00	2.13E-35	down
ENSMUSG00000022312	Eif3h	-1.75E+00	2.10E-29	down	-1.81E+00	3.64E-33	down
ENSMUSG00000079018	Ly6c1	5.35E+00	9.67E-12	up	3.28E+00	1.28E-03	no
ENSMUSG00000060794	Tssk5	-6.50E+00	2.76E-24	down	-3.50E+00	5.90E-03	no
ENSMUSG00000068252	Apol7b	-4.94E+00	8.42E-05	down	-3.09E+00	4.67E-01	no
ENSMUSG00000061360	Phf5a	2.48E+00	9.92E-21	up	3.53E-01	1.30E-01	no
ENSMUSG00000016619	Nup50	-2.52E+00	6.04E-19	down	-2.32E+00	1.18E-13	down
ENSMUSG00000052369	Tmem106c	7.44E-01	2.04E-01	no	-1.93E+00	5.55E-14	down
ENSMUSG00000003354	Ccdc65	-3.45E+00	1.09E-123	down	-2.29E+00	1.23E-53	down
ENSMUSG00000078907	Fam186b	-2.65E+00	8.14E-23	down	-2.24E+00	1.28E-23	down
ENSMUSG00000000532	Acvr1b	-1.22E+00	4.48E-01	no	-3.00E+00	3.11E-04	down
ENSMUSG00000049382	Krt8	-4.25E+00	1.55E-03	no	-4.74E+00	2.28E-05	down
ENSMUSG00000060992	Copz1	-4.60E-01	1.85E-01	no	-1.00E+00	2.34E-05	down
ENSMUSG00000050697	Prkaa1	-2.85E+00	1.92E-03	no	-3.41E+00	1.79E-06	down
ENSMUSG00000022332	Khdrbs3	-2.09E+00	1.09E-11	down	-2.49E+00	6.06E-31	down
ENSMUSG00000022587	Ly6e	4.81E+00	5.87E-21	up	3.61E+00	2.88E-05	up
ENSMUSG00000022445	Cyp2d26	-1.88E+00	1.82E-03	no	-2.09E+00	3.58E-05	down
ENSMUSG00000022434	Fam118a	-2.00E+00	3.70E-10	down	-2.62E+00	7.46E-28	down
ENSMUSG00000022280	Rnf19a	-3.32E+00	2.01E-83	down	-2.37E+00	7.83E-36	down
ENSMUSG00000038736	Nudcd1	-3.16E+00	1.88E-09	down	-3.41E+00	2.09E-13	down

ENSMUSG00000047003	Zfp41	-2.43E+00	1.15E-04	down	-2.70E+00	7.59E-09	down
ENSMUSG00000060036	Rpl3	-1.21E+00	1.63E-01	no	-2.51E+00	3.63E-04	down
ENSMUSG00000022453	Naga	-2.20E+00	2.81E-05	down	-3.68E-01	8.10E-01	no
ENSMUSG00000072235	Tuba1a	4.07E+00	2.23E-15	up	7.38E-01	2.40E-01	no
ENSMUSG00000022269	43535	-2.03E+00	1.63E-43	down	-2.15E+00	4.91E-59	down
ENSMUSG00000022300	Dcaf13	2.04E+00	4.27E-04	up	-1.43E+00	1.16E-05	down
ENSMUSG00000022337	Emc2	-1.19E+00	2.46E-05	down	-2.47E+00	5.51E-35	down
ENSMUSG00000022570	Tsta3	-1.26E+00	1.79E-09	down	-1.63E+00	6.42E-28	down
ENSMUSG00000068101	Cenpm	2.13E+00	2.07E-05	up	1.22E+00	4.83E-02	no
ENSMUSG00000041852	Tcf20	-4.06E+00	5.59E-09	down	-3.51E+00	4.24E-06	down
ENSMUSG00000022615	Tymp	-2.69E+00	9.52E-11	down	-2.12E+00	4.62E-08	down
ENSMUSG00000022375	Lrrc6	-2.74E+00	1.77E-39	down	-1.92E+00	1.16E-18	down
ENSMUSG00000075600	Zc3h3	-3.28E+00	2.31E-04	down	-2.68E+00	6.77E-03	no
ENSMUSG00000042535	Gtpbp1	-1.11E+00	6.54E-02	no	-2.02E+00	5.66E-06	down
ENSMUSG00000016541	Atxn10	-1.74E+00	3.97E-20	down	-1.66E+00	3.50E-20	down
ENSMUSG00000022385	Gtse1	1.14E+00	3.80E-01	no	-2.21E+00	6.72E-04	down
ENSMUSG00000045350	Fam186a	-3.54E+00	1.30E-09	down	-1.47E+00	1.01E-01	no
ENSMUSG00000022295	Atp6v1c1	-2.06E+00	1.12E-14	down	-2.35E+00	1.62E-22	down
ENSMUSG00000022336	Eif3e	-2.59E+00	5.24E-50	down	-3.25E+00	2.34E-102	down
ENSMUSG00000033047	Eif3l	-9.21E-01	9.29E-06	no	-1.20E+00	6.71E-11	down
ENSMUSG00000055065	Ddx17	-1.63E+00	1.33E-05	down	-3.16E+00	8.82E-20	down
ENSMUSG00000022412	Smcr7l	-3.07E+00	4.96E-06	down	-3.42E+00	7.28E-07	down
ENSMUSG00000051864	Tbc1d22a	-4.61E-01	4.46E-01	no	-1.75E+00	1.37E-05	down
ENSMUSG00000051934	Spats2	-2.51E+00	4.62E-09	down	-3.41E+00	1.86E-32	down
ENSMUSG00000022314	Rad21	-2.21E+00	4.45E-08	down	-8.83E-01	1.92E-01	no
ENSMUSG00000022362	9130401M01Rik	3.75E+00	3.14E-04	up	1.48E+00	1.14E-01	no
ENSMUSG00000022614	Lmf2	-1.84E+00	1.66E-05	down	-2.10E+00	2.67E-09	down
ENSMUSG00000022619	Mapk8ip2	-2.82E+00	1.42E-09	down	-2.48E+00	6.84E-11	down
ENSMUSG00000058655	Eif4b	-2.73E+00	1.64E-22	down	-2.01E+00	3.99E-09	down
ENSMUSG00000014313	Cox6c	2.65E+00	8.74E-74	up	9.76E-01	3.48E-18	no
ENSMUSG00000022353	Mtss1	5.25E+00	9.69E-18	up	4.64E+00	5.95E-16	up
ENSMUSG00000053929	Cyhr1	-3.65E+00	1.91E-19	down	-3.21E+00	8.46E-18	down
ENSMUSG00000016554	Eif3d	-2.09E+00	4.03E-24	down	-2.21E+00	1.27E-28	down
ENSMUSG00000022452	Smdt1	3.64E+00	1.03E-78	up	2.00E+00	2.21E-52	up
ENSMUSG00000022437	Samm50	8.84E-01	5.85E-02	no	-1.15E+00	4.52E-05	down
ENSMUSG00000022346	Myc	4.46E+00	1.60E-07	up	3.51E+00	7.48E-02	no
ENSMUSG00000033039	Micall1	-3.08E+00	1.38E-07	down	-2.78E+00	2.57E-04	down
ENSMUSG00000054277	Arfgap3	-2.33E+00	5.71E-18	down	-2.08E+00	1.04E-15	down
ENSMUSG00000022270	Fam134b	-4.79E+00	2.65E-11	down	-2.89E+00	1.22E-02	no
ENSMUSG00000033128	Gga1	-3.00E+00	1.90E-15	down	-3.33E+00	7.99E-24	down
ENSMUSG00000048175	Asb8	-2.57E+00	4.20E-13	down	-1.57E+00	8.32E-04	down
ENSMUSG00000022205	Sub1	2.46E+00	3.80E-37	up	2.90E-01	8.10E-02	no
ENSMUSG00000079105	C7	-4.51E+00	1.56E-10	down	-4.25E+00	4.46E-08	down
ENSMUSG00000062154	Tex33	-1.82E+00	3.01E-166	down	2.82E-01	3.56E-02	no
ENSMUSG00000039828	Wdr70	-1.95E+00	2.17E-07	down	-1.75E+00	8.80E-07	down
ENSMUSG00000022420	Dnalc4	-1.49E+00	4.69E-11	down	-9.45E-01	2.88E-05	no
ENSMUSG00000039704	Lmbrd2	-3.75E+00	2.91E-07	down	-2.37E+00	1.03E-01	no
ENSMUSG00000009575	Cbx5	-7.44E-01	3.82E-01	no	-2.72E+00	1.88E-07	down
ENSMUSG00000018040	Rrp7a	-1.60E+00	5.57E-03	no	-2.58E+00	8.65E-15	down
ENSMUSG00000054115	Skp2	-4.98E+00	1.49E-08	down	-3.34E+00	3.01E-02	no
ENSMUSG00000036800	Fam135b	-4.61E+00	7.85E-16	down	-1.38E+00	3.15E-01	no
ENSMUSG00000022456	43711	-4.00E+00	8.80E-07	down	-7.92E-01	7.33E-01	no
ENSMUSG00000015377	Dennd6b	-2.50E+00	1.09E-05	down	-1.76E+00	1.46E-02	no
ENSMUSG00000022422	Dscc1	-2.50E+00	6.45E-04	down	-4.08E-01	8.30E-01	no
ENSMUSG00000036561	Ppp6r2	-4.06E+00	1.21E-23	down	-1.42E+00	7.87E-02	no
ENSMUSG00000022620	Arsa	-3.40E+00	1.38E-41	down	-3.12E+00	5.00E-44	down
ENSMUSG00000022249	Ttc23l	-3.64E+00	1.25E-71	down	-1.17E+00	2.39E-03	no
ENSMUSG00000033099	Nol12	-1.16E+00	1.23E-01	no	-2.31E+00	2.24E-06	down
ENSMUSG00000018042	Cyb5r3	1.79E+00	2.39E-04	up	-2.05E-01	7.98E-01	no
ENSMUSG00000022378	Fam49b	-1.73E-01	8.38E-01	no	-1.88E+00	9.46E-04	down
ENSMUSG00000035805	Mlc1	-3.90E-01	5.54E-01	no	-2.38E+00	6.94E-17	down
ENSMUSG00000022234	Cct5	-1.87E+00	3.11E-54	down	-1.91E+00	2.26E-73	down
ENSMUSG00000022432	Smc1b	-1.51E+00	1.99E-20	down	-1.17E+00	1.01E-08	down
ENSMUSG00000039100	43530	-1.78E+00	1.06E-02	no	-2.48E+00	3.64E-04	down

ENSMUSG00000064373	Sepp1	2.80E+00	2.66E-13	up	1.98E+00	4.74E-03	no
ENSMUSG00000037343	Taf2	-7.58E-01	3.79E-01	no	-2.81E+00	3.24E-11	down
ENSMUSG00000041815	Poldip3	-1.56E+00	1.09E-03	no	-1.82E+00	8.86E-05	down
ENSMUSG00000064284	Cdpf1	-9.29E-01	1.26E-02	no	-1.12E+00	1.21E-05	down
ENSMUSG00000022361	Zhx1	-2.79E+00	1.41E-04	down	-3.80E+00	8.88E-12	down
ENSMUSG00000022360	Atad2	-2.06E+00	1.28E-04	down	-1.11E+00	4.03E-01	no
ENSMUSG00000058290	Espl1	-2.29E+00	1.18E-05	down	-3.07E+00	5.13E-11	down
ENSMUSG00000001280	Sp1	-2.17E+00	5.97E-02	no	-4.31E+00	1.08E-07	down
ENSMUSG00000022488	Nckap1l	-3.23E+00	1.32E-10	down	-8.72E-01	4.95E-01	no
ENSMUSG00000022285	Ywhaz	-4.32E-01	3.90E-01	no	-1.78E+00	4.63E-07	down
ENSMUSG00000035891	Cerk	-3.19E+00	3.11E-09	down	-3.03E+00	3.16E-11	down
ENSMUSG00000035845	Alg12	-4.56E+00	2.71E-10	down	-1.94E+00	1.09E-01	no
ENSMUSG00000078937	Cpt1b	-5.25E+00	1.02E-08	down	-4.71E+00	9.33E-08	down
ENSMUSG00000022351	Sqle	-2.68E+00	2.35E-12	down	-1.58E+00	1.84E-03	no
ENSMUSG00000023025	Larp4	-7.08E-01	4.52E-01	no	-2.41E+00	4.15E-04	down
ENSMUSG00000063704	Mapk15	-8.53E-01	9.20E-02	no	-2.19E+00	3.40E-21	down
ENSMUSG00000022477	Aco2	-1.94E+00	8.20E-11	down	-2.32E+00	4.58E-18	down
ENSMUSG00000016664	Pacsin2	-1.99E+00	4.52E-07	down	-1.33E+00	1.14E-02	no
ENSMUSG00000033237	Arid2	-2.61E+00	2.56E-09	down	-3.24E+00	2.46E-24	down
ENSMUSG00000022142	Nup155	-1.81E+00	5.60E-02	no	-3.34E+00	9.93E-06	down
ENSMUSG00000036167	Pphln1	-2.11E+00	1.30E-12	down	-5.48E-01	3.91E-01	no
ENSMUSG00000022246	Rai14	-4.83E+00	2.34E-75	down	-4.64E+00	2.69E-79	down
ENSMUSG00000034022	Cpsf1	-1.76E+00	3.38E-04	down	-2.42E+00	6.13E-09	down
ENSMUSG00000022472	Desi1	-2.81E+00	2.27E-50	down	-1.64E+00	5.36E-12	down
ENSMUSG00000037617	Spag1	-3.54E+00	1.96E-16	down	-3.23E+00	5.75E-19	down
ENSMUSG00000023027	Atf1	-1.08E+00	1.92E-03	no	-1.77E+00	3.51E-08	down
ENSMUSG00000022635	Zcrb1	-2.17E-01	6.42E-01	no	-1.48E+00	1.05E-07	down
ENSMUSG00000023055	Calcoco1	-8.14E-01	4.97E-01	no	-2.96E+00	5.20E-04	down
ENSMUSG00000022388	Ttll8	-6.91E+00	7.69E-48	down	-3.78E+00	2.13E-05	down
ENSMUSG00000022283	Pabpc1	-2.33E+00	3.41E-147	down	-1.79E+00	7.51E-102	down
ENSMUSG00000022155	Mroh2b	-5.43E+00	1.05E-103	down	-4.36E+00	3.53E-79	down
ENSMUSG00000022387	Brd1	-5.45E-01	5.49E-01	no	-2.17E+00	1.56E-04	down
ENSMUSG00000022454	Nell2	-1.15E+00	3.04E-01	no	-2.54E+00	7.03E-06	down
ENSMUSG00000056851	Pcbp2	-2.93E+00	2.91E-54	down	-3.19E+00	3.20E-83	down
ENSMUSG00000042564	Fam227a	-3.43E+00	9.96E-06	down	-2.45E+00	1.65E-02	no
ENSMUSG00000022442	Ttll1	-1.95E+00	5.52E-04	down	-2.27E+00	1.04E-07	down
ENSMUSG00000022439	Parvg	-4.59E+00	2.17E-09	down	-3.91E+00	2.55E-05	down
ENSMUSG00000033065	Pfkm	-3.45E+00	6.58E-37	down	-3.01E+00	2.69E-41	down
ENSMUSG00000053411	Cbx7	-2.47E+00	5.94E-03	no	-2.92E+00	4.67E-06	down
ENSMUSG00000022141	Nipbl	-2.10E+00	9.31E-16	down	-1.99E+00	7.40E-16	down
ENSMUSG00000022186	Oxct1	-1.56E+00	2.40E-05	down	-3.30E-01	6.19E-01	no
ENSMUSG00000033697	Arhgap39	-1.38E+00	7.09E-02	no	-2.57E+00	2.35E-04	down
ENSMUSG00000003360	Ddx23	-3.03E+00	1.11E-02	no	-4.63E+00	1.14E-06	down
ENSMUSG00000022391	Rangap1	-2.79E+00	9.32E-42	down	-1.29E+00	2.72E-05	down
ENSMUSG00000022999	Lmbr1l	-3.36E+00	2.41E-22	down	-2.80E+00	4.74E-14	down
ENSMUSG00000072487	Mroh5	-4.64E+00	1.28E-08	down	-1.20E+00	5.94E-01	no
ENSMUSG00000022555	Dgat1	-4.53E-01	2.69E-01	no	-1.46E+00	3.97E-09	down
ENSMUSG00000033819	Ppp1r16a	-1.59E+00	8.68E-05	down	-1.25E+00	3.32E-03	no
ENSMUSG00000022201	Zfr	-9.31E-01	3.54E-03	no	-1.62E+00	3.62E-08	down
ENSMUSG00000037487	Ubr5	-4.00E+00	4.05E-41	down	-3.98E+00	1.23E-47	down
ENSMUSG00000039458	Mtmr12	-2.61E+00	8.24E-07	down	-2.03E+00	2.74E-03	no
ENSMUSG00000022407	Adsl	2.32E+00	3.99E-06	up	-2.64E-01	6.55E-01	no
ENSMUSG00000039801	2410089E03Rik	-2.26E+00	1.77E-03	no	-2.18E+00	9.03E-04	down
ENSMUSG00000022369	Mtbp	-2.87E+00	1.17E-05	down	-2.77E+00	9.04E-05	down
ENSMUSG00000037627	Rgs22	-5.16E+00	1.20E-53	down	-3.87E+00	3.11E-22	down
ENSMUSG00000037458	Azin1	-1.98E+00	3.92E-05	down	-3.11E+00	3.24E-12	down
ENSMUSG00000022255	Mtdh	-2.28E+00	7.33E-11	down	-9.00E-01	1.36E-01	no
ENSMUSG00000022580	Rhpn1	-1.33E+00	2.11E-02	no	-1.38E+00	9.02E-04	down
ENSMUSG00000015365	Mov10l1	8.59E-01	6.05E-02	no	-1.39E+00	6.03E-06	down
ENSMUSG00000009739	Pou6f1	-1.27E+00	1.54E-01	no	-3.03E+00	1.54E-13	down
ENSMUSG00000022603	Mroh4	-2.59E+00	3.62E-63	down	-2.31E+00	7.25E-75	down
ENSMUSG00000022441	Efcab6	-5.01E+00	1.15E-66	down	-3.89E+00	5.83E-34	down
ENSMUSG00000047921	Trappc9	-1.92E+00	3.88E-04	down	-1.37E+00	4.93E-03	no
ENSMUSG00000042292	Mkl1	-2.41E+00	2.82E-04	down	-2.55E+00	1.77E-04	down

ENSMUSG00000036606	Plxnb2	-3.58E+00	7.53E-07	down	-3.27E+00	3.85E-05	down
ENSMUSG00000033075	Senp1	-2.61E+00	1.73E-04	down	-2.85E+00	4.80E-06	down
ENSMUSG00000023008	Fmnl3	-1.14E+00	3.86E-01	no	-3.51E+00	1.63E-04	down
ENSMUSG00000022617	Chkb	-1.50E+00	5.57E-02	no	-2.16E+00	2.12E-04	down
ENSMUSG00000022562	Oplah	-3.98E+00	1.11E-10	down	-2.13E+00	1.84E-02	no
ENSMUSG00000022364	Wdr67	-2.81E+00	4.85E-06	down	-2.50E+00	2.69E-04	down
ENSMUSG00000042961	Egflam	-2.86E+00	2.17E-04	down	-5.05E+00	5.25E-13	down
ENSMUSG00000068206	Pick1	-1.76E+00	9.51E-04	down	-1.23E+00	2.26E-02	no
ENSMUSG00000022307	Oxr1	-3.30E+00	1.94E-15	down	-2.70E+00	4.62E-08	down
ENSMUSG00000055762	Eef1d	-2.88E+00	5.80E-53	down	-1.13E+00	2.47E-05	down
ENSMUSG00000023026	Dip2b	-2.48E+00	6.53E-05	down	-5.57E-01	7.12E-01	no
ENSMUSG00000079022	Col22a1	-4.03E+00	8.19E-30	down	-1.72E+00	6.54E-03	no
ENSMUSG00000022394	L3mbtl2	-1.63E-01	8.79E-01	no	-2.70E+00	4.43E-09	down
ENSMUSG00000042632	Pla2g6	-2.97E+00	1.75E-36	down	-2.36E+00	6.37E-25	down
ENSMUSG00000072663	Spef2	-4.47E+00	3.11E-08	down	-3.99E+00	1.36E-07	down
ENSMUSG00000042303	Sgsm3	-1.44E+00	2.63E-04	down	-1.82E+00	1.38E-05	down
ENSMUSG00000022568	Scrib	-3.47E+00	5.63E-24	down	-3.34E+00	1.00E-32	down
ENSMUSG00000022483	Col2a1	-3.85E+00	1.18E-07	down	-3.05E+00	8.15E-05	down
ENSMUSG00000036529	Sbf1	-2.12E+00	7.15E-05	down	-2.58E+00	2.30E-08	down
ENSMUSG00000023007	Prpf40b	-1.76E+00	1.17E-04	down	-2.41E+00	9.46E-10	down
ENSMUSG00000022377	Asap1	-2.80E+00	6.72E-17	down	-1.45E+00	1.20E-03	no
ENSMUSG00000022565	Plec	-3.58E+00	2.72E-15	down	-2.49E+00	4.22E-07	down
ENSMUSG00000050058	Prm3	1.36E+00	4.59E-152	up	2.17E+00	3.98E-119	up
ENSMUSG00000091416	Gm6327	-5.00E+00	3.89E-07	down	-1.69E+00	4.77E-01	no
ENSMUSG00000071636	Rimbp3	-4.95E+00	1.24E-86	down	-3.89E+00	1.24E-53	down
ENSMUSG00000041566	Tssk1	-2.19E+00	8.16E-107	down	2.57E-02	9.37E-01	no
ENSMUSG00000045521	Tssk2	-1.92E+00	1.57E-112	down	6.21E-02	8.21E-01	no
ENSMUSG00000041378	Cldn5	4.23E+00	2.17E-04	up	2.71E+00	8.91E-03	no
ENSMUSG00000075269	Bex6	-2.15E+00	2.20E-01	no	-3.67E+00	6.52E-04	down
ENSMUSG00000067716	Gm7275	-4.09E+00	7.93E-16	down	-1.08E+00	3.24E-01	no
ENSMUSG00000050685	Ccdc54	-3.85E+00	7.90E-271	down	-1.79E+00	5.69E-19	down
ENSMUSG00000047293	Gpr15	-2.38E+00	1.74E-04	down	-5.34E-01	7.88E-01	no
ENSMUSG00000068167	Csnka2ip	-5.93E+00	2.76E-123	down	-3.02E+00	7.82E-14	down
ENSMUSG00000050299	Gm9843	3.16E+00	0.00E+00	up	2.25E+00	0.00E+00	up
ENSMUSG00000044227	Gm9789	1.71E+00	5.13E-04	up	2.44E+00	7.92E-03	no
ENSMUSG00000051728	4930563D23Rik	-2.64E+00	2.26E-37	down	-1.39E-02	9.85E-01	no
ENSMUSG00000038055	Dexi	3.19E+00	2.61E-19	up	2.85E+00	1.25E-26	up
ENSMUSG00000043050	Tnp2	-2.80E+00	0.00E+00	down	-2.50E+00	0.00E+00	down
ENSMUSG00000038015	Prm2	-2.24E+00	0.00E+00	down	-2.57E+00	0.00E+00	down
ENSMUSG00000022501	Prm1	-1.76E+00	0.00E+00	down	-1.97E+00	1.24E-156	down
ENSMUSG00000047141	Zfp654	-5.11E+00	1.92E-92	down	-4.00E+00	2.33E-56	down
ENSMUSG00000043140	Tmem186	-5.41E-01	5.99E-01	no	-1.85E+00	2.79E-04	down
ENSMUSG00000022769	Sdf2l1	3.35E-01	3.54E-01	no	-1.44E+00	1.57E-20	down
ENSMUSG00000022516	Nudt16l1	1.36E+00	1.27E-04	up	-8.51E-03	9.82E-01	no
ENSMUSG00000065968	Ifitm7	-6.73E-01	1.44E-01	no	-1.27E+00	3.68E-04	down
ENSMUSG00000002844	Adprh	5.51E-01	2.07E-01	no	-1.29E+00	2.65E-10	down
ENSMUSG00000022507	1810013L24Rik	-3.17E+00	8.32E-21	down	-3.40E+00	5.89E-22	down
ENSMUSG00000022765	Snap29	-2.35E+00	2.27E-06	down	-2.68E+00	8.70E-09	down
ENSMUSG00000090777	Gm7873	-3.44E+00	1.25E-49	down	-1.53E+00	8.96E-04	down
ENSMUSG00000059920	4930453N24Rik	-2.92E+00	1.35E-80	down	-2.34E+00	1.40E-45	down
ENSMUSG00000022878	Adipoq	-1.10E+00	6.11E-04	down	4.95E-01	5.46E-01	no
ENSMUSG00000022543	4930451G09Rik	-3.40E+00	3.43E-17	down	-3.20E+00	1.55E-19	down
ENSMUSG00000075033	Nxpe3	-4.73E+00	6.74E-03	no	-5.61E+00	3.17E-06	down
ENSMUSG00000004843	Chmp2b	-1.77E+00	5.59E-20	down	-2.19E+00	7.24E-40	down
ENSMUSG00000035376	Ptplb	-1.56E+00	2.15E-04	down	-5.62E-01	4.52E-01	no
ENSMUSG00000022828	Gtf2e1	-1.12E-01	9.33E-01	no	-2.50E+00	1.41E-04	down
ENSMUSG00000022798	4930435E12Rik	-4.72E+00	2.59E-36	down	-2.18E+00	2.69E-03	no
ENSMUSG00000039851	4932438H23Rik	-2.82E+00	1.31E-04	down	-1.20E+00	3.77E-01	no
ENSMUSG00000049916	2610318N02Rik	-2.68E+00	1.54E-145	down	-5.40E-01	5.05E-03	no
ENSMUSG00000062901	Klhl24	-2.56E+00	1.77E-04	down	-1.82E+00	3.87E-02	no
ENSMUSG00000004462	Tbccd1	-2.02E+00	1.40E-02	no	-3.53E+00	2.23E-10	down
ENSMUSG00000022542	43720	-1.96E+00	5.78E-37	down	-2.05E+00	1.86E-65	down
ENSMUSG00000051669	AU021092	2.90E+00	1.23E-01	no	3.11E+00	4.18E-04	up
ENSMUSG00000039763	Dnajc28	-1.88E+00	4.25E-03	no	-2.03E+00	6.29E-08	down

ENSMUSG00000022671	Mzt2	-1.41E+00	1.70E-24	down	-1.45E+00	1.55E-33	down
ENSMUSG00000022863	Btg3	4.62E+00	7.58E-05	up	1.91E+00	1.84E-01	no
ENSMUSG00000003527	Dgcr14	-2.41E+00	2.14E-06	down	-1.30E+00	3.28E-02	no
ENSMUSG00000021018	Polr2h	3.37E+00	3.28E-04	up	1.22E+00	2.20E-01	no
ENSMUSG00000035790	Cep19	-2.02E+00	7.69E-13	down	-1.39E+00	1.79E-06	down
ENSMUSG00000022787	Wdr53	-1.30E+00	5.42E-08	down	-1.56E+00	2.01E-15	down
ENSMUSG00000022832	Ropn1	-1.17E+00	2.24E-57	down	7.90E-01	6.30E-10	no
ENSMUSG00000022978	Mis18a	1.27E+00	2.02E-01	no	-1.99E+00	1.45E-04	down
ENSMUSG00000022938	Fam3b	-3.89E+00	2.40E-19	down	-1.01E+00	2.39E-01	no
ENSMUSG00000072214	43713	-5.14E+00	4.77E-23	down	-5.04E+00	1.65E-23	down
ENSMUSG00000039789	Zfp597	-3.39E+00	2.54E-10	down	-2.22E+00	2.95E-03	no
ENSMUSG00000022752	Tomm70a	-2.28E+00	1.52E-21	down	-2.71E+00	8.59E-35	down
ENSMUSG00000022889	Mrpl39	-1.95E+00	2.03E-22	down	-1.54E+00	2.75E-19	down
ENSMUSG00000039179	Tekt5	-4.54E+00	5.59E-64	down	-3.11E+00	2.26E-22	down
ENSMUSG00000022500	Litaf	5.94E+00	3.15E-18	up	4.56E+00	6.61E-07	up
ENSMUSG00000052363	Zdhhc19	-2.49E+00	4.51E-23	down	3.15E-01	6.49E-01	no
ENSMUSG00000022833	Ccdc14	-2.33E+00	5.09E-02	no	-3.51E+00	2.12E-05	down
ENSMUSG00000022683	Pla2g10	-3.31E+00	2.57E-26	down	-4.15E-01	6.33E-01	no
ENSMUSG00000022768	Ccdc116	-3.45E+00	1.33E-46	down	-1.13E+00	5.37E-03	no
ENSMUSG00000022808	Snx4	-5.87E-01	3.04E-01	no	-1.69E+00	1.24E-05	down
ENSMUSG00000022790	Igsf11	-2.84E+00	7.20E-15	down	-1.19E+00	4.32E-02	no
ENSMUSG00000032932	Hspa13	-3.02E+00	2.94E-04	down	-2.69E+00	1.92E-02	no
ENSMUSG00000044117	2900011O08Rik	-2.70E+00	1.69E-06	down	-2.60E+00	1.36E-06	down
ENSMUSG00000022657	Cd96	-2.30E+00	1.66E-06	down	-2.63E-01	8.51E-01	no
ENSMUSG00000022911	Arl13b	-1.88E+00	3.64E-08	down	4.68E-02	9.66E-01	no
ENSMUSG00000022864	D16Ertd472e	-2.90E+00	2.38E-26	down	-2.44E+00	1.20E-16	down
ENSMUSG00000022774	Ncbp2	-1.29E+00	6.85E-04	down	-1.58E+00	1.09E-05	down
ENSMUSG00000014301	Pam16	2.74E+00	5.98E-06	up	1.23E+00	1.63E-02	no
ENSMUSG00000040681	Hmgn1	1.73E+00	3.74E-06	up	9.15E-01	3.99E-02	no
ENSMUSG00000005846	Rsl1d1	3.82E-01	3.90E-01	no	-1.09E+00	9.25E-04	down
ENSMUSG00000022855	Senp2	-2.48E+00	4.95E-19	down	-3.26E+00	1.17E-47	down
ENSMUSG00000039456	Morc3	-9.05E-01	2.31E-01	no	-3.04E+00	3.90E-11	down
ENSMUSG00000005732	Ranbp1	2.44E-01	3.92E-01	no	-1.09E+00	1.00E-11	down
ENSMUSG00000034473	Sec22a	-1.45E+00	1.06E-01	no	-2.48E+00	2.33E-06	down
ENSMUSG00000022637	Cblb	-3.47E+00	3.89E-03	no	-4.52E+00	2.14E-07	down
ENSMUSG00000022749	Tbc1d23	-3.00E+00	3.40E-18	down	-1.15E+00	5.42E-02	no
ENSMUSG00000022964	Tmem50b	-3.89E-01	3.80E-01	no	-1.36E+00	1.41E-06	down
ENSMUSG00000050821	Fam131a	-3.36E+00	8.56E-12	down	-3.04E+00	2.36E-10	down
ENSMUSG00000000326	Comt	4.59E+00	2.37E-05	up	2.90E+00	2.05E-02	no
ENSMUSG00000005983	1700037C18Rik	-1.30E+00	2.46E-08	down	3.57E-01	3.68E-01	no
ENSMUSG00000003235	Eif2b5	-1.56E+00	3.32E-06	down	-2.13E+00	2.29E-18	down
ENSMUSG00000022753	Tmem30c	-1.48E-01	7.71E-01	no	-1.25E+00	1.12E-08	down
ENSMUSG00000022663	Atg3	-8.66E-01	7.19E-02	no	-1.43E+00	7.06E-04	down
ENSMUSG00000035578	Iqcg	-2.67E+00	2.34E-16	down	-2.59E+00	6.30E-24	down
ENSMUSG00000022867	Usp25	-4.92E+00	9.01E-29	down	-2.88E+00	1.99E-04	down
ENSMUSG00000060657	Marf1	-1.75E+00	1.59E-01	no	-4.25E+00	1.75E-08	down
ENSMUSG00000022744	Cldn25	-1.89E+00	1.19E-06	down	-2.81E-01	8.14E-01	no
ENSMUSG00000004070	Hmox2	-3.47E+00	8.44E-180	down	-1.75E+00	2.01E-23	down
ENSMUSG00000022529	Zfp263	-2.15E+00	3.04E-03	no	-3.11E+00	2.68E-07	down
ENSMUSG00000039345	Mettl22	-1.70E+00	1.21E-05	down	-1.53E+00	2.49E-06	down
ENSMUSG00000022972	1110004E09Rik	-4.02E-01	1.33E-03	no	-1.10E+00	2.59E-39	down
ENSMUSG00000022544	Fam86	1.78E+00	3.52E-04	up	7.66E-01	7.24E-02	no
ENSMUSG00000052299	Ltn1	-3.61E+00	4.22E-12	down	-3.48E+00	3.68E-08	down
ENSMUSG00000005981	Trap1	-1.29E+00	1.27E-06	down	-1.90E+00	2.80E-18	down
ENSMUSG00000004069	Dnaja3	-1.74E+00	3.05E-04	down	-1.26E+00	3.37E-02	no
ENSMUSG00000022711	Pmm2	-1.88E+00	6.93E-04	down	-2.11E+00	2.71E-06	down
ENSMUSG00000022805	Maats1	-3.41E+00	4.56E-20	down	-2.15E+00	1.10E-06	down
ENSMUSG00000022664	Slc35a5	-3.86E+00	1.33E-05	down	-2.58E+00	2.78E-02	no
ENSMUSG00000022536	Glyr1	-1.37E+00	6.95E-04	down	-3.05E+00	2.11E-25	down
ENSMUSG00000022722	Arl6	-1.40E+00	8.66E-05	down	-1.53E+00	6.60E-06	down
ENSMUSG00000022773	Ypel1	-9.07E-01	2.52E-06	no	-1.23E+00	2.41E-18	down
ENSMUSG00000041774	Ydjc	-2.68E+00	7.57E-04	down	-1.84E+00	7.51E-02	no
ENSMUSG00000032965	Ift57	-1.92E+00	1.12E-10	down	-1.47E+00	2.74E-06	down
ENSMUSG00000022545	Ercc4	-2.68E+00	8.11E-06	down	-2.12E+00	1.02E-03	no

ENSMUSG00000012114	Med15	-1.88E+00	8.90E-06	down	-2.02E+00	3.87E-08	down
ENSMUSG00000022757	Tfg	-1.67E+00	1.42E-06	down	-2.00E+00	7.93E-09	down
ENSMUSG00000022783	Spag6	-2.01E+00	8.54E-28	down	-2.25E+00	3.29E-50	down
ENSMUSG00000022812	Gsk3b	-1.84E+00	1.42E-05	down	-5.33E-02	9.66E-01	no
ENSMUSG00000071533	Pcnp	-8.28E-01	3.30E-01	no	-2.18E+00	2.87E-04	down
ENSMUSG00000022890	Atp5j	2.04E+00	1.40E-52	up	1.35E+00	4.69E-31	up
ENSMUSG00000045275	Lca5l	-2.87E+00	1.24E-19	down	-2.38E+00	1.04E-16	down
ENSMUSG00000022858	Tra2b	2.31E+00	8.46E-19	up	8.69E-01	5.54E-07	no
ENSMUSG00000036292	Gramd1c	-3.25E+00	2.13E-15	down	-3.39E+00	2.19E-25	down
ENSMUSG00000004460	Dnajb11	-8.48E-01	2.69E-02	no	-2.53E+00	2.13E-20	down
ENSMUSG00000014232	Cluap1	-2.36E+00	2.43E-11	down	-1.72E+00	9.17E-07	down
ENSMUSG00000022983	Scaf4	-2.57E+00	5.32E-07	down	-3.27E+00	2.67E-13	down
ENSMUSG00000062203	Gspt1	-1.98E+00	1.21E-06	down	-2.76E+00	1.29E-12	down
ENSMUSG00000038127	Ccdc50	-3.48E+00	6.52E-10	down	-1.29E+00	2.50E-01	no
ENSMUSG00000092009	Myh15	-5.24E+00	7.20E-06	down	-1.85E+00	5.80E-01	no
ENSMUSG00000022837	Iqcb1	1.31E+00	2.75E-02	no	-1.58E+00	5.28E-04	down
ENSMUSG00000039200	Atf7ip2	-2.87E+00	1.37E-04	down	-3.82E+00	2.71E-11	down
ENSMUSG00000003234	Abcf3	-2.68E+00	1.58E-20	down	-1.79E+00	9.20E-10	down
ENSMUSG00000005615	Pcyt1a	-2.64E+00	3.03E-06	down	-2.35E+00	1.89E-04	down
ENSMUSG00000022656	Pvrl3	-3.57E+00	9.52E-31	down	-2.95E+00	6.49E-21	down
ENSMUSG00000041617	Ccdc74a	-1.52E+00	3.84E-07	down	-1.50E+00	5.42E-10	down
ENSMUSG00000039903	Eva1c	-5.03E+00	1.06E-05	down	-4.30E+00	6.49E-03	no
ENSMUSG00000022681	Ntan1	-1.38E+00	2.39E-04	down	-5.41E-01	4.50E-01	no
ENSMUSG00000022800	Fyttd1	-1.41E+00	5.98E-08	down	-2.43E+00	3.58E-46	down
ENSMUSG00000039738	Slx4	-4.30E+00	1.15E-18	down	-4.08E+00	7.41E-24	down
ENSMUSG00000006998	Psmd2	-1.83E+00	1.60E-25	down	-1.91E+00	6.22E-32	down
ENSMUSG00000005982	Naa60	-2.58E+00	4.09E-04	down	-1.39E+00	2.63E-01	no
ENSMUSG00000022789	Dnm1l	-1.10E+00	3.84E-04	down	-5.49E-01	2.76E-01	no
ENSMUSG00000022723	Crybg3	-3.87E+00	1.72E-11	down	-4.18E+00	3.66E-23	down
ENSMUSG00000052459	Atp6v1a	-1.51E+00	1.14E-03	no	-1.80E+00	1.30E-05	down
ENSMUSG00000003166	Dgcr2	-1.96E+00	2.93E-04	down	-1.82E-01	8.91E-01	no
ENSMUSG00000022518	4930562C15Rik	-3.92E+00	2.18E-20	down	-1.66E+00	2.06E-02	no
ENSMUSG00000033031	C330027C09Rik	-3.26E+00	1.24E-06	down	-3.56E+00	2.46E-06	down
ENSMUSG00000039929	Urb1	-9.74E-01	1.75E-01	no	-2.39E+00	2.80E-08	down
ENSMUSG00000022538	Lsg1	-1.31E+00	1.39E-01	no	-2.68E+00	9.63E-06	down
ENSMUSG00000005262	Ufd1l	-1.80E+00	2.30E-07	down	-1.14E+00	1.19E-02	no
ENSMUSG00000058240	Cryzl1	-1.40E+00	3.40E-02	no	-1.91E+00	5.03E-05	down
ENSMUSG00000055692	Tmem191c	-1.76E+00	9.76E-09	down	2.32E-01	7.80E-01	no
ENSMUSG00000025616	Usp16	-2.51E+00	1.17E-09	down	-1.77E+00	5.85E-04	down
ENSMUSG00000022960	Donson	-2.43E+00	8.19E-13	down	-2.64E+00	1.06E-16	down
ENSMUSG00000035629	1700021K19Rik	-3.05E+00	1.47E-09	down	-2.62E+00	3.55E-09	down
ENSMUSG00000022961	Son	1.15E+00	1.46E-06	up	6.39E-01	1.99E-02	no
ENSMUSG00000022771	Ppil2	-1.38E+00	7.37E-04	down	-1.48E+00	9.86E-05	down
ENSMUSG00000071550	Wdr52	-3.86E+00	2.61E-10	down	-3.62E+00	4.29E-09	down
ENSMUSG00000022641	Bbx	-3.83E+00	2.92E-16	down	-1.01E+00	3.75E-01	no
ENSMUSG00000033210	Slc9c1	-4.90E+00	2.41E-40	down	-4.52E+00	1.99E-44	down
ENSMUSG00000037965	Zc3h7a	-5.10E-01	6.31E-01	no	-2.42E+00	6.26E-04	down
ENSMUSG00000022884	Eif4a2	-1.52E+00	2.07E-03	no	-3.28E+00	3.90E-22	down
ENSMUSG00000022680	Pdxdc1	-3.27E+00	2.00E-21	down	-2.72E+00	3.07E-12	down
ENSMUSG00000022779	Top3b	-1.54E+00	2.04E-02	no	-2.09E+00	6.17E-04	down
ENSMUSG00000022710	Usp7	-3.37E+00	6.63E-37	down	-2.21E+00	4.17E-10	down
ENSMUSG00000022801	Lrch3	-1.29E+00	3.37E-01	no	-3.03E+00	6.21E-05	down
ENSMUSG00000041720	Pi4ka	-2.67E+00	1.29E-05	down	-3.20E+00	4.92E-09	down
ENSMUSG00000005899	Smpd4	-2.85E+00	2.93E-08	down	-3.12E+00	9.52E-10	down
ENSMUSG00000045983	Eif4g1	-3.20E+00	1.12E-27	down	-1.45E+00	4.57E-03	no
ENSMUSG00000046173	Pabpc6	-3.12E+00	4.57E-65	down	-3.44E+00	4.32E-141	down
ENSMUSG00000044407	Qk	3.86E+00	6.66E-05	up	2.66E+00	7.55E-03	no
ENSMUSG00000091550	Gm6811	-4.72E+00	8.17E-04	down	-2.65E+00	6.30E-01	no
ENSMUSG00000067929	Gm10226	-1.31E+00	4.82E-01	no	-4.64E+00	4.29E-08	down
ENSMUSG00000046088	Gm9805	-5.07E+00	5.04E-85	down	-5.23E+00	4.73E-96	down
ENSMUSG00000045316	Fahd1	-1.62E+00	7.02E-03	no	-1.80E+00	3.42E-04	down
ENSMUSG00000049124	Gm8186	3.30E+00	0.00E+00	up	1.76E+00	1.61E-96	up
ENSMUSG00000023350	4921501E09Rik	-4.42E+00	1.02E-19	down	-1.58E+00	1.90E-01	no
ENSMUSG00000092519	Actl9	-2.01E+00	3.80E-08	down	-1.31E+00	1.73E-03	no

ENSMUSG00000031233	Pgk2	-3.37E+00	0.00E+00	down	-2.27E+00	1.59E-89	down
ENSMUSG00000062472	Gm4945	1.79E+00	1.20E-04	up	1.65E+00	1.84E-06	up
ENSMUSG00000044526	Znrf4	-1.18E+00	8.26E-30	down	8.34E-01	1.50E-06	no
ENSMUSG00000061062	Gm10093	-1.35E+00	1.27E-15	down	-1.06E+00	6.75E-10	down
ENSMUSG00000071035	Gm5499	-2.26E+00	1.14E-06	down	-1.97E-01	8.62E-01	no
ENSMUSG00000066878	Gm10184	-2.27E+00	2.66E-15	down	-1.29E+00	5.15E-04	down
ENSMUSG00000043445	Pgp	1.77E+00	1.51E-279	up	1.58E+00	0.00E+00	up
ENSMUSG00000007033	Hspa1l	-5.89E+00	4.09E-40	down	-4.10E+00	1.67E-10	down
ENSMUSG00000024409	Psors1c2	-2.35E+00	1.47E-01	no	-3.30E+00	1.97E-06	down
ENSMUSG00000003541	Ier3	5.27E+00	2.91E-59	up	3.25E+00	1.11E-08	up
ENSMUSG00000079722	Ttll2	-4.28E+00	2.40E-05	down	-3.27E+00	2.07E-01	no
ENSMUSG00000036533	Cdc42ep3	-7.48E-01	3.95E-03	no	-1.70E+00	3.23E-46	down
ENSMUSG00000066944	4921513D11Rik	-3.07E+00	1.09E-59	down	2.71E-01	6.19E-01	no
ENSMUSG00000071036	Gm10309	-4.24E+00	1.45E-04	down	-1.38E+00	6.87E-01	no
ENSMUSG00000053375	Atp6v1e2	-2.94E+00	4.67E-98	down	-4.75E-01	8.56E-02	no
ENSMUSG00000048602	Morc2b	-2.74E+00	9.35E-44	down	-1.66E+00	4.37E-21	down
ENSMUSG00000034509	Mad2l1bp	-2.17E+00	7.11E-06	down	-1.34E+00	7.21E-03	no
ENSMUSG00000094928	1700122O11Rik	-5.01E+00	3.27E-15	down	-1.54E+00	2.72E-01	no
ENSMUSG00000021545	1700067P10Rik	-2.62E+00	1.87E-59	down	-1.61E-01	7.25E-01	no
ENSMUSG00000044957	Pp2d1	-3.80E+00	1.61E-27	down	-7.21E-01	4.45E-01	no
ENSMUSG00000073380	Arrdc5	-4.22E+00	1.49E-111	down	-1.67E+00	1.08E-06	down
ENSMUSG00000090273	Prr22	-1.48E+00	7.51E-20	down	9.97E-01	2.01E-04	no
ENSMUSG00000050612	Txndc2	-5.86E+00	1.74E-252	down	-3.96E+00	6.79E-37	down
ENSMUSG00000039770	Ypel5	3.61E-01	5.22E-01	no	-1.47E+00	1.52E-05	down
ENSMUSG00000046196	Ttc39d	-4.22E+00	7.22E-100	down	-3.08E+00	2.47E-35	down
ENSMUSG00000037689	Tmem247	-1.32E+00	8.77E-49	down	1.47E-01	4.81E-01	no
ENSMUSG00000036648	Gm3417	1.86E+00	6.69E-78	up	1.26E+00	4.65E-64	up
ENSMUSG00000055839	Tceb2	2.11E+00	1.14E-237	up	1.81E+00	5.46E-269	up
ENSMUSG00000008482	Rnf151	-5.80E-01	5.81E-14	no	1.27E+00	5.51E-23	up
ENSMUSG00000051390	Zbtb22	-3.25E+00	1.19E-13	down	-2.88E+00	1.55E-11	down
ENSMUSG00000033739	Fkbpl	-2.23E+00	7.67E-52	down	-1.15E+00	5.22E-14	down
ENSMUSG00000004945	Tmem242	-1.45E+00	3.99E-13	down	-2.19E+00	3.34E-52	down
ENSMUSG00000057863	Rpl36	1.95E+00	1.64E-15	up	1.04E+00	2.65E-05	up
ENSMUSG00000002379	Ndufa11	3.96E+00	5.75E-50	up	2.70E+00	1.32E-49	up
ENSMUSG00000052031	Tagap1	-1.03E+00	8.65E-03	no	-1.43E+00	5.54E-04	down
ENSMUSG00000023892	Zfp51	-3.65E+00	1.41E-10	down	-3.10E+00	5.56E-06	down
ENSMUSG00000057411	Fam173a	1.49E+00	4.18E-04	up	-2.18E-01	4.99E-01	no
ENSMUSG00000044477	Zfand3	-2.68E+00	8.70E-49	down	-1.34E+00	1.75E-08	down
ENSMUSG00000023949	Tcte1	-4.03E+00	2.37E-61	down	-3.42E+00	6.99E-37	down
ENSMUSG00000096255	Dynlt1b	3.15E+00	1.55E-36	up	3.13E+00	1.65E-55	up
ENSMUSG00000095677	Dynlt1f	1.54E+00	2.04E-85	up	1.11E+00	1.80E-69	up
ENSMUSG00000045257	Morn2	1.38E+00	1.25E-138	up	1.34E+00	4.17E-151	up
ENSMUSG00000014769	Psmb1	1.75E+00	5.37E-32	up	-5.32E-02	7.71E-01	no
ENSMUSG00000033972	Zfp944	-3.01E+00	4.97E-07	down	-2.58E+00	1.02E-04	down
ENSMUSG00000024116	Prss21	-2.06E+00	9.12E-28	down	-2.25E+00	2.74E-42	down
ENSMUSG00000024175	Tekt4	-1.94E+00	1.03E-98	down	-1.75E-01	2.48E-01	no
ENSMUSG00000025739	Gng13	6.61E+00	2.86E-04	up	6.26E+00	1.82E-03	no
ENSMUSG00000024223	Armc12	-2.90E+00	1.95E-156	down	-3.08E-01	1.59E-01	no
ENSMUSG00000060586	H2-Eb1	4.90E+00	7.43E-27	up	5.39E+00	1.13E-06	up
ENSMUSG00000042419	Nfkbil1	-2.45E+00	6.12E-07	down	-2.38E+00	1.41E-06	down
ENSMUSG00000071073	Lrrc73	-3.03E+00	1.05E-22	down	-2.29E+00	2.13E-14	down
ENSMUSG00000002660	Clpp	1.93E+00	1.79E-05	up	5.82E-01	1.35E-01	no
ENSMUSG00000024091	Vapa	9.74E-02	7.37E-01	no	-1.53E+00	6.86E-20	down
ENSMUSG00000024072	Yipf4	3.68E-03	9.96E-01	no	-1.84E+00	4.33E-07	down
ENSMUSG00000024145	Pigf	-2.22E+00	7.36E-40	down	-9.10E-01	1.13E-04	no
ENSMUSG00000079707	Tcte3	1.07E+00	1.59E-02	no	3.52E+00	7.19E-68	up
ENSMUSG00000023905	Tnfrsf12a	6.44E+00	7.83E-14	up	3.85E+00	3.16E-04	up
ENSMUSG00000024132	Eci1	4.96E+00	8.55E-07	up	3.54E+00	2.59E-04	up
ENSMUSG00000024038	Ndufv3	2.28E+00	6.96E-14	up	1.61E+00	4.27E-13	up
ENSMUSG00000036983	Tfb1m	-2.86E+00	3.41E-17	down	-1.48E+00	1.82E-04	down
ENSMUSG00000015605	Srf	-2.06E+00	4.38E-05	down	-1.13E+00	1.40E-01	no
ENSMUSG00000023988	Bysl	-1.39E+00	4.07E-02	no	-2.11E+00	1.55E-06	down
ENSMUSG00000024248	Cox7a2l	-8.70E-01	1.36E-04	no	-2.00E+00	1.71E-27	down
ENSMUSG00000037196	Pacrg	-2.14E+00	4.85E-99	down	-1.07E+00	1.75E-21	down

ENSMUSG00000023067	Cdkn1a	3.48E+00	1.81E-08	up	2.39E+00	5.81E-03	no
ENSMUSG00000051611	Olfr112	-5.05E+00	2.28E-06	down	-1.32E+00	6.43E-01	no
ENSMUSG00000023961	Enpp4	-1.53E+00	7.47E-02	no	-2.28E+00	4.03E-04	down
ENSMUSG00000023935	Spats1	-1.31E+00	1.50E-02	no	-2.33E+00	9.43E-19	down
ENSMUSG00000034345	Gtf2h5	2.56E+00	6.61E-04	up	-4.29E-01	5.13E-01	no
ENSMUSG00000023806	Rsph3b	-1.80E+00	3.75E-05	down	-1.94E+00	2.08E-11	down
ENSMUSG00000034868	Myl12b	1.31E+00	1.33E-06	up	1.38E-01	7.80E-01	no
ENSMUSG00000073471	Rsph3a	-2.41E+00	2.15E-29	down	-2.55E+00	7.63E-70	down
ENSMUSG00000079710	Gm3448	2.10E+00	2.44E-04	up	3.89E+00	1.93E-57	up
ENSMUSG00000024190	Dusp1	1.66E+00	3.69E-04	up	2.20E+00	4.34E-02	no
ENSMUSG00000092586	Ly6g6c	-3.87E+00	1.67E-12	down	-1.58E+00	2.07E-01	no
ENSMUSG00000059791	Nrm	-2.14E+00	1.78E-20	down	5.53E-01	2.74E-01	no
ENSMUSG00000036214	Znrd1as	-2.61E+00	1.23E-49	down	-2.44E+00	8.04E-46	down
ENSMUSG00000023939	Mrpl14	3.06E+00	3.94E-05	up	1.71E+00	2.91E-03	no
ENSMUSG00000058704	Memo1	-1.00E+00	4.03E-07	down	-1.61E+00	3.41E-29	down
ENSMUSG00000095687	Rnaset2a	4.51E+00	1.02E-08	up	1.43E+00	2.02E-01	no
ENSMUSG00000055660	Mettl4	-2.78E+00	2.37E-05	down	-3.10E+00	2.03E-07	down
ENSMUSG00000024181	Mrpl28	2.49E+00	1.52E-20	up	1.48E+00	1.09E-12	up
ENSMUSG00000073421	H2-Ab1	4.14E+00	2.32E-39	up	2.11E+00	2.16E-04	up
ENSMUSG00000007041	Clic1	1.68E+00	1.53E-04	up	1.04E+00	1.02E-01	no
ENSMUSG00000023966	Rsph9	-1.10E+00	2.24E-12	down	-7.75E-01	1.45E-08	no
ENSMUSG00000003200	Sh3gl1	-1.13E+00	3.38E-06	down	-1.26E+00	5.44E-09	down
ENSMUSG00000024067	Dpy30	-1.68E+00	2.25E-36	down	-4.29E-01	5.61E-02	no
ENSMUSG00000024082	Ndufaf7	-8.40E-01	1.20E-01	no	-1.92E+00	1.72E-10	down
ENSMUSG00000033966	Cdkl4	-2.83E+00	2.15E-05	down	-4.11E+00	1.09E-23	down
ENSMUSG00000014763	Fam120b	-3.45E+00	1.80E-09	down	-3.18E+00	2.29E-09	down
ENSMUSG00000023904	Hcfc1r1	2.50E+00	4.33E-22	up	1.87E+00	9.08E-22	up
ENSMUSG00000073409	H2-Q6	4.42E+00	1.94E-04	up	1.54E+00	5.45E-01	no
ENSMUSG00000023919	Cenpq	3.79E-01	6.18E-01	no	-2.32E+00	3.93E-10	down
ENSMUSG00000023953	Polh	-1.72E+00	1.81E-01	no	-4.35E+00	7.34E-09	down
ENSMUSG00000010592	Dazl	3.05E+00	1.74E-66	up	5.43E-01	3.47E-03	no
ENSMUSG00000036368	Rmdn2	-3.60E+00	1.46E-39	down	-1.73E+00	7.04E-05	down
ENSMUSG00000035473	Galm	-2.79E+00	5.75E-07	down	-8.37E-01	4.80E-01	no
ENSMUSG00000052469	Tcp10c	-1.41E+00	8.34E-24	down	-1.14E+00	5.63E-17	down
ENSMUSG00000071267	Zfp942	-2.56E+00	2.61E-03	no	-3.26E+00	1.86E-04	down
ENSMUSG00000062012	Zfp13	-3.19E+00	4.72E-20	down	-1.36E+00	8.63E-03	no
ENSMUSG00000041130	Zfp598	-2.70E+00	8.78E-06	down	-3.00E+00	2.34E-07	down
ENSMUSG00000061126	Cyp4f39	-1.84E+00	6.25E-04	down	1.37E+00	2.46E-01	no
ENSMUSG00000002308	Cd320	-3.88E-01	1.23E-01	no	-1.24E+00	6.49E-18	down
ENSMUSG00000024325	Ring1	-1.93E+00	1.44E-02	no	-2.28E+00	5.43E-04	down
ENSMUSG00000092417	Gpank1	-1.77E+00	2.26E-10	down	-1.32E+00	1.94E-05	down
ENSMUSG00000001525	Tubb5	3.55E+00	4.39E-07	up	1.92E+00	2.42E-03	no
ENSMUSG00000047150	1700001C19Rik	-3.07E+00	2.85E-45	down	-1.51E+00	5.60E-05	down
ENSMUSG00000024212	Mllt1	-2.87E+00	5.42E-15	down	-2.64E+00	7.19E-14	down
ENSMUSG00000024146	Cript	-1.00E+00	1.32E-05	down	-1.32E+00	7.04E-10	down
ENSMUSG00000023882	Zfp54	-3.69E+00	3.45E-06	down	-3.93E+00	2.19E-09	down
ENSMUSG00000040097	Flywch1	-1.33E+00	5.27E-10	down	-1.15E+00	6.28E-12	down
ENSMUSG00000024194	Cuta	4.13E+00	2.03E-37	up	1.99E+00	5.68E-11	up
ENSMUSG00000040140	Tdrd6	-4.49E+00	4.60E-87	down	-4.11E+00	2.02E-95	down
ENSMUSG00000023931	Efhb	-3.36E+00	1.41E-40	down	-1.70E+00	1.79E-06	down
ENSMUSG00000038781	Stap2	-2.69E+00	3.49E-05	down	-2.72E+00	3.21E-04	down
ENSMUSG00000019579	D17Wsu104e	1.38E+00	5.23E-09	up	1.26E-01	6.44E-01	no
ENSMUSG00000002658	Gtf2f1	-1.98E+00	2.30E-60	down	-1.61E+00	5.18E-35	down
ENSMUSG00000024095	Hnrpll	-1.20E+00	1.30E-05	down	-2.29E+00	2.11E-27	down
ENSMUSG00000024253	Dync2li1	-1.98E+00	1.03E-08	down	-1.64E+00	8.05E-09	down
ENSMUSG00000024154	Gtf2a1l	-1.94E+00	7.02E-71	down	-1.11E+00	1.99E-23	down
ENSMUSG00000024137	E4f1	-3.25E-01	6.92E-01	no	-2.21E+00	5.69E-06	down
ENSMUSG00000024155	Meiob	1.91E+00	1.94E-06	up	-1.07E+00	4.10E-03	no
ENSMUSG00000056692	D17Wsu92e	-2.33E+00	1.34E-06	down	-1.22E+00	1.01E-01	no
ENSMUSG00000024218	Taf11	-1.91E+00	1.37E-06	down	-2.30E+00	5.26E-10	down
ENSMUSG00000024045	Akap8	-1.64E-02	9.82E-01	no	-2.63E+00	8.84E-14	down
ENSMUSG00000036594	H2-Aa	5.99E+00	9.79E-32	up	5.81E+00	6.07E-06	up
ENSMUSG00000024429	Gnl1	-2.20E+00	2.24E-19	down	-2.15E+00	9.71E-26	down
ENSMUSG00000002835	Chaf1a	-3.46E+00	9.59E-44	down	-2.62E+00	2.86E-22	down

ENSMUSG00000000579	Dynlt1c	5.61E+00	2.88E-30	up	4.03E+00	9.76E-34	up
ENSMUSG00000002017	Fam98a	-2.87E+00	1.06E-22	down	-2.79E+00	3.85E-22	down
ENSMUSG00000062078	Qk	-1.24E+00	2.35E-02	no	-2.61E+00	6.05E-12	down
ENSMUSG00000024193	Phf1	-1.88E+00	4.09E-10	down	-3.14E+00	2.22E-57	down
ENSMUSG00000015599	Ttbk1	-3.82E+00	2.46E-16	down	-3.20E+00	1.31E-11	down
ENSMUSG00000024207	Acsbg2	-4.65E+00	4.95E-84	down	-1.53E+00	6.88E-04	down
ENSMUSG00000053347	Zfp943	-3.24E+00	9.70E-04	down	-2.75E+00	9.56E-03	no
ENSMUSG00000061613	U2af1	2.59E+00	8.70E-08	up	7.16E-01	1.02E-01	no
ENSMUSG00000002076	Hsf2bp	-7.92E-01	4.63E-02	no	-1.21E+00	4.63E-05	down
ENSMUSG00000067212	H2-T23	6.51E+00	1.26E-05	up	6.98E+00	2.03E-02	no
ENSMUSG00000002833	Hdgfrp2	-1.59E+00	3.44E-15	down	-1.93E+00	1.59E-25	down
ENSMUSG00000002372	Ranbp3	-1.58E+00	7.57E-11	down	-2.12E+00	4.06E-30	down
ENSMUSG00000024059	Clip4	-4.41E+00	1.90E-92	down	-1.92E+00	1.21E-06	down
ENSMUSG00000024081	Cebpz	1.06E-01	8.73E-01	no	-1.47E+00	1.83E-04	down
ENSMUSG00000036438	Calm2	2.27E+00	7.56E-233	up	-4.08E-01	2.21E-31	no
ENSMUSG00000040048	Ndufb10	1.36E+00	2.00E-27	up	1.26E+00	6.90E-43	up
ENSMUSG00000024180	Tmem8	-2.94E+00	2.66E-15	down	-2.62E+00	5.71E-13	down
ENSMUSG00000024208	Mnf1	1.19E+00	7.22E-72	up	1.08E+00	2.53E-96	up
ENSMUSG00000024209	1700061G19Rik	-3.91E+00	4.21E-61	down	-7.58E-01	1.36E-01	no
ENSMUSG00000005823	Gpr108	-1.07E+00	8.12E-03	no	-2.01E+00	3.75E-14	down
ENSMUSG00000024056	Ndc80	-9.49E-01	1.12E-01	no	-2.85E+00	1.54E-15	down
ENSMUSG00000024068	Spast	-3.25E+00	6.60E-12	down	-3.71E+00	5.24E-18	down
ENSMUSG00000073411	H2-D1	7.47E+00	0.00E+00	up	4.26E+00	2.59E-55	up
ENSMUSG00000036315	Znrd1	-1.05E+00	1.24E-05	down	-1.01E+00	2.46E-04	down
ENSMUSG00000007670	Khsrp	-2.43E+00	1.16E-03	no	-4.18E+00	2.03E-11	down
ENSMUSG00000061607	Mdc1	-2.50E+00	5.71E-10	down	-4.08E+00	1.61E-45	down
ENSMUSG00000023266	Frs3	-8.15E-01	4.33E-01	no	-2.27E+00	3.81E-09	down
ENSMUSG00000057789	Bak1	-4.56E+00	4.99E-04	down	-4.61E+00	1.43E-09	down
ENSMUSG00000024078	Ttc27	-1.42E+00	1.94E-02	no	-2.03E+00	5.26E-05	down
ENSMUSG00000038002	Cramp1l	-2.10E+00	6.12E-02	no	-3.51E+00	6.14E-06	down
ENSMUSG00000042203	Tbc1d22b	-2.93E+00	6.68E-07	down	-2.77E+00	1.34E-05	down
ENSMUSG00000024446	Rpp21	1.82E+00	1.16E-14	up	1.43E+00	8.28E-17	up
ENSMUSG00000023938	Aars2	-2.51E+00	5.18E-05	down	-2.43E+00	1.83E-06	down
ENSMUSG00000023923	Tbc1d5	-2.12E+00	5.90E-04	down	-2.54E+00	2.39E-07	down
ENSMUSG00000040828	Catsperd	-2.83E+00	2.76E-29	down	-3.21E+00	8.04E-61	down
ENSMUSG00000024227	2610034M16Rik	-5.32E+00	2.13E-04	down	-5.22E+00	5.79E-05	down
ENSMUSG00000024101	Wash	-2.57E+00	7.89E-04	down	-2.80E+00	5.93E-06	down
ENSMUSG00000046991	Wdr27	-2.44E+00	9.22E-05	down	-2.59E+00	2.95E-07	down
ENSMUSG00000071172	Srsf3	1.07E+00	7.05E-02	no	-1.87E+00	6.75E-08	down
ENSMUSG00000067288	Rps28	4.00E+00	4.29E-35	up	2.75E+00	3.60E-18	up
ENSMUSG00000024309	H2-Ke2	1.88E+00	2.29E-29	up	1.23E+00	5.59E-21	up
ENSMUSG00000063576	Klhdc3	-2.86E+00	3.45E-112	down	-2.29E+00	1.91E-76	down
ENSMUSG00000024048	Myl12a	2.98E+00	7.08E-12	up	1.39E+00	1.05E-02	no
ENSMUSG00000033855	Ston1	-4.85E+00	2.09E-06	down	-3.16E+00	2.46E-01	no
ENSMUSG00000061130	Ppm1b	-1.87E+00	4.35E-11	down	-8.79E-01	3.22E-03	no
ENSMUSG00000059811	Atl2	-2.15E+00	3.51E-05	down	-2.35E+00	2.40E-07	down
ENSMUSG00000041881	Ndufa7	3.34E+00	2.28E-286	up	3.04E+00	8.32E-282	up
ENSMUSG00000023802	Nox3	-4.16E+00	7.89E-04	down	-1.36E+00	6.90E-01	no
ENSMUSG00000052525	Spdya	-7.30E-01	9.57E-04	no	-1.24E+00	3.37E-14	down
ENSMUSG00000035842	Ddx11	-1.01E+00	4.11E-01	no	-2.66E+00	1.22E-05	down
ENSMUSG00000048249	Crebrf	-1.44E+00	9.08E-02	no	-3.67E+00	3.18E-10	down
ENSMUSG00000058626	Capn11	-4.85E+00	4.19E-16	down	-4.70E+00	9.53E-31	down
ENSMUSG00000024150	Mcfd2	5.30E-01	5.17E-01	no	-1.81E+00	5.44E-05	down
ENSMUSG00000023883	Phf10	-1.38E+00	8.73E-08	down	-1.57E+00	4.59E-13	down
ENSMUSG00000015597	Zfp318	-2.76E+00	4.61E-11	down	-3.65E+00	2.49E-27	down
ENSMUSG00000040852	Plekhh2	-5.63E+00	5.64E-09	down	-4.18E+00	3.21E-03	no
ENSMUSG00000046711	Hmga1	-2.76E-01	7.56E-01	no	-1.60E+00	9.49E-04	down
ENSMUSG00000024085	Man2a1	-3.41E+00	7.22E-05	down	-1.23E+00	5.01E-01	no
ENSMUSG00000045036	Tmem232	-4.14E+00	1.53E-46	down	-2.72E+00	1.98E-14	down
ENSMUSG00000036196	Slc26a8	-3.27E+00	1.12E-27	down	-3.33E+00	1.05E-36	down
ENSMUSG00000023930	Crisp2	-4.16E+00	0.00E+00	down	-4.02E+00	0.00E+00	down
ENSMUSG00000067150	Xpo5	-3.23E+00	3.88E-11	down	-2.89E+00	4.05E-09	down
ENSMUSG00000000127	Fer	-1.97E+00	6.84E-05	down	-2.20E+00	4.27E-05	down
ENSMUSG00000036820	Amdhd2	-1.47E+00	2.24E-04	down	-1.96E+00	1.15E-09	down

ENSMUSG00000039512	Uhrf1bp1	-3.87E+00	6.48E-05	down	-1.60E+00	5.31E-01	no
ENSMUSG00000055602	Tcp10b	-1.60E+00	6.27E-15	down	-1.43E+00	8.48E-14	down
ENSMUSG00000042099	Kank3	-2.00E+00	1.29E-09	down	-1.99E+00	6.97E-17	down
ENSMUSG00000024206	Rfx2	-3.02E+00	4.41E-54	down	-2.44E+00	1.57E-40	down
ENSMUSG00000050705	2310061I04Rik	-1.13E+00	2.34E-04	down	-1.22E+00	5.88E-05	down
ENSMUSG00000024083	Pja2	-1.64E+00	4.96E-04	down	-2.29E+00	3.31E-09	down
ENSMUSG00000024182	Axin1	-2.45E-01	7.28E-01	no	-1.74E+00	3.31E-04	down
ENSMUSG00000024213	Nudt3	-2.42E+00	5.63E-15	down	-3.26E+00	1.38E-39	down
ENSMUSG00000023944	Hsp90ab1	2.16E+00	8.20E-64	up	1.12E+00	4.84E-21	up
ENSMUSG00000069135	Fgfr1op	-1.31E+00	6.33E-05	down	-1.94E+00	8.21E-12	down
ENSMUSG00000068037	Mas1	-3.83E+00	5.41E-17	down	-6.00E-01	6.56E-01	no
ENSMUSG00000024397	Aif1	-1.89E+00	4.57E-74	down	-2.57E-01	2.57E-01	no
ENSMUSG00000038954	Supt3	-2.76E+00	6.76E-07	down	-3.15E+00	2.32E-10	down
ENSMUSG00000067629	Syngap1	-6.12E+00	7.18E-10	down	-5.42E+00	2.15E-05	down
ENSMUSG00000038347	Tcte2	-1.31E+00	5.66E-04	down	-9.36E-01	3.33E-03	no
ENSMUSG00000045409	Trim39	-2.77E+00	2.21E-06	down	-3.28E+00	1.25E-09	down
ENSMUSG00000019320	Noxo1	-3.96E+00	4.27E-06	down	-3.38E+00	1.71E-04	down
ENSMUSG00000040276	Pacsin1	-3.15E+00	2.99E-02	no	-3.93E+00	5.58E-05	down
ENSMUSG00000002763	Pex6	-2.50E+00	1.12E-06	down	-2.06E+00	5.78E-04	down
ENSMUSG00000000708	Kat2b	-3.45E+00	4.64E-06	down	-1.13E+00	5.99E-01	no
ENSMUSG00000006705	Pknox1	-2.45E+00	1.04E-03	no	-2.65E+00	2.82E-05	down
ENSMUSG00000062859	Tcp11	-1.70E+00	1.96E-231	down	-7.23E-01	2.45E-40	no
ENSMUSG00000019578	Ubxn6	-1.99E+00	8.22E-25	down	-1.69E+00	4.28E-22	down
ENSMUSG00000024044	Epb4.1l3	-2.83E+00	1.44E-07	down	-5.43E-01	6.88E-01	no
ENSMUSG00000002307	Daxx	-2.08E+00	1.82E-12	down	-1.92E+00	4.16E-14	down
ENSMUSG00000004865	Srpk1	-2.17E+00	6.70E-17	down	-2.30E+00	6.27E-20	down
ENSMUSG00000061232	H2-K1	5.37E+00	1.42E-187	up	1.60E-01	7.12E-01	no
ENSMUSG00000034254	Agpat1	-4.26E+00	3.17E-76	down	-3.57E+00	6.63E-52	down
ENSMUSG00000019432	Ddx39b	9.05E-01	5.74E-02	no	-1.11E+00	2.34E-04	down
ENSMUSG00000040482	Dom3z	-1.01E-01	8.11E-01	no	-1.21E+00	6.93E-05	down
ENSMUSG00000024369	Nelfe	-1.21E+00	3.49E-11	down	-1.08E+00	3.03E-09	down
ENSMUSG00000023913	Pla2g7	-2.07E+00	4.18E-05	down	-2.94E-01	8.66E-01	no
ENSMUSG00000036036	Zfp57	-5.21E+00	2.87E-11	down	-3.53E+00	3.21E-03	no
ENSMUSG00000039220	Ppp1r10	-2.53E+00	1.19E-14	down	-2.09E+00	2.58E-07	down
ENSMUSG00000068039	Tcp1	-2.43E+00	4.30E-159	down	-2.07E+00	1.49E-153	down
ENSMUSG00000063952	Brpf3	-3.03E+00	8.50E-07	down	-2.66E+00	2.42E-07	down
ENSMUSG00000041354	Rgl2	-1.51E+00	1.09E-01	no	-2.96E+00	7.22E-05	down
ENSMUSG00000024422	Dhx16	-2.78E+00	2.08E-11	down	-3.13E+00	2.48E-19	down
ENSMUSG00000035435	Abca17	-3.78E+00	1.40E-09	down	-3.92E+00	1.39E-15	down
ENSMUSG00000080316	Ncrna00085	-3.44E+00	4.22E-06	down	-7.66E-01	7.17E-01	no
ENSMUSG00000007036	Abhd16a	-1.17E+00	8.68E-05	down	-1.33E+00	4.58E-07	down
ENSMUSG00000024220	Zfp523	-1.33E+00	8.81E-03	no	-2.25E+00	2.67E-10	down
ENSMUSG00000015461	Atf6b	-2.12E-01	8.14E-01	no	-2.13E+00	1.09E-04	down
ENSMUSG00000040312	Cchcr1	-4.30E+00	3.43E-66	down	-4.13E+00	3.78E-90	down
ENSMUSG00000034377	Tulp4	-2.34E+00	5.25E-03	no	-3.04E+00	2.04E-04	down
ENSMUSG00000014767	Tbp	-1.61E+00	3.38E-03	no	-1.94E+00	1.28E-05	down
ENSMUSG00000059562	Ccdc154	-2.21E+00	5.04E-07	down	-2.00E+00	2.28E-08	down
ENSMUSG00000024242	Map4k3	-2.92E+00	6.43E-05	down	-3.16E+00	4.48E-04	down
ENSMUSG00000071322	Tcp10a	-1.58E+00	8.97E-20	down	-1.46E+00	9.23E-20	down
ENSMUSG00000061046	Haghl	-1.13E+00	2.26E-02	no	-1.29E+00	1.26E-04	down
ENSMUSG00000024393	Prrc2a	-5.50E+00	1.08E-71	down	-5.24E+00	1.00E-77	down
ENSMUSG00000001228	Uhrf1	-3.60E+00	1.51E-07	down	-4.02E+00	1.32E-15	down
ENSMUSG00000013236	Ptprs	-8.77E-01	4.26E-01	no	-2.38E+00	6.69E-05	down
ENSMUSG00000024319	Vps52	-1.50E+00	1.17E-03	no	-1.74E+00	2.41E-06	down
ENSMUSG00000024335	Brd2	-2.28E+00	2.96E-24	down	-2.13E+00	1.26E-19	down
ENSMUSG00000023952	Gtpbp2	-3.45E+00	4.73E-08	down	-3.11E+00	2.34E-06	down
ENSMUSG00000059208	Hnrnpm	1.16E+00	2.34E-03	no	-1.47E+00	2.51E-12	down
ENSMUSG00000024122	Pdpk1	-3.30E+00	1.64E-09	down	-2.21E+00	2.10E-02	no
ENSMUSG00000034165	Ccnd3	-1.93E+00	1.23E-04	down	3.34E-01	8.37E-01	no
ENSMUSG00000071054	Safb	-5.11E-01	4.73E-01	no	-2.35E+00	5.57E-09	down
ENSMUSG00000036918	Ttc7	-3.52E+00	6.52E-06	down	-2.78E+00	7.18E-02	no
ENSMUSG00000033826	Dnahc8	-3.94E+00	2.41E-57	down	-3.77E+00	9.21E-110	down
ENSMUSG00000061950	Ppp4r1	-4.67E+00	7.14E-37	down	-2.75E+00	2.40E-08	down
ENSMUSG00000024169	Ift140	-3.77E+00	4.50E-41	down	-3.36E+00	2.83E-53	down

ENSMUSG00000042625	Safb2	-1.56E+00	4.10E-03	no	-1.87E+00	3.62E-04	down
ENSMUSG00000057246	BC051142	-2.65E+00	1.86E-101	down	-1.83E+00	6.65E-44	down
ENSMUSG00000007029	Vars	-3.39E+00	5.84E-33	down	-1.44E+00	1.04E-03	no
ENSMUSG00000024426	Atat1	-2.56E+00	1.29E-05	down	-7.52E-01	5.28E-01	no
ENSMUSG00000024112	Cacna1h	-4.62E+00	3.96E-13	down	-4.65E+00	1.44E-19	down
ENSMUSG00000040356	Skiv2l	-3.04E+00	6.23E-18	down	-3.00E+00	4.15E-21	down
ENSMUSG00000013787	Ehmt2	-3.69E+00	3.43E-38	down	-3.19E+00	4.73E-28	down
ENSMUSG00000024392	Bag6	-4.33E+00	1.73E-108	down	-3.75E+00	4.04E-88	down
ENSMUSG00000023868	Pde10a	-2.64E+00	5.55E-02	no	-2.97E+00	3.22E-04	down
ENSMUSG00000023942	Slc29a1	4.64E-01	3.37E-01	no	-1.85E+00	3.44E-05	down
ENSMUSG00000023805	Synj2	-4.27E+00	1.21E-06	down	-2.29E+00	2.50E-01	no
ENSMUSG00000058064	Gm10036	-5.11E-01	3.02E-01	no	-1.64E+00	1.49E-06	down
ENSMUSG00000091449	Gm10269	2.60E+00	1.12E-95	up	1.01E+00	3.09E-16	up
ENSMUSG00000050304	Slc25a2	-1.82E+00	1.48E-14	down	-1.40E+00	4.09E-08	down
ENSMUSG00000051732	Pabpc2	-2.73E+00	2.14E-174	down	-2.20E+00	2.61E-104	down
ENSMUSG00000048875	Hdhd1a	-2.93E+00	7.14E-52	down	-2.14E+00	6.53E-31	down
ENSMUSG00000069379	Gm4950	1.88E+00	1.45E-12	up	3.02E-01	2.02E-01	no
ENSMUSG00000024510	Ftmt	-2.32E+00	1.67E-31	down	4.17E-01	3.43E-01	no
ENSMUSG00000053624	Gykl1	-2.73E+00	2.03E-178	down	-1.51E+00	4.66E-41	down
ENSMUSG00000051401	Kctd16	-5.35E+00	7.19E-24	down	-3.90E+00	4.72E-05	down
ENSMUSG00000059040	Gm5506	-2.11E+00	9.75E-08	down	-1.56E+00	6.18E-03	no
ENSMUSG00000046886	Zfp474	-5.03E+00	6.60E-57	down	-2.67E+00	1.23E-05	down
ENSMUSG00000037253	Mex3c	-4.09E+00	8.78E-20	down	-2.53E+00	5.68E-03	no
ENSMUSG00000063281	Zfp35	-3.00E+00	5.95E-32	down	-3.17E+00	6.26E-62	down
ENSMUSG00000046668	Cxxc5	-2.07E+00	2.29E-19	down	4.70E-01	3.85E-01	no
ENSMUSG00000014294	Ndufa2	4.41E+00	0.00E+00	up	3.58E+00	0.00E+00	up
ENSMUSG00000033319	Fem1c	-3.98E+00	9.69E-44	down	-3.16E+00	3.52E-23	down
ENSMUSG00000036299	BC031181	1.71E+00	5.97E-09	up	2.64E-01	3.28E-01	no
ENSMUSG00000002477	Snrpd1	1.33E+00	1.16E-14	up	-5.47E-02	8.37E-01	no
ENSMUSG00000024276	Zfp397	-2.09E+00	5.56E-02	no	-3.80E+00	3.93E-08	down
ENSMUSG00000024258	Polr2d	2.14E+00	1.53E-06	up	-5.16E-01	1.47E-01	no
ENSMUSG00000091561	Gm6665	4.95E+00	9.46E-153	up	2.73E+00	8.37E-41	up
ENSMUSG00000059455	Plac8l1	-2.05E+00	1.21E-43	down	6.63E-01	6.98E-03	no
ENSMUSG00000033960	9430020K01Rik	-1.19E+00	5.02E-02	no	-1.60E+00	5.26E-04	down
ENSMUSG00000032905	Atg12	-1.06E+00	8.19E-03	no	-1.80E+00	2.75E-09	down
ENSMUSG00000073568	Arl14epl	-1.21E+00	1.15E-01	no	-3.18E+00	1.19E-15	down
ENSMUSG00000035420	Fam170a	-2.23E+00	1.02E-39	down	-1.85E+00	3.50E-36	down
ENSMUSG00000001473	Tubb6	1.30E+00	1.79E-10	up	1.86E+00	1.41E-04	up
ENSMUSG00000005873	Reep5	2.57E+00	4.21E-05	up	7.97E-01	2.90E-01	no
ENSMUSG00000038418	Egr1	6.23E+00	1.60E-182	up	3.54E+00	6.68E-10	up
ENSMUSG00000050945	Zfp438	-3.32E+00	4.99E-22	down	-2.20E+00	7.66E-07	down
ENSMUSG00000024486	Hbegf	4.72E+00	6.42E-05	up	1.03E+00	4.25E-01	no
ENSMUSG00000001383	Zmat2	6.18E-01	1.11E-01	no	-1.10E+00	4.05E-06	down
ENSMUSG00000024487	Yipf5	-9.82E-01	4.02E-03	no	-1.51E+00	4.56E-08	down
ENSMUSG00000024480	Ap3s1	-1.59E+00	1.90E-05	down	-9.94E-01	2.72E-02	no
ENSMUSG00000024645	Timm21	-1.74E+00	3.38E-04	down	-1.43E+00	5.75E-03	no
ENSMUSG00000036743	Psma8	5.51E-01	2.14E-02	no	-1.51E+00	1.16E-36	down
ENSMUSG00000057561	Eif1a	-4.33E-01	2.60E-01	no	-1.38E+00	1.59E-06	down
ENSMUSG00000024532	1700034E13Rik	-2.93E+00	4.37E-132	down	-2.03E+00	3.07E-19	down
ENSMUSG00000024259	Slc25a46	-2.47E+00	6.92E-15	down	-7.65E-01	1.78E-01	no
ENSMUSG00000041915	Ammecr1l	-2.71E+00	3.80E-10	down	-2.95E+00	1.31E-16	down
ENSMUSG00000038555	Reep2	-2.15E+00	1.26E-19	down	-1.57E+00	3.98E-11	down
ENSMUSG00000024350	Dnajc18	-2.80E+00	4.87E-16	down	-1.71E+00	2.82E-04	down
ENSMUSG00000024425	Ndfip1	-3.23E-01	1.37E-01	no	-1.44E+00	9.02E-18	down
ENSMUSG00000024386	Proc	-2.89E+00	7.60E-05	down	-1.25E+00	4.80E-01	no
ENSMUSG00000024233	Lyzl1	-2.30E+00	3.96E-96	down	1.63E-02	9.66E-01	no
ENSMUSG00000024234	Mtpap	-1.33E+00	4.72E-02	no	-2.17E+00	1.48E-04	down
ENSMUSG00000044906	4930503L19Rik	-2.21E+00	4.12E-17	down	-1.97E+00	1.50E-14	down
ENSMUSG00000041840	Haus1	-9.76E-01	4.46E-14	no	-1.58E+00	2.52E-56	down
ENSMUSG00000024395	Lims2	-1.56E+00	5.24E-03	no	-2.51E+00	3.15E-07	down
ENSMUSG00000034300	Fam53c	-3.52E+00	2.29E-20	down	-1.81E+00	1.12E-02	no
ENSMUSG00000024357	Sil1	-1.64E+00	3.54E-22	down	-1.33E+00	9.40E-16	down
ENSMUSG00000071855	Ccdc112	-6.49E-01	6.49E-01	no	-2.52E+00	2.67E-04	down
ENSMUSG00000052713	Zfp608	-4.16E+00	1.87E-08	down	-3.38E+00	2.19E-06	down

ENSMUSG00000039540	4921524L21Rik	-3.46E+00	3.96E-22	down	-3.33E+00	2.42E-30	down
ENSMUSG00000024286	Ccny	-1.01E+00	4.27E-02	no	-2.36E+00	9.81E-12	down
ENSMUSG00000024588	Fech	-1.78E-01	8.28E-01	no	-2.04E+00	2.89E-04	down
ENSMUSG00000090000	Ier3ip1	2.34E+00	4.56E-22	up	1.13E+00	2.80E-08	up
ENSMUSG00000024360	Etf1	-2.63E+00	1.52E-30	down	-1.45E+00	7.62E-05	down
ENSMUSG00000032900	1700065I17Rik	-1.50E+00	2.86E-35	down	1.57E-01	6.23E-01	no
ENSMUSG00000073542	Cep76	-2.23E+00	1.87E-03	no	-2.73E+00	2.78E-04	down
ENSMUSG00000024404	Riok3	-3.15E+00	1.55E-26	down	-3.24E+00	1.87E-24	down
ENSMUSG00000049357	4933408B17Rik	-2.42E+00	9.04E-02	no	-5.23E+00	2.82E-13	down
ENSMUSG00000024442	0610009O20Rik	-3.59E+00	3.57E-17	down	-4.24E+00	6.04E-40	down
ENSMUSG00000024603	Dctn4	-1.66E+00	1.24E-05	down	-1.79E+00	1.53E-08	down
ENSMUSG00000024269	Tpgs2	-1.75E+00	9.29E-11	down	-1.69E+00	1.49E-09	down
ENSMUSG00000056812	St8sia3	-5.14E+00	2.02E-13	down	-1.90E+00	1.81E-01	no
ENSMUSG00000036223	Ska1	-1.93E+00	4.01E-03	no	-2.46E+00	9.80E-07	down
ENSMUSG00000057130	Txnl4a	2.72E+00	4.47E-14	up	1.47E+00	1.68E-06	up
ENSMUSG00000069441	Dsg1a	-6.64E+00	5.77E-10	down	-7.58E+00	7.78E-02	no
ENSMUSG00000024317	Rnf138	-2.49E+00	3.09E-49	down	-1.69E+00	2.30E-14	down
ENSMUSG00000044201	Cdc25c	-2.38E+00	1.51E-06	down	-6.32E-01	5.31E-01	no
ENSMUSG00000037236	Matr3	-2.82E+00	1.16E-40	down	-3.72E+00	1.11E-89	down
ENSMUSG00000091896	Ube2d2a	-1.55E+00	1.18E-04	down	-2.56E+00	2.21E-14	down
ENSMUSG00000024590	Lmnb1	-1.85E+00	1.46E-01	no	-3.16E+00	8.51E-04	down
ENSMUSG00000024587	Nars	2.69E+00	2.57E-10	up	8.21E-01	2.56E-02	no
ENSMUSG00000024560	Cxxc1	-1.51E+00	1.22E-04	down	-2.63E+00	5.94E-15	down
ENSMUSG00000024561	Mbd1	-1.30E+00	3.76E-01	no	-3.60E+00	3.81E-06	down
ENSMUSG00000052102	Gnpda1	-8.53E-01	3.28E-01	no	-2.91E+00	1.10E-08	down
ENSMUSG00000024527	Afg3l2	-7.63E-01	1.79E-01	no	-2.14E+00	1.95E-07	down
ENSMUSG00000047989	Ino80c	-1.69E+00	9.63E-06	down	-2.80E+00	1.20E-21	down
ENSMUSG00000035984	Nme5	-2.11E+00	4.29E-62	down	-1.25E+00	3.37E-18	down
ENSMUSG00000024474	Ik	-2.12E+00	2.86E-37	down	-2.21E+00	4.22E-48	down
ENSMUSG00000033949	Trim36	-3.60E+00	1.73E-88	down	-1.16E+00	8.87E-04	down
ENSMUSG00000036501	Fam13b	-3.30E+00	1.06E-14	down	-3.99E+00	2.34E-28	down
ENSMUSG00000006050	Sra1	-7.00E-01	1.64E-01	no	-1.20E+00	7.68E-04	down
ENSMUSG00000048799	Cep120	-3.68E+00	1.26E-17	down	-3.39E+00	1.75E-17	down
ENSMUSG00000024622	Hmgxb3	-3.10E+00	2.86E-04	down	-2.96E+00	2.11E-03	no
ENSMUSG00000024513	Mbd2	-3.18E+00	6.77E-08	down	-3.39E+00	3.82E-06	down
ENSMUSG00000061802	Armc4	-3.72E+00	4.89E-18	down	-2.07E+00	1.47E-04	down
ENSMUSG00000033323	Ctdp1	-1.56E+00	1.17E-05	down	-3.26E-01	6.57E-01	no
ENSMUSG00000024646	Cyb5	2.65E+00	2.37E-07	up	-7.61E-02	9.16E-01	no
ENSMUSG00000024287	Thoc1	-2.43E+00	9.25E-12	down	-3.21E+00	5.23E-26	down
ENSMUSG00000042211	Fbxo38	-3.20E+00	5.66E-16	down	-3.28E+00	2.57E-14	down
ENSMUSG00000024270	Slc39a6	-2.39E+00	1.76E-11	down	-2.78E+00	1.94E-22	down
ENSMUSG00000058925	1700011I03Rik	-1.47E+00	2.28E-33	down	-9.72E-01	7.50E-19	no
ENSMUSG00000024644	Cndp2	-1.46E+00	7.32E-05	down	-2.41E+00	3.87E-13	down
ENSMUSG00000057719	Sh3rf2	-2.23E+00	3.87E-13	down	-1.77E-01	8.54E-01	no
ENSMUSG00000024359	Hspa9	-2.46E+00	1.32E-35	down	-2.44E+00	1.83E-37	down
ENSMUSG00000060450	Rnf14	-2.61E+00	1.62E-10	down	-2.81E+00	5.50E-10	down
ENSMUSG00000024610	Cd74	7.37E+00	1.35E-187	up	7.54E+00	1.01E-30	up
ENSMUSG00000014504	Srp19	1.80E+00	2.56E-13	up	6.62E-01	2.13E-03	no
ENSMUSG00000040560	Wdr7	-2.68E+00	5.18E-03	no	-3.89E+00	1.75E-10	down
ENSMUSG00000035394	Ccdc11	-2.12E+00	5.36E-28	down	-1.72E+00	6.89E-17	down
ENSMUSG00000024430	Cabyr	-2.63E+00	1.06E-107	down	3.93E-02	9.21E-01	no
ENSMUSG00000024566	Atp9b	-2.77E+00	1.21E-15	down	-1.71E+00	1.93E-06	down
ENSMUSG00000024400	Wdr33	-1.74E+00	4.53E-05	down	-1.97E+00	1.35E-06	down
ENSMUSG00000014503	Pkd2l2	-1.43E+00	9.62E-04	down	-1.40E+00	2.64E-04	down
ENSMUSG00000037058	Paip2	-1.52E+00	5.56E-18	down	-1.01E+00	3.57E-05	down
ENSMUSG00000037013	Ss18	-1.15E+00	4.73E-02	no	-1.67E+00	8.77E-04	down
ENSMUSG00000024493	Lars	-9.25E-01	1.69E-01	no	-2.12E+00	3.11E-06	down
ENSMUSG00000024304	Cdh2	-1.69E+00	5.35E-02	no	-2.23E+00	2.27E-05	down
ENSMUSG00000024302	Dtna	4.16E+00	5.52E-09	up	4.69E+00	1.02E-12	up
ENSMUSG00000024382	Ercc3	-2.47E+00	1.22E-08	down	-2.21E+00	1.94E-07	down
ENSMUSG00000024424	Ttc39c	-2.96E+00	7.55E-07	down	-2.67E+00	4.88E-07	down
ENSMUSG00000024381	Bin1	5.82E+00	1.67E-08	up	6.54E+00	9.85E-15	up
ENSMUSG00000024240	Epc1	-1.55E+00	8.85E-03	no	-2.03E+00	3.21E-05	down
ENSMUSG00000024507	Hsd17b4	-1.39E+00	2.11E-02	no	-2.07E+00	1.27E-05	down

ENSMUSG00000025428	Atp5a1	-2.97E-01	1.63E-01	no	-1.40E+00	6.00E-22	down
ENSMUSG00000041238	Rbbp8	-2.10E+00	4.61E-05	down	-3.47E+00	1.51E-12	down
ENSMUSG00000003779	Kif20a	-2.59E+00	6.84E-10	down	-3.00E+00	1.66E-14	down
ENSMUSG00000024454	Hdac3	-1.04E+00	6.29E-02	no	-1.64E+00	7.88E-05	down
ENSMUSG00000024491	Rbm27	-1.44E+00	7.45E-06	down	-1.49E+00	5.66E-06	down
ENSMUSG00000025423	Pias2	-2.74E+00	1.87E-53	down	-2.21E+00	1.04E-31	down
ENSMUSG00000024260	Sap130	-3.94E+00	2.37E-24	down	-2.36E+00	3.02E-05	down
ENSMUSG00000024500	Ppp2r2b	-4.96E+00	6.90E-10	down	-4.08E+00	1.94E-02	no
ENSMUSG00000033382	Trappc8	-2.03E+00	1.15E-02	no	-4.07E+00	4.23E-09	down
ENSMUSG00000024498	Tcerg1	-3.10E-01	5.94E-01	no	-1.79E+00	1.15E-07	down
ENSMUSG00000024306	Ccdc178	-3.12E+00	2.76E-39	down	-2.64E+00	2.31E-25	down
ENSMUSG00000024542	Cep192	-2.33E+00	1.70E-02	no	-3.68E+00	7.49E-07	down
ENSMUSG00000038425	Poli	-2.76E+00	1.06E-08	down	-1.21E+00	1.19E-01	no
ENSMUSG00000079608	Stard6	-3.39E+00	2.18E-33	down	-3.14E+00	5.51E-35	down
ENSMUSG00000024293	Esco1	-2.92E-01	7.54E-01	no	-2.67E+00	4.65E-06	down
ENSMUSG00000024501	Dpysl3	-3.57E+00	1.27E-41	down	-7.88E-01	1.90E-01	no
ENSMUSG00000041923	Nol4	-2.54E+00	4.45E-06	down	-8.08E-01	5.02E-01	no
ENSMUSG00000024598	Fbn2	-1.94E-01	8.91E-01	no	-2.11E+00	4.68E-04	down
ENSMUSG00000033632	AW554918	-3.52E+00	3.20E-09	down	-3.05E+00	2.08E-06	down
ENSMUSG00000024294	Mib1	-3.78E+00	1.83E-09	down	-3.84E+00	3.71E-07	down
ENSMUSG00000024576	Csnk1a1	-1.06E+00	2.04E-04	down	-1.41E+00	8.23E-08	down
ENSMUSG00000024231	Cul2	-1.68E+00	5.74E-02	no	-2.40E+00	2.81E-04	down
ENSMUSG00000033628	Pik3c3	-8.44E-01	5.17E-01	no	-2.76E+00	2.28E-04	down
ENSMUSG00000024534	Sncaip	-4.16E+00	6.19E-09	down	-8.08E-01	6.71E-01	no
ENSMUSG00000025420	Katnal2	-2.72E+00	2.61E-13	down	-2.51E+00	3.32E-14	down
ENSMUSG00000025885	Myo5b	-4.43E+00	1.02E-08	down	-9.53E-01	6.03E-01	no
ENSMUSG00000024283	Wac	-2.62E+00	1.12E-08	down	-2.44E+00	6.62E-07	down
ENSMUSG00000024456	Diap1	-1.82E+00	4.55E-02	no	-2.89E+00	1.32E-04	down
ENSMUSG00000044252	Osbpl1a	-3.94E+00	6.68E-11	down	-3.72E+00	1.91E-08	down
ENSMUSG00000063889	Crem	-1.74E+00	3.89E-18	down	-8.32E-01	5.91E-04	no
ENSMUSG00000067872	Ccdc87	-3.11E+00	5.70E-19	down	-2.20E+00	1.39E-10	down
ENSMUSG00000095098	Ccdc85b	6.32E+00	3.57E-14	up	4.15E+00	1.15E-03	no
ENSMUSG00000067608	Pcna-ps2	-2.62E-01	2.42E-01	no	-1.18E+00	5.05E-17	down
ENSMUSG00000090291	Lrrc10b	-5.30E+00	5.00E-09	down	-3.10E+00	9.64E-03	no
ENSMUSG00000044424	Gm9493	-2.21E+00	6.01E-23	down	-3.05E+00	1.77E-61	down
ENSMUSG00000051255	Gm6563	1.89E+00	2.80E-16	up	-9.61E-01	2.46E-12	no
ENSMUSG00000058927	Gm10053	2.19E+00	1.93E-06	up	1.11E+00	5.31E-02	no
ENSMUSG00000040105	Ppapdc2	-3.48E+00	3.58E-04	down	-2.36E+00	2.29E-01	no
ENSMUSG00000074909	Ranbp6	-5.13E+00	2.66E-10	down	-4.21E+00	5.42E-07	down
ENSMUSG00000024815	Trpd52l3	-3.46E+00	6.70E-29	down	-3.31E+00	7.46E-34	down
ENSMUSG00000053536	Cstf2t	-1.75E+00	3.57E-02	no	-3.55E+00	3.98E-13	down
ENSMUSG00000062456	Rpl9-ps6	-5.06E-01	1.08E-01	no	-2.40E+00	5.75E-36	down
ENSMUSG00000090369	4933411K16Rik	-4.48E+00	0.00E+00	down	-2.24E+00	2.65E-33	down
ENSMUSG00000071497	Nutf2-ps1	2.70E+00	1.10E-04	up	1.35E+00	3.86E-02	no
ENSMUSG00000067038	Rps12-ps3	-2.12E+00	1.86E-10	down	-3.61E+00	3.95E-34	down
ENSMUSG00000069475	Gm6020	-5.25E+00	2.06E-04	down	-4.37E+00	1.18E-02	no
ENSMUSG00000079382	Gm3443	3.30E+00	2.09E-38	up	1.82E+00	9.61E-52	up
ENSMUSG00000050957	Insl6	-1.31E+00	1.57E-48	down	-1.06E+00	1.76E-47	down
ENSMUSG00000079254	Itprip	-4.17E+00	4.82E-13	down	-3.54E+00	5.29E-09	down
ENSMUSG00000049562	Ap5b1	-2.60E+00	5.37E-02	no	-2.93E+00	2.19E-04	down
ENSMUSG00000035885	Cox8a	3.74E+00	4.02E-50	up	2.75E+00	2.21E-22	up
ENSMUSG00000090840	1700092M07Rik	-1.27E+00	3.04E-45	down	1.07E-01	6.79E-01	no
ENSMUSG00000071661	Zbtb3	-2.77E+00	2.04E-12	down	-2.77E+00	2.43E-21	down
ENSMUSG00000071654	AI462493	1.94E+00	1.38E-13	up	1.07E+00	9.64E-11	up
ENSMUSG00000075010	AW112010	4.89E+00	3.24E-06	up	1.61E+00	3.96E-01	no
ENSMUSG00000025171	Ubtd1	-3.35E+00	3.15E-71	down	-2.72E+00	1.17E-55	down
ENSMUSG00000024668	Sdhaf2	-3.05E-01	7.66E-01	no	-2.14E+00	4.75E-04	down
ENSMUSG00000024683	Mrpl16	-1.17E+00	1.75E-03	no	-1.82E+00	4.68E-14	down
ENSMUSG00000047044	D030056L22Rik	5.35E-02	9.07E-01	no	-2.45E+00	3.69E-51	down
ENSMUSG00000047368	Abhd17b	-2.11E+00	1.00E-22	down	-1.66E+00	4.87E-12	down
ENSMUSG00000035372	1810055G02Rik	-2.15E+00	8.85E-27	down	-1.55E+00	1.42E-15	down
ENSMUSG00000040414	Slc25a28	-2.10E+00	1.63E-08	down	-3.33E+00	5.00E-35	down
ENSMUSG00000025066	Sfr1	1.19E+00	5.16E-09	up	-1.71E-01	5.08E-01	no
ENSMUSG00000039330	Tsga10ip	-1.44E+00	5.23E-32	down	-7.83E-01	6.31E-10	no

ENSMUSG00000084957	Bbip1	-9.23E-01	2.76E-04	no	-1.57E+00	1.81E-17	down
ENSMUSG00000047423	AI837181	-2.80E+00	2.61E-15	down	-2.60E+00	4.35E-13	down
ENSMUSG00000024912	Fosl1	2.58E+00	4.51E-06	up	1.21E+00	3.05E-01	no
ENSMUSG00000024922	Ovol1	-3.07E+00	1.71E-14	down	-2.60E+00	1.59E-15	down
ENSMUSG00000056612	Ppp1r14b	1.05E+00	7.69E-06	up	7.67E-01	7.39E-04	no
ENSMUSG00000091558	Gm17227	-1.40E+00	1.86E-06	down	-1.20E+00	2.39E-08	down
ENSMUSG00000024661	Fth1	5.37E+00	0.00E+00	up	1.01E+00	4.56E-51	up
ENSMUSG00000048832	Vps37c	-1.05E+00	5.54E-02	no	-2.00E+00	1.32E-07	down
ENSMUSG00000054523	Ms4a5	-1.87E+00	4.84E-77	down	1.25E-01	6.48E-01	no
ENSMUSG00000049164	Zfp518a	-2.38E-01	8.46E-01	no	-3.03E+00	1.66E-04	down
ENSMUSG00000049670	Morn4	-2.12E+00	1.11E-25	down	-1.96E+00	2.14E-34	down
ENSMUSG00000057506	Bloc1s2a	3.63E-01	4.56E-01	no	-1.37E+00	9.40E-10	down
ENSMUSG00000024902	Mrpl11	-9.71E-01	9.56E-03	no	-1.57E+00	6.76E-09	down
ENSMUSG00000074746	Pdzd8	-4.80E+00	1.80E-26	down	-3.14E+00	3.11E-05	down
ENSMUSG00000047733	BC048609	-8.34E-01	1.36E-05	no	1.27E+00	8.55E-05	up
ENSMUSG00000079437	Tmem179b	2.83E+00	1.38E-06	up	1.65E-02	9.85E-01	no
ENSMUSG00000033053	1700028P14Rik	-3.73E+00	1.24E-215	down	-1.74E+00	9.03E-24	down
ENSMUSG00000025035	Arl3	-2.64E+00	3.46E-108	down	-2.68E+00	9.49E-195	down
ENSMUSG00000024870	Rab1b	-1.03E+00	2.00E-06	down	-1.48E+00	3.31E-16	down
ENSMUSG00000025024	Smndc1	-1.67E+00	4.14E-07	down	-1.88E+00	5.18E-08	down
ENSMUSG00000024844	Banf1	3.00E+00	5.81E-75	up	1.20E+00	2.23E-22	up
ENSMUSG00000024970	AI846148	-1.75E+00	6.91E-06	down	-3.60E-01	7.00E-01	no
ENSMUSG00000024729	1700017D01Rik	-3.19E+00	1.81E-117	down	-2.65E+00	3.83E-78	down
ENSMUSG00000074925	Ptar1	-1.89E+00	5.10E-06	down	-1.44E+00	2.80E-04	down
ENSMUSG00000024780	Cdc37l1	-1.58E+00	1.33E-05	down	-1.95E+00	2.55E-09	down
ENSMUSG00000059734	Ndufs8	1.66E+00	1.07E-12	up	1.14E-01	6.86E-01	no
ENSMUSG00000060803	Gstp1	5.53E+00	5.51E-11	up	2.40E+00	7.82E-03	no
ENSMUSG00000040385	Ppp1ca	4.30E+00	1.72E-27	up	2.82E+00	3.37E-10	up
ENSMUSG00000006456	Rbm14	-1.07E+00	1.09E-01	no	-1.78E+00	2.52E-04	down
ENSMUSG00000043969	Emx2	-4.15E+00	2.81E-02	no	-5.69E+00	3.72E-04	down
ENSMUSG00000057240	Ms4a13	-2.74E+00	2.10E-55	down	-2.16E+00	4.79E-37	down
ENSMUSG00000024764	Naa40	-1.33E+00	8.07E-03	no	-2.90E+00	1.09E-19	down
ENSMUSG00000071662	Polr2g	2.12E+00	3.95E-14	up	-5.36E-02	8.75E-01	no
ENSMUSG00000041857	Oosp1	3.35E+00	4.07E-02	no	7.30E+00	2.33E-07	up
ENSMUSG00000024696	Lpxn	-1.25E+00	1.62E-02	no	-2.66E+00	5.21E-23	down
ENSMUSG00000035783	Acta2	5.03E+00	1.39E-36	up	3.99E+00	4.90E-06	up
ENSMUSG00000025218	Poll	-3.06E+00	2.68E-23	down	-2.77E+00	2.25E-24	down
ENSMUSG00000056209	Npm3	-2.55E+00	1.76E-08	down	-3.45E+00	3.75E-21	down
ENSMUSG00000071645	Tut1	-2.50E+00	2.01E-12	down	-2.16E+00	2.03E-11	down
ENSMUSG00000034820	Cpsf7	-1.09E+00	1.69E-01	no	-2.84E+00	6.07E-09	down
ENSMUSG00000013662	Atad1	-3.25E+00	9.16E-29	down	-1.95E+00	5.37E-06	down
ENSMUSG00000024768	Lipf	-3.57E+00	5.26E-04	down	-5.11E-01	8.61E-01	no
ENSMUSG00000024885	Aldh3b1	-3.21E-01	2.44E-01	no	1.53E+00	7.55E-08	up
ENSMUSG00000025050	Pcgf6	-5.37E-02	9.06E-01	no	-1.78E+00	1.15E-18	down
ENSMUSG00000080268	Brms1	-1.81E+00	2.36E-52	down	-1.06E+00	1.98E-16	down
ENSMUSG00000024883	Rin1	-3.54E+00	1.22E-04	down	-1.45E+00	4.29E-01	no
ENSMUSG00000024911	Fibp	7.66E-01	1.30E-01	no	-1.19E+00	5.53E-05	down
ENSMUSG00000024654	Asrgl1	-1.25E+00	1.48E-46	down	-1.15E+00	3.04E-60	down
ENSMUSG00000055895	Plac1l	3.68E+00	1.44E-02	no	6.35E+00	3.54E-07	up
ENSMUSG00000071604	Fam189a2	-2.61E+00	2.87E-04	down	4.71E-02	9.84E-01	no
ENSMUSG00000024800	Rpp30	-7.12E-01	2.10E-02	no	-1.36E+00	1.85E-11	down
ENSMUSG00000024824	Rad9a	-8.47E-01	4.81E-01	no	-2.45E+00	7.92E-04	down
ENSMUSG00000025228	Actr1a	-2.58E+00	2.74E-46	down	-2.50E+00	2.55E-57	down
ENSMUSG00000024982	Zdhhc6	-1.17E+00	6.79E-02	no	-2.09E+00	3.12E-04	down
ENSMUSG00000024927	Rela	2.20E-01	7.22E-01	no	-1.58E+00	2.84E-05	down
ENSMUSG00000024772	Ehd1	-3.07E+00	2.58E-77	down	-2.59E+00	3.23E-42	down
ENSMUSG00000036278	Macrod1	-1.61E+00	1.22E-04	down	-1.28E+00	2.11E-03	no
ENSMUSG00000071573	Rnls	-4.39E-01	8.67E-02	no	-1.67E+00	5.71E-37	down
ENSMUSG00000024990	Rbp4	3.17E+00	2.97E-04	up	1.57E+00	2.57E-01	no
ENSMUSG00000042532	Golga7b	-3.21E+00	4.45E-04	down	2.17E-02	9.93E-01	no
ENSMUSG00000038498	Catsper1	-6.13E+00	1.45E-77	down	-3.24E+00	1.93E-07	down
ENSMUSG00000004054	Map3k11	-2.10E+00	1.16E-03	no	-2.77E+00	3.72E-07	down
ENSMUSG00000026931	1700019N19Rik	-2.87E+00	3.30E-123	down	-8.10E-01	8.82E-05	no
ENSMUSG00000056629	Fkbp2	2.03E+00	8.49E-10	up	2.16E-02	9.65E-01	no

ENSMUSG00000053279	Aldh1a1	2.82E+00	6.24E-13	up	1.54E+00	4.48E-04	up
ENSMUSG00000024897	Apba1	-3.76E+00	4.14E-07	down	-4.57E+00	9.39E-22	down
ENSMUSG00000048720	Tbc1d12	-1.01E+00	2.76E-01	no	-2.51E+00	5.82E-06	down
ENSMUSG00000015176	Nolc1	-9.11E-01	1.43E-01	no	-2.04E+00	4.81E-05	down
ENSMUSG00000003555	Cyp17a1	1.16E+00	4.46E-01	no	3.08E+00	3.57E-06	up
ENSMUSG00000035179	Ppp1r32	-3.08E+00	7.93E-123	down	-1.18E+00	1.12E-10	down
ENSMUSG00000024687	Osbp	-3.88E+00	4.14E-22	down	-2.40E+00	1.33E-04	down
ENSMUSG00000083282	Ctsf	3.41E+00	4.30E-05	up	4.97E-01	6.23E-01	no
ENSMUSG00000024926	Kat5	-2.14E+00	1.29E-13	down	-2.26E+00	3.77E-29	down
ENSMUSG00000048029	Eno4	-2.78E+00	4.33E-19	down	-2.15E+00	2.65E-12	down
ENSMUSG00000038274	Fau	3.51E+00	7.02E-38	up	2.65E+00	5.31E-30	up
ENSMUSG00000024966	Stip1	-1.14E+00	7.38E-10	down	-1.14E+00	3.23E-08	down
ENSMUSG00000071659	Hnrnpul2	-1.66E+00	1.16E-01	no	-3.83E+00	6.18E-09	down
ENSMUSG00000037418	Best1	-3.30E+00	1.58E-78	down	-5.33E-01	2.10E-01	no
ENSMUSG00000024878	Cbwd1	-1.19E+00	4.77E-03	no	-1.83E+00	9.65E-07	down
ENSMUSG00000025016	Tm9sf3	-2.34E+00	6.37E-07	down	-3.07E+00	4.47E-10	down
ENSMUSG00000025176	Hoga1	-3.88E+00	2.44E-12	down	-7.82E-01	6.20E-01	no
ENSMUSG00000025220	Mgea5	-2.77E+00	1.20E-15	down	-1.96E+00	3.44E-05	down
ENSMUSG00000024791	Cdca5	-1.90E+00	1.45E-05	down	-1.39E+00	1.46E-03	no
ENSMUSG00000034371	Dak	-2.83E+00	1.00E-24	down	-3.06E+00	7.76E-69	down
ENSMUSG00000013663	Pten	-3.16E+00	4.57E-07	down	-2.81E+00	1.56E-04	down
ENSMUSG00000006464	Bbs1	-2.34E+00	6.34E-02	no	-3.51E+00	3.79E-06	down
ENSMUSG00000033478	Fam160b1	-2.88E+00	5.17E-05	down	-3.27E+00	2.61E-08	down
ENSMUSG00000024941	Scyl1	-8.08E-01	1.52E-01	no	-1.93E+00	7.22E-06	down
ENSMUSG00000024786	1700123I01Rik	5.30E-01	4.99E-01	no	-1.47E+00	3.09E-04	down
ENSMUSG00000024805	Pcgf5	2.07E+00	1.17E-12	up	7.55E-02	8.27E-01	no
ENSMUSG00000024856	Cdk2ap2	4.79E+00	3.43E-16	up	3.21E+00	4.17E-05	up
ENSMUSG00000033760	Rbm4b	-2.06E+00	2.23E-08	down	-1.04E+00	7.94E-02	no
ENSMUSG00000046585	Ccdc147	-5.13E+00	2.02E-71	down	-2.47E+00	1.04E-07	down
ENSMUSG00000063904	Dpp3	-2.29E+00	6.77E-12	down	-2.16E+00	5.52E-14	down
ENSMUSG00000025025	Mxi1	-2.65E+00	2.97E-05	down	-3.07E+00	8.16E-07	down
ENSMUSG00000024660	Incenp	-2.12E+00	1.29E-07	down	-2.17E+00	6.76E-08	down
ENSMUSG00000024953	Prdx5	4.73E-01	8.25E-02	no	-1.06E+00	1.30E-10	down
ENSMUSG00000010095	Slc3a2	1.97E+00	6.19E-10	up	5.57E-01	5.98E-02	no
ENSMUSG00000071655	Ubxn1	-1.59E-01	3.90E-01	no	-1.25E+00	2.22E-25	down
ENSMUSG00000039148	Sart1	-1.64E+00	3.44E-03	no	-2.21E+00	2.37E-06	down
ENSMUSG00000024955	Esrra	-2.43E+00	1.43E-02	no	-3.61E+00	3.59E-07	down
ENSMUSG00000024853	Sf3b2	-1.16E+00	8.81E-10	down	-1.68E+00	5.18E-28	down
ENSMUSG00000079478	Sssca1	1.34E+00	3.13E-04	up	1.22E+00	5.55E-05	up
ENSMUSG00000010097	Nxf1	-1.97E+00	1.22E-22	down	-2.36E+00	3.20E-38	down
ENSMUSG00000012443	Kif11	-1.17E+00	1.54E-01	no	-2.71E+00	1.43E-04	down
ENSMUSG00000024981	Acsl5	-2.93E+00	1.40E-05	down	-1.53E+00	1.91E-01	no
ENSMUSG00000024991	Eif3a	-2.42E+00	1.73E-26	down	-1.48E+00	4.06E-07	down
ENSMUSG00000024812	Tjp2	-3.23E+00	2.58E-06	down	-3.08E-01	8.64E-01	no
ENSMUSG00000024943	Smc5	-3.26E+00	3.37E-31	down	-3.04E+00	4.96E-25	down
ENSMUSG00000025069	Gsto2	-2.42E+00	9.08E-54	down	-1.70E+00	2.44E-34	down
ENSMUSG00000024855	Pacs1	-3.10E+00	1.40E-36	down	-2.19E+00	1.68E-14	down
ENSMUSG00000033417	Cacul1	-3.74E+00	7.24E-23	down	-1.83E+00	5.92E-03	no
ENSMUSG00000024728	1700025F22Rik	-1.07E+00	2.77E-17	down	-7.86E-01	8.11E-10	no
ENSMUSG00000024827	Gldc	-3.01E+00	2.69E-01	no	-5.04E+00	2.13E-04	down
ENSMUSG00000024816	Frmd8	-1.69E+00	5.51E-03	no	-2.37E+00	2.02E-07	down
ENSMUSG00000071650	Ganab	-2.23E+00	4.72E-07	down	-2.70E+00	3.46E-08	down
ENSMUSG00000024759	Atl3	-4.01E+00	6.49E-27	down	-2.24E+00	9.59E-04	down
ENSMUSG00000024740	Ddb1	-2.26E+00	2.53E-21	down	-2.83E+00	1.60E-49	down
ENSMUSG00000047810	Ccdc88b	-1.79E+00	3.64E-02	no	-2.80E+00	7.98E-08	down
ENSMUSG00000024989	Cep55	-2.51E+00	1.69E-05	down	-3.17E+00	8.60E-12	down
ENSMUSG00000037578	Pkd2l1	-6.12E-01	6.70E-01	no	-3.15E+00	5.12E-08	down
ENSMUSG00000025217	Btrc	-2.15E+00	5.88E-07	down	-2.01E+00	1.50E-07	down
ENSMUSG00000024695	Zfp91	-2.59E+00	3.91E-12	down	-2.83E+00	2.31E-17	down
ENSMUSG00000025204	Ndufb8	1.65E+00	1.00E-32	up	1.68E+00	6.98E-48	up
ENSMUSG00000024795	Kif20b	-1.69E+00	1.32E-02	no	-2.82E+00	3.54E-07	down
ENSMUSG00000024758	Rtn3	-1.01E+00	4.24E-05	down	-1.72E+00	4.42E-16	down
ENSMUSG00000024974	Smc3	-1.54E+00	2.58E-05	down	-3.09E-01	7.70E-01	no
ENSMUSG00000071644	Eef1g	-2.39E+00	3.04E-08	down	-2.30E+00	1.72E-06	down

ENSMUSG00000046138	9930021J03Rik	-1.54E+00	6.64E-02	no	-2.80E+00	2.85E-06	down
ENSMUSG00000035049	Rrp12	-2.24E+00	3.01E-06	down	-1.60E+00	9.29E-03	no
ENSMUSG00000024958	Gpr137	-2.19E+00	9.82E-12	down	-2.07E+00	1.74E-15	down
ENSMUSG00000024993	Fam45a	-2.83E+00	5.73E-07	down	-3.06E+00	1.15E-11	down
ENSMUSG00000024776	Stambpl1	-3.40E+00	1.24E-09	down	-6.13E-01	7.19E-01	no
ENSMUSG00000037126	Psd	-3.37E+00	3.80E-08	down	-2.46E+00	3.09E-02	no
ENSMUSG00000024833	Pola2	-4.28E-02	9.52E-01	no	-1.30E+00	9.91E-04	down
ENSMUSG00000024952	Rps6ka4	-2.72E+00	7.65E-04	down	-3.18E+00	2.56E-05	down
ENSMUSG00000024975	Pdcd4	-1.28E+00	8.98E-12	down	-1.11E+00	2.49E-10	down
ENSMUSG00000040565	Btaf1	-2.09E+00	6.10E-06	down	-2.89E+00	3.54E-14	down
ENSMUSG00000024937	Ehbp1l1	-2.95E+00	4.04E-14	down	-2.83E+00	7.70E-13	down
ENSMUSG00000024727	Trpm6	-4.33E+00	2.46E-09	down	-1.69E+00	3.10E-01	no
ENSMUSG00000025154	Arhgap19	-2.43E+00	4.25E-08	down	-2.94E+00	4.77E-18	down
ENSMUSG00000024835	Coro1b	-6.84E-01	1.18E-01	no	-1.43E+00	5.41E-06	down
ENSMUSG00000071646	Mta2	-1.55E+00	5.72E-03	no	-2.52E+00	1.92E-08	down
ENSMUSG00000025026	Add3	-1.17E+00	3.71E-02	no	-2.26E+00	2.91E-08	down
ENSMUSG00000024735	Prpf19	-1.56E+00	1.85E-01	no	-3.24E+00	3.72E-05	down
ENSMUSG00000025231	Sufu	-3.73E+00	2.41E-14	down	-2.39E+00	1.42E-03	no
ENSMUSG00000035173	A630007B06Rik	-4.17E+00	1.56E-17	down	-1.75E+00	5.44E-02	no
ENSMUSG00000024914	Drap1	-1.53E+00	1.30E-28	down	-2.61E+00	1.25E-122	down
ENSMUSG00000025198	Erlin1	-1.63E+00	1.12E-02	no	-3.16E+00	8.26E-15	down
ENSMUSG00000024826	Dpf2	-7.02E-01	2.90E-01	no	-1.84E+00	9.12E-04	down
ENSMUSG00000037916	Ndufv1	-4.79E-01	8.15E-02	no	-1.15E+00	4.90E-08	down
ENSMUSG00000024663	Rab3il1	-3.32E+00	5.93E-71	down	-5.92E-01	1.41E-01	no
ENSMUSG00000056999	Ide	-4.15E+00	1.82E-36	down	-3.92E+00	5.58E-34	down
ENSMUSG00000025041	Nt5c2	-1.36E+00	5.37E-03	no	-1.73E+00	2.92E-04	down
ENSMUSG00000024913	Lrp5	-3.92E+00	1.94E-07	down	-1.72E+00	4.12E-01	no
ENSMUSG00000064202	4430402I18Rik	-5.35E+00	4.12E-107	down	-2.93E+00	5.89E-12	down
ENSMUSG00000024908	Ppp6r3	-1.65E+00	3.06E-03	no	-2.45E+00	1.03E-06	down
ENSMUSG00000046230	Vps13a	-3.58E+00	6.08E-15	down	-2.75E+00	1.46E-07	down
ENSMUSG00000040451	Sgms1	-3.22E+00	1.31E-13	down	-2.29E+00	8.25E-06	down
ENSMUSG00000024862	Klc2	-4.94E+00	7.06E-14	down	-5.43E+00	6.41E-23	down
ENSMUSG00000024807	Syvn1	-9.19E-01	3.20E-01	no	-2.89E+00	4.60E-09	down
ENSMUSG00000024843	Chka	-9.87E-01	1.23E-01	no	-1.88E+00	1.09E-04	down
ENSMUSG00000024906	Mus81	-2.55E+00	4.99E-05	down	-2.26E+00	8.76E-05	down
ENSMUSG00000024773	Atg2a	-2.09E+00	1.94E-03	no	-2.66E+00	6.81E-05	down
ENSMUSG00000039652	Cpeb3	-4.84E+00	2.57E-06	down	-3.56E+00	7.24E-02	no
ENSMUSG00000044948	Wdr96	-4.72E+00	1.08E-49	down	-4.12E+00	9.78E-44	down
ENSMUSG00000025081	Tdrd1	-5.97E-01	2.60E-01	no	-2.32E+00	4.58E-22	down
ENSMUSG00000024949	Sf1	-2.86E+00	7.29E-24	down	-2.46E+00	5.75E-14	down
ENSMUSG00000010110	Stx5a	-1.99E+00	4.61E-05	down	-1.90E+00	3.17E-04	down
ENSMUSG00000025027	Xpnpep1	-3.43E+00	5.76E-16	down	-7.66E-01	4.10E-01	no
ENSMUSG00000025224	Gbf1	-2.37E+00	1.66E-11	down	-2.44E+00	2.39E-11	down
ENSMUSG00000024969	Mark2	-1.03E+00	2.83E-01	no	-3.28E+00	1.80E-13	down
ENSMUSG00000024921	Smarca2	-2.45E+00	3.00E-17	down	-1.86E+00	4.05E-12	down
ENSMUSG00000075272	Ttc30a2	-2.02E+00	2.95E-21	down	-2.10E+00	1.22E-32	down
ENSMUSG00000075271	Ttc30a1	-5.12E-01	5.39E-01	no	-2.34E+00	9.57E-11	down
ENSMUSG00000050114	Prdx6b	-1.42E+00	1.01E-42	down	-1.15E+00	2.01E-34	down
ENSMUSG00000068809	Olfr1188	-4.24E+00	1.42E-05	down	-3.68E+00	1.11E-04	down
ENSMUSG00000074881	Mageb3	-3.21E+00	1.03E-01	no	-4.01E+00	1.46E-04	down
ENSMUSG00000074807	Gm10762	-1.22E+00	2.31E-03	no	-2.05E+00	1.17E-18	down
ENSMUSG00000027431	Scp2d1	-3.81E-01	9.15E-08	no	1.06E+00	1.44E-16	up
ENSMUSG00000078129	Actl10	-2.77E+00	2.05E-11	down	2.34E-01	8.36E-01	no
ENSMUSG00000097602	4930519P11Rik	-2.35E+00	4.26E-08	down	-1.36E+00	1.06E-02	no
ENSMUSG00000070708	Gtsf1l	-3.74E+00	0.00E+00	down	-1.47E+00	8.58E-24	down
ENSMUSG00000056501	Cebpb	9.12E+00	3.55E-21	up	6.88E+00	7.29E-05	up
ENSMUSG00000067581	AY702102	-3.11E+00	4.05E-44	down	-5.52E-01	2.53E-01	no
ENSMUSG00000055882	Abhd16b	-4.59E+00	6.72E-13	down	-3.40E+00	9.15E-03	no
ENSMUSG00000032671	A930018P22Rik	5.11E-01	3.25E-02	no	1.11E+00	9.06E-06	up
ENSMUSG00000027133	Nop10	1.51E+00	8.77E-44	up	1.04E+00	7.95E-37	up
ENSMUSG00000049692	Tmem239	-4.18E+00	2.90E-16	down	-3.56E+00	9.31E-05	down
ENSMUSG00000050645	Defb19	4.77E+00	2.58E-76	up	3.98E+00	9.03E-171	up
ENSMUSG00000044863	Defb36	2.60E+00	2.78E-05	up	2.63E+00	2.90E-15	up
ENSMUSG00000046229	Scand1	1.62E+00	8.90E-29	up	1.30E+00	2.24E-17	up

ENSMUSG00000070529	Wfdc10	3.87E+00	6.55E-09	up	3.68E+00	4.20E-21	up
ENSMUSG00000045822	Zswim3	-1.79E+00	2.92E-06	down	-1.50E+00	4.21E-05	down
ENSMUSG00000027505	Fam209	6.85E-02	4.80E-01	no	1.76E+00	7.45E-42	up
ENSMUSG00000039164	Naif1	-2.29E+00	1.93E-07	down	-1.28E-01	9.16E-01	no
ENSMUSG00000049950	Rpp38	-2.36E+00	5.38E-13	down	-2.43E+00	1.69E-15	down
ENSMUSG00000040794	C1qtnf4	2.21E+00	5.95E-208	up	2.47E+00	0.00E+00	up
ENSMUSG00000034226	Rhov	-1.53E+00	2.11E-02	no	-2.30E+00	8.44E-06	down
ENSMUSG00000074890	Lcmt2	6.06E+00	7.49E-07	up	2.78E+00	4.05E-03	no
ENSMUSG00000027445	Cst9	2.04E+00	4.38E-05	up	1.24E+00	1.08E-07	up
ENSMUSG00000078957	1700060C20Rik	1.85E+00	1.16E-05	up	1.62E+00	1.26E-05	up
ENSMUSG00000051769	Wfdc15a	1.61E+00	6.79E-79	up	9.71E-01	8.01E-53	no
ENSMUSG00000078935	1700025C18Rik	-5.54E+00	5.98E-10	down	-3.84E+00	1.61E-02	no
ENSMUSG00000027221	Chst1	-7.27E-01	2.59E-01	no	-2.84E+00	1.04E-23	down
ENSMUSG00000060802	B2m	5.27E+00	1.01E-202	up	2.85E+00	4.59E-23	up
ENSMUSG00000036924	Cst13	-2.83E+00	4.28E-59	down	-1.67E+00	3.01E-07	down
ENSMUSG00000061525	4921509C19Rik	-3.86E+00	8.94E-08	down	-1.04E+00	6.38E-01	no
ENSMUSG00000027518	1700021F07Rik	-4.20E+00	5.36E-188	down	-3.40E+00	1.29E-84	down
ENSMUSG00000056436	Cyct	4.83E-01	4.60E-05	no	-1.04E+00	4.00E-50	down
ENSMUSG00000075015	Gm10801	1.40E+01	1.39E-238	up	6.33E+00	3.98E-13	up
ENSMUSG00000027409	1700020A23Rik	-4.08E+00	3.38E-26	down	-2.09E+00	2.42E-04	down
ENSMUSG00000027350	Chgb	-3.22E+00	6.96E-04	down	-1.86E+00	1.91E-02	no
ENSMUSG00000017720	Trp53tg5	-3.68E+00	1.28E-66	down	-2.17E+00	6.77E-08	down
ENSMUSG00000017764	Zswim1	-1.79E+00	6.30E-04	down	-1.73E+00	3.75E-04	down
ENSMUSG00000017767	Spata25	-1.19E+00	9.67E-07	down	7.93E-01	6.71E-02	no
ENSMUSG00000047841	BC051628	-1.08E+00	3.15E-18	down	6.14E-01	1.78E-03	no
ENSMUSG00000044916	1700029I15Rik	3.38E-01	5.63E-02	no	2.09E+00	1.05E-16	up
ENSMUSG00000075014	Gm10800	1.62E+01	0.00E+00	up	1.09E+01	1.11E-114	up
ENSMUSG00000027443	Cst12	1.91E+00	9.16E-05	up	1.21E+00	3.70E-08	up
ENSMUSG00000079005	Gm14147	-5.18E+00	1.36E-08	down	-1.84E+00	4.96E-01	no
ENSMUSG00000016256	Ctsz	5.15E+00	3.15E-14	up	3.93E+00	3.99E-04	up
ENSMUSG00000036504	Phpt1	1.40E+00	1.35E-05	up	1.04E+00	8.28E-07	up
ENSMUSG00000026882	4930568D16Rik	-2.26E+00	3.41E-07	down	1.04E+00	2.72E-01	no
ENSMUSG00000053475	Tnfaip6	5.18E+00	2.35E-15	up	3.26E+00	7.29E-02	no
ENSMUSG00000027076	Timm10	2.06E+00	2.40E-08	up	7.91E-01	7.62E-03	no
ENSMUSG00000079602	9230102O04Rik	-7.61E+00	2.45E-05	down	#VALUE!	NA	no
ENSMUSG00000041358	Nutm1	-5.05E+00	2.14E-26	down	-2.20E+00	2.23E-02	no
ENSMUSG00000074825	Itpripl1	-3.69E+00	3.34E-06	down	-2.48E+00	6.90E-02	no
ENSMUSG00000037885	Stk35	-3.13E+00	3.69E-16	down	-2.44E+00	8.03E-09	down
ENSMUSG00000037307	Banf2	-1.58E+00	7.52E-23	down	-4.21E-01	2.68E-01	no
ENSMUSG00000079006	Gm14151	-4.58E+00	5.84E-06	down	-2.28E+00	4.99E-01	no
ENSMUSG00000074575	Kcng1	-4.44E+00	4.13E-03	no	-5.23E+00	1.73E-04	down
ENSMUSG00000016252	Atp5e	1.55E+00	7.31E-13	up	2.57E-01	1.75E-01	no
ENSMUSG00000038605	Samd10	-1.86E+00	4.26E-05	down	-2.21E+00	2.07E-08	down
ENSMUSG00000045794	4930526D03Rik	-1.75E+00	7.26E-47	down	-7.82E-01	2.48E-06	no
ENSMUSG00000026963	Tmem210	-9.63E-01	1.98E-14	no	1.31E+00	1.44E-10	up
ENSMUSG00000026880	Stom	-5.86E-01	4.27E-01	no	-2.49E+00	1.12E-09	down
ENSMUSG00000027102	Hoxd8	-1.88E+00	5.23E-07	down	-1.58E+00	1.77E-04	down
ENSMUSG00000025783	4930412O13Rik	-6.87E+00	1.91E-11	down	-4.62E+00	4.67E-03	no
ENSMUSG00000027447	Cst3	6.39E+00	0.00E+00	up	3.62E+00	1.18E-69	up
ENSMUSG00000027459	Fam110a	-3.15E+00	6.53E-23	down	-1.38E+00	2.20E-02	no
ENSMUSG00000032802	Srxn1	-2.88E+00	2.41E-07	down	-2.12E+00	1.13E-01	no
ENSMUSG00000059625	Sohlh1	2.37E+00	4.71E-04	up	3.41E+00	2.34E-04	up
ENSMUSG00000079502	1700101E01Rik	-3.78E+00	2.39E-10	down	-8.15E-01	6.41E-01	no
ENSMUSG00000026960	Arl6ip6	-8.38E-01	3.15E-03	no	-1.10E+00	4.70E-05	down
ENSMUSG00000027077	Smtnl1	-3.22E+00	1.96E-03	no	-3.43E+00	1.53E-04	down
ENSMUSG00000068614	Actc1	5.93E+00	2.35E-03	no	6.54E+00	7.29E-04	up
ENSMUSG00000027227	Sord	-2.68E+00	3.83E-157	down	-2.19E+00	3.54E-121	down
ENSMUSG00000048327	Ckap2l	-2.18E+00	5.00E-11	down	-1.86E+00	6.10E-06	down
ENSMUSG00000027475	Kif3b	-3.95E+00	8.47E-39	down	-3.54E+00	9.69E-35	down
ENSMUSG00000044405	Adig	-1.08E+00	2.13E-04	down	3.08E-01	6.86E-01	no
ENSMUSG00000026969	Fam166a	-2.57E+00	2.34E-94	down	-7.35E-02	8.55E-01	no
ENSMUSG00000026831	1700007K13Rik	-1.07E+00	8.42E-09	down	1.28E+00	4.56E-05	up
ENSMUSG00000027079	Clp1	-2.26E+00	1.00E-07	down	-1.80E+00	4.70E-05	down
ENSMUSG00000079170	Gm13941	-5.02E+00	1.17E-36	down	-3.55E+00	1.06E-20	down

ENSMUSG00000079169	Gm15130	-5.76E+00	2.11E-39	down	-4.70E+00	8.89E-35	down
ENSMUSG00000034850	Tmem127	-3.36E+00	2.07E-11	down	-3.59E+00	1.29E-16	down
ENSMUSG00000037279	Ovol2	-1.15E+00	1.39E-04	down	-1.77E+00	1.52E-13	down
ENSMUSG00000027602	Map1lc3a	1.25E+00	3.38E-46	up	1.20E+00	1.32E-42	up
ENSMUSG00000017009	Sdc4	3.99E+00	2.90E-08	up	2.44E+00	1.72E-05	up
ENSMUSG00000027498	Cstf1	-2.14E+00	9.53E-14	down	-1.63E+00	1.62E-08	down
ENSMUSG00000027517	Ankrd60	-2.86E+00	1.42E-86	down	-1.78E+00	2.83E-26	down
ENSMUSG00000026679	Enkur	-3.05E+00	4.84E-119	down	-2.21E+00	1.99E-48	down
ENSMUSG00000026966	Ssna1	4.34E+00	1.59E-181	up	1.85E+00	6.13E-114	up
ENSMUSG00000036401	Glt6d1	-5.07E+00	1.59E-17	down	-3.85E+00	8.91E-03	no
ENSMUSG00000042369	Rbm45	-2.53E+00	1.43E-01	no	-4.90E+00	2.16E-05	down
ENSMUSG00000046085	4931422A03Rik	-3.99E+00	1.19E-10	down	-3.92E+00	1.75E-12	down
ENSMUSG00000067847	Romo1	3.73E+00	3.98E-155	up	3.87E+00	9.01E-165	up
ENSMUSG00000054455	Vapb	-3.81E+00	1.78E-03	no	-4.91E+00	1.05E-05	down
ENSMUSG00000038987	1700019L03Rik	-3.77E+00	1.43E-49	down	-1.90E+00	3.67E-08	down
ENSMUSG00000026753	Ppp6c	-1.84E+00	8.66E-05	down	-2.29E+00	8.90E-06	down
ENSMUSG00000060703	Cd302	3.53E+00	5.04E-05	up	1.55E+00	2.01E-01	no
ENSMUSG00000070883	Ccdc173	-2.35E+00	5.43E-53	down	-1.62E+00	2.65E-19	down
ENSMUSG00000041255	Tmco5b	-4.33E+00	5.70E-19	down	-3.01E+00	1.04E-03	no
ENSMUSG00000027439	Gzf1	-8.59E-01	4.18E-01	no	-2.81E+00	1.20E-06	down
ENSMUSG00000055177	Cstl1	-2.48E+00	6.33E-23	down	-1.95E+00	6.04E-05	down
ENSMUSG00000067818	Myl9	6.01E+00	9.68E-70	up	2.84E+00	5.90E-11	up
ENSMUSG00000001403	Ube2c	1.65E+00	1.72E-06	up	1.14E+00	5.52E-03	no
ENSMUSG00000027573	Gid8	-2.81E+00	8.97E-11	down	-2.92E+00	5.13E-11	down
ENSMUSG00000026974	Zmynd19	-6.07E-01	2.76E-01	no	-1.55E+00	1.03E-04	down
ENSMUSG00000050592	Fam78a	-3.99E+00	2.03E-25	down	-2.28E+00	6.95E-05	down
ENSMUSG00000002732	Fkbp7	-1.77E+00	1.19E-06	down	-6.27E-01	3.15E-01	no
ENSMUSG00000005802	Slc30a4	-3.30E+00	6.17E-08	down	-2.81E+00	1.78E-02	no
ENSMUSG00000027442	Cst8	-2.33E+00	1.94E-58	down	-9.06E-01	3.72E-04	no
ENSMUSG00000038467	Chmp4b	-5.02E+00	1.61E-42	down	-5.56E+00	8.40E-51	down
ENSMUSG00000048707	Tprn	-2.82E+00	3.22E-67	down	-2.49E+00	6.69E-64	down
ENSMUSG00000048038	4932418E24Rik	-6.73E+00	2.57E-24	down	-4.15E+00	1.17E-03	no
ENSMUSG00000076437	2700094K13Rik	3.62E+00	5.79E-10	up	1.58E+00	2.31E-03	no
ENSMUSG00000027349	Fam98b	-6.09E-01	4.11E-01	no	-1.85E+00	8.04E-05	down
ENSMUSG00000017707	Serinc3	4.81E-02	8.67E-01	no	-1.14E+00	7.04E-06	down
ENSMUSG00000038932	Tcfl5	-2.89E+00	3.16E-50	down	-3.43E+00	1.32E-114	down
ENSMUSG00000038848	Ythdf1	2.38E+00	6.28E-07	up	1.28E+00	9.12E-03	no
ENSMUSG00000026750	Psmb7	-1.91E+00	2.06E-22	down	-2.53E+00	2.90E-52	down
ENSMUSG00000027122	Arl14ep	-2.89E+00	7.28E-06	down	-1.97E+00	5.03E-03	no
ENSMUSG00000003604	Aven	-2.35E+00	9.39E-18	down	-1.69E+00	7.91E-10	down
ENSMUSG00000033368	Trim69	-4.50E+00	2.66E-76	down	-4.24E+00	4.53E-70	down
ENSMUSG00000027203	Dut	2.61E+00	9.61E-15	up	-1.26E-01	6.28E-01	no
ENSMUSG00000037708	BC061194	-1.44E+00	5.66E-17	down	1.35E+00	1.11E-05	up
ENSMUSG00000048550	Thnsl1	-3.79E+00	1.21E-17	down	-2.67E+00	1.53E-06	down
ENSMUSG00000075425	Gm13547	-4.11E+00	1.15E-04	down	-2.51E+00	2.83E-01	no
ENSMUSG00000039715	Wdr34	1.20E+00	8.57E-05	up	-4.67E-01	4.24E-02	no
ENSMUSG00000026851	BC005624	-7.39E-01	2.28E-01	no	-1.82E+00	2.75E-05	down
ENSMUSG00000041777	Cir1	-2.95E+00	1.85E-10	down	-2.55E+00	6.80E-07	down
ENSMUSG00000009549	Srp14	1.50E+00	5.90E-75	up	4.80E-01	1.61E-16	no
ENSMUSG00000074812	Gm355	-5.78E+00	3.61E-10	down	-2.17E+00	2.96E-01	no
ENSMUSG00000047459	Dynlrb1	2.86E+00	6.90E-15	up	2.86E+00	3.88E-10	up
ENSMUSG00000038375	Trp53inp2	-4.39E+00	1.12E-20	down	-1.49E+00	1.44E-01	no
ENSMUSG00000039001	Rps21	1.38E+00	2.82E-19	up	-1.35E-01	4.10E-01	no
ENSMUSG00000027344	Fsip1	-3.49E+00	2.16E-35	down	-1.54E+00	2.04E-04	down
ENSMUSG00000027406	Idh3b	-1.71E+00	1.51E-07	down	-1.42E+00	1.35E-06	down
ENSMUSG00000016921	Srsf6	-1.21E-01	8.11E-01	no	-2.24E+00	3.35E-13	down
ENSMUSG00000035666	Gtf3c4	-2.01E+00	2.08E-02	no	-3.50E+00	3.17E-09	down
ENSMUSG00000026822	Lcn2	3.48E+00	2.86E-02	no	4.36E+00	8.23E-11	up
ENSMUSG00000034075	Zdhhc5	-3.36E+00	1.34E-63	down	-2.43E+00	1.38E-30	down
ENSMUSG00000023224	Serping1	3.67E+00	9.54E-25	up	2.30E+00	7.98E-03	no
ENSMUSG00000027342	Pcna	-2.92E-01	4.89E-01	no	-1.26E+00	1.65E-06	down
ENSMUSG00000027481	Bpifb2	5.05E+00	1.73E-40	up	5.44E+00	3.13E-52	up
ENSMUSG00000018322	Tomm34	8.16E-01	2.18E-01	no	-1.79E+00	1.15E-08	down
ENSMUSG00000015335	Zdhhc12	-9.64E-01	1.77E-02	no	-1.20E+00	3.61E-06	down

ENSMUSG00000048911	Rnf24	-4.42E+00	1.10E-04	down	-3.17E+00	1.10E-01	no
ENSMUSG00000026728	Vim	2.31E+00	1.27E-05	up	6.43E-02	9.54E-01	no
ENSMUSG00000001767	Crnkl1	-2.41E+00	1.00E-07	down	-2.79E+00	2.62E-10	down
ENSMUSG00000042662	Dusp15	-2.31E+00	2.35E-11	down	-2.47E+00	7.23E-22	down
ENSMUSG00000044083	Efcab8	-3.73E+00	2.31E-08	down	-4.30E+00	1.52E-19	down
ENSMUSG00000027482	Bpifa3	-6.10E+00	7.85E-24	down	-4.10E+00	1.53E-05	down
ENSMUSG00000027519	Rab22a	-1.38E+00	2.77E-03	no	-1.90E+00	2.26E-05	down
ENSMUSG00000015085	Entpd2	3.65E+00	6.27E-04	up	1.53E+00	6.20E-01	no
ENSMUSG00000026791	Slc2a8	-9.67E-01	2.88E-02	no	-1.43E+00	1.85E-05	down
ENSMUSG00000069495	Epc2	-3.38E+00	7.95E-08	down	-2.18E+00	2.44E-02	no
ENSMUSG00000018770	Atp5g3	3.61E+00	1.32E-32	up	1.41E+00	3.03E-11	up
ENSMUSG00000027099	Mtx2	6.58E-01	2.74E-01	no	-1.44E+00	3.20E-06	down
ENSMUSG00000034552	Zswim2	-3.78E+00	3.82E-57	down	-2.23E+00	8.64E-13	down
ENSMUSG00000007659	Bcl2l1	-1.80E+00	1.03E-06	down	-2.08E+00	2.75E-12	down
ENSMUSG00000026650	Meig1	1.16E+00	0.00E+00	up	1.17E+00	0.00E+00	up
ENSMUSG00000027001	Dusp19	-1.03E+00	2.52E-01	no	-1.94E+00	6.13E-05	down
ENSMUSG00000034800	Zfp661	-2.83E+00	1.38E-01	no	-4.09E+00	2.97E-05	down
ENSMUSG00000052056	Zfp217	-3.95E+00	6.04E-24	down	-3.22E+00	2.19E-13	down
ENSMUSG00000016257	Slmo2	-2.29E-01	5.95E-01	no	-1.00E+00	7.54E-04	down
ENSMUSG00000026774	4931423N10Rik	-3.16E+00	1.67E-13	down	-3.57E+00	1.62E-22	down
ENSMUSG00000026646	Suv39h2	-2.27E+00	1.32E-05	down	-5.69E-01	6.84E-01	no
ENSMUSG00000027088	Phospho2	-2.81E+00	1.46E-80	down	-2.95E+00	1.49E-114	down
ENSMUSG00000010914	Pdhx	-1.61E+00	5.10E-05	down	-1.69E+00	6.42E-07	down
ENSMUSG00000027329	Spef1	-1.97E+00	7.63E-06	down	-1.43E+00	2.52E-05	down
ENSMUSG00000074749	Plk1s1	-3.29E+00	3.93E-74	down	-3.02E+00	2.10E-71	down
ENSMUSG00000027454	Gins1	3.14E-01	5.24E-01	no	-1.11E+00	1.48E-05	down
ENSMUSG00000027596	a	-2.18E+00	3.71E-01	no	-4.22E+00	2.69E-04	down
ENSMUSG00000026994	Galnt3	-4.26E+00	1.49E-42	down	-4.08E+00	1.21E-39	down
ENSMUSG00000039108	Lsm14b	-2.33E+00	1.19E-03	no	-4.28E+00	3.52E-18	down
ENSMUSG00000016344	Ppdpf	7.38E+00	2.03E-10	up	6.95E+00	1.41E-13	up
ENSMUSG00000027404	Snrpb	4.45E+00	9.42E-72	up	1.52E+00	6.87E-22	up
ENSMUSG00000045624	Esf1	2.41E+00	6.68E-04	up	7.31E-02	9.47E-01	no
ENSMUSG00000027613	Eif6	2.25E+00	1.53E-11	up	2.77E-01	4.43E-01	no
ENSMUSG00000035877	Zhx3	-3.41E+00	4.63E-08	down	-1.82E+00	8.52E-02	no
ENSMUSG00000035399	3230401D17Rik	-2.35E+00	3.56E-12	down	-1.44E+00	8.80E-04	down
ENSMUSG00000027569	Mrgbp	1.59E-01	7.38E-01	no	-1.39E+00	1.18E-10	down
ENSMUSG00000036770	4933433C11Rik	-3.09E+00	7.91E-30	down	-6.78E-01	2.62E-01	no
ENSMUSG00000026922	Agpat2	-4.47E+00	1.41E-23	down	-3.13E+00	5.50E-04	down
ENSMUSG00000026807	Ak8	-2.19E+00	3.65E-57	down	-7.89E-01	5.06E-07	no
ENSMUSG00000069020	Urm1	-3.23E-01	7.94E-01	no	-2.48E+00	3.31E-04	down
ENSMUSG00000035295	Wdr38	-2.80E+00	2.06E-06	down	-1.43E+00	7.04E-02	no
ENSMUSG00000027248	Pdia3	-1.84E+00	1.86E-37	down	-2.65E+00	2.18E-85	down
ENSMUSG00000037514	Pank2	-2.14E+00	1.68E-06	down	-2.10E+00	1.03E-05	down
ENSMUSG00000027465	Tbc1d20	-2.60E+00	1.34E-21	down	-2.29E+00	1.28E-14	down
ENSMUSG00000026940	4921530D09Rik	-3.63E+00	8.45E-59	down	-8.87E-01	5.97E-02	no
ENSMUSG00000049897	Gm711	-2.42E+00	5.46E-26	down	-2.20E+00	8.21E-36	down
ENSMUSG00000033486	Catsper2	-3.10E+00	4.49E-40	down	-2.96E+00	4.15E-43	down
ENSMUSG00000027480	Sun5	-2.59E+00	1.76E-64	down	2.88E-01	4.55E-01	no
ENSMUSG00000018209	Stk4	-2.07E+00	1.48E-02	no	-3.15E+00	1.38E-07	down
ENSMUSG00000027566	Psma7	3.30E+00	1.01E-08	up	1.16E+00	4.24E-02	no
ENSMUSG00000026848	Tor1b	-1.69E+00	1.35E-01	no	-2.75E+00	4.23E-04	down
ENSMUSG00000046854	Pip5kl1	-4.82E+00	1.96E-04	down	-3.48E+00	3.09E-01	no
ENSMUSG00000027134	Lpcat4	-1.84E+00	1.74E-04	down	-1.98E+00	4.99E-05	down
ENSMUSG00000027306	Nusap1	-2.37E+00	3.37E-06	down	-3.33E+00	3.61E-14	down
ENSMUSG00000027364	Usp50	-2.07E+00	3.59E-22	down	6.04E-01	1.57E-01	no
ENSMUSG00000074764	Sel1l2	-3.23E+00	1.28E-29	down	-1.87E+00	9.23E-08	down
ENSMUSG00000042548	Asxl1	-3.13E+00	4.47E-16	down	-2.30E+00	3.28E-08	down
ENSMUSG00000052033	Pfdn4	5.17E-01	5.59E-01	no	-2.19E+00	2.65E-07	down
ENSMUSG00000026734	4921504E06Rik	-3.33E+00	1.74E-25	down	-3.55E-01	7.25E-01	no
ENSMUSG00000036249	Rbm43	-3.83E+00	1.02E-18	down	-1.03E+00	3.01E-01	no
ENSMUSG00000053896	4933409G03Rik	-4.19E+00	1.68E-84	down	-3.56E+00	1.58E-66	down
ENSMUSG00000037197	Rbm17	7.53E-01	1.31E-01	no	-1.13E+00	2.10E-04	down
ENSMUSG00000038844	Kif16b	-3.09E+00	3.32E-05	down	-2.99E+00	7.59E-04	down
ENSMUSG00000027510	Rbm38	3.22E+00	8.04E-04	up	1.49E+00	7.75E-02	no

ENSMUSG00000027170	Eif3m	-9.23E-01	7.53E-02	no	-1.70E+00	6.94E-06	down
ENSMUSG00000032869	Psmf1	-2.67E+00	3.36E-19	down	-1.08E+00	1.90E-02	no
ENSMUSG00000032680	6820408C15Rik	-2.31E+00	1.14E-23	down	-1.09E+00	4.75E-05	down
ENSMUSG00000017858	Ift52	-2.20E+00	3.44E-07	down	-2.71E+00	1.62E-13	down
ENSMUSG00000026879	Gsn	4.11E+00	1.15E-139	up	4.13E-01	3.85E-01	no
ENSMUSG00000027357	Crls1	-1.88E+00	1.37E-17	down	-9.15E-01	2.28E-03	no
ENSMUSG00000033068	Entpd6	-1.45E+00	5.68E-04	down	-2.18E+00	5.99E-15	down
ENSMUSG00000017299	Dnttip1	1.10E+00	2.38E-07	up	1.22E+00	5.59E-16	up
ENSMUSG00000026878	Rab14	1.69E+00	3.97E-04	up	2.48E-01	7.36E-01	no
ENSMUSG00000026655	Fam107b	-2.26E+00	2.70E-11	down	-3.10E+00	8.06E-35	down
ENSMUSG00000027157	4930430A15Rik	-3.24E+00	5.78E-31	down	-2.71E+00	7.29E-24	down
ENSMUSG00000027496	Aurka	-1.41E+00	4.18E-14	down	-1.23E+00	1.15E-16	down
ENSMUSG00000039050	Osbpl2	-8.82E-01	2.75E-01	no	-2.60E+00	3.81E-04	down
ENSMUSG00000026817	Ak1	-3.07E+00	1.12E-54	down	-1.53E+00	4.35E-07	down
ENSMUSG00000026999	Nup35	-2.06E+00	3.28E-04	down	-1.30E+00	8.43E-02	no
ENSMUSG00000040084	Bub1b	-1.94E+00	1.93E-04	down	-2.86E+00	2.02E-14	down
ENSMUSG00000027371	Fahd2a	-1.70E+00	5.57E-04	down	-1.63E+00	5.70E-07	down
ENSMUSG00000027649	Ctnnbl1	-1.86E+00	7.03E-18	down	-1.45E+00	2.60E-11	down
ENSMUSG00000015090	Ptgds	2.19E+00	7.20E-02	no	2.94E+00	9.00E-07	up
ENSMUSG00000027286	Lrrc57	-4.63E+00	1.80E-31	down	-3.91E+00	1.41E-08	down
ENSMUSG00000039804	Ncoa5	-1.05E+00	3.68E-02	no	-2.03E+00	2.34E-08	down
ENSMUSG00000036646	Man1b1	-2.91E+00	1.40E-05	down	-3.94E+00	3.05E-20	down
ENSMUSG00000027091	Zc3h15	-1.24E+00	9.34E-06	down	-1.77E+00	2.52E-11	down
ENSMUSG00000046971	Pla2g4f	4.50E+00	8.04E-04	up	6.17E+00	2.04E-04	up
ENSMUSG00000079056	Kcnip3	3.33E+00	1.17E-05	up	3.58E+00	5.31E-06	up
ENSMUSG00000026926	Pmpca	-2.01E+00	3.74E-08	down	-1.10E+00	1.27E-02	no
ENSMUSG00000039356	Exosc2	1.96E+00	3.69E-04	up	2.21E-01	7.37E-01	no
ENSMUSG00000017740	Slc12a5	-2.91E+00	7.46E-02	no	-4.38E+00	1.73E-04	down
ENSMUSG00000026775	Yme1l1	-3.57E+00	2.59E-16	down	-3.16E+00	8.10E-09	down
ENSMUSG00000026864	Hspa5	-1.12E-01	4.06E-01	no	-1.84E+00	8.87E-58	down
ENSMUSG00000043241	Upf2	-2.06E+00	9.43E-06	down	-2.22E+00	3.00E-06	down
ENSMUSG00000027006	Dnajc10	-2.37E+00	3.06E-09	down	-2.77E+00	2.64E-11	down
ENSMUSG00000050043	Tmx2	-1.70E+00	5.88E-04	down	-6.23E-01	4.08E-01	no
ENSMUSG00000027355	Tmco5	-3.14E+00	7.07E-231	down	-8.18E-01	1.58E-06	no
ENSMUSG00000040035	Disp2	8.97E+00	1.15E-02	no	9.50E+00	3.75E-04	up
ENSMUSG00000027635	Dsn1	-6.26E-01	3.96E-01	no	-2.31E+00	5.52E-09	down
ENSMUSG00000075249	Fsip2	-3.39E+00	1.12E-16	down	-2.07E+00	2.57E-05	down
ENSMUSG00000002103	Acp2	-1.61E+00	1.38E-01	no	-2.59E+00	6.50E-04	down
ENSMUSG00000037773	Pced1a	-1.31E+00	2.01E-01	no	-2.76E+00	2.89E-06	down
ENSMUSG00000027603	Ggt7	-2.65E+00	3.51E-04	down	-2.93E+00	1.30E-05	down
ENSMUSG00000037683	Armc3	-3.45E+00	1.67E-53	down	-2.59E+00	5.30E-32	down
ENSMUSG00000009555	Cdk9	5.49E-01	5.31E-01	no	-2.25E+00	1.50E-06	down
ENSMUSG00000027193	Api5	-5.20E-01	5.34E-01	no	-3.22E+00	1.84E-16	down
ENSMUSG00000033902	Mapkbp1	-2.92E+00	3.28E-04	down	-3.95E+00	2.12E-10	down
ENSMUSG00000027433	Xrn2	-1.65E+00	1.94E-07	down	-1.23E+00	9.89E-03	no
ENSMUSG00000061411	8430427H17Rik	-3.33E+00	1.86E-21	down	-2.93E+00	1.98E-21	down
ENSMUSG00000027522	Stx16	-6.16E-01	5.27E-01	no	-2.84E+00	5.11E-06	down
ENSMUSG00000039678	Tbc1d13	-2.61E+00	1.43E-02	no	-3.20E+00	7.62E-04	down
ENSMUSG00000039501	Znfx1	-3.23E+00	5.41E-06	down	-3.13E+00	7.57E-05	down
ENSMUSG00000027071	P2rx3	-4.13E+00	1.55E-12	down	-1.27E+00	3.12E-01	no
ENSMUSG00000027379	Bub1	-2.46E+00	2.29E-11	down	-2.46E+00	1.85E-08	down
ENSMUSG00000074627	Mroh8	-3.75E+00	1.87E-28	down	-3.34E+00	2.86E-31	down
ENSMUSG00000026975	Wdr85	-2.25E+00	2.91E-02	no	-3.02E+00	5.93E-04	down
ENSMUSG00000015095	Fbxw5	-1.67E+00	2.27E-29	down	-1.59E+00	4.51E-48	down
ENSMUSG00000036352	Ubac1	-7.89E-01	9.07E-03	no	-1.39E+00	4.13E-08	down
ENSMUSG00000070953	Rabepk	-1.64E+00	7.12E-17	down	-1.00E+00	5.12E-07	down
ENSMUSG00000026778	Prkcq	-5.43E+00	9.20E-21	down	-2.39E+00	1.66E-02	no
ENSMUSG00000052922	Bpi	-3.19E+00	1.68E-14	down	-3.03E+00	5.51E-15	down
ENSMUSG00000074582	Arfgef2	-3.50E+00	2.41E-07	down	-3.33E+00	2.54E-05	down
ENSMUSG00000026914	Psmd14	6.60E-01	2.73E-01	no	-1.73E+00	9.09E-07	down
ENSMUSG00000027378	Nphp1	-2.75E+00	2.18E-163	down	-2.28E+00	6.22E-138	down
ENSMUSG00000039033	Tasp1	-2.69E+00	3.67E-08	down	-1.07E+00	1.95E-01	no
ENSMUSG00000014959	Gorasp2	-1.28E+00	2.67E-10	down	-1.13E+00	4.36E-09	down
ENSMUSG00000027206	Cops2	-3.51E+00	1.21E-28	down	-3.63E+00	5.36E-27	down

ENSMUSG00000038085	Cnbd2	-2.65E+00	6.29E-15	down	-1.45E+00	5.92E-03	no
ENSMUSG00000005803	Sqrdl	-3.26E+00	5.17E-65	down	-4.07E-01	3.56E-01	no
ENSMUSG00000055371	Stam2	-1.96E+00	1.36E-04	down	-1.82E+00	1.14E-03	no
ENSMUSG00000027425	Csrp2bp	-1.48E+00	3.15E-01	no	-2.97E+00	9.98E-04	down
ENSMUSG00000026739	Bmi1	-3.52E+00	3.41E-22	down	-2.61E+00	3.34E-07	down
ENSMUSG00000043535	Setx	-2.25E+00	4.20E-19	down	-3.69E+00	4.42E-92	down
ENSMUSG00000025781	Atp5c1	-3.69E-01	7.79E-02	no	-1.01E+00	1.65E-09	down
ENSMUSG00000033256	Shf	-2.47E+00	1.02E-02	no	-2.70E+00	1.00E-03	down
ENSMUSG00000075302	Erich2	-3.24E+00	9.76E-51	down	-2.39E+00	1.75E-24	down
ENSMUSG00000068735	Trp53i11	-2.19E+00	5.56E-03	no	-3.24E+00	3.14E-08	down
ENSMUSG00000000827	Tpd52l2	2.96E+00	1.27E-04	up	1.24E+00	1.40E-01	no
ENSMUSG00000026672	Optn	-2.83E+00	1.02E-26	down	-1.99E+00	5.46E-14	down
ENSMUSG00000026766	Mmadhc	-1.77E+00	2.93E-27	down	-5.86E-01	2.72E-03	no
ENSMUSG00000027180	Fbxo3	3.71E-01	6.86E-01	no	-2.02E+00	1.09E-06	down
ENSMUSG00000027489	Necab3	-2.32E+00	1.29E-05	down	5.04E-01	7.05E-01	no
ENSMUSG00000005883	Spo11	-1.52E+00	9.33E-04	down	-2.44E+00	6.69E-18	down
ENSMUSG00000026925	Inpp5e	-3.52E+00	3.96E-13	down	-3.31E+00	4.11E-10	down
ENSMUSG00000026885	Ttll11	-1.66E+00	3.19E-04	down	-8.64E-01	1.18E-01	no
ENSMUSG00000063145	Bbs5	-3.22E+00	3.09E-29	down	-1.94E+00	6.06E-06	down
ENSMUSG00000068882	Ssb	1.62E+00	7.05E-07	up	-1.26E+00	1.87E-06	down
ENSMUSG00000051154	Commd3	-1.35E+00	3.13E-05	down	-7.93E-01	6.30E-02	no
ENSMUSG00000008200	Fnbp4	-1.89E+00	8.85E-03	no	-2.87E+00	1.17E-06	down
ENSMUSG00000027255	Arfgap2	-2.49E+00	4.13E-03	no	-2.87E+00	3.65E-06	down
ENSMUSG00000027363	Usp8	-3.85E+00	1.42E-64	down	-3.40E+00	4.38E-50	down
ENSMUSG00000061136	Prpf40a	-1.96E+00	3.39E-06	down	-2.25E+00	5.78E-07	down
ENSMUSG00000027177	Hipk3	-3.88E+00	1.91E-20	down	-3.45E+00	9.73E-12	down
ENSMUSG00000027422	Rrbp1	-1.24E+00	9.66E-04	down	-1.93E+00	1.22E-09	down
ENSMUSG00000002455	Prpf6	-1.98E+00	3.45E-06	down	-1.73E+00	4.85E-05	down
ENSMUSG00000015087	Rabl6	-2.02E+00	3.26E-04	down	-2.13E+00	2.11E-05	down
ENSMUSG00000026842	Abl1	-3.71E+00	1.54E-05	down	-3.59E+00	2.63E-04	down
ENSMUSG00000027244	Atg13	-2.54E+00	2.77E-06	down	-2.80E+00	1.94E-06	down
ENSMUSG00000038369	Ncoa6	-2.26E+00	3.15E-03	no	-2.90E+00	4.08E-06	down
ENSMUSG00000026856	Dolpp1	-7.67E-01	4.08E-01	no	-2.14E+00	4.11E-05	down
ENSMUSG00000039515	Ppp2r4	-1.40E-01	7.63E-01	no	-1.99E+00	5.82E-21	down
ENSMUSG00000027165	B230118H07Rik	-2.02E+00	7.64E-13	down	-2.09E+00	6.26E-22	down
ENSMUSG00000032046	Abhd12	-9.40E-01	1.36E-02	no	-1.66E+00	3.73E-07	down
ENSMUSG00000000823	Znf512b	-2.86E+00	4.36E-09	down	-3.25E+00	2.82E-16	down
ENSMUSG00000040174	Alkbh3	-1.83E+00	2.82E-07	down	-1.49E+00	7.22E-05	down
ENSMUSG00000060227	Casc4	-2.03E+00	2.32E-04	down	-2.17E+00	1.03E-03	no
ENSMUSG00000074771	Ankef1	-4.77E+00	8.01E-53	down	-2.58E+00	4.00E-07	down
ENSMUSG00000013523	Bcas1	-6.09E+00	6.21E-08	down	-6.40E+00	2.86E-07	down
ENSMUSG00000026781	Acbd5	-2.54E+00	1.97E-04	down	-1.86E+00	5.80E-02	no
ENSMUSG00000063972	Nr6a1	-3.65E+00	1.58E-20	down	-1.96E+00	4.95E-04	down
ENSMUSG00000051329	Nup160	-3.09E+00	1.40E-04	down	-1.73E+00	1.97E-01	no
ENSMUSG00000039686	Zer1	-3.12E+00	1.29E-04	down	-2.97E+00	1.08E-04	down
ENSMUSG00000027030	Stk39	-3.03E+00	1.02E-43	down	-2.95E+00	3.26E-54	down
ENSMUSG00000027331	Knstrn	-4.24E+00	6.99E-17	down	-3.12E+00	9.48E-07	down
ENSMUSG00000027209	Fam227b	-4.37E+00	6.50E-43	down	-4.23E+00	6.07E-44	down
ENSMUSG00000068115	Ninl	-1.77E+00	6.37E-02	no	-2.71E+00	2.73E-07	down
ENSMUSG00000027593	Raly	-1.19E+00	8.71E-02	no	-2.61E+00	4.95E-06	down
ENSMUSG00000038180	Spag4	-2.00E+00	1.58E-16	down	3.58E-01	5.40E-01	no
ENSMUSG00000034154	Ino80	-1.67E+00	2.21E-01	no	-3.51E+00	1.36E-04	down
ENSMUSG00000026927	Sdccag3	-3.55E+00	3.64E-57	down	-2.45E+00	3.66E-18	down
ENSMUSG00000036890	Gtdc1	-2.36E+00	3.29E-02	no	-3.65E+00	3.30E-04	down
ENSMUSG00000026970	Rbms1	-2.93E+00	4.28E-13	down	-7.68E-01	5.04E-01	no
ENSMUSG00000027067	Ssrp1	-2.15E+00	1.32E-09	down	-2.40E+00	1.28E-14	down
ENSMUSG00000005881	Ergic3	-5.05E-01	7.62E-03	no	-1.03E+00	4.70E-10	down
ENSMUSG00000017754	Pltp	5.12E+00	2.60E-18	up	3.19E+00	2.72E-02	no
ENSMUSG00000003660	Snrnp200	-1.81E+00	2.19E-04	down	-2.83E+00	6.96E-14	down
ENSMUSG00000038324	Trpc4ap	-1.36E+00	3.50E-03	no	-2.45E+00	3.33E-10	down
ENSMUSG00000039536	Stau1	-2.58E-02	9.80E-01	no	-2.54E+00	1.21E-06	down
ENSMUSG00000027411	Vps16	6.61E-01	4.66E-01	no	-1.81E+00	6.99E-04	down
ENSMUSG00000038914	Dido1	-2.29E+00	7.31E-04	down	-1.90E+00	1.83E-02	no
ENSMUSG00000026827	Gpd2	-4.24E+00	2.73E-66	down	-1.74E+00	6.34E-05	down

ENSMUSG00000034848	Ttc21b	-2.82E+00	1.51E-06	down	-3.23E+00	1.99E-13	down
ENSMUSG00000025314	Ptprj	-3.92E+00	4.28E-04	down	-1.01E+00	7.64E-01	no
ENSMUSG00000061186	Sfmbt2	-3.51E+00	1.22E-04	down	-3.71E+00	6.70E-07	down
ENSMUSG00000060445	Sycp2	-1.69E+00	8.38E-10	down	-3.75E+00	4.64E-87	down
ENSMUSG00000044320	1700001O22Rik	-3.60E+00	4.98E-30	down	-1.96E+00	9.06E-07	down
ENSMUSG00000001855	Nup214	-2.47E+00	4.82E-06	down	-2.07E+00	1.10E-03	no
ENSMUSG00000038831	Ralgps1	-2.04E+00	1.30E-03	no	-2.55E+00	2.27E-06	down
ENSMUSG00000090100	Ttbk2	-2.59E+00	3.87E-04	down	-2.74E+00	1.10E-04	down
ENSMUSG00000026819	Slc25a25	-2.63E+00	7.88E-08	down	-8.60E-01	4.37E-01	no
ENSMUSG00000027634	Ndrg3	-2.91E+00	2.09E-12	down	-3.11E+00	1.98E-14	down
ENSMUSG00000026853	Crat	-2.58E+00	1.44E-58	down	-2.27E+00	2.84E-65	down
ENSMUSG00000027002	Nckap1	-2.64E+00	2.45E-11	down	-3.02E+00	3.31E-13	down
ENSMUSG00000016356	Col20a1	-3.93E+00	9.36E-09	down	-3.95E+00	7.82E-07	down
ENSMUSG00000039254	Pomt1	-2.01E+00	1.61E-09	down	-2.25E+00	4.80E-20	down
ENSMUSG00000056486	Chn1	-2.02E+00	6.34E-10	down	-3.54E-01	6.17E-01	no
ENSMUSG00000027469	Tpx2	-1.20E+00	7.67E-02	no	-2.81E+00	2.23E-08	down
ENSMUSG00000039660	D2Wsu81e	-1.74E+00	8.70E-16	down	-1.24E+00	1.36E-11	down
ENSMUSG00000026918	Brd3	-2.19E+00	2.24E-11	down	-1.46E+00	7.99E-06	down
ENSMUSG00000074673	Ttll9	-3.07E+00	8.31E-45	down	-4.24E-01	3.31E-01	no
ENSMUSG00000027642	Rpn2	-8.91E-01	8.92E-07	no	-1.56E+00	9.09E-23	down
ENSMUSG00000026933	Camsap1	-3.41E+00	3.28E-28	down	-2.52E+00	8.28E-14	down
ENSMUSG00000038696	Mapkap1	-1.44E+00	1.24E-04	down	-2.35E+00	6.72E-14	down
ENSMUSG00000027184	Caprin1	-2.94E+00	3.51E-16	down	-2.79E+00	1.45E-13	down
ENSMUSG00000026821	Ralgds	-3.12E+00	4.34E-26	down	-2.71E+00	1.13E-20	down
ENSMUSG00000039021	Ttc16	-3.77E+00	9.85E-15	down	-3.04E+00	3.85E-10	down
ENSMUSG00000027605	Acss2	-1.71E+00	1.83E-02	no	-2.52E+00	1.34E-04	down
ENSMUSG00000026785	Pkn3	6.44E+00	3.40E-06	up	5.31E+00	3.15E-07	up
ENSMUSG00000026854	Usp20	-3.29E+00	3.62E-11	down	-2.87E+00	5.02E-09	down
ENSMUSG00000052533	Nup188	-3.62E+00	9.64E-23	down	-3.31E+00	6.35E-22	down
ENSMUSG00000009207	Lnp	-2.73E+00	4.09E-05	down	-1.92E+00	3.44E-02	no
ENSMUSG00000061689	Dlgap4	-2.40E+00	3.41E-04	down	-1.13E+00	3.44E-01	no
ENSMUSG00000079324	4932414N04Rik	-4.60E+00	2.12E-26	down	-4.01E+00	3.68E-26	down
ENSMUSG00000026924	Sec16a	-3.30E+00	1.99E-06	down	-3.06E+00	1.80E-06	down
ENSMUSG00000026754	Golga1	-2.17E+00	6.86E-03	no	-3.03E+00	3.19E-08	down
ENSMUSG00000033705	Stard9	-2.84E+00	7.26E-05	down	3.02E-02	9.88E-01	no
ENSMUSG00000027130	Slc12a6	-9.43E-01	5.45E-01	no	-3.21E+00	4.12E-04	down
ENSMUSG00000059173	Pde1a	-3.80E+00	1.22E-30	down	-1.46E+00	1.65E-02	no
ENSMUSG00000060131	Atp8b4	5.09E+00	1.57E-02	no	5.17E+00	7.29E-04	up
ENSMUSG00000037143	4930529M08Rik	-4.25E+00	9.88E-08	down	-3.23E+00	5.80E-04	down
ENSMUSG00000036202	Rif1	-1.02E+00	1.31E-01	no	-2.52E+00	1.94E-09	down
ENSMUSG00000040506	Ambra1	-2.40E+00	8.22E-04	down	-3.69E+00	1.32E-10	down
ENSMUSG00000027201	Myef2	-2.72E-01	8.28E-01	no	-3.06E+00	2.37E-07	down
ENSMUSG00000039844	Rapgef1	-2.97E+00	4.86E-07	down	-3.09E+00	2.47E-09	down
ENSMUSG00000040812	Agbl2	-2.70E+00	1.56E-07	down	-3.48E+00	9.85E-22	down
ENSMUSG00000005882	Uqcc	-2.48E+00	3.76E-05	down	-8.50E-01	3.73E-01	no
ENSMUSG00000002718	Cse1l	-2.34E+00	6.79E-07	down	-2.01E+00	5.27E-04	down
ENSMUSG00000036833	Pnpla7	-3.08E+00	5.34E-07	down	-3.08E+00	7.42E-10	down
ENSMUSG00000039205	Ciz1	-3.07E+00	6.13E-07	down	-2.88E+00	9.38E-08	down
ENSMUSG00000043909	Trp53bp1	-2.77E+00	8.66E-04	down	-2.36E+00	1.95E-02	no
ENSMUSG00000027012	Dync1i2	-1.11E+00	1.71E-03	no	-1.06E+00	1.58E-04	down
ENSMUSG00000026773	Pfkfb3	-2.48E+00	1.32E-04	down	2.36E-01	8.99E-01	no
ENSMUSG00000002546	Golga2	-1.64E+00	3.18E-03	no	-2.27E+00	5.90E-07	down
ENSMUSG00000026825	Dnm1	-3.76E+00	4.34E-03	no	-4.48E+00	1.33E-04	down
ENSMUSG00000027546	Atp9a	-2.65E+00	1.49E-06	down	-2.22E+00	9.04E-05	down
ENSMUSG00000027104	Atf2	-2.25E+00	5.85E-06	down	-2.64E+00	6.33E-07	down
ENSMUSG00000027185	Nat10	-2.17E+00	1.42E-02	no	-3.41E+00	7.60E-08	down
ENSMUSG00000027620	Rbm39	1.99E+00	9.17E-12	up	5.11E-02	9.15E-01	no
ENSMUSG00000079110	Capn3	5.20E+00	1.91E-05	up	5.66E+00	7.13E-07	up
ENSMUSG00000058318	Phf21a	-2.51E+00	5.68E-03	no	-4.17E+00	2.09E-10	down
ENSMUSG00000026790	Odf2	-3.22E+00	9.90E-236	down	-2.15E+00	6.52E-83	down
ENSMUSG00000038685	Rtel1	-2.07E+00	1.06E-02	no	-3.32E+00	7.69E-08	down
ENSMUSG00000057110	Cep110	-7.73E-01	2.77E-01	no	-2.18E+00	2.54E-05	down
ENSMUSG00000057738	Sptan1	-2.78E+00	4.23E-09	down	-3.52E+00	1.69E-14	down
ENSMUSG00000034101	Ctnnd1	-2.34E+00	2.25E-02	no	-3.47E+00	2.90E-06	down

ENSMUSG00000027564	Cypt12	-1.32E+00	7.27E-141	down	-4.77E-01	1.52E-05	no
ENSMUSG00000057036	Gm7536	-1.02E+00	9.60E-24	down	-2.18E+00	6.85E-130	down
ENSMUSG00000044330	Gm9790	2.57E+00	1.95E-97	up	1.91E+00	4.71E-89	up
ENSMUSG00000074479	Gm10704	-1.89E+00	5.76E-35	down	-2.47E+00	2.26E-85	down
ENSMUSG00000096177	Gm5070	1.06E+00	1.73E-07	up	7.22E-01	7.33E-04	no
ENSMUSG00000060438	Rps10-ps1	2.65E+00	1.48E-22	up	1.27E+00	8.04E-09	up
ENSMUSG00000068855	Hist2h2ac	5.33E+00	4.40E-30	up	3.86E+00	2.35E-21	up
ENSMUSG00000068854	Hist2h2be	-3.74E+00	9.85E-04	down	-5.01E+00	4.78E-16	down
ENSMUSG00000043468	Adam30	-2.38E+00	1.40E-20	down	-1.93E+00	7.40E-13	down
ENSMUSG00000092072	Gm4540	2.64E+00	1.12E-14	up	-2.41E-01	5.01E-01	no
ENSMUSG00000055891	Ubl4b	-4.29E+00	2.19E-94	down	-2.52E+00	5.21E-15	down
ENSMUSG00000047674	Pdha2	-7.52E-01	8.64E-06	no	-1.01E+00	5.99E-15	down
ENSMUSG00000070343	Gm10288	-1.28E+00	2.01E-33	down	-2.41E+00	2.28E-184	down
ENSMUSG00000047676	Rpsa-ps10	1.65E-01	5.25E-01	no	-1.28E+00	9.13E-14	down
ENSMUSG00000049230	Gm9833	-1.12E+00	2.15E-02	no	-2.09E+00	2.32E-09	down
ENSMUSG00000048109	Rbm15	-2.14E+00	1.69E-03	no	-2.56E+00	1.73E-05	down
ENSMUSG00000054091	1810037I17Rik	1.17E+00	6.10E-04	up	-6.13E-01	1.65E-02	no
ENSMUSG00000050549	5730508B09Rik	-6.40E-01	2.83E-01	no	-2.26E+00	2.84E-12	down
ENSMUSG00000037737	Actrt3	-3.69E+00	2.94E-16	down	-2.75E+00	5.41E-03	no
ENSMUSG00000027811	4930579G24Rik	1.18E+00	1.38E-02	no	-1.66E+00	3.67E-15	down
ENSMUSG00000027927	Lelp1	-1.58E+00	1.18E-42	down	1.87E-01	5.41E-01	no
ENSMUSG00000074435	Smcp	-1.48E+00	0.00E+00	down	-1.83E-01	2.61E-02	no
ENSMUSG00000053192	Mllt11	-3.61E+00	3.69E-49	down	-3.12E+00	6.31E-42	down
ENSMUSG00000078604	Gm10961	-3.57E+00	5.75E-16	down	-1.79E+00	5.18E-04	down
ENSMUSG00000078783	Gm9733	4.73E+00	5.48E-02	no	6.19E+00	5.37E-04	up
ENSMUSG00000038997	Asb17	-2.80E+00	1.20E-32	down	-8.25E-01	8.33E-02	no
ENSMUSG00000001025	S100a6	6.99E+00	3.78E-69	up	4.88E+00	9.40E-07	up
ENSMUSG00000091175	9130204L05Rik	-4.16E+00	1.75E-05	down	-1.05E+00	6.33E-01	no
ENSMUSG00000027907	S100a11	4.45E+00	8.25E-17	up	1.99E+00	6.08E-02	no
ENSMUSG00000052468	Pmp2	-1.11E+00	3.78E-01	no	-2.47E+00	8.36E-05	down
ENSMUSG00000027528	Fabp9	1.70E+00	0.00E+00	up	1.45E+00	0.00E+00	up
ENSMUSG00000087260	Lamtor5	2.66E+00	1.89E-32	up	7.07E-01	4.27E-06	no
ENSMUSG00000078590	Gm10959	4.66E+00	1.11E-04	up	#VALUE!	NA	no
ENSMUSG00000027667	Zfp639	-1.87E+00	1.88E-06	down	-2.42E+00	2.44E-16	down
ENSMUSG00000027806	Tsc22d2	-1.77E+00	2.97E-02	no	-3.08E+00	1.13E-04	down
ENSMUSG00000036432	Siah2	-1.68E+00	8.77E-12	down	-5.63E-01	1.62E-01	no
ENSMUSG00000069118	1700008P02Rik	-6.25E+00	6.03E-21	down	-4.64E+00	2.17E-05	down
ENSMUSG00000048039	Isg20l2	-3.59E+00	2.96E-19	down	-2.64E+00	4.55E-06	down
ENSMUSG00000078667	1700094D03Rik	-1.71E+00	1.27E-09	down	-3.41E-01	5.91E-01	no
ENSMUSG00000090733	Rps27	1.45E+00	9.67E-05	up	2.09E-01	7.19E-01	no
ENSMUSG00000051788	4930564D02Rik	-3.48E+00	1.16E-02	no	-3.87E+00	9.27E-05	down
ENSMUSG00000028195	Cyr61	4.45E+00	2.52E-30	up	3.62E+00	3.59E-04	up
ENSMUSG00000049940	Pgrmc2	-3.13E+00	1.17E-13	down	-3.05E+00	5.87E-15	down
ENSMUSG00000090527	Gm5538	-3.83E+00	1.52E-01	no	-5.89E+00	1.06E-05	down
ENSMUSG00000091685	Gm17359	-2.60E+00	9.36E-56	down	-1.32E+00	5.55E-11	down
ENSMUSG00000028066	Pmf1	2.73E+00	3.88E-14	up	6.92E-01	1.04E-02	no
ENSMUSG00000041355	Ssr2	1.51E+00	1.64E-14	up	-5.78E-01	4.89E-04	no
ENSMUSG00000068860	Gm128	-1.39E+00	1.77E-50	down	-9.09E-01	1.10E-20	no
ENSMUSG00000068732	Tmem167b	-1.37E+00	1.60E-01	no	-2.63E+00	2.41E-04	down
ENSMUSG00000028128	F3	5.17E+00	9.18E-25	up	1.81E+00	5.76E-02	no
ENSMUSG00000069041	Slc25a31	1.74E+00	2.17E-12	up	-7.33E-01	2.00E-04	no
ENSMUSG00000028081	Rps3a1	-1.83E-01	7.66E-01	no	-1.58E+00	6.68E-04	down
ENSMUSG00000015854	Cd5l	-2.97E+00	8.35E-05	down	1.02E-01	9.68E-01	no
ENSMUSG00000028070	Apoa1bp	1.10E+00	1.29E-08	up	3.35E-01	6.81E-02	no
ENSMUSG00000028044	Cks1b	3.17E+00	1.12E-06	up	8.27E-01	1.21E-01	no
ENSMUSG00000044080	S100a1	4.66E+00	2.40E-11	up	2.83E+00	7.11E-03	no
ENSMUSG00000069114	Zbtb10	-2.99E+00	2.01E-03	no	-3.14E+00	5.35E-04	down
ENSMUSG00000028141	Oaz3	-3.33E-01	1.33E-18	no	1.12E+00	1.83E-69	up
ENSMUSG00000038374	Rbm8a	2.76E+00	8.43E-10	up	4.52E-01	2.62E-01	no
ENSMUSG00000074344	Adora3	-3.21E+00	3.15E-83	down	-1.24E+00	8.05E-05	down
ENSMUSG00000053931	Cnn3	4.00E+00	1.69E-05	up	2.28E+00	8.11E-02	no
ENSMUSG00000028029	Aimp1	-1.57E+00	9.00E-15	down	-2.10E+00	7.62E-42	down
ENSMUSG00000028271	Gtf2b	-1.34E+00	4.29E-12	down	-1.86E+00	1.75E-38	down
ENSMUSG00000090202	4930503B20Rik	-2.58E+00	3.02E-08	down	-1.18E+00	1.55E-01	no

ENSMUSG00000027562	Car2	-5.39E+00	8.34E-24	down	-4.76E+00	6.30E-07	down
ENSMUSG00000004895	Prcc	-2.91E+00	1.89E-05	down	-4.00E+00	5.54E-12	down
ENSMUSG00000027944	Hax1	-2.26E+00	8.98E-08	down	-9.62E-01	4.18E-02	no
ENSMUSG00000038619	Ensa	2.06E-01	6.63E-01	no	-1.56E+00	9.02E-09	down
ENSMUSG00000038612	Mcl1	-5.09E-01	1.91E-01	no	-2.23E+00	2.17E-11	down
ENSMUSG00000027869	Hsd3b6	4.41E+00	1.09E-02	no	4.29E+00	7.03E-05	up
ENSMUSG00000027999	Pla2g12a	1.21E+00	3.70E-05	up	5.59E-01	3.87E-02	no
ENSMUSG00000046818	Ddit4l	-2.10E+00	9.70E-03	no	-2.66E+00	6.25E-07	down
ENSMUSG00000012042	4930579F01Rik	-2.72E+00	3.60E-40	down	5.15E-01	2.62E-01	no
ENSMUSG00000028156	Eif4e	-1.41E+00	3.90E-08	down	-1.62E+00	1.20E-10	down
ENSMUSG00000028182	Lrriq3	-3.81E+00	2.48E-53	down	-3.54E+00	1.27E-51	down
ENSMUSG00000027706	Sec62	2.39E+00	1.58E-11	up	4.50E-01	2.35E-01	no
ENSMUSG00000036615	Rfxap	2.89E+00	3.91E-07	up	-2.21E-01	6.51E-01	no
ENSMUSG00000001020	S100a4	6.60E+00	1.96E-20	up	5.28E+00	5.91E-03	no
ENSMUSG00000041959	S100a10	4.23E+00	1.55E-18	up	3.33E+00	2.35E-04	up
ENSMUSG00000044468	Fam46c	-4.04E+00	1.51E-63	down	-2.41E+00	4.28E-12	down
ENSMUSG00000000001	Gnai3	-5.39E-01	4.27E-01	no	-2.18E+00	2.06E-06	down
ENSMUSG00000027886	1700013F07Rik	-7.64E-01	4.55E-12	no	1.75E+00	2.88E-20	up
ENSMUSG00000027962	Vcam1	3.53E+00	1.69E-06	up	3.25E+00	1.87E-03	no
ENSMUSG00000050931	Sgms2	-3.15E+00	4.34E-15	down	-1.55E-01	9.13E-01	no
ENSMUSG00000055301	Adh7	-5.02E+00	8.22E-04	down	-4.78E-01	8.92E-01	no
ENSMUSG00000037062	Sh3glb1	-2.25E+00	8.87E-98	down	-2.00E+00	1.27E-78	down
ENSMUSG00000028187	Rpf1	-1.38E+00	8.00E-05	down	-1.44E+00	1.94E-05	down
ENSMUSG00000027793	Ccna1	-2.39E+00	2.54E-45	down	-2.54E+00	8.51E-68	down
ENSMUSG00000001018	Snapin	-5.65E-01	2.53E-01	no	-1.44E+00	8.55E-06	down
ENSMUSG00000046317	BC107364	-7.02E-01	5.88E-01	no	-2.56E+00	2.10E-04	down
ENSMUSG00000002228	Ppm1j	-2.90E+00	2.75E-36	down	-1.34E-01	8.45E-01	no
ENSMUSG00000037814	Larp1b	-3.15E+00	4.99E-143	down	-2.90E+00	1.34E-147	down
ENSMUSG00000037152	Ndufc1	4.21E+00	1.27E-256	up	3.24E+00	2.37E-263	up
ENSMUSG00000095832	Supt20	-1.62E+00	5.16E-20	down	-1.76E+00	1.63E-32	down
ENSMUSG00000075700	2810407C02Rik	-2.60E+00	3.28E-09	down	-2.86E+00	1.04E-12	down
ENSMUSG00000010538	Tsacc	1.01E+00	3.45E-33	up	1.09E+00	5.04E-44	up
ENSMUSG00000042390	Gatad2b	-2.73E+00	7.85E-11	down	-7.95E-01	3.72E-01	no
ENSMUSG00000074212	Dnajb14	-2.18E+00	2.63E-08	down	-1.84E+00	9.91E-06	down
ENSMUSG00000005813	Metap1	-1.62E+00	1.64E-04	down	-2.14E+00	7.80E-10	down
ENSMUSG00000027702	Lrrc34	-3.34E+00	8.22E-176	down	-2.21E+00	3.11E-57	down
ENSMUSG00000027805	Pfn2	-3.51E+00	1.51E-27	down	-3.74E+00	7.28E-40	down
ENSMUSG00000008604	Ubqln4	-2.81E+00	5.18E-07	down	-3.01E+00	1.83E-09	down
ENSMUSG00000028106	Rprd2	-2.90E+00	5.86E-03	no	-3.07E+00	4.42E-04	down
ENSMUSG00000062127	Cttnbp2nl	-3.45E+00	4.05E-06	down	-2.47E+00	1.46E-02	no
ENSMUSG00000010136	Pifo	-1.14E+00	1.73E-16	down	-1.88E-02	9.36E-01	no
ENSMUSG00000027973	1700006A11Rik	3.35E-02	9.36E-01	no	-1.93E+00	4.71E-40	down
ENSMUSG00000037531	Mrpl47	-2.43E+00	3.17E-11	down	-2.68E+00	3.07E-15	down
ENSMUSG00000028145	Them4	-7.26E-01	4.46E-01	no	-2.15E+00	7.95E-06	down
ENSMUSG00000054312	Mrps21	1.49E+00	9.57E-05	up	3.57E-01	3.69E-01	no
ENSMUSG00000070372	Capza1	-2.77E+00	3.61E-13	down	-3.50E+00	1.11E-28	down
ENSMUSG00000027968	Larp7	-1.36E+00	1.93E-45	down	-1.39E+00	3.25E-56	down
ENSMUSG00000027712	Anxa5	4.62E+00	1.35E-10	up	3.11E+00	1.82E-02	no
ENSMUSG00000047557	Lxn	-1.98E+00	4.77E-03	no	-3.08E+00	1.96E-04	down
ENSMUSG00000034317	Trim59	-2.13E+00	3.29E-09	down	-1.29E+00	8.67E-03	no
ENSMUSG00000042572	Ube2q1	-2.93E+00	1.15E-11	down	-2.98E+00	3.46E-14	down
ENSMUSG00000028137	Celf3	-2.88E+00	8.06E-13	down	2.23E-01	8.56E-01	no
ENSMUSG00000028134	Ptbp2	-1.52E+00	1.82E-04	down	-3.51E+00	1.22E-40	down
ENSMUSG00000027803	Wwtr1	-2.62E+00	2.70E-04	down	-2.97E-01	9.08E-01	no
ENSMUSG00000070471	Fam194a	-4.37E+00	1.32E-36	down	-1.82E+00	1.94E-03	no
ENSMUSG00000033882	Rbm46	4.53E-01	4.45E-01	no	-1.90E+00	8.35E-10	down
ENSMUSG00000028060	2810403A07Rik	-2.03E+00	2.05E-03	no	-2.90E+00	6.25E-05	down
ENSMUSG00000001016	Ilf2	-3.99E-01	2.53E-01	no	-1.87E+00	1.75E-17	down
ENSMUSG00000074655	Gm1527	-3.80E+00	1.95E-07	down	-1.49E+00	2.58E-01	no
ENSMUSG00000051036	Ttc24	-3.85E+00	1.33E-14	down	-3.18E+00	5.73E-04	down
ENSMUSG00000028062	Lamtor2	2.21E+00	3.32E-09	up	9.51E-01	3.32E-03	no
ENSMUSG00000042784	Muc1	-3.68E+00	2.65E-08	down	-3.68E+00	7.52E-10	down
ENSMUSG00000042747	Krtcap2	2.74E+00	1.09E-16	up	1.35E+00	1.44E-06	up
ENSMUSG00000027852	Nras	-4.16E-01	2.68E-01	no	-1.53E+00	8.70E-11	down

ENSMUSG00000027552	E2f5	-1.36E+00	8.12E-04	down	-6.89E-01	4.95E-01	no
ENSMUSG00000027630	Tbl1xr1	-2.79E+00	3.67E-04	down	-2.86E+00	8.01E-05	down
ENSMUSG00000047824	Pygo2	-1.25E+00	1.28E-01	no	-2.99E+00	5.17E-08	down
ENSMUSG00000027937	Jtb	3.48E+00	1.93E-06	up	8.95E-01	1.29E-01	no
ENSMUSG00000038642	Ctss	4.19E+00	2.54E-15	up	5.56E+00	3.62E-04	up
ENSMUSG00000002233	Rhoc	4.99E+00	3.14E-10	up	3.61E+00	8.39E-03	no
ENSMUSG00000027959	Sass6	-3.49E+00	1.69E-14	down	-3.01E+00	2.68E-11	down
ENSMUSG00000037072	43723	-8.63E-01	2.31E-06	no	-1.29E+00	4.89E-14	down
ENSMUSG00000068923	Syt11	-1.75E+00	2.57E-04	down	-2.22E+00	6.64E-09	down
ENSMUSG00000027905	Ddx20	-3.55E+00	5.59E-66	down	-1.95E+00	1.37E-11	down
ENSMUSG00000062006	Rpl34	2.16E+00	8.22E-04	up	7.58E-01	2.39E-01	no
ENSMUSG00000037797	Adh4	-2.05E+00	6.81E-04	down	-2.23E+00	3.28E-07	down
ENSMUSG00000027823	Gmps	-2.71E+00	2.09E-18	down	-2.13E+00	7.25E-10	down
ENSMUSG00000062200	Vmn2r7	-4.42E+00	7.73E-05	down	-2.55E+00	3.51E-01	no
ENSMUSG00000005779	Psmb4	1.85E+00	6.52E-41	up	4.17E-01	2.83E-04	no
ENSMUSG00000040600	Eps8l3	-3.17E+00	1.35E-08	down	-2.33E+00	6.93E-06	down
ENSMUSG00000027880	Slc25a54	-3.06E+00	8.83E-10	down	-3.08E+00	3.67E-13	down
ENSMUSG00000028035	Dnajb4	-2.33E+00	1.78E-22	down	-1.82E+00	2.18E-08	down
ENSMUSG00000027742	Cog6	-2.45E+00	1.84E-33	down	-2.87E+00	1.39E-59	down
ENSMUSG00000028140	Mrpl9	2.60E+00	5.92E-09	up	-2.41E-01	7.02E-01	no
ENSMUSG00000068823	Csde1	-4.01E+00	1.76E-104	down	-3.46E+00	4.28E-65	down
ENSMUSG00000058135	Gstm1	6.03E+00	2.83E-164	up	3.96E+00	1.94E-47	up
ENSMUSG00000042943	4922501L14Rik	-5.05E+00	4.12E-141	down	-2.32E+00	1.66E-09	down
ENSMUSG00000063273	Naa15	-1.52E+00	3.59E-06	down	-1.98E+00	5.57E-11	down
ENSMUSG00000027751	Supt20	-3.19E+00	4.01E-112	down	-2.53E+00	5.39E-66	down
ENSMUSG00000027822	Slc33a1	-3.39E+00	8.48E-08	down	-3.00E+00	1.04E-04	down
ENSMUSG00000027938	Creb3l4	-1.96E+00	5.66E-81	down	3.40E-01	1.15E-01	no
ENSMUSG00000046722	Cdc42se1	-2.02E+00	4.07E-09	down	-1.66E+00	6.30E-06	down
ENSMUSG00000027530	Fabp12	-2.81E+00	6.31E-10	down	-8.13E-01	3.83E-01	no
ENSMUSG00000027531	Impa1	-1.88E+00	5.93E-04	down	-2.48E+00	6.33E-11	down
ENSMUSG00000014601	Strip1	-5.70E-01	5.51E-01	no	-2.30E+00	2.54E-05	down
ENSMUSG00000027606	Dnajc5b	-1.90E+00	5.74E-65	down	-3.52E-02	9.25E-01	no
ENSMUSG00000039335	Spata16	-2.67E+00	3.06E-92	down	-2.15E+00	2.54E-80	down
ENSMUSG00000027703	Lrriq4	-3.86E+00	1.43E-27	down	-1.09E+00	9.67E-02	no
ENSMUSG00000045031	Cetn4	-2.00E+00	2.48E-15	down	-2.15E+00	1.42E-26	down
ENSMUSG00000015745	Plekho1	-3.68E+00	4.34E-09	down	-3.27E+00	1.45E-08	down
ENSMUSG00000040562	Gstm2	4.53E+00	4.22E-20	up	2.62E+00	1.73E-07	up
ENSMUSG00000048416	Mlf1	-1.54E+00	2.00E-120	down	-5.19E-01	2.13E-08	no
ENSMUSG00000001415	Smg5	-3.06E+00	2.86E-30	down	-3.08E+00	1.50E-38	down
ENSMUSG00000038393	Txnip	1.50E-01	8.72E-01	no	-2.72E+00	3.99E-08	down
ENSMUSG00000028104	Polr3gl	4.70E-01	3.91E-01	no	-1.68E+00	8.18E-07	down
ENSMUSG00000000563	Atp5f1	1.50E+00	8.79E-05	up	-2.50E-01	6.23E-01	no
ENSMUSG00000027883	Gpsm2	-1.26E+00	1.11E-01	no	-2.96E+00	1.03E-06	down
ENSMUSG00000027963	Extl2	-2.85E+00	2.38E-04	down	-3.34E+00	5.69E-07	down
ENSMUSG00000051860	Samd7	-2.09E+00	2.52E-01	no	-2.95E+00	4.42E-04	down
ENSMUSG00000028048	Gba	2.96E+00	5.99E-04	up	2.49E+00	7.39E-04	up
ENSMUSG00000028089	Chd1l	-2.14E+00	6.43E-13	down	-2.41E+00	1.41E-22	down
ENSMUSG00000045662	Henmt1	-2.45E+00	1.80E-03	no	-2.92E+00	8.27E-10	down
ENSMUSG00000027782	Kpna4	-2.19E+00	2.76E-09	down	-2.57E+00	8.77E-11	down
ENSMUSG00000027956	Tmem144	-2.78E+00	3.35E-17	down	5.46E-02	9.59E-01	no
ENSMUSG00000038543	BC028528	7.97E+00	6.85E-22	up	5.62E+00	1.97E-06	up
ENSMUSG00000037661	Gpr160	-1.76E+00	1.59E-09	down	-2.00E+00	1.05E-18	down
ENSMUSG00000089911	Hiat1	-2.09E+00	7.06E-11	down	-2.52E+00	3.28E-16	down
ENSMUSG00000028159	Dapp1	-2.76E+00	1.42E-04	down	-3.30E+00	2.52E-10	down
ENSMUSG00000028188	Spata1	-2.80E+00	8.12E-51	down	-2.59E+00	6.63E-40	down
ENSMUSG00000039988	Ankrd13c	-2.51E+00	3.61E-08	down	-2.35E+00	1.46E-06	down
ENSMUSG00000004897	Hdgf	-1.62E+00	2.70E-28	down	-1.94E+00	3.10E-58	down
ENSMUSG00000041912	Tdrkh	-2.13E-01	7.77E-01	no	-1.73E+00	4.62E-05	down
ENSMUSG00000038205	Prkab2	-1.28E+00	2.38E-01	no	-2.64E+00	6.41E-04	down
ENSMUSG00000027881	Prpf38b	-9.99E-01	3.14E-02	no	-1.97E+00	7.64E-07	down
ENSMUSG00000019710	Mrpl24	-8.73E-01	3.53E-03	no	-1.77E+00	9.18E-22	down
ENSMUSG00000049565	Aknad1	-3.41E+00	2.43E-04	down	-1.07E+00	6.58E-01	no
ENSMUSG00000041842	Fhdc1	-5.45E+00	1.58E-13	down	-3.19E+00	2.59E-02	no
ENSMUSG00000027865	Gdap2	-1.99E+00	3.99E-04	down	-1.75E+00	1.22E-02	no

ENSMUSG00000044098	Rsbn1	-2.87E+00	4.09E-13	down	-1.20E+00	7.17E-02	no
ENSMUSG00000027671	Actl6a	-2.33E+00	2.56E-16	down	-1.77E+00	1.19E-09	down
ENSMUSG00000008763	Man1a2	-2.20E+00	6.30E-05	down	-2.56E+00	1.47E-06	down
ENSMUSG00000068747	Sort1	-4.31E+00	3.24E-17	down	-1.81E+00	5.60E-02	no
ENSMUSG00000042642	Flad1	-1.92E+00	2.86E-04	down	-1.27E+00	9.78E-03	no
ENSMUSG00000039735	Fnbp1l	-2.39E+00	6.51E-04	down	-2.16E+00	3.46E-02	no
ENSMUSG00000027770	Dhx36	-1.47E+00	1.11E-02	no	-2.27E+00	2.11E-05	down
ENSMUSG00000043020	Wdr63	-3.82E+00	3.62E-23	down	-3.62E+00	1.21E-31	down
ENSMUSG00000027680	Fxr1	-3.51E+00	3.45E-200	down	-2.79E+00	1.54E-106	down
ENSMUSG00000027804	Ppid	-9.29E-01	1.99E-01	no	-2.50E+00	7.95E-07	down
ENSMUSG00000033233	Trim45	-3.78E+00	1.39E-04	down	-1.93E+00	1.28E-01	no
ENSMUSG00000005687	Bcas2	7.20E-01	1.11E-03	no	1.08E+00	2.56E-05	up
ENSMUSG00000056260	Lrif1	-2.69E+00	6.74E-19	down	-7.26E-01	1.43E-01	no
ENSMUSG00000027719	Adad1	-2.65E+00	5.72E-67	down	-1.85E+00	6.75E-26	down
ENSMUSG00000033767	D930015E06Rik	-2.65E+00	1.93E-15	down	-4.21E-01	7.08E-01	no
ENSMUSG00000042672	Dcst1	-3.16E+00	5.48E-18	down	1.10E-01	9.25E-01	no
ENSMUSG00000074579	Lekr1	-2.21E+00	2.39E-04	down	-1.35E+00	4.39E-02	no
ENSMUSG00000046519	Golph3l	-2.46E+00	2.49E-03	no	-3.67E+00	1.61E-08	down
ENSMUSG00000033147	Slc22a15	-3.49E+00	2.10E-05	down	-2.40E+00	1.55E-02	no
ENSMUSG00000000339	Rtca	-1.65E+00	2.85E-08	down	-1.74E+00	1.15E-10	down
ENSMUSG00000028158	Mttp	-3.56E+00	1.13E-05	down	-4.12E+00	1.62E-05	down
ENSMUSG00000033014	Trim33	-3.21E+00	6.45E-06	down	-3.49E+00	5.08E-07	down
ENSMUSG00000032952	Ap4b1	-1.97E+00	2.99E-06	down	-2.49E+00	3.35E-11	down
ENSMUSG00000023084	Lrrc71	-2.14E+00	1.22E-09	down	-2.98E+00	4.25E-39	down
ENSMUSG00000027939	Nup210l	-3.98E+00	8.69E-19	down	-3.44E+00	1.96E-17	down
ENSMUSG00000027971	Ndst4	-2.53E+00	4.98E-04	down	-1.95E+00	1.41E-02	no
ENSMUSG00000028018	Gstcd	-4.10E+00	1.51E-04	down	-2.92E+00	6.19E-02	no
ENSMUSG00000019338	Zfp687	-2.87E+00	1.50E-05	down	-2.57E+00	2.71E-04	down
ENSMUSG00000005968	Tuft1	-1.16E+00	1.51E-01	no	-3.09E+00	2.84E-08	down
ENSMUSG00000053897	Slc39a8	-3.86E+00	2.38E-06	down	-3.60E+00	1.39E-05	down
ENSMUSG00000028161	Ppp3ca	-1.86E+00	2.79E-06	down	-2.76E+00	6.80E-23	down
ENSMUSG00000028102	Pex11b	-1.52E+00	2.69E-03	no	-1.78E+00	1.14E-05	down
ENSMUSG00000037174	Elf2	-2.41E+00	3.84E-18	down	-2.72E+00	3.26E-35	down
ENSMUSG00000027787	Nmd3	2.73E-01	5.36E-01	no	-1.37E+00	1.78E-05	down
ENSMUSG00000001017	Chtop	-5.71E-01	1.77E-01	no	-2.21E+00	1.34E-19	down
ENSMUSG00000033502	Cdc14a	-5.42E+00	1.88E-13	down	-2.85E+00	5.59E-02	no
ENSMUSG00000045328	Cenpe	-2.11E+00	4.19E-07	down	-1.49E+00	2.37E-03	no
ENSMUSG00000048655	Ccdc169	-2.15E+00	5.81E-14	down	-3.40E-01	6.80E-01	no
ENSMUSG00000038298	Pdzk1	-3.92E+00	4.12E-50	down	-1.13E+00	3.87E-02	no
ENSMUSG00000068922	Msto1	-1.60E+00	9.04E-08	down	-9.13E-01	3.19E-03	no
ENSMUSG00000027708	Dcun1d1	-3.53E+00	3.47E-14	down	-2.26E+00	6.20E-04	down
ENSMUSG00000028049	Scamp3	-8.49E-01	1.43E-01	no	-1.87E+00	1.41E-05	down
ENSMUSG00000027998	Plrg1	-7.48E-01	1.15E-01	no	-1.77E+00	1.33E-07	down
ENSMUSG00000028132	Tmem56	-4.34E+00	1.43E-08	down	-3.43E+00	1.53E-02	no
ENSMUSG00000028013	Ppa2	-2.43E+00	2.01E-19	down	-2.49E+00	2.96E-35	down
ENSMUSG00000028180	Zranb2	-1.01E+00	5.33E-03	no	-1.78E+00	1.41E-07	down
ENSMUSG00000027601	Mtfr1	-2.81E+00	1.50E-31	down	-1.88E+00	1.91E-11	down
ENSMUSG00000027676	Ccdc39	-3.19E+00	5.86E-13	down	-3.67E+00	1.28E-24	down
ENSMUSG00000028099	Polr3c	-1.47E+00	2.60E-03	no	-2.63E+00	6.07E-14	down
ENSMUSG00000027985	Lef1	-1.33E+00	7.83E-02	no	-2.24E+00	3.38E-12	down
ENSMUSG00000050150	Slc9b1	-2.65E+00	1.80E-23	down	-2.39E+00	5.33E-28	down
ENSMUSG00000027722	Spata5	-1.38E+00	2.63E-04	down	-2.73E+00	7.38E-15	down
ENSMUSG00000027942	4933434E20Rik	-2.51E+00	2.67E-13	down	-2.03E+00	1.37E-07	down
ENSMUSG00000027714	Exosc9	-8.23E-01	2.96E-01	no	-2.02E+00	1.26E-04	down
ENSMUSG00000028136	Snx27	-4.64E-01	5.47E-01	no	-2.09E+00	3.71E-07	down
ENSMUSG00000038861	Pi4kb	-2.06E+00	8.33E-02	no	-4.47E+00	5.63E-07	down
ENSMUSG00000038495	Otud7b	-3.44E+00	7.20E-10	down	-3.54E+00	9.75E-12	down
ENSMUSG00000027867	Spag17	-2.29E+00	4.60E-20	down	-1.53E+00	6.83E-10	down
ENSMUSG00000028152	Tspan5	-1.10E+00	2.99E-01	no	-2.65E+00	8.37E-05	down
ENSMUSG00000038712	Fam63a	-1.63E+00	1.42E-01	no	-3.21E+00	4.72E-07	down
ENSMUSG00000027893	Ahcyl1	-1.45E+00	5.26E-02	no	-2.55E+00	8.64E-05	down
ENSMUSG00000027665	Pik3ca	-1.82E+00	1.99E-01	no	-3.62E+00	7.93E-04	down
ENSMUSG00000028086	Fbxw7	-2.35E+00	1.90E-03	no	-2.98E+00	1.02E-05	down
ENSMUSG00000037325	Bbs7	-3.28E+00	1.22E-15	down	-2.92E+00	5.63E-13	down

ENSMUSG00000001052	Sec24b	-3.50E+00	8.97E-22	down	-1.84E+00	5.06E-04	down
ENSMUSG00000028256	Odf2l	-1.98E+00	1.93E-05	down	-2.54E+00	1.98E-10	down
ENSMUSG00000036745	Ttll7	-2.39E+00	9.90E-09	down	5.70E-01	6.28E-01	no
ENSMUSG00000028108	Ecm1	4.44E+00	6.89E-18	up	2.72E+00	6.10E-03	no
ENSMUSG00000036825	Ssx2ip	-3.21E+00	3.41E-43	down	-2.14E+00	9.32E-15	down
ENSMUSG00000027668	Mfn1	-1.91E+00	4.95E-04	down	-2.30E+00	4.11E-05	down
ENSMUSG00000015748	Prpf3	-4.27E-01	6.93E-01	no	-2.62E+00	2.80E-05	down
ENSMUSG00000033721	Vav3	-3.66E+00	1.73E-04	down	-1.17E+00	6.75E-01	no
ENSMUSG00000034151	Zbbx	-5.04E+00	7.54E-131	down	-3.38E+00	9.78E-31	down
ENSMUSG00000025757	Hspa4l	-2.95E+00	1.92E-56	down	-2.23E+00	1.23E-29	down
ENSMUSG00000025758	Plk4	-2.73E+00	8.28E-09	down	-2.53E+00	2.29E-07	down
ENSMUSG00000040412	5330417C22Rik	-3.34E+00	1.04E-30	down	-2.60E+00	3.00E-20	down
ENSMUSG00000027977	Ndst3	-4.51E+00	4.65E-04	down	-2.29E+00	4.87E-01	no
ENSMUSG00000028109	Hormad1	6.10E-01	5.72E-03	no	-2.19E+00	8.19E-53	down
ENSMUSG00000027674	Pex5l	-4.06E+00	2.63E-16	down	-1.96E+00	5.28E-02	no
ENSMUSG00000042520	Ubap2l	-1.85E+00	6.61E-12	down	-1.59E+00	3.18E-06	down
ENSMUSG00000001416	Cct3	-1.05E+00	1.99E-10	down	-1.31E+00	7.67E-21	down
ENSMUSG00000059834	Sclt1	-4.08E+00	4.74E-38	down	-2.91E+00	3.94E-11	down
ENSMUSG00000064068	Mtx1	-1.33E+00	3.03E-10	down	-9.11E-01	6.39E-05	no
ENSMUSG00000055436	Srsf11	-7.47E-02	8.84E-01	no	-1.54E+00	1.54E-07	down
ENSMUSG00000068921	Dap3	-1.42E+00	5.07E-07	down	-1.56E+00	9.30E-13	down
ENSMUSG00000008730	Hipk1	-2.94E+00	2.23E-07	down	-1.86E+00	8.38E-03	no
ENSMUSG00000068917	Clk2	-2.70E+00	8.24E-04	down	-3.55E+00	7.80E-06	down
ENSMUSG00000054199	Gon4l	-3.16E+00	4.91E-03	no	-3.59E+00	7.92E-05	down
ENSMUSG00000028047	Thbs3	-1.10E+00	1.14E-01	no	-2.45E+00	1.67E-04	down
ENSMUSG00000034349	Smc4	-1.06E+00	3.27E-08	down	-1.76E+00	3.62E-37	down
ENSMUSG00000039831	Arhgap29	-4.28E+00	2.56E-39	down	-3.60E+00	1.40E-26	down
ENSMUSG00000015697	Setdb1	-1.04E+00	1.66E-01	no	-3.01E+00	3.49E-07	down
ENSMUSG00000027550	Lrrcc1	-3.94E+00	6.05E-42	down	-3.71E+00	1.96E-45	down
ENSMUSG00000037400	Atp11b	-1.03E+00	3.40E-01	no	-3.05E+00	4.66E-05	down
ENSMUSG00000028064	Sema4a	-4.57E+00	6.06E-08	down	-2.93E+00	7.62E-02	no
ENSMUSG00000027778	Ift80	-2.09E+00	6.25E-03	no	-3.41E+00	3.28E-11	down
ENSMUSG00000027699	Ect2	-2.21E+00	1.21E-03	no	-2.82E+00	1.16E-05	down
ENSMUSG00000058388	Phtf1	-1.94E+00	2.69E-05	down	-2.34E+00	2.53E-08	down
ENSMUSG00000038170	Pde4dip	-2.73E+00	1.07E-05	down	-2.62E+00	2.53E-07	down
ENSMUSG00000028059	Arhgef2	-3.48E+00	1.32E-22	down	-5.13E-01	5.68E-01	no
ENSMUSG00000032826	Ank2	-4.87E+00	3.82E-05	down	-1.49E+00	6.25E-01	no
ENSMUSG00000068240	Gm11808	1.12E+00	3.66E-97	up	1.01E+00	5.92E-52	up
ENSMUSG00000070999	Ccin	-4.21E+00	1.58E-79	down	-1.77E+00	5.98E-05	down
ENSMUSG00000070997	1700055D18Rik	-2.55E+00	1.97E-05	down	-3.18E+00	1.02E-05	down
ENSMUSG00000070980	Actl7b	-2.56E+00	7.06E-40	down	-3.82E-01	2.96E-01	no
ENSMUSG00000070979	Actl7a	-3.07E+00	2.90E-188	down	-2.00E-01	4.37E-01	no
ENSMUSG00000070934	Rraga	-1.80E+00	8.10E-03	no	-2.40E+00	1.77E-04	down
ENSMUSG00000070923	Klhl9	-4.23E-01	7.33E-01	no	-3.16E+00	5.12E-07	down
ENSMUSG00000054351	4930553M12Rik	-2.57E+00	1.73E-07	down	-6.01E-01	7.13E-01	no
ENSMUSG00000076438	Oxct2b	-6.67E+00	4.51E-40	down	-5.33E+00	6.10E-10	down
ENSMUSG00000076436	Oxct2a	-5.98E+00	2.16E-45	down	-5.07E+00	2.95E-10	down
ENSMUSG00000048686	Hmgb4	-2.20E+00	1.37E-285	down	-1.07E+00	1.63E-21	down
ENSMUSG00000051276	Actrt2	-3.57E+00	1.49E-193	down	-1.15E+00	7.39E-08	down
ENSMUSG00000044556	Tex38	-1.74E+00	2.76E-25	down	7.33E-01	1.49E-02	no
ENSMUSG00000078593	1700042G07Rik	-1.92E+00	3.34E-37	down	-1.08E+00	2.42E-04	down
ENSMUSG00000078577	Tmco2	-3.10E+00	7.35E-249	down	-1.34E+00	3.24E-17	down
ENSMUSG00000073761	4933427I04Rik	-4.87E+00	7.71E-25	down	-3.92E+00	2.81E-07	down
ENSMUSG00000000411	Tssk3	-2.37E+00	5.16E-52	down	-2.58E-01	4.93E-01	no
ENSMUSG00000047945	Marcksl1	2.52E+00	1.01E-06	up	5.83E-01	3.68E-01	no
ENSMUSG00000046694	Fam46b	-3.27E+00	3.44E-14	down	-2.52E+00	7.50E-07	down
ENSMUSG00000036921	4930549C01Rik	-5.44E+00	4.88E-14	down	-4.49E+00	7.55E-04	down
ENSMUSG00000041399	1700013G24Rik	-2.40E+00	2.22E-155	down	-7.69E-02	8.06E-01	no
ENSMUSG00000046447	Camk2n1	-4.07E+00	5.29E-06	down	-3.90E+00	1.20E-06	down
ENSMUSG00000028287	1700009N14Rik	-5.18E+00	4.98E-263	down	-2.92E+00	3.82E-35	down
ENSMUSG00000028310	Ppp3r2	-1.79E+00	4.49E-55	down	-2.04E+00	8.05E-111	down
ENSMUSG00000059343	Aldoart1	-2.41E+00	4.04E-28	down	7.05E-02	9.29E-01	no
ENSMUSG00000048626	Klf17	-3.76E+00	3.95E-11	down	-1.02E-01	9.57E-01	no
ENSMUSG00000036905	C1qb	3.60E+00	1.08E-42	up	3.47E+00	1.47E-07	up

ENSMUSG00000036887	C1qa	4.59E+00	2.84E-40	up	4.66E+00	5.55E-08	up
ENSMUSG00000006221	Hspb7	-3.52E+00	1.01E-08	down	-2.97E+00	1.24E-04	down
ENSMUSG00000044496	2510039O18Rik	4.07E-01	5.63E-01	no	-2.23E+00	1.60E-08	down
ENSMUSG00000036822	Topors	-1.45E+00	6.25E-05	down	-2.68E+00	4.84E-23	down
ENSMUSG00000028443	Nudt2	-1.76E+00	2.32E-04	down	-1.25E+00	3.48E-02	no
ENSMUSG00000094695	Gm21953	1.22E+00	8.84E-01	no	8.55E+00	1.61E-13	up
ENSMUSG00000095234	Gm21586	5.16E+00	3.33E-02	no	1.03E+01	5.21E-18	up
ENSMUSG00000039105	Atp6v1g1	2.48E+00	1.88E-47	up	4.96E-01	6.51E-06	no
ENSMUSG00000049969	Plekhf2	-4.78E-01	5.64E-01	no	-1.92E+00	1.21E-04	down
ENSMUSG00000028773	Fabp3	6.12E+00	2.04E-05	up	6.22E+00	1.74E-08	up
ENSMUSG00000040659	Efhd2	-1.89E+00	5.58E-08	down	-4.72E-01	5.39E-01	no
ENSMUSG00000028589	1700012P22Rik	-2.15E+00	2.62E-48	down	4.46E-01	8.24E-02	no
ENSMUSG00000047777	Phf13	-1.09E+00	5.14E-03	no	-2.54E+00	1.92E-19	down
ENSMUSG00000073700	Klhl21	-2.03E-01	8.27E-01	no	-2.62E+00	2.36E-05	down
ENSMUSG00000078722	Gm12394	-3.79E+00	2.24E-04	down	-7.19E-01	8.05E-01	no
ENSMUSG00000078721	Gm12429	-4.09E+00	3.41E-07	down	-1.34E+00	5.80E-01	no
ENSMUSG00000028314	4930547C10Rik	-3.96E+00	2.79E-87	down	-3.27E+00	8.53E-45	down
ENSMUSG00000028551	Cdkn2c	-2.54E+00	5.75E-12	down	-3.41E+00	7.59E-42	down
ENSMUSG00000047518	Slfnl1	-3.84E+00	1.56E-91	down	-1.34E+00	5.94E-05	down
ENSMUSG00000023263	9530002B09Rik	-2.35E+00	2.64E-03	no	-2.84E+00	8.33E-07	down
ENSMUSG00000078554	Fam229a	2.11E+00	5.23E-213	up	3.62E+00	2.26E-203	up
ENSMUSG00000036896	C1qc	4.44E+00	2.45E-18	up	4.53E+00	1.24E-03	no
ENSMUSG00000071015	Gm136	-7.66E+00	1.21E-39	down	-6.06E+00	5.11E-08	down
ENSMUSG00000028367	Txn1	3.82E+00	1.59E-10	up	2.44E+00	3.59E-03	no
ENSMUSG00000028517	Ppap2b	4.25E+00	1.85E-11	up	2.93E+00	8.33E-02	no
ENSMUSG00000090697	Nsun4	-6.63E+00	1.80E-31	down	-2.35E+00	1.73E-02	no
ENSMUSG00000042707	Dnali1	-1.98E+00	1.35E-37	down	-1.55E+00	2.86E-25	down
ENSMUSG00000000682	Cd52	3.64E+00	1.46E-05	up	1.56E+00	5.10E-01	no
ENSMUSG00000041556	Fbxo2	-5.65E+00	4.31E-13	down	-2.86E+00	1.59E-02	no
ENSMUSG00000039611	Tmem246	-2.36E+00	4.47E-06	down	-3.65E-01	7.60E-01	no
ENSMUSG00000028642	4930538K18Rik	-5.24E+00	1.35E-20	down	-3.07E+00	3.25E-04	down
ENSMUSG00000052137	Rbm12b2	-2.35E+00	4.44E-01	no	-4.99E+00	9.53E-04	down
ENSMUSG00000050213	Snip1	-1.69E+00	2.03E-06	down	-1.90E+00	1.45E-07	down
ENSMUSG00000028813	CK137956	-3.10E+00	5.82E-33	down	-3.67E+00	3.03E-67	down
ENSMUSG00000054428	Atpif1	2.20E+00	8.33E-162	up	1.72E+00	3.53E-131	up
ENSMUSG00000028843	Sh3bgrl3	2.73E+00	8.56E-21	up	1.16E+00	3.22E-03	no
ENSMUSG00000007872	Id3	5.43E+00	1.09E-16	up	3.88E+00	5.09E-02	no
ENSMUSG00000028291	Akirin2	-1.22E+00	1.95E-13	down	-1.58E+00	9.37E-24	down
ENSMUSG00000071014	Ndufb6	4.12E+00	2.13E-124	up	2.40E+00	5.54E-85	up
ENSMUSG00000028333	Anp32b	2.70E+00	8.86E-21	up	-3.99E-01	4.84E-02	no
ENSMUSG00000039634	Zfp189	-4.00E+00	6.56E-09	down	-3.57E+00	8.76E-08	down
ENSMUSG00000035212	Leprot	3.45E+00	4.01E-04	up	1.48E+00	2.46E-01	no
ENSMUSG00000028609	Magoh	5.18E-01	9.87E-02	no	-1.30E+00	3.87E-08	down
ENSMUSG00000045268	Zfp691	-2.52E+00	5.11E-06	down	-1.57E+00	5.73E-02	no
ENSMUSG00000028629	Exo5	-1.87E+00	5.20E-02	no	-2.83E+00	6.53E-05	down
ENSMUSG00000028847	Trappc3	2.44E+00	8.15E-05	up	-4.88E-01	3.96E-01	no
ENSMUSG00000037752	Xkr8	-1.87E+00	3.60E-06	down	-1.75E+00	7.46E-07	down
ENSMUSG00000028675	Pnrc2	-1.07E+00	1.54E-01	no	-2.69E+00	9.54E-09	down
ENSMUSG00000041161	Otud3	-2.07E+00	2.27E-15	down	-1.20E+00	3.16E-04	down
ENSMUSG00000028419	Chmp5	-1.38E+00	4.18E-04	down	-1.79E+00	8.65E-05	down
ENSMUSG00000049657	Zbtb5	-3.28E+00	7.36E-07	down	-3.14E+00	2.93E-07	down
ENSMUSG00000028214	Gem	7.22E-01	4.54E-01	no	-2.83E+00	4.42E-06	down
ENSMUSG00000037242	Clic4	-3.35E-01	6.89E-01	no	-2.77E+00	7.72E-08	down
ENSMUSG00000041120	Nbl1	5.31E+00	5.20E-09	up	3.12E+00	1.46E-01	no
ENSMUSG00000046637	Ttc34	-2.44E+00	8.18E-05	down	-2.36E+00	3.48E-05	down
ENSMUSG00000028436	Dcaf12	-2.51E+00	2.97E-22	down	-1.91E+00	2.21E-12	down
ENSMUSG00000028332	Hemgn	-4.26E+00	7.08E-29	down	-2.33E+00	2.83E-03	no
ENSMUSG00000003032	Klf4	-2.75E+00	4.64E-36	down	-1.34E+00	1.27E-02	no
ENSMUSG00000060491	4930522H14Rik	-3.15E+00	5.87E-61	down	-1.21E+00	1.02E-03	no
ENSMUSG00000028643	Ccdc23	-1.48E+00	1.96E-22	down	-1.94E+00	1.19E-44	down
ENSMUSG00000028967	Errfi1	4.31E+00	1.15E-30	up	1.44E+00	2.65E-02	no
ENSMUSG00000028955	Vamp3	1.85E+00	1.50E-07	up	-1.66E+00	5.90E-14	down
ENSMUSG00000073991	Cnbd1	-4.09E+00	5.61E-17	down	-1.08E+00	2.64E-01	no
ENSMUSG00000043633	Fam221b	-3.76E+00	1.28E-63	down	-1.48E+00	2.69E-05	down

ENSMUSG00000078713	Tomm5	3.56E+00	1.00E-05	up	7.00E-01	2.29E-01	no
ENSMUSG00000058935	1700018C11Rik	-2.78E+00	2.69E-147	down	-8.44E-01	2.19E-05	no
ENSMUSG00000028610	Dmrtb1	-1.74E+00	2.27E-88	down	-1.92E+00	6.31E-140	down
ENSMUSG00000028567	Txndc12	-1.07E+00	2.11E-02	no	-2.37E+00	4.22E-18	down
ENSMUSG00000028648	Ndufs5	2.60E+00	1.58E-12	up	1.90E+00	4.52E-12	up
ENSMUSG00000023075	Akirin1	-1.65E+00	2.21E-06	down	-1.56E+00	7.94E-05	down
ENSMUSG00000028779	Pef1	-2.05E-01	6.84E-01	no	-1.55E+00	1.22E-09	down
ENSMUSG00000028668	Tceb3	-5.24E-01	5.09E-01	no	-2.46E+00	2.44E-07	down
ENSMUSG00000028583	Pdpn	4.55E+00	8.78E-05	up	2.85E+00	3.50E-01	no
ENSMUSG00000067916	Gm13139	-3.45E+00	6.94E-05	down	-3.05E+00	2.66E-04	down
ENSMUSG00000029038	Ssu72	2.39E+00	6.85E-05	up	-3.90E-01	4.38E-01	no
ENSMUSG00000078639	Gm12695	-2.40E+00	4.12E-04	down	6.41E-01	7.25E-01	no
ENSMUSG00000028889	Yrdc	1.67E+00	8.60E-05	up	-4.30E-01	2.24E-01	no
ENSMUSG00000023120	Gm853	7.20E+00	9.83E-05	up	7.68E+00	3.68E-05	up
ENSMUSG00000006699	Cdc42	1.82E+00	3.32E-05	up	-5.40E-01	2.46E-01	no
ENSMUSG00000039492	Ccdc27	-4.33E+00	6.50E-58	down	-1.11E+00	3.71E-02	no
ENSMUSG00000029073	Gltpd1	-2.14E+00	2.15E-22	down	-1.47E+00	4.31E-12	down
ENSMUSG00000040520	Manea	-2.23E+00	1.66E-01	no	-3.80E+00	3.63E-04	down
ENSMUSG00000028389	Zfp37	-4.81E+00	2.48E-73	down	-2.91E+00	1.86E-09	down
ENSMUSG00000006398	Cdc20	-2.81E-01	2.25E-01	no	-1.34E+00	1.41E-20	down
ENSMUSG00000028851	Nudc	1.47E+00	2.63E-08	up	4.66E-01	2.45E-02	no
ENSMUSG00000056300	Gm13247	-3.96E+00	1.63E-03	no	-4.26E+00	1.17E-05	down
ENSMUSG00000028259	Fhl5	-3.09E+00	1.02E-126	down	-9.51E-01	4.74E-05	no
ENSMUSG00000053317	Sec61b	2.18E+00	3.91E-05	up	8.43E-01	9.30E-02	no
ENSMUSG00000032870	Smap2	-2.15E+00	1.89E-04	down	-2.89E+00	1.63E-09	down
ENSMUSG00000028837	Psmb2	2.91E+00	3.38E-45	up	1.66E+00	5.68E-18	up
ENSMUSG00000028879	Stx12	2.26E-01	7.88E-01	no	-2.31E+00	4.33E-07	down
ENSMUSG00000028822	Tmem50a	4.81E+00	7.36E-45	up	3.93E+00	1.65E-14	up
ENSMUSG00000028416	Bag1	-1.56E+00	6.66E-36	down	-1.59E+00	1.17E-44	down
ENSMUSG00000050141	BC049635	-3.44E+00	3.30E-123	down	-7.79E-01	5.25E-03	no
ENSMUSG00000028322	Exosc3	-1.20E+00	7.11E-07	down	-1.83E+00	9.25E-22	down
ENSMUSG00000039555	Cylc2	-3.85E+00	9.59E-101	down	-1.34E+00	4.45E-04	down
ENSMUSG00000038709	Txndc8	-2.85E+00	2.62E-148	down	-9.95E-01	2.57E-07	no
ENSMUSG00000028533	Izumo3	-4.26E+00	1.05E-126	down	-1.70E+00	1.56E-08	down
ENSMUSG00000049225	Pdp1	-3.28E+00	2.25E-09	down	-1.13E+00	3.55E-01	no
ENSMUSG00000028784	Spocd1	2.07E+00	9.08E-07	up	1.74E+00	2.59E-05	up
ENSMUSG00000050608	Minos1	2.20E+00	3.93E-39	up	1.23E+00	1.02E-12	up
ENSMUSG00000040550	Otud6b	-2.96E+00	1.02E-08	down	-1.56E+00	6.31E-02	no
ENSMUSG00000006442	Srm	2.42E+00	6.41E-04	up	1.30E+00	1.03E-01	no
ENSMUSG00000029030	Tprgl	-1.24E+00	1.01E-12	down	-1.53E+00	3.48E-28	down
ENSMUSG00000023571	Fam132a	3.65E+00	1.89E-13	up	2.85E+00	2.62E-12	up
ENSMUSG00000015242	Nipsnap3a	-2.14E+00	2.66E-20	down	5.74E-01	2.54E-01	no
ENSMUSG00000058046	4933430I17Rik	-4.28E+00	1.21E-20	down	-1.92E+00	5.45E-04	down
ENSMUSG00000028492	Fam154a	-3.65E+00	6.06E-32	down	-9.88E-01	1.58E-01	no
ENSMUSG00000063882	Uqcrh	1.44E+00	2.39E-38	up	2.46E-02	8.82E-01	no
ENSMUSG00000070806	Zmynd12	-4.39E+00	1.73E-25	down	-4.14E+00	4.46E-23	down
ENSMUSG00000043621	Ubxn10	-3.40E+00	1.08E-21	down	-2.72E+00	1.99E-11	down
ENSMUSG00000078503	Gm13225	-2.84E+00	1.51E-02	no	-4.34E+00	2.64E-04	down
ENSMUSG00000078485	Plekhn1	-3.83E+00	1.46E-07	down	-3.72E+00	5.27E-10	down
ENSMUSG00000028447	Dctn3	5.06E-01	1.37E-02	no	1.17E+00	7.59E-10	up
ENSMUSG00000066000	2610305D13Rik	-3.96E+00	2.38E-02	no	-4.50E+00	6.89E-05	down
ENSMUSG00000066196	Spag8	-1.91E+00	1.32E-12	down	-1.05E+00	1.17E-05	down
ENSMUSG00000028331	5830415F09Rik	-1.43E+00	1.47E-02	no	-1.81E+00	2.82E-04	down
ENSMUSG00000039813	Tbc1d2	-2.68E+00	3.39E-04	down	-1.18E+00	4.06E-01	no
ENSMUSG00000028420	Tmem38b	-5.17E-01	6.18E-01	no	-2.62E+00	8.85E-07	down
ENSMUSG00000028392	Bspry	-1.76E+00	7.04E-42	down	-1.14E+00	1.14E-18	down
ENSMUSG00000028575	Eqtn	-3.63E+00	5.86E-116	down	-1.17E+00	1.20E-04	down
ENSMUSG00000063800	Prpf38a	-1.34E+00	1.33E-02	no	-1.68E+00	6.13E-04	down
ENSMUSG00000078552	Dcdc2b	-3.91E-01	8.25E-01	no	-2.34E+00	6.63E-04	down
ENSMUSG00000028750	Pla2g2c	-2.57E+00	5.88E-08	down	-1.80E+00	6.38E-06	down
ENSMUSG00000040842	Szrd1	-7.08E-01	4.12E-01	no	-2.48E+00	6.01E-05	down
ENSMUSG00000039662	Icmt	-2.35E+00	1.71E-06	down	-2.92E+00	5.23E-16	down
ENSMUSG00000029048	Rer1	-1.19E+00	2.58E-06	down	-1.39E+00	2.92E-08	down
ENSMUSG00000056494	Cngb3	-4.47E+00	1.53E-04	down	-1.71E+00	5.46E-01	no

ENSMUSG00000028673	Fuca1	-1.05E+00	9.54E-03	no	-1.33E+00	2.87E-04	down
ENSMUSG00000047675	Rps8	4.00E+00	2.85E-05	up	3.20E+00	1.32E-04	up
ENSMUSG00000048772	Tmem53	-1.27E-01	5.16E-01	no	1.73E+00	1.79E-15	up
ENSMUSG00000042500	Ago4	-1.21E+00	4.33E-01	no	-3.03E+00	3.82E-04	down
ENSMUSG00000028832	Stmn1	4.57E+00	1.21E-116	up	3.24E+00	5.64E-123	up
ENSMUSG00000028756	Pink1	-1.47E+00	7.37E-11	down	-2.24E+00	9.11E-36	down
ENSMUSG00000045004	Spata21	-4.28E+00	1.11E-241	down	-1.67E+00	1.36E-13	down
ENSMUSG00000028990	Lzic	-1.38E+00	1.45E-03	no	-1.90E+00	7.63E-08	down
ENSMUSG00000028277	Ube2j1	-2.10E+00	2.57E-37	down	-1.11E+00	4.00E-07	down
ENSMUSG00000028264	Spaca1	-1.87E+00	2.57E-53	down	3.61E-01	1.76E-01	no
ENSMUSG00000028426	Rad23b	-4.09E+00	1.58E-52	down	-3.72E+00	2.58E-38	down
ENSMUSG00000038827	BC026590	-2.43E-01	8.59E-01	no	-2.49E+00	8.15E-06	down
ENSMUSG00000028607	Cpt2	-1.19E+00	5.89E-04	down	-1.64E+00	9.16E-09	down
ENSMUSG00000057236	Rbbp4	-2.08E+00	2.38E-04	down	-3.49E+00	1.81E-23	down
ENSMUSG00000001089	Luzp1	-2.61E+00	1.25E-16	down	-3.96E-01	7.08E-01	no
ENSMUSG00000023151	Lrrc69	-5.19E+00	3.55E-30	down	-3.44E+00	2.53E-05	down
ENSMUSG00000028343	Erp44	-1.47E+00	1.60E-03	no	-2.44E+00	1.21E-07	down
ENSMUSG00000066154	Mup3	4.42E+00	1.09E-04	up	2.37E+00	1.60E-01	no
ENSMUSG00000028523	Tctex1d1	-4.02E+00	1.08E-16	down	-1.85E+00	4.50E-02	no
ENSMUSG00000028878	Fam76a	-2.47E+00	1.82E-32	down	-1.37E+00	1.06E-06	down
ENSMUSG00000028801	Stpg1	-4.43E+00	1.73E-40	down	-1.83E+00	6.99E-04	down
ENSMUSG00000039911	Spsb1	-2.13E+00	1.25E-07	down	5.20E-01	6.07E-01	no
ENSMUSG00000073791	Efcab7	-2.37E+00	2.92E-04	down	-3.80E+00	1.07E-20	down
ENSMUSG00000025791	Pgm2	4.85E+00	2.03E-12	up	4.26E+00	1.24E-10	up
ENSMUSG00000028698	Pik3r3	-2.19E+00	8.45E-05	down	-3.56E+00	3.01E-16	down
ENSMUSG00000060268	Gm1661	-3.33E+00	3.48E-32	down	-2.50E+00	5.51E-20	down
ENSMUSG00000029028	Lrrc47	-2.87E+00	2.46E-07	down	-2.71E+00	2.05E-05	down
ENSMUSG00000028427	Aqp7	-3.61E+00	8.35E-48	down	-9.96E-01	4.63E-02	no
ENSMUSG00000028437	Ubap1	-1.67E+00	5.96E-08	down	-2.16E+00	7.29E-15	down
ENSMUSG00000036052	Dnajb5	-4.24E+00	4.65E-04	down	-5.15E+00	8.35E-05	down
ENSMUSG00000054362	BC055111	-5.63E+00	5.42E-17	down	-4.22E+00	9.02E-04	down
ENSMUSG00000063172	Hspb11	5.46E-02	9.37E-01	no	-1.54E+00	9.53E-04	down
ENSMUSG00000042616	Oscp1	-2.44E+00	7.68E-42	down	-1.71E+00	1.58E-23	down
ENSMUSG00000028788	Ptp4a2	-1.48E+00	1.53E-04	down	-2.46E+00	3.11E-12	down
ENSMUSG00000028936	Rpl22	-9.64E-01	7.22E-02	no	-2.66E+00	3.69E-10	down
ENSMUSG00000039523	Cep104	-2.89E+00	4.60E-14	down	-2.34E+00	6.96E-09	down
ENSMUSG00000028560	Usp1	-3.61E+00	6.01E-96	down	-3.22E+00	3.46E-93	down
ENSMUSG00000028550	Atg4c	-2.41E+00	9.01E-03	no	-3.04E+00	1.70E-04	down
ENSMUSG00000028692	Akr1a1	3.12E+00	6.46E-20	up	4.02E-01	1.23E-01	no
ENSMUSG00000028902	Sf3a3	-4.20E-01	2.94E-01	no	-1.01E+00	3.58E-04	down
ENSMUSG00000028882	Ppp1r8	6.19E-01	5.06E-01	no	-2.55E+00	1.58E-06	down
ENSMUSG00000057637	Prdm2	-3.58E+00	3.70E-13	down	-3.32E+00	3.67E-12	down
ENSMUSG00000028295	Smim8	-7.74E-01	7.95E-05	no	-1.04E+00	5.28E-11	down
ENSMUSG00000028409	Smu1	-2.59E-01	5.00E-01	no	-1.12E+00	3.03E-05	down
ENSMUSG00000036078	Sigmar1	-9.75E-01	4.20E-01	no	-2.66E+00	7.91E-04	down
ENSMUSG00000039158	Akna	-2.70E+00	2.63E-09	down	8.54E-02	9.54E-01	no
ENSMUSG00000028520	4921539E11Rik	-4.09E+00	1.15E-51	down	-2.12E+00	5.69E-06	down
ENSMUSG00000028729	Ebna1bp2	-1.11E+00	8.68E-03	no	-2.10E+00	8.01E-13	down
ENSMUSG00000028639	Ybx1	-3.10E+00	3.24E-245	down	-3.24E+00	0.00E+00	down
ENSMUSG00000028646	Rragc	-9.10E-01	1.24E-01	no	-2.10E+00	5.07E-06	down
ENSMUSG00000028790	Khdrbs1	-8.36E-01	2.83E-01	no	-2.49E+00	1.31E-07	down
ENSMUSG00000028884	Rpa2	3.37E+00	2.72E-10	up	6.43E-01	1.51E-01	no
ENSMUSG00000028439	Fam219a	-2.67E+00	3.54E-07	down	-2.72E+00	3.31E-07	down
ENSMUSG00000029656	C8b	-1.65E+00	1.13E-02	no	-1.78E+00	3.69E-04	down
ENSMUSG00000059482	2610301B20Rik	-1.66E+00	2.25E-07	down	-2.69E+00	1.93E-34	down
ENSMUSG00000010517	Faf1	-2.23E+00	6.46E-09	down	-3.07E+00	6.14E-18	down
ENSMUSG00000028868	Wasf2	-1.84E+00	4.33E-04	down	-2.10E-01	8.83E-01	no
ENSMUSG00000028854	Slc9a1	-3.55E+00	1.67E-06	down	-2.54E+00	4.53E-02	no
ENSMUSG00000048003	Catsper4	-3.24E+00	1.52E-58	down	-1.52E-01	8.03E-01	no
ENSMUSG00000028224	Nbn	-2.17E+00	9.40E-05	down	-2.76E+00	1.27E-07	down
ENSMUSG00000061322	Dnaic1	-1.60E+00	6.45E-14	down	-9.06E-01	3.92E-06	no
ENSMUSG00000028383	Hsdl2	-4.31E+00	2.96E-41	down	-2.58E+00	5.86E-09	down
ENSMUSG00000028691	Prdx1	2.80E+00	6.54E-27	up	6.07E-01	8.32E-02	no
ENSMUSG00000028452	Vcp	5.24E-01	3.19E-01	no	-1.17E+00	7.87E-04	down

ENSMUSG00000028328	Tmod1	-3.34E+00	8.69E-05	down	-1.12E+00	5.64E-01	no
ENSMUSG00000069733	Ube2u	-2.66E+00	2.06E-41	down	-2.07E+00	6.09E-32	down
ENSMUSG00000028716	Pdzk1ip1	-6.64E+00	3.26E-47	down	-4.40E+00	1.57E-08	down
ENSMUSG00000028703	Lrrc41	-1.97E+00	5.65E-08	down	-1.32E+00	2.45E-03	no
ENSMUSG00000034042	Gpbp1l1	-2.66E+00	1.15E-07	down	-3.63E+00	1.25E-14	down
ENSMUSG00000028669	Pithd1	-1.24E+00	1.15E-03	no	-1.77E+00	9.58E-12	down
ENSMUSG00000028976	Slc2a5	-3.16E+00	3.33E-126	down	-2.50E+00	8.10E-72	down
ENSMUSG00000028458	Tesk1	-5.56E+00	5.86E-84	down	-4.10E+00	1.58E-45	down
ENSMUSG00000038668	Lpar1	-2.24E+00	1.26E-04	down	2.77E-01	8.61E-01	no
ENSMUSG00000028657	Ppt1	-2.15E+00	2.38E-08	down	-6.44E-01	3.66E-01	no
ENSMUSG00000028863	Meaf6	-1.43E+00	3.73E-03	no	-1.93E+00	2.28E-06	down
ENSMUSG00000029070	Mxra8	-1.03E+00	4.96E-04	down	-1.41E+00	7.93E-07	down
ENSMUSG00000028890	Mtf1	-3.33E+00	2.19E-13	down	-1.70E+00	5.60E-03	no
ENSMUSG00000053730	Tmem39b	-2.91E+00	1.74E-17	down	-2.53E+00	2.66E-12	down
ENSMUSG00000028414	Fktn	-2.45E+00	3.39E-03	no	-3.15E+00	1.77E-08	down
ENSMUSG00000028826	Tmem57	-1.99E+00	1.57E-04	down	-2.98E+00	9.58E-10	down
ENSMUSG00000029076	Sdf4	-3.74E-01	4.01E-01	no	-1.64E+00	1.69E-08	down
ENSMUSG00000061455	Stx17	-1.95E+00	5.28E-04	down	-2.03E+00	1.11E-05	down
ENSMUSG00000028330	Ncbp1	-3.35E+00	5.33E-73	down	-3.16E+00	2.26E-78	down
ENSMUSG00000028494	Plin2	-1.81E+00	2.57E-09	down	1.12E+00	5.68E-02	no
ENSMUSG00000028845	Tekt2	-2.76E+00	2.64E-137	down	-6.57E-01	3.11E-05	no
ENSMUSG00000037266	D4Wsu53e	1.61E+00	5.58E-06	up	-1.47E+00	6.51E-13	down
ENSMUSG00000029074	Ttll10	-3.83E+00	5.68E-68	down	-9.24E-01	4.24E-02	no
ENSMUSG00000028869	Gnl2	-1.25E-02	9.83E-01	no	-2.12E+00	9.53E-13	down
ENSMUSG00000040859	Bsdc1	-2.96E+00	1.14E-07	down	-2.38E+00	1.21E-04	down
ENSMUSG00000028478	Clta	2.30E+00	3.48E-04	up	-3.64E-01	5.99E-01	no
ENSMUSG00000028549	Itgb3bp	-1.99E+00	6.68E-12	down	-2.65E+00	1.19E-27	down
ENSMUSG00000028820	Sfpq	-1.82E+00	8.55E-06	down	-3.07E+00	4.67E-20	down
ENSMUSG00000043003	Rasef	-3.92E+00	1.37E-09	down	-2.36E+00	4.60E-03	no
ENSMUSG00000028618	Tmem59	7.80E-01	1.15E-02	no	-1.34E+00	1.28E-07	down
ENSMUSG00000033985	Tesk2	-3.91E+00	1.15E-13	down	-2.17E+00	7.34E-03	no
ENSMUSG00000028441	1110017D15Rik	-2.56E+00	4.08E-71	down	-9.09E-01	6.03E-05	no
ENSMUSG00000042608	Stk40	-2.30E+00	1.01E-07	down	-1.84E+00	1.72E-03	no
ENSMUSG00000040945	Rcc2	-4.27E-01	4.95E-01	no	-2.12E+00	3.16E-11	down
ENSMUSG00000028454	Pigo	-2.15E+00	1.57E-03	no	-2.60E+00	2.18E-06	down
ENSMUSG00000028576	Ift74	-2.67E+00	1.03E-58	down	-2.55E+00	2.18E-55	down
ENSMUSG00000035601	Trmt10b	-2.16E+00	1.25E-03	no	-2.45E+00	3.84E-07	down
ENSMUSG00000028688	Toe1	-1.35E+00	1.99E-02	no	-1.99E+00	7.75E-05	down
ENSMUSG00000003731	Kpna6	-3.73E+00	4.39E-45	down	-2.39E+00	3.08E-11	down
ENSMUSG00000040761	Spen	-2.49E+00	1.17E-04	down	-2.09E+00	4.12E-03	no
ENSMUSG00000035517	Tdrd7	-3.60E+00	5.13E-58	down	-3.13E+00	3.14E-44	down
ENSMUSG00000033379	Atp6v0b	1.70E+00	7.05E-13	up	9.76E-02	8.07E-01	no
ENSMUSG00000033365	Ipo13	-2.98E+00	5.28E-20	down	-2.69E+00	6.28E-17	down
ENSMUSG00000028793	Rnf19b	-4.18E+00	6.56E-165	down	-3.56E+00	7.22E-94	down
ENSMUSG00000028937	Acot7	-1.15E+00	1.77E-37	down	-9.02E-01	2.05E-29	no
ENSMUSG00000028468	Rgp1	-2.85E+00	1.89E-02	no	-3.67E+00	9.23E-09	down
ENSMUSG00000046671	Mtfr1l	-1.07E+00	2.02E-03	no	-1.65E+00	9.29E-12	down
ENSMUSG00000028873	Cdca8	9.27E-01	7.46E-02	no	-1.04E+00	4.49E-04	down
ENSMUSG00000028730	Wdr65	-4.07E+00	1.19E-46	down	-3.71E+00	1.73E-54	down
ENSMUSG00000028581	Laptm5	4.09E+00	2.38E-05	up	3.98E+00	1.86E-01	no
ENSMUSG00000037366	Pafah2	-3.07E+00	1.65E-03	no	-4.17E+00	3.06E-13	down
ENSMUSG00000028294	1700003M02Rik	-3.39E+00	2.21E-280	down	-2.63E+00	1.31E-154	down
ENSMUSG00000028438	Kif24	-2.02E+00	1.60E-01	no	-3.35E+00	9.71E-04	down
ENSMUSG00000028347	Tmeff1	-3.21E+00	9.19E-08	down	-1.76E+00	1.07E-01	no
ENSMUSG00000037622	Wdtc1	-4.47E+00	4.70E-45	down	-3.52E+00	5.80E-29	down
ENSMUSG00000028758	Kif17	-5.52E+00	5.58E-68	down	-2.44E+00	3.53E-05	down
ENSMUSG00000041058	Wwp1	-2.46E+00	8.11E-05	down	-1.04E+00	5.35E-01	no
ENSMUSG00000028484	Psip1	7.86E-03	9.74E-01	no	-1.33E+00	1.16E-30	down
ENSMUSG00000028896	Rcc1	-3.64E+00	2.82E-22	down	-3.10E+00	1.03E-14	down
ENSMUSG00000051517	Arhgef39	-1.13E+00	3.62E-01	no	-2.63E+00	6.05E-06	down
ENSMUSG00000025413	Ttc4	-2.56E+00	6.26E-27	down	-2.91E+00	3.75E-44	down
ENSMUSG00000028218	Fam92a	-1.79E+00	1.09E-03	no	-1.86E+00	4.22E-04	down
ENSMUSG00000037443	Cep85	-6.83E-01	2.46E-01	no	-2.86E+00	5.92E-22	down
ENSMUSG00000028345	Tex10	-1.79E+00	1.43E-08	down	-1.69E+00	3.83E-07	down

ENSMUSG00000028753	Vwa5b1	-4.87E+00	5.66E-12	down	-2.00E+00	2.40E-01	no
ENSMUSG00000029009	Mthfr	-2.99E+00	6.28E-05	down	-3.11E+00	9.07E-08	down
ENSMUSG00000029063	Nadk	-4.93E-01	5.10E-01	no	-2.10E+00	3.05E-06	down
ENSMUSG00000006392	Med8	-6.67E-01	2.38E-01	no	-1.28E+00	8.50E-04	down
ENSMUSG00000028656	Cap1	-3.71E+00	7.01E-14	down	-4.55E+00	1.51E-31	down
ENSMUSG00000028917	Plekhm2	-3.03E+00	9.12E-11	down	-3.03E+00	1.47E-13	down
ENSMUSG00000063524	Eno1	-2.19E+00	3.27E-04	down	-2.32E+00	1.21E-04	down
ENSMUSG00000028811	Yars	-7.64E-01	3.70E-01	no	-1.92E+00	1.75E-04	down
ENSMUSG00000029022	Miip	-1.78E+00	8.14E-05	down	-2.09E+00	1.49E-09	down
ENSMUSG00000028337	Coro2a	-4.30E+00	1.50E-25	down	-1.59E+00	6.78E-02	no
ENSMUSG00000059939	9430015G10Rik	-8.23E-01	2.92E-01	no	-3.77E+00	7.22E-27	down
ENSMUSG00000028212	Ccne2	7.04E-01	3.70E-01	no	-2.49E+00	1.69E-09	down
ENSMUSG00000028789	Adc	-5.12E+00	1.76E-22	down	-3.07E+00	1.17E-04	down
ENSMUSG00000037692	Ahdc1	-3.61E+00	8.00E-10	down	-3.32E+00	6.54E-10	down
ENSMUSG00000029001	Fbxo44	-4.35E+00	1.86E-19	down	-2.27E+00	1.15E-03	no
ENSMUSG00000028964	Park7	1.76E+00	3.48E-17	up	7.13E-01	2.88E-05	no
ENSMUSG00000028248	Sfrs18	2.10E-01	8.37E-01	no	-2.17E+00	3.02E-04	down
ENSMUSG00000073758	Sh3d21	-3.87E+00	6.68E-191	down	-1.32E+00	6.44E-09	down
ENSMUSG00000041459	Tardbp	-2.67E-01	7.07E-01	no	-2.34E+00	4.65E-12	down
ENSMUSG00000042233	2010015L04Rik	-2.85E+00	1.16E-04	down	-2.88E+00	6.91E-08	down
ENSMUSG00000028391	Wdr31	-2.05E+00	3.13E-14	down	-1.19E+00	1.53E-05	down
ENSMUSG00000028292	Rars2	-2.08E-01	7.96E-01	no	-1.65E+00	2.64E-04	down
ENSMUSG00000052407	Ccdc171	-3.38E+00	1.05E-12	down	-2.81E+00	2.70E-08	down
ENSMUSG00000040720	1110037F02Rik	-1.89E+00	3.78E-02	no	-2.62E+00	2.83E-04	down
ENSMUSG00000028453	Fancg	-1.60E+00	1.24E-02	no	-3.15E+00	7.28E-19	down
ENSMUSG00000006215	Zbtb17	-1.24E+00	1.16E-01	no	-2.26E+00	5.78E-04	down
ENSMUSG00000038070	Cntln	-1.24E+00	8.34E-02	no	-2.07E+00	2.54E-05	down
ENSMUSG00000028923	Necap2	-8.83E-01	2.03E-01	no	-2.10E+00	6.29E-06	down
ENSMUSG00000040359	Ufl1	2.18E-01	7.96E-01	no	-1.63E+00	2.65E-05	down
ENSMUSG00000036002	Fam214b	-4.06E+00	3.31E-05	down	-2.59E+00	1.42E-01	no
ENSMUSG00000028718	Stil	-1.68E+00	6.04E-02	no	-2.56E+00	1.58E-04	down
ENSMUSG00000028693	Nasp	-2.05E+00	1.04E-65	down	-2.28E+00	1.07E-102	down
ENSMUSG00000028555	Ttc39a	-3.99E+00	5.54E-37	down	-5.87E-01	4.49E-01	no
ENSMUSG00000028603	Scp2	-1.93E+00	2.08E-04	down	-1.12E-01	9.51E-01	no
ENSMUSG00000028678	Kif2c	-4.06E+00	5.19E-38	down	-2.99E+00	2.30E-15	down
ENSMUSG00000066037	Hnrnpr	-1.26E+00	4.46E-02	no	-2.57E+00	2.55E-07	down
ENSMUSG00000043962	Thrap3	-9.05E-01	2.68E-01	no	-2.64E+00	4.00E-06	down
ENSMUSG00000028677	Rnf220	-1.15E+00	6.63E-05	down	-3.21E-01	5.04E-01	no
ENSMUSG00000078490	C030017K20Rik	-3.22E+00	1.10E-10	down	-3.44E+00	3.26E-20	down
ENSMUSG00000003038	Hmgn2	2.55E+00	2.15E-04	up	-1.63E-01	9.15E-01	no
ENSMUSG00000028745	Capzb	-2.20E+00	4.32E-23	down	-1.89E+00	9.06E-15	down
ENSMUSG00000028991	Mtor	-3.44E+00	1.98E-13	down	-3.34E+00	4.46E-12	down
ENSMUSG00000073888	Ccl27a	9.82E-01	2.02E-04	no	1.01E+00	1.33E-06	up
ENSMUSG00000078716	Tmem8b	-2.67E+00	7.77E-07	down	-1.70E+00	7.28E-03	no
ENSMUSG00000034171	Faah	-7.91E-01	4.04E-01	no	-2.12E+00	1.59E-04	down
ENSMUSG00000029064	Gnb1	-3.96E-01	2.72E-01	no	-1.59E+00	2.48E-08	down
ENSMUSG00000029060	Mib2	-1.33E+00	2.62E-01	no	-2.62E+00	9.50E-06	down
ENSMUSG00000028467	Gba2	-2.39E+00	5.71E-10	down	-2.00E+00	3.52E-08	down
ENSMUSG00000061298	Agbl4	-2.42E+00	2.31E-04	down	-2.02E+00	1.72E-03	no
ENSMUSG00000028637	Ccdc30	-2.35E+00	2.20E-37	down	-2.03E+00	1.24E-30	down
ENSMUSG00000054405	Dnajc8	3.46E-01	4.15E-01	no	-1.06E+00	8.94E-05	down
ENSMUSG00000042446	Zmym4	-3.17E+00	4.49E-09	down	-2.64E+00	2.37E-04	down
ENSMUSG00000040928	S100pbp	-1.05E+00	3.96E-01	no	-3.01E+00	5.92E-04	down
ENSMUSG00000035126	Wdr78	-3.82E+00	5.89E-12	down	-3.56E+00	9.21E-13	down
ENSMUSG00000012126	Ubxn11	-2.14E+00	1.33E-20	down	-1.61E+00	6.61E-15	down
ENSMUSG00000035969	Rusc2	-2.48E+00	1.27E-02	no	-3.73E+00	3.66E-05	down
ENSMUSG00000047502	Mroh7	-5.99E+00	2.91E-38	down	-3.12E+00	4.40E-04	down
ENSMUSG00000028689	Ccdc163	-1.82E+00	9.61E-07	down	-2.26E+00	1.10E-14	down
ENSMUSG00000029020	Mfn2	-3.70E+00	2.07E-33	down	-3.28E+00	2.88E-26	down
ENSMUSG00000028433	Ubap2	-2.94E+00	1.16E-28	down	-2.63E+00	8.65E-22	down
ENSMUSG00000028582	Cc2d1b	-2.47E+00	1.90E-03	no	-2.44E+00	3.16E-04	down
ENSMUSG00000036940	Kdm1a	-2.30E+00	5.96E-12	down	-2.29E+00	1.80E-12	down
ENSMUSG00000029071	Dvl1	-2.35E+00	1.13E-07	down	-3.36E+00	5.24E-32	down
ENSMUSG00000039298	Cdk5rap2	-3.81E+00	5.73E-16	down	-3.24E+00	1.31E-12	down

ENSMUSG00000028608	0610037L13Rik	-3.73E-01	5.58E-01	no	-1.25E+00	8.41E-04	down
ENSMUSG00000035539	E230008N13Rik	-2.74E+00	4.70E-10	down	-2.18E+00	1.21E-07	down
ENSMUSG00000034401	Spata6	-2.81E+00	5.04E-85	down	-1.59E+00	8.73E-17	down
ENSMUSG00000028797	Tmem234	2.76E+00	3.00E-08	up	5.36E-01	1.82E-01	no
ENSMUSG00000028572	Hook1	-4.37E+00	1.08E-44	down	-3.39E+00	3.31E-15	down
ENSMUSG00000028830	AU040320	-2.02E+00	2.08E-05	down	-2.07E+00	1.73E-05	down
ENSMUSG00000028457	Atp8b5	-2.32E+00	9.01E-04	down	-3.20E+00	7.59E-12	down
ENSMUSG00000040044	Orc3	-1.75E+00	9.96E-02	no	-3.17E+00	1.29E-06	down
ENSMUSG00000051435	Fhad1	-3.56E+00	1.81E-42	down	-1.38E+00	1.73E-04	down
ENSMUSG00000000085	Scmh1	-3.38E+00	2.88E-09	down	-3.29E+00	5.16E-09	down
ENSMUSG00000035696	Rnf38	-3.61E+00	1.55E-28	down	-2.51E+00	1.13E-09	down
ENSMUSG00000049488	Tmem67	-2.06E+00	3.71E-02	no	-2.78E+00	7.01E-04	down
ENSMUSG00000028580	Pum1	-1.64E+00	4.62E-04	down	-1.78E+00	2.24E-05	down
ENSMUSG00000058183	Mmel1	-4.74E+00	1.83E-62	down	-1.89E+00	4.03E-05	down
ENSMUSG00000017264	Exosc10	-2.23E+00	2.02E-10	down	-2.53E+00	6.07E-16	down
ENSMUSG00000028809	Srrm1	1.11E-01	8.84E-01	no	-1.88E+00	1.85E-06	down
ENSMUSG00000028931	Kcnab2	-3.50E+00	3.47E-04	down	-3.11E+00	5.75E-02	no
ENSMUSG00000028552	Eps15	-2.68E+00	5.68E-08	down	-3.61E+00	3.83E-17	down
ENSMUSG00000003644	Rps6ka1	-1.97E+00	5.72E-04	down	3.91E-01	7.69E-01	no
ENSMUSG00000028245	Nsmaf	-2.55E+00	4.99E-04	down	-2.60E+00	1.39E-03	no
ENSMUSG00000005045	Chd5	-4.19E+00	2.54E-38	down	-1.26E+00	7.95E-02	no
ENSMUSG00000053510	Nrd1	-4.44E+00	2.44E-90	down	-3.14E+00	7.56E-34	down
ENSMUSG00000028700	Pomgnt1	-2.44E+00	1.87E-15	down	-1.46E+00	2.43E-05	down
ENSMUSG00000028886	Eya3	-2.59E+00	3.99E-05	down	-1.79E+00	8.52E-03	no
ENSMUSG00000003810	Mast2	-2.25E+00	2.97E-08	down	-1.93E+00	2.21E-06	down
ENSMUSG00000028556	Dock7	-7.67E-01	5.74E-01	no	-2.27E+00	5.73E-04	down
ENSMUSG00000028760	Eif4g3	-3.95E+00	3.23E-34	down	-3.29E+00	1.27E-24	down
ENSMUSG00000039936	Pik3cd	-4.85E+00	2.56E-08	down	-4.68E+00	1.40E-07	down
ENSMUSG00000028559	Osbpl9	-3.33E+00	1.34E-11	down	-2.44E+00	4.25E-04	down
ENSMUSG00000066036	Ubr4	-3.22E+00	7.31E-18	down	-2.08E+00	2.36E-06	down
ENSMUSG00000040690	Col16a1	-3.21E+00	4.73E-12	down	-3.52E+00	3.10E-21	down
ENSMUSG00000094226	Gm21759	-4.11E+00	2.21E-05	down	-1.19E+00	6.80E-01	no
ENSMUSG00000062038	Gm10108	4.84E+00	4.43E-30	up	3.47E+00	9.96E-13	up
ENSMUSG00000067719	Gm10221	1.43E+00	1.40E-68	up	6.66E-01	4.83E-16	no
ENSMUSG00000038044	Cct8l1	-5.42E+00	1.66E-42	down	-3.17E+00	4.50E-05	down
ENSMUSG00000050677	Ccdc96	-3.08E+00	4.94E-60	down	-2.37E+00	1.42E-35	down
ENSMUSG00000094696	Gm6632	-2.27E+00	1.47E-02	no	-2.27E+00	9.50E-05	down
ENSMUSG00000089992	G6pd2	-4.06E+00	2.93E-20	down	-1.58E+00	2.14E-01	no
ENSMUSG00000070697	Utp3	4.54E-03	9.93E-01	no	-1.05E+00	9.02E-04	down
ENSMUSG00000050553	Gk2	-3.18E+00	3.88E-305	down	-2.61E+00	5.29E-208	down
ENSMUSG00000063447	Ube2d2b	-1.38E+00	2.40E-70	down	-9.46E-01	4.67E-33	no
ENSMUSG00000051503	Gm6583	-2.89E+00	1.54E-03	no	-2.55E+00	8.72E-04	down
ENSMUSG00000072722	Gm6588	-2.99E+00	1.48E-16	down	5.32E-01	5.54E-01	no
ENSMUSG00000078186	Gm13821	#VALUE!	NA	no	-6.02E+00	4.59E-04	down
ENSMUSG00000062438	Adam1b	-5.90E+00	3.75E-40	down	-5.57E+00	2.04E-34	down
ENSMUSG00000072647	Adam1a	-6.65E+00	3.01E-37	down	-3.68E+00	2.76E-04	down
ENSMUSG00000072612	Gm10382	-1.74E+00	1.26E-10	down	-1.54E+00	2.10E-07	down
ENSMUSG00000091970	Gm4868	-2.02E+00	1.29E-21	down	2.39E-01	6.91E-01	no
ENSMUSG00000070473	Cldn3	-4.34E+00	5.69E-11	down	-2.39E+00	5.05E-03	no
ENSMUSG00000074817	Papolb	-4.06E+00	3.33E-182	down	-3.43E+00	7.93E-100	down
ENSMUSG00000033794	Lpcat2b	-5.07E+00	3.33E-90	down	-2.20E+00	1.55E-06	down
ENSMUSG00000092279	1500011B03Rik	6.25E+00	1.15E-13	up	1.87E+00	8.56E-05	up
ENSMUSG00000092486	2610524H06Rik	4.12E+00	1.77E-08	up	6.07E-01	4.11E-01	no
ENSMUSG00000048988	Elfn1	-5.64E+00	3.05E-13	down	-3.91E+00	3.58E-05	down
ENSMUSG00000045435	Tmem60	-1.31E+00	1.15E-11	down	-1.31E+00	2.26E-13	down
ENSMUSG00000029138	4930548H24Rik	-3.38E+00	1.66E-05	down	-1.39E+00	4.38E-01	no
ENSMUSG00000055302	Mrfap1	-4.55E-01	4.07E-02	no	-1.51E+00	2.11E-24	down
ENSMUSG00000007907	Cabs1	-5.09E+00	1.24E-244	down	-3.50E+00	5.45E-37	down
ENSMUSG00000046000	Naa11	-1.57E+00	7.84E-02	no	-2.96E+00	5.28E-09	down
ENSMUSG00000067101	1700010H22Rik	-3.50E+00	5.68E-25	down	-8.19E-01	1.97E-01	no
ENSMUSG00000029536	Gatc	-1.06E+00	1.20E-01	no	-1.93E+00	2.74E-04	down
ENSMUSG00000029437	Il31	-4.26E-01	3.25E-02	no	2.19E+00	8.19E-14	up
ENSMUSG00000040576	Wbscr28	-2.66E+00	3.21E-22	down	-8.84E-01	1.08E-01	no
ENSMUSG00000029715	Pop7	3.14E+00	7.17E-06	up	1.46E+00	7.73E-03	no

ENSMUSG00000028998	Tomm7	2.01E+00	5.31E-116	up	1.47E+00	5.48E-94	up
ENSMUSG00000029373	Pf4	6.26E+00	7.30E-23	up	8.80E+00	3.90E-04	up
ENSMUSG00000029384	2010109A12Rik	-1.07E+00	1.51E-06	down	-9.47E-01	9.36E-06	no
ENSMUSG00000066278	Vps37b	-1.75E+00	1.72E-04	down	-7.07E-01	5.45E-01	no
ENSMUSG00000029044	4930584F24Rik	-2.78E+00	1.16E-55	down	-5.70E-01	1.33E-01	no
ENSMUSG00000029196	Tada2b	-4.03E+00	4.53E-14	down	-3.33E+00	3.96E-09	down
ENSMUSG00000029380	Cxcl1	4.54E+00	3.19E-14	up	3.20E+00	2.92E-08	up
ENSMUSG00000058427	Cxcl2	6.08E+00	3.49E-58	up	6.24E+00	6.32E-13	up
ENSMUSG00000070639	Lrrc8b	-5.28E+00	4.39E-116	down	-2.35E+00	7.28E-10	down
ENSMUSG00000029265	Dr1	-2.20E+00	4.85E-10	down	-3.68E+00	7.36E-49	down
ENSMUSG00000091933	Gm8857	-1.82E+00	2.05E-04	down	-1.54E+00	1.03E-03	no
ENSMUSG00000041697	Cox6a1	4.20E+00	0.00E+00	up	3.61E+00	0.00E+00	up
ENSMUSG00000066735	Vkorc1l1	-2.30E+00	2.08E-01	no	-3.64E+00	4.89E-04	down
ENSMUSG00000004951	Hspb1	5.97E+00	6.99E-07	up	3.21E+00	6.01E-06	up
ENSMUSG00000096798	Gm6309	-1.95E+00	2.86E-05	down	1.67E-01	9.28E-01	no
ENSMUSG00000096527	Gm6370	-2.18E+00	2.46E-06	down	2.12E-01	8.97E-01	no
ENSMUSG00000079091	Gm3404	-2.31E+00	5.26E-05	down	1.91E-01	9.29E-01	no
ENSMUSG00000029642	Polr1d	-8.52E-01	3.02E-04	no	-1.50E+00	1.34E-16	down
ENSMUSG00000059645	Gm7361	-2.27E+00	1.87E-04	down	5.47E-01	7.75E-01	no
ENSMUSG00000038803	Ost4	-1.01E+00	9.73E-03	no	-1.12E+00	8.36E-04	down
ENSMUSG00000079555	Haus3	-1.08E+00	1.20E-01	no	-2.97E+00	3.48E-12	down
ENSMUSG00000050010	Shisa3	-2.95E+00	3.20E-04	down	-2.88E+00	3.01E-05	down
ENSMUSG00000079659	Tmem243	-2.48E+00	6.86E-24	down	-5.56E-01	3.52E-01	no
ENSMUSG00000029474	Rnf34	-1.27E+00	2.82E-06	down	-1.29E+00	5.88E-06	down
ENSMUSG00000029402	Snrnp35	-1.97E+00	4.68E-07	down	-2.71E+00	9.12E-28	down
ENSMUSG00000008348	Ubc	4.62E+00	0.00E+00	up	4.91E+00	0.00E+00	up
ENSMUSG00000029526	1700123K08Rik	-2.72E+00	2.08E-09	down	-9.45E-01	2.88E-01	no
ENSMUSG00000029649	Pomp	2.32E+00	4.75E-32	up	-3.76E-02	8.67E-01	no
ENSMUSG00000053856	Dnajc5g	-1.92E+00	5.17E-43	down	-1.35E+00	1.58E-27	down
ENSMUSG00000037913	Tmem156	-3.22E+00	1.02E-21	down	-4.97E-01	5.16E-01	no
ENSMUSG00000029156	Sgcb	-2.28E+00	1.78E-05	down	-2.24E+00	3.21E-05	down
ENSMUSG00000057816	1700007G11Rik	-2.43E+00	3.06E-77	down	-2.04E+00	1.10E-79	down
ENSMUSG00000094282	Ccdc42b	-3.46E+00	2.17E-11	down	-2.33E+00	6.45E-08	down
ENSMUSG00000070493	Chchd2	3.37E+00	1.75E-43	up	1.36E+00	1.19E-12	up
ENSMUSG00000040731	Eif4h	-1.21E+00	2.64E-07	down	-1.79E+00	3.27E-20	down
ENSMUSG00000050552	Lamtor4	2.94E+00	5.18E-05	up	7.54E-01	3.47E-01	no
ENSMUSG00000039533	Mmd2	4.78E+00	8.55E-06	up	1.69E+00	1.59E-01	no
ENSMUSG00000029660	Tex26	-3.73E+00	4.58E-37	down	-1.42E+00	9.75E-03	no
ENSMUSG00000053194	Cib4	-2.70E+00	4.75E-19	down	-6.59E-01	3.15E-01	no
ENSMUSG00000029128	Rab28	-1.25E+00	1.94E-04	down	-2.01E+00	1.09E-13	down
ENSMUSG00000040514	4921511H03Rik	-2.89E+00	1.74E-24	down	-1.36E+00	5.84E-04	down
ENSMUSG00000063820	Arl9	-5.13E+00	3.72E-32	down	-3.75E+00	6.62E-12	down
ENSMUSG00000033467	Crlf2	-3.19E-01	5.77E-01	no	-2.30E+00	2.17E-08	down
ENSMUSG00000095829	Speer1-ps1	9.98E-02	8.96E-01	no	-1.33E+00	1.72E-06	down
ENSMUSG00000043388	Tmem130	-3.20E+00	4.89E-04	down	-3.82E+00	8.53E-06	down
ENSMUSG00000040367	Lrrd1	-4.93E+00	2.86E-16	down	-2.71E+00	3.61E-02	no
ENSMUSG00000042184	1700069L16Rik	-8.16E-01	4.77E-01	no	-2.08E+00	1.90E-04	down
ENSMUSG00000040013	Fkbp6	3.53E+00	7.71E-51	up	1.71E+00	8.37E-20	up
ENSMUSG00000029725	Ppp1r35	1.29E+00	1.02E-04	up	-5.21E-01	7.89E-03	no
ENSMUSG00000079562	Maea	-1.28E+00	9.07E-06	down	-1.68E+00	9.88E-11	down
ENSMUSG00000029089	Pacrgl	-1.73E+00	4.99E-12	down	-8.55E-01	2.69E-03	no
ENSMUSG00000034855	Cxcl10	5.04E+00	2.06E-08	up	3.08E+00	9.01E-03	no
ENSMUSG00000050856	Atp5k	4.06E+00	0.00E+00	up	2.98E+00	0.00E+00	up
ENSMUSG00000029601	Iqcd	-2.68E+00	1.29E-32	down	-2.01E+00	2.32E-22	down
ENSMUSG00000001168	Oas1h	8.31E+00	2.88E-05	up	8.72E+00	4.35E-04	up
ENSMUSG00000034110	Kctd7	-7.80E-01	4.38E-01	no	-2.39E+00	1.76E-09	down
ENSMUSG00000039771	Polr2j	2.98E+00	1.80E-19	up	1.34E+00	8.38E-06	up
ENSMUSG00000029726	Mepce	-2.91E+00	3.48E-19	down	-3.51E+00	1.62E-37	down
ENSMUSG00000029607	Ankrd61	-2.67E+00	1.93E-54	down	3.13E-01	4.91E-01	no
ENSMUSG00000044968	Napepld	-1.85E+00	1.14E-03	no	-2.17E+00	3.01E-06	down
ENSMUSG00000029182	1700001C02Rik	-2.94E+00	1.59E-45	down	-1.21E+00	9.19E-04	down
ENSMUSG00000029147	Ppm1g	-2.35E+00	2.86E-150	down	-1.61E+00	2.89E-64	down
ENSMUSG00000019295	Tmem129	7.03E-01	2.14E-01	no	-1.31E+00	7.85E-05	down
ENSMUSG00000032867	Fbxw8	-6.47E-01	5.04E-01	no	-2.32E+00	6.77E-04	down

ENSMUSG00000023104	Rfc2	-1.81E+00	4.77E-43	down	-1.47E+00	1.36E-32	down
ENSMUSG00000029560	Snx8	3.46E+00	6.42E-04	up	2.57E+00	3.19E-02	no
ENSMUSG00000029624	Ptcd1	-2.18E+00	3.92E-03	no	-3.15E+00	4.39E-13	down
ENSMUSG00000007415	Gatad1	-2.23E+00	1.44E-10	down	-2.54E+00	8.12E-19	down
ENSMUSG00000029235	Pdcl2	-2.12E+00	1.01E-266	down	-2.25E+00	0.00E+00	down
ENSMUSG00000036256	Igfbp7	5.65E+00	4.50E-96	up	3.06E+00	9.89E-12	up
ENSMUSG00000002240	4930432K09Rik	-2.76E+00	1.02E-74	down	4.28E-01	2.20E-01	no
ENSMUSG00000009013	Dynll1	2.42E+00	8.19E-192	up	2.04E+00	6.13E-113	up
ENSMUSG00000029518	Rab35	-1.80E+00	6.17E-02	no	-2.77E+00	3.71E-04	down
ENSMUSG00000029616	Erp29	-1.10E+00	7.22E-03	no	-1.70E+00	6.88E-07	down
ENSMUSG00000038582	Pptc7	-1.76E+00	3.57E-04	down	-3.34E-01	7.57E-01	no
ENSMUSG00000025532	Crcp	-1.05E+00	5.80E-05	down	-1.22E+00	1.87E-11	down
ENSMUSG00000001687	Ubl3	-2.58E+00	7.39E-28	down	-1.92E+00	4.62E-08	down
ENSMUSG00000029326	Enoph1	-1.01E+00	6.37E-02	no	-2.03E+00	3.30E-06	down
ENSMUSG00000008090	Fgfrl1	-4.56E-01	6.23E-01	no	-2.27E+00	3.53E-04	down
ENSMUSG00000037979	Ccdc92	-1.53E+00	3.90E-39	down	-8.33E-01	2.70E-17	no
ENSMUSG00000025337	Sbds	-1.71E+00	2.40E-12	down	-2.42E+00	1.45E-42	down
ENSMUSG00000029291	Rufy3	-3.44E+00	1.90E-08	down	-1.77E+00	1.41E-01	no
ENSMUSG00000042190	Cmklr1	-4.27E+00	3.77E-11	down	-2.13E+00	6.08E-02	no
ENSMUSG00000048578	Mlec	-2.53E+00	3.96E-15	down	-2.68E+00	2.98E-18	down
ENSMUSG00000029427	Zcchc8	-3.36E+00	1.05E-22	down	-1.42E+00	2.29E-02	no
ENSMUSG00000053094	Tmem248	-1.29E+00	9.81E-06	down	-2.90E-01	5.52E-01	no
ENSMUSG00000038970	Lmtk2	-2.83E+00	3.07E-02	no	-4.05E+00	1.02E-05	down
ENSMUSG00000028953	Abcf2	-2.69E+00	1.78E-54	down	-1.86E+00	9.47E-24	down
ENSMUSG00000029111	Nelfa	-2.45E-01	7.92E-01	no	-2.00E+00	4.66E-05	down
ENSMUSG00000046985	Tapt1	-2.21E+00	1.46E-03	no	-3.00E+00	9.26E-07	down
ENSMUSG00000029169	Dhx15	-1.72E+00	2.97E-08	down	-1.96E+00	3.90E-11	down
ENSMUSG00000029447	Cct6a	-4.84E-01	5.37E-02	no	-1.27E+00	7.02E-11	down
ENSMUSG00000039754	Alkbh4	-1.36E+00	8.70E-02	no	-2.78E+00	8.77E-09	down
ENSMUSG00000029617	Ccz1	-1.95E+00	1.93E-13	down	-2.35E+00	5.43E-23	down
ENSMUSG00000028932	Psmc2	-1.55E+00	8.61E-20	down	-8.46E-01	6.93E-06	no
ENSMUSG00000062604	Srpk2	-2.69E+00	2.51E-22	down	-1.42E+00	3.18E-05	down
ENSMUSG00000028954	Nub1	-2.88E+00	2.85E-18	down	-2.41E+00	9.91E-11	down
ENSMUSG00000040274	Cdk6	2.47E+00	1.16E-04	up	1.80E+00	4.17E-04	up
ENSMUSG00000005103	Wdr1	-2.20E+00	4.05E-13	down	-4.30E-01	5.67E-01	no
ENSMUSG00000029368	Alb	1.63E+00	4.92E-06	up	2.66E-01	7.20E-01	no
ENSMUSG00000032959	Pebp1	1.17E+00	1.65E-107	up	1.01E+00	1.96E-112	up
ENSMUSG00000034118	Tpst1	-1.78E+00	1.49E-14	down	-1.42E+00	1.45E-07	down
ENSMUSG00000029586	4933411G11Rik	-1.56E+00	3.60E-02	no	-2.58E+00	2.15E-14	down
ENSMUSG00000029404	Arl6ip4	3.32E-01	4.12E-01	no	-1.32E+00	2.82E-09	down
ENSMUSG00000079215	Zfp664	-1.21E+00	9.41E-02	no	-3.36E+00	4.74E-12	down
ENSMUSG00000029681	Bcl7b	-2.80E-02	9.66E-01	no	-1.90E+00	3.04E-10	down
ENSMUSG00000025857	Heatr2	-1.17E+00	2.76E-02	no	-1.75E+00	2.29E-05	down
ENSMUSG00000045078	Rnf216	-2.78E+00	2.39E-18	down	-1.79E+00	4.12E-05	down
ENSMUSG00000047221	Fam185a	-2.87E+00	1.30E-04	down	-7.31E-01	6.61E-01	no
ENSMUSG00000048439	Nupl2	-2.51E+00	1.61E-12	down	-1.09E+00	3.68E-02	no
ENSMUSG00000073102	Drc1	-3.37E+00	7.24E-42	down	-2.85E+00	1.33E-37	down
ENSMUSG00000067365	Tmem128	4.70E-01	3.10E-01	no	-1.04E+00	3.80E-04	down
ENSMUSG00000029338	Antxr2	-3.72E+00	7.66E-04	down	-3.55E+00	1.11E-01	no
ENSMUSG00000029439	Sfswap	-7.72E-01	2.93E-01	no	-2.36E+00	2.38E-06	down
ENSMUSG00000038690	Atp5j2	1.86E+00	5.16E-50	up	8.42E-01	1.26E-20	no
ENSMUSG00000028938	Galntl5	-3.74E+00	5.62E-189	down	-1.30E+00	2.41E-08	down
ENSMUSG00000008307	1700109H08Rik	-2.60E+00	3.16E-57	down	-4.44E-01	1.49E-01	no
ENSMUSG00000029223	Uchl1	2.01E+00	2.65E-05	up	1.98E+00	9.26E-06	up
ENSMUSG00000029330	Cds1	-2.27E+00	1.25E-18	down	-1.98E+00	1.25E-17	down
ENSMUSG00000033805	Ephx4	-3.06E+00	1.31E-04	down	-2.11E+00	1.17E-01	no
ENSMUSG00000029345	Tfip11	-1.99E+00	3.44E-05	down	-1.74E+00	4.40E-04	down
ENSMUSG00000018974	Sart3	-2.12E-01	8.15E-01	no	-2.07E+00	9.16E-04	down
ENSMUSG00000029524	Sirt4	-2.83E+00	1.51E-06	down	-1.86E+00	1.01E-02	no
ENSMUSG00000056076	Eif3b	-2.84E+00	2.85E-107	down	-2.32E+00	3.06E-70	down
ENSMUSG00000038199	4931409K22Rik	-3.48E+00	5.48E-48	down	8.38E-02	9.11E-01	no
ENSMUSG00000029175	Slc35f6	-1.18E+00	2.89E-01	no	-2.51E+00	1.22E-05	down
ENSMUSG00000070858	Gm1673	4.96E+00	1.30E-146	up	4.36E+00	1.06E-220	up
ENSMUSG00000079666	1700015F17Rik	-2.05E+00	1.48E-48	down	-2.33E-01	5.00E-01	no

ENSMUSG00000029253	Cenpc1	-1.28E+00	1.07E-01	no	-3.30E+00	1.18E-11	down
ENSMUSG00000016833	Mrps18c	1.98E+00	5.41E-14	up	1.62E-01	4.82E-01	no
ENSMUSG00000043913	Ccdc60	-3.22E+00	2.43E-88	down	-2.67E+00	4.98E-63	down
ENSMUSG00000029599	Ddx54	-2.68E+00	1.93E-11	down	-2.40E+00	2.32E-09	down
ENSMUSG00000037364	Srrt	-1.25E+00	2.39E-05	down	-2.99E+00	1.26E-59	down
ENSMUSG00000001467	Cyp51	-3.15E+00	5.18E-14	down	-7.85E-01	3.97E-01	no
ENSMUSG00000063015	Ccni	-2.91E+00	4.57E-30	down	-3.40E+00	5.57E-49	down
ENSMUSG00000042249	Adrbk2	-3.49E+00	7.83E-07	down	-2.87E+00	6.28E-06	down
ENSMUSG00000029550	Sppl3	-8.71E-01	8.07E-02	no	-1.65E+00	1.32E-05	down
ENSMUSG00000029640	Usp12	-5.52E+00	3.23E-14	down	-3.89E+00	3.70E-04	down
ENSMUSG00000029655	N4bp2l2	-2.34E+00	1.07E-06	down	-2.01E+00	1.05E-03	no
ENSMUSG00000002221	Paxip1	-2.59E+00	5.34E-06	down	-2.31E+00	2.70E-03	no
ENSMUSG00000029096	Htra3	4.54E+00	8.07E-11	up	2.18E+00	2.45E-01	no
ENSMUSG00000039178	Tbc1d19	-2.53E+00	4.01E-06	down	-2.09E+00	3.25E-03	no
ENSMUSG00000029423	Piwil1	-1.79E+00	7.68E-10	down	-3.43E+00	9.44E-131	down
ENSMUSG00000019179	Mdh2	1.60E+00	4.08E-07	up	1.24E+00	5.89E-04	up
ENSMUSG00000039737	Prkrip1	-7.24E-01	2.50E-01	no	-1.60E+00	1.85E-04	down
ENSMUSG00000001847	Rac1	2.71E+00	8.55E-07	up	-3.88E-01	5.80E-01	no
ENSMUSG00000016510	Mtif3	-2.09E+00	7.23E-02	no	-3.11E+00	6.39E-06	down
ENSMUSG00000014956	Ppp1cb	-1.80E-01	8.56E-01	no	-2.55E+00	9.11E-07	down
ENSMUSG00000036693	Nop14	-2.77E+00	5.06E-31	down	-1.98E+00	4.26E-11	down
ENSMUSG00000029415	Sdad1	-1.11E+00	1.96E-03	no	-1.90E+00	6.97E-08	down
ENSMUSG00000029503	P2rx2	-3.88E+00	4.13E-05	down	-1.20E+00	3.83E-01	no
ENSMUSG00000060152	Pop5	2.69E+00	4.44E-04	up	1.47E+00	3.10E-02	no
ENSMUSG00000005374	Tbl2	-1.88E+00	8.23E-06	down	-2.34E+00	4.19E-13	down
ENSMUSG00000037390	Muc3	-2.02E+00	1.15E-06	down	-2.11E+00	6.80E-12	down
ENSMUSG00000039000	Ube3c	-3.02E+00	1.20E-10	down	-3.24E+00	3.22E-12	down
ENSMUSG00000043059	Zfp513	-2.25E+00	4.55E-05	down	-2.10E+00	4.45E-03	no
ENSMUSG00000029248	1700023E05Rik	-3.95E+00	1.01E-50	down	-2.34E+00	8.58E-13	down
ENSMUSG00000029477	Morn3	-2.94E+00	2.88E-98	down	-6.87E-01	5.74E-03	no
ENSMUSG00000063409	Lrrc43	-3.51E+00	4.46E-45	down	-2.31E+00	2.59E-17	down
ENSMUSG00000029613	Eif2ak1	-1.57E+00	5.73E-02	no	-2.30E+00	3.56E-04	down
ENSMUSG00000036087	Slain2	-3.36E+00	3.33E-22	down	-3.05E+00	1.57E-17	down
ENSMUSG00000029152	Ociad1	-1.35E+00	4.88E-05	down	-1.53E+00	1.17E-06	down
ENSMUSG00000066900	Suds3	-3.01E+00	1.82E-29	down	-2.73E+00	7.17E-26	down
ENSMUSG00000064267	Hvcn1	-2.48E+00	4.79E-02	no	-4.84E+00	9.32E-05	down
ENSMUSG00000029388	Eif2b1	2.81E-01	7.41E-01	no	-1.69E+00	2.58E-06	down
ENSMUSG00000040751	Lat2	-7.80E-01	5.87E-01	no	-4.44E+00	2.82E-13	down
ENSMUSG00000014932	Yes1	-1.11E+00	4.33E-01	no	-3.44E+00	2.46E-05	down
ENSMUSG00000029186	Pi4k2b	-3.62E+00	2.04E-23	down	-2.41E+00	6.79E-06	down
ENSMUSG00000042002	Foxn4	6.27E+00	8.27E-05	up	6.26E+00	1.29E-06	up
ENSMUSG00000053293	Pom121	-3.04E+00	5.68E-12	down	-2.91E+00	4.06E-12	down
ENSMUSG00000029166	Mapre3	-3.50E+00	2.03E-22	down	-2.40E+00	9.08E-09	down
ENSMUSG00000029103	Lrpap1	1.31E+00	4.43E-04	up	-1.11E+00	2.14E-04	down
ENSMUSG00000029486	Mrpl1	-2.17E+00	3.92E-09	down	-2.25E+00	4.73E-12	down
ENSMUSG00000029501	Ankle2	-1.90E+00	5.88E-04	down	-2.98E+00	3.37E-13	down
ENSMUSG00000029504	Ddx51	-2.71E-01	8.14E-01	no	-2.77E+00	8.28E-08	down
ENSMUSG00000089984	Fbxo24	-7.10E+00	3.50E-44	down	-4.47E+00	9.75E-09	down
ENSMUSG00000014529	4930511M11Rik	-2.08E+00	8.80E-37	down	-1.98E+00	3.27E-52	down
ENSMUSG00000029320	1700016H13Rik	-3.72E+00	1.44E-61	down	-2.42E+00	6.10E-11	down
ENSMUSG00000055991	Zkscan5	-3.10E+00	2.21E-04	down	-3.49E+00	4.62E-08	down
ENSMUSG00000049971	Glt1d1	-5.68E+00	2.21E-17	down	-5.03E+00	4.71E-13	down
ENSMUSG00000040532	Abhd11	-1.93E+00	7.41E-05	down	-3.70E+00	1.43E-26	down
ENSMUSG00000019054	Fis1	1.38E+00	9.88E-09	up	-3.93E-01	8.95E-02	no
ENSMUSG00000029203	Ube2k	-1.74E+00	3.61E-07	down	-1.17E+00	1.52E-02	no
ENSMUSG00000023707	Ogfod2	-5.49E-01	3.43E-01	no	-1.64E+00	3.52E-06	down
ENSMUSG00000029723	Tsc22d4	-2.45E+00	8.63E-13	down	1.42E-01	8.92E-01	no
ENSMUSG00000037017	Zscan21	-2.32E+00	3.13E-26	down	-1.65E+00	6.20E-14	down
ENSMUSG00000038722	Bud31	-1.11E+00	3.47E-07	down	-1.21E+00	7.01E-09	down
ENSMUSG00000029119	Man2b2	-3.97E+00	1.20E-32	down	-3.60E+00	6.58E-29	down
ENSMUSG00000029199	Lias	-1.52E+00	2.21E-03	no	-2.04E+00	1.04E-07	down
ENSMUSG00000029578	Wipi2	-9.29E-01	7.81E-02	no	-1.77E+00	1.83E-05	down
ENSMUSG00000029151	Slc30a3	-1.59E+00	1.65E-10	down	-1.13E+00	1.01E-06	down
ENSMUSG00000029202	Pds5a	-1.78E+00	2.89E-02	no	-2.99E+00	2.06E-04	down

ENSMUSG00000029250	Polr2b	3.19E-01	7.00E-01	no	-2.16E+00	5.01E-06	down
ENSMUSG00000044060	A830010M20Rik	-1.98E+00	1.84E-01	no	-3.67E+00	6.26E-04	down
ENSMUSG00000029455	Aldh2	3.15E+00	6.03E-05	up	1.24E+00	2.17E-01	no
ENSMUSG00000037108	Zcwpw1	1.90E+00	7.93E-04	up	-8.01E-01	2.07E-01	no
ENSMUSG00000029580	Actb	1.68E+00	1.94E-111	up	1.28E+00	2.88E-24	up
ENSMUSG00000000600	Krit1	-3.33E-01	7.97E-01	no	-2.84E+00	2.25E-04	down
ENSMUSG00000029310	Nudt9	-2.01E+00	3.28E-27	down	-1.58E+00	2.59E-18	down
ENSMUSG00000058153	Sez6l	-4.54E+00	6.06E-04	down	-9.12E-01	7.95E-01	no
ENSMUSG00000029564	4930519G04Rik	-1.89E+00	2.68E-07	down	-1.15E+00	1.77E-02	no
ENSMUSG00000010721	Lmbr1	-2.39E+00	3.37E-05	down	-1.73E+00	6.64E-03	no
ENSMUSG00000029094	Afap1	-3.36E+00	9.32E-04	down	-3.92E+00	3.11E-02	no
ENSMUSG00000029279	Brdt	-2.41E+00	2.23E-26	down	-2.65E+00	6.24E-33	down
ENSMUSG00000001098	Kctd10	-2.44E+00	1.00E-04	down	-2.31E+00	3.72E-03	no
ENSMUSG00000075569	Rsph10b2	-4.31E+00	3.62E-23	down	-3.36E+00	8.30E-11	down
ENSMUSG00000042508	Dmtf1	-2.94E+00	4.12E-06	down	-2.97E+00	5.50E-06	down
ENSMUSG00000014668	Chfr	-2.32E+00	9.38E-06	down	-3.74E+00	3.15E-26	down
ENSMUSG00000043036	Ccdc63	-2.90E+00	4.97E-68	down	-1.57E-01	7.61E-01	no
ENSMUSG00000067642	Gpr113	-5.22E+00	3.32E-07	down	-1.48E+00	6.02E-01	no
ENSMUSG00000029312	Klhl8	-2.16E+00	4.10E-04	down	-2.27E+00	8.36E-06	down
ENSMUSG00000037890	Wdr19	-3.13E+00	3.97E-06	down	-3.03E+00	1.85E-06	down
ENSMUSG00000034826	Nup54	-1.00E-01	9.34E-01	no	-2.30E+00	8.71E-04	down
ENSMUSG00000064280	Ccdc146	-3.51E+00	3.49E-21	down	-2.63E+00	3.66E-12	down
ENSMUSG00000061882	Ccdc62	-2.89E+00	1.98E-26	down	-2.85E+00	4.22E-30	down
ENSMUSG00000051306	Usp42	-2.19E+00	4.47E-11	down	-2.05E+00	5.30E-10	down
ENSMUSG00000029622	Arpc1b	3.87E+00	8.63E-07	up	3.26E+00	1.11E-04	up
ENSMUSG00000052783	Grk4	-2.52E+00	4.58E-17	down	-2.42E+00	2.22E-18	down
ENSMUSG00000029408	Abcb9	-2.88E+00	4.11E-05	down	-3.02E+00	2.96E-05	down
ENSMUSG00000029131	Dnajb6	-1.75E+00	5.71E-10	down	-2.03E+00	3.31E-17	down
ENSMUSG00000029168	Dpysl5	-4.07E+00	7.55E-08	down	-1.28E+00	3.96E-01	no
ENSMUSG00000037210	Fam193a	-3.00E+00	6.25E-12	down	-2.90E+00	2.77E-10	down
ENSMUSG00000028995	Fam126a	-2.60E+00	1.45E-07	down	-2.92E+00	9.53E-13	down
ENSMUSG00000015880	Ncapg	-2.49E+00	2.79E-05	down	-3.47E+00	3.73E-10	down
ENSMUSG00000029188	Slc34a2	-4.57E+00	5.60E-14	down	-4.62E+00	1.39E-18	down
ENSMUSG00000029154	Cwh43	5.03E+00	2.33E-04	up	4.85E+00	2.76E-05	up
ENSMUSG00000044221	Grsf1	-1.27E+00	7.14E-03	no	-3.01E+00	4.45E-19	down
ENSMUSG00000015806	Qdpr	2.87E+00	3.28E-05	up	6.77E-01	3.68E-01	no
ENSMUSG00000029314	Agpat9	-4.12E+00	2.36E-10	down	-1.28E+00	3.86E-01	no
ENSMUSG00000029017	Pmpcb	-2.35E+00	3.53E-14	down	-2.46E+00	6.97E-22	down
ENSMUSG00000029247	Paics	-5.18E-01	3.73E-01	no	-1.75E+00	3.17E-06	down
ENSMUSG00000041870	Ankrd13a	-2.72E+00	2.20E-31	down	-2.54E+00	8.22E-39	down
ENSMUSG00000042594	Sh2b3	-1.95E+00	7.06E-07	down	-2.14E+00	2.66E-12	down
ENSMUSG00000002486	Tchp	-1.32E+00	6.92E-03	no	-2.44E+00	1.32E-22	down
ENSMUSG00000013622	Atraid	4.08E+00	1.54E-09	up	1.64E+00	4.50E-03	no
ENSMUSG00000004642	Slbp	1.42E+00	2.06E-07	up	-7.38E-03	9.85E-01	no
ENSMUSG00000029405	G3bp2	-6.09E-01	3.90E-01	no	-1.72E+00	1.79E-04	down
ENSMUSG00000035297	Cops4	-1.61E+00	4.38E-11	down	-1.15E+00	7.68E-07	down
ENSMUSG00000050050	Ccdc158	-3.64E+00	7.03E-20	down	-3.03E+00	9.61E-15	down
ENSMUSG00000029428	Stx2	-2.49E+00	1.12E-08	down	-1.57E+00	2.11E-03	no
ENSMUSG00000041298	Katnal1	-2.38E+00	1.26E-06	down	-2.04E+00	5.61E-06	down
ENSMUSG00000038564	Ift172	-4.06E+00	3.08E-53	down	-3.51E+00	2.30E-45	down
ENSMUSG00000042328	Hps4	-1.69E+00	1.87E-04	down	-7.96E-01	9.37E-02	no
ENSMUSG00000042726	Trafd1	-2.12E+00	8.05E-25	down	-2.15E+00	1.01E-36	down
ENSMUSG00000015942	Gtf2ird2	-3.15E+00	9.60E-03	no	-4.13E+00	9.48E-06	down
ENSMUSG00000039477	Tnrc18	-4.70E+00	1.64E-15	down	-1.83E+00	1.27E-01	no
ENSMUSG00000039782	Cpeb2	-4.13E+00	8.04E-46	down	-2.77E+00	1.60E-12	down
ENSMUSG00000029381	Shroom3	-1.91E+00	1.46E-04	down	-1.73E+00	1.62E-03	no
ENSMUSG00000029458	Brap	-3.41E+00	1.08E-30	down	-3.16E+00	1.24E-20	down
ENSMUSG00000029155	Spata18	-5.70E+00	4.38E-118	down	-3.43E+00	5.03E-18	down
ENSMUSG00000036377	C530008M17Rik	-4.65E+00	6.91E-53	down	-3.49E+00	8.85E-36	down
ENSMUSG00000029469	Ift81	-3.14E+00	4.61E-36	down	-2.34E+00	1.19E-14	down
ENSMUSG00000029386	Tctn2	-3.09E+00	2.72E-10	down	-1.92E+00	1.45E-03	no
ENSMUSG00000029206	Nsun7	-4.35E+00	1.82E-38	down	-3.50E+00	1.27E-22	down
ENSMUSG00000029625	Cpsf4	-8.36E-01	4.01E-01	no	-2.39E+00	1.75E-05	down
ENSMUSG00000029012	Orc5	-2.12E-01	7.45E-01	no	-1.44E+00	3.71E-05	down

ENSMUSG00000029141	Slc4a1ap	-1.55E+00	3.10E-02	no	-2.14E+00	6.98E-04	down
ENSMUSG00000002297	Dbf4	-1.46E+00	5.83E-03	no	-2.12E+00	6.96E-06	down
ENSMUSG00000025745	Hadha	-3.29E+00	1.33E-41	down	-2.79E+00	2.67E-29	down
ENSMUSG00000029635	Cdk8	-1.51E+00	1.04E-01	no	-2.80E+00	1.62E-04	down
ENSMUSG00000029403	Cdkl2	-2.10E+00	2.36E-08	down	-2.39E+00	8.29E-14	down
ENSMUSG00000029502	Golga3	-3.70E+00	1.72E-11	down	-3.89E+00	1.07E-13	down
ENSMUSG00000029703	Lrwd1	-2.03E+00	1.26E-17	down	-1.68E+00	6.89E-16	down
ENSMUSG00000048271	Rbm33	-2.06E+00	1.48E-02	no	-2.80E+00	2.91E-04	down
ENSMUSG00000029110	Rnf4	-1.76E+00	8.70E-07	down	-9.00E-01	1.25E-01	no
ENSMUSG00000038384	Setd1b	-4.14E+00	4.39E-03	no	-4.66E+00	6.62E-04	down
ENSMUSG00000000915	Hip1r	-2.74E+00	3.75E-04	down	-3.17E+00	4.13E-08	down
ENSMUSG00000037936	Scarb1	-2.29E+00	1.62E-04	down	-3.19E-01	8.06E-01	no
ENSMUSG00000038780	Smurf1	-1.70E+00	7.99E-04	down	-2.17E+00	1.08E-07	down
ENSMUSG00000048520	Fbxl13	-4.31E+00	3.98E-57	down	-1.49E+00	5.21E-03	no
ENSMUSG00000028973	Abcb8	1.29E-01	9.03E-01	no	-1.71E+00	7.08E-04	down
ENSMUSG00000067367	Lyar	-9.54E-01	7.66E-48	no	-1.13E+00	5.53E-113	down
ENSMUSG00000036323	Srp72	-2.39E+00	5.14E-10	down	-1.93E+00	1.36E-05	down
ENSMUSG00000029467	Atp2a2	-1.46E+00	2.33E-04	down	-2.75E+00	2.63E-16	down
ENSMUSG00000029730	Mcm7	-1.60E+00	3.31E-08	down	-1.41E+00	1.73E-06	down
ENSMUSG00000037313	Tacc3	-1.72E+00	2.10E-05	down	-3.05E+00	5.51E-23	down
ENSMUSG00000038095	Sbno1	-1.42E+00	3.10E-04	down	-2.50E+00	1.28E-16	down
ENSMUSG00000029209	Gnpda2	-2.20E+00	3.59E-05	down	5.11E-02	9.75E-01	no
ENSMUSG00000037795	N4bp2	-3.81E+00	4.85E-05	down	-3.38E+00	3.98E-04	down
ENSMUSG00000029577	Ube3b	-3.18E+00	3.74E-23	down	-2.62E+00	1.01E-17	down
ENSMUSG00000036980	Taf6	-1.70E+00	9.02E-08	down	-1.98E+00	9.48E-10	down
ENSMUSG00000000568	Hnrnpd	-2.38E+00	2.88E-04	down	-3.16E+00	2.59E-09	down
ENSMUSG00000033773	Rpap2	-2.95E+00	2.33E-05	down	-3.37E+00	1.62E-07	down
ENSMUSG00000019178	Styxl1	-2.12E+00	3.28E-16	down	-1.45E+00	2.45E-08	down
ENSMUSG00000029528	Pxn	-4.36E+00	8.27E-10	down	-4.13E+00	1.13E-06	down
ENSMUSG00000039753	Fbxl5	-1.46E+00	1.76E-02	no	-2.53E+00	3.35E-07	down
ENSMUSG00000029442	Wdr66	-3.10E+00	4.26E-10	down	-2.68E+00	5.22E-14	down
ENSMUSG00000029657	Hsph1	-2.57E+00	1.88E-04	down	-2.86E+00	9.37E-06	down
ENSMUSG00000041264	Uspl1	-2.55E+00	9.18E-05	down	-3.09E+00	4.10E-09	down
ENSMUSG00000029014	Dnajc2	-1.74E+00	2.80E-12	down	-2.04E+00	5.86E-19	down
ENSMUSG00000041740	Rnf10	-3.07E+00	7.56E-43	down	-3.30E+00	5.10E-48	down
ENSMUSG00000054256	Msi1	-4.37E+00	1.14E-03	no	-5.13E+00	1.67E-06	down
ENSMUSG00000028944	Prkag2	-2.84E+00	1.58E-15	down	-2.82E+00	2.42E-16	down
ENSMUSG00000090262	Mpv17	3.33E+00	1.09E-06	up	2.26E+00	4.02E-02	no
ENSMUSG00000040473	A330021E22Rik	-3.88E+00	1.74E-45	down	-3.22E+00	5.75E-29	down
ENSMUSG00000042719	Naa25	-2.62E+00	1.77E-05	down	-2.62E+00	2.06E-05	down
ENSMUSG00000028959	Fastk	-1.25E+00	1.18E-03	no	-1.70E+00	1.02E-08	down
ENSMUSG00000029098	Acox3	-2.23E+00	1.82E-02	no	-3.21E+00	3.07E-05	down
ENSMUSG00000038828	Tmem214	-1.60E+00	1.32E-06	down	-1.55E+00	9.93E-09	down
ENSMUSG00000051674	Dcun1d4	-3.00E+00	2.59E-18	down	-1.44E+00	4.14E-03	no
ENSMUSG00000038126	Mphosph9	-2.38E+00	7.65E-04	down	-1.75E+00	3.21E-02	no
ENSMUSG00000029276	Glmn	-2.19E+00	2.09E-05	down	-2.34E+00	1.40E-06	down
ENSMUSG00000034021	Pds5b	-1.15E+00	2.67E-01	no	-3.24E+00	1.16E-04	down
ENSMUSG00000072720	Myo18b	-4.64E+00	6.09E-06	down	-1.65E+00	4.70E-01	no
ENSMUSG00000029713	Gnb2	-3.16E+00	1.89E-19	down	-3.72E+00	2.57E-36	down
ENSMUSG00000029144	Gtf3c2	-2.02E+00	1.42E-04	down	-1.59E+00	3.35E-02	no
ENSMUSG00000042605	Atxn2	-2.33E+00	2.98E-05	down	-2.20E+00	3.87E-05	down
ENSMUSG00000029104	Htt	-2.00E+00	7.32E-02	no	-3.23E+00	6.27E-04	down
ENSMUSG00000029174	Tbc1d1	-2.12E+00	4.85E-06	down	-2.51E+00	9.52E-13	down
ENSMUSG00000035325	Sec31a	-2.12E+00	3.53E-06	down	-1.74E+00	3.59E-04	down
ENSMUSG00000046709	Mapk10	-2.83E+00	1.12E-04	down	-2.01E+00	2.39E-02	no
ENSMUSG00000036555	Iqce	-4.08E+00	5.33E-14	down	-2.70E+00	2.43E-05	down
ENSMUSG00000029191	Rfc1	-2.24E+00	5.50E-09	down	-2.19E+00	2.93E-09	down
ENSMUSG00000034842	Art3	-2.31E+00	1.23E-24	down	-2.19E+00	2.75E-32	down
ENSMUSG00000062234	Gak	-1.88E+00	1.06E-02	no	-2.32E+00	4.45E-04	down
ENSMUSG00000029406	Pitpnm2	-3.75E+00	9.62E-05	down	-1.93E+00	3.10E-01	no
ENSMUSG00000043410	Hfm1	-1.63E+00	1.00E-01	no	-3.69E+00	1.48E-07	down
ENSMUSG00000029165	Agbl5	-4.10E+00	1.78E-64	down	-3.33E+00	6.80E-48	down
ENSMUSG00000038011	Dnahc10	-4.35E+00	8.70E-08	down	-3.80E+00	6.25E-07	down
ENSMUSG00000029647	Pan3	-3.79E+00	7.57E-13	down	-3.55E+00	3.87E-09	down

ENSMUSG00000036928	Stag3	-2.71E+00	2.20E-32	down	-3.03E+00	1.19E-42	down
ENSMUSG00000057406	Whsc1	-2.64E+00	4.51E-06	down	-2.60E+00	9.37E-06	down
ENSMUSG00000029267	Mtf2	-1.64E-01	8.34E-01	no	-2.05E+00	1.20E-06	down
ENSMUSG00000041638	Gcn1l1	-2.91E+00	1.20E-05	down	-2.71E+00	1.36E-04	down
ENSMUSG00000037736	Limch1	-3.33E+00	1.44E-06	down	1.33E-02	9.95E-01	no
ENSMUSG00000029192	Tbc1d14	-3.34E+00	3.46E-08	down	-2.83E+00	7.28E-05	down
ENSMUSG00000029505	Ep400	-1.97E+00	5.77E-08	down	-2.38E+00	3.36E-15	down
ENSMUSG00000079173	Zan	-4.95E+00	3.36E-04	down	-1.86E+00	5.94E-01	no
ENSMUSG00000036817	Sun1	-2.17E+00	8.08E-06	down	-2.45E+00	7.34E-06	down
ENSMUSG00000042744	Gm15800	-3.21E+00	3.01E-06	down	-3.09E+00	1.32E-07	down
ENSMUSG00000029086	Prom1	-4.65E+00	1.50E-15	down	-3.65E+00	1.08E-07	down
ENSMUSG00000029475	Kdm2b	-2.41E+00	2.29E-02	no	-3.56E+00	2.69E-04	down
ENSMUSG00000049550	Clip1	-2.84E+00	2.45E-20	down	-5.15E-02	9.59E-01	no
ENSMUSG00000029134	Plb1	-4.99E+00	5.20E-09	down	-2.59E+00	1.25E-01	no
ENSMUSG00000029705	Cux1	-2.94E+00	5.21E-07	down	-2.42E+00	5.81E-04	down
ENSMUSG00000023079	Gtf2ird1	-2.94E+00	4.08E-03	no	-3.20E+00	1.42E-04	down
ENSMUSG00000045482	Trrap	-5.18E-01	7.16E-01	no	-2.47E+00	6.19E-04	down
ENSMUSG00000060261	Gtf2i	-3.15E+00	2.66E-19	down	-2.44E+00	8.68E-17	down
ENSMUSG00000096403	Gm9825	9.77E-01	5.82E-02	no	-1.17E+00	4.43E-04	down
ENSMUSG00000078179	Rnf148	-4.77E+00	2.04E-54	down	-2.94E+00	1.22E-07	down
ENSMUSG00000054431	Olfr450	3.55E+00	1.72E-10	up	5.48E+00	1.72E-10	up
ENSMUSG00000036463	4930544G11Rik	-3.12E+00	5.09E-159	down	-2.85E+00	5.74E-149	down
ENSMUSG00000056197	4931417E11Rik	-2.30E+00	1.59E-30	down	-1.34E+00	5.08E-16	down
ENSMUSG00000051695	Pcbp1	1.56E+00	9.21E-13	up	-8.49E-01	2.52E-14	no
ENSMUSG00000044927	H1fx	-7.26E-01	8.94E-02	no	-2.16E+00	1.38E-14	down
ENSMUSG00000048206	Dnajb8	-4.71E+00	1.53E-132	down	-2.38E+00	2.13E-14	down
ENSMUSG00000072878	1700123L14Rik	-6.05E+00	0.00E+00	down	-5.34E+00	0.00E+00	down
ENSMUSG00000037827	Gm5884	-2.63E+00	1.45E-14	down	-2.04E+00	7.37E-09	down
ENSMUSG00000047104	Pbp2	-1.17E+00	2.03E-32	down	-9.39E-01	1.39E-28	no
ENSMUSG00000053935	Atf7ip	-1.07E+00	4.40E-01	no	-3.47E+00	4.12E-07	down
ENSMUSG00000060032	H2afj	6.16E+00	2.97E-125	up	5.25E+00	9.47E-142	up
ENSMUSG00000046717	Igbp1b	-3.05E+00	3.40E-23	down	-2.15E+00	9.69E-14	down
ENSMUSG00000041791	Capza3	-3.27E+00	2.67E-125	down	-1.02E+00	1.36E-05	down
ENSMUSG00000063171	Rps4y2	1.27E+00	1.22E-47	up	9.97E-01	2.17E-36	no
ENSMUSG00000090685	Gm9047	-2.71E+00	2.96E-10	down	-6.58E-01	5.91E-01	no
ENSMUSG00000029867	1700034O15Rik	-1.59E+00	1.83E-130	down	6.43E-01	8.66E-08	no
ENSMUSG00000000942	Hoxa4	-1.98E+00	7.56E-14	down	-1.78E+00	2.57E-17	down
ENSMUSG00000045725	Prr15	-5.13E+00	3.29E-06	down	-2.18E+00	2.28E-01	no
ENSMUSG00000033940	Brk1	2.10E+00	6.51E-20	up	2.70E-01	1.96E-01	no
ENSMUSG00000079346	1700013D24Rik	-2.33E+00	2.12E-81	down	-4.84E-01	4.29E-02	no
ENSMUSG00000051956	Rnf133	-5.11E+00	2.17E-104	down	-3.00E+00	1.57E-14	down
ENSMUSG00000029682	Spam1	-4.33E+00	5.00E-55	down	-1.04E+00	6.27E-02	no
ENSMUSG00000029678	Hyal5	-4.85E+00	2.19E-94	down	-2.29E+00	3.13E-07	down
ENSMUSG00000037621	Atoh8	-1.45E+00	8.15E-17	down	-1.03E+00	2.02E-06	down
ENSMUSG00000072952	Gm5878	-1.13E+00	2.62E-01	no	-2.72E+00	3.41E-13	down
ENSMUSG00000034203	Chchd4	2.32E+00	2.88E-06	up	3.26E-01	4.74E-01	no
ENSMUSG00000057841	Rpl32	1.27E+00	6.72E-130	up	1.56E-01	3.12E-03	no
ENSMUSG00000029632	Ndufa4	3.98E+00	1.64E-39	up	1.33E+00	3.79E-07	up
ENSMUSG00000030161	Gabarapl1	-1.42E+00	7.15E-17	down	-1.01E+00	5.50E-05	down
ENSMUSG00000047515	BC049715	-3.95E+00	2.90E-58	down	-2.23E+00	1.20E-07	down
ENSMUSG00000030218	Mgp	5.17E+00	2.00E-141	up	2.58E+00	3.29E-05	up
ENSMUSG00000029679	Hyal6	-3.73E+00	1.23E-22	down	-3.02E+00	8.23E-16	down
ENSMUSG00000043421	Hilpda	1.13E+00	1.04E-04	up	1.81E-02	9.62E-01	no
ENSMUSG00000029840	Mtpn	4.16E-01	5.14E-01	no	-1.53E+00	5.49E-04	down
ENSMUSG00000019689	1110001J03Rik	2.23E+00	1.21E-17	up	1.80E+00	5.65E-18	up
ENSMUSG00000051896	Tex37	-3.21E+00	2.41E-82	down	-2.18E+00	2.79E-13	down
ENSMUSG00000054422	Fabp1	3.09E+00	4.36E-07	up	2.72E+00	5.30E-03	no
ENSMUSG00000058706	0610030E20Rik	-7.11E-01	5.01E-01	no	-2.30E+00	1.74E-04	down
ENSMUSG00000030030	1700003E16Rik	-1.98E+00	1.28E-91	down	-1.13E+00	3.95E-28	down
ENSMUSG00000057278	Snrpg	3.71E+00	2.28E-61	up	2.64E+00	3.22E-30	up
ENSMUSG00000064177	Ghrl	-4.35E-01	7.83E-02	no	-1.18E+00	1.20E-14	down
ENSMUSG00000030137	Tuba8	-1.80E+00	4.22E-31	down	2.34E-02	9.66E-01	no
ENSMUSG00000067702	Tuba3a	2.35E+00	0.00E+00	up	2.21E+00	0.00E+00	up
ENSMUSG00000047720	4922502D21Rik	-3.05E+00	0.00E+00	down	-2.77E+00	0.00E+00	down

ENSMUSG00000067338	Tuba3b	2.50E+00	0.00E+00	up	2.13E+00	0.00E+00	up
ENSMUSG00000050732	Vamp8	3.74E+00	2.49E-05	up	2.10E+00	7.84E-02	no
ENSMUSG00000051343	Rab11fip5	-3.27E+00	2.04E-34	down	-2.73E+00	8.55E-25	down
ENSMUSG00000030344	Akap3	-5.60E+00	0.00E+00	down	-2.87E+00	5.07E-27	down
ENSMUSG00000079304	4933413G19Rik	-4.80E+00	5.04E-10	down	-3.39E+00	4.83E-02	no
ENSMUSG00000079299	Klrb1	-3.63E+00	1.09E-12	down	-2.63E+00	1.90E-02	no
ENSMUSG00000040163	1700034J05Rik	-2.93E+00	2.78E-85	down	-8.07E-01	1.97E-03	no
ENSMUSG00000051936	Prss58	-4.47E+00	7.06E-39	down	-2.87E+00	3.07E-09	down
ENSMUSG00000009281	Rarres2	4.28E+00	9.45E-15	up	3.02E+00	2.04E-02	no
ENSMUSG00000042607	Asb4	-2.38E+00	2.57E-06	down	-2.18E+00	9.34E-07	down
ENSMUSG00000053012	Krcc1	-1.44E+00	4.20E-13	down	-2.24E+00	1.75E-44	down
ENSMUSG00000014747	Ankrd53	-2.96E+00	3.80E-24	down	-2.35E-01	7.85E-01	no
ENSMUSG00000001156	Mxd1	-2.83E+00	4.08E-09	down	-2.49E+00	7.42E-07	down
ENSMUSG00000030105	Arl8b	-2.25E+00	7.85E-18	down	-2.56E+00	6.20E-26	down
ENSMUSG00000068011	2510049J12Rik	4.26E+00	2.51E-06	up	4.62E+00	1.37E-08	up
ENSMUSG00000052763	Zfp212	-2.42E-01	7.94E-01	no	-1.92E+00	1.26E-05	down
ENSMUSG00000057691	Zfp746	-3.69E-01	6.92E-01	no	-2.23E+00	8.58E-05	down
ENSMUSG00000073002	Vamp5	1.71E-01	8.49E-01	no	-1.63E+00	2.56E-05	down
ENSMUSG00000055239	Kcmf1	-2.36E+00	3.67E-28	down	-2.03E+00	2.03E-25	down
ENSMUSG00000061762	Tac1	-3.56E+00	1.11E-06	down	-1.72E+00	2.48E-01	no
ENSMUSG00000084950	Gm5577	-2.32E+00	2.00E-02	no	-3.26E+00	3.59E-06	down
ENSMUSG00000000439	Mkrn2	-2.54E+00	2.48E-13	down	-2.66E+00	1.39E-18	down
ENSMUSG00000030275	Etnk1	-2.40E+00	1.61E-06	down	-3.05E+00	3.14E-13	down
ENSMUSG00000039032	Tsga13	-3.16E+00	1.97E-37	down	-7.10E-01	1.37E-01	no
ENSMUSG00000029766	1700012A03Rik	-5.29E+00	2.07E-98	down	-4.49E+00	2.50E-29	down
ENSMUSG00000029909	Prss37	-3.94E+00	3.53E-97	down	-2.04E+00	1.09E-08	down
ENSMUSG00000029817	Tra2a	-1.13E+00	2.07E-03	no	-3.14E+00	5.38E-45	down
ENSMUSG00000036371	Serbp1	-1.31E+00	7.84E-05	down	-2.08E+00	2.62E-17	down
ENSMUSG00000052144	Ppp4r2	-2.80E+00	8.46E-07	down	-2.30E+00	2.09E-04	down
ENSMUSG00000033963	Fancd2os	-2.78E+00	8.90E-30	down	3.26E-02	9.70E-01	no
ENSMUSG00000033933	Vhl	-1.68E+00	7.09E-08	down	-2.08E+00	5.55E-09	down
ENSMUSG00000019210	Atp6v1e1	1.35E+00	1.07E-04	up	-8.94E-01	7.97E-05	no
ENSMUSG00000029708	Gcc1	-3.51E+00	2.46E-04	down	-2.62E+00	1.49E-02	no
ENSMUSG00000029784	Ssmem1	-3.54E+00	1.87E-139	down	-2.77E+00	3.81E-81	down
ENSMUSG00000053907	Mat2a	1.52E+00	3.39E-05	up	-1.06E+00	8.27E-04	down
ENSMUSG00000049694	BC048671	-3.29E+00	2.66E-59	down	-4.07E-01	4.12E-01	no
ENSMUSG00000034192	Lsm3	1.62E+00	1.14E-07	up	-1.42E+00	3.46E-18	down
ENSMUSG00000030069	Prok2	-7.36E-01	9.17E-02	no	-1.92E+00	7.75E-28	down
ENSMUSG00000030286	Emc3	8.44E-02	8.77E-01	no	-1.69E+00	5.55E-09	down
ENSMUSG00000029707	Fscn3	-6.01E+00	1.63E-42	down	-4.83E+00	4.64E-09	down
ENSMUSG00000044156	Hepacam2	-8.54E-01	7.33E-01	no	-3.56E+00	9.63E-05	down
ENSMUSG00000073096	Lrrc61	-1.14E+00	1.75E-01	no	-2.95E+00	1.20E-16	down
ENSMUSG00000029828	4921507P07Rik	-6.11E+00	4.27E-96	down	-4.05E+00	6.97E-14	down
ENSMUSG00000006906	Stambp	1.80E+00	2.73E-10	up	-2.25E-01	3.39E-01	no
ENSMUSG00000033735	Spr	5.15E+00	5.28E-04	up	2.60E+00	7.78E-02	no
ENSMUSG00000030062	Rpn1	-1.04E+00	5.38E-08	down	-1.72E+00	5.47E-28	down
ENSMUSG00000038871	Bpgm	-1.85E+00	2.99E-12	down	-1.24E+00	5.54E-06	down
ENSMUSG00000079523	Tmsb10	9.31E-01	5.40E-75	no	1.02E+00	7.21E-139	up
ENSMUSG00000038451	Spsb2	-3.13E+00	2.89E-16	down	-1.98E+00	2.37E-05	down
ENSMUSG00000029517	Ankrd7	-3.80E+00	1.48E-134	down	-2.90E+00	1.97E-65	down
ENSMUSG00000045466	Zfp956	-2.21E+00	1.21E-10	down	-3.43E+00	3.86E-69	down
ENSMUSG00000030041	M1ap	-1.48E+00	4.80E-11	down	-2.22E-01	7.83E-01	no
ENSMUSG00000030007	Cct7	-1.14E+00	3.83E-41	down	-8.22E-01	8.51E-27	no
ENSMUSG00000015053	Gata2	-2.00E+00	2.02E-05	down	2.11E-01	8.87E-01	no
ENSMUSG00000058979	Cecr5	-4.26E-01	7.55E-01	no	-2.34E+00	1.06E-04	down
ENSMUSG00000046192	Iqub	-3.75E+00	6.51E-52	down	-2.25E+00	1.50E-12	down
ENSMUSG00000020440	Arf5	1.15E+00	1.23E-04	up	-6.68E-02	8.69E-01	no
ENSMUSG00000079652	Fam71f2	-4.78E+00	5.17E-149	down	-2.94E+00	4.53E-24	down
ENSMUSG00000059182	Skap2	1.19E+00	8.20E-02	no	-1.63E+00	4.21E-07	down
ENSMUSG00000063568	Jazf1	-2.94E+00	1.36E-09	down	-1.74E+00	2.50E-03	no
ENSMUSG00000052955	Cpvl	-2.69E+00	8.77E-10	down	3.47E-01	8.03E-01	no
ENSMUSG00000058446	Znrf2	-6.84E-01	2.17E-01	no	-1.85E+00	3.99E-09	down
ENSMUSG00000029754	Dlx6	-6.35E+00	5.57E-04	down	-3.25E+00	2.94E-01	no
ENSMUSG00000030031	Kbtbd8	-1.77E+00	3.45E-01	no	-3.69E+00	1.00E-05	down

ENSMUSG00000023367	Tmem176a	6.49E+00	8.10E-13	up	4.93E+00	3.75E-08	up
ENSMUSG00000006269	Atp6v1b1	-5.65E+00	2.84E-10	down	-2.78E+00	1.57E-01	no
ENSMUSG00000030213	Atf7ip	-2.47E+00	8.20E-05	down	-2.74E+00	2.21E-06	down
ENSMUSG00000030224	Strap	-2.59E+00	5.14E-37	down	-2.91E+00	3.85E-51	down
ENSMUSG00000029814	Igf2bp3	-3.06E+00	2.97E-12	down	-1.55E+00	6.00E-03	no
ENSMUSG00000030096	Slc6a6	-2.01E+00	1.71E-08	down	4.72E-01	6.65E-01	no
ENSMUSG00000038279	Nop2	-2.04E+00	3.11E-06	down	-1.89E+00	1.61E-04	down
ENSMUSG00000048668	Rhno1	-1.94E+00	1.77E-10	down	-4.98E-01	3.63E-01	no
ENSMUSG00000014748	Tex261	-6.61E-01	6.90E-02	no	-1.43E+00	1.02E-07	down
ENSMUSG00000023089	Ndufa5	2.72E+00	8.61E-162	up	2.26E+00	4.55E-146	up
ENSMUSG00000029883	1700074P13Rik	-2.79E+00	3.53E-161	down	-2.11E+00	2.62E-86	down
ENSMUSG00000034063	4930590J08Rik	-5.22E+00	5.88E-37	down	-3.92E+00	6.17E-11	down
ENSMUSG00000003178	Mical3	-5.52E+00	1.21E-45	down	-3.58E+00	3.60E-07	down
ENSMUSG00000030120	Mlf2	1.62E+00	3.51E-07	up	-3.39E-01	3.28E-01	no
ENSMUSG00000030246	Ldhb	3.36E+00	6.87E-09	up	2.08E+00	7.26E-07	up
ENSMUSG00000002416	Ndufb2	2.66E+00	5.40E-07	up	1.60E+00	4.19E-05	up
ENSMUSG00000032667	Pon2	-1.27E+00	3.15E-02	no	-2.16E+00	3.86E-04	down
ENSMUSG00000038022	Fam188b	-2.34E+00	2.69E-08	down	-2.01E+00	2.04E-08	down
ENSMUSG00000030342	Cd9	3.94E+00	4.39E-07	up	2.98E+00	4.36E-02	no
ENSMUSG00000030189	Ybx3	-2.71E+00	0.00E+00	down	-2.44E+00	0.00E+00	down
ENSMUSG00000023456	Tpi1	-2.62E+00	1.78E-68	down	-1.57E+00	4.97E-23	down
ENSMUSG00000029685	Asb15	-3.93E+00	6.21E-10	down	-1.39E+00	4.11E-01	no
ENSMUSG00000029861	Fam131b	-5.60E+00	6.88E-04	down	-4.73E+00	2.13E-01	no
ENSMUSG00000030042	Pole4	3.12E+00	2.56E-10	up	8.03E-01	6.60E-02	no
ENSMUSG00000056952	Tatdn2	-3.83E+00	1.28E-03	no	-5.39E+00	3.53E-06	down
ENSMUSG00000030220	Arhgdib	3.54E+00	1.96E-08	up	1.25E+00	3.11E-01	no
ENSMUSG00000030089	Slc41a3	-1.62E+00	1.56E-05	down	-1.35E+00	1.37E-04	down
ENSMUSG00000040649	Rimklb	-2.23E+00	1.97E-10	down	-2.43E+00	7.78E-16	down
ENSMUSG00000029552	Tes	-2.95E+00	2.28E-24	down	-3.27E-01	6.30E-01	no
ENSMUSG00000029810	Tmem176b	3.45E+00	7.06E-07	up	3.98E+00	3.00E-04	up
ENSMUSG00000047115	Fam221a	4.36E-03	9.97E-01	no	-2.48E+00	2.39E-08	down
ENSMUSG00000079511	Ccdc142	-1.27E+00	5.35E-01	no	-3.90E+00	7.22E-06	down
ENSMUSG00000001157	Gmcl1	-9.94E-01	1.46E-02	no	-3.01E+00	2.93E-58	down
ENSMUSG00000030057	Cnbp	1.98E+00	5.44E-33	up	-1.33E-02	9.60E-01	no
ENSMUSG00000030060	8430410A17Rik	1.62E+00	9.42E-04	up	5.51E-01	5.30E-01	no
ENSMUSG00000030271	Ogg1	-2.77E+00	3.78E-04	down	-2.69E+00	1.44E-05	down
ENSMUSG00000030138	Bms1	-3.32E+00	5.33E-20	down	-3.57E+00	3.41E-34	down
ENSMUSG00000030214	Plbd1	-3.71E+00	6.64E-12	down	-5.47E-01	6.80E-01	no
ENSMUSG00000029798	Herc6	-2.18E+00	2.98E-06	down	-2.01E+00	1.43E-06	down
ENSMUSG00000063810	Alms1	-4.31E+00	8.41E-11	down	-3.49E+00	2.64E-05	down
ENSMUSG00000030301	Ccdc91	-3.56E+00	2.42E-92	down	-1.66E+00	3.29E-09	down
ENSMUSG00000063884	Ptcd3	-2.06E+00	9.35E-05	down	-2.99E+00	4.84E-12	down
ENSMUSG00000052738	Suclg1	-1.14E+00	1.66E-05	down	-1.09E+00	8.50E-07	down
ENSMUSG00000030125	Lrrc23	-3.31E+00	6.52E-47	down	-3.36E+00	1.32E-77	down
ENSMUSG00000040234	Tm7sf3	1.70E+00	2.18E-02	no	2.69E+00	6.83E-04	up
ENSMUSG00000030077	Chl1	-4.45E+00	1.42E-71	down	-1.77E+00	1.93E-04	down
ENSMUSG00000001517	Foxm1	-3.82E+00	6.25E-28	down	-2.58E+00	3.69E-10	down
ENSMUSG00000030200	Bcl2l14	-4.25E+00	4.22E-82	down	-1.84E+00	1.85E-06	down
ENSMUSG00000030216	Wbp11	-2.52E+00	1.90E-49	down	-2.11E+00	1.22E-38	down
ENSMUSG00000008540	Mgst1	3.25E+00	6.75E-04	up	1.62E+00	5.11E-02	no
ENSMUSG00000030292	Smco2	-2.47E+00	1.70E-51	down	-2.45E+00	2.11E-78	down
ENSMUSG00000029848	Stra8	5.16E+00	1.21E-24	up	4.08E+00	2.52E-08	up
ENSMUSG00000053119	Chmp3	-2.82E-01	5.61E-01	no	-1.53E+00	5.91E-07	down
ENSMUSG00000045160	Bola3	2.02E+00	2.39E-18	up	1.91E+00	1.96E-28	up
ENSMUSG00000023505	Cdca3	-1.54E+00	1.61E-22	down	-1.44E+00	4.60E-25	down
ENSMUSG00000056215	Lrguk	-3.16E+00	9.04E-08	down	-3.22E+00	6.76E-12	down
ENSMUSG00000005225	Plekha8	-7.81E-01	3.72E-01	no	-2.14E+00	3.06E-04	down
ENSMUSG00000072770	Acrbp	-1.35E+00	1.38E-21	down	-1.14E+00	8.77E-18	down
ENSMUSG00000030206	Gsg1	-1.61E+00	9.34E-150	down	7.93E-01	6.07E-15	no
ENSMUSG00000039841	Zfp800	-6.22E-01	4.15E-01	no	-2.13E+00	6.30E-06	down
ENSMUSG00000030346	Rad51ap1	-2.48E-01	7.57E-01	no	-1.70E+00	2.33E-04	down
ENSMUSG00000030188	Magohb	-4.92E-01	5.82E-01	no	-2.06E+00	3.34E-04	down
ENSMUSG00000032641	Gpr19	-1.55E+00	4.53E-03	no	-1.81E+00	8.25E-06	down
ENSMUSG00000000811	Txnrd3	-2.86E+00	4.86E-99	down	-2.41E+00	3.39E-78	down

ENSMUSG00000030231	Plekha5	-2.30E+00	2.04E-05	down	-2.84E+00	1.43E-10	down
ENSMUSG00000039742	Fam71f1	-4.82E+00	9.04E-140	down	-2.64E+00	1.01E-15	down
ENSMUSG00000039419	Cntnap2	-2.68E+00	1.88E-03	no	-3.31E+00	8.78E-06	down
ENSMUSG00000038252	Ncapd2	-2.05E+00	4.11E-05	down	-3.38E+00	3.42E-17	down
ENSMUSG00000030264	Thumpd3	-3.09E+00	1.73E-22	down	-3.17E+00	4.54E-32	down
ENSMUSG00000030126	Tmcc1	-4.19E+00	2.04E-17	down	-3.97E+00	6.34E-14	down
ENSMUSG00000041477	Dcp1b	-2.63E+00	4.83E-08	down	-1.90E+00	8.53E-05	down
ENSMUSG00000030345	Dyrk4	-4.61E+00	9.70E-15	down	-2.83E+00	1.94E-02	no
ENSMUSG00000030357	Fkbp4	-1.56E+00	2.84E-48	down	-1.47E+00	8.10E-62	down
ENSMUSG00000004980	Hnrnpa2b1	1.10E+00	6.42E-09	up	-1.24E+00	3.73E-20	down
ENSMUSG00000030059	Tmf1	-1.40E+00	3.74E-20	down	-1.46E+00	1.90E-15	down
ENSMUSG00000030341	Tnfrsf1a	4.22E+00	3.00E-06	up	4.20E+00	8.11E-12	up
ENSMUSG00000038784	Cnot4	-1.90E+00	2.23E-07	down	-1.72E+00	7.10E-06	down
ENSMUSG00000049553	Polr1a	-1.91E+00	1.05E-02	no	-2.65E+00	4.06E-06	down
ENSMUSG00000034245	Hdac11	-5.13E+00	8.88E-22	down	-2.83E+00	9.08E-03	no
ENSMUSG00000042213	Zfand4	-3.22E+00	5.28E-46	down	-2.55E+00	1.35E-29	down
ENSMUSG00000055027	Smyd1	-3.64E+00	6.22E-02	no	-5.53E+00	9.19E-05	down
ENSMUSG00000059430	Actg2	-2.62E+00	1.96E-76	down	-6.15E-01	4.83E-02	no
ENSMUSG00000030061	Uba3	6.42E-01	4.98E-01	no	-1.79E+00	4.37E-04	down
ENSMUSG00000048930	Tada3	-2.34E+00	2.26E-08	down	-2.60E+00	8.47E-15	down
ENSMUSG00000054966	Ifltd1	-3.90E+00	6.29E-07	down	-1.03E+00	6.20E-01	no
ENSMUSG00000062190	Lancl2	-3.09E+00	1.13E-42	down	-2.78E+00	6.51E-38	down
ENSMUSG00000013736	Trnt1	-2.39E+00	3.85E-08	down	-2.49E+00	9.57E-09	down
ENSMUSG00000029922	Mkrn1	-2.17E+00	1.10E-53	down	-1.84E+00	2.15E-46	down
ENSMUSG00000004535	Tax1bp1	-2.54E+00	1.42E-32	down	-1.70E+00	1.20E-08	down
ENSMUSG00000029752	Asns	-3.25E+00	2.17E-22	down	-6.08E-01	4.13E-01	no
ENSMUSG00000000628	Hk2	3.31E+00	8.70E-04	up	1.62E+00	3.13E-01	no
ENSMUSG00000030082	Sec61a1	-2.04E+00	6.94E-12	down	-1.58E+00	1.47E-05	down
ENSMUSG00000030313	Dennd5b	-1.72E+00	2.22E-01	no	-3.06E+00	7.02E-04	down
ENSMUSG00000068250	Amn1	-1.29E+00	1.85E-05	down	-1.05E+00	7.20E-05	down
ENSMUSG00000042079	Hnrnpf	-1.77E+00	1.90E-15	down	-2.13E+00	4.57E-26	down
ENSMUSG00000015733	Capza2	2.72E+00	2.14E-08	up	-1.37E-01	8.75E-01	no
ENSMUSG00000029788	Cpa5	-5.03E+00	1.31E-32	down	-2.06E+00	1.81E-02	no
ENSMUSG00000005362	Crbn	-1.89E+00	5.44E-08	down	-2.06E+00	1.62E-09	down
ENSMUSG00000003153	Slc2a3	-2.06E+00	2.97E-24	down	-3.14E+00	4.78E-180	down
ENSMUSG00000033174	Mgll	-7.78E-01	3.13E-01	no	-1.94E+00	6.72E-15	down
ENSMUSG00000037997	Parp11	-3.14E+00	1.82E-05	down	-2.15E+00	4.60E-03	no
ENSMUSG00000030230	Plcz1	-5.10E+00	4.80E-120	down	-2.66E+00	6.10E-11	down
ENSMUSG00000029701	Rbm28	-1.85E+00	2.38E-05	down	-1.16E+00	4.49E-02	no
ENSMUSG00000040250	Asun	-3.39E+00	2.86E-24	down	-3.34E+00	5.23E-27	down
ENSMUSG00000029775	Klhdc10	-3.40E+00	1.24E-07	down	-2.90E+00	1.17E-04	down
ENSMUSG00000038759	Nup205	-3.26E+00	3.70E-25	down	-3.77E+00	3.72E-52	down
ENSMUSG00000004270	Lpcat3	-2.02E+00	3.25E-20	down	-1.73E+00	3.03E-17	down
ENSMUSG00000056832	Ttc26	-1.67E+00	1.27E-02	no	-2.68E+00	1.37E-08	down
ENSMUSG00000055850	Rnf181	-1.69E+00	4.70E-16	down	-1.34E+00	3.37E-13	down
ENSMUSG00000053470	Kdm3a	-2.56E+00	1.12E-13	down	-3.40E+00	1.92E-34	down
ENSMUSG00000056698	Elmod3	-2.43E+00	2.08E-05	down	-2.67E+00	2.65E-09	down
ENSMUSG00000029992	Gfpt1	-2.70E+00	1.47E-10	down	-2.46E+00	1.68E-09	down
ENSMUSG00000048794	Ccdc37	-4.00E+00	7.16E-22	down	-8.35E-01	3.76E-01	no
ENSMUSG00000053768	Chchd3	-1.63E+00	3.07E-17	down	-1.45E-01	7.37E-01	no
ENSMUSG00000046679	C87436	-2.92E+00	1.24E-18	down	-8.98E-01	1.09E-01	no
ENSMUSG00000004633	Chn2	-3.88E+00	2.29E-57	down	-1.60E+00	2.35E-04	down
ENSMUSG00000043541	Casc1	-4.61E+00	4.90E-12	down	-3.28E+00	6.37E-07	down
ENSMUSG00000000441	Raf1	-2.80E-01	7.69E-01	no	-1.98E+00	8.37E-04	down
ENSMUSG00000038388	Mpp6	-2.16E+00	8.91E-19	down	-2.03E+00	3.51E-21	down
ENSMUSG00000029790	Cep41	-2.76E+00	1.19E-10	down	-1.61E+00	3.47E-03	no
ENSMUSG00000029686	Cul1	-1.95E+00	5.23E-11	down	-2.13E+00	7.45E-12	down
ENSMUSG00000056737	Capg	4.30E+00	1.08E-04	up	3.89E+00	8.61E-02	no
ENSMUSG00000030177	Ccdc77	-7.58E-01	5.57E-01	no	-2.72E+00	3.07E-06	down
ENSMUSG00000052631	Sh2d6	-2.37E+00	1.14E-09	down	-1.49E+00	5.15E-04	down
ENSMUSG00000059900	Tmem40	6.53E+00	1.60E-03	no	5.73E+00	2.95E-07	up
ENSMUSG00000030276	Ttll3	-4.30E+00	4.33E-19	down	-1.42E+00	1.30E-01	no
ENSMUSG00000030243	Recql	-2.75E+00	1.54E-04	down	-3.07E+00	1.94E-04	down
ENSMUSG00000030016	Zfml	-1.77E+00	2.52E-02	no	-4.24E+00	1.91E-17	down

ENSMUSG00000025607	Copg2	-3.29E+00	2.10E-06	down	-4.67E+00	4.87E-15	down
ENSMUSG00000023403	Stk31	2.36E-01	6.41E-01	no	-2.20E+00	1.42E-20	down
ENSMUSG00000029833	Trim24	-4.06E+00	4.27E-25	down	-3.80E+00	1.03E-24	down
ENSMUSG00000030254	Rad18	-6.69E-01	3.28E-01	no	-1.60E+00	4.74E-04	down
ENSMUSG00000029670	Ing3	-2.31E+00	2.74E-04	down	-1.20E+00	2.50E-01	no
ENSMUSG00000052337	Immt	-2.30E+00	1.06E-21	down	-2.00E+00	1.35E-15	down
ENSMUSG00000042810	Krba1	-2.74E+00	3.10E-06	down	-3.02E+00	1.99E-09	down
ENSMUSG00000038271	Iffo1	-1.21E+00	1.68E-01	no	-2.63E+00	2.02E-08	down
ENSMUSG00000029769	Ccdc136	-2.70E+00	3.66E-181	down	-2.06E+00	1.29E-105	down
ENSMUSG00000029823	Luc7l2	-1.29E+00	1.68E-01	no	-3.29E+00	1.19E-07	down
ENSMUSG00000038836	Agbl3	-3.41E+00	1.92E-07	down	-2.11E+00	1.13E-02	no
ENSMUSG00000030304	Ergic2	-2.66E+00	1.21E-12	down	-2.01E+00	2.27E-05	down
ENSMUSG00000062995	Ica1	-3.60E+00	5.40E-30	down	-6.41E-01	3.33E-01	no
ENSMUSG00000029782	Tmem209	-1.54E+00	6.87E-02	no	-2.35E+00	8.62E-04	down
ENSMUSG00000029687	Ezh2	-1.80E+00	1.31E-04	down	-1.45E+00	2.01E-03	no
ENSMUSG00000041301	Cftr	-3.44E+00	5.98E-04	down	-2.16E+00	1.66E-01	no
ENSMUSG00000041540	Sox5	-2.47E+00	4.95E-08	down	4.30E-01	7.35E-01	no
ENSMUSG00000063870	Chd4	-2.11E+00	5.11E-10	down	-2.29E+00	9.46E-13	down
ENSMUSG00000038346	Zfp384	-2.62E+00	8.12E-04	down	-3.18E+00	7.81E-06	down
ENSMUSG00000004347	Pde1c	-2.87E+00	2.84E-10	down	-2.81E+00	1.96E-13	down
ENSMUSG00000030314	Atg7	-2.45E+00	1.35E-05	down	-2.16E+00	1.69E-04	down
ENSMUSG00000040669	Phc1	-3.69E+00	2.61E-28	down	-2.81E+00	8.19E-16	down
ENSMUSG00000030323	Ift122	-3.02E+00	1.83E-17	down	-2.65E+00	1.37E-15	down
ENSMUSG00000052861	Dnahc6	-4.16E+00	2.56E-07	down	-4.01E+00	2.55E-09	down
ENSMUSG00000031865	Dctn1	-1.54E+00	4.63E-03	no	-1.95E+00	8.00E-05	down
ENSMUSG00000029772	Ahcyl2	-3.07E+00	1.47E-06	down	-2.67E+00	5.29E-05	down
ENSMUSG00000045962	Wnk1	-2.59E+00	1.70E-08	down	-3.67E+00	1.12E-22	down
ENSMUSG00000070802	Pnmal2	-3.46E+00	2.66E-15	down	-1.05E+00	2.95E-01	no
ENSMUSG00000055826	Tescl	-1.56E+00	4.49E-34	down	5.62E-01	1.06E-02	no
ENSMUSG00000050347	Gm9844	2.53E+00	5.75E-31	up	2.64E+00	2.89E-48	up
ENSMUSG00000046058	Eid2	-1.20E+00	2.50E-01	no	-2.66E+00	4.71E-06	down
ENSMUSG00000051976	6330444E15Rik	-4.21E+00	5.50E-10	down	-6.82E-01	6.61E-01	no
ENSMUSG00000094462	Gm21028	-3.10E+00	1.87E-01	no	-4.39E+00	8.81E-06	down
ENSMUSG00000070563	Spaca4	-4.19E-02	7.42E-01	no	2.11E+00	6.67E-43	up
ENSMUSG00000084234	4933405O20Rik	-3.10E+00	4.02E-49	down	2.34E-01	7.02E-01	no
ENSMUSG00000047370	Gm7367	-3.38E+00	1.31E-10	down	-1.78E+00	6.88E-02	no
ENSMUSG00000033585	Ndn	4.60E+00	4.26E-06	up	3.47E+00	6.27E-02	no
ENSMUSG00000094854	Gm5334	-6.18E+00	4.58E-14	down	-3.94E+00	1.20E-02	no
ENSMUSG00000039133	9330171B17Rik	-2.21E+00	1.12E-10	down	-1.85E+00	7.93E-09	down
ENSMUSG00000044362	Ccdc89	-2.58E+00	1.47E-60	down	-1.23E+00	1.94E-11	down
ENSMUSG00000055643	4931431F19Rik	-5.22E+00	3.96E-28	down	-3.04E+00	1.06E-03	no
ENSMUSG00000051437	Ubqlnl	-6.26E+00	1.11E-55	down	-3.87E+00	1.19E-07	down
ENSMUSG00000073894	Rbmxl2	1.55E+00	4.91E-14	up	1.08E+00	9.59E-09	up
ENSMUSG00000051614	4930533L02Rik	-3.02E+00	3.76E-08	down	-1.07E+00	4.10E-01	no
ENSMUSG00000090457	4930571K23Rik	-3.71E+00	1.59E-186	down	-1.11E+00	9.80E-07	down
ENSMUSG00000097810	AC125169.1	4.32E+00	8.46E-05	up	3.22E+00	2.74E-03	no
ENSMUSG00000049091	Sephs2	-1.49E+00	1.22E-23	down	-1.50E+00	9.31E-32	down
ENSMUSG00000062758	Gm16477	-4.52E+00	2.12E-08	down	-2.26E+00	2.31E-01	no
ENSMUSG00000051618	Ubqln3	-3.60E+00	2.24E-137	down	-8.56E-01	1.90E-03	no
ENSMUSG00000037060	Prkcdbp	4.39E+00	3.10E-05	up	2.25E+00	3.19E-01	no
ENSMUSG00000091900	Gm4353	-2.60E+00	2.23E-17	down	-1.54E+00	5.82E-05	down
ENSMUSG00000045165	AI467606	-1.32E+00	4.34E-01	no	-3.53E+00	1.37E-08	down
ENSMUSG00000047371	Zfp768	-2.87E+00	6.40E-11	down	-2.11E+00	2.25E-04	down
ENSMUSG00000030858	1700007K09Rik	-7.64E-01	7.10E-21	no	1.40E+00	6.76E-24	up
ENSMUSG00000060591	Ifitm2	7.42E+00	2.84E-263	up	5.90E+00	3.23E-34	up
ENSMUSG00000025492	Ifitm3	7.26E+00	1.40E-217	up	5.04E+00	1.87E-27	up
ENSMUSG00000038489	Polr2l	4.46E+00	5.59E-06	up	3.22E+00	1.54E-05	up
ENSMUSG00000062028	Irgc1	-2.43E+00	1.37E-197	down	-2.38E-01	1.42E-01	no
ENSMUSG00000090330	9130221H12Rik	-4.71E+00	1.24E-08	down	-3.83E+00	4.16E-08	down
ENSMUSG00000044786	Zfp36	2.71E+00	2.82E-09	up	7.37E-01	5.36E-01	no
ENSMUSG00000049761	4930479M11Rik	-2.43E+00	3.96E-46	down	-3.04E-01	5.34E-01	no
ENSMUSG00000013083	2200002J24Rik	-2.63E+00	7.99E-22	down	-2.73E-01	7.47E-01	no
ENSMUSG00000091692	4930433I11Rik	-3.07E+00	1.92E-11	down	2.27E-01	8.85E-01	no
ENSMUSG00000030544	Mesp1	1.14E+00	3.26E-11	up	7.17E-01	5.99E-15	no

ENSMUSG00000057706	Mex3b	-3.62E+00	2.34E-07	down	-2.46E+00	1.35E-01	no
ENSMUSG00000045928	4933440M02Rik	-9.58E-01	1.08E-01	no	-1.32E+00	1.56E-04	down
ENSMUSG00000042462	Dctpp1	4.02E+00	6.87E-65	up	2.15E+00	2.39E-15	up
ENSMUSG00000090663	Srcap	-8.90E-01	2.81E-01	no	-2.70E+00	1.21E-07	down
ENSMUSG00000060034	Ctf2	6.18E+00	5.53E-07	up	4.66E+00	9.21E-03	no
ENSMUSG00000013668	4933402N03Rik	-5.92E+00	1.25E-10	down	-5.34E+00	1.05E-02	no
ENSMUSG00000030859	Fam24a	-3.84E-01	9.42E-05	no	1.68E+00	5.33E-28	up
ENSMUSG00000019158	Tmem160	7.33E+00	5.15E-09	up	6.39E+00	1.19E-04	up
ENSMUSG00000041141	Pnmal1	-1.48E+00	1.65E-01	no	-4.11E+00	1.98E-17	down
ENSMUSG00000046541	Zfp526	-4.57E+00	1.50E-08	down	-3.68E+00	2.55E-04	down
ENSMUSG00000058741	Prr19	-2.30E+00	4.27E-19	down	-2.37E+00	4.53E-30	down
ENSMUSG00000063439	B9d2	2.76E+00	8.03E-08	up	1.41E+00	3.60E-04	up
ENSMUSG00000030604	Zfp626	-4.30E+00	7.10E-10	down	-2.99E+00	4.07E-03	no
ENSMUSG00000055452	Gm7353	-4.69E+00	2.99E-09	down	-3.91E+00	5.93E-06	down
ENSMUSG00000094233	Gm21276	-3.75E+00	7.58E-04	down	-1.63E+00	6.03E-01	no
ENSMUSG00000053742	Gm5114	-3.38E+00	4.53E-04	down	-2.27E-01	9.44E-01	no
ENSMUSG00000078670	Fam174b	-9.79E-01	5.20E-01	no	-3.44E+00	3.12E-08	down
ENSMUSG00000042797	Aqp11	-2.29E+00	1.38E-80	down	-1.79E+00	8.75E-42	down
ENSMUSG00000045989	4930451I11Rik	-1.34E+00	3.01E-13	down	3.61E-01	3.63E-01	no
ENSMUSG00000030976	Tex36	-3.19E+00	2.27E-153	down	-7.88E-01	2.75E-04	no
ENSMUSG00000078794	Dact3	-3.12E+00	1.04E-04	down	-1.13E+00	3.97E-01	no
ENSMUSG00000050428	Fbxo46	-4.07E+00	2.86E-08	down	-2.55E+00	2.44E-03	no
ENSMUSG00000058717	Gm4763	1.48E+00	2.82E-09	up	-3.37E-01	6.49E-03	no
ENSMUSG00000045252	Zfp574	-3.24E+00	5.69E-30	down	-2.59E+00	1.45E-25	down
ENSMUSG00000003444	Med29	5.28E-01	8.76E-02	no	1.31E+00	2.21E-05	up
ENSMUSG00000037166	Ppp1r14a	4.35E+00	1.10E-08	up	1.08E+00	2.20E-01	no
ENSMUSG00000036751	Cox6b1	1.97E+00	6.65E-28	up	1.32E+00	1.11E-10	up
ENSMUSG00000056216	Cebpg	-1.59E+00	1.95E-04	down	-2.38E+00	1.08E-12	down
ENSMUSG00000056383	AI987944	-1.90E+00	1.02E-09	down	-1.58E+00	7.32E-07	down
ENSMUSG00000030463	4933421I07Rik	-1.80E+00	4.78E-05	down	-8.27E-01	1.19E-01	no
ENSMUSG00000040364	Sec1	-2.09E+00	2.39E-05	down	-1.64E+00	3.76E-04	down
ENSMUSG00000094445	1700015G11Rik	-3.47E+00	7.21E-135	down	-2.89E+00	2.31E-31	down
ENSMUSG00000030532	Hddc3	-1.68E+00	5.21E-04	down	-1.97E+00	3.15E-07	down
ENSMUSG00000032725	Folr2	3.98E+00	3.01E-08	up	1.70E+00	1.24E-01	no
ENSMUSG00000030842	Lamtor1	1.60E+00	4.31E-07	up	5.91E-01	1.20E-01	no
ENSMUSG00000073982	Rhog	2.52E+00	4.48E-04	up	7.08E-01	5.83E-01	no
ENSMUSG00000030678	Maz	-1.08E+00	1.43E-02	no	-2.50E+00	2.92E-18	down
ENSMUSG00000057101	Zfp180	-1.01E+00	4.18E-01	no	-3.07E+00	7.37E-05	down
ENSMUSG00000011632	Pinlyp	-2.58E+00	2.43E-05	down	-2.02E+00	3.05E-05	down
ENSMUSG00000003380	Rabac1	1.51E+00	5.17E-24	up	7.78E-01	2.21E-09	no
ENSMUSG00000030579	Tyrobp	6.26E+00	3.88E-45	up	5.69E+00	4.10E-08	up
ENSMUSG00000038973	Cldnd2	-1.85E+00	9.65E-22	down	6.54E-01	9.81E-02	no
ENSMUSG00000063089	Klk1b8	-3.23E+00	1.33E-09	down	-2.65E+00	1.50E-02	no
ENSMUSG00000059042	Klk1b9	-5.19E+00	5.72E-04	down	-5.32E+00	2.19E-01	no
ENSMUSG00000063177	Klk1b27	1.59E+00	5.63E-01	no	3.99E+00	6.69E-04	up
ENSMUSG00000066516	Klk1b21	3.26E+00	1.73E-01	no	4.26E+00	3.78E-06	up
ENSMUSG00000063713	Klk1b24	4.14E+00	2.37E-01	no	4.70E+00	1.70E-04	up
ENSMUSG00000062073	Gm10109	3.41E+00	1.60E-03	no	2.82E+00	4.04E-04	up
ENSMUSG00000038782	1700028J19Rik	1.00E+00	9.46E-40	up	1.53E+00	4.85E-154	up
ENSMUSG00000051113	Fam71e1	-4.70E+00	1.79E-240	down	-3.09E+00	1.65E-36	down
ENSMUSG00000003429	Rps11	1.59E+00	1.77E-128	up	5.91E-01	1.01E-23	no
ENSMUSG00000095276	Gm581	-3.87E+00	4.01E-18	down	-2.17E+00	2.70E-03	no
ENSMUSG00000013091	Tmem190	-5.77E-01	2.08E-07	no	1.63E+00	1.36E-17	up
ENSMUSG00000030647	Ndufc2	2.13E+00	2.39E-19	up	-4.03E-01	6.97E-03	no
ENSMUSG00000035227	Spcs2	-1.25E+00	4.72E-07	down	-7.23E-01	2.17E-02	no
ENSMUSG00000030654	Arl6ip1	1.14E+00	1.98E-07	up	-1.24E+00	1.64E-19	down
ENSMUSG00000030385	2900092C05Rik	-3.09E+00	5.35E-61	down	-1.01E+00	5.11E-03	no
ENSMUSG00000030682	Cdipt	-1.46E+00	9.40E-04	down	4.53E-01	6.75E-01	no
ENSMUSG00000049739	Zfp646	-4.58E+00	1.50E-40	down	-4.11E+00	2.08E-35	down
ENSMUSG00000096145	Vkorc1	4.62E+00	3.04E-17	up	2.49E+00	1.77E-04	up
ENSMUSG00000033916	Chmp2a	-4.47E-01	4.36E-04	no	-1.11E+00	2.45E-27	down
ENSMUSG00000060260	Pwwp2b	-5.84E+00	1.90E-92	down	-3.46E+00	1.57E-12	down
ENSMUSG00000062773	Tex101	5.21E+00	0.00E+00	up	2.64E+00	0.00E+00	up
ENSMUSG00000031493	Ggn	-3.19E+00	2.51E-79	down	-3.05E+00	6.96E-83	down

ENSMUSG00000006311	Etv2	-4.39E+00	4.74E-04	down	-3.84E+00	2.77E-01	no
ENSMUSG00000046826	Fam187b	-2.77E+00	5.41E-38	down	-6.13E-01	1.42E-01	no
ENSMUSG00000091474	2610021A01Rik	-1.90E+00	2.76E-01	no	-3.71E+00	9.82E-04	down
ENSMUSG00000066500	Izumo2	-3.53E+00	2.97E-116	down	-1.16E+00	3.18E-06	down
ENSMUSG00000038539	Atf5	-3.32E+00	2.55E-05	down	-2.77E+00	7.96E-02	no
ENSMUSG00000043858	Nup62	-1.25E+00	5.73E-10	down	-9.18E-01	3.55E-06	no
ENSMUSG00000003873	Bax	4.41E+00	6.80E-16	up	2.42E+00	4.08E-06	up
ENSMUSG00000086784	Isoc2a	-6.81E+00	4.14E-11	down	-8.39E+00	3.81E-40	down
ENSMUSG00000039745	Htatip2	-1.50E+00	5.40E-05	down	-2.03E+00	2.90E-16	down
ENSMUSG00000055652	Klhl25	-2.46E+00	1.08E-07	down	-1.87E+00	3.32E-05	down
ENSMUSG00000030641	4632434I11Rik	-3.91E+00	1.24E-22	down	-3.83E+00	1.14E-22	down
ENSMUSG00000070366	Ppapdc1a	3.54E+00	1.72E-03	no	2.47E+00	6.10E-07	up
ENSMUSG00000040564	Apoc1	3.70E+00	1.19E-06	up	3.82E+00	5.39E-11	up
ENSMUSG00000045587	BC049730	1.72E+00	3.72E-17	up	-2.53E-01	1.37E-02	no
ENSMUSG00000040857	Erf	-4.81E+00	6.37E-07	down	-3.69E+00	7.66E-02	no
ENSMUSG00000040424	Hipk4	-3.54E+00	2.14E-32	down	-6.63E-01	3.29E-01	no
ENSMUSG00000074224	4932431P20Rik	-4.10E+00	4.20E-10	down	-4.27E+00	3.35E-15	down
ENSMUSG00000006315	Tmem147	2.16E+00	8.70E-32	up	1.24E+00	2.49E-14	up
ENSMUSG00000030423	Pop4	-9.35E-01	5.78E-02	no	-2.57E+00	3.51E-15	down
ENSMUSG00000003420	Fcgrt	4.97E+00	1.10E-15	up	4.16E+00	2.70E-03	no
ENSMUSG00000055150	Zfp78	-2.97E+00	1.33E-04	down	-3.00E+00	2.36E-05	down
ENSMUSG00000070837	Aurkc	-5.56E+00	3.00E-04	down	-5.43E+00	1.47E-07	down
ENSMUSG00000063902	Gm7964	-3.38E-02	8.77E-01	no	-1.41E+00	6.74E-24	down
ENSMUSG00000052305	Hbb-b1	5.30E+00	8.74E-08	up	3.28E+00	1.86E-02	no
ENSMUSG00000030887	Pdzd9	-2.82E+00	1.76E-67	down	-2.66E+00	1.96E-75	down
ENSMUSG00000030717	Nupr1	6.09E+00	1.21E-10	up	6.86E+00	2.58E-02	no
ENSMUSG00000025491	Ifitm1	6.10E+00	5.56E-16	up	4.31E+00	7.93E-02	no
ENSMUSG00000038580	Sct	9.96E+00	3.88E-15	up	7.65E+00	1.11E-02	no
ENSMUSG00000048481	Mypop	-3.93E+00	6.90E-12	down	-3.71E+00	1.38E-13	down
ENSMUSG00000004961	Syt5	-4.60E+00	2.94E-05	down	-3.72E+00	2.41E-05	down
ENSMUSG00000025103	Btbd1	-1.85E+00	1.54E-21	down	-2.18E+00	2.21E-39	down
ENSMUSG00000030747	Dgat2	-2.31E+00	2.39E-49	down	-2.24E-02	9.66E-01	no
ENSMUSG00000036744	Olfr701	-4.91E+00	1.46E-08	down	-2.80E+00	2.42E-01	no
ENSMUSG00000030697	Ppp4c	-2.51E+00	8.71E-36	down	-2.57E+00	7.74E-39	down
ENSMUSG00000057176	Gm166	-2.31E+00	1.47E-25	down	-1.32E+00	1.94E-08	down
ENSMUSG00000052833	Sae1	7.03E-01	1.15E-02	no	-1.27E+00	1.13E-17	down
ENSMUSG00000011263	Exoc3l2	-2.03E+00	2.92E-02	no	-2.60E+00	5.55E-06	down
ENSMUSG00000055305	Zfp93	-3.96E+00	5.95E-03	no	-5.21E+00	1.08E-04	down
ENSMUSG00000045948	Mrps12	7.52E-01	1.58E-02	no	1.64E+00	9.61E-06	up
ENSMUSG00000037239	Spred3	-6.46E+00	3.66E-06	down	-3.82E+00	5.88E-02	no
ENSMUSG00000040435	Ppp1r15a	3.54E+00	3.42E-09	up	1.54E+00	5.97E-02	no
ENSMUSG00000061374	Fiz1	-1.70E+00	2.69E-04	down	-2.26E+00	7.38E-10	down
ENSMUSG00000061119	Prcp	-2.15E+00	4.27E-04	down	-1.82E+00	1.23E-01	no
ENSMUSG00000030895	Hpx	6.01E+00	1.88E-18	up	7.72E+00	4.14E-31	up
ENSMUSG00000030878	Cdr2	-2.79E+00	3.49E-13	down	-2.85E+00	1.94E-14	down
ENSMUSG00000030867	Plk1	-2.02E+00	9.23E-17	down	-1.41E+00	4.55E-07	down
ENSMUSG00000030722	Nfatc2ip	-2.88E+00	6.41E-12	down	-1.74E+00	2.80E-04	down
ENSMUSG00000030733	Sh2b1	-2.09E-01	7.54E-01	no	-1.54E+00	1.60E-05	down
ENSMUSG00000047721	Bola2	2.54E+00	1.61E-11	up	1.58E+00	1.32E-08	up
ENSMUSG00000054715	Zscan22	-2.92E+00	3.99E-04	down	-2.30E+00	7.94E-04	down
ENSMUSG00000030376	Slc8a2	-7.05E+00	1.41E-07	down	-6.15E+00	5.23E-10	down
ENSMUSG00000085601	Gm4969	3.90E+00	2.44E-04	up	1.41E+00	6.65E-01	no
ENSMUSG00000049643	2310022A10Rik	-9.00E-01	3.39E-01	no	-2.21E+00	7.09E-06	down
ENSMUSG00000053395	Cacng8	-4.33E+00	9.87E-05	down	-3.95E+00	1.32E-04	down
ENSMUSG00000004610	Etfb	-1.14E+00	3.11E-06	down	6.57E-01	1.94E-01	no
ENSMUSG00000094152	Slc6a16	-3.60E+00	2.23E-17	down	-3.45E+00	1.96E-15	down
ENSMUSG00000063511	Snrnp70	-1.57E+00	5.09E-13	down	-3.16E+00	2.57E-51	down
ENSMUSG00000040212	Emp3	2.65E+00	2.85E-22	up	-7.76E-01	1.84E-02	no
ENSMUSG00000045795	Whamm	-2.46E+00	6.33E-07	down	-3.16E+00	2.23E-13	down
ENSMUSG00000025439	Clns1a	-8.36E-01	9.64E-03	no	-1.25E+00	1.32E-06	down
ENSMUSG00000031016	Wee1	-2.15E+00	8.73E-12	down	-1.19E+00	1.04E-02	no
ENSMUSG00000042423	Fbrs	-1.25E+00	2.85E-03	no	-2.45E+00	9.02E-15	down
ENSMUSG00000030811	Fbxl19	-2.73E+00	1.26E-03	no	-3.42E+00	4.04E-06	down
ENSMUSG00000030801	Kat8	-1.87E+00	3.50E-21	down	-1.26E+00	2.94E-11	down

ENSMUSG00000030421	Uri1	-2.86E+00	2.56E-27	down	-6.46E-01	2.95E-01	no
ENSMUSG00000054161	Fam83e	-1.87E+00	1.82E-07	down	6.29E-01	3.97E-01	no
ENSMUSG00000030510	Cers3	-5.64E-01	2.77E-01	no	-2.46E+00	2.50E-27	down
ENSMUSG00000038797	Zscan2	-6.59E+00	7.26E-46	down	-6.21E+00	9.48E-34	down
ENSMUSG00000038570	Fam154b	1.70E+00	1.10E-05	up	2.37E+00	2.33E-09	up
ENSMUSG00000030987	Stim1	-2.01E+00	1.93E-08	down	-2.43E+00	3.19E-16	down
ENSMUSG00000031029	Eif3f	-1.51E+00	9.08E-09	down	-1.82E+00	3.98E-14	down
ENSMUSG00000030968	Pdilt	-4.15E+00	3.92E-126	down	-1.16E+00	3.50E-04	down
ENSMUSG00000074377	Sult2a4	4.05E+00	2.86E-03	no	4.46E+00	1.01E-04	up
ENSMUSG00000006310	Zbtb32	2.24E-01	4.68E-01	no	-1.12E+00	1.67E-13	down
ENSMUSG00000036634	Mag	-1.15E+00	1.12E-01	no	-1.98E+00	8.72E-06	down
ENSMUSG00000070570	Slc17a7	-3.48E+00	8.58E-05	down	-1.26E+00	5.54E-01	no
ENSMUSG00000055809	Dnaaf3	-3.46E+00	3.65E-33	down	-2.46E+00	7.72E-16	down
ENSMUSG00000041560	Gltscr2	-2.96E-01	2.09E-01	no	-1.47E+00	5.29E-20	down
ENSMUSG00000040866	Rsph6a	-2.42E+00	9.34E-50	down	-2.25E+00	3.47E-55	down
ENSMUSG00000051403	Ppp1r37	-3.50E+00	2.30E-07	down	-5.08E+00	2.42E-21	down
ENSMUSG00000037513	Samd4b	-3.34E+00	1.16E-25	down	-2.60E+00	5.84E-11	down
ENSMUSG00000074227	Spint2	2.31E+00	8.64E-33	up	8.42E-01	8.68E-11	no
ENSMUSG00000040231	Syngr4	6.29E-01	6.41E-13	no	1.37E+00	1.37E-102	up
ENSMUSG00000025326	Ube3a	-2.26E+00	2.34E-07	down	-2.64E+00	1.60E-10	down
ENSMUSG00000000605	Clcn4-2	-1.34E+00	6.93E-02	no	-2.80E+00	1.18E-07	down
ENSMUSG00000038503	Mesdc2	-5.07E-01	2.84E-01	no	-1.96E+00	2.81E-16	down
ENSMUSG00000030718	Ppme1	-1.60E+00	1.95E-07	down	-1.38E+00	7.84E-05	down
ENSMUSG00000048787	Dcun1d3	-3.33E+00	7.39E-08	down	-2.52E+00	1.58E-03	no
ENSMUSG00000042502	Cd2bp2	-1.04E+00	1.63E-01	no	-2.60E+00	4.09E-08	down
ENSMUSG00000025503	Taldo1	2.92E+00	1.19E-06	up	2.29E+00	1.03E-03	no
ENSMUSG00000030406	Gipr	-5.22E+00	3.89E-05	down	-3.60E+00	1.38E-01	no
ENSMUSG00000002981	Clptm1	-1.45E+00	1.58E-05	down	-1.87E+00	9.47E-09	down
ENSMUSG00000030591	Psmd8	-9.59E-01	1.32E-22	no	-1.11E+00	4.30E-40	down
ENSMUSG00000030619	Eed	-8.84E-01	7.35E-02	no	-1.76E+00	1.23E-05	down
ENSMUSG00000035165	Kcne3	-2.42E+00	7.37E-53	down	3.41E-01	3.83E-01	no
ENSMUSG00000058230	Grlf1	-2.45E+00	5.06E-06	down	-2.30E+00	1.65E-05	down
ENSMUSG00000030410	Dmwd	-1.43E+00	5.33E-04	down	-1.14E+00	2.34E-02	no
ENSMUSG00000003436	Dll3	1.83E+00	2.40E-05	up	1.84E+00	9.05E-08	up
ENSMUSG00000002771	Grin2d	-2.34E+00	1.11E-06	down	-1.92E+00	6.73E-07	down
ENSMUSG00000030549	Rhcg	-2.24E+00	1.03E-06	down	-2.39E+00	2.92E-12	down
ENSMUSG00000030629	Zfand6	-1.59E+00	4.56E-17	down	-2.05E-01	6.67E-01	no
ENSMUSG00000040298	Btbd16	-2.75E+00	7.71E-45	down	-1.48E+00	5.39E-10	down
ENSMUSG00000059119	Nap1l4	-3.32E-01	3.29E-01	no	-1.72E+00	2.59E-21	down
ENSMUSG00000078796	Zfp541	-3.80E+00	5.71E-46	down	-3.57E+00	6.70E-42	down
ENSMUSG00000058709	Egln2	-1.86E+00	3.13E-58	down	-1.58E+00	2.26E-52	down
ENSMUSG00000046750	BC089491	-2.43E+00	4.10E-77	down	-1.22E-01	7.25E-01	no
ENSMUSG00000030556	Lrrc28	-1.89E+00	4.33E-14	down	-9.05E-01	5.44E-03	no
ENSMUSG00000030922	Lyrm1	-1.53E+00	1.31E-06	down	-1.71E+00	1.49E-12	down
ENSMUSG00000030868	Dctn5	-4.27E-01	5.04E-01	no	-1.49E+00	9.13E-05	down
ENSMUSG00000030403	Vasp	-3.57E+00	1.95E-14	down	-2.47E+00	9.09E-04	down
ENSMUSG00000051768	Xrcc1	-1.32E+00	4.19E-07	down	-1.66E+00	2.04E-18	down
ENSMUSG00000030595	Nfkbib	-1.12E+00	5.79E-08	down	-4.55E-01	1.31E-01	no
ENSMUSG00000055102	Zfp819	-1.94E+00	1.50E-05	down	7.92E-01	4.16E-01	no
ENSMUSG00000050708	Ftl1	7.51E+00	0.00E+00	up	5.42E+00	7.77E-110	up
ENSMUSG00000064158	Izumo1	-4.36E+00	1.61E-40	down	-1.45E+00	2.23E-02	no
ENSMUSG00000039202	Abhd2	-4.14E+00	3.97E-12	down	-2.59E+00	6.85E-03	no
ENSMUSG00000016626	Nlrp14	-2.82E+00	6.35E-40	down	-3.01E+00	1.89E-60	down
ENSMUSG00000030877	4933427G17Rik	-3.10E+00	1.49E-35	down	-1.03E+00	1.01E-02	no
ENSMUSG00000073795	6430531B16Rik	-6.45E-01	6.51E-02	no	-1.28E+00	1.01E-11	down
ENSMUSG00000025474	Tubgcp2	-2.41E+00	9.58E-12	down	-2.30E+00	7.57E-12	down
ENSMUSG00000025482	Odf3	-1.95E+00	1.13E-34	down	6.38E-01	3.68E-02	no
ENSMUSG00000030491	Tdrd12	-2.92E-01	4.75E-01	no	-1.26E+00	2.18E-08	down
ENSMUSG00000030521	Mphosph10	-4.13E-01	6.13E-01	no	-2.38E+00	9.86E-07	down
ENSMUSG00000039236	Isg20	-2.04E+00	5.56E-16	down	4.91E-01	4.13E-01	no
ENSMUSG00000039099	Wdr93	-2.41E+00	1.09E-15	down	-2.64E+00	4.14E-30	down
ENSMUSG00000061787	Rps17	-7.59E-01	1.44E-01	no	-1.44E+00	2.44E-04	down
ENSMUSG00000011154	1700026D08Rik	-2.20E+00	1.66E-24	down	-1.73E+00	7.50E-19	down
ENSMUSG00000030978	Rrm1	-1.86E+00	2.32E-08	down	-2.36E+00	8.40E-15	down

ENSMUSG00000055319	Sec23ip	-3.52E+00	9.73E-37	down	-2.96E+00	2.75E-21	down
ENSMUSG00000008028	1700008O03Rik	-2.15E+00	2.30E-04	down	-1.61E+00	4.12E-03	no
ENSMUSG00000007783	Cpt1c	-2.59E+00	4.17E-17	down	-1.77E+00	1.76E-08	down
ENSMUSG00000057342	Sphk2	-2.16E+00	9.62E-03	no	-2.58E+00	7.31E-05	down
ENSMUSG00000030605	Mfge8	4.22E+00	2.35E-10	up	1.71E+00	2.27E-02	no
ENSMUSG00000035211	Xrra1	-2.97E+00	4.01E-98	down	-1.48E+00	7.29E-23	down
ENSMUSG00000030816	Rnf40	-3.75E+00	1.16E-39	down	-3.52E+00	1.62E-37	down
ENSMUSG00000038650	Rnh1	3.23E+00	3.15E-09	up	9.55E-01	1.46E-01	no
ENSMUSG00000031085	Gm498	-5.62E+00	1.07E-35	down	-5.03E+00	4.12E-08	down
ENSMUSG00000003378	Grik5	-2.44E+00	1.96E-06	down	-3.14E+00	2.13E-17	down
ENSMUSG00000070709	1700049G17Rik	-2.90E+00	1.16E-04	down	-8.02E-01	6.60E-01	no
ENSMUSG00000038194	Lhb	-2.72E+00	3.64E-13	down	-5.43E-01	4.63E-01	no
ENSMUSG00000002778	Kdelr1	-7.31E-01	2.99E-01	no	-2.12E+00	1.04E-05	down
ENSMUSG00000035401	2210018M11Rik	-1.33E+00	1.12E-01	no	-2.68E+00	4.59E-07	down
ENSMUSG00000070424	Art5	-3.12E+00	1.29E-24	down	-3.85E+00	6.46E-47	down
ENSMUSG00000030738	Eif3c	-1.02E+00	4.66E-17	down	-5.81E-01	5.21E-06	no
ENSMUSG00000042606	Hirip3	-3.20E+00	2.18E-21	down	-3.28E+00	4.70E-27	down
ENSMUSG00000037706	Cd81	4.45E+00	6.93E-21	up	2.80E+00	1.39E-06	up
ENSMUSG00000040466	Blvrb	4.78E+00	6.42E-12	up	3.09E+00	3.08E-02	no
ENSMUSG00000036882	Arhgap33	-2.85E+00	5.14E-08	down	-3.15E+00	1.30E-09	down
ENSMUSG00000025324	Atp10a	-4.69E+00	2.04E-15	down	-1.59E+00	2.60E-01	no
ENSMUSG00000039361	Picalm	-3.74E+00	1.19E-28	down	-3.04E+00	1.22E-10	down
ENSMUSG00000030744	Rps3	1.36E+00	3.23E-06	up	-9.36E-01	2.78E-05	no
ENSMUSG00000038296	Galnt18	3.07E+00	5.93E-04	up	3.28E+00	2.18E-07	up
ENSMUSG00000030754	Copb1	-1.48E+00	2.61E-04	down	-2.30E+00	3.56E-09	down
ENSMUSG00000030671	Pde3b	-2.69E+00	7.73E-06	down	-3.05E+00	6.38E-09	down
ENSMUSG00000030967	Zranb1	-2.62E+00	8.74E-14	down	-2.83E+00	8.12E-17	down
ENSMUSG00000053111	Fank1	-1.43E+00	4.24E-34	down	-1.15E+00	2.89E-28	down
ENSMUSG00000002957	Ap2a2	-2.05E+00	2.53E-08	down	-2.15E-01	8.14E-01	no
ENSMUSG00000025139	Tollip	-2.24E+00	6.23E-19	down	-2.85E+00	7.46E-42	down
ENSMUSG00000057177	Gsk3a	-4.47E+00	4.77E-14	down	-5.64E+00	1.07E-31	down
ENSMUSG00000035674	Ndufa3	2.99E+00	3.95E-57	up	2.17E+00	5.89E-76	up
ENSMUSG00000059891	Tsks	-3.95E+00	5.96E-171	down	-1.65E+00	6.79E-13	down
ENSMUSG00000030708	Dnajb13	-2.31E+00	1.11E-103	down	-6.34E-01	2.40E-05	no
ENSMUSG00000063550	Nup98	-5.43E+00	2.93E-41	down	-4.61E+00	7.40E-17	down
ENSMUSG00000025510	Cd151	-1.48E+00	2.25E-02	no	-2.94E+00	1.33E-09	down
ENSMUSG00000040725	Hnrnpul1	-4.18E+00	8.47E-49	down	-4.42E+00	4.03E-70	down
ENSMUSG00000078786	BC024978	-2.85E+00	1.78E-06	down	-1.55E+00	1.01E-01	no
ENSMUSG00000025133	Ints4	-2.58E+00	1.07E-07	down	-2.23E+00	1.71E-06	down
ENSMUSG00000058638	Zfp110	-3.83E+00	2.54E-32	down	-3.46E+00	3.50E-28	down
ENSMUSG00000036570	Fxyd1	4.45E+00	5.78E-21	up	4.34E+00	7.43E-08	up
ENSMUSG00000030499	Kctd15	3.80E+00	9.68E-05	up	5.40E+00	3.17E-04	up
ENSMUSG00000006335	Tfpt	-2.16E+00	6.17E-07	down	-2.08E+00	1.32E-07	down
ENSMUSG00000052296	Ppp6r1	-3.65E+00	7.36E-27	down	-2.89E+00	5.98E-11	down
ENSMUSG00000051811	Cox6b2	3.24E+00	0.00E+00	up	3.05E+00	0.00E+00	up
ENSMUSG00000030435	U2af2	-1.74E+00	5.74E-03	no	-4.33E+00	3.10E-23	down
ENSMUSG00000038886	Man2a2	-2.91E+00	3.07E-06	down	-3.73E+00	3.19E-19	down
ENSMUSG00000066232	Ipo7	-3.30E+00	8.77E-08	down	-3.78E+00	7.13E-07	down
ENSMUSG00000030946	Lhpp	4.18E+00	6.41E-05	up	3.01E+00	1.06E-03	no
ENSMUSG00000038292	Ccdc155	-1.59E+00	9.49E-11	down	-1.83E+00	8.08E-21	down
ENSMUSG00000038260	Trpm4	-5.31E+00	1.15E-10	down	-2.34E+00	1.76E-01	no
ENSMUSG00000078681	Tm2d3	3.47E+00	1.51E-10	up	6.47E-01	8.20E-02	no
ENSMUSG00000025500	1600016N20Rik	-3.62E+00	1.33E-59	down	-1.08E+00	2.65E-03	no
ENSMUSG00000003438	Timm50	1.98E+00	1.61E-04	up	3.71E-01	6.14E-01	no
ENSMUSG00000014402	Tsg101	-6.22E-02	9.24E-01	no	-1.63E+00	2.37E-05	down
ENSMUSG00000035228	Ccdc106	-1.58E+00	2.30E-02	no	-1.91E+00	2.94E-05	down
ENSMUSG00000030704	Rab6a	-6.99E-01	1.08E-01	no	-2.22E+00	2.88E-10	down
ENSMUSG00000030711	Sult1a1	4.03E+00	4.47E-05	up	1.41E+00	4.46E-01	no
ENSMUSG00000025486	Sirt3	-8.61E-01	5.81E-03	no	-1.13E+00	3.01E-07	down
ENSMUSG00000007891	Ctsd	3.80E+00	7.02E-24	up	2.38E+00	9.47E-07	up
ENSMUSG00000054385	Ceacam2	-4.41E+00	1.06E-08	down	-1.78E+00	3.34E-01	no
ENSMUSG00000030617	Ccdc83	-3.44E+00	2.73E-37	down	-2.94E+00	7.05E-32	down
ENSMUSG00000059263	Usp47	-2.08E+00	1.22E-35	down	-2.25E+00	3.32E-27	down
ENSMUSG00000030751	Psma1	1.25E+00	2.71E-04	up	-6.09E-01	4.46E-02	no

ENSMUSG00000040390	Map3k10	-3.22E+00	3.58E-05	down	-4.21E+00	1.07E-09	down
ENSMUSG00000061099	Gapdhs	-6.42E+00	0.00E+00	down	-3.77E+00	1.82E-60	down
ENSMUSG00000074141	Il4i1	-2.32E+00	1.82E-10	down	-1.98E+00	5.10E-12	down
ENSMUSG00000045639	Zfp629	-4.17E+00	1.81E-06	down	-2.31E+00	7.89E-02	no
ENSMUSG00000070357	E030019B06Rik	-2.61E+00	2.93E-09	down	-3.29E+00	4.58E-34	down
ENSMUSG00000040734	Ppp1r13l	-3.00E+00	7.48E-09	down	-2.86E+00	1.82E-12	down
ENSMUSG00000002043	Trappc6a	1.73E+00	6.41E-11	up	9.89E-01	5.60E-08	no
ENSMUSG00000049123	Catsperg2	-2.98E+00	4.24E-19	down	-3.27E+00	2.73E-40	down
ENSMUSG00000034867	Ankrd27	-3.08E+00	4.92E-03	no	-4.52E+00	4.26E-08	down
ENSMUSG00000063065	Mapk3	-3.59E+00	1.27E-45	down	-3.06E+00	2.62E-28	down
ENSMUSG00000012848	Rps5	1.49E+00	1.67E-32	up	3.45E-01	2.84E-03	no
ENSMUSG00000019539	Rcn3	4.58E+00	3.17E-10	up	3.03E+00	4.21E-02	no
ENSMUSG00000040189	Ccdc114	-1.96E+00	1.48E-02	no	-2.91E+00	4.93E-07	down
ENSMUSG00000030980	Knop1	-1.95E+00	1.36E-14	down	-6.13E-01	1.19E-01	no
ENSMUSG00000003123	Lipe	-5.78E+00	5.95E-43	down	-4.13E+00	1.98E-10	down
ENSMUSG00000002608	Ccdc97	-2.16E+00	3.15E-04	down	-3.28E+00	3.24E-14	down
ENSMUSG00000003190	Bcl2l12	-7.11E-02	9.19E-01	no	-2.36E+00	2.19E-26	down
ENSMUSG00000070568	1700039E15Rik	-2.94E+00	1.02E-70	down	-2.26E+00	5.09E-49	down
ENSMUSG00000039391	Ccdc81	-4.63E+00	2.50E-120	down	-1.54E+00	3.83E-05	down
ENSMUSG00000041328	Pcf11	-4.11E+00	1.72E-26	down	-3.59E+00	1.62E-14	down
ENSMUSG00000042246	Tmc7	-4.17E+00	1.77E-25	down	-8.87E-01	3.36E-01	no
ENSMUSG00000053964	Lgals4	-1.39E+00	5.02E-01	no	-4.59E+00	8.57E-04	down
ENSMUSG00000045467	Ttll13	-3.92E+00	4.71E-99	down	-2.68E+00	1.74E-34	down
ENSMUSG00000036427	Gpi1	-2.94E+00	5.84E-19	down	-1.20E+00	3.59E-02	no
ENSMUSG00000074165	Zfp788	-3.37E+00	4.01E-03	no	-4.25E+00	3.16E-05	down
ENSMUSG00000012777	Acpt	-3.79E+00	2.90E-10	down	-6.75E-01	6.56E-01	no
ENSMUSG00000092518	Fam71e2	-4.25E+00	2.88E-136	down	-6.34E-01	8.62E-02	no
ENSMUSG00000025511	Tspan4	4.59E+00	2.49E-07	up	2.61E+00	4.58E-02	no
ENSMUSG00000053898	Ech1	-8.99E-01	7.37E-03	no	-1.00E+00	2.74E-04	down
ENSMUSG00000040331	Nsmce4a	9.46E-01	1.72E-01	no	-2.14E+00	3.32E-08	down
ENSMUSG00000006333	Rps9	-1.32E-01	6.32E-01	no	-1.17E+00	5.01E-09	down
ENSMUSG00000035458	Tnni3	-2.46E+00	3.31E-10	down	-1.71E+00	2.56E-04	down
ENSMUSG00000035203	Epn1	-4.58E+00	1.95E-13	down	-4.78E+00	3.01E-12	down
ENSMUSG00000035582	Gdpd4	-3.07E+00	8.34E-06	down	-3.77E+00	3.14E-16	down
ENSMUSG00000050605	Zfp61	-2.03E+00	4.97E-02	no	-2.83E+00	1.39E-05	down
ENSMUSG00000035585	Tsen34	3.91E+00	2.21E-05	up	1.68E+00	1.55E-01	no
ENSMUSG00000030771	Micalcl	-4.17E+00	1.43E-50	down	-1.45E+00	7.55E-03	no
ENSMUSG00000032777	Gtf3c1	-2.03E+00	1.48E-04	down	-2.89E+00	2.37E-14	down
ENSMUSG00000030714	Ccdc101	-1.43E+00	2.46E-24	down	7.14E-01	8.80E-03	no
ENSMUSG00000002985	Apoe	5.31E+00	0.00E+00	up	3.91E+00	1.34E-40	up
ENSMUSG00000019738	Polr2i	1.09E+00	9.30E-11	up	1.01E+00	1.22E-13	up
ENSMUSG00000030471	Zdhhc13	4.38E-01	7.33E-01	no	-2.66E+00	3.09E-04	down
ENSMUSG00000042675	Ypel3	1.03E+00	9.96E-04	up	2.29E+00	1.00E-04	up
ENSMUSG00000030541	Idh2	4.30E+00	5.07E-04	up	2.04E+00	3.33E-01	no
ENSMUSG00000030536	Iqgap1	-3.32E+00	6.01E-15	down	-1.94E+00	4.05E-04	down
ENSMUSG00000030884	Uqcrc2	-5.10E-01	1.24E-01	no	-1.72E+00	2.93E-18	down
ENSMUSG00000005575	Ube2m	-3.29E+00	1.82E-42	down	-3.49E+00	6.81E-66	down
ENSMUSG00000040136	Abcc8	6.01E+00	4.12E-06	up	5.90E+00	7.08E-07	up
ENSMUSG00000025584	Pde8a	-3.38E+00	5.75E-34	down	-3.21E+00	3.02E-48	down
ENSMUSG00000038187	Btbd10	-2.22E+00	1.16E-11	down	-1.45E+00	8.22E-04	down
ENSMUSG00000059981	Taok2	-2.21E+00	3.37E-02	no	-4.56E+00	7.48E-13	down
ENSMUSG00000030994	D7Ertd443e	-5.40E+00	2.99E-28	down	-2.91E+00	1.16E-03	no
ENSMUSG00000030835	Nomo1	-2.10E+00	2.69E-05	down	-2.69E+00	2.16E-10	down
ENSMUSG00000030880	Polr3e	-1.48E+00	4.86E-03	no	-1.94E+00	1.11E-05	down
ENSMUSG00000035390	Brsk1	-7.39E+00	4.95E-10	down	-8.58E+00	7.20E-06	down
ENSMUSG00000013353	4931406B18Rik	-3.12E+00	5.67E-03	no	-3.47E+00	1.78E-05	down
ENSMUSG00000063229	Ldha	-1.17E+00	2.90E-46	down	-1.01E+00	9.43E-51	down
ENSMUSG00000060538	Tmem219	-9.52E-01	9.56E-02	no	-2.36E+00	1.08E-09	down
ENSMUSG00000005566	Trim28	-1.60E+00	2.04E-21	down	-1.97E+00	1.53E-48	down
ENSMUSG00000002210	Smg9	-2.46E+00	5.11E-10	down	-3.55E+00	3.04E-60	down
ENSMUSG00000030726	Pold3	-2.37E+00	5.35E-20	down	-1.71E+00	1.97E-08	down
ENSMUSG00000030788	Rnf141	-2.27E+00	2.68E-03	no	-2.63E+00	3.95E-04	down
ENSMUSG00000040952	Rps19	3.16E+00	3.06E-14	up	1.58E+00	3.29E-04	up
ENSMUSG00000002409	Dyrk1b	-3.63E+00	2.40E-68	down	-2.12E+00	2.88E-18	down

ENSMUSG00000030528	Blm	-2.60E+00	3.27E-03	no	-3.23E+00	1.72E-05	down
ENSMUSG00000047554	Tmem41b	-1.65E+00	1.81E-02	no	-2.60E+00	1.22E-05	down
ENSMUSG00000015980	Lrrc27	-2.83E+00	7.64E-105	down	-1.51E+00	7.08E-31	down
ENSMUSG00000003865	Gys1	7.43E+00	4.35E-08	up	4.45E+00	3.60E-02	no
ENSMUSG00000064307	Lrrc51	-1.09E+00	4.59E-47	down	1.01E-01	4.37E-01	no
ENSMUSG00000030689	Ino80e	-1.61E+00	6.95E-03	no	-2.52E+00	1.50E-10	down
ENSMUSG00000052997	Uba2	-1.06E+00	2.94E-03	no	-2.44E+00	3.71E-18	down
ENSMUSG00000038637	Lrrc56	-2.51E+00	1.27E-10	down	-2.24E+00	5.60E-12	down
ENSMUSG00000000154	Slc22a18	-2.09E+00	4.74E-03	no	-2.57E+00	2.48E-05	down
ENSMUSG00000003184	Irf3	1.85E-01	7.81E-01	no	-1.80E+00	2.32E-09	down
ENSMUSG00000041769	Ppp2r2d	-1.85E+00	1.74E-13	down	-1.23E+00	2.74E-04	down
ENSMUSG00000038406	Scaf1	-2.21E+00	8.10E-02	no	-3.47E+00	1.87E-04	down
ENSMUSG00000030822	Prr14	-1.27E+00	1.82E-02	no	-1.81E+00	3.18E-06	down
ENSMUSG00000001794	Capns1	-1.49E+00	3.53E-21	down	-1.33E+00	2.02E-19	down
ENSMUSG00000033904	Ccp110	-1.22E+00	3.47E-02	no	-3.29E+00	2.62E-33	down
ENSMUSG00000030779	Rbbp6	-2.86E+00	7.47E-21	down	-1.81E+00	9.41E-06	down
ENSMUSG00000030688	Stard10	-2.91E+00	1.13E-107	down	-6.82E-01	2.51E-03	no
ENSMUSG00000025509	Pnpla2	-1.70E+00	6.82E-27	down	-1.37E+00	3.55E-19	down
ENSMUSG00000031027	Stk33	-2.56E+00	1.79E-67	down	-2.07E+00	8.44E-57	down
ENSMUSG00000005609	Ctr9	-2.18E+00	3.72E-06	down	-2.44E+00	2.10E-07	down
ENSMUSG00000030815	Phkg2	-4.56E+00	1.01E-121	down	-3.76E+00	1.65E-81	down
ENSMUSG00000003437	Paf1	-1.64E+00	4.58E-08	down	-1.39E+00	1.74E-04	down
ENSMUSG00000006599	Gtf2h1	-1.35E+00	8.87E-08	down	-2.45E+00	7.80E-42	down
ENSMUSG00000041343	Ankrd42	-3.83E+00	2.35E-57	down	-2.87E+00	2.00E-27	down
ENSMUSG00000040714	Klc3	-3.29E+00	7.72E-04	down	-2.23E+00	3.80E-01	no
ENSMUSG00000032737	Inppl1	-2.69E+00	1.59E-02	no	-3.58E+00	6.48E-05	down
ENSMUSG00000062017	Abca14	-4.43E+00	2.12E-15	down	-3.81E+00	9.54E-20	down
ENSMUSG00000030374	Strn4	-1.30E+00	1.56E-03	no	-1.50E+00	2.90E-05	down
ENSMUSG00000003099	Ppp5c	3.86E-01	5.23E-01	no	-1.43E+00	3.65E-07	down
ENSMUSG00000030638	Sh3gl3	-2.01E+00	8.70E-08	down	-2.08E+00	1.39E-12	down
ENSMUSG00000025495	Ptdss2	-1.91E+00	6.88E-20	down	-9.36E-01	2.55E-05	no
ENSMUSG00000052273	Dnahc3	-4.01E+00	8.72E-13	down	-9.50E-01	4.94E-01	no
ENSMUSG00000030965	Fam175b	-2.93E+00	1.11E-05	down	-2.39E+00	1.77E-03	no
ENSMUSG00000025477	Inpp5a	-2.95E+00	1.11E-14	down	-2.36E+00	3.11E-09	down
ENSMUSG00000030516	Tjp1	-3.44E+00	3.74E-07	down	-3.07E+00	1.00E-03	down
ENSMUSG00000030590	Fam98c	-2.38E+00	1.98E-14	down	-2.49E+00	1.22E-22	down
ENSMUSG00000002205	Vrk3	-1.95E+00	4.00E-12	down	-1.82E+00	1.28E-14	down
ENSMUSG00000031026	Trim66	-4.31E+00	2.26E-04	down	-1.55E+00	5.39E-01	no
ENSMUSG00000023072	Cep89	-1.84E+00	2.44E-09	down	-1.46E+00	1.05E-05	down
ENSMUSG00000038563	Eftud1	-2.32E+00	1.31E-08	down	-6.32E-01	4.12E-01	no
ENSMUSG00000074129	Rpl13a	1.03E+00	4.41E-09	up	-8.22E-01	3.09E-07	no
ENSMUSG00000030759	Far1	-1.74E+00	2.00E-02	no	-2.62E+00	2.35E-05	down
ENSMUSG00000032637	Atxn2l	-3.46E+00	1.26E-92	down	-2.63E+00	2.78E-67	down
ENSMUSG00000030924	2610020H08Rik	-1.20E+00	1.12E-04	down	-2.18E+00	1.32E-15	down
ENSMUSG00000032743	D430042O09Rik	-3.73E+00	3.61E-10	down	-3.98E+00	2.61E-13	down
ENSMUSG00000051900	Abca16	-4.23E+00	9.33E-13	down	-3.71E+00	7.78E-14	down
ENSMUSG00000041375	Ccdc9	-2.13E+00	4.47E-02	no	-3.76E+00	2.81E-07	down
ENSMUSG00000054746	Abca15	-3.60E+00	1.07E-07	down	-3.70E+00	1.81E-10	down
ENSMUSG00000030882	Dnhd1	-3.33E+00	7.75E-06	down	-3.83E+00	5.77E-16	down
ENSMUSG00000038611	Phrf1	-1.86E+00	2.90E-03	no	-2.52E+00	1.42E-06	down
ENSMUSG00000003269	Cyth2	-1.42E+00	6.98E-09	down	-1.53E+00	1.64E-12	down
ENSMUSG00000030555	Ttc23	-3.19E+00	2.07E-05	down	-2.30E+00	4.01E-04	down
ENSMUSG00000060601	Nr1h2	-9.93E-01	1.38E-01	no	-2.14E+00	1.32E-05	down
ENSMUSG00000001829	Clpb	-3.38E+00	1.05E-42	down	-1.09E+00	1.37E-02	no
ENSMUSG00000051910	Sox6	-3.52E+00	1.02E-09	down	-1.58E+00	2.97E-02	no
ENSMUSG00000035545	Leng8	-2.94E+00	3.56E-12	down	-4.39E+00	6.01E-31	down
ENSMUSG00000019254	Ppp1r12c	-1.41E+00	1.59E-02	no	-2.61E+00	3.51E-10	down
ENSMUSG00000034990	Otoa	5.99E+00	5.47E-05	up	6.38E+00	7.59E-05	up
ENSMUSG00000023118	Sympk	-4.35E+00	2.47E-21	down	-3.59E+00	1.93E-12	down
ENSMUSG00000007279	Scube2	-4.04E+00	4.90E-06	down	-1.10E+00	6.32E-01	no
ENSMUSG00000015149	Sirt2	-1.54E+00	4.91E-09	down	-1.38E+00	7.74E-09	down
ENSMUSG00000030650	Tmc5	-4.61E+00	2.03E-27	down	-1.45E+00	8.20E-02	no
ENSMUSG00000009687	Fxyd5	2.56E+00	5.88E-04	up	1.17E-01	9.44E-01	no
ENSMUSG00000030795	Fus	-3.05E+00	2.63E-09	down	-3.87E+00	4.36E-14	down

ENSMUSG00000023467	Tulp2	-3.22E+00	7.84E-189	down	-9.49E-01	1.04E-07	no
ENSMUSG00000025487	Psmd13	-1.46E+00	6.09E-07	down	-1.86E+00	3.69E-15	down
ENSMUSG00000040811	Eml2	-4.25E+00	6.17E-04	down	-1.87E+00	5.65E-01	no
ENSMUSG00000070369	Itgad	-3.34E+00	1.57E-04	down	-1.97E+00	1.53E-01	no
ENSMUSG00000015165	Hnrnpl	-1.22E+00	4.79E-02	no	-3.90E+00	1.14E-35	down
ENSMUSG00000030846	Tial1	-2.21E+00	4.73E-03	no	-4.15E+00	3.91E-16	down
ENSMUSG00000037020	Wdr62	-3.14E+00	7.34E-50	down	-2.65E+00	2.27E-41	down
ENSMUSG00000005610	Eif4g2	-2.83E+00	5.21E-30	down	-3.07E+00	3.91E-32	down
ENSMUSG00000030970	Ctbp2	-3.46E+00	3.14E-04	down	-3.52E+00	7.74E-04	down
ENSMUSG00000030400	Ercc2	-9.84E-01	3.35E-01	no	-2.68E+00	5.47E-07	down
ENSMUSG00000038943	Prc1	-4.69E-01	4.54E-01	no	-1.61E+00	6.57E-06	down
ENSMUSG00000005442	Cic	-1.48E+00	1.10E-01	no	-2.48E+00	9.86E-04	down
ENSMUSG00000039176	Polg	-2.16E+00	2.71E-05	down	-2.44E+00	3.28E-08	down
ENSMUSG00000004056	Akt2	-1.01E+00	2.95E-01	no	-2.22E+00	6.21E-04	down
ENSMUSG00000060279	Ap2a1	-3.99E+00	5.42E-40	down	-2.66E+00	5.66E-11	down
ENSMUSG00000030990	Pgap2	-2.19E+00	1.03E-04	down	-8.14E-01	4.43E-01	no
ENSMUSG00000042055	Wdr11	-2.07E+00	4.25E-02	no	-2.93E+00	1.02E-04	down
ENSMUSG00000006307	Wbp7	-7.50E-01	4.36E-01	no	-2.96E+00	3.46E-07	down
ENSMUSG00000030889	Vwa3a	-4.96E+00	3.28E-16	down	-2.34E+00	7.39E-02	no
ENSMUSG00000011751	Sptbn4	-3.71E+00	6.87E-05	down	-3.47E+00	1.35E-07	down
ENSMUSG00000014418	Hps5	-2.85E+00	5.95E-03	no	-3.72E+00	3.08E-05	down
ENSMUSG00000030655	Smg1	-1.93E+00	1.82E-02	no	-3.32E+00	1.85E-05	down
ENSMUSG00000000131	Xpo6	-3.33E+00	5.26E-18	down	-2.99E+00	5.21E-14	down
ENSMUSG00000040488	Ltbp4	3.36E+00	6.43E-08	up	2.23E+00	2.49E-01	no
ENSMUSG00000047248	C2cd3	-3.32E+00	2.21E-05	down	-3.00E+00	1.98E-04	down
ENSMUSG00000070425	Trpc2	-2.46E+00	8.95E-04	down	-1.57E+00	1.12E-01	no
ENSMUSG00000030451	Herc2	-2.18E+00	5.38E-04	down	-2.10E+00	4.54E-03	no
ENSMUSG00000038371	Sbf2	-2.41E+00	3.94E-04	down	-2.92E+00	1.81E-07	down
ENSMUSG00000049676	Catsperg1	-3.41E+00	7.80E-16	down	-3.70E+00	5.12E-27	down
ENSMUSG00000053046	Brsk2	-1.80E+00	5.26E-04	down	-2.87E-01	8.25E-01	no
ENSMUSG00000043192	Gm1840	-3.15E+00	1.18E-48	down	-1.58E+00	1.65E-06	down
ENSMUSG00000057116	AF366264	-6.19E+00	5.89E-18	down	-4.01E+00	5.37E-03	no
ENSMUSG00000074384	AI429214	-4.26E+00	1.58E-17	down	-4.45E+00	9.54E-27	down
ENSMUSG00000050190	Gm5346	-4.20E+00	6.84E-05	down	-2.21E+00	4.85E-01	no
ENSMUSG00000047654	Tssk6	-4.22E+00	2.97E-47	down	-2.90E+00	2.49E-10	down
ENSMUSG00000071076	Jund	1.36E+00	6.67E-05	up	1.57E+00	1.67E-02	no
ENSMUSG00000070713	Gm10282	4.08E+00	2.19E-11	up	1.97E+00	2.68E-02	no
ENSMUSG00000074215	Gm10643	-3.39E+00	5.45E-04	down	-2.33E+00	3.12E-01	no
ENSMUSG00000053560	Ier2	4.92E+00	5.71E-27	up	1.34E+00	7.49E-02	no
ENSMUSG00000052837	Junb	3.57E+00	1.55E-26	up	1.37E+00	2.74E-02	no
ENSMUSG00000074140	Zfp319	-4.61E+00	3.58E-05	down	-2.48E+00	2.37E-01	no
ENSMUSG00000061104	Gm10094	1.36E+00	1.74E-27	up	4.14E-01	1.05E-04	no
ENSMUSG00000045538	Ddx28	1.14E+00	4.22E-01	no	-2.41E+00	2.95E-05	down
ENSMUSG00000060019	Gm10073	3.86E+00	0.00E+00	up	2.44E+00	3.79E-245	up
ENSMUSG00000057657	Rps18-ps3	-7.12E-01	3.76E-02	no	-1.27E+00	5.82E-06	down
ENSMUSG00000057387	4922502B01Rik	-3.50E+00	5.26E-02	no	-5.45E+00	4.95E-04	down
ENSMUSG00000096262	Ap1g1	-6.11E-01	5.80E-01	no	-2.98E+00	3.80E-06	down
ENSMUSG00000074030	Exoc8	-1.12E+00	3.04E-01	no	-2.30E+00	6.91E-05	down
ENSMUSG00000050930	Map10	-2.83E+00	5.65E-08	down	-2.40E+00	2.23E-07	down
ENSMUSG00000038894	Irs2	3.86E+00	1.18E-04	up	2.19E+00	9.75E-02	no
ENSMUSG00000031504	Rab20	2.03E+00	3.15E-04	up	6.08E-01	4.25E-01	no
ENSMUSG00000068631	Gm7676	2.46E+00	2.11E-09	up	4.75E-01	2.95E-01	no
ENSMUSG00000046354	Defb14	-2.31E+00	1.48E-09	down	-1.19E+00	8.57E-03	no
ENSMUSG00000049476	1700104B16Rik	-1.35E+00	5.66E-12	down	-1.04E+00	1.02E-07	down
ENSMUSG00000046723	Adam24	-5.10E+00	6.99E-63	down	-1.81E+00	2.38E-03	no
ENSMUSG00000071937	Adam25	-4.37E+00	2.49E-24	down	-1.67E+00	7.58E-02	no
ENSMUSG00000046282	Adam20	-4.37E+00	1.60E-35	down	-1.55E+00	4.61E-02	no
ENSMUSG00000054033	Adam39	-3.89E+00	5.84E-26	down	-1.02E+00	2.42E-01	no
ENSMUSG00000063900	Adam26b	-2.71E+00	2.85E-10	down	-3.00E-01	8.19E-01	no
ENSMUSG00000048516	Adam26a	-4.24E+00	2.30E-39	down	-1.60E+00	2.61E-02	no
ENSMUSG00000079058	Adam34	-4.96E+00	4.44E-15	down	-2.60E+00	6.49E-02	no
ENSMUSG00000046258	Adam29	-3.74E+00	4.79E-24	down	-9.82E-01	2.68E-01	no
ENSMUSG00000079019	Insl3	7.14E+00	2.18E-36	up	8.14E+00	3.50E-186	up
ENSMUSG00000037070	Rbmxl1	2.65E-01	7.39E-01	no	-1.91E+00	5.87E-05	down

ENSMUSG00000056155	Nanos3	3.81E+00	2.29E-09	up	2.78E+00	6.54E-08	up
ENSMUSG00000053226	Dand5	-1.66E+00	2.46E-07	down	-1.05E+00	1.30E-03	no
ENSMUSG00000040467	4921522P10Rik	-4.40E+00	7.01E-16	down	-3.37E+00	5.29E-08	down
ENSMUSG00000036840	Siah1a	-7.18E-01	3.54E-01	no	-2.90E+00	5.74E-13	down
ENSMUSG00000038917	3930402G23Rik	6.09E+00	1.15E-06	up	8.70E+00	4.58E-04	up
ENSMUSG00000031953	Tmem170	-1.05E+00	3.10E-01	no	-1.99E+00	8.55E-04	down
ENSMUSG00000033430	Terf2ip	3.46E+00	2.41E-05	up	2.54E-01	8.59E-01	no
ENSMUSG00000031847	1700030J22Rik	-3.64E+00	2.49E-88	down	-1.56E+00	2.87E-09	down
ENSMUSG00000048478	4732415M23Rik	7.94E-01	1.87E-132	no	1.21E+00	0.00E+00	up
ENSMUSG00000074454	Defb33	-1.62E+00	6.27E-08	down	-9.42E-01	1.56E-01	no
ENSMUSG00000031490	Eif4ebp1	1.63E+00	1.90E-06	up	3.09E+00	2.44E-07	up
ENSMUSG00000071138	Tex24	-3.52E+00	8.42E-29	down	-3.62E-01	6.88E-01	no
ENSMUSG00000038069	Cdkn2aip	-3.09E+00	8.37E-30	down	-1.88E+00	6.09E-07	down
ENSMUSG00000025519	Tktl2	-2.72E+00	1.93E-05	down	-2.97E+00	1.97E-09	down
ENSMUSG00000045248	Med26	-2.14E+00	8.04E-14	down	-2.41E+00	6.97E-21	down
ENSMUSG00000005483	Dnajb1	-1.29E+00	3.16E-36	down	-1.15E+00	3.17E-30	down
ENSMUSG00000019362	D8Ertd738e	1.41E+00	3.49E-26	up	9.62E-01	6.09E-17	no
ENSMUSG00000031762	Mt2	3.86E+00	0.00E+00	up	2.96E+00	0.00E+00	up
ENSMUSG00000031765	Mt1	4.38E+00	0.00E+00	up	4.41E+00	0.00E+00	up
ENSMUSG00000013158	4933405L10Rik	-2.46E+00	9.64E-23	down	-1.81E+00	2.87E-14	down
ENSMUSG00000069895	Atxn1l	-4.06E+00	1.23E-08	down	-4.05E+00	6.60E-07	down
ENSMUSG00000047388	Atmin	-2.19E+00	2.46E-05	down	-1.82E+00	6.14E-04	down
ENSMUSG00000031839	Hsbp1	1.88E+00	0.00E+00	up	1.74E+00	0.00E+00	up
ENSMUSG00000031458	Coprs	2.88E+00	2.12E-91	up	1.06E+00	5.83E-46	up
ENSMUSG00000017049	Ccdc70	-3.69E+00	5.69E-150	down	-1.40E+00	1.40E-09	down
ENSMUSG00000031487	Brf2	-2.63E+00	2.40E-07	down	-2.30E+00	2.70E-06	down
ENSMUSG00000031530	Dusp4	-3.04E+00	6.19E-06	down	-5.69E-01	7.72E-01	no
ENSMUSG00000031609	Sap30	7.59E-01	1.99E-01	no	-2.27E+00	1.31E-25	down
ENSMUSG00000036934	4921524J17Rik	1.44E+00	7.34E-05	up	-7.05E-02	8.89E-01	no
ENSMUSG00000040459	Arglu1	-1.12E+00	3.02E-03	no	-3.01E+00	4.50E-23	down
ENSMUSG00000049717	Lig4	-4.24E+00	1.14E-05	down	-4.21E+00	5.47E-07	down
ENSMUSG00000041679	Lrrc29	-2.00E+00	3.68E-09	down	-1.72E+00	1.87E-09	down
ENSMUSG00000005705	Agrp	1.30E-01	9.53E-01	no	-3.58E+00	6.94E-04	down
ENSMUSG00000033106	Slc7a6os	-8.85E-01	9.32E-02	no	-2.22E+00	2.41E-10	down
ENSMUSG00000031924	Cyb5b	-2.20E-01	8.09E-01	no	-1.93E+00	1.67E-05	down
ENSMUSG00000031844	Hsd17b2	5.07E+00	1.82E-51	up	5.51E+00	1.57E-80	up
ENSMUSG00000006589	Aprt	1.07E+00	6.14E-29	up	8.55E-01	4.23E-39	no
ENSMUSG00000015013	Trappc2l	2.06E+00	1.63E-62	up	1.99E+00	3.10E-70	up
ENSMUSG00000033594	Spata2l	-3.32E+00	3.56E-08	down	-5.10E-01	7.68E-01	no
ENSMUSG00000031980	Agt	4.60E+00	4.83E-08	up	4.23E+00	2.19E-09	up
ENSMUSG00000031986	Sprtn	-2.21E+00	4.98E-11	down	-3.09E+00	1.32E-63	down
ENSMUSG00000049008	BB014433	-2.18E+00	6.62E-49	down	1.64E-02	9.74E-01	no
ENSMUSG00000054408	Spcs3	-2.37E-01	8.12E-01	no	-3.13E+00	1.74E-09	down
ENSMUSG00000054717	Hmgb2	3.98E+00	2.30E-23	up	2.70E-01	4.36E-01	no
ENSMUSG00000036199	Ndufa13	1.79E+00	2.53E-54	up	2.03E+00	4.40E-84	up
ENSMUSG00000038508	Gdf15	6.62E+00	1.54E-14	up	5.77E+00	9.92E-03	no
ENSMUSG00000035559	Mpv17l2	4.36E+00	1.89E-05	up	2.12E+00	1.57E-02	no
ENSMUSG00000007721	Ccdc124	1.07E+00	4.78E-15	up	5.98E-01	1.85E-07	no
ENSMUSG00000045128	Rpl18a	1.49E+00	1.41E-08	up	3.35E-01	2.47E-01	no
ENSMUSG00000007950	Abhd8	-1.39E+00	3.06E-14	down	-1.26E+00	4.92E-20	down
ENSMUSG00000046718	Bst2	3.64E+00	1.96E-07	up	3.31E+00	4.16E-04	up
ENSMUSG00000033938	Ndufb7	2.99E+00	1.58E-33	up	1.93E+00	8.78E-24	up
ENSMUSG00000074219	Gm10644	-1.76E+00	1.49E-02	no	-2.17E+00	2.76E-05	down
ENSMUSG00000005699	Pard6a	-1.22E+00	8.59E-05	down	-1.15E+00	7.75E-05	down
ENSMUSG00000031509	1700016D06Rik	-2.84E+00	7.38E-47	down	-5.98E-01	1.51E-01	no
ENSMUSG00000006519	Cyba	5.77E+00	3.04E-36	up	5.09E+00	3.60E-07	up
ENSMUSG00000056820	Tsnax	-1.14E+00	6.22E-03	no	-2.23E+00	9.40E-14	down
ENSMUSG00000031532	Tmem66	-1.47E-01	6.20E-01	no	-1.17E+00	3.71E-07	down
ENSMUSG00000040028	Elavl1	-1.21E+00	2.41E-02	no	-1.72E+00	1.47E-04	down
ENSMUSG00000031651	Triml1	-3.39E+00	8.71E-34	down	-6.77E-02	9.43E-01	no
ENSMUSG00000063049	Ing2	-1.62E+00	3.41E-03	no	-2.04E+00	5.38E-05	down
ENSMUSG00000023073	Slc10a2	2.10E+00	1.63E-04	up	4.11E+00	8.76E-05	up
ENSMUSG00000031518	Spata4	-2.17E+00	0.00E+00	down	-2.03E+00	0.00E+00	down
ENSMUSG00000031682	1700011L22Rik	-1.75E+00	3.32E-62	down	4.18E-01	4.59E-02	no

ENSMUSG00000069971	4933402J07Rik	-4.07E+00	7.91E-174	down	-1.83E+00	1.55E-11	down
ENSMUSG00000031796	Gtl3	-7.39E-01	5.22E-06	no	-1.03E+00	1.95E-16	down
ENSMUSG00000013155	Enkd1	-9.62E-01	3.58E-07	no	-1.25E+00	9.05E-19	down
ENSMUSG00000031951	Tmem231	-1.86E+00	2.08E-02	no	-2.63E+00	3.01E-06	down
ENSMUSG00000045246	Kcng4	-2.57E+00	2.53E-03	no	-2.47E+00	1.83E-05	down
ENSMUSG00000023336	Wfdc1	4.12E+00	2.46E-06	up	1.97E+00	1.54E-02	no
ENSMUSG00000031971	Ccsap	-4.16E+00	2.62E-14	down	-3.23E+00	1.68E-03	no
ENSMUSG00000031984	2810004N23Rik	1.64E-01	7.52E-01	no	-1.14E+00	2.76E-04	down
ENSMUSG00000010435	Spaca7	-2.54E+00	1.24E-139	down	-2.43E-01	3.16E-01	no
ENSMUSG00000078840	Gm10999	-5.41E+00	2.02E-22	down	-2.87E+00	8.86E-02	no
ENSMUSG00000031533	Mrps31	-2.24E+00	2.27E-07	down	-2.87E+00	1.24E-19	down
ENSMUSG00000009630	Ppp2cb	-3.48E+00	9.25E-46	down	-3.34E+00	5.04E-43	down
ENSMUSG00000031636	Pdlim3	4.25E+00	9.15E-04	up	2.03E+00	3.85E-01	no
ENSMUSG00000031633	Slc25a4	3.69E+00	5.35E-05	up	1.38E+00	2.15E-01	no
ENSMUSG00000052456	Asna1	-3.30E+00	9.34E-52	down	-3.03E+00	3.37E-43	down
ENSMUSG00000031897	Psmb10	3.97E+00	1.07E-05	up	1.84E+00	2.30E-02	no
ENSMUSG00000031723	Txnl4b	-3.78E+00	9.31E-31	down	-3.39E+00	6.94E-32	down
ENSMUSG00000034105	Tldc1	-1.34E+00	4.49E-01	no	-2.91E+00	7.95E-04	down
ENSMUSG00000050052	2610019F03Rik	-2.44E+00	1.58E-03	no	-2.65E+00	4.44E-09	down
ENSMUSG00000052906	Ubxn8	-2.79E+00	6.76E-10	down	-1.61E+00	1.02E-02	no
ENSMUSG00000096914	Galntl6	-2.97E+00	3.77E-21	down	-3.01E+00	2.08E-23	down
ENSMUSG00000035964	Tmem59l	-1.04E+00	2.13E-02	no	-2.21E+00	1.54E-09	down
ENSMUSG00000031838	Ifi30	5.77E+00	1.22E-24	up	5.02E+00	3.95E-26	up
ENSMUSG00000031774	Fam192a	-1.15E+00	4.72E-04	down	-1.39E+00	1.91E-06	down
ENSMUSG00000031906	Smpd3	-3.22E+00	8.42E-10	down	-4.89E-01	7.34E-01	no
ENSMUSG00000031728	Zfp821	-3.28E+00	5.68E-33	down	-2.08E+00	3.22E-11	down
ENSMUSG00000031447	Lamp1	-1.48E+00	1.90E-54	down	-1.95E+00	1.36E-100	down
ENSMUSG00000038398	Upf3a	-1.54E+00	8.15E-39	down	-1.60E+00	8.75E-63	down
ENSMUSG00000037725	Ckap2	1.39E+00	9.46E-02	no	-1.47E+00	4.56E-04	down
ENSMUSG00000015994	Fnta	-1.38E+00	3.69E-11	down	-2.05E+00	5.42E-39	down
ENSMUSG00000037214	Thap1	-4.60E-02	9.13E-01	no	-1.16E+00	4.99E-06	down
ENSMUSG00000039720	Got1l1	-2.26E+00	1.71E-25	down	-3.49E-01	4.25E-01	no
ENSMUSG00000037852	Cpe	2.36E+00	8.12E-04	up	8.71E-01	1.72E-01	no
ENSMUSG00000005470	Asf1b	1.72E+00	3.55E-08	up	-3.77E-01	2.25E-01	no
ENSMUSG00000031701	Dnaja2	-1.56E+00	8.27E-13	down	-2.21E+00	8.44E-31	down
ENSMUSG00000036598	Ccdc113	-1.65E+00	4.21E-60	down	-1.70E+00	2.22E-85	down
ENSMUSG00000035824	Tk2	-6.14E-01	6.09E-01	no	-2.27E+00	8.44E-05	down
ENSMUSG00000013160	Atp6v0d1	-2.55E-01	6.44E-01	no	-1.59E+00	4.95E-05	down
ENSMUSG00000031729	Ist1	-3.70E-01	4.43E-01	no	-1.70E+00	1.30E-06	down
ENSMUSG00000006517	Mvd	3.25E+00	5.04E-04	up	1.43E+00	6.71E-02	no
ENSMUSG00000015016	Acsf3	-2.59E+00	3.25E-05	down	-2.74E+00	1.63E-07	down
ENSMUSG00000038697	Taf5l	-2.47E+00	1.28E-06	down	-1.60E+00	2.36E-02	no
ENSMUSG00000031848	Lsm4	1.86E+00	2.35E-36	up	8.51E-01	1.39E-12	no
ENSMUSG00000005413	Hmox1	5.56E+00	6.45E-75	up	3.82E+00	2.05E-13	up
ENSMUSG00000031711	Zfp330	-2.34E+00	3.93E-19	down	-5.87E-01	2.33E-01	no
ENSMUSG00000005469	Prkaca	-2.78E+00	6.31E-22	down	-2.72E+00	3.26E-23	down
ENSMUSG00000039199	Zdhhc1	-1.16E+00	8.86E-03	no	-1.49E+00	2.25E-05	down
ENSMUSG00000031508	Ankrd10	1.23E+00	9.78E-02	no	-1.83E+00	8.29E-07	down
ENSMUSG00000019278	Dpep1	3.88E+00	4.09E-09	up	2.65E+00	1.58E-01	no
ENSMUSG00000038482	Tfdp1	-7.72E-01	3.68E-01	no	-3.32E+00	2.09E-15	down
ENSMUSG00000037363	Letm2	-3.25E+00	1.24E-65	down	-2.25E+00	2.32E-26	down
ENSMUSG00000031647	Mfap3l	-3.14E+00	3.69E-21	down	-4.92E-01	5.79E-01	no
ENSMUSG00000059355	BC056474	4.34E+00	2.10E-09	up	1.61E+00	1.02E-03	no
ENSMUSG00000048400	Prss54	-3.18E+00	3.90E-63	down	-2.65E-01	5.73E-01	no
ENSMUSG00000031756	Cenpn	-2.20E+00	9.55E-39	down	-1.88E+00	1.87E-30	down
ENSMUSG00000031831	Dnaaf1	-1.98E+00	2.34E-146	down	-1.38E+00	2.81E-88	down
ENSMUSG00000031483	Erlin2	-2.05E+00	4.91E-05	down	-2.94E+00	6.76E-15	down
ENSMUSG00000031600	Vps37a	-1.76E+00	3.86E-03	no	-2.56E+00	4.87E-08	down
ENSMUSG00000071104	Ccdc110	-5.08E+00	7.08E-67	down	-4.73E+00	4.65E-55	down
ENSMUSG00000070002	Ell	-1.21E+00	4.56E-02	no	-2.63E+00	1.16E-07	down
ENSMUSG00000031799	Tpm4	3.60E+00	4.59E-05	up	1.78E+00	3.45E-01	no
ENSMUSG00000031776	Arl2bp	-1.85E+00	4.37E-24	down	-1.45E+00	2.41E-10	down
ENSMUSG00000046707	Csnk2a2	-2.22E+00	4.82E-13	down	-2.20E+00	2.07E-16	down
ENSMUSG00000035770	Dync1li2	-2.20E+00	4.88E-12	down	-2.70E+00	8.52E-25	down

ENSMUSG00000033596	Rfwd3	-1.75E+00	1.57E-02	no	-2.92E+00	6.97E-07	down
ENSMUSG00000014470	Rnf166	-4.57E-01	1.64E-01	no	-1.66E+00	1.78E-28	down
ENSMUSG00000033862	Cdk10	-5.76E-01	2.80E-01	no	-1.97E+00	7.86E-14	down
ENSMUSG00000038497	Tmco3	3.07E+00	5.72E-05	up	6.64E-01	3.57E-01	no
ENSMUSG00000031545	Agpat6	-3.99E+00	2.70E-85	down	-2.72E+00	5.73E-31	down
ENSMUSG00000015341	Golga7	-2.19E-01	7.59E-01	no	-2.21E+00	1.72E-09	down
ENSMUSG00000022322	Shcbp1	-3.23E+00	5.47E-32	down	-1.43E+00	8.77E-04	down
ENSMUSG00000037103	Dcaf15	-2.72E+00	3.37E-22	down	-2.87E+00	1.25E-34	down
ENSMUSG00000037415	Ranbp10	-2.14E+00	1.88E-04	down	-2.15E+00	3.47E-05	down
ENSMUSG00000015027	Galns	2.14E+00	3.52E-04	up	2.38E+00	1.94E-06	up
ENSMUSG00000031536	Polb	-2.63E+00	8.45E-89	down	-2.39E+00	2.45E-93	down
ENSMUSG00000063932	Poteg	-2.69E+00	2.82E-13	down	-2.24E+00	5.72E-10	down
ENSMUSG00000011306	Sugp1	-2.15E+00	6.02E-20	down	-1.91E+00	3.43E-14	down
ENSMUSG00000031683	Lsm6	3.40E+00	4.26E-04	up	4.27E-01	6.19E-01	no
ENSMUSG00000014837	4931428F04Rik	-3.68E+00	1.09E-15	down	-1.41E+00	1.79E-01	no
ENSMUSG00000010154	Spire2	-2.76E+00	6.19E-06	down	-3.14E+00	1.95E-11	down
ENSMUSG00000071103	1700029J07Rik	-3.64E+00	3.90E-28	down	-1.14E+00	6.82E-02	no
ENSMUSG00000014786	Slc9a5	-1.52E+00	1.20E-02	no	-2.76E+00	2.91E-11	down
ENSMUSG00000031893	Tsnaxip1	-3.39E+00	1.17E-111	down	-2.86E+00	2.08E-87	down
ENSMUSG00000031916	Cog8	-1.87E+00	9.14E-06	down	-1.66E+00	1.61E-04	down
ENSMUSG00000031812	Map1lc3b	1.93E+00	9.84E-04	up	8.28E-01	1.78E-01	no
ENSMUSG00000000740	Rpl13	2.69E+00	2.61E-06	up	1.08E+00	3.13E-02	no
ENSMUSG00000019732	Calr3	-4.23E+00	1.34E-46	down	-2.52E+00	1.00E-05	down
ENSMUSG00000031913	Vps4a	-2.78E+00	9.45E-39	down	-2.75E+00	8.21E-38	down
ENSMUSG00000070003	Ssbp4	2.75E+00	6.44E-13	up	1.52E+00	2.81E-05	up
ENSMUSG00000005410	Mcm5	2.32E+00	4.86E-06	up	1.52E+00	2.85E-02	no
ENSMUSG00000031681	Smad1	-6.76E-01	5.98E-01	no	-2.44E+00	7.59E-04	down
ENSMUSG00000031696	Vps35	-1.72E+00	7.07E-07	down	-2.73E+00	5.46E-17	down
ENSMUSG00000008892	Vdac3	-1.20E+00	8.23E-13	down	-1.52E+00	3.57E-29	down
ENSMUSG00000061313	Ddhd2	3.41E+00	9.70E-04	up	9.19E-01	5.12E-01	no
ENSMUSG00000039530	Tusc3	-1.68E+00	3.97E-12	down	-2.05E+00	1.30E-23	down
ENSMUSG00000003037	Rab8a	-1.43E+00	1.11E-02	no	-1.92E+00	9.28E-06	down
ENSMUSG00000052794	1700030K09Rik	-2.72E+00	4.33E-11	down	-2.00E+00	1.26E-07	down
ENSMUSG00000052488	Cherp	-1.93E+00	2.75E-05	down	-3.28E+00	1.55E-14	down
ENSMUSG00000058355	Abce1	-2.54E+00	4.78E-12	down	-2.30E+00	7.33E-06	down
ENSMUSG00000031703	Itfg1	-2.87E+00	3.82E-24	down	-3.26E+00	3.26E-26	down
ENSMUSG00000031786	Ccdc135	-4.01E+00	2.82E-29	down	-2.99E+00	7.46E-23	down
ENSMUSG00000031976	Urb2	-1.66E+00	4.77E-03	no	-2.83E+00	5.02E-12	down
ENSMUSG00000031849	Comp	-3.17E+00	2.44E-57	down	-3.13E+00	8.48E-75	down
ENSMUSG00000031617	Tmem184c	-4.03E+00	7.53E-18	down	-2.36E+00	1.20E-02	no
ENSMUSG00000003814	Calr	-3.86E-01	9.16E-04	no	-1.30E+00	2.80E-33	down
ENSMUSG00000014791	Elmo3	-4.25E+00	5.95E-03	no	-5.24E+00	1.53E-06	down
ENSMUSG00000046691	Chtf8	-2.97E+00	4.99E-04	down	-3.19E+00	2.79E-04	down
ENSMUSG00000031970	Dbndd1	-4.19E+00	3.92E-15	down	-3.10E+00	1.09E-10	down
ENSMUSG00000035057	4933434I20Rik	-3.22E+00	3.21E-21	down	-3.86E+00	6.93E-52	down
ENSMUSG00000050079	Rspry1	-2.01E+00	4.05E-04	down	-1.87E+00	1.33E-02	no
ENSMUSG00000031787	Katnb1	-2.86E+00	5.78E-37	down	-1.81E+00	1.32E-13	down
ENSMUSG00000031727	Pmfbp1	-5.82E+00	3.42E-186	down	-2.95E+00	2.03E-16	down
ENSMUSG00000031512	Tex29	-1.77E+00	2.26E-41	down	6.09E-01	2.19E-02	no
ENSMUSG00000049577	Zfpm1	-2.97E+00	8.59E-04	down	-1.99E+00	3.83E-02	no
ENSMUSG00000001995	Sipa1l2	-1.97E+00	2.22E-02	no	-2.49E+00	6.71E-04	down
ENSMUSG00000031641	Cbr4	-8.05E-01	4.33E-01	no	-2.14E+00	1.73E-05	down
ENSMUSG00000031706	Rfx1	-3.87E+00	3.78E-21	down	-1.11E+00	1.91E-01	no
ENSMUSG00000031885	Cbfb	-1.45E+00	7.60E-02	no	-3.07E+00	1.59E-05	down
ENSMUSG00000042269	Fam92b	-2.65E+00	1.14E-02	no	-3.08E+00	1.28E-04	down
ENSMUSG00000008129	4930432K21Rik	-2.31E+00	5.92E-14	down	-1.76E+00	7.89E-08	down
ENSMUSG00000052566	Hook2	-2.01E+00	1.34E-11	down	-1.76E+00	2.54E-10	down
ENSMUSG00000031755	Bbs2	-2.24E+00	1.60E-04	down	-3.06E+00	1.47E-10	down
ENSMUSG00000031876	Cmtm1	-2.10E+00	3.22E-10	down	-1.23E+00	1.18E-03	no
ENSMUSG00000033579	Fa2h	-3.76E+00	2.25E-26	down	-3.14E+00	3.52E-14	down
ENSMUSG00000033409	Syce1l	-1.41E+00	1.32E-06	down	-1.63E+00	3.62E-13	down
ENSMUSG00000031631	4933411K20Rik	-2.85E+00	1.88E-46	down	-2.37E+00	1.91E-45	down
ENSMUSG00000031563	Wwc2	-2.51E+00	4.01E-08	down	-2.78E+00	8.02E-16	down
ENSMUSG00000037148	Arhgap10	-1.96E+00	7.84E-02	no	-4.03E+00	1.98E-05	down

ENSMUSG00000031751	Amfr	-8.95E-01	1.70E-02	no	-1.36E+00	3.81E-06	down
ENSMUSG00000031930	Wwp2	-2.98E+00	3.04E-13	down	-1.58E+00	2.09E-02	no
ENSMUSG00000033658	Ddx19b	-2.09E+00	2.00E-05	down	-3.18E+00	7.11E-20	down
ENSMUSG00000014633	Cmc2	1.66E+00	1.05E-07	up	1.64E+00	4.35E-10	up
ENSMUSG00000065954	Tacc1	-3.98E-01	6.59E-01	no	-2.18E+00	1.21E-05	down
ENSMUSG00000031585	Gtf2e2	-1.96E+00	7.48E-33	down	-2.10E+00	5.37E-47	down
ENSMUSG00000058301	Upf1	-2.42E+00	2.09E-04	down	-4.56E+00	3.50E-32	down
ENSMUSG00000005142	Man2b1	5.28E+00	4.99E-51	up	5.57E+00	2.43E-82	up
ENSMUSG00000031568	Rwdd4a	-1.60E+00	6.22E-06	down	-1.18E+00	5.16E-04	down
ENSMUSG00000033732	Sf3b3	-1.23E-01	8.77E-01	no	-2.05E+00	8.56E-06	down
ENSMUSG00000039509	Nup133	-1.13E+00	3.90E-02	no	-2.16E+00	6.15E-07	down
ENSMUSG00000040340	1700019B03Rik	-4.46E+00	1.18E-16	down	-2.78E+00	1.05E-03	no
ENSMUSG00000046408	1700067K01Rik	-1.27E+00	2.10E-04	down	-3.85E-02	9.59E-01	no
ENSMUSG00000037993	Dhx38	-2.46E+00	6.74E-07	down	-2.83E+00	1.64E-10	down
ENSMUSG00000031584	Gsr	4.22E+00	4.06E-26	up	7.13E+00	1.95E-65	up
ENSMUSG00000031529	Tnks	-4.82E+00	6.94E-20	down	-3.42E+00	5.66E-07	down
ENSMUSG00000038005	2700029M09Rik	-1.08E+00	2.39E-03	no	-1.19E+00	4.05E-04	down
ENSMUSG00000014856	Tmem208	2.98E+00	1.01E-09	up	8.61E-01	4.42E-02	no
ENSMUSG00000031818	Cox4i1	2.84E+00	0.00E+00	up	1.86E+00	0.00E+00	up
ENSMUSG00000031481	Tpte	-4.28E+00	5.60E-06	down	-2.21E+00	3.34E-01	no
ENSMUSG00000058833	2810428I15Rik	4.11E+00	5.11E-11	up	1.68E+00	3.21E-02	no
ENSMUSG00000031620	1700007B14Rik	-3.63E+00	1.09E-39	down	-8.56E-01	1.30E-01	no
ENSMUSG00000031809	1700018B08Rik	-1.22E+00	3.93E-28	down	1.26E-01	5.36E-01	no
ENSMUSG00000009628	Tex15	-2.52E+00	9.37E-08	down	-4.96E-01	7.70E-01	no
ENSMUSG00000090137	Uba52	2.02E+00	8.63E-06	up	1.79E+00	1.13E-03	no
ENSMUSG00000002190	Clgn	-2.56E+00	6.22E-114	down	-2.68E+00	8.48E-170	down
ENSMUSG00000031770	Herpud1	-1.21E+00	9.06E-06	down	-1.27E+00	4.70E-05	down
ENSMUSG00000031452	1700029H14Rik	-3.26E+00	7.19E-164	down	-7.66E-01	1.92E-04	no
ENSMUSG00000090206	Tepp	-4.76E+00	3.03E-36	down	-3.30E+00	6.01E-07	down
ENSMUSG00000003033	Ap1m1	-1.25E+00	1.68E-16	down	-1.17E+00	1.20E-18	down
ENSMUSG00000025808	Ccdc7	-2.57E+00	1.02E-08	down	-1.90E+00	2.84E-05	down
ENSMUSG00000039396	Neil3	-2.75E+00	1.79E-02	no	-3.68E+00	1.26E-04	down
ENSMUSG00000054320	Lrrc36	-3.49E+00	5.30E-32	down	-2.30E+00	2.82E-12	down
ENSMUSG00000031845	Bcmo1	-4.53E+00	2.58E-08	down	-1.75E+00	2.97E-01	no
ENSMUSG00000031851	Ntpcr	-1.49E+00	4.96E-05	down	-2.23E+00	4.94E-18	down
ENSMUSG00000031837	Necab2	-2.90E+00	4.82E-04	down	-4.05E+00	1.03E-07	down
ENSMUSG00000060038	Dhps	2.64E+00	2.81E-05	up	6.15E-01	4.83E-01	no
ENSMUSG00000031592	Pcm1	-1.84E+00	1.87E-08	down	-2.61E+00	7.28E-21	down
ENSMUSG00000031634	Ufsp2	4.08E-01	4.54E-01	no	-1.03E+00	9.57E-04	down
ENSMUSG00000031708	Tecr	1.08E+00	4.11E-05	up	7.25E-01	5.50E-03	no
ENSMUSG00000036180	Gatad2a	-2.02E+00	4.68E-07	down	-1.01E+00	1.10E-01	no
ENSMUSG00000004994	Ccdc130	-1.74E+00	2.49E-10	down	-1.25E+00	8.14E-07	down
ENSMUSG00000031660	Brd7	2.15E-01	7.09E-01	no	-1.14E+00	9.56E-04	down
ENSMUSG00000051648	Kctd19	-3.23E+00	9.08E-84	down	-3.11E+00	1.70E-104	down
ENSMUSG00000031576	Kcnu1	-4.78E+00	1.39E-28	down	-1.82E+00	2.15E-02	no
ENSMUSG00000057788	Ddx49	-2.16E+00	1.16E-09	down	-1.69E+00	5.93E-06	down
ENSMUSG00000055932	Fto	-2.66E+00	3.78E-04	down	-2.01E+00	3.08E-02	no
ENSMUSG00000031552	Adam18	-3.03E+00	1.66E-39	down	-3.09E+00	1.90E-61	down
ENSMUSG00000003813	Rad23a	-1.37E+00	5.19E-04	down	-1.22E+00	3.50E-04	down
ENSMUSG00000031985	Gnpat	-2.75E+00	1.95E-43	down	-2.00E+00	4.12E-21	down
ENSMUSG00000031948	Kars	-2.34E+00	7.38E-15	down	-2.75E+00	1.20E-27	down
ENSMUSG00000031637	Lrp2bp	-3.56E+00	7.09E-22	down	-1.04E+00	2.02E-01	no
ENSMUSG00000038215	Cep44	-7.39E-01	1.91E-01	no	-1.63E+00	1.20E-05	down
ENSMUSG00000055553	Kxd1	-8.42E-01	7.65E-02	no	-1.29E+00	4.02E-04	down
ENSMUSG00000031666	Rbl2	-3.95E+00	1.16E-10	down	-2.22E+00	2.94E-02	no
ENSMUSG00000031960	Aars	-1.81E+00	3.89E-06	down	-1.90E+00	6.65E-08	down
ENSMUSG00000031709	Tbc1d9	-4.81E+00	3.44E-09	down	-1.86E+00	3.40E-01	no
ENSMUSG00000037101	Ttc29	-3.70E+00	8.19E-54	down	-2.95E+00	2.63E-39	down
ENSMUSG00000036779	Papd5	-4.53E+00	3.15E-22	down	-4.00E+00	2.90E-12	down
ENSMUSG00000031601	Cnot7	-1.59E+00	1.33E-02	no	-3.10E+00	1.09E-08	down
ENSMUSG00000031691	Tnpo2	-2.46E+00	1.70E-02	no	-3.57E+00	5.28E-08	down
ENSMUSG00000014778	Fhod1	-2.43E+00	3.55E-03	no	-2.94E+00	3.17E-05	down
ENSMUSG00000031453	Rasa3	-2.92E+00	4.76E-07	down	-1.98E+00	6.62E-03	no
ENSMUSG00000031629	Mlf1ip	-2.05E+00	3.12E-07	down	-1.63E+00	2.17E-05	down

ENSMUSG00000038542	Pcid2	-1.64E+00	2.56E-04	down	-2.79E+00	1.18E-20	down
ENSMUSG00000031748	Gnao1	-2.19E+00	8.73E-02	no	-3.07E+00	9.57E-05	down
ENSMUSG00000031825	Crispld2	4.13E+00	7.80E-04	up	2.46E+00	4.37E-01	no
ENSMUSG00000019428	Fkbp8	-1.10E+00	1.21E-03	no	-1.98E+00	2.14E-14	down
ENSMUSG00000060098	Prmt7	-1.61E+00	1.59E-06	down	-1.50E+00	1.89E-06	down
ENSMUSG00000031921	Terf2	-1.45E+00	1.58E-01	no	-3.09E+00	9.23E-07	down
ENSMUSG00000001911	Nfix	3.07E+00	6.41E-04	up	3.91E+00	1.20E-03	no
ENSMUSG00000031446	Cul4a	-1.30E+00	3.98E-02	no	-2.39E+00	5.22E-09	down
ENSMUSG00000033282	Rpgrip1l	-2.98E+00	5.94E-04	down	-2.18E+00	8.55E-02	no
ENSMUSG00000031788	Kifc3	-3.66E+00	1.62E-10	down	-3.12E+00	2.76E-07	down
ENSMUSG00000052926	Rnaseh2a	-1.95E+00	5.84E-57	down	-1.87E+00	2.61E-64	down
ENSMUSG00000031554	Adam5	-4.19E+00	0.00E+00	down	-3.64E+00	0.00E+00	down
ENSMUSG00000002949	Timm44	-6.87E-01	1.45E-01	no	-1.30E+00	2.27E-05	down
ENSMUSG00000002395	Use1	-5.56E-01	3.96E-02	no	-1.10E+00	1.45E-07	down
ENSMUSG00000048827	Pkd1l3	-5.56E+00	5.92E-11	down	-3.05E+00	1.05E-01	no
ENSMUSG00000019470	Xab2	-1.67E+00	8.55E-05	down	-1.15E+00	2.27E-02	no
ENSMUSG00000036990	Otud4	-3.12E+00	1.24E-15	down	-1.36E+00	9.76E-02	no
ENSMUSG00000014782	Plekhg4	-5.82E-01	6.79E-01	no	-3.05E+00	2.44E-05	down
ENSMUSG00000031583	Wrn	-3.67E+00	6.07E-05	down	-4.05E+00	1.57E-05	down
ENSMUSG00000047866	Lonp2	-1.23E+00	1.24E-01	no	-2.30E+00	5.87E-04	down
ENSMUSG00000031586	Rbpms	2.33E+00	6.39E-04	up	7.58E-01	3.91E-01	no
ENSMUSG00000036054	Sugp2	-2.70E+00	1.61E-16	down	-2.46E+00	3.28E-17	down
ENSMUSG00000036686	Cc2d1a	-3.23E+00	1.31E-10	down	-2.68E+00	2.92E-06	down
ENSMUSG00000052616	Ccdc79	-1.54E+00	1.94E-02	no	-2.84E+00	8.20E-10	down
ENSMUSG00000056018	1700008F21Rik	-3.90E+00	8.34E-98	down	-3.73E+00	2.76E-81	down
ENSMUSG00000037437	Adam32	-3.26E+00	1.70E-186	down	-2.86E+00	2.81E-156	down
ENSMUSG00000018796	Acsl1	-4.64E+00	2.07E-50	down	-2.29E+00	5.02E-05	down
ENSMUSG00000031864	Ints10	-3.43E+00	3.95E-26	down	-3.07E+00	4.22E-24	down
ENSMUSG00000031878	Nae1	7.76E-01	4.34E-01	no	-1.93E+00	1.74E-04	down
ENSMUSG00000059854	Hydin	-4.43E+00	1.17E-09	down	-3.38E+00	9.05E-08	down
ENSMUSG00000038291	Snx25	-3.57E+00	2.43E-07	down	-4.65E+00	1.54E-19	down
ENSMUSG00000030465	Psd3	-3.81E+00	2.09E-06	down	-1.77E+00	2.53E-01	no
ENSMUSG00000004626	Stxbp2	-1.29E+00	3.53E-03	no	-1.67E+00	4.23E-06	down
ENSMUSG00000036550	Cnot1	-3.52E+00	4.13E-22	down	-3.83E+00	4.86E-34	down
ENSMUSG00000031753	Cog4	-1.99E+00	7.99E-09	down	-2.06E+00	6.57E-11	down
ENSMUSG00000002948	Map2k7	-3.75E+00	2.39E-13	down	-3.41E+00	1.14E-10	down
ENSMUSG00000031553	Adam3	-3.67E+00	0.00E+00	down	-2.71E+00	1.48E-144	down
ENSMUSG00000031575	Ash2l	-2.05E+00	2.96E-12	down	-2.23E+00	8.44E-20	down
ENSMUSG00000036270	Edc4	-1.21E+00	3.38E-02	no	-3.15E+00	4.29E-21	down
ENSMUSG00000000738	Spg7	-9.77E-01	1.29E-02	no	-1.48E+00	2.13E-06	down
ENSMUSG00000011832	Evi5l	-3.74E+00	2.50E-14	down	-3.14E+00	9.63E-09	down
ENSMUSG00000036872	Abcc12	-3.92E+00	6.59E-13	down	-4.01E+00	6.41E-29	down
ENSMUSG00000047619	Ddi1	-3.08E+00	4.55E-105	down	-4.07E-01	1.73E-01	no
ENSMUSG00000091460	Gm7808	2.47E+00	9.83E-12	up	3.06E+00	7.80E-10	up
ENSMUSG00000047995	Cypt4	7.08E-01	7.69E-57	no	1.41E+00	9.75E-62	up
ENSMUSG00000049932	H2afx	5.05E+00	3.24E-228	up	3.29E+00	1.20E-193	up
ENSMUSG00000042293	Gm5617	1.06E+00	1.04E-151	up	1.23E+00	1.15E-265	up
ENSMUSG00000058443	Rpl10-ps3	6.74E+00	5.90E-04	up	3.52E+00	1.23E-01	no
ENSMUSG00000044820	AY074887	5.96E+00	2.92E-04	up	4.57E+00	2.02E-02	no
ENSMUSG00000032288	Imp3	1.89E+00	1.73E-07	up	3.16E-01	3.93E-01	no
ENSMUSG00000035337	Uchl4	-8.75E-01	4.08E-04	no	-1.34E+00	3.28E-11	down
ENSMUSG00000055125	M5C1000I18Rik	-2.03E+00	1.50E-01	no	-4.16E+00	5.71E-10	down
ENSMUSG00000064225	Paqr9	-5.04E+00	3.47E-26	down	-2.17E+00	6.52E-03	no
ENSMUSG00000062933	Gm10123	1.57E+00	2.67E-151	up	-5.17E-02	4.08E-01	no
ENSMUSG00000091735	Gpr62	-1.97E+00	1.06E-07	down	-2.30E+00	1.66E-16	down
ENSMUSG00000066368	Actl11	-3.92E+00	3.81E-59	down	-7.85E-01	1.25E-01	no
ENSMUSG00000074075	Gm10621	-1.98E+00	9.56E-04	down	-8.58E-01	3.46E-01	no
ENSMUSG00000066415	Msl2	-3.13E+00	1.01E-03	no	-4.87E+00	1.72E-07	down
ENSMUSG00000066382	Iqcf5	-1.91E+00	2.83E-60	down	-9.97E-02	7.54E-01	no
ENSMUSG00000091129	Iqcf6	-2.76E+00	9.59E-25	down	-1.22E+00	2.69E-02	no
ENSMUSG00000010045	Tmem115	-1.43E+00	6.48E-04	down	-2.07E+00	7.13E-12	down
ENSMUSG00000063856	Gpx1	5.86E+00	2.91E-56	up	3.70E+00	3.67E-17	up
ENSMUSG00000049305	Ccdc71	-2.37E+00	1.82E-06	down	-1.86E+00	5.21E-04	down
ENSMUSG00000025652	Tmem89	5.94E-01	6.73E-08	no	1.09E+00	2.12E-25	up

ENSMUSG00000031931	Ankrd49	-2.79E+00	1.09E-20	down	-1.52E+00	2.98E-05	down
ENSMUSG00000096472	Cdkn2d	-6.49E-02	8.80E-01	no	-1.72E+00	1.09E-25	down
ENSMUSG00000059830	Gm16380	3.76E+00	2.20E-04	up	2.58E+00	1.41E-02	no
ENSMUSG00000046846	Spesp1	-1.50E+00	3.03E-35	down	-3.64E-01	6.43E-03	no
ENSMUSG00000032244	Fem1b	-3.07E+00	1.75E-35	down	-2.15E+00	4.12E-12	down
ENSMUSG00000032403	2300009A05Rik	1.87E+00	4.65E-64	up	1.63E+00	1.66E-61	up
ENSMUSG00000035048	Anapc13	1.40E+00	6.07E-45	up	6.89E-01	1.96E-17	no
ENSMUSG00000032566	1700080E11Rik	-6.20E+00	2.67E-50	down	-5.08E+00	7.33E-15	down
ENSMUSG00000041009	Iqcf4	-2.41E+00	4.85E-87	down	-4.10E-01	9.80E-02	no
ENSMUSG00000039461	Tcta	-2.93E-01	5.80E-01	no	-1.60E+00	1.74E-10	down
ENSMUSG00000038691	Mbd3l1	-2.26E+00	4.41E-60	down	2.78E-01	4.11E-01	no
ENSMUSG00000032060	Cryab	3.50E+00	2.05E-05	up	3.58E+00	5.98E-02	no
ENSMUSG00000007892	Rplp1	2.69E+00	0.00E+00	up	1.64E+00	4.89E-166	up
ENSMUSG00000032226	Gcnt3	-2.93E+00	7.67E-04	down	-3.52E+00	4.92E-05	down
ENSMUSG00000031998	1700128F08Rik	-3.30E+00	1.66E-02	no	-3.89E+00	9.29E-04	down
ENSMUSG00000032353	Tmed3	2.21E+00	4.05E-08	up	2.79E-01	5.66E-01	no
ENSMUSG00000032611	1700102P08Rik	1.22E+00	2.68E-17	up	-8.99E-01	1.61E-38	no
ENSMUSG00000050641	BC048562	-2.86E+00	6.86E-41	down	-5.26E-01	2.48E-01	no
ENSMUSG00000091537	Tma7	2.40E+00	3.81E-61	up	1.50E+00	4.45E-40	up
ENSMUSG00000032434	Cmtm6	-1.19E+00	1.89E-01	no	-2.90E+00	9.94E-05	down
ENSMUSG00000032515	Csrnp1	-1.71E+00	1.78E-05	down	-2.32E+00	2.93E-07	down
ENSMUSG00000006941	Eif1b	1.15E+00	5.07E-21	up	-6.34E-01	7.94E-16	no
ENSMUSG00000043773	1700048O20Rik	3.26E-01	3.40E-01	no	-1.35E+00	2.98E-11	down
ENSMUSG00000095352	Gm20783	-1.27E+00	3.37E-03	no	-1.30E+00	1.91E-04	down
ENSMUSG00000032171	Pin1	3.51E+00	1.09E-18	up	1.73E+00	1.17E-13	up
ENSMUSG00000091028	Gm10722	1.42E+01	5.59E-105	up	4.87E+00	3.01E-05	up
ENSMUSG00000032110	Acrv1	-3.10E+00	8.33E-205	down	-5.84E-01	3.67E-03	no
ENSMUSG00000032121	Tmem218	-6.49E-01	1.94E-01	no	-1.46E+00	4.84E-06	down
ENSMUSG00000042138	Msantd2	-1.40E+00	2.33E-01	no	-3.30E+00	7.82E-05	down
ENSMUSG00000040541	Tmem225	-3.10E+00	3.27E-79	down	-7.30E-01	1.46E-02	no
ENSMUSG00000032014	Oaf	5.99E+00	6.72E-08	up	5.59E+00	1.07E-01	no
ENSMUSG00000079564	Gm11149	-3.51E+00	2.72E-07	down	-4.07E-01	8.56E-01	no
ENSMUSG00000032291	Crabp1	6.19E+00	6.46E-41	up	6.34E+00	3.30E-24	up
ENSMUSG00000066383	Iqcf1	-1.39E+00	1.40E-44	down	5.46E-02	8.57E-01	no
ENSMUSG00000044664	Prss42	-2.08E+00	2.42E-12	down	-2.31E+00	4.71E-24	down
ENSMUSG00000048752	Prss50	5.52E+00	4.80E-78	up	2.71E+00	4.45E-08	up
ENSMUSG00000057802	Gm10030	2.25E+00	2.27E-06	up	1.16E-01	8.52E-01	no
ENSMUSG00000086596	Susd5	-2.21E+00	3.88E-01	no	-3.53E+00	6.29E-04	down
ENSMUSG00000063488	Zkscan7	-1.60E+00	1.50E-01	no	-2.68E+00	3.25E-04	down
ENSMUSG00000053914	Kdm4d	-2.76E+00	2.79E-22	down	-2.61E+00	4.23E-28	down
ENSMUSG00000074476	Spc24	3.82E+00	2.02E-20	up	1.62E+00	1.66E-06	up
ENSMUSG00000095186	Gm10718	1.47E+01	5.21E-63	up	4.55E+00	2.73E-04	up
ENSMUSG00000009927	Rps25	2.56E+00	8.69E-69	up	8.56E-01	1.71E-09	no
ENSMUSG00000042396	Rbm7	-2.11E+00	8.86E-22	down	-4.10E-01	3.85E-01	no
ENSMUSG00000032065	Tex12	2.77E+00	1.97E-21	up	-1.38E-01	6.86E-01	no
ENSMUSG00000032056	Btg4	-3.42E+00	2.58E-08	down	-2.39E+00	8.46E-05	down
ENSMUSG00000045620	Odf3l1	-2.78E+00	3.09E-62	down	2.13E-01	6.38E-01	no
ENSMUSG00000000088	Cox5a	3.29E+00	0.00E+00	up	2.67E+00	0.00E+00	up
ENSMUSG00000049526	Tmem202	-6.33E+00	1.21E-42	down	-6.55E+00	9.87E-71	down
ENSMUSG00000041837	Pdcd7	1.52E+00	1.46E-02	no	-1.25E+00	7.08E-04	down
ENSMUSG00000032383	Ppib	2.04E+00	4.35E-09	up	-3.29E-01	5.10E-01	no
ENSMUSG00000032381	Fam96a	1.79E-01	7.55E-01	no	-1.63E+00	4.84E-09	down
ENSMUSG00000032451	Trim42	-4.93E+00	7.21E-12	down	-3.10E+00	5.93E-02	no
ENSMUSG00000096316	Gm6432	-3.89E+00	4.89E-22	down	-2.67E+00	1.42E-04	down
ENSMUSG00000032606	Nicn1	-1.78E+00	1.08E-03	no	-2.34E+00	1.62E-11	down
ENSMUSG00000007815	Rhoa	3.85E-01	3.26E-01	no	-1.42E+00	1.05E-06	down
ENSMUSG00000032599	Ip6k2	-2.97E+00	6.72E-07	down	-4.13E+00	9.65E-17	down
ENSMUSG00000058398	Prss43	-1.07E+00	4.33E-02	no	-2.34E+00	2.88E-13	down
ENSMUSG00000025794	Rpl14	5.19E+00	1.71E-82	up	1.51E+00	1.87E-17	up
ENSMUSG00000047036	Zfp445	-3.06E+00	2.26E-02	no	-4.00E+00	2.80E-04	down
ENSMUSG00000058173	Smco4	-4.71E+00	3.33E-17	down	-2.47E+00	1.41E-02	no
ENSMUSG00000057551	Zfp317	-5.91E+00	3.44E-17	down	-3.58E+00	7.54E-05	down
ENSMUSG00000038742	Angptl6	7.45E+00	1.28E-13	up	6.57E+00	2.25E-08	up
ENSMUSG00000057982	Zfp809	-2.09E+00	5.39E-03	no	-2.17E+00	5.52E-04	down

ENSMUSG00000050555	Hyls1	-3.64E+00	4.64E-16	down	-4.12E+00	3.37E-29	down
ENSMUSG00000025894	Aasdhppt	-6.88E-01	2.63E-01	no	-2.03E+00	8.23E-09	down
ENSMUSG00000003131	Pafah1b2	-1.19E+00	7.76E-06	down	-1.35E+00	3.40E-07	down
ENSMUSG00000032057	4833427G06Rik	-2.06E+00	3.61E-35	down	-1.61E+00	5.74E-24	down
ENSMUSG00000035594	Chrna5	-4.84E+00	5.21E-07	down	-2.12E+00	3.71E-01	no
ENSMUSG00000050721	Plekho2	-2.13E+00	6.64E-04	down	-2.37E+00	8.80E-02	no
ENSMUSG00000032202	Rab27a	-2.18E+00	1.58E-02	no	-3.31E+00	9.55E-07	down
ENSMUSG00000032417	Rwdd2a	-2.55E+00	2.00E-27	down	-2.23E+00	1.49E-25	down
ENSMUSG00000032475	Nck1	2.88E-01	7.17E-01	no	-1.57E+00	5.63E-05	down
ENSMUSG00000047220	Ccdc36	-4.79E-01	5.46E-01	no	-2.33E+00	1.13E-04	down
ENSMUSG00000032518	Rpsa	-2.09E-01	6.66E-01	no	-1.53E+00	1.64E-05	down
ENSMUSG00000004096	Cwc15	-4.66E-01	7.24E-03	no	-1.44E+00	1.14E-29	down
ENSMUSG00000037405	Icam1	4.24E+00	3.12E-09	up	3.23E+00	2.90E-02	no
ENSMUSG00000036411	9530077C05Rik	-2.20E+00	4.30E-17	down	-2.42E+00	1.29E-32	down
ENSMUSG00000031991	Spata19	-1.98E+00	6.06E-287	down	-2.91E-01	5.51E-03	no
ENSMUSG00000046240	Hepacam	-2.71E+00	5.87E-07	down	-8.40E-01	4.12E-01	no
ENSMUSG00000053310	Nrgn	-2.38E+00	1.03E-04	down	-1.80E-01	9.27E-01	no
ENSMUSG00000032083	Apoa1	3.76E+00	4.18E-08	up	2.28E+00	2.35E-02	no
ENSMUSG00000032324	Tspan3	1.27E+00	1.30E-05	up	1.04E-01	8.03E-01	no
ENSMUSG00000057367	Birc2	-2.48E+00	5.72E-12	down	-3.03E+00	3.76E-24	down
ENSMUSG00000053070	9230110C19Rik	-2.08E+00	1.08E-12	down	-2.92E+00	3.42E-38	down
ENSMUSG00000032251	Irak1bp1	-1.12E+00	2.70E-06	down	-1.21E+00	2.12E-10	down
ENSMUSG00000032412	Atp1b3	-2.20E+00	3.80E-55	down	-1.71E+00	2.88E-17	down
ENSMUSG00000048758	Rpl29	-8.83E-01	1.04E-02	no	-1.98E+00	3.31E-08	down
ENSMUSG00000023577	Iqcf3	-2.98E+00	2.06E-160	down	-9.95E-01	4.83E-07	no
ENSMUSG00000057895	Zfp105	-2.96E+00	4.45E-24	down	-2.11E+00	3.99E-11	down
ENSMUSG00000019471	Cdc37	-1.02E+00	6.28E-04	down	-1.69E+00	1.60E-11	down
ENSMUSG00000040883	Tmem205	-1.05E+00	4.91E-10	down	6.55E-01	1.42E-02	no
ENSMUSG00000008429	Herpud2	-3.12E+00	2.17E-16	down	-2.09E+00	3.42E-03	no
ENSMUSG00000066705	Fxyd6	3.19E+00	7.81E-05	up	1.02E+00	3.41E-01	no
ENSMUSG00000050702	Rfpl3s	-5.26E+00	1.49E-35	down	-4.03E+00	6.91E-18	down
ENSMUSG00000032299	Commd4	3.24E+00	4.74E-09	up	1.25E+00	1.55E-03	no
ENSMUSG00000036943	Rab8b	-1.30E+00	8.96E-02	no	-2.76E+00	3.22E-07	down
ENSMUSG00000032002	Dcun1d5	-4.15E-01	2.06E-02	no	-1.40E+00	1.78E-20	down
ENSMUSG00000032459	Mrps22	-1.07E+00	3.91E-05	down	-1.46E+00	2.36E-12	down
ENSMUSG00000020257	Wdr82	-1.46E+00	2.48E-01	no	-2.90E+00	4.63E-04	down
ENSMUSG00000032602	Slc25a20	-1.67E+00	7.95E-04	down	-2.06E+00	9.96E-10	down
ENSMUSG00000049719	Prss46	-1.71E+00	2.14E-28	down	4.13E-01	1.49E-01	no
ENSMUSG00000032436	Cmtm7	3.86E+00	7.51E-05	up	2.93E+00	1.34E-02	no
ENSMUSG00000032134	Muc16	-3.12E+00	3.33E-04	down	-1.02E+00	6.13E-01	no
ENSMUSG00000003299	Mrpl4	1.75E+00	4.13E-09	up	8.78E-01	8.93E-04	no
ENSMUSG00000005131	4930550C14Rik	-2.04E+00	9.66E-26	down	-1.21E+00	7.64E-09	down
ENSMUSG00000040188	Scamp2	-1.82E+00	5.36E-21	down	-1.08E-01	8.50E-01	no
ENSMUSG00000032323	Cyp11a1	2.36E+00	3.67E-02	no	4.31E+00	1.29E-13	up
ENSMUSG00000032218	Ccnb2	-1.19E+00	3.93E-18	down	-9.18E-01	2.93E-12	no
ENSMUSG00000032009	Sesn3	-1.10E+00	1.65E-01	no	-2.80E+00	5.91E-10	down
ENSMUSG00000002820	Atg4d	-1.07E+00	8.19E-02	no	-1.60E+00	2.04E-05	down
ENSMUSG00000040563	BC018242	-3.94E+00	2.62E-05	down	-2.00E+00	1.80E-01	no
ENSMUSG00000037257	Aagab	-2.66E-01	7.97E-01	no	-2.12E+00	9.16E-04	down
ENSMUSG00000032399	Rpl4	1.19E-01	4.00E-01	no	-1.77E+00	9.03E-82	down
ENSMUSG00000032221	Mns1	-1.72E+00	4.45E-75	down	-1.46E+00	1.79E-60	down
ENSMUSG00000010057	Nprl2	-1.32E+00	2.28E-03	no	-1.32E+00	1.47E-04	down
ENSMUSG00000032601	Prkar2a	-5.89E+00	6.11E-28	down	-4.70E+00	3.37E-06	down
ENSMUSG00000032493	Prss44	-1.25E+00	3.78E-02	no	-2.17E+00	2.18E-12	down
ENSMUSG00000047257	Prss45	-2.16E+00	5.64E-17	down	-2.67E+00	1.38E-44	down
ENSMUSG00000031993	Snx19	-2.90E+00	1.93E-04	down	-3.54E+00	1.85E-11	down
ENSMUSG00000047412	Zbtb44	-2.14E+00	2.60E-05	down	-2.08E+00	2.65E-06	down
ENSMUSG00000032038	St3gal4	-2.48E+00	3.71E-04	down	-4.78E-01	7.79E-01	no
ENSMUSG00000055720	Ubl7	-1.96E+00	1.02E-69	down	-1.21E+00	9.18E-28	down
ENSMUSG00000036244	Tbc1d21	-3.04E+00	1.01E-107	down	-3.35E-01	2.80E-01	no
ENSMUSG00000033629	Ptplad1	-8.20E-02	9.23E-01	no	-2.41E+00	8.57E-08	down
ENSMUSG00000040729	AK129341	-3.48E+00	1.49E-49	down	-3.02E+00	3.69E-37	down
ENSMUSG00000074149	Gm10634	-3.48E+00	8.02E-05	down	-4.95E+00	5.98E-07	down
ENSMUSG00000032463	Faim	2.87E+00	2.53E-09	up	4.08E-01	3.15E-01	no

ENSMUSG00000010044	Zmynd10	-1.45E+00	2.77E-28	down	-1.86E+00	1.14E-118	down
ENSMUSG00000019039	Dalrd3	-1.66E+00	2.51E-07	down	-1.67E+00	1.20E-08	down
ENSMUSG00000064299	4921528I07Rik	-4.71E+00	7.23E-20	down	-2.73E+00	7.30E-02	no
ENSMUSG00000032508	Myd88	4.88E-01	6.40E-01	no	-2.61E+00	1.56E-06	down
ENSMUSG00000079084	Ccdc82	-4.23E+00	1.94E-06	down	-4.21E+00	1.06E-03	no
ENSMUSG00000032101	Ddx25	-1.38E+00	1.37E-83	down	-1.09E+00	1.03E-69	down
ENSMUSG00000090626	Tex9	-1.58E+00	2.15E-04	down	-1.81E+00	7.44E-07	down
ENSMUSG00000042487	Leo1	-1.54E+00	5.31E-06	down	-1.18E+00	2.78E-04	down
ENSMUSG00000074146	4930579C12Rik	-3.12E+00	7.58E-05	down	-4.47E-01	8.66E-01	no
ENSMUSG00000023495	Pcbp4	-2.66E+00	4.86E-05	down	-2.62E+00	1.75E-04	down
ENSMUSG00000037949	Ano10	-1.52E+00	1.88E-05	down	-1.92E+00	1.98E-11	down
ENSMUSG00000031939	Taf1d	1.34E+00	5.03E-05	up	-5.92E-01	6.36E-02	no
ENSMUSG00000010205	Raver1	-2.87E+00	1.39E-11	down	-3.33E+00	8.78E-19	down
ENSMUSG00000032023	4931429I11Rik	-4.72E+00	1.83E-30	down	-3.76E+00	1.05E-16	down
ENSMUSG00000032290	Ptpn9	-1.86E+00	5.78E-04	down	-1.38E+00	3.76E-02	no
ENSMUSG00000032316	Clk3	-2.13E+00	1.09E-19	down	-1.39E+00	8.76E-07	down
ENSMUSG00000095547	Gm10719	5.06E+00	5.06E-06	up	#VALUE!	NA	no
ENSMUSG00000032097	Ddx6	-1.46E+00	1.24E-01	no	-3.55E+00	3.60E-10	down
ENSMUSG00000025232	Hexa	-9.85E-01	2.06E-06	no	-1.43E+00	1.25E-13	down
ENSMUSG00000032405	Pias1	-3.57E+00	1.30E-44	down	-1.77E+00	5.71E-06	down
ENSMUSG00000040524	Zfp609	-2.77E+00	5.57E-02	no	-4.36E+00	2.16E-04	down
ENSMUSG00000013584	Aldh1a2	-1.70E+00	1.24E-12	down	-1.95E+00	1.59E-23	down
ENSMUSG00000032352	Lrrc1	-3.75E+00	1.62E-34	down	-2.97E+00	2.61E-20	down
ENSMUSG00000032418	Me1	-2.69E+00	8.24E-18	down	-2.90E+00	3.00E-29	down
ENSMUSG00000032803	Cdv3	-1.54E+00	1.42E-07	down	-4.95E-01	4.38E-01	no
ENSMUSG00000023262	Acy1	5.01E+00	7.63E-04	up	2.42E+00	1.50E-01	no
ENSMUSG00000032477	Cdc25a	-2.38E+00	5.82E-14	down	-1.58E+00	2.68E-04	down
ENSMUSG00000032530	Lyzl4	-2.38E+00	1.14E-29	down	1.27E-01	8.26E-01	no
ENSMUSG00000025785	Exosc7	4.06E+00	1.53E-08	up	1.62E+00	4.97E-04	up
ENSMUSG00000064145	Arih2	-2.59E+00	4.72E-23	down	-2.37E+00	4.66E-17	down
ENSMUSG00000032437	Stt3b	-3.80E+00	2.09E-106	down	-3.48E+00	5.67E-85	down
ENSMUSG00000032536	Trak1	-2.49E+00	9.38E-03	no	-4.09E+00	9.04E-07	down
ENSMUSG00000034342	Cbl	-3.18E+00	2.84E-09	down	-2.76E+00	9.00E-07	down
ENSMUSG00000032127	Vps11	-1.10E+00	5.23E-02	no	-1.98E+00	1.87E-05	down
ENSMUSG00000032264	Zw10	-2.13E+00	9.59E-05	down	-2.46E+00	2.56E-06	down
ENSMUSG00000025235	Bbs4	-1.75E+00	2.12E-03	no	-1.92E+00	7.97E-05	down
ENSMUSG00000092192	Dyx1c1	-1.13E+00	1.65E-01	no	-3.07E+00	2.49E-15	down
ENSMUSG00000032350	Gclc	7.34E-01	4.21E-01	no	-2.19E+00	2.49E-05	down
ENSMUSG00000035382	Pcsk7	-1.88E+00	3.07E-02	no	-3.70E+00	3.80E-06	down
ENSMUSG00000070291	Ddx43	6.01E-02	9.43E-01	no	-2.29E+00	3.38E-07	down
ENSMUSG00000038895	Zfp653	-1.48E+00	2.16E-03	no	-1.96E+00	2.77E-06	down
ENSMUSG00000056267	Cep70	-6.16E-01	1.56E-01	no	-1.92E+00	3.38E-12	down
ENSMUSG00000032512	Wdr48	-2.94E+00	7.98E-62	down	-2.70E+00	2.80E-67	down
ENSMUSG00000035047	Kri1	-2.53E+00	9.99E-03	no	-4.61E+00	1.36E-15	down
ENSMUSG00000034485	Uaca	-3.08E+00	4.23E-10	down	-1.99E+00	5.57E-03	no
ENSMUSG00000056167	Cnot10	-7.57E-01	5.44E-02	no	-1.89E+00	1.40E-09	down
ENSMUSG00000094985	Topaz1	-1.09E+00	2.07E-01	no	-2.74E+00	2.28E-08	down
ENSMUSG00000031966	Glb1l3	-2.83E+00	8.50E-12	down	-2.79E-02	9.82E-01	no
ENSMUSG00000096385	Gm11168	8.52E+00	3.25E-49	up	#VALUE!	NA	no
ENSMUSG00000025602	Zfp202	-1.87E+00	8.14E-02	no	-2.91E+00	1.14E-04	down
ENSMUSG00000032096	Arcn1	-1.63E+00	3.82E-03	no	-2.97E+00	3.03E-09	down
ENSMUSG00000032285	Dnaja4	-3.49E+00	5.39E-41	down	-1.39E+00	3.75E-03	no
ENSMUSG00000032320	Rcn2	9.31E-01	2.06E-01	no	-1.88E+00	9.59E-05	down
ENSMUSG00000032278	Paqr5	-3.98E+00	7.86E-11	down	-2.31E+00	7.53E-02	no
ENSMUSG00000032245	Cln6	-1.45E+00	3.57E-05	down	-1.10E+00	1.06E-04	down
ENSMUSG00000079334	Nat6	-1.52E+00	4.04E-07	down	1.48E+00	3.63E-03	no
ENSMUSG00000006673	Qrich1	-2.49E+00	7.97E-33	down	-2.66E+00	3.71E-41	down
ENSMUSG00000035202	Lars2	2.07E+00	6.48E-58	up	1.14E+00	1.23E-11	up
ENSMUSG00000032212	Sltm	-2.26E+00	5.41E-33	down	-1.87E+00	3.88E-19	down
ENSMUSG00000032612	Usp4	-2.86E+00	2.61E-34	down	-2.40E+00	9.18E-25	down
ENSMUSG00000085576	Dpy19l2	-3.77E+00	4.05E-44	down	-3.63E+00	7.09E-45	down
ENSMUSG00000032293	Ireb2	-3.99E+00	9.83E-14	down	-2.35E+00	8.71E-03	no
ENSMUSG00000032306	Mpi	-1.07E+00	1.59E-10	down	-1.14E+00	8.09E-21	down
ENSMUSG00000032294	Pkm	-1.99E+00	6.25E-20	down	-5.85E-01	1.87E-01	no

ENSMUSG00000058444	Map2k5	-7.63E-01	7.97E-02	no	-1.60E+00	7.35E-09	down
ENSMUSG00000038535	Zfp280d	-2.05E+00	1.26E-02	no	-2.76E+00	5.45E-04	down
ENSMUSG00000032328	Tmem30a	-1.79E+00	3.04E-05	down	-2.88E+00	2.99E-12	down
ENSMUSG00000038379	Ttk	-2.81E+00	6.16E-12	down	-3.37E+00	1.13E-22	down
ENSMUSG00000032458	Copb2	-2.54E-01	5.59E-01	no	-1.94E+00	2.51E-10	down
ENSMUSG00000032175	Tyk2	-6.17E-01	6.80E-01	no	-3.15E+00	2.45E-04	down
ENSMUSG00000011114	Tbrg1	-1.22E+00	1.79E-07	down	-1.15E+00	1.36E-08	down
ENSMUSG00000032254	Kif23	-1.83E+00	6.29E-05	down	-2.29E+00	1.49E-04	down
ENSMUSG00000036781	Rps27l	4.06E+00	2.43E-294	up	2.26E+00	4.51E-139	up
ENSMUSG00000074139	1700057G04Rik	-2.84E+00	2.85E-22	down	-5.56E-01	3.62E-01	no
ENSMUSG00000038708	Golga4	-2.98E+00	1.74E-24	down	-2.79E+00	2.24E-24	down
ENSMUSG00000032523	Hhatl	-1.80E+00	2.81E-02	no	-3.10E+00	6.46E-05	down
ENSMUSG00000036777	Anln	-1.15E+00	2.30E-01	no	-2.60E+00	1.02E-04	down
ENSMUSG00000032215	Rsl24d1	-1.10E+00	1.90E-02	no	-1.81E+00	9.28E-07	down
ENSMUSG00000036057	Ptpn23	-2.09E+00	1.11E-03	no	-2.51E+00	1.17E-05	down
ENSMUSG00000032021	Crtam	-3.08E+00	1.82E-10	down	-3.65E+00	4.29E-29	down
ENSMUSG00000034858	Fam214a	-2.33E+00	1.30E-02	no	-3.57E+00	1.39E-07	down
ENSMUSG00000042688	Mapk6	-2.23E+00	1.33E-19	down	-2.07E+00	1.00E-15	down
ENSMUSG00000062270	Morf4l1	-1.79E+00	2.49E-06	down	-2.60E+00	4.56E-16	down
ENSMUSG00000031938	4931406C07Rik	-1.99E+00	5.74E-03	no	-3.05E+00	1.47E-08	down
ENSMUSG00000006241	Ccdc159	-4.33E+00	2.96E-24	down	-2.64E+00	1.59E-03	no
ENSMUSG00000039632	Ccdc151	-2.90E+00	1.26E-21	down	-2.68E+00	2.22E-25	down
ENSMUSG00000056617	4931429L15Rik	-5.03E+00	4.68E-54	down	-1.89E+00	9.69E-04	down
ENSMUSG00000034263	Vwa9	-2.03E+00	3.42E-04	down	-2.35E+00	1.75E-06	down
ENSMUSG00000032456	Nmnat3	8.77E-01	2.54E-02	no	1.17E+00	1.65E-04	up
ENSMUSG00000032555	Topbp1	-8.95E-02	9.19E-01	no	-2.43E+00	1.85E-06	down
ENSMUSG00000001833	43715	-2.09E-01	6.53E-01	no	-1.99E+00	7.48E-09	down
ENSMUSG00000032281	Acsbg1	3.50E+00	4.53E-09	up	1.78E+00	8.20E-03	no
ENSMUSG00000032497	Lrrfip2	-3.06E+00	3.58E-50	down	-2.44E+00	7.74E-29	down
ENSMUSG00000070280	Slc22a14	-3.02E+00	3.09E-67	down	-1.04E-01	8.51E-01	no
ENSMUSG00000074500	Zfp558	-4.63E+00	2.10E-11	down	-2.77E+00	6.44E-03	no
ENSMUSG00000032540	Abhd5	-2.39E+00	2.54E-22	down	-1.58E+00	2.58E-06	down
ENSMUSG00000032314	Etfa	2.71E+00	3.47E-05	up	6.52E-01	4.38E-01	no
ENSMUSG00000032186	Tmod2	-4.34E+00	5.77E-09	down	-1.74E+00	3.54E-01	no
ENSMUSG00000040219	Ttc12	-3.53E+00	3.08E-13	down	-3.60E+00	3.80E-21	down
ENSMUSG00000000167	Pih1d2	-2.32E+00	1.45E-07	down	-2.46E+00	7.34E-16	down
ENSMUSG00000034007	Scaper	-1.66E+00	2.16E-04	down	-1.86E+00	3.11E-05	down
ENSMUSG00000037742	Eef1a1	-2.24E+00	1.67E-141	down	-2.92E+00	3.58E-262	down
ENSMUSG00000032567	Aste1	-2.53E+00	7.60E-12	down	-1.93E+00	1.04E-05	down
ENSMUSG00000032579	Hemk1	4.06E-01	7.21E-01	no	-1.64E+00	4.27E-04	down
ENSMUSG00000013076	Amotl1	-3.34E+00	7.13E-11	down	-3.03E-01	8.56E-01	no
ENSMUSG00000031996	Aplp2	-2.90E-01	4.29E-01	no	-2.15E+00	6.29E-22	down
ENSMUSG00000032382	Snx1	-2.31E+00	2.43E-19	down	-2.17E+00	7.17E-23	down
ENSMUSG00000036768	Kif15	-2.84E+00	4.40E-06	down	-3.40E+00	9.85E-09	down
ENSMUSG00000015357	Clpx	-3.63E+00	4.88E-35	down	-2.52E+00	9.84E-12	down
ENSMUSG00000046186	Cd109	-3.86E+00	6.77E-05	down	-1.60E+00	5.62E-01	no
ENSMUSG00000003402	Prkcsh	-2.61E-01	5.99E-01	no	-1.96E+00	1.06E-09	down
ENSMUSG00000007656	Arpp19	2.33E+00	1.44E-08	up	1.65E+00	6.58E-06	up
ENSMUSG00000025241	Fyco1	-3.46E+00	2.27E-11	down	-3.42E+00	2.38E-12	down
ENSMUSG00000032178	Ilf3	-1.62E+00	2.20E-05	down	-2.72E+00	1.23E-21	down
ENSMUSG00000095891	Gm10717	6.44E+00	2.76E-14	up	#VALUE!	NA	no
ENSMUSG00000000168	Dlat	-1.93E+00	2.60E-05	down	-2.57E+00	5.57E-14	down
ENSMUSG00000032116	Stt3a	-1.29E+00	4.09E-02	no	-2.44E+00	6.69E-05	down
ENSMUSG00000032050	Rdx	-1.94E+00	6.14E-12	down	-2.09E+00	1.28E-13	down
ENSMUSG00000050471	Fam118b	-1.31E+00	6.66E-05	down	-1.49E+00	1.12E-09	down
ENSMUSG00000032307	Ube2q2	-2.09E+00	3.14E-04	down	-2.32E+00	1.44E-05	down
ENSMUSG00000054792	Klhl18	-1.84E+00	2.05E-01	no	-3.63E+00	1.18E-04	down
ENSMUSG00000032393	Dpp8	-3.53E+00	1.47E-15	down	-3.95E+00	3.70E-29	down
ENSMUSG00000056919	4922501C03Rik	-3.32E+00	5.90E-06	down	-2.53E+00	1.89E-03	no
ENSMUSG00000032594	Ip6k1	-2.94E+00	3.87E-14	down	-2.82E+00	1.62E-10	down
ENSMUSG00000001366	Fbxo9	1.05E+00	1.89E-01	no	-1.42E+00	5.78E-04	down
ENSMUSG00000032301	Psma4	-4.13E-01	2.21E-01	no	-1.32E+00	5.83E-09	down
ENSMUSG00000037287	Tbcel	-2.90E+00	2.00E-04	down	-3.37E+00	1.17E-07	down
ENSMUSG00000043987	Cep164	-1.62E+00	9.52E-07	down	-1.02E+00	2.69E-04	down

ENSMUSG00000079235	Ccdc13	-2.45E+00	8.28E-25	down	-1.84E+00	2.66E-18	down
ENSMUSG00000032204	Aqp9	-2.33E+00	3.48E-06	down	-2.62E+00	3.68E-16	down
ENSMUSG00000032409	Atr	-1.99E+00	1.16E-06	down	-3.78E+00	7.06E-42	down
ENSMUSG00000009741	Ubp1	-2.66E+00	4.94E-15	down	-1.05E+00	9.90E-02	no
ENSMUSG00000084786	Ubl5	1.30E+00	3.00E-06	up	7.61E-01	1.95E-03	no
ENSMUSG00000039977	Ccdc67	-4.07E+00	6.35E-80	down	-1.75E+00	5.82E-07	down
ENSMUSG00000053199	Arhgap20	-2.06E+00	1.30E-01	no	-3.54E+00	4.61E-06	down
ENSMUSG00000090150	Acad11	-1.75E+00	9.83E-02	no	-3.34E+00	1.66E-05	down
ENSMUSG00000035032	Nek11	-3.40E+00	9.78E-08	down	-2.61E+00	1.05E-04	down
ENSMUSG00000033688	1300017J02Rik	-2.87E+00	1.44E-03	no	-2.93E+00	3.41E-04	down
ENSMUSG00000032059	Alg9	-2.46E+00	1.89E-04	down	-2.11E+00	1.30E-04	down
ENSMUSG00000039285	Azi2	-2.99E+00	2.44E-13	down	-2.10E+00	5.53E-04	down
ENSMUSG00000031928	Mre11a	-2.06E+00	1.75E-03	no	-2.43E+00	5.36E-04	down
ENSMUSG00000025234	Arih1	-3.77E+00	3.16E-29	down	-2.68E+00	1.75E-08	down
ENSMUSG00000004661	Arid3b	-2.49E+00	2.40E-05	down	-2.14E+00	8.26E-04	down
ENSMUSG00000032534	Cep63	-3.83E+00	1.96E-62	down	-3.61E+00	5.37E-68	down
ENSMUSG00000032078	Zfp259	-2.05E+00	4.73E-09	down	-1.59E+00	2.76E-07	down
ENSMUSG00000032786	Alas1	-2.25E+00	3.63E-06	down	-2.75E+00	9.18E-13	down
ENSMUSG00000032177	Pde4a	-3.02E+00	7.17E-13	down	1.42E-02	9.91E-01	no
ENSMUSG00000025245	Lztfl1	-2.16E+00	5.50E-04	down	-2.55E+00	1.52E-05	down
ENSMUSG00000040875	Osbpl10	-3.40E+00	5.54E-22	down	-2.61E-01	8.00E-01	no
ENSMUSG00000025237	Parp6	-1.39E+00	9.09E-03	no	-2.52E+00	6.77E-07	down
ENSMUSG00000040325	Vprbp	-3.69E+00	2.90E-14	down	-2.69E+00	8.42E-04	down
ENSMUSG00000032872	Cyb5r4	-3.23E+00	4.70E-04	down	-3.36E+00	1.89E-03	no
ENSMUSG00000002031	Ift46	-1.35E+00	6.31E-04	down	-1.79E+00	8.67E-12	down
ENSMUSG00000032386	Trip4	-2.39E+00	7.43E-04	down	-2.69E+00	8.14E-05	down
ENSMUSG00000031918	Mtmr2	-2.04E+00	7.79E-04	down	-2.52E+00	4.32E-07	down
ENSMUSG00000032297	Celf6	-2.66E+00	6.76E-04	down	6.69E-02	9.79E-01	no
ENSMUSG00000032582	Rbm6	-1.05E+00	2.72E-01	no	-2.35E+00	3.83E-04	down
ENSMUSG00000032489	Kif9	-3.17E+00	1.37E-56	down	-2.59E+00	1.93E-39	down
ENSMUSG00000044791	Setd2	-1.26E+00	1.80E-01	no	-2.90E+00	3.96E-06	down
ENSMUSG00000032514	Ttc21a	-4.39E+00	3.91E-35	down	-2.75E+00	6.73E-09	down
ENSMUSG00000043067	Dpy19l1	-3.37E+00	1.19E-08	down	-2.96E+00	2.38E-06	down
ENSMUSG00000032010	Usp2	-2.04E+00	7.44E-34	down	-9.20E-01	1.89E-08	no
ENSMUSG00000037716	Ccdc33	-3.67E+00	3.48E-20	down	-8.30E-01	3.61E-01	no
ENSMUSG00000032396	Dis3l	-3.69E+00	3.23E-36	down	-3.66E+00	6.61E-63	down
ENSMUSG00000025648	Pfkfb4	-2.95E+00	4.51E-05	down	-3.44E+00	7.63E-08	down
ENSMUSG00000059475	Zfp426	-3.89E+00	1.76E-06	down	-2.70E+00	2.67E-02	no
ENSMUSG00000035934	Pknox2	-4.24E+00	6.87E-13	down	-1.10E+00	4.78E-01	no
ENSMUSG00000032030	Cul5	-2.71E+00	7.70E-07	down	-2.48E+00	8.12E-05	down
ENSMUSG00000032185	Carm1	-4.64E+00	3.56E-12	down	-2.95E+00	3.10E-03	no
ENSMUSG00000031922	Cep57	-2.76E+00	3.45E-36	down	-2.03E+00	8.54E-15	down
ENSMUSG00000059890	Ube4a	-2.54E+00	3.49E-02	no	-3.94E+00	1.53E-05	down
ENSMUSG00000032479	Map4	-3.56E+00	5.30E-18	down	-2.98E+00	2.89E-11	down
ENSMUSG00000032604	Qars	-2.05E+00	5.12E-05	down	-2.08E+00	2.62E-05	down
ENSMUSG00000006676	Usp19	-1.74E+00	7.59E-06	down	-2.46E+00	4.63E-14	down
ENSMUSG00000032525	Nktr	-2.22E+00	9.41E-06	down	-2.57E+00	2.26E-07	down
ENSMUSG00000037801	Iqch	-4.15E+00	2.63E-28	down	-3.39E+00	1.78E-14	down
ENSMUSG00000034135	Sik3	-2.99E+00	4.60E-06	down	-2.71E+00	1.49E-04	down
ENSMUSG00000032400	Zwilch	-7.75E-01	4.01E-01	no	-3.05E+00	2.93E-09	down
ENSMUSG00000047766	Lrrc49	-3.64E+00	1.87E-11	down	-3.23E+00	1.01E-08	down
ENSMUSG00000052698	Tln2	-4.90E+00	2.18E-12	down	-1.93E+00	2.13E-01	no
ENSMUSG00000004099	Dnmt1	-2.47E-01	8.23E-01	no	-2.97E+00	3.45E-07	down
ENSMUSG00000042557	Sin3a	-2.38E+00	4.13E-07	down	-1.89E+00	4.24E-04	down
ENSMUSG00000032064	Dixdc1	-2.67E+00	1.89E-02	no	-3.31E+00	2.10E-04	down
ENSMUSG00000032187	Smarca4	-3.73E-01	3.37E-01	no	-1.43E+00	2.32E-08	down
ENSMUSG00000032058	Ppp2r1b	-2.56E+00	9.94E-13	down	-2.07E+00	1.11E-07	down
ENSMUSG00000032115	Hyou1	-3.12E+00	3.17E-06	down	-5.21E+00	2.23E-35	down
ENSMUSG00000038060	Dlec1	-4.74E+00	4.20E-08	down	-4.41E+00	4.93E-09	down
ENSMUSG00000035919	Bbs9	-2.44E+00	3.97E-03	no	-3.28E+00	1.15E-07	down
ENSMUSG00000040111	Gramd1b	-4.44E+00	4.88E-18	down	-3.88E+00	8.65E-19	down
ENSMUSG00000034908	Sidt2	-2.11E+00	2.05E-05	down	-2.03E+00	1.00E-04	down
ENSMUSG00000032422	Snx14	-2.60E+00	3.09E-03	no	-4.15E+00	1.87E-12	down
ENSMUSG00000039585	Myo9a	-2.10E+00	6.89E-05	down	-2.63E+00	2.26E-08	down

ENSMUSG00000034252	Senp6	-6.99E-01	2.02E-01	no	-1.47E+00	4.37E-04	down
ENSMUSG00000032580	Rbm5	-1.05E-01	9.11E-01	no	-2.26E+00	9.19E-06	down
ENSMUSG00000033335	Dnm2	-1.52E+00	3.28E-10	down	-1.67E+00	7.75E-13	down
ENSMUSG00000040936	Ulk4	-3.59E+00	5.17E-26	down	-2.37E+00	2.03E-11	down
ENSMUSG00000032295	Man2c1	-2.56E+00	2.99E-05	down	-3.04E+00	4.96E-09	down
ENSMUSG00000032480	Dhx30	-2.30E+00	3.91E-10	down	-2.25E+00	7.13E-12	down
ENSMUSG00000032280	Tle3	-3.70E+00	4.37E-06	down	-1.11E+00	5.50E-01	no
ENSMUSG00000041528	Rnf123	-3.07E+00	2.49E-05	down	-2.01E+00	1.56E-03	no
ENSMUSG00000047193	Dync2h1	-1.88E+00	4.61E-03	no	-2.14E+00	2.63E-05	down
ENSMUSG00000045797	4930402K13Rik	-2.88E+00	4.81E-05	down	-8.52E-01	7.13E-01	no
ENSMUSG00000040456	1700054O13Rik	-1.97E+00	5.53E-54	down	-4.51E-01	7.42E-02	no
ENSMUSG00000048994	4930557A04Rik	-2.05E+00	8.21E-33	down	-6.45E-01	8.10E-02	no
ENSMUSG00000095445	Gm14475	-2.51E+00	2.73E-06	down	-2.34E+00	4.76E-02	no
ENSMUSG00000025043	Dusp21	-1.70E+00	1.71E-06	down	5.52E-01	5.65E-01	no
ENSMUSG00000090102	Gm4985	-3.18E+00	1.36E-15	down	1.82E-01	8.79E-01	no
ENSMUSG00000046615	Actrt1	-3.00E+00	6.93E-12	down	-1.62E-01	9.02E-01	no
ENSMUSG00000035651	4930480E11Rik	-4.23E+00	7.77E-11	down	-2.09E+00	1.37E-01	no
ENSMUSG00000078315	Fam47c	-3.01E+00	1.65E-13	down	-3.65E-01	7.73E-01	no
ENSMUSG00000031330	Zcchc13	-2.84E+00	7.55E-26	down	-1.00E+00	6.64E-02	no
ENSMUSG00000083616	H2afb3-ps	-1.15E-01	8.03E-01	no	1.93E+00	1.07E-04	up
ENSMUSG00000049815	4921511C20Rik	-5.34E+00	9.30E-15	down	-3.48E+00	1.88E-02	no
ENSMUSG00000091863	Gm7157	-2.93E+00	1.81E-07	down	-6.86E-01	6.72E-01	no
ENSMUSG00000095597	Gm6472	-1.90E+00	4.32E-124	down	-2.96E+00	0.00E+00	down
ENSMUSG00000090132	Cypt2	5.63E-01	1.42E-08	no	1.47E+00	1.21E-14	up
ENSMUSG00000050435	Ube2dnl1	-1.18E+00	8.17E-04	down	1.08E+00	1.26E-01	no
ENSMUSG00000043569	4930412D23Rik	-2.73E+00	8.14E-15	down	-4.40E-01	6.68E-01	no
ENSMUSG00000071686	Tex13a	-5.39E+00	3.57E-39	down	-3.21E+00	5.36E-06	down
ENSMUSG00000042525	4933428M09Rik	-2.63E+00	7.42E-27	down	-1.37E-01	8.59E-01	no
ENSMUSG00000072100	Gm16390	-1.97E+00	1.42E-05	down	-8.78E-02	9.55E-01	no
ENSMUSG00000057402	1700042B14Rik	-2.01E+00	4.99E-06	down	2.14E-01	8.79E-01	no
ENSMUSG00000078320	Gm362	-4.89E+00	3.59E-17	down	-1.59E+00	2.12E-01	no
ENSMUSG00000073207	Ccdc160	-4.07E+00	2.09E-04	down	-1.73E+00	4.21E-01	no
ENSMUSG00000031194	4931400O07Rik	-1.17E+00	1.83E-04	down	9.86E-01	9.28E-02	no
ENSMUSG00000031182	4930447F04Rik	-2.41E+00	1.26E-10	down	-3.89E-01	7.14E-01	no
ENSMUSG00000025288	4933436I01Rik	-2.76E+00	3.88E-22	down	-4.80E-01	5.55E-01	no
ENSMUSG00000056815	Gm6812	-2.02E+00	1.63E-19	down	-9.06E-01	5.83E-02	no
ENSMUSG00000059690	1700020N15Rik	-2.13E+00	1.48E-21	down	-9.12E-01	5.49E-02	no
ENSMUSG00000071738	Gm4937	-1.81E+00	7.27E-05	down	3.15E-01	8.26E-01	no
ENSMUSG00000035522	Tsga8	-1.07E+00	2.52E-34	down	1.07E+00	2.60E-11	up
ENSMUSG00000079513	4932429P05Rik	-5.76E+00	1.16E-07	down	-3.42E+00	1.33E-01	no
ENSMUSG00000064129	Gm14781	-3.02E+00	8.43E-07	down	-1.57E+00	3.66E-01	no
ENSMUSG00000061392	Mageb5	-2.95E+00	1.16E-05	down	-6.02E-01	7.48E-01	no
ENSMUSG00000096153	BC061195	2.31E+00	2.23E-55	up	8.64E-01	4.34E-02	no
ENSMUSG00000079479	Gm9112	2.99E+00	2.56E-05	up	3.29E+00	9.61E-18	up
ENSMUSG00000079476	1700011M02Rik	2.51E+00	2.17E-06	up	2.77E+00	6.57E-25	up
ENSMUSG00000072995	Cpxcr1	-4.20E+00	1.27E-25	down	-1.79E+00	2.16E-02	no
ENSMUSG00000062791	Gm382	-6.16E+00	1.46E-07	down	-3.03E+00	2.01E-01	no
ENSMUSG00000042750	Bex2	5.49E+00	1.48E-07	up	2.78E+00	9.35E-03	no
ENSMUSG00000051257	Trap1a	2.45E+00	7.76E-06	up	2.44E+00	2.12E-02	no
ENSMUSG00000059663	4930524N10Rik	-2.62E+00	2.97E-08	down	-6.22E-01	6.19E-01	no
ENSMUSG00000025051	Samt4	-3.38E+00	2.02E-05	down	-2.12E+00	2.48E-01	no
ENSMUSG00000031059	Ndufb11	3.12E+00	6.23E-12	up	2.36E+00	1.47E-04	up
ENSMUSG00000095316	Gm21876	-3.09E+00	1.51E-09	down	-1.52E+00	1.72E-01	no
ENSMUSG00000006373	Pgrmc1	1.43E+00	1.03E-07	up	8.08E-01	1.33E-01	no
ENSMUSG00000016427	Ndufa1	3.77E+00	2.53E-53	up	1.59E+00	1.79E-08	up
ENSMUSG00000051038	Rhox11	-2.61E+00	3.39E-06	down	6.95E-01	6.53E-01	no
ENSMUSG00000050197	Rhox13	4.44E+00	1.08E-17	up	3.61E+00	1.51E-04	up
ENSMUSG00000031181	Ctag2	-2.07E+00	1.43E-28	down	3.91E-01	4.16E-01	no
ENSMUSG00000079536	Gm6880	-1.42E+00	2.54E-05	down	8.35E-01	1.95E-01	no
ENSMUSG00000035454	Samt3	-2.82E+00	1.48E-07	down	-1.08E-01	9.48E-01	no
ENSMUSG00000073006	Gm732	-4.75E+00	2.14E-17	down	-1.56E+00	1.56E-01	no
ENSMUSG00000058670	4932411N23Rik	-4.62E+00	3.37E-06	down	-1.97E+00	4.91E-01	no
ENSMUSG00000031270	4930513O06Rik	-1.12E+00	2.05E-04	down	1.05E+00	7.97E-02	no
ENSMUSG00000048573	Cypt3	1.48E+00	2.41E-64	up	1.71E+00	6.15E-22	up

ENSMUSG00000064137	Rhox8	3.78E+00	6.61E-03	no	3.50E+00	2.05E-07	up
ENSMUSG00000060673	4930595M18Rik	-3.22E+00	6.37E-07	down	-1.18E+00	4.91E-01	no
ENSMUSG00000073001	Cylc1	-3.80E+00	6.71E-58	down	-1.57E+00	4.64E-04	down
ENSMUSG00000042712	Wbp5	4.39E+00	2.38E-13	up	2.34E+00	9.72E-05	up
ENSMUSG00000068113	Gm4907	-2.51E+00	9.83E-11	down	-2.09E-01	8.70E-01	no
ENSMUSG00000073085	4930428D18Rik	-2.38E+00	2.99E-11	down	3.61E-01	7.11E-01	no
ENSMUSG00000036551	Akap14	-1.69E+00	2.29E-04	down	7.78E-01	5.16E-01	no
ENSMUSG00000031231	Cox7b	3.00E+00	5.25E-04	up	1.67E+00	2.50E-01	no
ENSMUSG00000031384	Asb9	-2.38E+00	5.36E-25	down	9.48E-03	9.89E-01	no
ENSMUSG00000079583	Gm7073	-3.04E+00	5.11E-18	down	-8.76E-01	3.06E-01	no
ENSMUSG00000079460	4933403O08Rik	-4.86E+00	3.78E-05	down	-1.75E+00	5.84E-01	no
ENSMUSG00000060726	Tmsb15a	-1.23E+00	1.89E-08	down	3.91E-01	4.44E-01	no
ENSMUSG00000055357	4933400A11Rik	-4.10E+00	1.37E-22	down	-1.62E+00	4.08E-02	no
ENSMUSG00000037636	Slc25a43	3.51E+00	1.16E-04	up	3.56E+00	7.04E-02	no
ENSMUSG00000016319	Slc25a5	4.02E+00	4.10E-14	up	3.24E+00	6.60E-07	up
ENSMUSG00000031118	1700080O16Rik	-1.41E+00	3.10E-07	down	8.44E-01	1.74E-01	no
ENSMUSG00000031179	3830417A13Rik	-3.18E+00	9.58E-11	down	-3.48E-01	8.23E-01	no
ENSMUSG00000002015	Bcap31	2.54E+00	1.34E-11	up	2.94E+00	1.33E-05	up
ENSMUSG00000049775	Tmsb4x	4.36E+00	9.41E-201	up	2.56E+00	8.45E-23	up
ENSMUSG00000025630	Hprt	4.27E+00	9.58E-08	up	2.48E+00	3.94E-02	no
ENSMUSG00000052549	Arl13a	-4.54E+00	8.00E-16	down	-2.55E+00	5.88E-02	no
ENSMUSG00000058252	1700008I05Rik	-1.92E+00	1.53E-12	down	7.94E-01	1.85E-01	no
ENSMUSG00000050071	Bex1	4.96E+00	1.27E-04	up	4.91E+00	1.48E-02	no
ENSMUSG00000025257	Ribc1	-2.01E+00	1.38E-16	down	7.37E-01	1.81E-01	no
ENSMUSG00000046432	Ngfrap1	3.68E+00	5.44E-08	up	2.13E+00	3.64E-02	no
ENSMUSG00000090141	Gm614	-5.68E+00	1.34E-12	down	-3.90E+00	9.04E-03	no
ENSMUSG00000056537	Rlim	-2.48E+00	2.75E-04	down	-4.75E-01	8.10E-01	no
ENSMUSG00000025527	Satl1	-5.43E+00	6.41E-17	down	-2.61E+00	4.63E-02	no
ENSMUSG00000031411	Prame	-3.33E+00	2.47E-05	down	-3.33E-01	8.88E-01	no
ENSMUSG00000031430	Vsig1	-2.62E+00	3.53E-12	down	-1.67E-01	8.75E-01	no
ENSMUSG00000073094	Smim9	-3.33E+00	3.65E-04	down	-4.79E-01	8.47E-01	no
ENSMUSG00000031158	Timm17b	1.57E+00	4.85E-04	up	2.43E+00	3.69E-03	no
ENSMUSG00000034403	Pja1	-1.65E+00	2.71E-04	down	2.04E-02	9.87E-01	no
ENSMUSG00000073130	Gm1141	-4.37E+00	6.39E-39	down	-1.44E+00	6.48E-02	no
ENSMUSG00000051159	Cited1	4.54E+00	2.43E-09	up	2.52E+00	3.10E-04	up
ENSMUSG00000025038	Efhc2	-3.28E+00	6.23E-11	down	-8.97E-01	5.43E-01	no
ENSMUSG00000062170	Fmr1nb	2.53E+00	9.94E-29	up	2.26E+00	4.56E-07	up
ENSMUSG00000031431	Tsc22d3	4.25E+00	1.33E-07	up	4.00E+00	5.06E-03	no
ENSMUSG00000079584	Gm364	1.69E+00	2.49E-05	up	1.05E+00	2.37E-01	no
ENSMUSG00000035232	Pdk3	-1.64E+00	1.92E-08	down	3.94E-01	6.34E-01	no
ENSMUSG00000036013	Fam122c	-1.68E+00	3.61E-16	down	5.19E-01	2.60E-01	no
ENSMUSG00000031397	Tktl1	2.79E+00	3.54E-05	up	2.54E+00	5.23E-02	no
ENSMUSG00000050089	Akap4	-5.73E+00	0.00E+00	down	-3.13E+00	2.66E-39	down
ENSMUSG00000079606	Gm595	-2.72E+00	1.15E-28	down	-3.20E-01	6.77E-01	no
ENSMUSG00000073007	Fam46d	-3.73E+00	3.99E-15	down	-1.77E+00	9.57E-02	no
ENSMUSG00000067377	Tspan6	-3.53E+00	3.22E-20	down	-2.66E+00	2.41E-05	down
ENSMUSG00000031292	Cdkl5	-4.12E+00	7.50E-07	down	-1.81E+00	4.40E-01	no
ENSMUSG00000067878	Mtap7d3	-3.25E+00	3.03E-09	down	-8.01E-01	6.17E-01	no
ENSMUSG00000025529	Zfp711	-2.54E+00	7.99E-05	down	-6.59E-01	7.44E-01	no
ENSMUSG00000062168	Ppef1	-4.71E+00	6.64E-10	down	-2.70E+00	1.39E-01	no
ENSMUSG00000009596	Taf7l	1.45E+00	2.21E-08	up	2.29E+00	2.91E-05	up
ENSMUSG00000025283	Sat1	4.05E+00	3.03E-05	up	2.02E+00	2.28E-01	no
ENSMUSG00000031157	Pqbp1	4.16E+00	6.57E-05	up	2.45E+00	1.35E-01	no
ENSMUSG00000002014	Ssr4	3.99E+00	6.36E-07	up	2.58E+00	3.21E-02	no
ENSMUSG00000060090	Rp2h	-3.21E+00	1.08E-05	down	-1.32E+00	4.37E-01	no
ENSMUSG00000031375	Bgn	3.55E+00	3.74E-07	up	2.48E+00	3.30E-01	no
ENSMUSG00000031358	Msl3	-3.00E+00	2.56E-04	down	-4.15E-01	8.59E-01	no
ENSMUSG00000025287	Acot9	-1.59E+00	1.56E-06	down	5.29E-01	5.63E-01	no
ENSMUSG00000031351	Zfp185	-3.67E+00	2.45E-04	down	-1.57E+00	5.27E-01	no
ENSMUSG00000031353	Rbbp7	3.27E+00	2.20E-13	up	1.82E+00	2.52E-02	no
ENSMUSG00000031170	Slc38a5	4.99E+00	6.63E-09	up	3.19E+00	1.79E-01	no
ENSMUSG00000002010	Idh3g	2.89E+00	5.20E-05	up	2.73E+00	2.46E-02	no
ENSMUSG00000031065	Cdk16	-2.10E+00	5.44E-07	down	-4.53E-01	7.06E-01	no
ENSMUSG00000019087	Atp6ap1	3.65E+00	1.50E-05	up	1.88E+00	1.31E-01	no

ENSMUSG00000031256	Cstf2	-3.10E+00	3.53E-05	down	-1.22E+00	5.62E-01	no
ENSMUSG00000001924	Uba1	-2.63E+00	4.48E-12	down	-4.75E-01	6.74E-01	no
ENSMUSG00000025268	Maged2	3.18E+00	5.45E-04	up	1.06E+00	3.99E-01	no
ENSMUSG00000031095	Cul4b	-3.79E+00	6.19E-19	down	-1.49E+00	8.26E-02	no
ENSMUSG00000041718	Alg13	-2.31E+00	6.53E-04	down	-1.52E-01	9.50E-01	no
ENSMUSG00000025862	Stag2	-3.52E+00	9.06E-05	down	-1.32E+00	5.87E-01	no
ENSMUSG00000031174	Rpgr	-3.62E+00	1.45E-07	down	-1.54E+00	4.31E-01	no
ENSMUSG00000031392	Irak1	5.13E+00	5.10E-04	up	3.42E+00	1.33E-01	no
ENSMUSG00000069049	Eif2s3y	-1.75E+00	3.45E-05	down	-4.87E-01	7.35E-01	no
